# Murine Broadly Reactive Antineuraminidase Monoclonal Antibodies Protect Mice from Recent Influenza B Virus Isolates and Partially Inhibit Virus Transmission in the Guinea Pig Model

**DOI:** 10.1128/msphere.00927-21

**Published:** 2022-09-07

**Authors:** Jessica Tan, Veronika Chromikova, George O'Dell, Emilia Mia Sordillo, Viviana Simon, Harm van Bakel, Florian Krammer, Meagan McMahon

**Affiliations:** a Department of Microbiology, Icahn School of Medicine at Mount Sinai, New York, New York, USA; b Graduate School of Biomedical Sciences, Icahn School of Medicine at Mount Sinai, New York, New York, USA; c Department of Pathology, Icahn School of Medicine at Mount Sinai, New York, New York, USA; d Global Health and Emerging Pathogens Institute, Icahn School of Medicine at Mount Sinai, New York, New York, USA; e Division of Infectious Diseases, Department of Medicine, Icahn School of Medicine at Mount Sinai, New York, New York, USA; f Department of Pathology, Molecular and Cell-Based Medicine, Icahn School of Medicine at Mount Sinai, New York, New York, USA; g Department of Genetics and Genomic Sciences, Icahn School of Medicine at Mount Sinai, New York, New York, USA; h Icahn Institute for Data Science and Genomic Technology, Icahn School of Medicine at Mount Sinai, New York, New York, USA; Emory University School of Medicine

**Keywords:** Influenza, experimental therapeutics, monoclonal antibodies

## Abstract

Current influenza virus vaccines and antivirals have limitations, some of which disproportionately affect their utilization against influenza B viruses. To inform ongoing efforts to address the considerable global burden of influenza B viruses, we previously described five murine monoclonal antibodies that broadly bind conserved epitopes on the neuraminidase of influenza B viruses and protect against lethal challenge in a mouse model when delivered via intraperitoneal injection. Here, we validate the continued relevance of these antibodies by demonstrating that their protective effects extend to lethal challenge with mouse-adapted influenza B viruses recently isolated from humans. We also found that humanization of murine antibodies 1F2 and 4F11 resulted in molecules that retain the ability to protect mice from lethal challenge when administered prophylactically. Intranasal administration as an alternative route of 1F2 delivery revealed no differences in the mouse challenge model compared to intraperitoneal injection, supporting further assessment of this more targeted and convenient administration method. Lastly, we evaluated the potential for intranasal 1F2 administration initiated 1 day after infection to prevent transmission of an influenza B virus between cocaged guinea pigs. Here, we observed a 40% rate of transmission with the 1F2 antibody administered to the infected donor compared to 100% transmission with administration of an irrelevant control antibody. These data suggest that intranasal administration could be a viable route of administration for antibody therapeutics. Collectively, these findings demonstrate the potential of broad antineuraminidase antibodies as therapeutics to prevent and treat infections caused by influenza B viruses.

**IMPORTANCE** The global health burden of influenza B viruses, especially in children, has long been underappreciated. Although two antigenically distinct influenza B virus lineages cocirculated before the coronavirus disease 2019 (COVID-19) pandemic, the commonly used trivalent seasonal vaccines contain antigens from only one influenza B virus, providing limited cross-protection against viruses of the other lineage. Additionally, studies have called into question the clinical effectiveness of the neuraminidase inhibitors that comprise the majority of available antivirals in treating influenza B virus infections. We previously described antibodies that bind broadly to neuraminidases of influenza B viruses across decades of antigenic evolution and potently protect mice against lethal challenge. Here we appraise additional factors to develop these antineuraminidase antibodies as antivirals to prevent and treat infections caused by an extensive range of influenza B viruses. In addition this work assesses recent clinical isolates belonging to the two influenza B virus lineages, finding evidence supporting the development of these antibodies for prophylactic and therapeutic use.

## INTRODUCTION

While a large proportion of influenza virus research focuses on influenza A viruses (IAVs), the burden of influenza B viruses (IBVs) can no longer be overlooked. The larger proportion of human influenza virus infections attributable to IAVs and their pandemic potential have contributed to this greater focus on IAVs. Recently, however, scientific evidence has conclusively demonstrated the importance of IBV infections as a global health concern, combatting the longstanding misperception of IBVs’ negligible impact (1, 2). The increasing value placed on protecting against IBV infection has driven an ongoing shift to use of quadrivalent seasonal influenza virus vaccines that include both a B/Yamagata/16/88-like lineage and a B/Victoria/2/87-like lineage vaccine strain in addition to H1 and H3 influenza A virus strains.

Influenza virus vaccination remains the uncontested mainstay for minimizing the impact of influenza-associated morbidity and mortality. Because two antigenically distinct lineages of IBVs cocirculated with IAVs before the coronavirus disease 2019 (COVID-19) pandemic, the B/Yamagata/16/88-like lineage and the B/Victoria/2/87-like lineage, trivalent influenza virus vaccines (containing antigens from one H1N1 virus, one H3N2 and one IBV from either lineage) provided limited cross-protection against viruses of the IBV lineage not included in the vaccine. Quadrivalent vaccines that include the additional IBV lineage have been developed and are in use in some countries to provide broader protection (3). However, countries continue to consider the cost-effectiveness and fiduciary impact of increasing vaccine valency (2, 4, 5). Increasing valency also does not alleviate other important shortcomings that may leave even routinely vaccinated individuals vulnerable to influenza, such as antigenic differences between vaccine strains and circulating viruses, as well as the transient nature of vaccine-induced protection (6).

Individuals infected with IBVs may also respond less favorably to antivirals than individuals infected with IAVs. Some studies have indicated that oseltamivir, the prototypic neuraminidase (NA) inhibitor, and other drugs in this class may have less clinical efficacy against IBV infections compared to IAV infections (7, 8). More recently, a first-in-class influenza antiviral that targets the viral cap-dependent endonuclease, baloxavir marboxil (hereafter referred to as baloxavir), has been approved by the US Food and Drug Administration (FDA) for the treatment and chemoprophylaxis of influenza for individuals ages 12 and older. However, *in vitro* assays have suggested that higher concentrations of baloxavir are needed to achieve inhibition of IBVs than are needed to inhibit IAVs (9). Although baloxavir enhances the ability to prevent and treat influenza, perhaps especially in the context of IBV infections, current influenza antivirals still have a number of significant drawbacks. Like all drugs, influenza antivirals may cause adverse events and cannot be administered to individuals with contraindications. Furthermore, like many other antivirals, the NA inhibitors and baloxavir remain useful only while circulating viruses remain susceptible. As drug-resistant viruses emerge and become widespread, the cost-to-benefit ratio of drug administration no longer justifies a drug’s use, as exemplified by the adamantane drugs, amantadine and rimantidine, previously used for the treatment and prevention of IAV infections (10, 11). Additionally, while all data currently available regarding baloxavir’s clinical efficacy are limited to studies in which treatment was administered within 48 h of symptom onset, the importance of early administration of small-molecule NA inhibitors on their clinical benefit in treating influenza has long been known and informs the FDA approval of antivirals for use within 2 days of symptom onset (12).

We previously described five murine monoclonal antibodies (mu-MAbs) that may address some of the unmet needs for IBV antivirals (13). These mu-MAbs were shown to bind to and inhibit the activity of IBV NAs spanning more than 70 years of antigenic drift. Furthermore, they provided *in vivo* protection against lethal challenge when administered prophylactically and therapeutically to mice. One of these mu-MAbs, 1F2, was also found to provide protection superior to that of oseltamivir when administered to mice 72 h after infection with a lethal dose of influenza virus. Only the intraperitoneal (IP) route of administration was tested for these mu-MAbs previously, but studies of targeted delivery by intranasal (IN) or aerosol/nebulizer administration of broadly neutralizing hemagglutinin (HA)-targeting antibodies in animal models showed increased benefits compared to the systemic delivery methods of IP and intravenous injections (14, 15).

Here, we evaluated essential factors that affect the translation of these mu-MAbs into clinical use. We found that four of the five mu-MAbs demonstrate prophylactic protection against lethal challenge with clinical influenza viruses isolated in 2017 and 2018. Additionally, prophylactic and therapeutic administration of humanized versions of two of these mu-MAbs, 1F2 and 4F11, in the setting of lethal IBV challenge in mice, provided protection comparable to the original mu-MAbs. IN administration of 1F2 had similar protective effects as IP administration in the mouse challenge model, and reduced transmission of IBV between cocaged guinea pigs when administered IN to animals that were directly infected.

## RESULTS

### mu-MAbs protect mice from challenge with recent IBV isolates.

We first tested the activity spectrum of these mu-MAbs against contemporary IBVs circulating and causing disease in our New York City metropolitan community. Two human viral isolates, B/New York City/PV00094/2017 (GenBank MW651803), a B/Yamagata/16/88-like lineage virus, and B/New York City/PV01181/2018 (GenBank MW651780), a B/Victoria/2/87-like lineage virus, were obtained through the Mount Sinai Pathogen Surveillance Program and serially passaged in DBA/2J mice for mouse adaptation. The isolates were passaged in mice, with lungs harvested and homogenized 4 days postinfection to serve as the inoculum for the next passage. The lung homogenates from the fifth passages were used to infect eggs. Lung-to-lung passaging through five mice succeeded with the B/New York City/PV01181/2018 virus but an attempt to directly passage the B/New York City/PV00094/2017 virus through a fifth mouse failed to yield detectable levels of virus in the lungs of that mouse. The lung homogenate from the fourth passage was amplified in eggs to generate an inoculum containing 10^6^ plaque forming units (PFU) of virus to infect another mouse, which yielded a high viral lung titer. The mouse-passaged variants of both viruses were tested for virulence in the BALB/c mouse model and were compared to nonmouse-passaged viruses that were grown in Madin Darby canine kidney (MDCK) cells. Serial passaging through five mice allowed sufficient adaptation of the B/New York City/PV00094/2017 virus such that it caused significant weight loss at a dose of 10^4^ PFU and lethality in all mice challenged with 10^5^ PFU (Fig. S1A). In contrast, no morbidity was observed in mice challenged with the MDCK-grown B/New York CityPV00094/2017 virus (Fig. S1B). The B/New York City/PV01181/2018 isolate was already virulent in mice prior to serial passaging and showed no increased virulence after passaging, with both variants lethal at doses greater than or equal to 10^3^ PFU (Fig. S1C and S1D). Sequence changes between the mouse-adapted and original isolates are also detailed in Table 1.

**TABLE 1:** Sequence differences between original and mouse-adapted B/New York City/PV01181/2018 and B/New York City/PV00094/2017 viruses

Segment	B/New York city/PV01181/2018	B/New York city/PV00094/2017
Segment 1 – PB2	No changes	K54R
Segment 2 – PB1	K157R E527G	No changes
Segment 3 – PA	No changes	No changes
Segment 4 – HA	T213I	N211D
Segment 5 – NP	No changes	No changes
Segment 6 – NA/NB	No changes	No changes
Segment 7 – M1/BM2	No changes	No changes
Segment 8 – NS1	No changes	No changes

10.1128/msphere.00927-21.2 FIG S1 Mouse lethality of recent influenza B virus isolates. Mice were IN infected with 10^5^, 10^4^, 10^3^, 10^2^, or 10^1^ PFU of mouse-passaged B/New York City/PV00094/2017 influenza virus (A) or B/New York City/PV01181/2018 influenza virus (B). Mice were IN infected with 10^6^, 10^5^, or 10^4^ PFU of cell-grown B/New York City/PV00094/2017 influenza virus (C) or 10^6^, 10^5^, 10^4^, 10^3^, or 10^2^ PFU of B/New York City/PV01181/2018 influenza virus (D). Weight loss was monitored for 14 days post challenge and any mouse that lost more than 25% of its initial body weight was humanely culled. Survival is indicated in parentheses. The calculated mLD_50_ values are indicated where applicable. Download FIG S1, TIF file, 0.2 MB. Copyright © 2022 Tan et al. 2022 Tan et al. https://creativecommons.org/licenses/by/4.0/ This content is distributed under the terms of the Creative Commons Attribution 4.0 International license.

**FIG 1:**
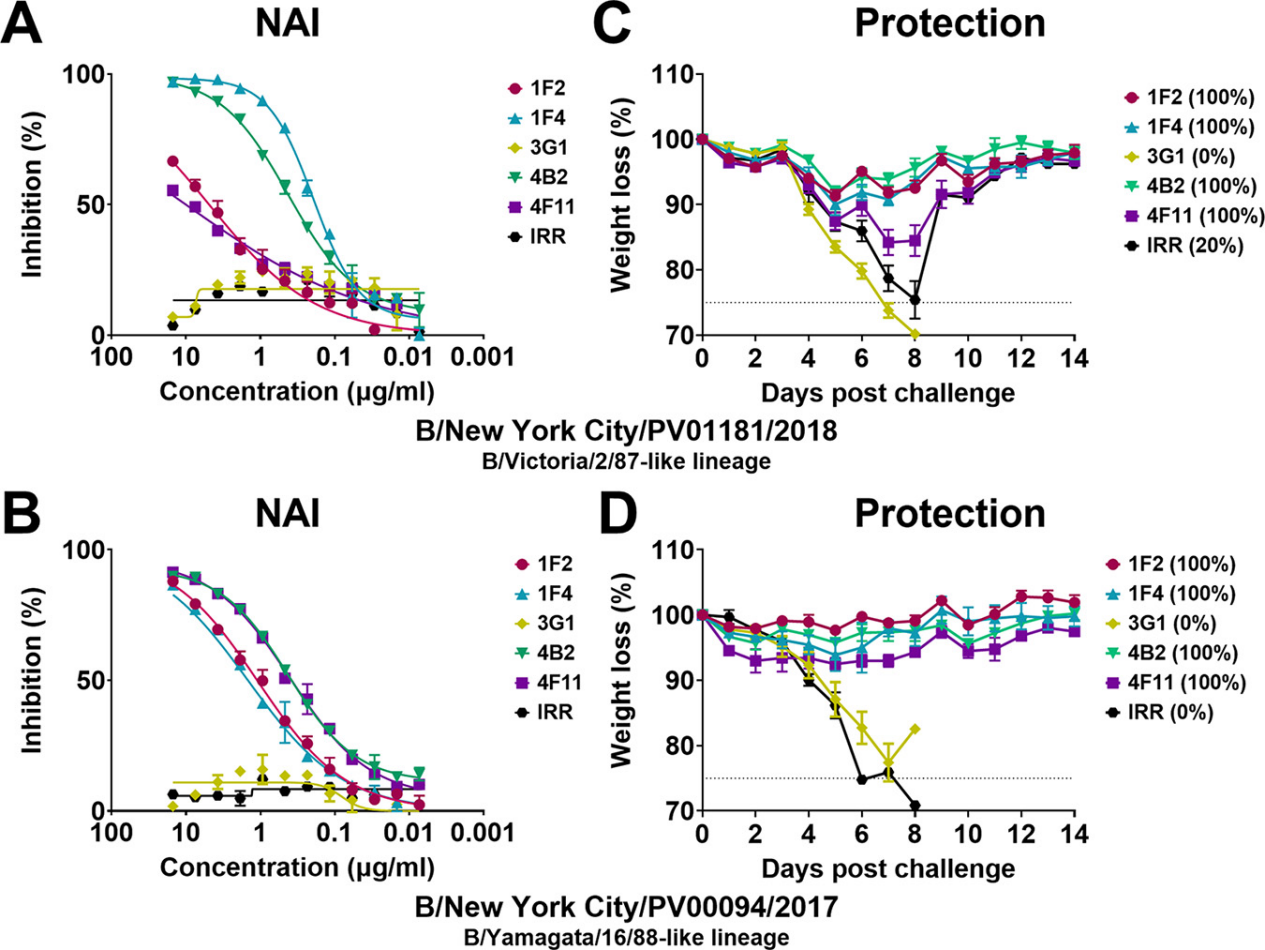
Four broad anti-IBV NA mu-MAbs inhibit and protect against recent mouse-adapted IBV isolates. NAI against B/New York City/PV01181/2018 (A) and B/New York City/PV00094/2017 (B) by 1F2, 1F4, 3G1, 4B2, 4F11, and an irrelevant control mu-MAb. *In vivo* protection against lethal challenge with B/New York City/PV01181/2018 (C) and B/New York City/PV00094/2017 (D) by 1F2, 1F4, 3G1, 4B2, 4F11, or an irrelevant control when mu-MAbs were administered 2 h prior to challenge. Weight loss was monitored for 14 days postchallenge and any mouse that lost more than 25% of its initial body weight was humanely culled. Survival is indicated in parentheses.

Next, we tested the five mu-MAbs previously shown to bind broadly to IBV NAs (13) — named 1F2, 1F4, 3G1, 4B2, and 4F11 — for NA inhibition (NAI) activity using established enzyme-linked lectin assays (ELLAs) against mouse-adapted B/New York City/PV01181/2018 (Fig. 1A) and mouse-adapted B/New York City/PV00094/2017 (Fig. 1B). Excluding 3G1, which was previously found to have the narrowest binding profile (13), the tested anti-NA mu-MAbs exhibited potent NAI activity against recent IBVs. Following *in vitro* characterization, we tested the *in vivo* prophylactic potential of the mu-MAbs in the context of lethal challenge with mouse-adapted versions of B/New York City/PV01181/2018 and B/New York City/PV00094/2017 in a well-established influenza virus challenge system. Female BALB/c mice were administered 10 mg/kg of mu-MAb IP 2 h prior to challenge with 5× the median lethal dose (5xmLD_50_) of mouse-adapted B/New York City/PV01181/2018 (Fig. 1C) or mouse-adapted B/New York City/PV00094/2017 (Fig. 1D). Other than 3G1, the MAbs protected against morbidity at varying degrees, as measured by weight loss, and protected fully against mortality (indicated in parentheses).

### Humanized versions of MAbs 1F2 and 4F11 protect against IBV challenge following IP injection.

**FIG 2:**
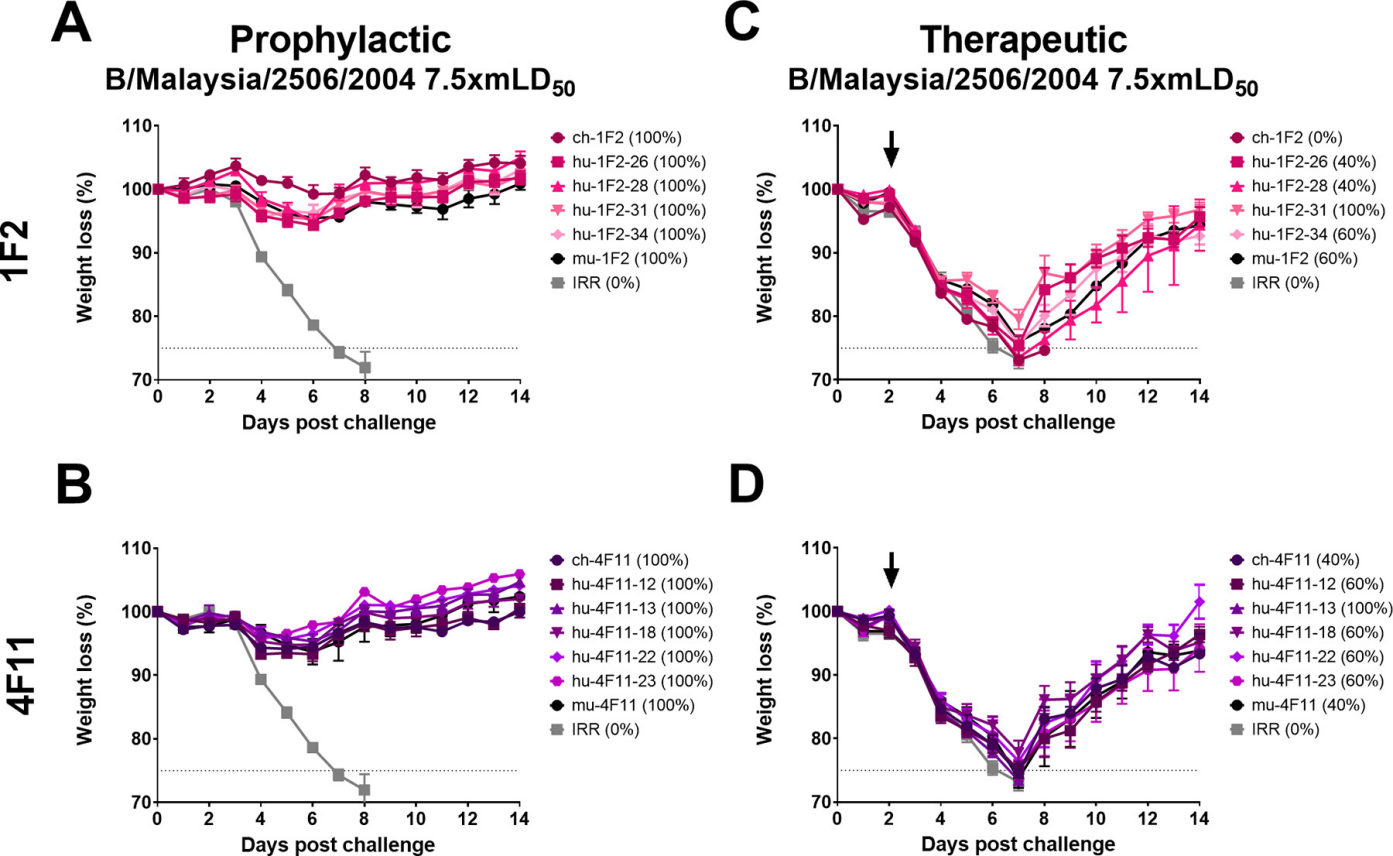
Protection provided by humanized 1F2 and 4F11 in a prophylactic and therapeutic setting. Mice were IP administered at 5 mg/kg of humanized 1F2 (A) or 4F11 (B) 2 h prior to IN challenge with 7.5xmLD_50_ of B/Malaysia/2506/2004. Mice were IP administered humanized 5 mg/kg of humanized 1F2 (C) or 4F11 (D) 48 h after IN challenge with 7.5xmLD_50_ of B/Malaysia/2506/2004 as indicated by the black arrows. Weight loss was monitored for 14 days postchallenge and any mouse that lost more than 25% of its initial body weight was humanely culled. Survival is indicated in parentheses.

Two anti-IBV NA MAbs, 1F2 and 4F11, were selected for humanization based on their previously described binding profiles (13), where 1F2 and 4F11 were shown to have a broader binding profile and binding strength compared to 1F4 and 4B2, as well as their potent protection against recent IBV isolates (Fig. 1). After an initial screening for NAI activity was conducted on the candidate humanized antibodies (hu-MAbs) (Fig. S2 and S3), four 1F2 hu-MAbs and five 4F11 hu-MAbs were tested in mouse challenge experiments along with the parental mu-MAb and a chimerized version (ch-MAb) with 7.5xmLD_50_ of B/Malaysia/2506/2004, the virus used in the original study (13). We delivered 5 mg/kg of the MAbs through IP injection 2 h prior to infection to test prophylactic administration or 48 h after infection to test therapeutic administration. In these studies (and subsequent experiments) we elected to use the B/Malaysia/2506/2004 strain as it is well-characterized in the mouse and guinea pig models. We also used 5 mg/kg for the humanized MAb studies as we have previously used this dose to successfully protect mice from challenge in other NA MAb studies (16) allowing us to use MAb sparingly. While all selected hu-MAbs conferred protection against morbidity and mortality when administered prophylactically (Fig. 2A and B), they did not fully protect against weight loss or death (indicated in parentheses) in the therapeutic setting (Fig. 2C and D). Only hu-1F2-31 and hu-4F11-13 protected 100% of the mice from mortality (indicated in parenthesis), although the mice lost an average of 20% of their initial body weight (Fig. 1C and D).

10.1128/msphere.00927-21.3 FIG S2 NAI by 1F2 humanized variants. NAI of B/Wisconsin/1/2010, B/Malaysia/2506/2004, B/Yamagata/16/88, B/New York City/PV01181/2018 and B/New York City/PV00094/2017 by 1F2 humanized variant MAbs and the mouse hybridoma and chimeric equivalents. The * represents humanized MAbs and chimerized MAbs that were selected for characterization *in vivo*. Download FIG S2, TIF file, 0.1 MB. Copyright © 2022 Tan et al. 2022 Tan et al. https://creativecommons.org/licenses/by/4.0/ This content is distributed under the terms of the Creative Commons Attribution 4.0 International license.

10.1128/msphere.00927-21.4 FIG S3 NAI by 4F11 humanized variants. NAI of B/Wisconsin/1/2010, B/Malaysia/2506/2004, B/Yamagata/16/88, B/New York City/PV01181/2018 and B/New York City/PV00094/2017 by 4F11 humanized variant MAbs and the mouse hybridoma and chimeric equivalents. The * represents humanized MAbs and chimerized MAbs that were selected for characterization *in vivo*. Download FIG S3, TIF file, 0.1 MB. Copyright © 2022 Tan et al. 2022 Tan et al. https://creativecommons.org/licenses/by/4.0/ This content is distributed under the terms of the Creative Commons Attribution 4.0 International license.

### IN administration of mouse MAbs is as protective as IP administration.

**FIG 3:**
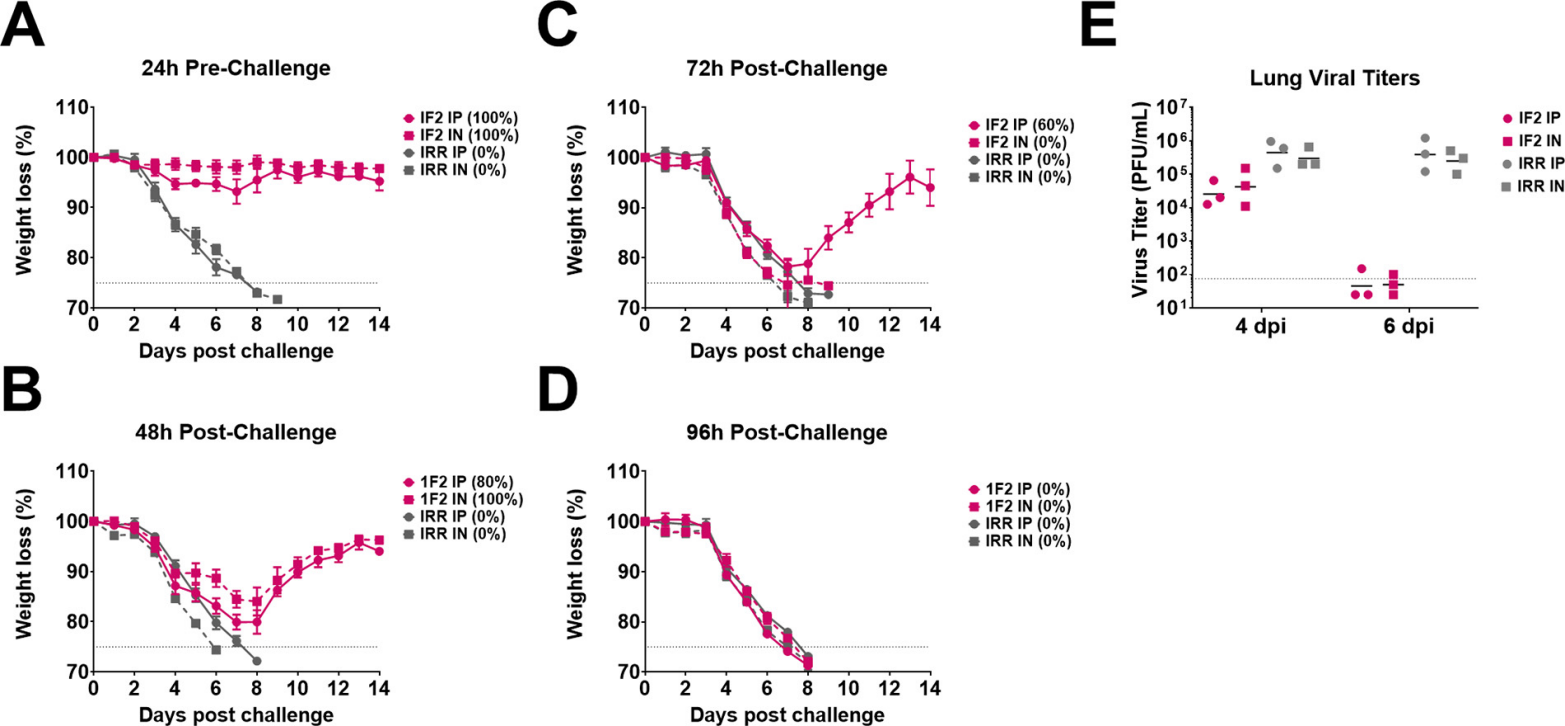
IP and IN administration of 1F2 have similar protective capacities against lethal challenge in both prophylactic and therapeutic settings. Mice were IP administered 10 mg/kg of mu-MAb 1F2 or an irrelevant control mu-MAb delivered IN or IP at 24 h prior to IN challenge with 5xmLD_50_ of B/Malaysia/2506/2004 virus (A). Mice were administered 10 mg/kg of 1F2 or an irrelevant control mu-MAb delivered IN or IP at 48 (B), 72 (C), or 96 (D) hours post-IN challenge with 5xmLD_50_ of B/Malaysia/2506/2004 virus. Weight loss was monitored for 14 days postchallenge and any mouse that lost more than 25% of its initial body weight was humanely culled. Survival is indicated in parentheses. Mice were IP administered 10 mg/kg of 1F2 or an irrelevant control mu-MAb delivered IN or IP at 48 h post IN challenge with 1xmLD_50_ of B/Malaysia/2506/2004 virus and lungs were harvested at 4 or 6 days postchallenge for determining virus titers via plaque assay (E).

One anti-IBV NA mu-MAb, 1F2, was selected to compare IP and IN administration in the mouse model, as we were interested in determining the relevance of the IN route in this context. mu-MAb 1F2 was delivered at 10 mg/kg prophylactically 24 h prior to challenge or therapeutically 48, 72, or 96 h after challenge with 5xmLD_50_ of B/Malaysia/2506/2004. This mu-MAb provided similar levels of protection against both morbidity and mortality when administered either IP or IN prophylactically (Fig. 3A). With 1F2 administration at 48 h postchallenge, 4 out of 5 mice were protected from mortality in the IP group, while all the animals survived in the IN group. The morbidity appeared comparable (Fig. 3B). When mu-1F2 was administered therapeutically 72 h after challenge, 60% of the mice receiving IP mu-1F2 survived compared to 0% of the IN-treated mice (Fig. 3C). At 96 h postinfection, neither IP nor IN mu-1F2 treatment were protective from lethal challenge (Fig. 3D).

We also assessed differences in lung viral titers at days 4 and 6 postchallenge when mu-MAb was administered 48 h after challenge with 1xmLD_50_ of B/Malaysia/2506/2004. No detectable differences in viral titers were measured in lung homogenates at 4 and 6 days postchallenge (Fig. 3E).

**FIG 4:**
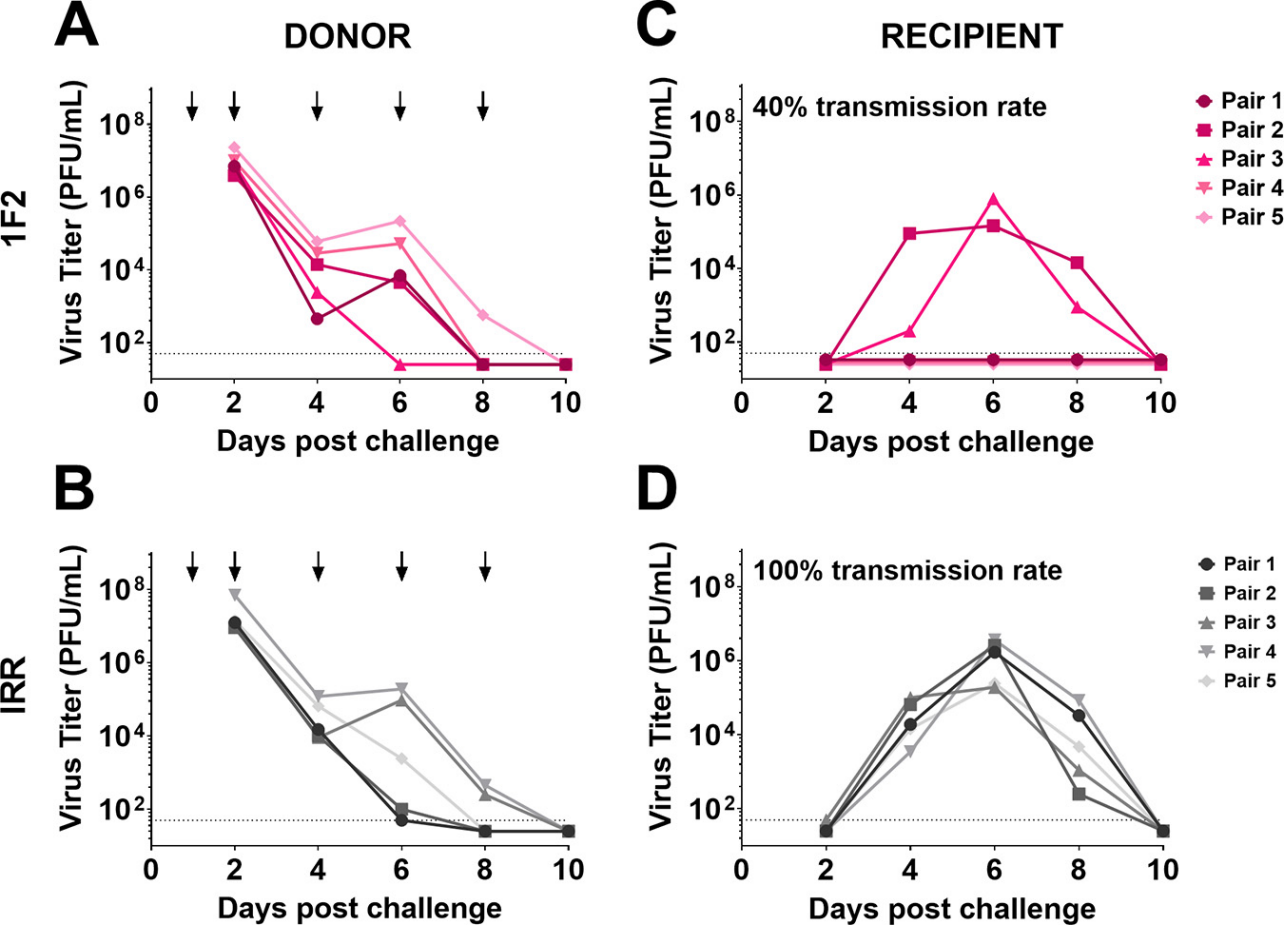
Anti-NA mu-MAb 1F2 administration to donors reduces transmission to recipients in a direct contact setting. Donor guinea pigs IN infected with 10^4^ PFU of B/Malaysia/2506/2004 virus were IN administered 1F2 (A) or an irrelevant (IRR) control mu-MAb (B) at 10 mg/kg on days 1, 2, 4, 6, and 8 postinfection. Nasal washes were collected at days 2, 4, 6, 8, and 10 post initial challenge and viral titers were measured via plaque assay. Transmission to cocaged recipients from 1F2-administered donors (C) or IRR-administered donors (D) was assessed by collecting nasal washes at 2, 4, 6, 8, and 10 days post initial challenge and measuring viral titers via plaque assay.

**FIG 5:**
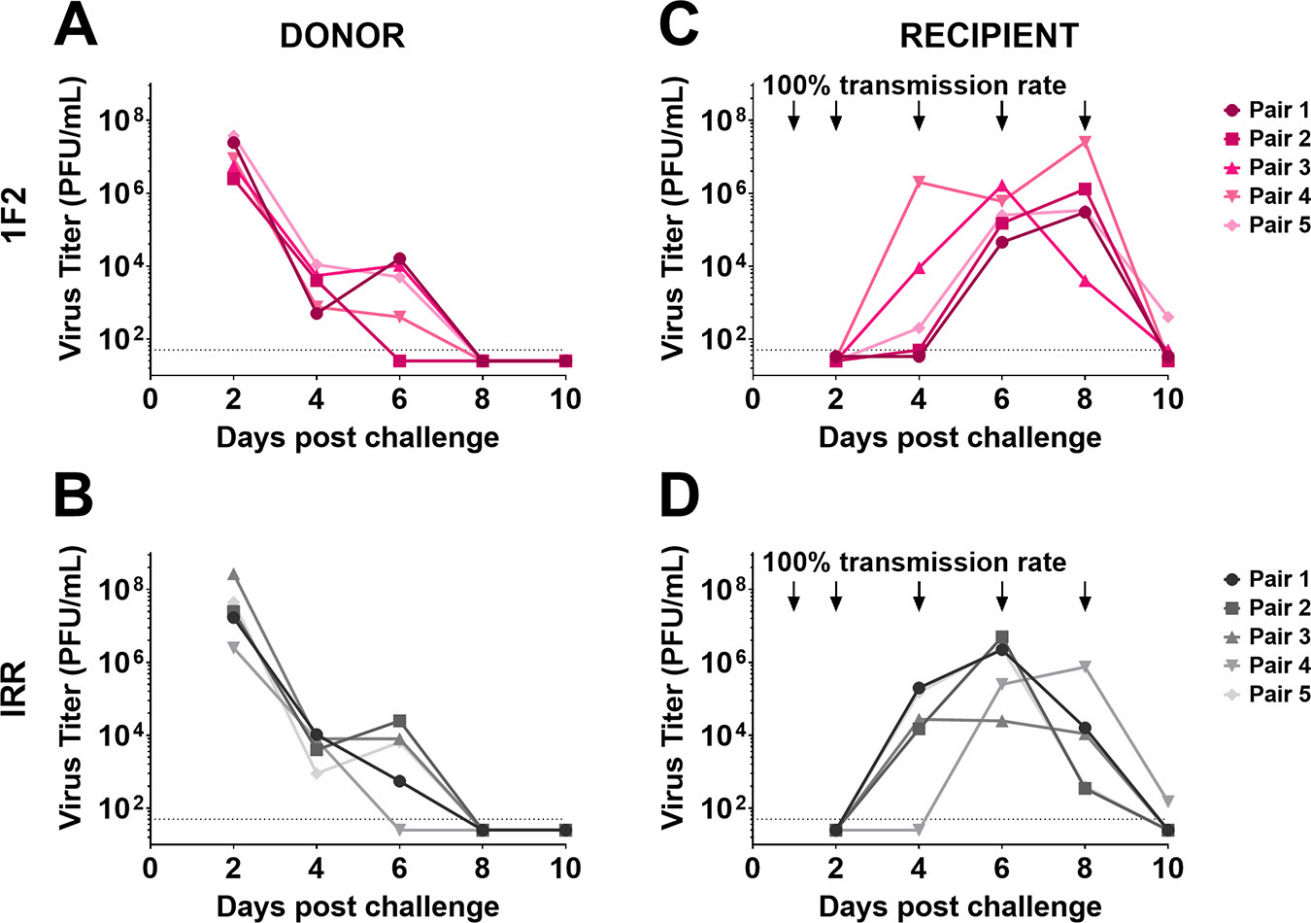
Anti-NA mu-MAb 1F2 administration to recipients does not affect transmission in a direct contact setting. Donor guinea pigs were IN infected with 10^4^ PFU of B/Malaysia/2506/2004 virus and were cocaged with recipients administered 1F2 (A) or an irrelevant (IRR) control mu-MAb (B) at 10 mg/kg on days 1, 2, 4, 6, and 8 postinfection. Nasal washes were collected at 2, 4, 6, 8, and 10 days post initial challenge and viral titers were measured via plaque assay. Transmission to cocaged recipients IN administered 1F2 (C) or IRR mu-MAb control (D) was assessed by collecting nasal washes at 2, 4, 6, 8, and 10 days post initial challenge and measuring viral titers via plaque assay.

Lastly, we utilized the guinea pig model of influenza virus transmission to determine if IN administration of mu-MAb 1F2 at a dose of 10 mg/kg on days 1, 2, 4, 6, and 8 after infection of either the infected donor or the recipient could impact transmission of an IBV between cocaged animals. This model allows viral transmission to occur through multiple modes: direct contact between the infected and exposed animals, indirect contact, and airborne mechanisms. This model is also much more stringent in ensuring transmission than models in which only airborne virus can be transmitted (16). Transmission was monitored by measuring the viral titer in nasal washes collected on days 2, 4, 6, 8, and 10. Donor guinea pigs infected with 10^4^ PFU of B/Malaysia/2506/2004 virus that were administered mu-MAb 1F2 IN showed decreased transmission to cocaged recipients, with a transmission rate of 40% (Fig. 4A and B). Donors administered an irrelevant control antibody transmitted at a rate of 100% to cocaged recipients (Fig. 4C and D). There was no observable impact on transmission when mu-MAb 1F2 was administered to the recipient guinea pigs paired with untreated donors under otherwise identical conditions in this stringent model (Fig. 5).

## DISCUSSION

Here, we further characterized the prophylactic and therapeutic potential of the previously described mu-MAbs that broadly bind and inhibit IBV NAs. To assess the relevance of our BNA mu-MAbs against recent influenza B virus isolates, we successfully conducted challenge experiments in mice using two human clinical IBV isolates, B/New York City/PV00094/2017, a virus of the B/Yamagata/16/88-like lineage collected in 2017, and B/New York City/PV01181/2018, a virus of the B/Victoria/2/87-like lineage collected in 2018. Of the five broad anti-IBV NA mu-MAbs we tested, only 3G1, which showed the narrowest profile as published previously, did not inhibit the NAs of these recent isolates (13). The critical G346R mutation that was previously identified to confer 3G1 escape was not responsible for this loss of binding and no other residues near or proximal to 346 were found to differ between 3G1-sensitive and 3G1-insensitive NAs (13). We assessed the NA amino acid conservation of a random selection of IBVs from available data spanning 81 years and found no changes to amino acids or predicated glycosylation sites that would be responsible for the loss of binding by 3G1 (Fig. S4). The four anti-IBV NA mu-MAbs that demonstrate *in vitro* inhibition also conferred *in vivo* protection when administered through IP injection at 5 mg/kg 2 h before lethal challenge with 5xmLD_50_ of mouse-adapted clinical isolate B/New York City/PV00094/2017 and mouse-passaged isolate B/New York City/PV01181/2018. Prophylactic administration of humanized versions of 1F2 and 4F11 at 5 mg/kg in mice afforded complete protection against morbidity and mortality in a challenge study with 7.5xmLD_50_ of B/Malaysia/2506/2004 virus, a higher challenge dose than used in the original characterization of these mu-MAbs in an effort to measure differential effectiveness (13). The abilities of the tested antibodies, including the original mu-MAbs, to provide protection against weight loss were not sustained with therapeutic administration at 48 h after challenge with 7.5xmLD_50_ of the B/Malaysia/2506/2004 virus. In this setting, protection against mortality ranged from 40% to 100% for the 1F2-based hu-MAbs, compared to 60% protection with mu-1F2, and 60% to 100% for the 4F11-based hu-MAbs, compared to 40% with mu-4F11.

10.1128/msphere.00927-21.5 FIG S4 Amino acid conservation mapped onto a three-dimensional structure of the NA. The top view of the NA of B/Brisbane/60/2008 (PDB ID 4CPL) is depicted as a tetramer with 3 monomers colored in grey. Each amino acid of the remaining subunit is colored ranging red through yellow. Red indicates 100% amino acid conservation at a particular amino acid site, the highest % amino acid conservation we found. Yellow indicates 47% amino acid conservation at a particular amino acid site, the lowest % amino acid conservation we found. Amino acid conservation was determined using all available sequences prior to 2001 and 50 randomly selected IBV NAs collected from each year from 2001 – July 6, 2021. The previously identified 3G1 mutation at amino acid 346 is highlighted in cyan. Download FIG S4, TIF file, 0.3 MB. Copyright © 2022 Tan et al. 2022 Tan et al. https://creativecommons.org/licenses/by/4.0/ This content is distributed under the terms of the Creative Commons Attribution 4.0 International license.

Humanization of mu-MAbs minimizes the risks of immunogenicity and development of antidrug antibodies in humans receiving these biopharmaceuticals. Our studies indicate that many of the hu-MAbs lost binding breadth — they retained binding to older NAs, but lost activity against more recent NAs (e.g., B/New York City/PV01181/2018). Although we could not dissect the reasons for these changes in this study, it is important for hu-MAbs to retain binding; however, to improve on the prophylactic and therapeutic efficacy of broadly protective anti-NA mu-MAbs is essential for continuing to develop and translate these and other promising mu-MAbs into clinically useful therapeutics, which may gain advantageous attributes through protein engineering.

Another important consideration in the development of these MAbs for clinical use is the route of administration. Previous studies testing anti-NA MAbs utilized IP injection, a systemic delivery mechanism (13, 17–19). When testing the more targeted delivery method of IN instillation, we detected no differences compared with IP administration of 10 mg/kg of mu-MAb 1F2 prophylactically at 24 h prior to challenge with 50xmLD_50_ of B/Malaysia/2506/2004 virus or therapeutically at 48 or 96 h after challenge with 5xmLD_50_ of B/Malaysia/2506/2004 virus in terms of protection against morbidity and mortality. There was, however, a nonstatistically significant but perceptible difference between the two routes of administration at 72 h after infection, with 3 of the 5 mice receiving mu-MAb 1F2 through IP injection surviving compared to 0 of 5 mice receiving mu-MAb 1F2 through IN instillation. We also assessed the impact of IP or IN mu-MAb 1F2 administration 48 h after infection on viral titers in lungs harvested on days 4 and 6 postinfection and found no differences between IN and IP administration, although these findings were limited by small group size (*n *=* *3) and testing with mu-MAb 1F2 administration 72 h after infection may aid our understanding of the observed effect in the challenge study.

Delivery of mu-MAb 1F2 through the IN route to an infected animal also reduced IBV transmission between cocaged guinea pigs in a relatively transmission-permissive setup that allowed for viral transmission via direct contact, indirect contact, and/or airborne particles, referred to as the close-contact or direct-contact model, in contrast to models that exclude this mode of transmission. In this study, donor animals received an inoculum of 10^4^ PFU of B/Malaysia/2506/2004 on day 0 and were cohoused with recipient guinea pigs on the following day. The quantification of shed virus from nasal washes indicated that each directly infected donor guinea pig experienced productive infection with titers exceeding 10^6^ PFU/mL on day 2 that subsequently declined until they fell below the limit of detection by day 6 or day 8. This pattern resembles the dynamics observed in some human challenge studies, in which viral shedding peaked on day 2 (19).

Our findings strongly suggest that IN administration of an anti-NA antibody to an infected individual may reduce onward transmission to close contacts even without impacting viral replication or duration of viral shedding, factors that underpin the prevailing understanding of how small molecule NA inhibitors such as oseltamivir are believed to reduce influenza virus transmission (20, 21). Although these consequences of NAI have been identified and linked to decreased transmissibility, other mechanisms by which decreased NA activity reduces influenza virus transmission should be investigated and brought to bear in efforts to limit viral spread and control influenza outbreaks. Indeed, previous work has indicated that NA antibodies are associated with shortened duration of both shedding and symptoms, meaning that, although NA antibodies did not protect individuals from infection, higher levels of NA antibodies correlated with decreased severity of illness in infected individuals (20). Decreased duration of illness and virus shedding may impact influenza virus transmission from individuals with higher NA antibody levels. Although, importantly, our work does indicate that antibody treatment has no obvious effect on virus titers in the nasal washes obtained from infected guinea pigs. This is in line with our previous work where the administration of anti-N1 and anti-N2 MAbs did not significantly affect infection a H1N1 or H3N2 virus, respectively, but the administration of the anti-N1 and anti-N2 MAbs did impact transmission, as we also observed in this work when infected donors were administered 1F2.

The lack of an observable benefit from administration of the anti-NA mu-MAb to the recipient guinea pigs in this study does not rule out the possibility that such prophylaxis may protect an exposed individual from becoming infected by an influenza virus. We chose the close-contact model of transmission, in which the infected donor and the recipient animals share a cage and have direct physical contact with one another, rather than an airborne transmission model in which the animals are positioned in different cages and physically separated with wire mesh, so that air flows from the donor cage to the recipient cage. Previous studies comparing the transmission rate in these two settings predictably discerned higher transmission efficiency in the close-contact setting (21, 22). We did not have the resources to conduct these experiments in both transmission settings, but we would expect that anti-NA antibody administration to the donor would result in greater reduction of airborne influenza virus transmission and that anti-NA antibody administration of the recipient might cause a detectable reduction in the airborne setting, similar to vaccination studies where recipient guinea pigs were vaccinated with recombinant NA and protected from transmission in an airborne setting (23). Altering the timing and dosing of anti-NA antibody administration may also result in differential reductions of influenza virus transmission in the guinea pig model.

Because IN delivery of an anti-NA mu-MAb can prophylactically and therapeutically protect against morbidity and mortality in the context of influenza virus challenge and reduce close-contact transmission from an infected individual to a nontreated individual, further work should be carried out to explore IN routes of administration. We summarize the potential benefits for the intranasal administration of MAbs in Table 2. Antibody-based antivirals that currently have FDA approval or emergency use authorization for treating infectious diseases are delivered intravenously, requiring administration by medically trained personnel in inpatient or outpatient settings. In contrast, IN delivery systems, such as nasal sprays, inhalers, and nebulizers, are utilized to dispense some therapeutics directly to the airway. In addition to providing localized delivery, IN administration is noninvasive, occurs rapidly and does not require medical training, safe injection practices, or additional equipment. These differences may have substantial impacts, especially during pandemics when medical personnel and supplies can become rate-limiting, and when clustering in clinics and hospitals should be minimized. Individuals with influenza disease requiring treatment and those in need of prophylaxis, whether throughout the influenza season or after a potential exposure, could self-administer an IN anti-NA antibody or antibody cocktail according to a prescribed schedule for protection against influenza virus, and to reduce viral transmission to their close contacts.

**TABLE 2:** Comparison of intravenous versus intranasal administration of MAbs

Route of administration	Intravenous	Intranasal sprays and inhalers
Distribution	Systemic	Airway
Approximate length of time for drug delivery	1 hr	Seconds
Invasiveness	Invasive – needle and catheter inserted through skin and into vein	Noninvasive
Potential adverse events	Bruising, infection, vein inflammation, diarrhea, nausea and infusion-related reactions, including anaphylaxis	Epistaxis, headache, local irritation, loss of taste or smell and anaphylaxis
Difficulty of use	High – requires medical training and sterile equipment	Low – can be used without medical training and additional equipment

This study provides supporting evidence for the development of broadly reactive anti-NA MAbs as potential prophylactic and therapeutic biologicals, and the consideration of NA epitopes as vaccine targets that induce immune responses against relatively conserved epitopes. Current anti-NA antibodies and vaccines that induce them might not only prevent infection by a broad spectrum of influenza viruses, but potentially could reduce their transmission drastically.

## MATERIALS AND METHODS

### Ethics statement.

Nasopharyngeal swab specimens were collected as part of the routine virus surveillance conducted by the Mount Sinai Pathogen Surveillance program (IRB approved, HS number 13-00981). All animal procedures were performed in accordance with the Icahn School of Medicine at Mount Sinai Institutional Animal Care and Use Committee (IACUC).

### Cells.

Madin-Darby canine kidney (MDCK) cells were grown and maintained at 37°C in 5% CO_2_ in complete Dulbecco’s modified Eagle’s medium (cDMEM), comprised of DMEM (Gibco) supplemented with penicillin-streptomycin (100 U/mL penicillin, 100 μg/mL streptomycin), fetal bovine serum (FBS) (10%, Corning) and 4-(2-hydroxyethyl)-1-piperazineethanesulfonic acid (HEPES) buffer (0.01 M, Gibco).

### Viruses.

Clinical isolates A/New York City/PV00094/2017 (B/Yamagata/16/88-like) and A/New York City/PV01181/2018 (B/Victoria/2/87-like lineage) were obtained through the Mount Sinai Pathogen Surveillance Program at the Icahn School of Medicine at Mount Sinai. The original nasopharyngeal swabs were used to infect MDCK cells in 1× minimum essential medium (MEM) comprised of 10% 10× MEM (Gibco), 2 mM l-glutamine, 0.1% sodium bicarbonate (wt/vol, Gibco), 0.01 M HEPES buffer (Gibco), penicillin-streptomycin (100 U/mL penicillin, 100 μg/mL streptomycin), 0.2% bovine serum albumin (BSA), 1 μg/mL tolylsulfonyl phenylalanyl chloromethyl ketone (TPCK)-treated trypsin, and 0.1% (wt/vol) diethylaminoethanol (DEAE)-dextran (Sigma). Infected cells were incubated at 33°C with 5% CO_2_ for 72 h and checked daily for cytopathic effect (CPE). After 72 h and detection of CPE, supernatants were collected, clarified by centrifugation, and stored at −80°C.

Mouse adaptation of B/New York City/PV00094/2017 and B/New York City/PV01181/2018 was carried out by serially infecting female DBA/2J mice purchased from the Jackson Laboratory with cell-grown virus isolates. Lungs were harvested 4 days postinfection, homogenized in 600 μL sterile phosphate-buffered saline (PBS), aliquoted, and frozen at −80°C for storage. Plaque assays were completed using lung homogenates as described below to determine successful recovery of virus before carrying out the subsequent passage. Successive passaging through mice was performed by inoculating an anesthetized mouse with 50 μL of lung homogenate from the previous passage diluted 10-fold in sterile PBS. The B/New York City/PV01181/2018 virus was recovered successfully after each of the 5 passages through mice, however, the B/New York City/PV00094/2017 virus could not be recovered after the fifth passage. The lung homogenate from the fourth passage had low viral titer and was amplified in embryonated chicken eggs to provide a higher inoculum of 10^6^ PFU for the fifth mouse passage. The lung homogenates from the fifth mouse passages were used to inoculate eggs.

Stocks of B/New York City/PV00094/2017 and B/New York City/PV01181/2018 generated after the second passage through MDCK cells and after five passages through mice were considered the “unadapted” and “adapted” stocks, respectively. These stocks and a stock of a mouse-adapted B/Malaysia/2506/2004 virus were grown in 10-day-old embryonated chicken eggs incubated at 33°C for 72 h. To generate a stock of allantoic fluid to use for mock infection of mice as a control for comparison to infection with B/Malaysia/2506/2004 virus, 10-day-old embryonated chicken eggs were injected with sterile PBS or B/Malaysia/2506/2004 virus in parallel and later steps, including the dilution of the stocks for inoculating mice, which was also performed in parallel. Allantoic fluid from infected eggs was screened individually for relative viral titer in standard hemagglutination assays using chicken erythrocytes. Samples with high hemagglutination activity were pooled to generate stocks. Viral titers of the stocks were quantified using plaque assays with MDCK cells as described below.

B/Malaysia/2506/2004, B/Wisconsin/1/2010, and B/Yamagata/16/88 viruses were grown in 10-day-old specific-pathogen-free embryonated chicken eggs (Charles River Laboratories) for 72 h at 33°C. After 72 h, allantoic fluid was collected, clarified by centrifugation, and stored at −80°C.

### Antibodies.

mu-MAbs were produced from monoclonal hybridomas generated previously (9). Hybridomas were recovered from cryostocks in ClonaCell-HY Medium E (Stemcell Technologies) before they were gradually switched into serum-free hybridoma medium (Hybridoma-SFM, Life Technologies) for expansion and antibody production. Ten to thirteen days after the final expansion step, when cells no longer appeared viable, the cultures were harvested, centrifuged at 4,000 × *g* at 4°C for 30 min, and filtered. The filtrate was then applied to gravity flow columns packed with protein G-Sepharose 4 Fast Flow beads (GE Healthcare). After 3× washes with sterile PBS, MAbs were eluted using 45 mL 0.1 M glycine buffer (pH 2.7) directly into 5 mL 2 M Tris-HCl buffer (pH 10) for neutralization. The MAbs were then concentrated, and buffer exchanged in PBS (pH 7.4) using Amicon Ultra centrifugal filter units with a 30-kDa cutoff (Millipore). The final mu-MAb concentration was determined using a NanoDrop device (Thermo Scientific) based on the absorbance at 280 nm.

MAb humanization was carried out by a contract research organization, Fusion Antibodies, using a combination of industry-standard CDR grafting and their proprietary technology. Briefly, using the sequences of mu-MAbs 1F2 and 4F11, 1 chimeric and 25 humanized variants of each were synthesized upon identification of a number of human framework acceptor sequences. The acceptor sequences all came from mature human IgGs from a human source. The humanized sequences were screened for T cell epitopes, Fv glycosylation sites, and deamidation sites, which could all negatively impact the properties of the final product. The antibody gene sequences were subcloned into an appropriate mammalian transient expression vector. Transient transfections of each of the humanized antibody variant vector DNA were carried out in Chinese hamster ovary (CHO) cells. Following batch culture, expressed hu-MAb variants were purified from the cell culture supernatant. Finally, the kinetic interactions between the humanized antibody variants and their respective antigens were characterized before they were shipped to Mount Sinai.

### Mouse challenge experiments.

Five- to 6-week-old female BALB/c mice were ordered from the Jackson Laboratory for all mouse experiments. Mice were randomly assigned to groups of five per group for challenge studies. All mice were anesthetized prior to infection or mock infection with 50 μL allantoic fluid diluted in sterile PBS; mock-infected allantoic fluid was diluted in an identical manner as the stock used to infect mice for the same experiment. All mice were also anesthetized prior to MAb administration when comparing IP and IN delivery methods. The body weight of animals was monitored daily during the virus challenge experiments and mice were scored dead on the day when they crossed the predefined humane weight loss endpoint (25% weight loss). For studying viral titers in lungs, mice were randomly assigned to groups of three for the IBV infection condition and groups of two for the mock infection condition. Lungs were harvested, homogenized in 600 μL of sterile PBS with 3 mm zirconium beads, centrifuged, aliquoted, and stored at −80°C.

### Guinea pig transmission experiments.

Five- to 6-week-old female guinea pigs were purchased from Charles River Laboratories. Blood was collected from animals from the lateral saphenous vein before experiments were conducted. Donor guinea pigs were randomly selected and anesthetized with ketamine (30 mg/kg) and xylazine (5 mg/kg) before being infected with 10^4^ PFU of B/Malaysia/2506/2004 delivered IN in 300 μL sterile PBS. The following day, recipient guinea pigs were introduced into the same cages as the infected donor guinea pigs. All guinea pigs were anesthetized with ketamine (30 mg/kg) and xylazine (5 mg/kg) and nasal washes were performed with 1 mL sterile PBS on each animal on days 2, 4, 6, 8, and 10 after donor animals were infected. Plaque assays were performed using nasal wash samples as described below. Guinea pigs were anesthetized with ketamine (44 mg/kg) and xylazine (5 mg/kg) and terminally bled 14 to18 days after donors were infected.

mu-MAb 1F2 was delivered IN to anesthetized animals at 10 mg/kg in 350 μL sterile PBS. To test antibody administration in donor guinea pigs, the infected animals were given the antibody on days 1, 2, 4, 6, and 8 after infection and paired with untreated recipients. To test antibody administration in recipient guinea pigs, the naive recipients were given the antibody on days 1, 2, 4, 6, and 8 after the donor animals were infected and paired with untreated donors.

### Plaque assays.

Virus titers were measured by performing plaque assays on MDCK cells seeded at 3 × 10^5^ cells/mL in 12-well plates. The plates were incubated overnight at 37°C in 5% CO_2_. Virus stocks, lung homogenates, or guinea pig nasal wash samples diluted serially in 1× MEM by a factor of 10 were added to MDCK monolayers and incubated at 33°C for 1 h with shaking every 15 min. The inoculum in each well was then replaced with an overlay containing 0.64% agar (Oxoid), 1× MEM, 1 μg/mL tosylsulfonyl phenylalanyl chloromethyl ketone (TPCK)-treated trypsin and 0.1% (wt/vol) diethylaminoethanol (DEAE)-dextran (Sigma). The cells were then incubated for 72 h at 33°C with 5% CO_2_. Plaques were visualized by immunostaining with a cocktail of the five anti-IBV NA MAbs. The limit of detection for these plaque assays was 50 PFU/mL.

### Enzyme-linked lectin assays (ELLAs).

To determine NA activity, samples were tested on flat-bottom Immunolon 4BX 96-well plates coated overnight at 4°C with 100 μL of fetuin (Sigma) at 25 μg/mL diluted in PBS and subsequently washed 3× with PBS containing 0.1% Tween 20 (Fisher) (PBS-T). On a separate plate, viruses were serially diluted 2-fold in sample diluent (PBS [Gibco] with 0.9 mM CaCl_2_ and 0.5 mM MgCl_2_ supplemented with 1% bovine serum albumin [MP Biomedicals], and 0.5% Tween 20 [Fisher Scientific]). Subsequently, 100 μL of diluted virus samples were added to the washed fetuin-coated plates. The fetuin-coated plates were then incubated overnight (for 16–18 h) at 37°C. Plates were washed 3× with PBS-T before 100 μL/well horseradish peroxidase (HRP)-conjugated peanut agglutinin (PNA) in PBS was added to the plates. The plates were incubated for 2 h at room temperature before being washed 4× with PBS-T with shaking. To develop the plates, 100 μL of *O*-phenylenediamine dihydrochloride (OPD) substrate (SigmaFast OPD; Sigma-Aldrich) was added to each well. After a 10-min incubation, the reaction was stopped by adding 50 μL of 3 M HCl to each well. The optical density at 490 nm (OD_490_) was measured on a Synergy 4 plate reader (BioTek). The half maximal effective concentration (EC_50_) was determined using GraphPad Prism 8.

To measure NAI activity, antibodies were serially diluted in sample diluent with a starting concentration of 30 μg/mL and incubated for 18 h at 37°C with an equal volume (50 μL) of the respective virus dilution in fetuin-coated plates. The remainder of the assay was performed as described above. One column on the plate contained sample diluent without antibody and served as a positive (virus-only) control. Another column contained sample diluent only (no virus) and served as a negative (background) control. Data were analyzed in GraphPad Prism 8.

### Mapping of amino acid identity conservation in IBV NAs.

All available full IBV NA sequences were downloaded from the Global Initiative for sharing all influenza data (GISAID) on July 6, 2021. All sequences collected prior to 2001 and 50 randomly selected IBV NA sequences collected from each year during 2001 to 2021 were mapped onto the NA of B/Brisbane/6/2008 (PDB ID 4CPL) in ChimeraX (Table S1). Amino acids for one NA subunit were colored ranging red through yellow. Red indicates 100% amino acid conservation at a particular amino acid site, the highest % amino acid conservation we found. Yellow indicates 47% amino acid conservation at a particular amino acid site, the lowest % amino acid conservation we found.

10.1128/msphere.00927-21.1 TABLE S1 IBV NA sequences used for conservation mapping by collection year. IBV NA sequences downloaded from the Global Initiative for sharing all influenza data on July 6, 2021 and mapped onto the NA of B/Brisbane/06/08 (PDB ID 4CPL) in ChimeraX. Download Table S1, DOCX file, 0.10 MB. Copyright © 2022 Tan et al. 2022 Tan et al. https://creativecommons.org/licenses/by/4.0/ This content is distributed under the terms of the Creative Commons Attribution 4.0 International license.
