# Supplementary figures and images for: Murine Broadly Reactive Antineuraminidase Monoclonal Antibodies Protect Mice from Recent Influenza B Virus Isolates and Partially Inhibit Virus Transmission in the Guinea Pig Model
Source: mSphere. 2022 Sep 7;7(5):e00927-21. doi: 10.1128/msphere.00927-21 (PMC9599422; doi:10.1128/msphere.00927-21)

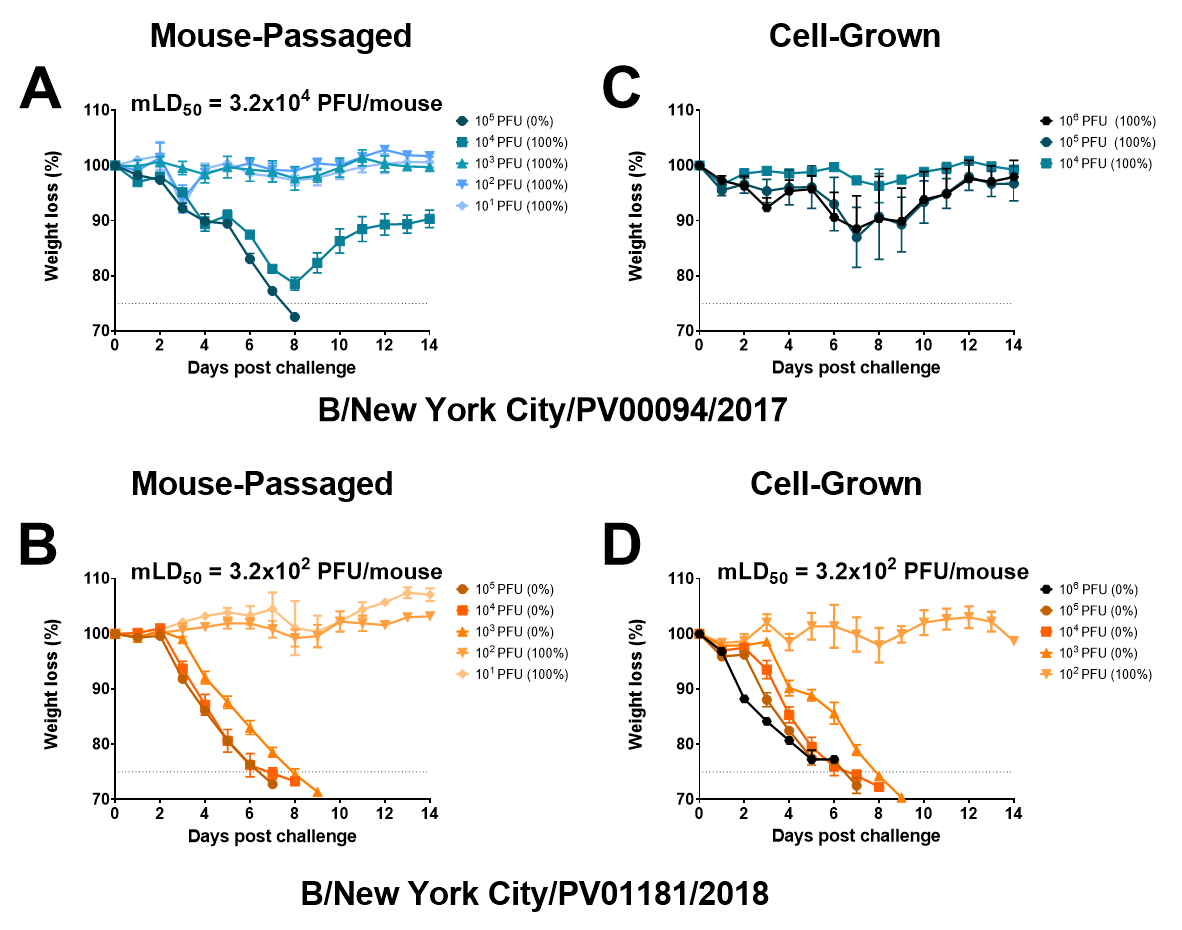

Supplement: FIG S1 [file msphere.00927-21-s0002.tif]

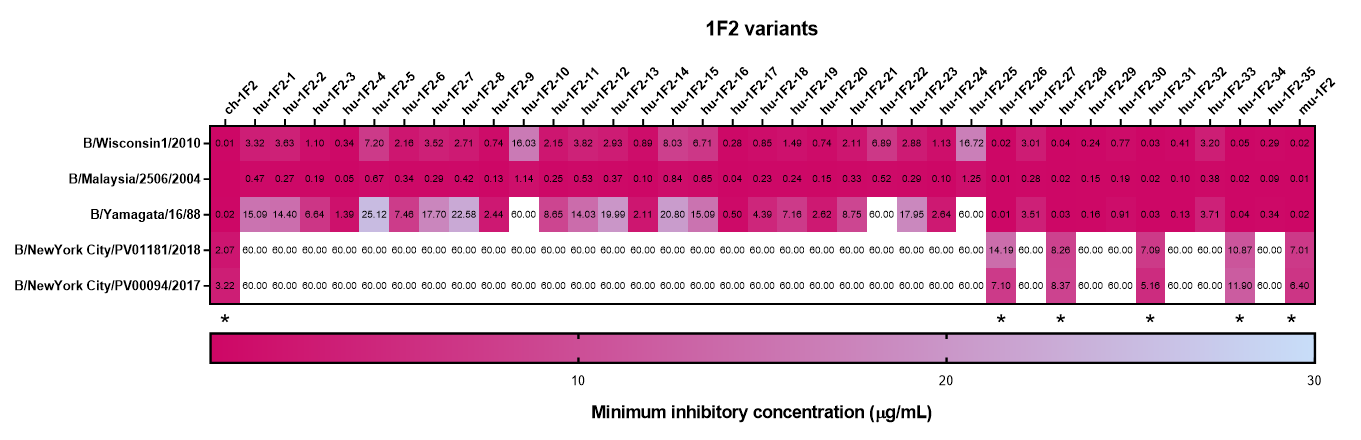

Supplement: FIG S2 [file msphere.00927-21-s0003.tif]

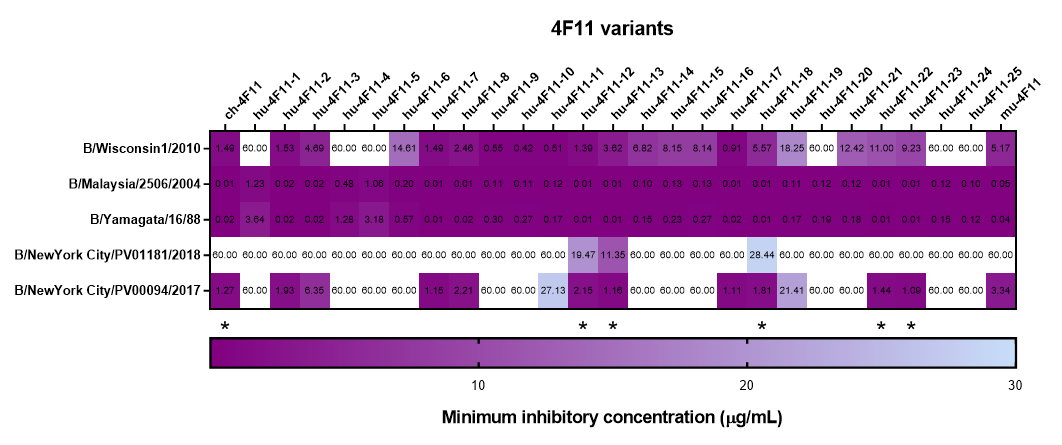

Supplement: FIG S3 [file msphere.00927-21-s0004.tif]

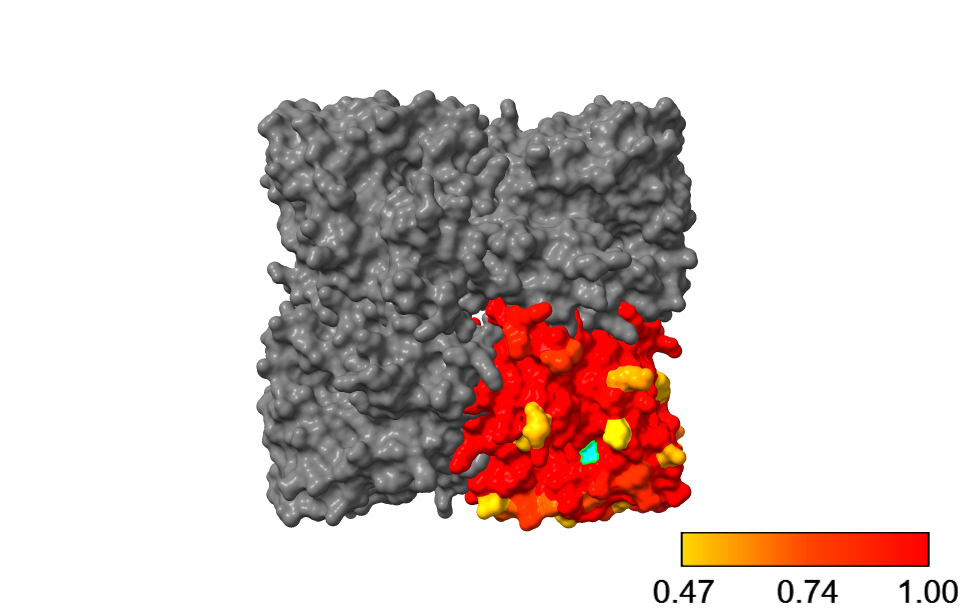

Supplement: FIG S4 [file msphere.00927-21-s0005.tif]
