# Supplementary material for: Murine Broadly Reactive Antineuraminidase Monoclonal Antibodies Protect Mice from Recent Influenza B Virus Isolates and Partially Inhibit Virus Transmission in the Guinea Pig Model
Source: mSphere. 2022 Sep 7;7(5):e00927-21. doi: 10.1128/msphere.00927-21 (PMC9599422; doi:10.1128/msphere.00927-21)
Supplement: TABLE S1 [file msphere.00927-21-s0001.docx]

| **Virus** | **Year** | **NA amino acid sequence** |
| --- | --- | --- |
| B/Lee/1940 | 1940 | LPSTVQTLTLLLTSGGVLLSLYVSASLSYLLYSDVLLKFSSTKTTAPTMSLECTNASNAQTVNHSATKEMTFPPPEPEWTYPRLSCQGSTFQKALLISPHRFGEIKGNSAPLIIREPFVACGPKECRHFALTHYAAQPGGYYNGTRKDRNKLRHLVSVKLGKIPTVENSIFHMAAWSGSACHDGREWTYIGVDGPDNDALVKIKYGEAYTDTYHSYAHNILRTQESACNCIGGDCYLMITDGSASGISKCRFLKIREGRIIKEILPTGRVEHTEECTCGFASNKTIECACRDNSYTAKRPFVKLNVETDTAEIRLMCTKTYLDTPRPDDGSIAGPCESNGDKWLGGIKGGFVHQRMASKIGRWYSRTMSKTNRMGMELYVKYDGDPWTDSDALTLSGVMVSIEEPGWYSFGFEIKDKKCDVPCIGIEMVHDGGKDTWHSAATAIYCLMGSGQLLWDTATGVDMAL |
| B/Russia/69 | 1969 | LPSTIQTLTLFLTSGGVLLSLYVSASLSYLLYSDILLKFSPTKITAPTMSLDCANVSNVQAVNRSATKEMTFLLPEPEWTYPRLSCQGSTFQKALLISPHRFGETRGNSAPLIIREPFVACGPKECRHFALTHYAAQPGGYYNGTRKDRNKLRHLISVKLGKIPTVENSIFHMAAWSGSACHDGREWTYIGVDGPDSNALIKIKYGEAYTDTYHSYANNILRTQESACNCIGGDCYLMITDGSASGISKCRFLKIREGRIIKEIFPTGRVEHTEECTCGFASNKTIECACRDNSYTAKRPFVKLNVETDTAEIRLMCTETYLDTPRPDDGSITGPCESNGDKGLGGIKGGFVHQRMASKIGRWYSRTMSKTERMGMELYVKYDGDPWTDSDALAPSGVMVSMKEPGWYSFGFEIKDKKCDVPCIGIEMVHDGGKETWHSAATAIYCLMGSGQLLWDTVTGVDMAL |
| B/Hong Kong/8/1973 | 1973 | LPSTIQTLTLFLTSGGVLLSLYVSASLSYLLYSDILLKFSSTKRTAPTMSLDCANVSNVQAVNRSATKEMTFLLPEPEWTYPRLSCQGSTFQKALLISPHRFGETRGNSAPLIIREPFVACGPKECRHFALTHYAAQPGGYYNGTRKDRNKLRHLISVKLGKIPTVENSIFHMAAWSGSACHDGREWTYVGVDGPDSNALIKIKYGEAYTDTYHSYANNILRTQESACNCIGGDCYLMITDGSASGISKCRFLKIREGRIIKEIFPTGRVEHTEECTCGFASNKTIECACRDNSYTAKRPFVKLNVETDTAEIRLMCTETYLDTPRPDDGSITGPCESNGDKGLGGIKGGFVHQRMASKIGRWYSRTMSKTERMGMELYVKYDGDPWTDSDALAPSGVMVSMKEPGWYSFGFEIKDKKCDVPCIGIEMVHDGGKETWHSAATAIYCLMGSGQLLWDTVTGVDMAL |
| B/Yamagata/1/73 | 1973 | LPSTIQTLTLFLTSGGVLLSLYVSASLSYLLYSDILLKFSSTKITAPTMSLDCANVSNVQAVNRSATKEMTSLLPEPEWTYPRLSCQGSTFQKALLISPHRFGETRGNSAPLIIREPFVACGPKECRHFALTHYAAQPGGYYNGTRRDRNKLRHLISVKLGKIPTVENSIFHMAAWSGSACHDGREWTYVGVDGPDSNALIKIKYGEAYTDTYHSYANNILRTQESACNCIGGDCYLMITDGSASGISKCRFLKIREGRIIKEIFPTGRVEHTEECTCGFASNKTIECACRDNSYTAKRPFVKLNVETDTAEIRLMCTETYLDTPRPDDGSITGPCESNGDKGLGGIKGGFVHQRMASKIGRWYSRTMSKTERMGMELYVKYDGDPWTDSDALAPSGVMVSMKEPGWYSFGFEIKDKKCDVPCIGIEMVHDGGKETWHSAATAIYCLMGSGQLLWDTVTGVDMAL |
| B/Singapore/222/79 | 1979 | LPSTIQTLTLFLTSGGVLLSLYVSASLSYLLYSDILLKFSPTKRTAPTMSLDCANVSNVQAVNRSATKEMTFLLPEPEWTYPRLSCQGSTFQKALLISPHRFGEARGNSAPLIIREPFIACGPKECKHFALTHYAAQPGGYYNGTRKDRNKLRHLISVKLGKIPTVENSIFHMAAWSGSACHDGREWTYIGVDGPDSNALIKIKYGEAYTDTYHSYANNILRTQESACNCIGGDCYLMITDGSASGISKCRFLKIREGRIIKEIFPTGRVEHTEECTCGFASNKTIECACRDNSYTAKRPFVKLNVETDTAEIRLMCTETYLDTPRPDDGSITGPCESNGDKGLGGIKGGFVHQRMASKIGRWYSRTMSKTERMGMELYVKYDGDPWTDSEALAPSGVMVSMKEPGWYSFGFEIKDKKCDVPCIGIEMVHDGGKETWHSAATAIYCLMGSGQLLWDTVTGVDMAL |
| B/Oregon/5/80 | 1980 | LPSTIQTLTLFLTSGGVLLSLYVSASLSYLLYSDILLKFSTTKITAPTMSLDCANISNVQAVNRSATKEMTFLLPEPEWTYPRLSCQGSTFQKALLISPHRFGEARGNSAPLIIREPFIACGPKECKHFALTHYAAQPGGYYNGTRKDRNKLRHLISVKLGKIPTVENSIFHMAAWSGSACHDGREWTYIGVDGPDSNALIKIKYGEAYTDTYHSYANNILRTQESACNCIGGDCYLMITDGSASGISKCRFLKIREGRIIKEIFPTGRVEHTEECTCGFASNKTIECACRDNSYTAKRPFVKLNVETDTAEIRLMCTETYLDTPRPDDGSITGPCESNGDKGLGGIKGGFVHQRMASKIGRWYSRTMSKTERMGMELYVKYDGDPWTDSDALAPSGVMVSMKEPGWYSFGFEIKDKKCDVPCIGIEMVHDGGKETWHSAATAIYCLMGSGQLLWDTVTGVDMAL |
| B/Singapore/04/1991 | 1981 | LPSTIQTLTLFLTSGGVLLSLYVSVSLSYLLYSDILLRFSPTEITAPTMPLDCANASNVQAVNRSATKGMTLRLPEPEWTYPRLSCPGSTFQKALLISPHRFGETRGNSAPLIIREPFIACGPKECKHFALTHYAAQPGGYYNGTREDRNKLRHLISVKLGKIPTVENSIFHMAAWSGSACHDGREWTYIGVDGPDSNALIKIKYGEAYTDTYHSYANNILRTQESACNCIGGDCYLMITDGSASEISKCRFLKIREGRIIKEIFPTGRVEHTEECTCGFASNKTIECACRDNSYTAKRPFVKLNVETDTAEIRLMCTETYLDTPRPDDGSITGPCESNGDKGRGGIKGGFVHQRMASKIGRWYSRTMSKTERMGMELYVKYDGDPWTDSEALAPSGVMVSMEEPGWYSFGFEIKDKKCDVPCIGIEMVHDGGKKTWHSAATAIYCLMGSGQLLWDTVTGVDMAL |
| B/USSR/100/83 | 1983 | LPSTIQTLTLFLTSGGVLLSLYVSASLSYLLYSDILLKFSPTKRTAQTMSLDCANASNVQAVNHSATKEMTFLLPEPEWTYPRLSCQGSTFQKALLISPHRFGEARGNSAPLIIREPFIACGPKECKHFALTHYAAQPGGYYNGTREDRNKLRHLISVKLGKIPTVENSIFHMAAWSGSACHDGREWTYIGVDGPDSNALIKIKYGEAYTDTYHSYANNILRTQESACNCIGGDCYLMITDGSASGISKCRFLKIREGRIIKEIFPTGRVEHTEECTCGFASNKTIECACRDNSYTAKRPFVKLNVETDTAEIRLMCTETYLDTPRPDDGSITGPCESNGDKGRGGIKGGFVHQRMASKIGRWYSRTMSKTERMGMELYVKYDGDPWTDSDALAPSGVMVSMKEPGWYSFGFEIKDKKCDVPCIGIEMVHDGGKKTWHSAATAIYCLMGSGQLLWDTVTGVDMAL |
| B/Norway/1/84 | 1984 | LPSTIQTLTLFLTSGGVLLSLYVSASLSYLLYSDILLKFSPTKITAQTMSLDCANASNVQVVNHSATKEMTFLLPEPEWTYPRLSCQGSTFQKALLISPHRFGEARGNSAPLIIREPFVACGPKECKHFALTHYAAQPGGYYNGTREDRNKLRHLISVKLGKIPTVENSIFHMAAWSGSACHDGREWTYIGVDGPDSNALIKIKYGEAYTDTYHSYANNILRTQESACNCIGGDCYLMITDGSASGISKCRFLKIREGRIIKEILPTGRVEHTEECTCGFASNKTIECACRDNSYTAKRPFVKLNVETDTAEIRLMCTETYLDTPRPDDGSITGPCESNGDKGRGGIKGGFVHQRMASKIGRWYSRTMSKTERMGMELYVKYDGDPWTDSDALAPSGVMVSMKEPGWYSFGFEIKDKKCDVPCIGIEMVHDGGKKTWHSAATAIYCLMGSGQLLWDTVTGVDMAL |
| B/Ibaraki/2/85 | 1985 | LPSTIQTLTLFLTSGGVLLSLYVSASLSYLLYSDILLKFSP-KITAPTMTLDCANASNVQAVNRSATKEMTFLLPEPEWTYPRLSCQGSTFQKALLISPHRFGEARGNSAPLIIREPFIACGPKECKHFALTHYAAQPGGYYNGTREDRNKLRHLISVKLGKIPTVENSIFHMAAWSGSACHDGREWTYIGVDGPDSNALIKIKYGEAYTDTYHSYANNILRTQESACNCIGGDCYLMITDGSASGISKCRFLKIREGRIIKEIFPTGRVEHTEECTCGFASNKTIECACRDNNYTAKRPFVKLNVETDTAEIRLMCTETYLDTPRPDDGSITGPCESNGDKGRGGIKGGFVHQRMASKIGRWYSRTMSKTERMGMELYVKYDGDPWTDSDALAPSGVMVSMKEPGWYSFGFEIKDKKCDVPCIGIEMVHDGGKKTWHSAATAIYCLMGSGQLLWDTVTGVDMAL |
| B/Victoria/3/85 | 1985 | LPSTIQTLTLFLTSGGVLLSLYVSASLSYLLYSDILLKFSP-KITAPTMTLDCTNASNVQAVNRSATKEMTFLLPEPEWTYPRLSCQGSTFQKALLISPHRFGEARGNSAPLIIREPFIACGPKECKHFALTHYAAQPGGYYNGTREDRNKLRHLISVKLGKIPTVENSIFHMAAWSGSACHDGREWTYIGVDGPDSNALIKIKYGEAYTDTYHSYANNILRTQESACNCIGGDCYLMITDGSASGISKCRFLKIREGRIIKEIFPTGRVEHTEECTCGFASNKTIECACRDNNYTAKRPFVKLNVETDTAEIRLMCTETYLDTPRPDDGSITGPCESNGDKGRGGIKGGFVHQRMASKIGRWYSRTMSKTERMGMELYVKYDGDPWTDSDALDPSGVMVSIKEPGWYSFGFEIKDKKCDVPCIGIEMVHDGGKKTWHSAATAIYCLMGSGQLLWDTVTGVDMAL |
| B/Ann Arbor/1/1986 | 1986 | LPSTIQTLTLFLTSGGVLLSLYVSASLSYLLYSDILLKFSP-KITAPTMTLDCANASNVQAVNRSATKEMTFLLPEPEWTYPRLSCQGSTFQKALLISPHRFGEARGNSAPLIIREPFIACGPKECKHFALTHYAAQPGGYYNGTREDRNKLRHLISVKLGKIPTVENSIFHMAAWSGSACHDGREWTYIGVDGPDSNALIKIKYGEAYTDTYHSYANNILRTQESACNCIGGDCYLMITDGSASGISKCRFLKIREGRIIKEIFPTGRVEHTEECTCGFASNKTIECACRDNNYTAKRPFVKLNVETDTAEIRLMCTETYLDTPRPDDGSITGPCESNGDKGRGGIKGGFVHQRMASKIGRWYSRTMSKTERMGMELYVKYDGDPWTDSDALDPSGVMVSMKEPGWYSFGFEIKDKKCDVPCIGIEMVHDGGKKTWHSAATAIYCLMGSGQLLWDTVTGVDMAL |
| B/Leningrad/179/86 | 1986 | LPSTIQTLTLFLTSGGVLLSLYVSASLSYLLYSDILLKFSP-KITAPTMTLDCANASNVQAVNRSATKEMTFLLPEPEWTYPRLSCQGSTFQKALLISPHRFGEARGNSAPLIIREPFIACGPKECKHFALTHYAAQPGGYYNGTREDRNKLRHLISVKLGKIPTVENSIFHMAAWSGSACHDGREWTYIGVDGPDSNALIKIKYGEAYTDTYHSYANNILRTQESACNCIGGDCYLMITDGSASGISECRFLKIREGRIIKEIFPTGRVEHTEECTCGFASNKTIECACRDNNYTAKRPFVKLNVETDTAEIRLMCTETYLDTPRPDDGSITGPCESNGDKGRGGIKGGFVHQRMASKIGRWYSRTMSKTERMGMELYVKYDGDPWTDSDALAPSGVMVSIKEPGWYSFGFEIKDKKCDVPCIGIEMVHDGGKKTWHSAATAIYCLMGSGQLLWDTVTGVDMAL |
| B/Memphis/6/86 | 1986 | LPSTIQTLTLFLTSGGVLLSLYVSASLSYLLYSDILLKFSP-KITAPTMTLDCANASNVQAVNRSATKEMTFLLPEPEWTYPRLSCQGSTFQKALLISPHRFGEARGNSAPLIIREPFIACGPKECKHFALTHYAAQPGGYYNGTREDRNKLRHLISVKLGKIPTVENSIFHMAAWSGSACHDGREWTYIGVDGPDSNALIKIKYGEAYTDTYHSYANNILRTQESACNCIGGDCYLMITDGSASGISECRFLKIREGRIIKEIFPTGRVEHTEECTCGFASNKTIECACRDNNYTAKRPFVKLNVETDTAEIRLMCTETYLDTPRPDDGSITGPCESNGDKGRGGIKGGFVHQRMASKIGRWYSRTMSKTERMGMELYVKYDGDPWTDSDALAPSGVMVSIKEPGWYSFGFEIKDKKCDVPCIGIEMVHDGGKKTWHSAATAIYCLMGSGQLLWDTVTGVDMAL |
| B/Beijing/1/87 | 1987 | LPSTIQTLTLFLTSGGVLLSLYVSASLSYLLYSDILLKFSS-KITAPTMTLDCANASNVQAVNRSATKEMTFLLPEPEWTYPRLSCQGSTFQKALLISPHRFGEARGNSAPLIIRGPFIACGPKECKHFALTHYAAQPGGYYNGTREDRNKLRHLISVKLGKIPTVENSIFHMAAWSGSACHDGREWTYIGVDGPDSNALIKIKYGEAYTDTYHSYANNILRTQESACNCIGGDCYLMITDGSASGISKCRFLKIREGRIIKEIFPTGRVEHTEECTCGFASNKTIECACRDNSYTAKRPFVKLNVETDTAEIRLMCTETYLDTPRPDDGSITGPCESNGDKGRGGIKGGFVHQRMASKIGRWYSRTMSKTERMGMELYVKYDGDPWTDSEALAHSGVMVSMKEPGWYSFGFEIKDKKCDVPCIGIEMVHDGGKKTWHSAATAIYCLMGSGQLLWDTVTGVDMAL |
| B/Victoria/02/1987 | 1987 | LPSTIQTLTLFLTSGGVLLSLYVSALLSYLLYSDILLKFSPTKIIAPTMSLDCANASNVQAANHSATKEMTFLLPEPEWTYPRLSCQGSTFQKALLISPHRFGEAKGNSAPLIIREPFIACGPKECKHFALTHYAAQPGGYYNGTREDRNKLRHLISVNLGKIPTVENSIFHMAAWSGSACHDGREWTYIGVDGPDSNALIKIKYGEAYTDTYHSYANNILRTQESACNCIGGDCYLMITDGSASGISKCRFLKIREGRIIKEIFPTGRVEHTEECTCGFASNKTIECACRDNSYTAKRPFVKLNVETDTAEIRLMCTETYLDTPRPDDGSITGPCESNGEKGRGGIKGGFVHQRMASKIGRWYSRTMSKTERMGMELYVKYDGDPWTDSDALAPSGVMVSMKEPGWYSFGFEIKDKKCDVPCIGIEMVHDGGKKTWHSAATAIYCLMGSGQLLWDTVTGVDMAL |
| B/Victoria/2/87 | 1987 | LPSTIQTLTLFLTSGGVLLSLYVSALLSYLLYSDILLKFSPTKIIAPTMSLDCANASNVQAANHSATKEMTFLLPEPEWTYPRLSCQGSTFQKALLISPHRFGEAKGNSAPLIIREPFIACGPKECKHFALTHYAAQPGGYYNGTREDRNKLRHLISVNLGKIPTVENSIFHMAAWSGSACHDGREWTYIGVDGPDSNALIKIKYGEAYTDTYHSYANNILRTQESACNCIGGDCYLMITDGSASGISKCRFLKIREGRIIKEIFPTGRVEHTEECTCGFASNKTIECACRDNSYTAKRPFVKLNVETDTAEIRLMCTETYLDTPRPDDGSITGPCESNGEKGRGGIKGGFVHQRMASKIGRWYSRTMSKTERMGMELYVKYDGDPWTDSDALAPSGVMVSMKEPGWYSFGFEIKDKKCDVPCIGIEMVHDGGKKTWHSAATAIYCLMGSGQLLWDTVTGVDMAL |
| B/Aichi/5/88 | 1988 | LPSTIQTLTLFLTSGGVLLSLYVSALLSYLLYSDILLKFSPTKIIAPTMSLDCANASNVQAVNHSATKEMTFLLPEPEWTYPRLSCQGSTFQKALLISPHRFGEAKGNSAPLIIREPFIACGPKECKHFALTHYAAQPGGYYNGTREDRNKLRHLISVNLGKIPTVENSIFHMAAWSGSACHDGREWTYIGVDGPDSNALIKIKYGEAYTDTYHSYANNILRTQESACNCIGGDCYLMITDGSASGISKCRFLKIREGRIIKEIFPTGRVEHTEECTCGFASNKTIECACRDNSYTAKRPFVKLNVETDTAEIRLMCTETYLDTPRPDDGSITGPCESNGDKGRGGIKGGFVHQRMASKIGRWYSRTMSKTERMGMELYVKYDGDPWTDSDALDPSGVMVSMKEPGWYSFGFEIKDKKCDVPCIGIEMVHDGEKKTWHSAATAIYCLMGSGQLLWDTVTGVDMAL |
| B/Texas/37/1988 | 1988 | LPSTIQTLTLFLTSGGVLLSLYVSASLSYLLYSDILLRFSS-KITAPTMTLDCANASNVQAVNRSATKEMTFLLPEPEWTYPRLSCQGSTFQKALLISPHRFGEARGNSAPLIIREPFIACGPKECKHFALTHYAAQPGGYYNGTREDRNKLRHLISVKLGKIPTVENSIFHMAAWSGSACHDGREWTYIGVDGPDSNALIKIKYGEAYTDTYHSYANNILRTQESACNCIGGDCYLMITDGSASGISECRFLKIREGRIIKEIFPTGRVEHTEECTCGFASNKTIECACRDNSYTAKRPFVKLNVETDTAEIRLMCTETYLDTPRPDDGSITGPCESDGDKGRGGIKGGFVHQRMASKIGRWYSRTMSKTERMGMELYVKYDGDPWTDSDALAPSGVMVSMKEPGWYSFGFEIKDKKCDVPCIGIEMVHDGGKKTWHSAATAIYCLMGSGQLLWDTVTGVDMAL |
| B/Yamagata/16/88 | 1988 | LPSTIQTLTLFLTSGGVLLSLYVSASLSYLLYSDILLKFSPTEITAPKVPLDCANASNVQAVNRSATKGMTLLLSEPEWTYPRLSCQGSTFQKALLISPHRFGETRGNSAPLIIREPFIACGPKECKHFALTHYAAQPGGYYNGTREDRNKLRHLISVKLGKIPTVENSIFHMAAWSGSACHDGREWTYIGLDGPDSNALIKIKYGEAYTDTYHSYANNILRTQESACNCIGGDCYLMITDGSASGISKCRFLKIREGRIIKEIFPTGRVEHTEECTCGFASNKTIECACRDNSYTAKRPFVKLNVETDTAEIRLMCTETYLDTPRPDDGSITGPCESNGDKGRGGIKGGFVHQRMASKIGRWYSRTMSKTERMGMELYVKYDGDPWTDSDALAPSGVMVSMKEPGWYSFGFEIKDKKCDVPCIGIEMVHDGGKETWHSAATAIYCLMGSGQLLWDTVTGVDMAL |
| B/Hong Kong/22/1989 | 1989 | LPSTIQTLTLFLTSGGVLLSLYVSASLSYLLYSDILLKFSPTEITAPTMPLDCANASNVQAVNRSATKGMTLLLPEPEWTYPRLSCPGSTFQKALLISPHRFGETRGNSAPLIIREPFIACGPKECKHFALTHYAAQPGGYYNGTREDRNKLRHLISVKLGKIPTVENSIFHMAAWSGSACHDGREWTYIGVDGPDSNALIKIKYGEAYTDTYHSYANNILRTQESACNCIGGDCYLMITDGSASGISKCRFLKIREGRIIKEIFPTGRVEHTEECTCGFASNKTIECACRDNSYTAKRPFVKLNVETDTAEIRLMCTETYLDTPRPDDGSITGPCESNGDKGRGGIKGGFVHQRMASKIGRWYSRTMSKTERMGMELYVKYDGDPWTDSEALAPSGVMVSMEEPGWYSFGFEIKDKKCDVPCIGIEMVHDGGKKTWHSAATAIYCLMGSGQLLWDTVTGVDMAL |
| B/Memphis/3/89 | 1989 | LPSTIQTLTLFLTSGGVLLSLYVSASLSYLLYSDILLRFSS-KITAPTMTLDCANASNVQAVNRSATKEMTFLLPEPEWTYPRLSCQGSTFQKALLISPHRFGEARGNSAPLIIREPFIACGPKECKHFALTHYAAQPGGYYNGTREDRNKLRHLISVKLGKIPTVENSIFHMAAWSGSACHDGREWTYIGVDGPDSNALIKIKYGEAYTDTYHSYANNILRTQESACNCIGGDCYLMITDGSASGISECRFLKIREGRIIKEIFPTGRVEHTEECTCGFASNKTIECACRDNSYTAKRPFVKLNVETDTAEIRLMCTETYLDTPRPDDGSITGPCESDGDKGRGGIKGGFVHQRMASKIGRWYSRTMSKTERMGMELYVKYDGDPWTDSDALAPSGVMVSMKEPGWYSFGFEIKDKKCDVPCIGIEMVHDGGKKTWHSAATAIYCLMGSGQLLWDTVTGVDMAL |
| B/Nashville/6/89 | 1989 | LPSTIQTLTLFLTSGGVLLSLYVSASLSYLLYSDILLRFSS-KITAPTMTLDCANASNVQAVNRSATKEMTFLLPEPEWTYPRLSCQGSTFQKALLISPHRFGEARGNSAPLIIREPFIACGPKECKHFALTHYAAQPGGYYNGTREDRNKLRHLISVKLGKIPTVENSIFHMAAWSGSACHDGREWTYIGVDGPDSNALIKIKYGEAYTDTYHSYANNILRTQESACNCIGGDCYLMITDGSASGISECRFLKIREGRIIKEIFPTGRVEHTEECTCGFASNKTIECACRDNSYTAKRPFVKLNVETDTAEIRLMCTETYLDTPRPDDGSITGPCESDGDKGRGGIKGGFVHQRMASKIGRWYSRTMSKTERMGMELYVKYDGDPWTDSDALAPSGVMVSMKEPGWYSFGFEIKDKKCDVPCIGIEMVHDGGKKTWHSAATAIYCLMGSGQLLWDTVTGVDMAL |
| B/Bangkok/153/1990 | 1990 | LPSTIQTLTLFLTSGGVLLSLYVSALLSYLLYSDILLKFSP-KIIAPTMSLDCANASNVQAVNHSATKEMTFLLPEPEWTYPRLSCQGSTFQKALLISPHRFGEAKGNSAPLIIREPFIACGPKECKHFALTHYAAQPGGYYNGTREDRNKLRHLISVNLGKIPTVENSIFHMAAWSGSACHDGREWTYIGVDGPDSNALIKIKYGEAYTDTYHSYANNILRTQESACNCIGGDCYLMITDGSASGISKCRFLKIREGRIIKEIFPTGRVEHTEECTCGFASNKTIECACRDNSYTAKRPFVKLNVETDTAEIRLMCTETYLDTPRPDDGSITGPCESNGDKGRGGVKGGFVHQRMASKIGRWYSRTMSKTERMGMELYVKYDGDPWTDSDALAPSGVMVSMEEPGWYSFGFEIKDKKCDVPCIGIEMVHDGGKKTWHSAATAIYCLMGSGQLLWDTVTGVDMAL |
| B/Bangkok/163/1990 | 1990 | LPSTIQTLTLFLTSGGVLLSLYVSALLSYLLYSDILLKFSP-KIIAPTMSLDCANASNVQAVNHSATKEMTFLLPEPEWTYPRLSCQGSTFQKALLISPHRFGEAKGNSAPLIIREPFIACGPKECKHFALTHYAAQPGGYYNGTREDRNKLRHLISVNLGKIPTVENSIFHMAAWSGSACHDGREWTYIGVDGPDSNALIKIKYGEAYTDTYHSYANNILRTQESACNCIGGDCYLMITDGSASGISKCRFLKIREGRIIKEIFPTGRVEHTEECTCGFASNKTIECACRDNSYTAKRPFVKLNVETDTAEIRLMCTETYLDTPRPDDGSITGPCESNGDKGRGGVKGGFVHQRMASKIGRWYSRTMSKTERMGMELYVKYDGDPWTDSDALAPSGVMVSMEEPGWYSFGFEIKDKKCDVPCIGIEMVHDGGKKTWHSAATAIYCLMGSGQLLWDTVTGVDMAL |
| B/Czechoslovakia/69/1990 | 1990 | LPSTIQTLTLFLTSGGVLLSLYVSALLSYLLYSDILLKFSPTKIIAPTMSLDCANASNVQAVNHSATEEMTFLLPEPEWTYPRLSCQGSTFQKALLISPHRFGEAKGNSAPLIIREPFIACGPKECKHFALTHYAAQPGGYYNGTREDRNKLRHLISVNLGKIPTVENSIFHMAAWSGSACHDGREWTYIGVDGPDSNALIKIKYGEAYTDTYHSYANNILRTQESACNCIGGDCYLMITDGSASGISKCRFLKIREGRIIKEIFPTGRVEHTEECTCGFASNKTIECACRDNSYTAKRPFVKLNVETDTAEIRLMCTETYLDTPRPDDGSITGPCESNGGKGSGGIKGGFVHQRMASKIGRWYSRTMSKTKRMGMELYVKYDGDPWTDSDALAPSGVMVSMEEPGWYSFGFEIKDKKCDVPCIGIEMVHDGGKKTWHSAATAIYCLMGSGQLLWDTVTGVDMAL |
| B/Panama/45/1990 | 1990 | LPSTIQTLTLFLTSGGVLLSLYVSASLSYLLYSDILLKFSPTEITAPTMPLDCANASNVQAVNRSATKEMTLLLPEPEWTYPRLSCPGSTFQKALLISPHRFGETRGNSAPLTIREPFIACGPKECKHFALTHYAAQPGGYYNGTREDRNKLRHLISVKLGKIPTVENSIFHMAAWSGSACHDGREWTYIGVDGPDSNALIKIKYGEAYTDTYHSYANNILRTQESACNCIGGDCYLMITDGSASGISKCRFLKIREGRIIKEIFPTGRVEHTEECTCGFASNKTIECACRDNSYTAKRPFVKLNVETDTAEIRLMCTETYLDTPRPDDGSITGPCESNGDKGRGGIKGGFVHQRMASKIGRWYSRTMSKTERMGMELYVKYDGDPWTDSEALAPSGVMVSMEEPGWYSFGFEIKDKKCDVPCIGIEMVHDGGKKTWHSAATAIYCLMGSGQLLWDTVTGVDMAL |
| B/Panama/45/90 | 1990 | LPSTIQTLTLFLTSGGVLLSLYVSASLSYLLYSDILLKFSPTEITAPTMPLDCANASNVQAVNRSATKGMTLLLPEPEWTYPRLSCPGSTFQKALLISPHRFGETRGNSAPLTIREPFIACGPKECKHFALTHYAAQPGGYYNGTREDRNKLRHLISVKLGKIPTVENSIFHMAAWSGSACHDGREWTYIGVDGPDSNALIKIKYGEAYTDTYHSYANNILRTQESACNCIGGDCYLMITDGSASGISKCRFLKIREGRIIKEIFPTGRVEHTEECTCGFASNKTIECACRDNSYTAKRPFVKLNVETDTAEIRLMCTETYLDTPRPDDGSITGPCESNGDKGRGGIKGGFVHQRMASKIGRWYSRTMSKTERMGMELYVKYDGDPWTDSEALAPSGVMVSMEEPGWYSFGFEIKDKKCDVPCIGIEMVHDGGKKTWHSAATAIYCLMGSGQLLWDTVTGVDMAL |
| B/Cordoba/2979/1991 | 1991 | LPSTIQTLTLFLTSGGVLLSLYVSALLSYLLYSDILLKFSP-KIIAPTMSLDCANASNVQAVNHSATKEMTFLLPEPEWTYPRLSCQGSTFQKALLISPHRFGEAKGNSAPLIIREPFIACGPKECKHFALTHYAAQPGGYYNGTREDRNKLRHLISVKLGKIPTVENSIFHMAAWSGSACHDGREWTYIGVDGPDSNALVKIKYGEAYTDTYHSYANNILRTQESACNCIGGDCYLMITDGSASGISKCRFLKIREGRIIKEIFPTGRVEHTEECTCGFASNKTIECACRDNSYTAKRPFVKLNVETDTAEIRLMCTETYLDTPRPDDGSITGPCESNGDKGRGGVKGGFVHQRMASKIGRWYSRTMSKTERMGMELYVKYDGDPWTDSDALAPSGVMVSMEEPGWYSFGFEIKDKKCDVPCIGIEMVHDGGKKTWHSAATAIYCLMGSGQLLWDTVTGVDMAL |
| B/Houston/1/91 | 1991 | LPSTIQTLTLFLTSGGVLLSLYVSALLSYLLYSDILLKFSP-KIIAPTMLLDCANASNVQAVNHSATKEMTFLLPEPEWTYPRLSCQGSTFQKALLISPHRFGEAKGNSAPLIIREPFIACGPKECKHFALTHYAAQPGGYYNGTREDRNKLRHLISVNLGKIPTVENSIFHMAAWSGSACHDGREWTYIGVDGPDSNALIKIKYGEAYTDTYHSYANNILRTQESACNCIGGDCYLMITDGSASGISKCRFLKIREGRIIKEIFPTGRVEHTEECTCGFASNKTIECACRDNSYTAKRPFVKLNVETDTAEIRLMCTETYLDTPRPDDGSITGPCESNGDKGRGGVKGGFVHQRMASKIGRWYSRTMSKTERMGMELYVKYDGDPWTDSDALAPSGVMVSMEEPGWYSFGFEIKDKKCDVPCIGIEMVHDGGKKTWHSAATAIYCLMGSGQLLWDTVTGVDMAL |
| B/Nashville/45/91 | 1991 | LPSTIQTLTLFFTSGGVLLSLYVSALLSYLLYSDILLKFSP-KIIAPTMLLDCANASNVQAVNHSATKEMTFLLPEPEWTYPRLSCQGSTFQKALLISPHRFGETKGNSAPLIIREPFIACGPKECKHFALTHYAAQPGGYYNGTREDRNKLRHLISVNLGKIPTVENSIFHMAAWSGSACHDGREWTYIGVDGPDSNALIKIKYGEAYTDTYHSYANNILRTQESACNCIGGDCYLMITDGSASGISKCRFLKIREGRIIKEIFPTGRVEHTEECTCGFASNKTIECACRDNSYTAKRPFVKLNVETDTAEIRLMCTETYLDTPRPDDGSITGPCESNGDKGRGGVKGGFVHQRMASKIGRWYSRTMSKTERMGMELYVKYDGDPWTDSDALAPSGVMVSMEEPGWYSFGFEIKDKKCDVPCIGIEMVHDGGKKTWHSAATAIYCLMGSGQLLWDTVTGVDMAL |
| B/Nashville/48/91 | 1991 | LPSTIQTLTLFFTSGGVLLSLYVSALLSYLLYSDILLKFSP-KIIAPTMLLDCANASNVQAVNHSATKEMTFLLPEPEWTYPRLSCQGSTFQKALLISPHRFGEAKGNSAPLIIREPFIACGPKECKHFALTHYAAQPGGYYNGTREDRNKLRHLISVNLGKIPTVENSIFHMAAWSGSACHDGREWTYIGVDGPDSNALIKIKYGEAYTDTYHSYANNILRTQESACNCIGGDCYLMITDGSASGISKCRFLKIREGRIIKEIFPTGRVEHTEECTCGFASNKTIECACRDNSYTAKRPFVKLNVETDTAEIRLMCTETYLDTPRPDDGSITGPCESNGDKGRGGVKGGFVHQRMASKIGRWYSRTMSKTERMGMELYVKYDGDPWTDSDALAPSGVMVSMEEPGWYSFGFEIKDKKCDVPCIGIEMVHDGGKKTWHSAATAIYCLMGSGQLLWDTVTGVDMAL |
| B/New York/39/1991 | 1991 | LPSTIQTLTLFLTSGGVLLSLYVSALLSYLLYSDILLKFSP-KIIAPTMLLDCANASNVQAVNHSATKEMTFLLPEPEWTYPRLSCQGSTFQKALLISPHRFGEAKGNSAPLIIREPFIACGPKECKHFALTHYAAQPGGYYNGTREDRNKLRHLISVNLGKIPTVENSIFHMAAWSGSACHDGREWTYIGVDGPDSNALIKIKYGEAYTDTYHSYANNILRTQESACNCIGGDCYLMITDGSASGISKCRFLKIREGRIIKEIFPTGRVEHTEECTCGFASNKTIECACRDNSYTAKRPFVKLNVETDTAEIRLMCTETYLDTPRPDDGSITGPCESNGDKGRGGVKGGFVHQRMASKIGRWYSRTMSKTERMGMELYVKYDGDPWTDSDALAPSGVMVSMEEPGWYSFGFEIKDKKCDVPCIGIEMVHDGGKKTWHSAATAIYCLMGSGQLLWDTVTGVDMAL |
| B/Sichuan/15/1991 | 1991 | LPSTIQTLTLFLTSGGVLLSLYVSASLSYLLYSGILLKFSPTEITAPTMPLDCANASNVQAVNRSATKGVTLLLPEPEWTYPRLSCPGSTFQKALLISPHRFGETRGNSAPLIIREPFIACGPKECKHFALTHYAAQPGGYYNGTREDRNKLRHLISVKLGKIPTVENSIFHMAAWSGSACHDGREWTYIGVDGPDSNALIKIKYGEAYTDTYHSYANNILRTQESACNCIGGDCYLMITDGSASGISKCRFLKIREGRIIKEIFPTGRVEHTEECTCGFASNKTIECACRDNSYTAKRPFVKLNVETDTAEIRLMCTETYLDTPRPDDGSITGPCESNGDKGRGGIKGGFVHQRMASKIGRWYSRTMSKTERMGMELYVKYDGDPWTDSDALALSGVMVSMEEPGWYSFGFEIKDKKCDVPCIGIEMVHDGGKKTWHSAATAIYCLMGSGQLLWDTVTGVDMAL |
| B/Texas/14/1991 | 1991 | LPSTIQTLTLFLTSGGVLLSLYVSALLSYLLYSDILLKFSP-KIIAPTMSLDCANASNVQAVNHSATKEMTFLLPEPEWTYPRLSCQGSTFQKALLISPHRFGEAKGNSAPLIIREPFIACGPKECKHFALTHYAAQPGGYYNGTREDRNKLRHLISVNLGKIPTVENSIFHMAAWSGSACHDGREWTYIGVDGPDSNALIKIKYGEAYTDTYHSYANNILRTQESACNCIGGDCYLMITDGSASGISKCRFLKIREGRIIKEIFPTGRVEHTEECTCGFASNKTIECACRDNSYTAKRPFVKLNVETDTAEIRLMCTETYLDTPRPDDGSITGPCESNGDKGRGGVKGGFVHQRMASKIGRWYSRTMSKTERMGMELYVKYDGDPWTDSDALAPSGVMVSMEEPGWYSFGFEIKDKKCDVPCIGIEMVHDGGKKTWHSAATAIYCLMGSGQLLWDTVTGVDMAL |
| B/Alaska/03/1992 | 1992 | LPSTIQTLTLFLTSGGVLLSLYVSALLSYLLYSDVLLKFSP-KIIAPTMSLDCANASNVQAVNHSATEEMTFLLPEPEWTYPRLSCQGSTFQKALLISPHRFGEAKGNSAPLIIREPFIACGPKECKHFALTHYAAQPGGYYNGTREDRNKLRHLISVNLGKIPTVENSIFHMAAWSGSACHDGREWTYIGVDGPDSNALIKIKYGEAYTDTYHSYANNILRTQESACNCIGGDCYLMITDGSASGISKCRFLKIREGRIIKEIFPTGRVEHTEECTCGFASNKTIECACRDNSYTAKRPFVKLNVETDTAEIRLMCTETYLDTPRPDDGSITGPCESNGDKGHGGIKGGFVHQRMASKIGRWYSRTMSKTKRMGMELYVKYDGDPWTDSDALAPSGVMVSMEEPGWYSFGFEIKDKKCDVPCIGIEMVHDGGKKTWHSAATAIYCLMGSGQLLWDTVTGVDMAL |
| B/Guangzhou/86/92 | 1992 | LPSTIQTLTLFLTSGGVLLSLYVSASLSYLLYSDILLKFSPTEITAPTMPLDCANASNVQAVNRSATKGVTLLLPEPEWTYPRLSCPGSTFQKALLISPHRFGETRGNSAPLIIREPFIACGPKECKHFALTHYAAQPGGYYNGTREDRNKLRHLISVKLGKIPTVENSIFHMAAWSGSACHDGREWTYIGVDGPDSNALLKIKYGEAYTDTYHSYANNILRTQESACNCIGGDCYLMITDGSASGISKCRFLKIREGRIIKEIFPTGRVEHTEEWTCGFASNKTLECACRDNSYTAKRPFVKLNVETDTAEIRLMCTETYLDTPRPDDGSITGPCESNGDKGRGGIKGGFVHQRMASKIGRWYSRTMSKTERMGMELYVKYDGDPWTDSDALALGGVMVSMEEPGWYSFGFEIKDKKCDVPCIGIEMVHDGGKKTWHSAATAIYCLMGSGQLLWDTVTGVDMTL |
| B/Guangzhou/87/92 | 1992 | LPSTIQTLTLFLTSGGVLLSLYVSASLSYLLYSDILLKFSPTEITAPTMPLDCANASNVQAVNRSATKGVTLLLPEPEWTYPRLSCPGSTFQKALLISPHRFGETRGNSAPLIIREPFIACGPKECKHFALTHYAAQPGGYYNGTREDRNKLRHLISVKLGKIPTVENSIFHMAAWSGSACHDGREWTYIGVDGPDSNALLKIKYGEAYTDTYHSYANNILRTQESACNCIGGDCYLMITDGSASGISKCRFLKIREGRIIKEIFPTGRVEHTEECTCGFASNKTIECACRDNSYTAKRPFVKLNVETDTAEIRLMCTETYLDTPRPDDGSITGPCESNGDKGRGGIKGGFVHQRMASKIGRWYSRTMSKTERMGMELYVKYDGDPWTDSDALALGGVMVSMEEPGWYSFGFEIKDKKCDVPCIGIEMVHDGGKKTWHSAATAIYCLMGSGQLLWDTVTGVDMTL |
| B/Hawaii/1/92 | 1992 | LPSTIQTLTLFLTSGGVLLSLYVSALLSYLLYSDVLLKFSP-KIIAPTMSLDCANASNVQAVNHSATEEMTFLLPEPEWTYPRLSCQGSTFQKALLISPHRFGEAKGNSAPLIIREPFIACGPKECKHFALTHYAAQPGGYYNGTREDRNKLRHLISVNLGKIPTVENSIFHMAAWSGSACHDGREWTYIGVDGPDSNALIKIKYGEAYTDTYHSYANNILRTQESACNCIGGDCYLMITDGSASGISECRFLKIREGRIIKEIFPTGRVEHTEECTCGFASNKTIECACRDNSYTAKRPFVKLNVETDTAEIRLMCTETYLDTPRPDDGSITGPCESNGDKGHGGIKGGFVHQRMASKIGRWYSRTMSKTKRMGMELYVKYDGDPWTDSDALAPSGVMVSMEEPGWYSFGFEIKDKKCDVPCIGIEMVHDGGKKTWHSAATAIYCLMGSGQLLWDTVTGVDMAL |
| B/Hong Kong/03/1992 | 1992 | LPSTIQTLTLFLTSGGVLLSLYVSASLSYLLYSGVLLKFSPTEITAPTMPLDCANASNVQAVNRSATKGVTLLLPEPEWTYPRLSCPGSTFQKALLISPHRFGETRGNSAPLIIREPFIACGPKECKHFALTHYAAQPGGYYNGTREDRNKMRHLISVKLGKIPTVENSIFHMAAWSGSACHDGREWTYIGVDGPDSNALIKIKYGEAYTDTYHSYANNILRTQESACNCIGGDCYLMITDGSASGISKCRFLKIREGRIIKEIFPTGRVEHTEECTCGFASNKTIECACRDNSYTAKRPFVKLNVETDTAEIRLMCTETYLDTPRPDDGSITGPCESNGDKGLGGIKGGFVHQRMASKIGRWYSRTMSKTERMGMELYVKYDGDPWTDSDALALSGVMVSMEEPGWYSFGFEIKDKKCDVPCIGIEMVHDGGKKTWHSAATAIYCLMGSGQLLWDTVTGVDMAL |
| B/Oita/15/92 | 1992 | LPSTIQTLTLFLTSGGVLLSLYVSALLSYLLYSDILLKFSP-KIIAPTISLDCANASNVQAVNHSATKEMTFLLPEPEWTYPRLSCQGSIFQKALLISPHRFGEAKGNSAPLIIREPFIACGPKECKHFALTHYAAQPGGYYNGTREDRNKLRHLISVNLGKIPTVENSIFHMAAWSGSACHDGREWTYIGVDGPDSNALIKIKYGEAYTDTYHSYANNILRTQESACNCIGGDCYLMITDGSASGISKCRFLKIREGRIIKEIFPTGRVEHTEECTCGFASNKTIECACRDNSYTAKRPFVKLNVETDTAEIRLMCTETYLDTPRPDDGSITGPCESNGDKGRGGVKGGFVHQRIASKIGRWYSRTMSKTERMGMELYVKYDGDPWTDSDALAPSGVMVSMEEPGWYSFGFEIKDKKCDVPCIGIEMVHDGGKKTWHSAATAIYCLMGSGQLLWDTVTGVDMAL |
| B/Sichuan/8/92 | 1992 | LPSTIQTLTLFLTSGGVLLSLYVSASLSYLLYSDILLKFSPTEITAPTMPLDCANASNVQAVNRSATKGVTLLLPEPEWTYPRLSCPGSTFQKALLISPHRFGETKGNSAPLIIREPFIACGPKECKHFALTHYAAQPGGYYNGTREDRNKLRHLISVKLGKIPTVENSIFHMAAWSGSACHDGREWTYIGVDGPDSNALLKIKYGEAYTDTYHSYANNILRTQESACNCIGGDCYLMITDGSASGISKCRFLKIREGRIIKEIFPTGRVEHTEECTCGFASNKTIECACRDNSYTAKRPFVKLNVETDTAEIRLMCTETYLDTPRPDDGSITGPCESNGDKGRGGIKGGFVHQRMASKIGRWYSRTMSKTERMGMELYVKYDGDPWTDSDALALSGVMVSMEEPGWYSFGFEIKDKKCDVPCIGIEMVHDGGKKTWHSAATAIYCLMGSGQLLWDTVTGVDMAL |
| B/Aichi/1/93 | 1993 | LPSTIQTLTLFLTSGGVLLSLYVSASLSYLLYSDILLKFSPTEITAPPMPLDCANASNVQAVNRSATKGVTLLLPEPEWTYPRLSCPGSTFQKALLISPHRFGETRGNSAPLIIREPFIACGPKECKHFAITHYAAQPGGYYNGTREDRNKLRHLISVKLGKIPTVENSIFHMAAWSGSACHDGREWTYIGVDGPDSNALLKIKYGEAYTDTYHSYANNILRTQESACNCIGGDCYLMITDGSASGISKCRFLKIREGRIIKEIFPTGRVEHTEECTCGFASNKTIECACRDNSYTAKRPFVKLNVETDTAEIRLMCTETYLDTPRPDDGSITGPCESNGDKGRGGIKGGFVHQRMASKIGRWYSRTMSKTDRMGMELYVKYDGDPWTDSDALALSGVMVSMEEPGWYSFGFEIKDKKCDVPCIGIEMVHDGGKKTWHSAATAIYCLMGSGQLLWDTVTGVDMAL |
| B/Beijing/184/93 | 1993 | LPSTIQTLTLFLTSGGVLLSLYVSASLSYLLYSDILLKFSPTEITAPTMPLDCANASNVQAVNRSATKGVTLLLPEPEWTYPRLSCPGSTFQKALLISPHRFGETRGNSAPLIIREPFIACGPKECKHFALTHYAAQPGGYYNGTREDRNKLRHLISVKLGKIPTVENSIFHMAAWSGSACHDGREWTYIGVDGPDSNALLKIKYGEAYTDTYHSYANNILRTQESACNCIGGDCYLMITDGSASGTSKCRFLKIREGRIIKEIFPTGRVEHTEECTCGFASNKTIECACRDNNYTAKRPFVKLNVETDTAEIRLMCTETYLDTPRPDDGSITGPCESNGDKGRGGIKGGFVHQRMASKIGRWYSRTMSKTERMGMELYVKYDGDPWTDSDALALSGVMVSMEEPGWYSFGFEIKDKKCDVPCIGIEMVHDGGKKTWHSAATAIYCLMGSGQLLWDTVTGVDMAL |
| B/Connecticut/07/1993 | 1993 | LPSTIQTLTLFLTSGGVLLSLYVSALLSYLLYSDVLLKFSP-KIIAPTMSLDCANASNVQAVNHSATEEMTFLLPEPEWTYPRLSCQGSTFQKALLISPHRFGEAKGNSAPLIIREPFIACGPKECKHFALTHYAAQPGGYYNGTREDRNKLRHLISVNLGKIPTVENSIFHMAAWSGSACHDGREWTYIGVDGPDSNALIKIKYGEAYTDTYHSYANNILRTQESACNCIGGDCYLMITDGSASGISKCRFLKIREGRIIKEIFPTGRVEHTEECTCGFASNKTIECACRDNSYTAKRPFVKLNVETDTAEIRLMCTETYLDTPRPDDGSITGPCESNGDKGHGGIKGGFVHQRMASKIGRWYSRTMSKTKRMGMELYVKYDGDPWTDSDALAPSGVMVSMEEPGWYSFGFEIRDKKCDVPCIGIEMVHDGGKKTWHSAATAIYCLMGSGQLLWDTVTGVDMAL |
| B/Guangdong/8/93 | 1993 | LPSTIQTLTLFLTSGGVLLSLYVSALLSYSLYSDILLKFSPTKTIAPTMSLDCANASNVQAVNHSATKGMTLLLPEPEWTYPRLSCQGSTFQKALLISPHRFGETKGNSAPLIIREPFIACGPKECKHFALTHYAAQPGGYYNGTREDRNKLRHLISVSLGKIPTVENSIFHMAAWSGSACHDGREWTYIGVDGPDSNALIKIKYGEAYTDTYHSYANNILRTQESACNCIGGDCYLMITDGSASGISKCRFLKIREGRIIKEIFPTGRVDHTEECTCGFASNKTIECACRDNSYTAKRPFVKLNVETDTAEIRLMCTETYLDTPRPDDGSITGPCESNGDKGRGGIKGGFVHQRMASKIGRWYSRTMSKTERLGMELYVKYDGDPWTDSDALAPSGVMVSAEEPGWYSFGFEIKDKKCDVPCIGIEMVHDGGKKTWHSAATAIYCLMGSGQLLWDTVTGVDMAL |
| B/Hong Kong/02/1993 | 1993 | LPSTIQTLTLFLTSGGVLLSLYVSALLSYSLYSDILLKFSPTKTIAPTMSLDCANASNVQAVNHSATKGMTLLLPEPEWTYPRLSCQGSTFQKALLISPHRFGETKGNSAPLIIREPFIACGPKECKHFALTHYAAQPGGYYNGTREDRNKLRHLISVSLGKIPTVENSIFHMAAWSGSACHDGREWTYIGVDGPDSNALIKIKYGEAYTDTYHSYANNILRTQESACNCIGGDCYLMITDGSASGISKCRFLKIREGRIIKEIFPTGRVDHTEECTCGFASNKTIECACRDNSYTAKRPFVKLNVETDTAEIRLMCTETYLDTPRPDDGSITGPCESNGDKGRGGIKGGFVHQRMASKIGRWYSRTMSKTERLGMELYVKYDGDPWTDSDALAPSGVMVSAEEPGWYSFGFEIKDKKCDVPCIGIEMVHDGGKKTWHSAATAIYCLMGSGQLLWDTVTGVDMAL |
| B/Houston/2/93 | 1993 | LPSTIQTLTLFLTSGGVLLSLYVSALLSYLLYSDVLLKFSP-KIIAPTMSLDCANASNVQAVNHSATEEMTFLLPEPEWTYPRLSCQGSTFQKALLISPHRFGEAKGNSAPLIIREPFIACGPKECKHFALTHYAAQPGGYYNGTREDRNKLRHLISVNLGKIPTVENSIFHMAAWSGSACHDGREWTYIGVDGPDSNALLKIKYGEAYTDTYHSYANNILRTQESACNCIGGDCYLMITDGSASGISECRFLKIREGRIIKEIFPTGRVEHTEECTCGFASNKTIECACRDNSYTAKRPFVKLNVETDTAEIRLMCTETYLDTPRPDDGSITGPCESNGDKGHGGIKGGFVHQRMASKIGRWYSRTMSKTKRMGMELYVKYDGDPWTDSDALAPSGVMVSMEEPGWYSFGFEIKDKKCDVPCIGIEMVHDGGKKTWHSAATAIYCLMGSGQLLWDTVTGVDMAL |
| B/Memphis/4/93 | 1993 | LPSTIQTLTLFLTSGGVLLSLYVSALLSYLLYSDILLKFSP-KIIAPTMSLDCANASNVQAVNHSATKEMTFLLPEPEWTYPRLSCQGSTFQKALLISPHRFGEAKGNSAPLIIREPFIACGPKECKHFALTHYAAQPGGYYNGTREDRNKLRHLISVNLGKIPTVENSIFHMAAWSGSACHDGREWTYIGVDGPDSNALIKIKYGEAYTDTYHSYANNILRTQESACNCIGGDCYLMITDGSASGISKCRFLKIREGRIIKEIFPTGRVEHTEECTCGFASNKTIECACRDNSYTAKRPFVKLNVETDTAEIRLMCTETYLDTPRPDDGSITGPCESNGDKGRGGVKGGFVHQRMASKIGRWYSRTMSKTERMGMELYVKYDGDPWTDSDALAPSGVMVSMEEPGWYSFGFEIKDKKCDVPCIGIEMVHDGGKKTWHSAATAIYCLMGSGQLLWDTVTGVDMAL |
| B/Memphis/5/93 | 1993 | LPSTIQTLTLFLTSGGVLLSLYVSALLSYLLYSDVLLKFSP-KIIAPTMSLDCANASNVQAVNHSATEEMTFLLPEPEWTYPRLSCQGSTFQKALLISPHRFGEAKGNSAPLIIREPFIACGPKECKHFALTHYAAQPGGYYNGTREDRNKLRHLISVNLGKIPTVENSIFHMAAWSGSACHDGREWTYIGVDGPDSNALIKIKYGEAYTDTYHSYANNILRTQESACNCIGGDCYLMITDGSASGISKCRFLKIREGRIIKEIFPTGRVEHTEECTCGFASNKTIECACRDNSYTAKRPFVKLNVETDTAEIRLMCTETYLDTPRPDDGSITGPCESNGDKGHGGIKGGFVHQRMASKIGRWYSRTMSKTKRMGMELYVKYDGDPWIDSDALAPSGVMVSMEEPGWYSFGFEIKDKKCDVPCIGIEMVHDGGKKTWHSAATAIYCLMGSGQLLWDTVTGVDMAL |
| B/Mie/01/1993 | 1993 | LPSTIQTLTLFLTSGGVLLSLYVSASLSYLLYSDILLKFSPTEITAPPMPLDCANASNVQAVNRSATKGVTLLLPEPEWTYPRLSCPGSTFQKALLISPHRFGETRGNSAPLIIREPFIACGPKECKHFAITHYAAQPGGYYNGTREDRNKLRHLISVKLGKIPTVENSIFHMAAWSGSACHDGREWTYIGVDGPDSNALLKIKYGEAYTDTYHSYANNILRTQESACNCIGGDCYLMITDGSASGISKCRFLKIREGRIIKEIFPTGRVEHTEECTCGFASNKTIECACRDNSYTAKRPFVKLNVETDTAEIRLMCTETYLDTPRPDDGSITGPCESNGDKGRGGIKGGFVHQRMASKIGRWYSRTMSKTDRMGMELYVKYDGDPWTDSDALALSGVMVSMEEPGWYSFGFEIKDKKCDVPCIGIEMVHDGGKKTWHSAATTIYCLMGSGQLLWDTVTGVDMAL |
| B/Nanchang/26/93 | 1993 | LPSTIQTLTLFLTSGGVLLSLYVSASLSYLLYSDILLKFSPTEITAPTMPLDCANASNVQAVNRSATKGGTLLLPEPEWTYPRLSCPGSTFQKALLISPHRFGETRGNSAPLIIREPFIACGPKECKHFALTHYAAQPGGYYNGTREDRNKLRHLISVKLGKIPTVENSIFHMAAWSGSACHDGREWTYIGVDGPDSNALLKIKYGEAYTDTYHSYANNILRTQESACNCIGGDCYLMITDGSASGISKCRFLKIREGRIIKEIFPTGRVEHTEECTCGFASNKTIECACRDNSYTAKRPFVKLNVETDTAEIRLMCTETYLDTPRPDDGSITGPCESNGDKGRGGIKGGFVHQRMASKIGRWYSRTMSKTERMGMELYVKYDGDPWTDSDALALSGVMVSMEEPGWYSFGFEIKDKKCDVPCIGIEMVHDGGKKTWHSAATAIYCLMGSGQLLWDTVTGVDMAL |
| B/Nashville/107/93 | 1993 | LPSTIQTLTLFLTSGGVLLSLYVSALLSYLLYSDVLLKFSP-KIIAPTMSLDCANASNVQAVNHSATEEMTFLLPEPEWTYPRLSCQGSTFQKALLISPHRFGEAKGNSAPLIIREPFIACGPKECKHFALTHYAAQPGGYYNGTREDRNKLRHLISVNLGKIPTVENSIFHMAAWSGSACHDGREWTYIGVDGPDSNALIKIKYGEAYTDTYHSYANNILRTQESACNCIGGDCYLMITDGSASGISKCRFLKIREGRIIKEIFPTGRVEHTEECTCGFASNKTIECACRDNSYTAKRPFVKLNVETDTAEIRLMCTETYLDTPRPDDGSITGPCESNGDKGHGGIKGGFVHQRMASKIGRWYSRTMSKTKRMGMELYVKYDGDPWTDSDALAPSGVMVSMEEPGWYSFGFEIKDKKCDVPCIGIEMVHDGGKKTWHSAATAIYCLMGSGQLLWDTVTGVDMAL |
| B/New York/1000/1993 | 1993 | LPSTIQTLTLFLTSGGVLLSLYVSALLSYLLYSDILLKFSP-KIIAPTMSLDCANASNVQAVNHSATKEMKFLPPEPEWTYPRLSCQGSTFQKALLISPHRFGEAKGNSAPLIIREPFIACGPKECKHFALTHYAAQPGGYYNGTREDRNKLRHLISVNLGKIPTVENSIFHMAAWSGSACHDGREWTYIGVDGPDSNALIKIKYGEAYTDTYHSYANNILRTQESACNCIGGDCYLMITDGSASGISKCRFLKIREGRIIKEIFPTGRVEHTEECTCGFASNKTIECACRDNSYTAKRPFVKLNVETDTAEIRLMCTETYLDTPRPDDGSITGPCESNGDKGRGGVKGGFVHQRMASKIGRWYSRTMSKTKRMGMELYVKYDGDPWTDSDALAPSGVMVSMEEPGWYSFGFEIKDKKCDVPCIGIEMVHDGGKRTWHSAATAIYCLMGSGQLLWDTVTGVDMAL |
| B/New York/1001/1993 | 1993 | LPSTIQTLTLFLTSGGVLLSLYVSALLSYLLYSDILLKFSP-KIIAPTMSLDCANASNVQAVNHSATKEMKFLPSEPEWTYPRLSCQGSTFQKALLISPHRFGEAKGNSAPLIIREPFIACGPKECKHFALTHYAAQPGGYYNGTREDRNKLRHLISVNLGKIPTVENSIFHMAAWSGSACHDGREWTYIGVDGPDSNALIKIKYGEAYTDTYHSYANNILRTQESACNCIGGDCYLMITDGSASGISKCRFLKIREGRIIKEIFPTGRVEHTEECTCGFASNKTIECACRDNSYTAKRPFVKLNVETDTAEIRLMCTETYLDTPRPDDGSITGPCESNGDKGRGGVKGGFVHQRMASKIGRWYSRTMSKTKRMGMELYVKYDGDPWTDSDALAPSGVMVSMEEPGWYSFGFEIKDKKCDVPCIGIEMVHDGGKRTWHSAATAIYCLMGSGQLLWDTVTGVDMAL |
| B/New York/1002/1993 | 1993 | LPSTIQTLTLFLTSGGVLLSLYVSALLSYLLYSDVLLKFSP-KIIAPTMSLDCANASNVQAVNHSATEEMTFLLPEPEWTYPRLSCQGSTFQKALLISPHRFGEAKGNSAPLIIREPFIACGPKECKHFALTHYAAQPGGYYNGTREDRNKLRHLISVNLGKIPTVENSIFHMAAWSGSACHDGREWTYIGVDGPDSNALIKIKYGEAYTDTYHSYANNILRTQESACNCIGGDCYLMITDGSASGISKCRFLKIREGRIIKEIFPTGRVEHTEECTCGFASNKTIECACRDNIYTAKRPFVKLNVETDTAEIRLMCTETYLDTPRPDDGSITGPCESNGDKGHGGIKGGFVHQRMASKIGRWYSRTMSKTKRMGMELYVKYDGDPWTDSDALAPSGVMVSMEEPGWYSFGFEIKDKKCDVPCIGIEMVHDGGKKTWHSAATAIYCLMGSGQLLWDTVTGVDMAL |
| B/New York/1003/1993 | 1993 | LPSTIQTLTLFLTSGGVLLSLYVSTLLSYLLYSDILLKFSP-KIIAPTTSLDCANASNVQAVNHSATKEMKFLPTEPEWTYPRLSCQGSTFQKALLISPHRFGEAKGNSAPLIIREPFIACGPKECKHFALTHYAAQPGGYYNGTREDRNKLRHLISVNLGKIPTVENSIFHMAAWSGSACHDGREWTYIGVDGPDSNALIKIKYGEAYTDTYHSYANNILRTQESACNCIGGDCYLMITDGSASGISKCRFLKIREGRIIKEIFPTGRVEHTEECTCGFASNKTIECACRDNSYTAKRPFVKLNVETDTAEIRLMCTETYLDTPRPDDGSITGPCESNGDKGSGGVKGGFVHQRMTSKIGRWYSRTMSKTKRMGMELYVKYDGDPWTDSDALAPSGVMVSMEEPGWYSFGFEIKDKKCDVPCIGIEMVHDGGKRTWHSAATAIYCLMGSGQLLWDTVTGVDMAL |
| B/New York/24/1993 | 1993 | LPSTIQTLTLFLTSGGVLLSLYVSALLSYLLYSDVLLKFSP-KMIAPTMSLDCANASNVQAVNHSATEEMTFLLPEPEWTYPRLSCQGSTFQKALLISPHRFGEAKGNSAPLIIREPFIACGPKECKHFALTHYAAQPGGYYNGTREDRNKLRHLISVNLGKIPTVENSIFHMAAWSGSACHDGREWTYIGVDGPDSNALIKIKYGEAYTDTYHSYANNILRTQESACNCIGGDCYLMITDGSASGISKCRFLKIREGRIIKEIFPTGRVEHTEECTCGFASNKTIECACRDNSYTAKRPFVKLNVETDTAEIRLMCTETYLDTPRPDDGSITGPCESNGDKGHGGIKGGFVHQRMASKIGRWYSRTMSKTKRMGMELYVKYDGDPWTDSDALAPSGVMVSMEEPGWYSFGFEIKDKKCDVPCIGIEMVHDGGKKTWHSAATAIYCLMGSGQLLWDTVTGVDMAL |
| B/Bangkok/141/1994 | 1994 | LPSTIQTLTLFLTSGGVLLSLYVSASLSYLLYSDILLKFSPTEITAPTMPLDCANASNVQAVNRSATKGVTLLLPEPEWTYPRLSCPGSTFQKALLISPHRFGETRGNSAPLIIREPFIACGPKECKHFALTHYAAQPGGYYNGTREDRNKLRHLISVKLGKIPTVENSIFHMAAWSGSACHDGREWTYIGVDGPDSNALLKIKYGEAYTDTYHSYANNILRTQESACNCIGGDCYLMITDGSASGISKCRFLKIREGRIIKEIFPTGRVEHTEECTCGFASNKTIECACRDNSYTAKRPFVKLNVETDTAEIRLMCTETYLDTPRPDDGSITGPCESNGDKGLGGIKGGFVHQRMASKIGRWYSRTMSKTKRMGMELYVKYDGDPWTDSDALALSGVMVPMEEPGWYSFGFEIKDKKCDVPCIGIEMVHDGGKKTWHSAATAIYCLMGSGQLLWDTVTGVDMAL |
| B/Beijing/1/1994 | 1994 | LPSTIQTLTLFLTSGGVLLSLYVSASLSYLLYSDILLKFSPTEITAPTMPLDCANASNVQAVNRSATKGVTLLLPEPEWTYPRLSCPGSTFQKALLISPHRFGETRGNSAPLIIREPFIACGPKECKHFALTHYAAQPGGYYNGTREDRNKLRHLISVKLGKIPTVENSIFHMAAWSGSACHDGREWTYIGVDGPDSNALLKIKYGEAYTDTYHSYANNILRTQESACNCIGGDCYLMITDGSASGISKCRFLKIREGRIIKEIFPTGRVEHTEECTCGFASNKTIECACRDNSYTAKRPFVKLNVETDTAEIRLMCTETYLDTPRPDDGSITGPCESNGDKGSGGIKGGFVHQRMASKIGRWYSRTMSKTKRMGMELYVKYDGDPWTDSDALALSGVMVSMEEPGWYSFGFEIKDKKCDVPCIGIEMVHDGGKKTWHSAATAIYCLMGSGQLLWDTVTGVDMAL |
| B/Beijing/6/1994 | 1994 | LPSTIQTLTLFLTSGGVLLSLYVSASLSYLLYSDILLKFSPTEITAPTMPLDCANASSVQTVNRSATKGVTLLLPEPEWTYPRLSCPGSTFQKALLISPHRFGETRGNSAPLIIREPFIACGPKECKHFALTHYAAQPGGYYNGTREDRNKLRHLISVKLGKIPTVENSIFHMAAWSGSACHDGREWTYIGVDGPDSNALLKIKYGEAYTDTYHSYANNILRTQESACNCIGGDCYLMITDGSASGISKCRFLKIREGRIIKEIFPTGRVEHTEECTCGFASNKTIECACRDNSYTAKRPFVKLNVETDTAEIRLMCTETYLDTPRPDDGSITGPCESNGDKGLGGIKGGFVHQRMASKIGRWYSRTMSKTKRMGMELYVKYDGDPWTDSDALALSGVMVPMEEPGWYSFGFEIKDKKCDVPCIGIEMVHDGGKNTWHSAATAIYCLMGSGQLLWDTVTGVDMAL |
| B/California/02/1994 | 1994 | LPSTIQTLTLFLTSGGVLLSLYVSASLSYLLYSDILLKFSPTEITAPTMPLDCANASNVQAVNRSATKGVTLLLPEPEWTYPRLSCPGSTFQKALLISPHRFGETRGNSAPLIIREPFIACGPKECKHFALTHYAAQPGGYYNGTREDRNKLRHLISVKLGKIPTVENSIFHMAAWSGSACHDGREWTYIGVDGPDSNALLKIKYGEAYTDTYHSYANNILRTQESACNCIGGDCYLMITDGSASGISKCRFLKIREGRIIKEIFPTGRVEHTEECTCGFASNKTIECACRDNSYTAKRPFVKLNVETDTAEIRLMCTETYLDTPRPDDGSITGPCESNGDKGSGGIKGGFVHQRMASKIGRWYSRTMSKTKRMGMGLYVKYDGDPWTDSDALALSGVMVSMEEPGWYSFGFEIKDKKCDVPCIGIEMVHDGGKKTWHSAATAIYCLMGSGQLLWDTVTGVDMAL |
| B/Guangdong/5/94 | 1994 | LPSTIQTLTLFLTSGGVLLSLYVSALLSYLLYSDVLLKFSPTKIIAPTMSLDCANASNVQAVNHSATEEMTFLLPEPEWTYPRLSCQGSTFQKALLISPHRFGEAKGNSAPLIIREPFIACGPKECKHFALTHYAAQPGGYYNGTREDRNKLRHLISVNLGKIPTVENSIFHMAAWSGSACHDGREWTYIGVDGPDSNALIKIEYGEAYTDTYHSYAYNILRTQESACNCIGGDCYLMITDGSASGISKCRFLKIREGRIIKEIFPTGRVEHTEECTCGFASNKTIECACRDNSYTAKRPFVKLNVETDTAEIRLMCTETYLDTPRPDDGSITGPCESNGDKGSGGIKGGFVHQRMASKIGRWYSRTMSKTKRMGMELYVKYDGDPWTDSDALAPSGVMVSMEEPGWYSFGFEIKDKKCDVPCIGIEMVHDGGKKTWHSAATAIYCLMGSGQLLWDTVTGVDMAL |
| B/Harbin/7/1994 | 1994 | LPSTIQTLTLFLTSGGVLLSLYVSASLSYLLYSDILLKFSPTEITAPTMPLDCANASNVQAVNRSATKGVTLLLPEPEWTYPRLSCPGSTFQKALLISPHRFGETRGNSAPLIIREPFIACGPKECKHFALTHYAAQPGGYYNGTREDRNKLRHLISVKLGKIPTVENSIFHMAAWSGSACHDGREWTYIGVDGPDSNALLKIKYGEAYTDTYHSYANNILRTQESACNCIGGDCYLMITDGSASGISKCRFLKIREGRIIKEIFPTGRVEHTEECTCGFASNKTIECACRDNSYTAKRPFVKLNVETDTAEIRLMCTETYLDTPRPDDGSITGPCESNGDKGSGGIKGGFVHQRMASKIGRWYSRTMSKTKRMGMGLYVKYDGDPWTDSDALALSGVMVSMEEPGWYSFGFEIKDKKCDVPCIGIEMVHDGGKKTWHSAATAIYCLMGSGQLLWDTVTGVDMAL |
| B/Johannesburg/06/1994 | 1994 | LPSTIQTLTLFLTLGGVLLSLYVSALLSYLLYSDVLLKFSP-KMIAPTMSLDCANASNVQAVNHSATEEMTFLLPEPEWTYPRLSCQGSTFQKALLISPHRFGEAKGNSAPLIIREPFIACGPKECKHFALTHYAAQPGGYYNGTREDRNKLRHLISVNLGKIPTVENSIFHMAAWSGSACHDGREWTYIGVDGPDSNALIKIKYGEAYTDTYHSYANNILRTQESACNCIGGDCYLMITDGSASGISKCRFLKIREGRIIKEIFPTGRVEHTEECTCGFASNKTIECACRDNSYTAKRPFVKLNVETDTAEIRLMCTETYLDTPRPDDGSITGPCESNGDKGHGGIKGGFVHQRMASKIGRWYSRTMSKTKRMGMELYVKYDGDPWTDSDALAPSGVMVSMEEPGWYSFGFEIKDKKCDVPCIGIEMVHDGGKKTWHSAATAIYCLMGSGQLLWDTVTGVDMAL |
| B/Lisbon/02/1994 | 1994 | LPSTIQTLTLFLTSGGVLLSLYVSALLSYLLYSDILLKFSP-KIIAPTTSLDCANASNVQAVNHSATKEMKFLPPEPEWTYPRLSCQGSTFQKALLISPHRFGEAKGNSAPLIIREPFIACGPKECKHFALTHYAAQPGGYYNGTREDRNKLRHLISVNLGKIPTVENSIFHMAAWSGSACHDGREWTYIGVDGPDSNALIKIKYGEAYTDTYHSYANNILRTQESACNCIGGDCYLMITDGSASGISKCRFLKIREGRIIKEIFPTGRVEHTEECTCGFASNKTIECACRDNSYTAKRPFVKLNVETDTAEIRLMCTETYLDTPRPDDGSITGPCESNGDKGSGGVKGGFVHQRMASKIGRWYSRTMSKTKRMGMELYVKYDGDPWTDSDALAPSGVMVSMEEPGWYSFGFEIKDKKCDVPCIGIEMVHDGGKRTWHSAATAIYCLMGSGQLLWDTVTGVNMAL |
| B/Nanchang/195/94 | 1994 | LPSTIQTLTLFLTSGGVLLSLYVSASLSYLLYSDILLKFSPTEITAPTMPLDCANATNVQAVNRSATKGVTLLLPEPEWTYPRLSCPGSTFQKALLISPHRFGETRGNSAPLIIREPFIACGPKECKHFALTHYAAQPGGYYNGTREDRNKLRHLISVKLGKIPTVENSIFHMAAWSGSACHDGREWTYIGVDGPDSNALLKIKYGEAYTDTYHSYANNILRTQESACNCIGGDCYLMITDGSASGISKCRFLKIREGRIIKEIFPTGRVEHTEECTCGFASNKTIECACRDNSYTAKRPFVKLNVETDTAEIRLMCTETYLDTPRPDDGSITGPCESNGDKGSGGIKGGFVHQRMASKIGRWYSRTMSKTKRMGMELYVKYDGDPWTDSDALALSGVMVSMEEPGWYSFGFEIKDKKCDVPCIGIEMVHDGGKKTWHSAATAIYCLMGSGQLLWDTVTGVDMAL |
| B/Nanchang/480/94 | 1994 | LPSTIQTLTLFLTSGGVLLSLYVSASLSYLLYSDILLKFSPTEITAPTMPLDCANASNVQAVNRSAPKGATLLFPEPEWTYPRLSCPGSTFQKALLISPHRFGETRGNSAPLIIREPFIACGPKECKHFALTHYAAQPGGYYNGTREDRNKLRHLISVKLGKIPTVENSIFHMAAWSGSACHDGREWTYIGVDGPDSNALLKIKYGEAYTDTYHSYANNILRTQESACNCIGGDCYLMITDGSASGISKCRFLKIREGRIIKEIFPTGRVEHTEECTCGFASNKTIECACIDNSYTAKRPFVKLNVETDTAEIRLMCTETYLDTPRPDDGSITGPCESNGDKGSGGIKGGFVHQRMASKIGRWYSRTMSKTKRMGMGLYVKYDGDPWTDSDALALSGVMVSMEEPGWYSFGFEIKDKKCDVPCIGIEMVHDGGKNTWHSAATAIYCLMGSGQLLWDTVTGVDMAL |
| B/Nanchang/560/94 | 1994 | LPSTIQTLTLFLTSGGVLLSLYVSASLSYSLYSDILLKFSPTEKSAPTMSLDCANASNVQAVNRSATKGMTLLLPEPEWTYPRLSCQGSTFQKALLISPHRFGETRGNSAPLIIREPFIACGPKECKHFALTHYAAQPGGYYNGTREDRNKLRHLISVKLGKIPTVENSIFHMAAWSGSACHDGREWTYIGVDGPDSNALIKIKYGEAYTDTYHSYANNILRTQESACNCIGGDCYLMITDGSASGISKCRFLKIREGRIIKEIFPTGRVDHTEECTCGFASNKTIECACRDNSYTAKRPFVKLNVETDTAEIRLMCTETYLDTPRPDDGSKTGPCESNGDKGSGGIKGGFVHQRMASKIGRWYSRTMSKTERMGMELYVKYDGDPWTDSDALAPSGVMVSMEEPGWYSFGFEIKDKKCDVPCIGIEMVHDGGKNTWHSAATAIYCLMGSGQLLWDTVTGVDMAL |
| B/Nanchang/630/94 | 1994 | LPSTIQTLTLFLTSGGVLLSLYVSASLSYLLYSDILLKFSPTEITAPTMPLDCANASNVQAVNRSATKGVTLLFPEPEWTYPRLSCPGSTFQKALLISPHRFGETRGNSAPLIIREPFIACGPKECKHFALTHYAAQPGGYYNGTREDRNKLRHLISVKLGKIPTVENSIFHMAAWSGSACHDGREWTYIGVDGPDSNALLKIKYGEAYTDTYHSYANNILRTQESACNCIGGDCYLMITDGSASGISKCRFLKIREGRIIKEIFPTGRVDHTEECTCGFASNKTIECACRDNSYTAKRPFVKLNVETDTAEIRLMCTETYLDTPRPDDGSITGPCESNGDKGSGGIKGGFVHQRMASKIGRWYSRTMSKTKRMGMELYVKYDGDPWTDSDALALSGVMVSMEEPGWYSFGFEIKDKKCDVPCIGIEMVHDGGKNTWHSAATAIYCLMGSGQLLWDTVTGVDMAL |
| B/Shanghai/4/1994 | 1994 | LPSTIQTLTLFLTSGGVLLSLYVSASLSYLLYSDMLLKFSQTEITAPTIPLDCANASNVQAVNRSATKGVTLLLPEPEWTYPRLSCPGSTFQKALLISPHRFGETRGNSAPLIIREPFIACGPKECKHFALTHYAAQPGGYYNGTREDRNKLRHLISVKLGKIPTVENSIFHMAAWSGSACHDGREWTYIGVDGPDSNALLKIKYGEAYTDTYHSYANNILRTQESACNCIGGDCYLMITDGSASGISKCRFLKIREGRIIKEIFPTGRVEHTEECTCGFASNKTIECACRDNSYTAKRPFVKLNVETDTAEIRLMCTETYLDTPRPDDGSITGPCESNGDKGLGGIKGGFVHQRMASKIGRWYSRTMSKTKRMGMELYVKYDGDPWTDSDALALSGVMVPMEEPGWYSFGFEIKDKKCDVPCIGIEMVHDGGKKTWHSAATAIYCLMGSGQLLWDTVTGVDMAL |
| B/Singapore/11/94 | 1994 | LPSTIQTLTLFLTSGGVLLSLYVSASLSYLLYSGILLKFSPTEITAPTMPLDCANASNVQAVNRSATKGVTLLLPEPEWTYPRLSCPGSTFQKALLISPHRFGETRGNSAPLIIREPFIACGPKECKHFALTHYAAQPGGYYNGTREDRNKLRHLISVKLGKIPTVENSIFHMAAWSGSACHDGREWTYIGVDGPDSNALLKIKYGEAYTDTYHSYANNILRTQESACNCIGGDCYLMITDGSASGISKCRFLKIREGRIIKEIFPTGRVEHTEECTCGFASNKTIECACRDNSYTAKRPFVKLNVETDTAEIRLMCTETYLDTPRPDDGSITGPCESNGDKGSGGIKGGFVHQRMASKIGRWYSRTMSKTKRMGMGLYVKYDGDPWTDSDALALSGVMVSMEEPGWYSFGFEIKDKKCDVPCIGIEMVHDGGKKTWHSAATAIYCLMGSGQLLWDTVTGVDMAL |
| B/Taiwan/1197/94 | 1994 | LPSTIQTLTLFLTSGGVLLSLYVSASLSYLLYSDILLKFSPTKITAPTMPLDCANASNVQAVNHSATKGVTLLLPEPEWTYPRLSCPGSTFQKALLISPHRFGETRGNSAPLIIREPFIACGPKECKHFALTHYAAQPGGYYNGTREDRNKLRHLISVKLGKIPTVENSIFHMAAWSGSACHDGREWTYIGVDGPDSNALLKIKYGEAYTDTYHSYANNILRTQESACNCIGGDCYLMITDGSASGISECRFLKIREGRIIKEIFPTGRVEHTEECTCGFASNKTIECACRDNSYTAKRPFVKLNVETDTAEIRLMCTETYLDTPRPDDGSITGPCESNGDKGSGGIKGGFVHQRMASKIGRWYSRTMSKTKRMGMGLYVKYDGDPWTDSDALALSGVMVSMEEPGWYSFGFEIKDKKCDVPCIGIEMVHDGGKKTWHSAATAIYCLMGSGQLLWDTVTGVDMAL |
| B/Wellington/01/1994 | 1994 | LPSTIQTLTLFLTSGGVLLSLYVSASLSYLLYSGILLKFSPTEITAPTMPLDCANASNVQAVNRSATKGVTLLLPEPEWTYPRLSCPGSTFQKALLISPHRFGETRGNSAPLIIREPFIACGPKECKHFALTHYAAQPGGYYNGTREDRNKLRHLISVKLGKIPTVENSIFHMAAWSGSACHDGREWTYIGVDGPDSNALLKIKYGEAYTDTYHSYANNILRTQESACNCIGGDCYLMITDGSASGISKCRFLKIREGRIIKEIFPTGRVEHTEECTCGFASNKTIECACRDNSYTAKRPFVKLNVETDTAEIRLMCTETYLDTPRPDDGSITGPCESNGDKGSGGIKGGFVHQRMASKIGRWYSRTMSKTKRMGMGLYVKYDGDPWTDSDALALSGVMVSMEEPGWYSFGFEIKDKKCDVPCIGIEMVHDGGKKTWHSAATAIYCLMGSGQLLWDTVTGVDMAL |
| B/California/01/1995 | 1995 | LPSTIQTLTLFLTSGGVLLSLYVSASLSYLLYSDILLKFSPTEITAPTMPLDCANASNVQAVNRSATKGVTLLLPEPEWTYPRLSCPGSTFQKALLISPHRFGETRGNSAPLIIREPFIACGPKECKHFALTHYAAQPGGYYNGTREDRNKLRHLISVKLGKIPTVENSIFHMAAWSGSACHDGREWTYIGVDGPDSNALLKIKYGEAYTDTYHSYANNILRTQESACNCIGGDCYLMITDGSASGISKCRFLKIREGRIIKEIFPTGRVEHTEECTCGFASNKTIECACRDNSYTAKRPFVKLNVETDTAEIRLMCTETYLDTPRPDDGSITGPCESNGDKGSGGIKGGFVHQRMASKIGRWYSRTMSKTKRMGMGLYVKYDGDPWTDSDALALSGVMVSMEEPGWYSFGFEIKDKKCDVPCIGIEMVHDGGKKTWHSAATAIYCLMGSGQLLWDTVTGVDMAL |
| B/California/02/1995 | 1995 | LPSTIQTLTLFLTSGGVLLSLYVSASLSYLLYSDILLKFSPTEITAPTMPLDCANASNVQAVNRSATKGVTLLLPEPEWTYPRLSCPGSTFQKALLISPHRFGETKGNSAPLIIREPFIACGPKECKHFALTHYAAQPGGYYNGTREDRNKLRHLISVKLGKIPTVENSIFHMAAWSGSACHDGREWTYIGVDGPDSNALLKIKYGEAYTDTYHSYANNILRTQESACNCIGGDCYLMITDGSASGISECRFLKIREGRIIKEIFPTGRVEHTEECTCGFASNKTIECACRDNSYTAKRPFVKLNVETDTAEIRLMCTETYLDTPRPDDGSITGPCESNGDKGSGGIKGGFVHQRMASKIGRWYSRTMSKTKRMGMGLYVKYDGDPWTDSDALALSGVMVSMEEPGWYSFGFEIKDKKCDVPCIGIEMVHDGGKKTWHSAATAIYCLMGSGQLLWDTVTGVDMAL |
| B/Connecticut/02/1995 | 1995 | LPSTIQTLTLFLTSGGVLLSLYVSALLSYLLYSDILLKFSP-KIIAPTTSLDCANASNVQAVNHSATKEMKFLPPEPEWTYPRLSCQGSTFQKALLISPHRFGEAKGNSAPLIIREPFIACGPKECKHFALTHYAAQPGGYYNGTREDRNKLRHLISVNLGKIPTVENSIFHMAAWSGSACHDGREWTYIGVDGPDSNALIKIKYGEAYTDTYHSYANNILRTQESACNCIGGDCYLMITDGSASGISKCRFLKIREGRIIKEIFPTGRVEHTEECTCGFASNKTIECACRDNSYTAKRPFVKLNVETDTAEIRLMCTETYLDTPRPDDGSITGPCESNGDKGSGGVKGGFVHQRMASKIGRWYSRTMSKTKRMGMELYVKYDGDPWTDSDALAPSGVMVSMEEPGWYSFGFEIKDKKCDVPCIGIEMVHDGGKRTWHSAATAIYCLMGSGQLLWDTVTGVNMAL |
| B/hanfang/263/1995 | 1995 | LPSTIQTLTLFLTSGGVLLSLYVSASLSYLLYSDILLKFSPTEITAPTMPLDCANASNVQAVNRSATKGVTLLLPEPEWTYPRLSCPGSTFQKALLISPHRFGETRGNSAPLIIREPFIACGPKECKHFALTHYAAQPGGYYNGTREDRNKLRHLISVKLGKIPTVENSIFHMAAWSGSACHDGREWTYIGVDGPDSNALLKIKYGEAYTDTYHSYANNILRTQESACNCIGGDCYLMITDGSASGISKCRFLKIREGRIIKEIFPTGRVEHTEECTCGFASNKTIECACRDNSYTAKRPFVKLNVETDTAEIRLMCTETYLDTPRPDDGSITGPCESNGDKGSGGVKGGFVHQRMASKIGRWYSRTMSKTKRMGMGLYVKYDGDPWTDSDALALSGVMVSMEEPGWYSFGFEIKDKKCDVPCIGIEMVHDGGKNTWHSAATAIYCLMGSGQLLWDTVTGVDMAL |
| B/Indiana/01/1995 | 1995 | LPSTIQTLTLFLTSGGVLLSLYVSASLSYLLYSDILLKFSPTEITAPTMPLDCANASNVQAVNRSATKGVTLLLPEPEWTYPRLSCPGSTFQKALLISPHRFGETKGNSAPLIIREPFIACGPKECKHFALTHYAAQPGGYYNGTREDRNKLRHLISVKLGKIPTVENSIFHMAAWSGSACHDGREWTYIGVDGPDSNALLKIKYGEAYTDTYHSYANNILRTQESACNCIGGDCYLMITDGSASGISECRFLKIREGRIIKEIFPTGRVEHTEECTCGFASNKTIECACRDNSYTAKRPFVKLNVETDTAEIRLMCTETYLDTPRPDDGSITGPCESNGDKGSGGIKGGFVHQRMASKIGRWYSRTMSKTKRMGMGLYVKYDGDPWTDSDALALSGVMVSMEEPGWYSFGFEIKDKKCDVPCIGIEMVHDGGKKTWHSAATAIYCLMGSGQLLWDTVTGVDMAL |
| B/Malaysia/06535/1995 | 1995 | LPSTIQTLTLFLTSGGVLLSLYVSASLSYLLYSDILLKFSPTEITAPTMPLDCANASNVQAVNRSATKGVTLLLPEPEWTYPRLSCPGSTFQKALLISPHRFGETKGNSAPLIIREPFIACGPKECKHFALTHYAAQPGGYYNGTREDRNKLRHLISVKLGKIPTVENSIFHMAAWSGSACHDGREWTYIGVDGPDSNALLKIKYGEAYTDTYHSYANNILRTQESACNCIGGDCYLMITDGSASGISECRFLKIREGRIIKEIFPTGRVEHTEECTCGFASNKTIECACRDNSYTAKRPFVKLNVETDTAEIRLMCTETYLDTPRPDDGSITGPCESNGDKGSGGIKGGFVHQRMASKIGRWYSRTMSKTKRMGMGLYVKYDGDPWTDSDALALSGVMVSMEEPGWYSFGFEIKDKKCDVPCIGIEMVHDGGKKTWHSAATAIYCLMGSGQLLWDTVTGVDMAL |
| B/Memphis/18/95 | 1995 | LPSTIQTLTLFLTSGGVLLSLYVSALLSYLLYSDILLKFSP-KIIAPTTSLDCANASNVQAVNHSATKEMKFLPPEPEWTYPRLSCQGSTFQKALLISPHRFGEAKGNSAPLIIREPFIACGPKECKHFALTHYAAQPGGYYNGTREDRNKLRHLISVNLGKIPTVENSIFHMAAWSGSACHDGREWTYIGVDGPDSNALIKIKYGEAYTDTYHSYANNILRTQESACNCIGGDCYLMITDGSASGISKCRFLKIREGRIIKEIFPTGRVEHTEECTCGFASNKTIECACRDNSYTAKRPFVKLNVETDTAEIRLMCTETYLDTPRPDDGSITGPCESNGDKGSGGVKGGFVHQRMASKIGRWYSRTMSKTKRMGMELYVKYDGDPWTDSDALAPSGVMVSMEEPGWYSFGFEIKDKKCDVPCIGIEMVHDGGKRTWHSAATAIYCLMGSGQLLWDTVTGVNMAL |
| B/Nanchang/15/95 | 1995 | LPSTIQTLTLFLTSGGVLLSLYVSASLSYLLYSDILLKFSPTEITAPTMPLDCANASNVQAVNRSATKGVTLLLPEPEWTYPRLSCPGSTFQKALLISPHRFGETRGNSAPLIIREPFIACGPKECKHFALTHYAAQPGGYYNGTREDRNKLRHLISVKLGKIPTVENSIFHMAAWSGSACHDGREWTYIGVDGPDSNALLKIKYGEAYTDTYHSYANNILRTQESACNCIGGDCYLMITDGSASGISKCRFLKIREGRIIKEIFPTGRVEHTEECTCGFASNKTIECACRDNSYTAKRPFVKLNVETDTAEIRLMCTETYLDTPRPDDGSITGPCESNGDKGSGGVKGGFVHQRMASKIGRWYSRTMSKTKRMGMGLYVKYDGDPWTDSDALALSGVMVSMEEPGWYSFGFEIKDKKCDVPCIGIEMVHDGGKNTWHSAATAIYCLMGSGQLLWDTVTGVDMAL |
| B/Nanchang/3/95 | 1995 | LPSTIQTLTLFLTSGGVLLSLYVSALLSYLLYSDILLKFSPTETTAPTMPLDWANASNVQAVNRSATKGVTLLFPEPEWTYPRLSCPGSTFQKALLISPHRFGETRGNSAPLIIREPFIACGPKECKHFALTHYAAQPGGYYNGTREDRNKLRHVISVKLGKIPTVENSIFHMAAWSGSACHDGREWTYIGVDGPDSNALIKIKYGEAYTDTYHSYANNILRTQESACNCIGGDCYLMITDGSASGISKCRFLKIREGRIIKEIFPTGRVDHTEECTCGFASNKTIECACRDNSYTAKRPFVKLNVETDTAEIRLMCTETYLDTPRPDDGSITGPCESNGDKGSGGIKGGFVHQRMASKIGRWYSRTMSKTKRMGMELYVKYDGDPWTDSDALAPSGVMVSMEEPGWYSFGFEIKDKKCDVPCIGIEMVHDGGKNTWHSAATAIYCLMGSGQLLWDTVTGVDMAL |
| B/Nanchang/8/95 | 1995 | LPSTIQTLTLFLTSGGVLLSLYVSALLSYSLYSDILLKFSPTKTIAPTMSLDCANASNVQAVNHSATKGMTLLLPEPEWTYPRLSCQGSTFQKALLISPHRFGETKGNSAPLIIREPFIACGPKECKHFALTHYAAQPGGYYNGTREDRNKLRHLISVSLGKIPTVENSIFHMAAWSGSACHDGREWTYIGVDGPDSNALIKIKYGEAYTDTYHSYANNILRTQESACNCIGGDCYLMITDGSASGISRCRFLKIREGRIIKEIFPTGRVEHTEECTCGFASNKTIECACRDNSYTAKRPFVKLNVETDTAEIRLMCTETYLDTPRPDDGSTTGPCESNGDKGRGGIKGGFVHQRMASKIGRWYSRTMSKTERLGMELYVKYDGDPWTDSDALAPSGVMVSAEEPGWYSFGFEIKDKKCDVPCIGIEMVHDGGKKTWHSAATAIYCLMGSGQLLWDTVTGVDMAL |
| B/New York/1004/1995 | 1995 | LPSTIQTLTLFLTSGGVLLSLYVSASLSYLLYSGILLKFSPTEITAPTMPLDCANASNVQAVNRSATKGVTLLLPEPEWTYPRLSCPGSTFQKALLISPHRFGETRGNSAPLIIREPFIACGPKECKHFALTHYAAQPGGYYNGTREDRNKLRHLISVKLGKIPTVENSIFHMAAWSGSACHDGREWTYIGVDGPDSNALLKIKYGEAYTDTYHSYANNILRTQESACNCIGGDCYLMITDGSASGISKCRFLKIREGRIIKEIFPTGRVEHTEECTCGFASNKTIECACRDNSYTAKRPFVKLNVETDTAEIRLMCTETYLDTPRPDDGSITGPCESNGDKGSGGIKGGFVHQRMASKIGRWYSRTMSKTKRMGMGLYVKYDGDPWTDSDALALSGVMVSMEEPGWYSFGFEIKDKKCDVPCIGIEMVHDGGKKTWHSAATAIYCLMGSGQLLWDTVTGVDMAL |
| B/New York/1005/1995 | 1995 | LPSTIQTLTLFLTSGGVLLSLYVSASLSYLLYSDILLKFSPTEITAPTMPLDCANASNVQAVNRSATKGVTLLLPEPEWTYPRLSCPGSTFQKALLISPHRFGETKGNSAPLIIREPFIACGPKECKHFALTHYAAQPGGYYNGTREDRNKLRHLISVKLGKIPTVENSIFHMAAWSGSACHDGREWTYIGVDGPDSNALLKIKYGEAYTDTYHSYANNILRTQESACNCIGGDCYLMITDGSASGISECRFLKIREGRIIKEIFPTGRVEHTEECTCGFASNKTIECACRDNSYTAKRPFVKLNVETDTAEIRLMCTETYLDTPRPDDGSITGPCESNGDKGSGGIKGGFVHQRMASKIGRWYSRTMSKTKRMGMGLYVKYDGDPWTDSDALALSGVMVSMEEPGWYSFGFEIKDKKCDVPCIGIEMVHDGGKKTWHSAATAIYCLMGSGQLLWDTVTGVDMAL |
| B/New York/1006/1995 | 1995 | LPSTIQTLTLFLTSGGVLLSLYVSASLSYLLYSDILLKFSPTEITAPTMPLDCANASNVQAVNRSATKGVTLLLPEPEWTYPRLSCPGSTFQKALLISPHRFGETKGNSAPLIIREPFIACGPKECKHFALTHYAAQPGGYYNGTREDRNKLRHLISVKLGKIPTVENSIFHMAAWSGSACHDGREWTYIGVDGPDSNALLKIKYGEAYTDTYHSYANNILRTQESACNCIGGDCYLMITDGSASGISECRFLKIREGRIIKEIFPTGRVEHTEECTCGFASNKTIECACRDNSYTAKRPFVKLNVETDTAEIRLMCTETYLDTPRPDDGSITGPCESNGDKGSGGIKGGFVHQRMASKIGRWYSRTMSKTKRMGMGLYVKYDGDPWTDSDALALSGVMVSMEEPGWYSFGFEIKDKKCDVPCIGIEMVHDGGKKTWHSAATAIYCLMGSGQLLWDTVTGVDMAL |
| B/New York/1007/1995 | 1995 | LPSTIQTLTLFLTSGGVLLSLYVSASLSYLLYSGILLKFSPTEITAPTMPLDCANASNVQAVNRSATKGVTLLLPEPEWTYPRLSCPGSTFQKALLISPHRFGETRGNSAPLIIREPFIACGPKECKHFALTHYAAQPGGYYNGTREDRNKLRHLISVKLGKIPTVENSIFHMAAWSGSACHDGREWTYIGVDGPDSNALLKIKYGEAYTDTYHSYANNILRTQESACNCIGGDCYLMITDGSASGISKCRFLKIREGRIIKEIFPTGRVEHTEECTCGFASNKTIECACRDNSYTAKRPFVKLNVETDTAEIRLMCTETYLDTPRPDDGSITGPCESNGDKGSGGIKGGFVHQRMASKIGRWYSRTMSKTKRMGMGLYVKYDGDPWTDSDALALSGVMVSMEEPGWYSFGFEIKDKKCDVPCIGIEMVHDGGKKTWHSAATAIYCLMGSGQLLWDTVTGVDMAL |
| B/New York/1008/1995 | 1995 | LPSTIQTLTLFLTSGGVLLSLYVSASLSYLLYSGILLKFSPTEITAPTMPLDCANASNVQAVNRSATKGVTLLLPEPEWTYPRLSCPGSTFQKALLISPHRFGETRGNSAPLIIREPFIACGPKECKHFALTHYAAQPGGYYNGTREDRNKLRHLISVKLGKIPTVENSIFHMAAWSGSACHDGREWTYIGVDGPDSNALLKIKYGEAYTDTYHSYANNILRTQESACNCIGGDCYLMITDGSASGISKCRFLKIREGRIIKEIFPTGRVEHTEECTCGFASNKTIECACRDNSYTAKRPFVKLNVETDTAEIRLMCTETYLDTPRPDDGSITGPCESNGDKGSGGIKGGFVHQRMASKIGRWYSRTMSKTKRMGMGLYVKYDGDPWTDSDALALSGVMVSMEEPGWYSFGFEIKDKKCDVPCIGIEMVHDGGKKTWHSAATAIYCLMGSGQLLWDTVTGVDMAL |
| B/New York/1009/1995 | 1995 | LPSTIQTLTLFLTSGGVLLSLYVSALLSYLLYSDILLKFSP-KIIAPTTSLDCANASNVQAVNHSATKEMKFLPPEPEWTYPRLSCQGSTFQKALLISPHRFGEAKGNSAPLIIREPFIACGPKECKHFALTHYAAQPGGYYNGTREDRNKLRHLISVNLGKIPTVENSIFHMAAWSGSACHDGREWTYIGVDGPDSNALIKIKYGEAYTDTYHSYANNILRTQESACNCIGGDCYLMITDGSASGISKCRFLKIREGRIIKEIFPTGRVEHTEECTCGFASNKTIECACRDNSYTAKRPFVKLNVETDTAEIRLMCTETYLDTPRPDDGSITGPCESNGDKGSGGVKGGFVHQRMASKIGRWYSRTMSKTKRMGMELYVKYDGDPWTDSDALAPSGVMVSMEEPGWYSFGFEIKDKKCDVPCIGIEMVHDGGKRTWHSAATAIYCLMGSGQLLWDTVTGVNMAL |
| B/New York/1010/1995 | 1995 | LPSTIQTLTLFLTSGGVLLSLYVSASLSYLLYSDILLKFSPTEITAPTMPLDCANASNVQAVNRSATKGVTLLLPEPEWTYPRLSCPGSTFQKALLISPHRFGETKGNSAPLIIREPFIACGPKECKHFALTHYAAQPGGYYNGTREDRNKLRHLISVKLGKIPTVENSIFHMAAWSGSACHDGREWTYIGVDGPDSNALLKIKYGEAYTDTYHSYANNILRTQESACNCIGGDCYLMITDGSASGINECRFLKIREGRIIKEIFPTGRVEHTEECTCGFASNKTIECACRDNSYTAKRPFVKLNVETDTAEIRLMCTETYLDTPRPDDGSITGPCESNGDKGSGGIKGGFVHQRMASKIGRWYSRTMSKTRRMGMGLYVKYDGDPWTDSDALALSGVMVSMEEPGWYSFGFEIKDKKCDVPCIGIEMVHDGGKKTWHSAATAIYCLMGSGQLLWDTVTGVDMAL |
| B/New York/1011/1995 | 1995 | LPSTIQTLTLFLTSGGVLLSLYVSASLSYLLYSDILLKFSPTEITAPTMPLDCANASNVQAVNRSATKGVTLLLPEPEWTYPRLSCPGSTFQKALLISPHRFGETKGNSAPLIIREPFIACGPKECKHFALTHYAAQPGGYYNGTREDRNKLRHLISVKLGKIPTVENSIFHMAAWSGSACHDGREWTYIGVDGPDSNALLKIKYGEAYTDTYHSYANNILRTQESACNCIGGDCYLMITDGSASGINECRFLKIREGRIIKEIFPTGRVEHTEECTCGFASNKTIECACRDNSYTAKRPFVKLNVETDTAEIRLMCTETYLDTPRPDDGSITGPCESNGDKGSGGIKGGFVHQRMASKIGRWYSRTMSKTRRMGMGLYVKYDGDPWTDSDALALSGVMVSMEEPGWYSFGFEIKDKKCDVPCIGIEMVHDGGKKTWHSAATAIYCLMGSGQLLWDTVTGVDMAL |
| B/New York/1012/1995 | 1995 | LPSTIQTLTLFLTSGGVLLSLYVSASLSYLLYSDILLKFSPTEITAPTMPLDCANASNVQAVNRSATKGVTLLLPEPEWTYPRLSCPGSTFQKALLISPHRFGETKGNSAPLIIREPFIACGPKECKHFALTHYAAQPGGYYNGTREDRNKLRHLISVKLGKIPTVENSIFHMAAWSGSACHDGREWTYIGVDGPDSNALLKIKYGEAYTDTYHSYANNILRTQESACNCIGGDCYLMITDGSASGISECRFLKIREGRIIKEIFPTGRVEHTEECTCGFASNKTIECACRDNSYTAKRPFVKLNVETDTAEIRLMCTETYLDTPRPDDGSITGPCESNGDKGSGGIKGGFVHQRMASKIGRWYSRTMSKTKRMGMGLYVKYDGDPWTDSDALALSGVMVSMEEPGWYSFGFEIKDKKCDVPCIGIEMVHDGGKKTWHSAATAIYCLMGSGQLLWDTVTGVDMAL |
| B/New York/1013/1995 | 1995 | LPSTIQTLTLFLTSGGVLLSLYVSASLSYLLYSDILLKFSPTEITAPTMPLDCANASNVQAVNRSATKGVTLLLPEPEWTYPRLSCPGSTFQKALLISPHRFGETKGNSAPLIIREPFIACGPKECKHFALTHYAAQPGGYYNGTREDRNKLRHLISVKLGKIPTVENSIFHMAAWSGSACHDGREWTYIGVDGPDSNALLKIKYGEAYTDTYHSYANNILRTQESACNCIGGDCYLMITDGSASGISECRFLKIREGRIIKEIFPTGRVEHTEECTCGFASNKTIECACRDNSYTAKRPFVKLNVETDTAEIRLMCTETYLDTPRPDDGSITGPCESNGDKGSGGIKGGFVHQRMASKIGRWYSRTMSKTKRMGMGLYVKYDGDPWTDSDALALSGVMVSMEEPGWYSFGFEIKDKKCDVPCIGIEMVHDGGKKTWHSAATAIYCLMGSGQLLWDTVTGVDMAL |
| B/Russia/22/1995 | 1995 | LPSTIQTLTLFLTSGGVLLSLYVSASLSYLLYSDILLKFSPTEITAPTMPLDCANASNVQAVNRSATKGVTLLLPEPEWTYPRLSCPGSTFQKALLISPHRFGETKGNSAPLIIREPFIACGPKECKHFALTHYAAQPGGYYNGTREDRNKLRHLISVKLGKIPTVENSIFHMAAWSGSACHDGREWTYIGVDGPDSNALLKIKYGEAYTDTYHSYANNILRTQESACNCIGGDCYLMITDGSASGISECRFLKIREGRIIKEIFPTGRVEHTEECTCGFASNKTIECACRDNSYTAKRPFVKLNVETDTAEIRLMCTETYLDTPRPDDGSITGPCESNGDKGSGGIKGGFVHQRMASKIGRWYSRTMSKTKRMGMGLYVKYDGDPWTDSDALALSGVMVSMEEPGWYSFGFEIKDKKCDVPCIGIEMVHDGGKKTWHSAATAIYCLMGSGQLLWDTVTGVDMAL |
| B/Alaska/12/96 | 1996 | LPSTIQTLTLFLTSGGVLLSLYVSASLSYLLYSDILLKFSPTEITAPTMPLDCANASNVQAVNRSATKGVTLLLPEPEWTYPRLSCPGSTFQKALLISPHRFGETKGNSAPLIIREPFIACGPKECKHFALTHYAAQPGGYYNGTREDRNKLRHLISVKLGKIPTVENSIFHMAAWSGSACHDGREWTYIGVDGPDSNALLKIKYGEAYTDTYHSYANNILRTQESACNCIGGNCYLMITDGSASGISECRFLKIREGRIIKEIFPTGRVEHTEECTCGFASNKTIECACRDNSYTAKRPFVKLNVETDTAEIRLMCTETYLDTPRPDDGSITGPCESNGDKGSGGIKGGFVHQRMASKIGRWYSRTMSKTKRMGMGLYVKYDGDPWTDSDALALSGVMVSMEEPGWYSFGFEIKDKKCDVPCIGIEMVHDGGKKTWHSAATAIYCLMGSGQLLWDTVTGVDMAL |
| B/Florida/1/96 | 1996 | LPSTIQTLTLFLTSGGVLLSLYVSASLSYLLYSDILLKFSPTEITAPTIPLDCANASNVQAVNRSATKGATLLLPEPEWTYPRLSCPGSTFQKALLISPHRFGETKGNSAPLIIREPFIACGPKECKHFALTHYAAQPGGYYNGTREDRNKLRHLISVKLGKIPTVENSIFHMAAWSGSACHDGREWTYIGVDGPDSNALLKIKYGEAYTDTYHSYANNILRTQESACNCIGGDCYLMITDGSASGISECRFLKIREGRIIKEIFPTGRVEHTEECTCGFASNKTIECACRDNSYTAKRPFVKLNVETDTAEIRLMCTETYLDTPRPDDGSITGPCESNGDKGSGGIKGGFVHQRMASKIGRWYSRTMSKTKRMGMGLYVKYDGDPWTDSDALALSGVMVSMEEPGWYSFGFEIKDKKCDVPCIGIEMVHDGGKKTWHSAATAIYCLMGSGQLLWDTVTGVDMAL |
| B/Hong Kong/70/1996 | 1996 | LPSTIQTLTLFLTSGGVLLSLYVSALLSYLLYSDILLKFSPTKIIAPTMSLDCANASNVQAVNHSATEEMTFLLPEPEWTYPRLSCQGSTFQKALLISPHRFGEAKGNSAPLIIREPFIACGPKECKHFALTHYAAQPGGYYNGTREDRNKLRHLISVNLGKIPTVENSIFHMAAWSGSACHDGREWTYIGVDGPDSNALIKIKYGEAYTDTYHSYANNILRTQESACNCIGGDCYLMITDGSASGISKCRFLKIREGRIIKEIFPTGRVEHTEECTCGFASNKTIECACRDNSYTAKRPFVKLNVETDTAEIRLMCTETYLDTPRPDDGSITGPCESNGDKGSGGIKGGFVHQRMASKIGRWYSRTMSKTKRMGMELYVKYDGDPWTDSDALAPSGVMVSMEEPGWYSFGFEIKDKKCDVPCIGIEMVHDGGKKTWHSAATAIYCLMGSGQLLWDTVTGVDMAL |
| B/jinfang/33/1996 | 1996 | LPSTIQTLTLFLTSGGVLLSLYVSASLSYLLYSGILLKFSPTEITAPTMPLDCANASNVQAVNRSATKGVTLLLPEPEWTYPRLSCPGSTFQKALLISPHRFGETRGNSAPLIIREPFIACGPKECKHFALTHYAAQPGGYYNGTREDRNKLRHLISVTLGKIPTVENSIFHMAAWSGSACHDGREWTYIGVDGPDSNALLKIKYGEAYTDTYHSYANNILRTQESACNCIGGDCYLMITDGSASEISKCRFLKIREGRIIKEIFPTGRVEHTEECTCGFASNKTIECACRDNSYTAKRPFVKLNVETDTAEIRLMCTETYLDTPRPDDGSITGPCESNGDKGSGGIKGGFVHQRMASKIGRWYSRTMSKTKRMGMGLYVKYDGDPWTDSDALALSGVMVSMEEPGWYSFGFEIKDKKCDVPCIGIEMVHDGGKKTWHSAATAIYCLMGSGQLLWDTVTGVDMAL |
| B/jingfang/39/1996 | 1996 | LPSTIQTLTLFLTSGGVLLSLYVSASLSYLLYSGILLKFSPTEITAPTMPLDCANASNVQAVNRSATKGVTLLLPEPEWTYPRLSCPGSTFQKALLISPHRFGETRGNSAPLIIREPFIACGPKECKHFALTHYAAQPGGYYNGTREDRNKLRHLISVKLGKIPTVENSIFHMAAWSGSACHDGREWTYIGVDGPDSNALLKIKYGEAYTDTYHSYANNILRTQESACNCIGGDCYLMITDGSASEISKCRFLKIREGRIIKEIFPTGRVEHTEECTCGFASNKTIECACRDNSYTAKRPFVKLNVETDTAEIRLMCTETYLDTPRPDDGSITGPCESNGDKGSGGIKGGFVHQRMASKIGRWYSRTMSKTKRMGMGLYVKYDGDPWTDSDALALSGVMVSMEEPGWYSFGFEIKDKKCDVPCIGIEMVHDGGKKTWHSAATAIYCLMGSGQLLWDTVTGVDMAL |
| B/jingfang/53/1996 | 1996 | LPSTIQTLTLFLTSGGVLLSLYVSASLSYLLYSDILLKFSPTEITAPTMPLDCANASNVQAVNRSATKGVTLLLPEPEWTYPRLSCPGSTFQKALLISPHRFGETKGNSAPLIIREPFIACGPKECKHFALTHYAAQPGGYYNGTREDRNKLRHLISVKLGKIPTVENSIFHMAAWSGSACHDGREWTYIGVDGPDSNALLKIKYGEAYTDTYHSYANNILRTQESACNCIGGDCYLMITDGSASGISECRFLKIREGRIIKEIFPTGRVEHTEECTCGFASNKTIECACRDNSYTAKRPFVKLNVETDTAEIRLMCTETYLDTPRPDDGSITGPCESNGDKGSGGIKGGFVHQRMASKIGRWYSRTMSKTKRMGMGLYVKYDGDPWTDSDALALSGVMVSMEEPGWYSFGFEIKDKKCDVPCIGIEMVHDGGKKTWHSAATAIYCLMGSGQLLWDTVTGVDMAL |
| B/Malaysia/10837/1996 | 1996 | LPSTIQTLTLFLTSGGVLLSLYVSASLSYLLYSDILLKFSPTEITAPTMPLDCANASNVQAVNRSATKGVTLLLPEPEWTYPRLSCPGSTFQKALLISPHRFGETKGNSAPLIIREPFIACGPKECKHFALTHYAAQPGGYYNGTREDRNKLRHLISVKLGKIPTVENSIFHMAAWSGSACHDGREWTYIGVDGPDSNALLKIKYGEAYTDTYHSYANNILRTQESACNCIGGNCYLMITDGSASGISECRFLKIREGRIIKEIFPTGRVEHTEECTCGFASNKTIECACRDNSYTAKRPFVKLNVETDTAEIRLMCTETYLDTPRPDDGSITGPCESNGDKGSGGIKGGFVHQRMASKIGRWYSRTMSKTKRMGMGLYVKYDGDPWTDSDALALSGVMVSMEEPGWYSFGFEIKDKKCDVPCIGIEMVHDGGKKTWHSAATAIYCLMGSGQLLWDTVTGVDMAL |
| B/Malaysia/10928/1996 | 1996 | LPSTIQTLTLFLTSGGVLLSLYVSASLSYLLYSDILLKFSPTEITAPTMPLDCANASNVQAVNRSATKGVTLLLPEPEWTYPRLSCPGSTFQKAILISPHRFGETKGNSAPLIIREPFIACGPKECKHFALTHYAAQPGGYYNGTREDRNKLRHLISVKLGKIPTVENSIFHMAAWSGSACHDGREWTYIGVDGPDSNALLKIKYGEAYTDTYHSYANNILRTQESACNCIGGNCYLMITDGSASGISECRFLKIREGRIIKEIFPTGRVEHTEECTCGFASNKTIECACRDNSYTAKRPFVKLNVETDTAEIRLMCTETYLDTPRPDDGSITGPCESNGDKGSGGIKGGFVHQRMASKIGRWYSRTMSKTKRMGMGLYVKYDGDPWTDSDALALSGVMVSMEEPGWYSFGFEIKDKKCDVPCIGIEMVHDGGKKTWHSAATAIYCLMGSGQLLWDTVTGVDMAL |
| B/Malaysia/10941/1996 | 1996 | LPSTIQTLTLFLTSGGVLLSLYVSASLSYLLYSDILLKFSPTEITAPTIPLDCANASNVQAVNRSATKGVTLLLPEPEWTYPRLSCPGSTFQKALLISPHRFGEIKGNSAPLIIREPFIACGPKECKHFALTHYAAQPGGYYNGTREDRNKLRHLISVKLGKIPTVENSIFHMAAWSGSACHDGKEWTYIGVDGPDSNALLKIKYGEAYTDTYHSYANNILRTQESACNCIGGNCYLMITDGSASGISECRFLKIREGRIIKEIFPTGRVEHTEECTCGFASNKTIECACRDNSYTAKRPFVKLNVETDTAEIRLMCTETYLDTPRPDDGSITGPCESNGDKGSGGIKGGFVHQRMASKIGRWYSRTMSKTKRMGMGLYVKYDGDPWTDSDALALSGVMVSMEEPGWYSFGFEIKDKKCDIPCIGIEMVHDGGKETWHSAATAIYCLMGSGQLLWDTVTGVDMAL |
| B/Malaysia/11087/1996 | 1996 | LPSTIQTLTLFLTSGGVLLSLYVSASLSYLLYSDILLKFSPTEITVPTMPLDCANASNVQAVNRSATKGVTLLLPEPEWTYPRLSCPGSTFQKALLISPHRFGETKGNSAPLIIREPFIACGPKECKHFALTHYAAQPGGYYNGTREDRNKLRHLISVKLGKIPTVENSIFHMAAWSGSACHDGREWTYIGVDGPDSNALLKIKYGEAYTDTYHSYANNILRTQESACNCIGGNCYLMITDGSASGISECRFLKIREGRIIKEIFPTGRVEHTEECTCGFASNKTIECACRDNSYTAKRPFVKLNVETDTAEIRLMCTETYLDTPRPDDGSITGPCESNGDKGSGGIKGGFVHQRMASKIGRWYSRTMSKTKRMGMGLYVKYDGDPWTDSDALALSGVMVSMEEPGWYSFGFEIKDKKCDVPCIGIEMVHDGGKKTWHSAATAIYCLMGSGQLLWDTVTGVDMAL |
| B/Malaysia/11169/1996 | 1996 | LPSTIQTLTLFLTSGGVLLSLYVSASLSYLLYSGILLKFSPTEITAPTMPLNCANASNFQAVNRSATKGVTLLLPEPEWTYPRLSCPGSTFQKALLISPHRFGETKGNSAPLIIREPFIACGPKECKHFALTHYAAQPGGYYNGTREDRNKLRHLISVKLGKIPTVENSIFHMAAWSGSACHDGREWTYIGVDGPDSNALLKIKYGEAYTDTYHSYANNILRTQESACNCIGGDCYLMITDGSASGISECRFLKIREGRIIKEIFPTGRVEHTEECTCGFASNKTIECACRDNSYTAKRPFVKLNVETDTAEIRLMCTETYLDTPRPDDGSITGPCESNGDKGSGGIKGGFVHQRMASKIGRWYSRTMSKTKRMGMGLYVKYDGDPWIDSDALTLSGVMVSMEEPGWYSFGFEIKDKKCDVPCIGIEMVHDGGKKTWHSAATAIYCLMGSGQLLWDTVTGVDMAL |
| B/Malaysia/11185/1996 | 1996 | LPSTIQTLTLFLTSGGVLLSLYVSASLSYLLYSDILLKFSPTEITAPTMPLDCANASNVQAVNRSATKGVTLLLPEPEWTYPRLSCPGSTFQKALLISPHRFGETKGNSAPLIIREPFIACGPKECKHFALTHYAAQPGGYYNGTREDRNKLRHLISVKLGKIPTVENSIFHMAAWSGSACHDGREWTYIGVDGPDSNALLKIKYGEAYTDTYHSYANNILRTQESACNCIGGNCYLMITDGSASGISECRFLKIREGRIIKEIFPTGRVEHTEECTCGFASNKTIECACRDNSYTAKRPFVKLNVETDTAEIRLMCTETYLDTPRPDDGSITGPCESNGDKGSGGIKGGFVHQRMASKIGRWYSRTMSKTKRMGMGLYVKYDGDPWTDSDALALSGVMVSMEEPGWYSFGFEIKDKKCDVPCIGIEMVHDGGKKTWHSAATAIYCLMGSGQLLWDTVTGVDMAL |
| B/Memphis/20/96 | 1996 | LPSTIQTLTLFLTSGGVLLSLYVSALLSYLLYSDVLLKFSP-KIIAPTMSLDCANASNVQAVNHSATEEMTFLLPEPEWTYPRLSCQGSTFQKALLISPHRFGEAKGNSAPLIIREPFIACGPKECKHFALTHYAAQPGGYYNGTREDRNKLRHLISVNLGKIPTVENSIFHMAAWSGSACHDGREWTYIGVDGPDSNALIKIKYGEAYTDTYHSYANNILRTQESACNCIGGDCYLMITDGPASGISKCRFLKIREGRIIKEIFPTGRVEHTEECTCGFASNKTIECACRDNSYTAKRPFVKLNVETDTAEIRLMCTETYLDTPRPDDGSITGPCESNGDKGHGGIKGGFVHQRMASKIGRWYSRTMSKTKRMGMELYVKYDGDPWIDSDALAPSGVMVSMEEPGWYSFGFEIKDKKCDVPCIGIEMVHDGGKKTWHSAATAIYCLMGSGQLLWDTVTGVDMAL |
| B/Memphis/21/96 | 1996 | LPSTIQTLTLFLTSGGVLLSLYVSASLSYLLYSDILLKFSPTEITAPTMPLDCANASNVQAVNRSATKGVTLFLPEPEWTYPRLSCPGSTFQKALLISPHRFGETKGNSAPLIIREPFIACGPKECKHFALTHYAAQPGGYYNGTREDRNKLRHLISVKLGKIPTVENSIFHMAAWSGSACHDGREWTYIGVDGPDSNALLKIKYGEAYTDTYHSYANNILRTQESACNCIGGNCYLMITDGSASGISECRFLKIREGRIIKEIFPTGRVEHTEECTCGFASNKTIECACRDNSYTAKRPFVKLNVETDTAEIRLMCTETYLDTPRPDDGSITGPCESNGDKGSGGIKGGFVHQRMASKIGRWYSRTMSKTKRMGMGLYVKYDGDPWTDSDALALSGVMVSMEEPGWYSFGFEIKDKKCDVPCIGIEMVHDGGKKTWHSAATAIYCLMGSGQLLWDTVTGVDMAL |
| B/Memphis/28/96 | 1996 | LPSTIQTLTLFLTSGGVLLSLYVSASLSYLLYSDILLKFSPTEITAPTMPLDCANASNVQAVNRSATKGVTLFLPEPEWTYPRLSCPGSTFQKALLISPHRFGETKGNSAPLIIREPFIACGPKECKHFALTHYAAQPGGYYNGTREDRNKLRHLISVKLGKIPTVENSIFHMAAWSGSACHDGREWTYIGVDGPDSNALLKIKYGEAYTDTYHSYANNILRTQESACNCIGGDCYLMITDGSASGISECRFLKIREGRIIKEIFPTGRVEHTEECTCGFASNKTIECACRDNSYTAKRPFVKLNVETDTAEIRLMCTETYLDTPRPDDGSITGPCESNGDKGSGGIKGGFVHQRMASKIGRWYSRTMSKTKRMGMGLYVKYDGDPWTDSDALALSGVMVSMEEPGWYSFGFEIKDKKCDVPCIGIEMVHDGGKKTWHSAATAIYCLMGSGQLLWDTVTGVDMAL |
| B/Nanchang/20/96 | 1996 | LPSTIQTLTLFLTSGGVLLSLYVSASLSYLLYSDILLKLSPTEITAPTVPLDCANASNFQAVNRSATKGVTLLLPEPEWTYPRLSCPGSTFQKALLISPHRFGETKGNSAPLIIREPFIACGPKECKHFALTHYAAQPGGYYNGTREDRNKLRHLISVKLGKIPTVENSIFHMAAWSGSACHDGREWTYIGVDGPDSNALLKIKYGEAYTDTYHSYANNILRTQESACNCIGGDCYLMITDGSASGISECRFLKIREGRIIKEIFPTGRVKHTEECTCGFASNKTIECACRDNSYTAKRPFVKLNVETDTAEIRLMCTETYLDTPRPDDGSITGPCESNGDKGSGGIKGGFVHQRMASKIGRWYSRTMSKTKRMGMGLYVKYDGDPWTDSDALALSGVMVSMEEPGWYSFGFEIKDKKCDVPCIGIEMVHDGGKKTWHSAATAIYCLMGSGQLLWDTATGVDMAL |
| B/Nanchang/24/1996 | 1996 | LPSTIQTLTLFLTSGGVLLSLYVSASLSYLLYSDILLKFSPTEITAPTMPLDCANASNVQAVNRSATKGVTLLLPEPEWTYPRLSCPGSTFQKALLISPHRFGETKGNSAPLIIREPFIACGPKECKHFALTHYAAQPGGYYNGTREDRNKLRHLISVKLGKIPTVENSIFHMAAWSGSACHDGREWTYIGVDGPDSNALLKIKYGEAYTDTYHSYANNILRTQESACNCIGGDCYLMITDGSASGISECRFLKIREGRIIKEIFPTGRVKHTEECTCGFASNKTIECACRDNSYTAKRPFVKLNVETDTAEIRLMCTETYLDTPRPDDGSITGPCESNGDKGSGGIKGGFVHQRMASKIGRWYSRTMSKTKRMGMGLYVKYDGDPWTDSDALALSGVMVSMEEPGWYSFGFEIKDKKCDVPCIGIEMVHDGGKKTWHSAATAIYCLMGSGQLLWDTVTGVDMAL |
| B/Nanchang/6/96 | 1996 | LPSTIQTLTLFLTSGGVLLSLYVSALLSYSLYSDILLKFSPTKTIAPTMSLDCANASNVQAVNHSATKGMTLLLPEPEWTYPRLSCQGSTFQKALLISPHRFGETKGNSAPLIIREPFIACGPKECKHFALTHYAAQPGGYYNGTREDRNKLRHLISVSLGKIPTVENSIFHMAAWSGSACHDGREWTYIGVDGPDSNALIKIKYGEAYTDTYHSYANNILRTQESACNCIGGDCYLMITDGSASGISKCRFLKIREGRIIKEIFPTGRVDHTEECTCGFASNKTIECACRDNSYTAKRPFVKLNVETDTAEIRLMCTETYLDTPRPDDGSTTGPCESNGDKGRGGIKGGFVHQRMASKIGRWYSRTMSKTERLGMELYVKYDGDPWTDSDALAPSGVMVSAEEPGWYSFGFEIKDKKCDVPCIGIEMVHDGGKKTWHSAATAIYCLMGSGQLLWDTVTGVDMAL |
| B/Nashville/3/96 | 1996 | LPSTIQTLTLFLTSGGVLLSLYVSASLAYLLYSDILLKFSPTEITAPTMPLDCANASNVQAVNRSATKGVTLLLPEPEWTYPRLSCPGSTFQKALLISPHRFGETKGNSAPLIIREPFIACGPKECKHFALTHYAAQPGGYYNGTREDRNKLRHLISVKLGKIPTVENSIFHMAAWSGSACHDGREWTYIGVDGPDSNALLKIKYGEAYTDTYHSYANNILRTQESACNCIGGDCYLMITDGSASGISECRFLKIREGRIIKEIFPTGRVEHTEECTCGFASNKTIECACRDNSYTAKRPFVKLNVETDTAEIRLMCTETYLDTPRPDDGSITGPCESNGDKGSGGIKGGFVHQRMASKIGRWYSRTMSKTKRMGMGLYVKYDGDPWTDSDALALSGVMVSMEEPGWYSFGFEIKDKKCDVPCIGIEMVHDGGKKTWHSAATAIYCLMGSGQLLWDTVTGVDMAL |
| B/Nashville/34/96 | 1996 | LPSTIQTLTLFLTSGGVLLSLYVSASLSYLLYSDILLKFSPTEITAPTMPLDCANASNVQAVNRSATKGVTLLLPEPEWTYPRLSCPGSTFQKALLISPHRFGETKGNSAPLIIREPFIACGPKECKHFALTHYAAQPGGYYNGTREDRNKLRHLISVKLGKIPTVENSIFHMAAWSGSACHDGREWTYIGVDGPDSNALLKIKYGEAYTDTYHSYANNILRTQESACNCIGGDCYLMITDGSASGISECRFLKIREGRIIKEIFPTGRVEHTEECTCGFASNKTIECACRDNSYTAKRPFVKLNVETDTAEIRLMCTETYLDTPRPDDGSITGPCESNGDKGSGGIKGGFVHQRMASKIGRWYSRTMSKTKRMGMGLYVKYDGDPWTDSDALALSGVMVSMEEPGWYSFGFEIKDKKCDVPCIGIEMVHDGGKETWHSAATAIYCLMGSGQLLWDTVTGVDMAL |
| B/Netherlands/6/96 | 1996 | LPSTIQTLTLFLTSGGVLLSLYVSASLSYLLYSGILLKFSPTEITAPTMPLDCANASNVQAVNRSATKGVTLLLPEPEWTYPRLSCPGSTFQKALLISPHRFGETRGNSAPLIIREPFIACGPKECKHFALTHYAAQPGGYYNGTREDRNKLRHLISVKLGKIPTVENSIFHMAAWSGSACHDGREWTYIGVDGPDSNALLKIKYGEAYTDTYHSYANNILRTQESACNCIGGDCYLMITDGSASGISKCRFLKIREGRIIKEIFPTGRVEHTEECTCGFASNKTIECACRDNSYTAKRPFVKLNVETDTAEIRLMCTETYLDTPRPDDGSITGPCESNGDKGSGGIKGGFVHQRMASKIGRWYSRTMSKTKRMGMGLYVKYDGDPWTDSGALALSGVMVSMEEPGWYSFGFEIKDKKCDVPCIGIEMVHDGGKKTWHSAATAIYCLMGSGQLLWDTVTGVDMAL |
| B/New York/1014/1996 | 1996 | LPSTIQTLTLFLTSGGVLLSLYVSASLSYLLYSDVLLKFSPTEITAPTMPLDCANASNVQAVNRSATKGVTLLLPEPEWTYPRLSCPGSTFQKALLISPHRFGETKGNSAPLIIREPFIACGPKECKHFALTHYAAQPGGYYNGTREDRNKLRHLISVKLGKIPTVENSIFHMAAWSGSACHDGREWTYIGVDGPDSNALLKIKYGEAYTDTYHSYANNILRTQESACNCIGGDCYLMITDGSASGISECRFLKIREGRIVKEIFPTGRVEHTEECTCGFASNKTIECACRDNSYTAKRPFVKLNVETDTAEIRLMCTETYLDTPRPEDGSITGPCESNGDKGSGGIKGGFVHQRMASKIGRWYSRTMSKTKRMGMGLYVKYDGDPWTDSDALALSGVMVSMEEPGWYSFGFEIKDKKCDVPCIGIEMVHDGGKETWHSAATAIYCLMGSGQLLWDTVTGVDMAL |
| B/New York/1015/1996 | 1996 | LPSTIQTLTLFLTSGGVLLSLYVSASLSYLLYSDVLLKFSPTEITAPTMPLDCANASNVQAVNRSATKGVTLLLPEPEWTYPRLSCPGSTFQKALLISPHRFGETKGNSAPLIIREPFIACGPKECKHFALTHYAAQPGGYYNGTREDRNKLRHLISVKLGKIPTVENSIFHMAAWSGSACHDGREWTYIGVDGPDSNALLKIKYGEAYTDTYHSYANNILRTQESACNCIGGDCYLMITDGSASGISECRFLKIREGRIVKEIFPTGRVEHTEECTCGFASNKTIECACRDNSYTAKRPFVKLNVETDTAEIRLMCTETYLDTPRPEDGSITGPCESNGDKGSGGIKGGFVHQRMASKIGRWYSRTMSKTKRMGMGLYVKYDGDPWTDSDALALSGVMVSMEEPGWYSFGFEIKDKKCDVPCIGIEMVHDGGKETWHSAATAIYCLMGSGQLLWDTVTGVDMAL |
| B/New York/1016/1996 | 1996 | LPSTIQTLTLFLTSGGVLLSLYVSASLSYLLYSDVLLKFSPTEITAPTMPLDCANASNVQAVNRSATKGVTLLLPEPEWTYPRLSCPGSTFQKALLISPHRFGETKGNSAPLIIREPFIACGPKECKHFALTHYAAQPGGYYNGTREDRNKLRHLISVKLGKIPTVENSIFHMAAWSGSACHDGREWTYIGVDGPDSNALLKIKYGEAYTDTYHSYANNILRTQESACNCIGGDCYLMITDGSASGISECRFLKIREGRIVKEIFPTGRVEHTEECTCGFASNKTIECACRDNSYTAKRPFVKLNVETDTAEIRLMCTETYLDTPRPEDGSITGPCESNGDKGSGGIKGGFVHQRMASKIGRWYSRTMSKTKRMGMGLYVKYDGDPWTDSDALALSGVMVSMEEPGWYSFGFEIKDKKCDVPCIGIEMVHDGGKETWHSAATAIYCLMGSGQLLWDTVTGVDMAL |
| B/shenfang/102/1996 | 1996 | LPSTIQTLTLFLTSGGVLLSLYVSASLSYLLYSGILLKFSPTEITAPTMPLDCANASNVQAVNRSATKGVTLLLPEPEWTYPRLSCPGSTFQKALLISPHRFGETKGNSAPLIIREPFIACGPKECKHFALTHYAAQPGGYYNGTREDRNKLRHLISVKLGKIPTVENSIFHMAAWSGSACHDGREWTYIGVDGPDSNALLKIKYGEAYTDTYHSYANNILRTQESACNCIGGDCYLMITDGSASGISECRFLKIREGRIIKEIFPTGRVEHTEECTCGFASNKTIECACRDNSYTAKRPFVKLNVETDTAEIRLMCTETYLDTPRPDDGSITGPCESNGDKGSGGIKGGFVHQRMASKIGRWYSRTMSKTKRMGMGLYVKYDGDPWTDSDALALSGVMVSMEEPGWYSFGFEIKDKKCDVPCIGIEMVHDGGKKTWHSAATAIYCLMGSGQLLWDTVTGVDMAL |
| B/Sichuan/281/96 | 1996 | LPSTIQTLTLFLTSGGVLLSLYVSALLSYLLYSDILLKFSPTKVIAPTMSLDCANASNVQAVNHSATEEMTFLLPEPEWTYPRLSCQGSTFQKALLISPHRFGEAKGNSAPLIIREPFIACGPKECIHFALTHYAAQPGGYYNGTREDRNKLRHLISVNLGKIPTVENSIFHMAAWSGSACHDGREWTYIGVDGPDSNALIKIKYGEAYTDTYHSYANNILRTQESACNCIGGDCYLMITDGSASGISRCRFLKIREGRIIKEIFPTGRVEHTEECTCGFASNKTIECVCRDNSYTAKRPFVKLNVETDTAEIRLMCTETYLDTPRPDDGSITGPCESNGDKGNGGIKGGFVHQRMASRIGRWYSRTMSKTKRMGMELYVKYDGDPWTDSDALAPSGVMVSMEEPGWYSFGFEIKDKKCDVPCIGIEMVHDGGKKTWHSAATAIYCLMGSGQLLWDTVTGVDMAL |
| B/Sichuan/53/1996 | 1996 | LPSTIQTLTLFLTSGGVLLSLYVSALLSYLLYSDILLKFSPTKVIAPTMSLDCANASNVQAVNHSATEEMTFLLPEPEWTYPRLSCQGSTFQKALLISPHRFGEAKGNSAPLIIREPFIACGPKECKHFALTHYAAQPGGYYNGTREDRNKLRHLISVNLGKIPTVENSIFHMAAWSGSACHDGREWTYIGVDGPDSNALIKIKYGEAYTDTYHSYANNILRTQESACNCIGGDCYLMITDGSASGISRCRFLKIREGRIIKEIFPTGRXEHTEECTCGFASNKTIECACRDNSYTAKRPFVKLNVETDTAEIRLMCTETYLDTPRPDDGSITGPCESNGDKGNGGIKGGFVHQRMASRIGRWYSRTMSKTKRMGMELYVKYDGDPWTDSDALAPSGVMVSMEEPGWYSFGFEIKDKKCDVPCIGIEMVHDGGKKTWHSAATAIYCLMGSGQLLWDTVTGVDMAL |
| B/Texas/19/96 | 1996 | LPSTIQTLTLFLTSGGVLLSLYVSASLSYLLYSDVLLKFSPTEITAPTMPLDCANASNVQAVNRSATKGVTLLLPEPEWTYPRLSCPGSTFQKALLISPHRFGETKGNSAPLIIREPFIACGPKECKHFALTHYAAQPGGYYNGTREDRNKLRHLISVKLGKIPTVENSIFHMAAWSGSACHDGREWTYIGVDGPDSNALLKIKYGEAYTDTYHSYANNILRTQESACNCIGGDCYLMITDGSASGISECRFLKIREGRIVKEIFPTGRVEHTEECTCGFASNKTIECACRDNSYTAKRPFVKLNVETDTAEIRLMCTETYLDTPRPEDGSITGPCESNGDKGSGGIKGGFVHQRMASKIGRWYSRTMSKTKRMGMGLYVKYDGDPWTDSDALALSGVMVSMEEPGWYSFGFEIKDKKCDVPCIGIEMVHDGGKETWHSAATAIYCLMGSGQLLWDTVTGVDMAL |
| B/Argentina/275/1997 | 1997 | LPSTIQTLTLFLTSGGVLLSLYVSASLSYLLYSDILLKFSPTEITAPTMPLNCANASNVQAVNRSATKGVTLLLPEPEWTYPRLSCPGSTFQKALLISPHRFGETKGNSAPLIIREPFIACGPKECKHFALTHYAAQPGGYYNGTREDRNKLRHLISVKLGKIPTVENSIFHMAAWSGSACHDGREWTYIGVDGPDSNALLKIKYGEAYTDTYHSYANNILRTQESACNCIGGDCYLMITDGSASGISECRFLKIREGRIIKEIFPTGRVEHTEECTCGFASNKTIECACRDNSYTAKRPFVKLNVETDTAEIRLMCTETYLDTPRPDDGSITGPCESNGDKGSGGIKGGFVHQRMASKIGRWYSRTMSKTKRMGMGLYVKYDGDPWIDSDALTLSGVMVSMEEPGWYSFGFEIKDKKCDVPCIGIEMVHDGGKKTWHSAATAIYCLMGSGQLLWDTVTGVDMAL |
| B/Beijing/243/97 | 1997 | LPSTIQTLTLFLTSGGVLLSLYVSALLSYLLYSDILLKFSPTKVIAPTMSLDCANASNVQAVNHSATEEMTFLLPEPEWTYPRLSCQGSTFQKALLISPHRFGEAKGNSAPLIIREPFIACGPKECKHFALTHYAAQPGGYYNGTREDRNKLRHLISVNLGKIPTVENSIFHMAAWSGSACHDGREWTYIGVDGPDSNALIKIKYGEAYTDTYHSYANNILRTQESACNCIGGDCYLMITDGSASGISRCRFLKIREGRIIKEIFPTGRVEHTEECTCGFASNKTIECACRDNSYTAKRPFVKLNVETDTAEIRLMCTETYLDTPRPDDGSITGPCESNGDKGNGGIKGGFVHQRMASRIGRWYSRTMSKTKRMGMELYVKYDGDPWTDSDALAPSGVMVSMEEPGWYSFGFEIKDKKCDVPCIGIEMVHDGGKKTWHSAATAIYCLMGSGQLLWDTVTGVDMAL |
| B/Guangzhou/7/97 | 1997 | LPSTIQTLTLFLTSGGVLLSLYVSALLSYLLYSDILLKFSPTKVIAPTMSLDCANASNVQAVNHSATEEMTFLLPEPEWTYPRLSCQGSTFQKALLISPHRFGEAKGNSAPLIIREPFIACGPKECKHFALTHYAAQPGGYYNGTREDRNKLRHLISVNLGKIPTVENSIFHMAAWSGSACHDGREWTYIGVDGPDSNALIKIKYGEAYTDTYHSYANNILRTQESACNCIGGDCYLMITDGSASGISRCRFLKIREGRIIKEIFPTGRVEHTEECTCGFASNKTIECACRDNSYTAKRPFVKLNVETDTAEIRLMCTETYLDTPRPDDGSITGPCESNGDKGNGGIKGGFVHQRMASRIGRWYSRTMSKTKRMGMELYVKYDGDPWTDSDALAPSGVMVSMEEPGWYSFGFEIKDKKCDVPCIGIEMVHDGGKKTWHSAATAIYCLMGSGQLLWDTVTGVDMAL |
| B/Henan/22/97 | 1997 | LPSTIQTLTLFLTSGGVLLSLYVSALLSYLLYSDILLKFSPTKVIAPTMSLDCANASNVQAVNHSATEEMTFLLPEPGWTYPRLSCQGSTFQKALLISPHRFGEAKGNSAPLIIREPFIACGPKECKHFALTHYAAQPGGYYNGTREDRNKLRHLISVNLGKIPTVENSIFHMAAWSGSACHDGREWTYIGVDGPDSNALIKIKYGEAYTDTYHSYANNILRTQESACNCIGGDCYLMITDGSASGISRCRFLKIREGRIIKEIFPTGRVEHTEECTCGFASNKTIECACRDNSYTAKRPFVKLNVETDTAEIRLMCTETYLDTPRPDDGSITGPCESNGDKGNGGIKGGFVHQRMASRIGRWYSRTMSKTKRMGMELYVKYDGDPWTDSDALAPSGVMVSMEEPGWYSFGFEIKDKKCDVPCIGIEMVHDGGKKTWHSAATAIYCLMGSGQLLWDTVTGVDMAL |
| B/Houston/B56/1997 | 1997 | LPSTIQTLTLFLTSGGVLLSLYVSASLSYLLYSDILLKFSPTEITAPTMPLNCANASNVQAVNRSATKGVTLPLPEPEWTYPRLSCPGSTFQKALLISPHRFGETKGNSAPLIIREPFIACGPKECKHFALTHYAAQPGGYYNGTREDRNKLRHLISVKLGKIPTVENSIFHMAAWSGSACHDGREWTYIGVDGPDSNALLKIKYGEAYTDTYHSYANNILRTQESACNCIGGDCYLMITDGSASGISECRFLKIREGRIIKEIFPTGRVEHTEECTCGFASNKTIECACRDNSYTAKRPFVKLNVETDTAEIRLMCTETYLDTPRPDDGSITGPCESNGDKGSGGIKGGFVHQRMASKIGRWYSRTMSKTKRMGMGLYVKYDGDPWIDSDALTLSGVMVSMEEPGWYSFGFEIKDKKCDVPCIGIEMVHDGGKKTWHSAATAIYCLMGSGQLLWDTVTGVDMAL |
| B/Houston/B57/1997 | 1997 | LPSTIQTLTLFLTSGGVLLSLYVSASLSYLLYSDILLKFSPTEITAPTMPLNCANASNVQAVNRSATKGVTLLLPEPEWTYPRLSCPGSTFQKALLISPHRFGETKGNSAPLIIREPFIACGPKECKHFALTHYAAQPGGYYNGTREDRNKLRHLISVKLGKIPTVENSIFHMAAWSGSACHDGREWTYIGVDGPDSNALLKIKYGEAYTDTYHSYANNILRTQESACNCIGGDCYLMITDGSASGISECRFLKIREGRIIKEIFPTGRVEHTEECTCGFASNKTIECACRDNSYTAKRPFVKLNVETDTAEIRLMCTETYLDTPRPDDGSITGPCESNGDKGSGGIKGGFVHQRMASKIGRWYSRTMSKTKRMGMGLYVKYDGDPWIDSDALTLSGVMVSMEEPGWYSFGFEIKDKKCDVPCIGIEMVHDGGKKTWHSAATAIYCLMGSGQLLWDTVTGVDMDL |
| B/Houston/B58/1997 | 1997 | LPSTIQTLTLFLTSGGVLLSLYVSASLSYLLYSDILLKFSPTEITAPTMPLNCANASNVQAVNRSATKGVTLLLPEPEWTYPRLSCPGSTFQKALLISPHRFGETKGNSAPLIIREPFIACGPKECKHFALTHYAAQPGGYYNGTREDRNKLRHLISVKLGKIPTVENSIFHMAAWSGSACHDGREWTYIGVDGPDSNALLKIKYGEAYTDTYHSYANNILRTQESACNCIGGDCYLMITDGSASGISECRFLKIREGRIIKEIFPTGRVEHTEECTCGFASNKTIECACRDNSYTAKRPFVKLNVETDTAEIRLMCTETYLDTPRPDDGSITGPCESNGDKGSGGIKGGFVHQRMASKIGRWYSRTMSKTKRMGMGLYVKYDGDPWIDSDALTLSGVMVSMEEPGWYSFGFEIKDKKCDVPCIGIEMVHDGGKKTWHSAATAIYCLMGSGQLLWDTVTGVDMDL |
| B/Houston/B59/1997 | 1997 | LPSTIQTLTLFLTSGGVLLSLYVSASLSYLLYSDILLKFSPTEITAPTMPLNCANASNVQAVNRSATKGVTLPLPEPEWTYPRLSCPGSTFQKALLISPHRFGETKGNSAPLIIREPFIACGPKECKHFALTHYAAQPGGYYNGTREDRNKLRHLISVKLGKIPTVENSIFHMAAWSGSACHDGREWTYIGVDGPDSNALLKIKYGEAYTDTYHSYANNILRTQESACNCIGGDCYLMITDGSASGISECRFLKIREGRIIKEIFPTGRVEHTEECTCGFASNKTIECACRDNSYTAKRPFVKLNVETDTAEIRLMCTETYLDTPRPDDGSITGPCESNGDKGSGGIKGGFVHQRMASKIGRWYSRTMSKTKRMGMGLYVKYDGDPWIDSDALTLSGVMVSMEEPGWYSFGFEIKDKKCDVPCIGIEMVHDGGKKTWHSAATAIYCLMGSGQLLWDTVTGVDMAL |
| B/Houston/B60/1997 | 1997 | LPSTIQTLTLFLTSGGVLLSLYVSASLSYLLYSDILLKFSPTEITAPTMPLNCANASNVQAVNRSATKGVTLLLPEPEWTYPRLSCPGSTFQKALLISPHRFGETKGNSAPLIIREPFIACGPKECKHFALTHYAAQPGGYYNGTREDRNKLRHLISVKLGKIPTVENSIFHMAAWSGSACHDGREWTYIGVDGPDSNALLKIKYGEAYTDTYHSYANNILRTQESACNCIGGDCYLMITDGSASGISECRFLKIREGRIIKEIFPTGRVEHTEECTCGFASNKTIECACRDNSYTAKRPFVKLNVETDTAEIRLMCTETYLDTPRPDDGSITGPCESNGDKGSGGIKGGFVHQRMASKIGRWYSRTMSKTKRMGMGLYVKYDGDPWIDSDALTLSGVMVSMEEPGWYSFGFEIKDKKCDVPCIGIEMVHDGGKKTWHSAATAIYCLMGSGQLLWDTVTGVDMAL |
| B/Houston/B61/1997 | 1997 | LPSTIQTLTLFLTSGGVLLSLYVSASLSYLLYSDILLKFSPTEITAPTMPLNCANASNVQAVNRSATKGVTLLLPEPEWTYPRLSCPGSTFQKALLISPHRFGETKGNSAPLIIREPFIACGPKECKHFALTHYAAQPGGYYNGTREDRNKLRHLISVKLGKIPTVENSIFHMAAWSGSACHDGREWTYIGVDGPDSNALLKIKYGEAYTDTYHSYANNILRTQESACNCIGGDCYLMITDGSASGISECRFLKIREGRIIKEIFPTGRVEHTEECTCGFASNKTIECACRDNSYTAKRPFVKLNVETDTAEIRLMCTETYLDTPRPDDGSITGPCESNGDKGSGGIKGGFVHQRMASKIGRWYSRTMSKTKRMGMGLYVKYDGDPWIDSDALTLSGVMVSMEEPGWYSFGFEIKDKKCDVPCIGIEMVHDGGKKTWHSAATAIYCLMGSGQLLWDTVTGVDMAL |
| B/Houston/B63/1997 | 1997 | LPSTIQTLTLFLTSGGVLLSLYVSASLSYLLYSDILLKFSPTEITAPTMPLNCANASNVQAVNRSATKGVTLLLPEPEWTYPRLSCPGSTFQKALLISPHRFGETKGNSAPLIIREPFIACGPKECKHFALTHYAAQPGGYYNGTREDRNKLRHLISVKLGKIPTVENSIFHMAAWSGSACHDGREWTYIGVDGPDSNALLKIKYGEAYTDTYHSYANNILRTQESACNCIGGDCYLMITDGSASGISECRFLKIREGRIIKEIFPTGRVEHTEECTCGFASNKTIECACRDNSYTAKRPFVKLNVETDTAEIRLMCTETYLDTPRPDDGSITGPCESNGDKGSGGIKGGFVHQRMASKIGRWYSRTMSKTKRMGMGLYVKYDGDPWIDSDALTLSGVMVSMEEPGWYSFGFEIKDKKCDVPCIGIEMVHDGGKKTWHSAATAIYCLMGSGQLLWDTVTGVDMAL |
| B/Malaysia/11721/1997 | 1997 | LPSTIQTLTLFLTSGGVLLSLYVSASLSYLLYSDILLKFSPTEITAPTMPLDCANASNVQAVNRSATKGVTLLLPEPEWTYPRLSCPGSTFQKALLISPHRFGETKGNSAPLIIREPFIACGPKECKHFALTHYAAQPGGYYNGTREDRNKLRHLISVKLGKIPTVENSIFHMAAWSGSACHDGREWTYIGVDGPDSNALLKIKYGEAYTDTYHSYANNILRTQESACNCIGGDCYLMITDGSASGISECRFLKIREGRIIKEIFPTGRVEHTEECTCGFASNKTIECACRDNSYTAKRPFVKLNVETDTAEIRLMCTETYLDTPRPDDGSITGPCESNGDKGSGGIKGGFVHQRMTSKIGRWYSRTMSKTKRMGMGLYVKYDGDPWTDSDALALSGVMVSMEEPGWYSFGFEIKDKKCDVPCIGIEMVHDGGKKTWHSAATAIYCLMGSGQLLWDTVTGVDMAL |
| B/Malaysia/13367/1997 | 1997 | LPSTIQTLTLFLTSGGVLLSLYVSASLSYLLYSGILLKFSLTEITAPTMPLNCANASNVQAVNRSATKGVTLFLPEPEWTYPRLSCPGSTFQKALLISPHRFGETKGNSAPLIIREPFIACGPKECKHFALTHYAAQPGGYYNGTREDRNKLRHLISVELGKIPTVENSIFHMAAWSGSACHDGKEWTYIGVDGPDSNALLKIKYGEAYTDTYHSYANNILRTQESACNCIGGDCYLMITDGSASGISECRFLKIREGRIIKEIFPTGRVEHTEECTCGFASNKTIECACRDNSYTAKRPFVKLNVETDTAEIRLMCTETYLDTPRPDDGSITGPCESNGDKGSGGIKGGFVHQRMASKIGRWYSRTMSKTKRMGMGLYVKYDGDPWIDSDALTLSGVMVSMEEPGWYSFGFEIKDKKCDVPCIGIEMVHDGGKKTWHSAATAIYCLMGSGQLLWDTVTGVDMAL |
| B/Malaysia/13395/1997 | 1997 | LPSTIQTLTLFLTSGGVLLSLYVSASLSYLLYSGILLKFSLTEITAPTMPLNCANASNVQAVNRSATKGVTLFLPEPEWTYPRLSCPGSTFQKALLISPHRFGETKGNSAPLIIREPFIACGPKECKHFALTHYAAQPGGYYNGTREDRNKLRHLISVELGKIPTVENSIFHMAAWSGSACHDGKEWTYIGVDGPDSNALLKIKYGEAYTDTYHSYANNILRTQESACNCIGGDCYLMITDGSASGISECRFLKIREGRIIKEIFPTGRVEHTEECTCGFASNKTIECACRDNSYTAKRPFVKLNVETDTAEIRLMCTETYLDTPRPDDGSITGPCESNGDKGSGGIKGGFVHQRMASKIGRWYSRTMSKTKRMGMGLYVKYDGDPWIDSDALTLSGVMVSMEEPGWYSFGFEIKDKKCDVPCIGIEMVHDGGKKTWHSAATAIYCLMGSGQLLWDTVTGVDMAL |
| B/Malaysia/13396/1997 | 1997 | LPSTIQTLTLFLTSGGVLLSLYVSASLSYLLYSGILLKFSLTEITAPTMPLNCANASNVQAVNRSATKGVTLFLPEPEWTYPRLSCPGSTFQKALLISPHRFGETKGNSAPLIIREPFIACGPKECKHFALTHYAAQPGGYYNGTREDRNKLRHLISVELGKIPTVENSIFHMAAWSGSACHDGKEWTYIGVDGPDSNALLKIKYGEAYTDTYHSYANNILRTQESACNCIGGDCYLMITDGSASGISECRFLKIREGRIIKEIFPTGRVEHTEECTCGFASNKTIECACRDNSYTAKRPFVKLNVETDTAEIRLMCTETYLDTPRPDDGSITGPCESNGDKGSGGIKGGFVHQRMASKIGRWYSRTMSKTKRMGMGLYVKYDGDPWIDSDALTLSGVMVSMEEPGWYSFGFEIKDKKCDVPCIGIEMVHDGGKKTWHSAATAIYCLMGSGQLLWDTVTGVDMAL |
| B/Malaysia/13494/1997 | 1997 | LPSTIQTLTLFLTSGGVLLSLYVSASLSYLLYSGILLKFSPTEITAPTMPLNCANASNVQAVNRSATKGVTLLLPEPEWTYPRLSCPGSTFQKALLISPHRFGETKGNSAPLIIREPFIACGPKECKHFALTHYAAQPGGYYNGTREDRNKLRHLISVKLGKIPTVENSIFHMAAWSGSACHDGREWTYIGVDGPDSNALLKIKYGEAYTDTYHSYANNILRTQESACNCIGGDCYLMITDGSASGISECRFLKIREGRIIKEIFPTGRVEHTEECTCGFASNKTIECACRDNSYTAKRPFVKLNVETDTAEIRLMCTETYLDTPRPDDGSITGPCESHGDKGSGGIKGGFVHQRMASKIGRWYSRTMSKTKRMGMGLYVKYDGDPWIDSGALTLSGVMVSMEEPGWYSFGFEIKDKKCDVPCIGIEMVHDGGKKTWHSAATAIYCLMGSGQLLWDTVTGVDMAL |
| B/Malaysia/13693/1997 | 1997 | LPSTIQTLTLFLTSGGVLLSLYVSASLSYLLYSDILLKFSPTEITAPTMPLDCANASNVQAVNRSATKGVTLLLPEPEWTYPRLSCPGSTFQKALLISPHRFGETKGNSAPLIIREPFIACGPKECKHFALTHYAAQPGGYYNGTREDRNKLRHLISVKLGKIPTVENSIFHMAAWSGSACHDGKEWTYIGVDGPDSNALLKIKYGEAYTDTYHSYANNILRTQESACNCIGGNCYLMITDGSASGISECRFLKIREGRIIKEIFPTGRVKHTEECTCGFASNKTIECACRDNSYTAKRPFVKLNVETDTAEIRLMCTETYLDTPRPDDGSITGPCESNGDKGSGGIKGGFVHQRMASKIGRWYSRTMSKTKRMGMGLYVKYDGDPWTDSDALALSGVMVSMEEPGWYSFGFEIKDKKCDVPCIGIEMVHDGGKETWHSAATAIYCLMGSGQLLWDTVTGVDMAL |
| B/Malaysia/13696/1997 | 1997 | LPSTIQTLTLFLTSGGVLLSLYVSASLSYLLYSGILLKFSLTEITAPTMPLNCANASNVQAVNRSATKGVTLFLPEPEWTYPRLSCPGSTFQKALLISPHRFGETKGNSAPLIIREPFIACGPKECKHFALTHYAAQPGGYYNGTREDRNKLRHLISVELGKIPTVENSIFHMAAWSGSACHDGKEWTYIGVDGPDSNALLKIKYGEAYTDTYHSYANNILRTQESACNCIGGDCYLMITDGSASGISECRFLKIREGRIIKEIFPTGRVEHTEECTCGFASNKTIECACRDNSYTAKRPFVKLNVETDTAEIRLMCTETYLDTPRPDDGSITGPCESNGDKGSGGIKGGFVHQRMASKIGRWYSRTMSKTKRMGMGLYVKYDGDPWIDSDALTLSGVMVSMEEPGWYSFGFEIKDKKCDVPCIGIEMVHDGGKKTWHSAATAIYCLMGSGQLLWDTVTGVDMAL |
| B/Nanchang/15/97 | 1997 | LPSTIQTLTLFLTSGGVLLSLYVSALLSYLLYSDILLKFSPTKVIAPTMSLDCANASNVQAVNHSATEEMTFLLPEPEWTYPRLSCQGSTFQKALLISPHRFGEAKGNSAPLIIREPFIACGPKECKHFALTHYAAQPGGYYNGTREDRNKLRHLISVNLGKIPTVENSIFHMAAWSGSACHDGREWTYIGVDGPDSNALIKIKYGEAYTDTYHSYANNILRTQESACNCIGGDCYLMITDGSASGISRCRFLKIREGRIIKEIFPTGRVEHTEECTCGFASNKTIECACRDNSYTAKRPFVKLNVETDTAEIRLMCTETYLDTPRPDDGSITGPCESNGDKGNGGIKGGFVHQRMASRIGRWYSRTMSKTKRMGMELYVKYDGDPWTDSDALAPSGVMVSMEEPGWYSFGFEIKDKKCDVPCIGIEMVHDGGKKTWHSAATAIYCLMGSGQLLWDTVTGVNMAL |
| B/Nanchang/2/97 | 1997 | LPSTIQTLTLFLTSGGVLLSLYVSALLSYLLYSDILLKFSPTKVIAPTMSLDCANASNVQAVNHSATEEMTFLLPEPEWTYPRLSCQGSTFQKALLISPHRFGETKGNSAPLIIREPFIACGPKECKHFALTHYAAQPGGYYNGTREDRNKLRHLISVNLGKIPTVENSIFHMAAWSGSACHDGREWTYIGVDGPDSNALIKIKYGEAYTDTYHSYANNILRTQESACNCIGGDCYLMITDGSASGISRCRFLKIREGRIIKEIFPTGRVEHTEECTCGFASNKTIECACRDNSYTAKRPFVKLNVETDTAEIRLMCTETYLDTPRPDDGSITGPCESNGDKGNGGIKGGFVHQRMASRIGRWYSRTMSKTKRMGMELYVKYDGDPWTDSDALAPSGVMVSMEEPGWYSFGFEIKDKKCDVPCIGIEMVHDGGKKTWHSAATAIYCLMGSGQLLWDTVTGVDMAL |
| B/Nanchang/4/97 | 1997 | LPSTIQTLTLFLTSGGVLLSLYVSALLSYLLYSDILLKFSPTKVIAPTMSLDCANASNVQAVNHSATEEMTFLLPEPEWTYPRLSCQGSTFQKALLISPHRFGETKGNSAPLIIREPFIACGPKECKHFALTHYAAQPGGYYNGTREDRNKLRHLISVNLGKIPTVENSIFHMAAWSGSACHDGREWTYIGVDGPDSNALIKIKYGEAYTDTYHSYANNILRTQESACNCIGGDCYLMITDGSASGISRCRFLKIREGRIIKEIFPTGRVEHTEECTCGFASNKTIECACRDNSYTAKRPFVKLNVETDTAEIRLMCTETYLDTPRPDDGSITGPCESNGDKGNGGIKGGFVHQRMASRIGRWYSRTMSKTKRMGMELYVKYDGDPWTDSDALAPSGVMVSMEEPGWYSFGFEIKDKKCDVPCIGIEMVHDGGKKTWHSAATAIYCLMGSGQLLWDTVTGVDMAL |
| B/Nanchang/5/97 | 1997 | LPSTIQTLTLFLTSGGVLLSLYVSALLSYLLYSDILLKFSPTKVIAPTMSLDCANASNVQAVNHSATEEMTFLLPEPEWTYPRLSCQGSTFQKALLISPHRFGETKGNSAPLIIREPFIACGPKECKHFALTHYAAQPGGYYNGTREDRNKLRHLISVNLGKIPTVENSIFHMAAWSGSACHDGREWTYIGVDGPDSNALIKIKYGEAYTDTYHSYANNILRTQESACNCIGGDCYLMITDGSASGISRCRFLKIREGRIIKEIFPTGRVEHTEECTCGFASNKTIECACRDNSYTAQRPFVKLNVETDTAEIRLMCTETYLDTPRPDDGSITGPCESNGDKGNGGIKGGFVHQRMASRIGRWYSRTMSKTKRMGMELYVKYDGDPWTDSDALAPSGVMVSMEEPGWYSFGFEIKDKKCDVPCIGIEMVHDGGKKTWHSAATAIYCLMGSGQLLWDTVTGVDMAL |
| B/New York/1017/1997 | 1997 | LPSTIQTLTLFLTSGGVLLSLYVSASLSYLLYSDILLKFSPTEITAPTMPLNCANASNVQAVNRSATKGVTLPLPEPEWTYPRLSCPGSTFQKALLISPHRFGETKGNSAPLIIREPFIACGPKECKHFALTHYAAQPGGYYNGTREDRNKLRHLISVKLGKIPTVENSIFHMAAWSGSACHDGREWTYIGVDGPDSNALLKIKYGEAYTDTYHSYANNILRTQESACNCIGGDCYLMITDGSASGISECRFLKIREGRIIKEIFPTGRVEHTEECTCGFASNKTIECACRDNSYTAKRPFVKLNVETDTAEIRLMCTETYLDTPRPDDGSITGPCESNGDKGSGGIKGGFVHQRMASKIGRWYSRTMSKTKRMGMGLYVKYDGDPWIDSDALTLSGVMVSMEEPGWYSFGFEIKDKKCDVPCIGIEMVHDGGKKTWHSAATAIYCLMGSGQLLWDTVTGVDMAL |
| B/New York/1018/1997 | 1997 | LPSTIQTLTLFLTSGGVLLSLYVSASLSYLLYSDILLKFSPTEITAPTMPLNCANASNVQAVNRSATKGVTLPLPEPEWTYPRLSCPGSTFQKALLISPHRFGETKGNSAPLIIREPFIACGPKECKHFALTHYAAQPGGYYNGTREDRNKLRHLISVKLGKIPTVENSIFHMAAWSGSACHDGREWTYIGVDGPDSNALLKIKYGEAYTDTYHSYANNILRTQESACNCIGGDCYLMITDGSASGISECRFLKIREGRIIKEIFPTGRVEHTEECTCGFASNKTIECACRDNSYTAKRPFVKLNVETDTAEIRLMCTETYLDTPRPDDGSITGPCESNGDKGSGGIKGGFVHQRMASKIGRWYSRTMSKTKRMGMGLYVKYDGDPWIDSDALTLSGVMVSMEEPGWYSFGFEIKDKKCDVPCIGIEMVHDGGKKTWHSAATAIYCLMGSGQLLWDTVTGVDMAL |
| B/Osaka/547/1997 | 1997 | LPSTIQTLTLFLTSGGVLLSLYVSALLSYLLYSDILLKFSPTKVIAPTMSLDCANASNVQAVNHSATEEMTFLLPEPEWTYPRLSCQGSTFQKALLISPHRFGEAKGNSAPLIIREPFIACGPKECKHFALTHYAAQPGGYYNGTREDRNKLRHLISVNLGKIPTVENSIFHMAAWSGSACHDGREWTYIGVDGPDSNALIKIKYGEAYTDTYHSYANNILRTQESACNCIGGDCYLMITDGSASGISRCRFLKIREGRIIKEIFPTGRVEHTEECTCGFASNKTIECACRDNSYTAKRPFVKLNVETDTAEIRLMCTETYLDTPRPDDGSITGPCESNGDKGNGGIKGGFVHQRMASRIGRWYSRTMSKTKRMGMELYVKYDGDPWTDSDALAPSGVMVSMEEPGWYSFGFEIKDKKCDVPCIGIEMVHDGGKKTWHSAATAIYCLMGSGQLLWDTVTGVDMAL |
| B/Osaka/547/97 | 1997 | LPSTIQTLTLFLTSGGVLLSLYVSALLSYLLYSDILLKFSPTKVIAPTMSLDCANASNVQAVNHSATEEMTFLLPEPEWTYPRLSCQGSTFQKALLISPHRFGEAKGNSAPLIIREPFIACGPKECKHFALTHYAAQPGGYYNGTREDRNKLRHLISVNLGKIPTVENSIFHMAAWSGSACHDGREWTYIGVDGPDSNALIKIKYGEAYTDTYHSYANNILRTQESACNCIGGDCYLMITDGSASGISRCRFLKIREGRIIKEIFPTGRVEHTEECTCGFASNKTIECACRDNSYTAKRPFVKLNVETDTAEIRLMCTETYLDTPRPDDGSITGPCESNGDKGNGGIKGGFVHQRMASRIGRWYSRTMSKTKRMGMELYVKYDGDPWTDSDALAPSGVMVSMEEPGWYSFGFEIKDKKCDVPCIGIEMVHDGGKKTWHSAATAIYCLMGSGQLLWDTVTGVDMAL |
| B/Shandong/7/97 | 1997 | LPSTIQTLTLFLTSGGVLLSLYVSALLSYLLYSDILLKFSPTKVIAPTMSLDCANASNVQAVNHSATEEMTFLLPEPEWTYPRLSCQGSTFQKALLISPHRFGEAKGNSAPLIIREPFIACGPKECKHFALTHYAAQPGGYYNGTREDRNKLRHLISVNLGKIPTVENSIFHMAAWSGSACHDGREWTYIGVDGPDSNALLKIKYGEAYTDTYHSYANNILRTQESACNCIGGDCYLMITDGSASGISRCRFLKIREGRIIKEIFPTGRVEHTEECTCGFASNKTIECACRDNSYTAKRPFVKLNVETDTAEIRLMCTETYLDTPRPDDGSITGPCESNGDKGNGGIKGGFVHQRMASRIGRWYSRTMSKTKRMGMELYVKYDGDPWTDSDALAPSGVMVSIEEPGWYSFGFEIKDKKCDVPCIGIEMVHDGGKKTWHSAATAIYCLMGSGQLLWDTVTGVDMAL |
| B/Switzerland/4291/97 | 1997 | LPSTIQTLTLFLTSGGVLLSLYVSALLSYLLYSDILLKFSPTEITAPTMPLDCANASNVQAVNRSATKGVTLLLPEPEWTYPRLSCPGSTFQKALLISPHRFGETKGNSAPLIIREPFIACGPKECKHFALTHYAAQPGGYYNGTREDRNKLRHLISVKLGKIPTVENSIFHMAAWSGSACHDGREWTYIGVDGPDSNALLKIKYGEAYTDTYHSYANNILRTQESACNCIGGNCYLMITDGSASGISECRFLKIREGRIIKEIFPTGRVEHTEECTCGFASNKTIECACRDNSYTAKRPFVKLNVETDTAEIRLMCTETYLDTPRPDDGSITGPCESNGDKGSGGIKGGFVHQRMASKIGRWYSRTMSKTKRMGMGLYVKYDGDPWTDSDALALSGVMVSMEEPGWYSFGFEIKDKKCDVPCIGIEMVHDGGKKTWHSAATAIYCLMGSGQLLWDTVTGVDMVL |
| B/Taiwan/217/97 | 1997 | LPSTIQTLILFLTSGGVLLSLYVSALLSYLLYSDILLKFSPTKIIAPTMSLDCANASNVQAVNHSATKEMTFLLPEPEWTYPRLSCQGSTFQKALLISPHRFGEAKGNSAPLIIREPFIACGPKECKHFALTHYAAQPGGYYNGTREDRNKLRHLISVNLGKIPTVENSIFHMAAWSGSACHDGREWTYIGVDGPDSNALIKIKYGEAYTDTYHSYANNILRTQESACNCIGGDCYLMITDGSASGISKCRFLKIREGRIIKEIFPTGRVEHTEECTCGFASNKTIECACRDNSYTAKRPFVKLNVETDTAEIRLMCTKTYLDTPRPDDGSITGPCESNGDKGSGGIKGGFVHQRMASKIGRWYSRTMSKTKRMGMELYVKYDGDPWTDSDALAPSGVMVSIEEPGWYSFGFEIKDKKCDVPCIGIEMVHDGGKKTWHSAATAIYCLMGSGQLLWDTVTGVDMAL |
| B/Beijing/76/98 | 1998 | LPSTIQTLTLFLTSGGVLLSLYVSASLSYLLYSDILLKFSQTEITAPTMPLDCANASNVQAVNRSATKGVTLLLPEPEWTYPRLSCPGSTFQKALLISPHRFGETKGNSAPLIIREPFIACGPKECKHFALTHYAAQPGGYYNGTREDRNKLRHLISVKLGKIPTVENSIFHMAAWSGSACHDGREWTYIGVDGPDNNALLKIKYGEAYTDTYHSYANNILRTQESACNCIGGDCYLMITDGSASGISECRFLKIREGRIIKEIFPTGRVKHTEECTCGFASNKTIECACRDNSYTAKRPFVKLNVETDTAEIRLMCTETYLDTPRPDDGSITGPCESNGDKGSGGIKGGFVHQRMASKIGRWYSRTMSKTKRMGMGLYVKYDGDPWTDSDALALSGVMVSMEEPGWYSFGFEIKDKKCDVPCIGIEMVHDGGKKTWHSAATAIYCLMGSGQLLWDTVTGVDMAL |
| B/Bucharest/311/1998 | 1998 | LPSTIQTLTLFLTSGGVLLSLYVSASLSYLLYSDILLKFSPTEITAPTMPLDCANASNVQAVNRSATKGVTLLLPEPEWTYPRLSCPGSTFQKALLISPHRFGETKGNSAPLIIREPFIACGPKECKHFALTHYAAQPGGYYNGTREDRNKLRHLISVKLGKIPTVENSIFHMAAWSGSACHDGKEWTYIGVDGPDSNALLKIKYGEAYTDTYHSYANNILRTQESACNCIGGNCYLMITDGSASGISECRFLKIREGRIIKEIFPTGRVEHTEECTCGFASNKTIECACRDNSYTAKRPFVKLNVETDTAEIRLMCTETYLDTPRPDDGSITGPCESNGDKGSGGIKGGFVHQRMASKIGRWYSRTMSKTKRMGMGLYVKYDGDPWTDSDALALSGVMVSMEEPGWYSFGFEIKDKKCDVPCIGIEMVHDGGKETWHSAATAIYCLMGSGQLLWDTVTGVDMAL |
| B/Chiba/447/98 | 1998 | LPSTIQTLTLFLTSGGVLLSLYASALLSYLLYSDILLKFSPTKVIAPTMSLDCANASNVQAVNHSATEEMTFPLPEPEWTYPRLSCQGSTFQKALLISPHRFGEAKGNSAPLIIREPFIACGPKECKHFALTHYAAQPGGYYNGTREDRNKLRHLISVNLGKIPTVENSIFHMAAWSGSACHDGREWTYIGVDGPDSNALIKIKYGEAYTDTYHSYANNILRTQESACNCIGGDCYLMITDGSASGISRCRFLKIREGRIIKEIFPTGRVEHTEECTCGFASNKTIECACRDNSYTAKRPFVKLNVETDTAEIRLMCTETYLDTPRPDDGSITGPCESNGDKGNGGIKGGFVHQRMASRIGRWYSRTMSKTKRMGMELYVKYDGDPWTDSDALAPSGVMVSMEEPGWYSFGFEIKDKKCDVPCIGIEMVHDGGKKTWHSAATAIYCLMGSGQLLWDTVTGVNMAL |
| B/Florida/02/1998 | 1998 | LPSTIQTLTLFLTSGGVLLSLYVSASLSYLLYSDILLKFSPTEITAPTMPLNCANASNVQAVNRSATKGVTLPLPEPEWTYPRLSCPGSTFQKALLISPHRFGETKGNSAPLIIREPFIACGPKECKHFALTHYAAQPGGYYNGTREDRNKLRHLISVKLGKIPTVENSIFHMAAWSGSACHDGREWTYIGVDGPDSNALLKIKYGEAYTDTYHSYANNILRTQESACNCIGGDCYLMITDGSASGISECRFLKIREGRIIKEIFPTGRVEHTEECTCGFASNKTIECACRDNSYTAKRPFVKLNVETDTAEIRLMCTETYLDTPRPDDGSITGPCESNGDKGSGGIKGGFVHQRMASKIGRWYSRTMSKTKRMGMGLYVKYDGDPWIDSDALTLSGVMVSMEEPGWYSFGFEIKDKKCDVPCIGIEMVHDGGKTTWHSAATAIYCLMGSGQLLWDTVTGVDMAL |
| B/Georgia/02/1998 | 1998 | LPSTIQTLTLFLTSGGVLLSLYVSASLSYLLYSDILLKFSPTEITAPTMPLNCANASNVQAVNRSATKGVTLPLPEPEWTYPRLSCPGSTFQKALLISPHRFGETKGNSAPLIIREPFIACGPKECKHFALTHYAAQPGGYYNGTREDRNKLRHLISVKLGKIPTVENSIFHMAAWSGSACHDGREWTYIGVDGPDSNALLKIKYGEAYTDTYHSYANNILRTQESACNCIGGDCYLMITDGSASGISECRFLKIREGRIIKEIFPTGRVEHTEECTCGFASNKTIECACRDNSYTAKRPFVKLNVETDTAEIRLMCTETYLDTPRPDDGSITGPCESNGDKGSGGIKGGFVHQRMASKIGRWYSRTMSKTKRMGMGLYVKYDGDPWIDSDALTLSGVMVSMEEPGWYSFGFEIKDKKCDVPCIGIEMVHDGGKTTWHSAATAIYCLMGSGQLLWDTVTGVDMAL |
| B/Georgia/04/1998 | 1998 | LPSTIQTLTLFLTSGGVLLSLYVSASLSYLLYSDILLKFSPTEITAPTMPLNCANASNVQAVNRSATKGVTLPLPEPEWTYPRLSCPGSTFQKALLISPHRFGETKGNSAPLIIREPFIACGPKECKHFALTHYAAQPGGYYNGTREDRNKLRHLISVKLGKIPTVENSIFHMAAWSGSACHDGREWTYIGVDGPDSNALLKIKYGEAYTDTYHSYANNILRTQESACNCIGGDCYLMITDGSASGISECRFLKIREGRIIKEIFPTGRVEHTEECTCGFASNKTIECACRDNSYTAKRPFVKLNVETDTAEIRLMCTETYLDTPRPDDGSITGPCESNGDKGSGGIKGGFVHQRMASKIGRWYSRTMSKTKRMGMGLYVKYDGDPWIDSDALTLSGVMVSMEEPGWYSFGFEIKDKKCDVPCIGIEMVHDGGKTTWHSAATAIYCLMGSGQLLWDTVTGVDMAL |
| B/Houston/B65/1998 | 1998 | LPSTIQTLTLFLTSGGVLLSLYVSASLSYLLYSDILLKFSPTEITAPTMPLNCANASNVQAVNRSATKGVTLPLPEPEWTYPRLSCPGSTFQKALLISPHRFGETKGNSAPLIIREPFIACGPKECKHFALTHYAAQPGGYYNGTREDRNKLRHLISVKLGKIPTVENSIFHMAAWSGSACHDGREWTYIGVDGPDSNALLKIKYGEAYTDTYHSYANNILRTQESACNCIGGDCYLMITDGSASGISECRFLKIREGRIIKEIFPTGRVEHTEECTCGFASNKTIECACRDNSYTAKRPFVKLNVETDTAEIRLMCTETYLDTPRPDDGSITGPCESNGDKGSGGIKGGFVHQRMASKIGRWYSRTMSKTKRMGMGLYVKYDGDPWIDSDALTLSGVMVSMEEPGWYSFGFEIKDKKCDVPCIGIEMVHDGGKTTWHSAATAIYCLMGSGQLLWDTVTGVDMAL |
| B/Malaysia/15048/1998 | 1998 | LPSTIQTLTLFLTSGGVLLSLYVSALLSYLLYSDILLKFSPTKVIAPTMSLDCANASNVQAVNHSATEEMTFLLPEPEWTYPRLSCQGSTFQKALLISPHRFGEAKGNSAPLIIREPFIACGPKECKHFALTHYAAQPGGYYNGTREDRNKLRHLISVNLGKIPTVENSIFHMAAWSGSACHDGREWTYIGVDGPDSNALIKIKYGEAYTDTYHSYANNILRTQESACNCIGGDCYLMITDGSASGISRCRFLKIREGRIIKEIFPTGRVEHTEECTCGFASNKTIECACRDNSYTAKRPFVKLNVETDTAEIRLMCTETYLDTPRPDDGSITGPCESNGDKGNGGIKGGFVHQRMASRIGRWYSRTMSKTKRMGMELYVKYDGDPWTDSDALAPSGVMVSMEEPGWYSFGFEIKDKKCDVPCIGIEMVHDGGKTTWHSAATAIYCLMGSGQLLWDTVTGVDMAL |
| B/Nanchang/12/98 | 1998 | LPSTIQTLTLFLTSGGVLLSLYVSASLSYLLYSDILLKFSQTEITAPTMPLDCANASNVQAVNRSATKGVTLLLPEPEWTYPRLSCPGSTFQKALLISPHRFGETKGNSAPLIIREPFIACGPKECKHFALTHYAAQPGGYYNGTREDRNKLRHLISVKLGKIPTVENSIFHMAAWSGSACHDGREWTYIGVDGPDSNALLKIKYGEAYTDTYHSYANNILRTQESACNCIGGDCYLMITDGSASGISECRFLKIREGRIIKEIFPTGRVKHTEECTCGFASNKTIECACRDNSYTAKRPFVKLNVETDTAEIRLMCTETYLDTPRPDDGSITGPCESNGDKGSGGIKGGFVHQRMASKIGRWYSRTMSKTKRMGMGLYVKYDGDPWTDSDALALSGVMVSMEEPGWYSFGFEIKDKKCDVPCIGIEMVHDGGKKTWHSAATAIYCLMGSGQLLWDTVTGVDMAL |
| B/Nanchang/6/98 | 1998 | LPSTIQTLTLFLTSGGVLLSLYVSALLSYLLYSDILLKFSPTKVIAPTMSLDCVNASNVQAVNHSATEEMTFLLPEPEWTYPRLSCQGSTFQKALLISPHRFGEAKGNSAPLIIREPFIACGPKECKHFALTHYAAQPGGYYNGTREDRNKLRHLISVNLGKIPTVENSIFHMAAWSGSACHDGREWTYIGVDGPDSNALIKIKYGEAYTDTYHSYANNILRTQESACNCIGGDCYLMITDGSASGISRCRFLKIREGRIIKEIFPTGRVEHTEECTCGFASNKTIECACRDNSYTAKRPFVKLNVETDTAEIRLMCTETYLDTPRPDDGSITGPCESNGDKGNGGIKGGFVHQRMASRIGRWYSRTMSKTKRMGMELYVKYDGDPWTDSDALAPSGVMVSMEEPGWYSFGFEIKDKKCDVPCIGIEMVHDGGKKTWHSAATAIYCLMGSGQLLWDTVTGVDMAL |
| B/Nanchang/7/98 | 1998 | LPSTIQTLTLFLTSGGVLLSLYVSASLSYLLYSDILLKFSPTEITAPTMPLDCANASNVQAVNRSATKGVTLFLPEPEWTYPRLSCPGSTFQKALLISPHRFGETKGNSAPLIIREPFIACGPKECKHFALTHYAAQPGGYYNGTREDRNKLRHLISVKLGKIPTVENSIFHMAAWSGSACHDGREWTYIGVDGPDSNALLKIKYGEAYTDTYHSYANNILRTQESACNCIGGNCYLMITDGSASGISECRFLKIREGRIIKEIFPTGRVEHTEECTCGFASNKTIECACRDNSYTAKRPFVKLNVETDTAEIRLMCTETYLDTPRPDDGSITGPCESNGDKGSGGIKGGFVHQRMASKIGRWYSRTMSKTKRMGMGLYVKYDGDPWTDSDALALSGVMVSMEEPGWYSFGFEIKDKKCDVPCIGIEMVHDGGKKTWHSAATAIYCLMGSGQLLWDTVTGVDMAL |
| B/Romania/318/1998 | 1998 | LPSTIQTLTLFLTSGGVLLSLYVSASLSYLLYSDILLKFSPTEITAPTMPLDCANASNVQAVNRSATKGVTLLLPEPEWTYPRLSCPGSTFQKALLISPHRFGETKGNSAPLIIREPFIACGPKECKHFALTHYAAQPGGYYNGTREDRNKLRHLISVKLGKIPTVENSIFHMAAWSGSACHDGKEWTYIGVDGPDSNALLKIKYGEAYTDTYHSYANNILRTQESACNCIGGNCYLMITDGSASGISECRFLKIREGRIIKEIFPTGRVEHTEECTCGFASNKTIECACRDNSYTAKRPFVKLNVETDTAEIRLMCTETYLDTPRPDDGSITGPCESNGDKGSGGIKGGFVHQRMASKIGRWYSRTMSKTKRMGMGLYVKYDGDPWTDSDALALSGVMVSMEEPGWYSFGFEIKDKKCDVPCIGIEMVHDGGKETWHSAATAIYCLMGSGQLLWDTVTGVDMAL |
| B/Shiga/51/98 | 1998 | LPSTIQTLTLFLTSGGVLLSLYVSALLSYLLYSDILLKFSPTKVIAPTMSLDCANASNVQAVNHSATEEMTFLLPEPEWTYPRLSCQGSTFQKALLISPHRFGEAKGNSAPLIIREPFIACGPKECKHFALTHYAAQPGGYYNGTREDRNKLRHLISVNLGKIPTVENSIFHMAAWSGSACHDGREWTYIGVDGPDSNALIKIKYGEAYTDTYHSYANNILRTQESACNCIGGDCYLMITDGSASGISRCRFLKIREGRIIKEIFPTGRVEHTEECTCGFASNKTIECACRDNSYTAKRPFVKLNVETDTAEIRLMCTETYLDTPRPDDGSITGPCESNGDKGNGGIKGGFVHQRMASRIGRWYSRTMSKTKRMGMELYVKYDGDPWTDSDALAPSGVMVSMEEPGWYSFGFEIKDKKCDVPCIGIEMVHDGGKKTWHSAATAIYCLMGSGQLLWDTVTGVDMAL |
| B/Shiga/T30/98 | 1998 | LPSTIQTLTLFLTSGGVLLSLYVSASLSYLLYSDILLKFSPTEITAPTMPLDCANASNVQAVNRSATKGVTLLLPEPEWTYPRLSCPGSTFQKALLISPHRFGETKGNSAPLIIREPFIACGPKECKHFALTHYAAQPGGYYNGTREDRNKLRHLISVKLGKIPTVENSIFHMAAWSGSACHDGKEWTYIGVDGPDSNALLKIKYGEAYTDTYHSYANNILRTQESACNCIGGNCYLMITDGSASGISECRFLKIREGRIIKEIFPTGRVEHTEECTCGFASNKTIECACRDNSYTAKRPFVKLNVETDTAEIRLMCTETYLDTPRPDDGSITGPCESNGDKGSGGIKGGFVHQRMASKIGRWYSRTMSKTKRMGMGLYVKYDGDPWTDSDALALSGVMVSMEEPGWYSFGFEIKDKKCDVPCIGIEMVHDGGKETWHSAATAIYCLMGSGQLLWDTVTGVDMAL |
| B/Singapore/22/1998 | 1998 | LPSTIQTLTLFLTSGGVLLSLYVSASLSYLLYSDILLKFSQTEITAPTMPLDCANASNVQAVNLSATKGVTLLLPEPEWTYPRLSCPGSTFQKALLISPHRFGETKGNSAPLIIREPFIACGPKECKHFALTHYAAQPGGYYNGTREDRNKLRHLISVKLGKIPTVENSIFHMAAWSGSACHDGREWTYIGVDGPDSNALLKIKYGEAYTDTYHSYANNILRTQESACNCIGGNCYLMITDGSASGISECRFLKIREGRIIKEIFPTGRVEHTEECTCGFASNKTIECACRDNSYTAKRPFVKLNVETDTAEIRLMCTETYLDTPRPDDGSITGPCESNGDKGSGGIKGGFVHQRMASKIGRWYSRTMSKTKRMGMGLYVKYDGDPWTDSDALALSGVMVSMEEPGWYSFGFEIKDKKCDVPCIGIEMVHDGGKRTWHSAATAIYCLMGSGQLLWDTVTGVDMAL |
| B/Singapore/31/1998 | 1998 | LPSTIQTLTLFLTSGGVLLSLYVSASLSYLLYSDILLKFSQTEITAPTMPLDCANASNVQAVNLSATKGVTLLLPEPEWTYPRLSCPGSTFQKALLISPHRFGETKGNSAPLIIREPFIACGPKECKHFALTHYAAQPGGYYNGTREDRNKLRHLISVKLGKIPTVENSIFHMAAWSGSACHDGREWTYIGVDGPDSNALLKIKYGEAYTDTYHSYANNILRTQESACNCIGGNCYLMITDGSASGISECRFLKIREGRIIKEIFPTGRVEHTEECTCGFASNKTIECACRDNSYTAKRPFVKLNVETDTAEIRLMCTETYLDTPRPDDGSITGPCESNGDKGSGGIKGGFVHQRMASKIGRWYSRTMSKTKRMGMGLYVKYDGDPWTDSDALALSGVMVSMEEPGWYSFGFEIKDKKCDVPCIGIEMVHDGGKRTWHSAATAIYCLMGSGQLLWDTVTGVDMAL |
| B/Singapore/35/1998 | 1998 | LPSTIQTLTLFLTSGGVLLSLYVSASLSYLLYSGILLKFSPTEITAPTMPLNCANASNVQAVNRSATKGVTLLLPEPEWTYPRLSCPGSTFQKALLISPHRFGETKGNSAPLIIREPFIACGPKECKHFALTHYAAQPGGYYNGTREDRNKLRHLISVKLGKIPTVENSIFHMAAWSGSACHDGREWTYIGVDGPDSNALLKIKYGEAYTDTYHSYANNILRTQESACNCIGGDCYLMITDGSASGISECRFLKIREGRIIKEIFPTGRVEHTEECTCGFASNKTIECACRDNSYTAKRPFVKLNVETDTAEIRLMCTETYLDTPRPDDGSITGPCESNGDKGSGGIKGGFVHQRMASKIGRWYSRTMSKTKRMGMGLYVKYDGDPWIDSDALTLSGVMVSMEEPGWYSFGFEIKDKKCDVPCIGIEMVHDGGKKTWHSAATAIYCLMGSGQLLWDTVTGVDMAL |
| B/Yamanashi/166/98 | 1998 | LPSTIQTLTLFLTSGGVLLSLYVSASLSYLLYSDILLKFSPTEITAPTMPLNCANASNVQAVNRSATKGVTLPLPEPEWTYPRLSCPGSTFQKALLISPHRFGETKGNSAPLIIREPFIACGPKECRHFALTHYAAQPGGYYNGTREDRNKLRHLISVKLGKIPTVENSIFHMAAWSGSACHDGREWTYIGVDGPDSNALLKIKYGEAYTDTYHSYANNILRTQESACNCIGGDCYLMITDGSASGISECRFLKIREGRIIKEIFPTGRVEHTEECTCGFASNKTIECACRDNSYTAKRPFVKLNVETDTAEIRLMCTETYLDTPRPDDGSITGPCESNGDKGSGGIKGGFVHQRMASKIGRWYSRTMSKTKRMGMGLYVKYDGDPWIDSDALTLSGVMVSMEEPGWYSFGFEIKDKKCDVPCIGIEMVHDGGKKTWHSAATAIYCLMGSGQLLWDTVTGVDMAL |
| B/Aichi/20/99 | 1999 | LPSTIQTLTLFLTSGGVLLSLYVSALLSYLLYSDILLKFSPTKVIAPTMSLDCANASNVQAVNHSATEEMTFLLPEPEWTYPRLSCQGSTFQKALLISPHRFGEAKGNSAPLIIREPFIACGPKECKHFALTHYAAQPGGYYNGTREDRNKMRHLISVNLGKIPTVENSIFHMAAWSGSACHDGREWTYIGVDGPDSNALIKIKYGEAYTDTYHSYANNILRTQESACNCIGGDCYLMITDGSASGISRCRFLKIREGRIIKEIFPTGRIEHTEECTCGFASNKTIECACRDNSYTAKRPFVKLNVETDTAEIRLMCTETYLDTPRPDDGSITGPCESNGDKGNGGIKGGFVHQRMASRIGRWYSRTMSKTKRMGMELYVKYDGDPWTDSDALAPSGVMVSMEEPGWYSFGFEIKDKKCDVPCIGIEMVHDGGKKTWHSAATAIYCLMGSGQLLWDTVTGVDMAL |
| B/Argentina/3640/1999 | 1999 | LPSTIQTLTLFLTSGGVLLSLYVSASLSYLLYSDILLKFSPTETTAPTMPLDCANASNVQAVNCSATKGVTLLLPEPEWTYPRLSCPGSTFQKALLISPHRFGETKGNSAPLIIREPFIACGPKECKHFALTHYAAQPGGYYNGTREDRNKLRHLISVKLGKIPTVENSIFHMAAWSGSACHDGKEWTYIGVDGPDSNALLKIKYGEAYTDTYHSYANNILRTQESACNCIGGNCYLMITDGSASGISECRFLKIREGRIIKEIFPTGRVEHTEECTCGFASNKTIECACRDNSYTAKRPFVKLNVETDTAEIRLMCTETYLDTPRPDDGSITGPCESNGDKGSGGIKGGFVHQRMASKIGRWYSRTMSKTKRMGMGLYVKYDGDPWTDSDALALSGVMVSMEEPGWYSFGFEIKDKKCDVPCIGIEMVHDGGKETWHSAATAIYCLMGSGQLLWDTVTGVDMAL |
| B/Bangkok/34/99 | 1999 | LPSTIQTLTLFLTSGGVLLSLYVSALLSYLLYSDILLKFSPTKVIAPTMSLDCANASNVQAVNHSATEEMTFLLPEPEWTYPRLSCQGSTFQKALLISPHRFGEAKGNSAPLIIREPFIACGPKECKHFALTHYAAQPGEYYNGTREDRNKLRHLISVNLGKIPTVENSIFHMAAWSGSACHDGREWTYIGVDGPDSNALIKIKYGEAYTDTYHSYANNILRTQESACNCIGGDCYLMITDGSASGISRCRFLKIREGRIIKEIFPTGRVEHTEECTCGFASNKTIECACRDNSYTAKRPFVKLNVETDTAEIRLMCTETYLDTPRPDDGSITGPCESNGDKGNGGIKGGFVHQRMASRIGRWYSRTMSKTKRMGMELYVKYDGDPWTDSDALAPSGVMVSMEEPGWYSFGFEIKDKKCDVPCIGIEMVHDGGKKTWHSAATAIYCLMGSGQLLWDTVTGVDMAL |
| B/Bangkok/54/99 | 1999 | LPSTIQTLTLFLTSGGVLLSLYVSALLSYLLYSDILLKFSPTKVIAPTMSLDCANASNVQAVNHSATEEMTFLLPEPEWTYPRLSCQGSTFQKALLISPHRFGEAKGNSAPLIIREPFIACGPKECKHFALTHYAAQPGGYYNGTREDRNKLRHLISVNLGKIPTVENSIFHMAAWSGSACHDGREWTYIGVDGPDSNALIKIKYGEAYTDTYHSYANNILRTQESACNCIGGDCYLMITDGSASGISRCRFLKIREGRILKEIFPTGRVEHTEECTCGFASNKTIECACRDNSYTAKRPFVQLNVETDTAEIRLMCTETYLDTPRPDDGSITGPCESNGDKGNGGIKGGFVHQRMASRIGRWYSRTMSKTKRMGMDLYVKYDGDPWTDSDALAPSGVMVSMEEPGWYSFGFEIKDKKCDVPCIGIEMVHDGGKKTWHSAATAIYCLMGSGQLLWDTVTGVDMAL |
| B/hanfang/383/1999 | 1999 | LPSTIQTLTLFLTSGGVLLSLYVSASLSYLLYSDILLKFSQTEITAPTMPLDCANASNVQAVNRSATKGVTLLLPEPEWTYPRLSCPGSTFQKALLISPHRFGETKGNSAPLIIREPFIACGPKECKHFALTHYAAQPGGYYNGTREDRNKLRHLISVKLGKIPTVENSIFHMAAWSGSACHDGREWTYIGVDGPDSNALLKIKYGEAYTDTYHSYANNILRTQESACNCIGGDCYLMITDGSASGISECRFLKIREGRIIKEIFPTGRVKHTEECTCGFASNKTIECACRDNSYTAKRPFVKLNVETDTAEIRLMCTETYLDTPRPDDGSITGPCESNGDKGSGGIKGGFVHQRMASKIGRWYSRTMSKTKRMGMGLYVKYDGDPWTDSEALALNGVMVSMEEPGWYSFGFEIKDKKCDVPCIGIEMVHDGGKKTWHSAATAIYCLMGSGQLLWDTVTGVDMAL |
| B/Houston/B15/1999 | 1999 | LPSTIQTLTLFLTSGGVLLSLYVSASLSYLLYSDILLKFSPKEITTPTMPLNCANASNVQAVNRSATKGVTLLLPEPEWTYPRLSCPGSTFQKALLISPHRFGETKGNSAPLIIREPFIACGPKECKHFALTHYAAQPGGYYNGTREDRNKLRHLISVKLGKIPTVENSIFHMAAWSGSACHDGREWTYIGVDGPDSNALLKIKYGEAYTDTYHSYANNILRTQESACNCIGGDCYLMITDGSASGISECRFLKIREGRIIKEIFPTGRVEHTEECTCGFASNKTIECACRDNSYTAKRPFVKLNVETDTAEIRLMCTETYLDTPRPDDGSITGPCESNGDKGSGGIKGGFVHQRMASKIGRWYSRTMSKTKRMGMGLYVKYDGDPWIDSDALTLSGVMVSMEEPGWYSFGFEIKDKKCDVPCIGIEMVHDGGKTTWHSAATAIYCLMGSGQLLWDTVTGVDMAL |
| B/Johannesburg/05/1999 | 1999 | LPSTIQTLTLFLTSGGVLLSLYVSASLSYLLYSDILLKFSPTEITAPAMPLDCANASNVQAVNRSATKGVTLLLPEPEWTYPRLSCPGSTFQKALLISPHRFGETKGNSAPLIIREPFIACGPKECKHFALTHYAAQPGGYYNGTREDRNKLRHLISVKLGKIPTVENSIFHMAAWSGSACHDGKEWTYIGVDGPDSNALLKIKYGEAYTDTYHSYANNILRTQESACNCIGGNCYLMITDGSASGISECRFLKIREGRIIKEIFPTGRVKHTEECTCGFASNKTIECACRDNSYTAKRPFVKLNVETDTAEIRLMCTETYLDTPRPDDGSITGPCESNGDKGSGGIKGGFVHQRMASKIGRWYSRTMSKTKRMGMGLYVKYDGDPWTDSDALALSGVMVSMEEPGWYSFGFEIKDKKCDVPCIGIEMVHDGGKETWHSAATAIYCLMGSGQLLWDTVTGVDMAL |
| B/Kouchi/193/99 | 1999 | LPSTIQTLTLFLTSGGVLLSLYVSASLSYLLYSDILLKFSQTEITAPTMPLDCANASNVQAVNLSATKGVTLLLPEPEWTYPRLSCPGSTFQKALLISPHRFGETKGNSAPLIIREPFIACGPKECKHFALTHYAAQPGGYYNGTREDRNKLRHLISVKLGKIPTVENSIFHMAAWSGSACHDGREWTYIGVDGPDSNALLKIKYGEAYTDTYHSYANNILRTQESACNCIGGNCYLMITDGSASGISECRFLKIREGRIIKEIFPTGRVEHTEECTCGFASNKTIECACRDNSYTAKRPFVKLNVETDTAEIRLMCTETYLDTPRPDDGSITGPCESNGDKGSGGIKGGFVHQRMASKIGRWYSRTMSKTKRMGMGLYVKYDGDPWTDSDALALSGVMVSMEEPGWYSFGFEIKDKKCDVPCIGIEMVHDGGKRTWHSAATAIYCLMGSGQLLWDTVTGVDMAL |
| B/Memphis/8/99 | 1999 | LPSTIQTLTLFLTSGGVLLSLYVSASLSYLLYSDILLKFSPTEITAPTMPLNCANASNVQAVNRSATKGVTLPLPEPEWTYPRLSCPGSTFQKALLISPHRFGETKGNSAPLIIREPFIACGPKECKHFALTHYAAQPGGYYNGTREDRNKLRHLISVKLGKIPTVENSIFHMAAWSGSACHDGREWTYIGVDGPDSNALLKIKYGEAYTDTYHSYANNILRTQESACNCIGGDCYLMITDGSASGISECRFLKIREGRIIKEIFPTGRVEHTEECTCGFASNKTIECACRDNSYTAKRPFVKLNVETDTAEIRLMCTETYLDTPRPDDGSITGPCESNGDEGSGGIKGGFVHQRMASKIGRWYSRTMSKTKRMGMGLYVKYDGDPWIDSDALTLSGVMVSMEEPGWYSFGFEIKDKKCDVPCIGIEMVHDGGKTTWHSAATAIYCLMGSGQLLWDTVTGVDMAL |
| B/New York/1019/1999 | 1999 | LPSTIQTLTLFLTSGGVLLSLYVSASLSYLLYSDILLKFSPKEITAPTMPLNCANASNVQAVNRSATKGVTLPLPEPEWTYPRLSCPGSTFQKALLISPHRFGETKGNSAPLIIREPFIACGPKECKHFALTHYAAQPGGYYNGTREDRNKLRHLISVKLGKIPTVENSIFHMAAWSGSACHDGREWTYIGVDGPDNNALLKIKYGEAYTDTYHSYANNILRTQESACNCIGGDCYLMITDGSASGISECRFLKIREGRIIKEIFPTGRVEHTEECTCGFASNKTIECACRDNSYTAKRPFVKLNVETDTAEIRLMCTETYLDTPRPDDGSITGPCESNGDKGSGGIKGGFVHQRMASKIGRWYSRTMSKTKRMGMGLYVKYDGDPWIDSDALTLSGVMVSMEEPGWYSFGFEIKDKKCDVPCIGIEMVHDGGKTTWHSAATAIYCLMGSGQLLWDTVTGVDMAL |
| B/New York/1020/1999 | 1999 | LPSTIQTLTLFLTSGGVLLSLYVSASLSYLLYSDILLKFTPTEITAPTMPLDCANASNVQAVNLSATKGVTLLLPEPEWTYPRLSCPGSTFQKALLISPHRFGETKGNSAPLIIREPFIACGPKECKHFALTHYAAQPGGYYNGTREDRNKLRHLISVKLGKIPTVENSIFHMAAWSGSACHDGKEWTYIGVDGPDSNALLKIKYGEAYTDTYHSYANNILRTQESACNCIGGNCYLMITDGSASGISECRFLKIREGRIIKEIFPTGRVKHTEECTCGFASNKTIECACRDNSYTAKRPFVKLNVETDTAEIRLMCTETYLDTPRPDDGSITGPCESNGNKGSGGIKGGFVHQRMASKIGRWYSRTMSKTKRMGMGLYVKYDGDPWTDSDALALSGVMVSMEEPGWYSFGFEIKDKKCDVPCIGIEMVHDGGKETWHSAATAIYCLMGSGQLLWDTVTGVDMAL |
| B/New York/1021/1999 | 1999 | LPSTIQTLTLFLTSGGVLLSLYVSASLSYLLYSDILLKFTPTEITAPTMPLDCANASNVQAVNLSATKGVTLLLPEPEWTYPRLSCPGSTFQKALLISPHRFGETKGNSAPLIIREPFIACGPKECKHFALTHYAAQPGGYYNGTREDRNKLRHLISVKLGKIPTVENSIFHMAAWSGSACHDGKEWTYIGVDGPDSNALLKIKYGEAYTDTYHSYANNILRTQESACNCIGGNCYLMITDGSASGISECRFLKIREGRIIKEIFPTGRVKHTEECTCGFASNKTIECACRDNSYTAKRPFVKLNVETDTAEIRLMCTETYLDTPRPDDGSITGPCESNGNKGSGGIKGGFVHQRMASKIGRWYSRTMSKTKRMGMGLYVKYDGDPWTDSDALALSGVMVSMEEPGWYSFGFEIKDKKCDVPCIGIEMVHDGGKETWHSAATAIYCLMGSGQLLWDTVTGVDMAL |
| B/New York/347/1999 | 1999 | LPSTIQTLTLFLTSGGVLLSLYVSASLSYLLYSDILLKFSPTEITAPTMPLDCANASNVQAVNRSATKGVTLLLPEPEWTYPRLSCPGSTFQKALLISPHRFGETKGNSAPLIIREPFIACGPKECKHFALTHYAAQPGGYYNGTREDRNKLRHLISVKLGKIPTVENSIFHMAAWSGSACHDGKEWTYIGVDGPDSNALLKIKYGEAYTDTYHSYANNILRTQESACNCIGGNCYLMITDGSASGISECRFLKIREGRIIKEIFPTGRVEHTEECTCGFASNKTIECACRDNSYTAKRPFVKLNVETDTAEIRLMCTETYLDTPRPDDGSITGPCESNGDKGSGGIKGGFVHQRMASKIGRWYSRTMSKTKRMGMGLYVKYDGDPWTDSDALALSGVMVSMEEPGWYSFGFEIKDKKCDVPCIGIEMVHDGGKETWHSAATAIYCLMGSGQLLWDTVTGVDMAL |
| B/Paris/549/1999 | 1999 | LPSTIQTLTLFLTSGGVLLSLYVSASLSYLLYSDILLKFSPTEITAPTMPLDCANASNVQAVNRSATKGVTLLLPEPEWTYPRLSCPGSTFQKALLISPHRFGETKGNSAPLIIREPFIACGPKECKHFALTHYAAQPGGYYNGTREDRNKLRHLISVKLGKIPTVENSIFHMAAWSGSACHDGKEWTYIGVDGPDSNALLKIKYGEAYTDTYHSYANNILRTQESACNCIGGNCYLMITDGSASGISECRFLKIREGRIIKEIFPTGRVEHTEECTCGFASNKTIECACRDNSYTAKRPFVKLNVETDTAEIRLMCTETYLDTPRPDDGSITGPCESNGDKGSGGIKGGFVHQRMASKIGRWYSRTMSKTKRMGMGLYVKYDGDPWTDSDALALSGVMVSMEEPGWYSFGFEIKDKKCDVPCIGIEMVHDGGKETWHSAATAIYCLMGSGQLLWDTVTGVDMAL |
| B/Romania/217/1999 | 1999 | LPSTIQTLTLFLTSGGVLLSLYVSASLSYLLYSDILLKFSPTEITAPTMPLDCANASNVQAVNRSATKGVTLLLPEPEWTYPRLSCPGSTFQKALLISPHRFGETKGNSAPLIIREPFIACGPKECKHFALTHYAAQPGGYYNGTREDRNKLRHLISVKLGKIPTVENSIFHMAAWSGSACHDGKEWTYIGVDGPDSNALLKIKYGEAYTDTYHSYANNILRTQESACNCIGGNCYLMITDGSASGISECRFLKIREGRIIKEIFPTGRVEHTEECTCGFASNKTIECACRDNSYTAKRPFVKLNVETDTAEIRLMCTETYLDTPRPDDGSITGPCESNGDKGSGGIKGGFVHQRMASKIGRWYSRTMSKTKRMGMGLYVKYDGDPWTDSDALALSGVMVSMEEPGWYSFGFEIKDKKCDVPCIGIEMVHDGGKETWHSAATAIYCLMGSGQLLWDTVTGVNMAL |
| B/Saga/S172/99 | 1999 | LPSTIQTLTLFLTSGGVLLSLYVSASLSYLLYSGILLKFSPTEITAPTMPLNCANASNVQAVNRSATKGVTLLLPEPEWTYPRLSCPGSTFQKALLISPHRFGETKGNSAPLIIREPFIACGPKECKHFALTHYAAQPGGYYNGTREDRNKLRHLISVKLGKIPTVENSIFHMAAWSGSACHDGREWTYIGVDGPDSNALLKIKYGEAYTDTYHSYANNILRTQESACNCIGGDCYLMITDGSASGISECRFLKIREGRIIKEIFPTGRVEHTEECTCGFASNKTIECACRDNSYTAKRPFVKLNVETDTAEIRLMCTETYLDTPRPDDGSITGPCESNGDKGSGGIKGGFVHQRMASKIGRWYSRTMSKTKRMGMGLYVKYDGDPWIDSDALTLSGVMVSMEEPGWYSFGFEIKDKKCDVPCIGIEMVHDGGKKTWHSAATAIYCLMGSGQLLWDTVTGVDMAL |
| B/Sichuan/207/1999 | 1999 | LPSTIQTLTLFLTSGGVLLSLYVSALLSYLLYSDILLKFSPTKVIAPTMSLDCANASNVQAVNHSATEEMTFLLPEPEWTYPRLSCQGSTFQKALLISPHRFGEAKGNSAPLIIREPFIACGPKECKHFALTHYAAQPGGYYNGTREDRNKLRHLISVNLGKIPTVENSIFHMAAWSGSACHDGREWTYIGVDGPDSNALIKIKYGEAYTDTYHSYANNILRTQESACNCIGGDCYLMITDGSASGISRCRFLKIREGRIIKEIFPTGRVEHTEECTCGFASNKTIECACRDNSYTAKRPFVKLNVETDTAEIRLMCTETYLDTPRPDDGSITGPCESNGDKGNGGIKGGFVHQRMASRIGRWYSRTMSKTKRMGMELYVKYDGDPWTDSDALAPSGVMVSMEEPGWYSFGFEIKDKKCDVPCIGIEMVHDGGKKTWHSAATAIYCLMGSGQLLWDTVTGVDMAL |
| B/Sichuan/379/99 | 1999 | LPSTIQTLTLFLTSGGVLLSLYVSASLSYLLYSDILLKFSPTEITAPTMPLDCANASNVQAVNRSATKGVTFLLPEPEWTYPRLSCPGSTFQKALLISPHRFGETKGNSAPLIIREPFIACGPKECKHFALTHYAAQPGGYYNGTREDRNKLRHLISVKLGKIPTVENSIFHMAAWSGSACHDGKEWTYIGVDGPDSNALLKIKYGEAYTDTYHSYANNILRTQESACNCIGGNCYLMITDGSASGISECRFLKIREGRIIKEIFPTGRVKHTEECTCGFASNKTIECACRDNSYTAKRPFVKLNVETDTAEIRLMCTETYLDTPRPDDGSITGPCESNGDKGSGGIKGGFVHQRMASKIGRWYSRTMSKTKRMGMGLYVKYDGDPWTDSDALALSGVMVSMEEPGWYSFGFEIKDKKCDVPCIGIEMVHDGGKETWHSAATAIYCLMGSGQLLWDTVTGVDMAL |
| B/South Australia/5/1999 | 1999 | LPSTIQTLTLFLTSGGVLLSLYVSASLSYLLYSDILLKFSPTEITAPTMPLDCANASNVQAVNRSATKGVALLLPEPEWTYPRLSCPGSTFQKALLISPHRFGETKGNSAPLIIREPFIACGPKECKHFALTHYAAQPGGYYNGTREDRNKLRHLISVKLGKIPTVENSIFHMAAWSGSACNDGKEWTYIGVDGPDSNALLKIKYGEAYTDTYHSYANNILRTQESACNCIGGNCYLMITDGSASGISECRFLKIREGRIIKEIFPTGRVKHTEECTCGFASNKTIECACRDNSYTAKRPFVKLNVETDTAEIRLMCTETYLDTPRPDDGSITGPCESNGDKGSGGIKGGFVHQRMASKIGRWYSRTMSKTKRMGMGLYVKYDGDPWTDSDALALSGVMVSMEEPGWYSFGFEIKDKKCDVPCIGIEMVHDGGKETWHSAATAIYCLMGSGQLLWDTVTGVDMAL |
| B/Temple/B10/1999 | 1999 | LPSTIQTLTLFLTSGGVLLSLYVSASLSYLLYSDILLKFSPKEITTPTMPLNCANASNVQAVNRSATKGVTLLLPEPEWTYPRLSCPGSTFQKALLISPHRFGETKGNSAPLIIREPFIACGPKECKHFALTHYAAQPGGYYNGTREDRNKLRHLISVKLGKIPTVENSIFHMAAWSGSACHDGREWTYIGVDGPDSNALLKIKYGEAYTDTYHSYANNILRTQESACNCIGGDCYLMITDGSASGISECRFLKIREGRIIKEIFPTGRVEHTEECTCGFASNKTIECACRDNSYTAKRPFVKLNVETDTAEIRLMCTETYLDTPRPDDGSITGPCESNGDKGSGGIKGGFVHQRMASKIGRWYSRTMSKTKRMGMGLYVKYDGDPWIDSDALTLSGVMVSMEEPGWYSFGFEIKDKKCDVPCIGIEMVHDGGKTTWHSAATAIYCLMGSGQLLWDTVTGVDMAL |
| B/Temple/B3/1999 | 1999 | LPSTIQTLTLFLTSGGVLLSLYVSASLSYLLYSDILLKFPPTEITAPTMPLDCANASNVQAVNRSATKGVTLLLPEPEWTYPRLSCPGSTFQKALLISPHRFGETKGNSAPLIIREPFIACGPKECKHFALTHYAAQPGGYYNGTREDRNKLRHLISVKLGKIPTVENSIFHMAAWSGSACHDGREWTYIGVDGPDSNALLKIKYGEAYTDTYHSYANNILRTQESACNCIGGNCYLMITDGSASGISECRFLKIREGRIIKEIFPTGRVKHTEECTCGFASNKTIECACRDNSYTAKRPFVKLNVETDTAEIRLMCTETYLDTPRPDDGSITGPCESNGDKGSGGIKGGFVHQRMASKIGRWYSRTMSKTKRMGMGLYVKYDGDPWTDSDPLALSGVMVSMEEPGWYSFGFEIKDKKCDVPCIGIEMVHDGGKETWHSAATAIYCLMGSGQMLWDTVTGVDMAL |
| B/Temple/B7/1999 | 1999 | LPSTIQTLTLFLTSGGVLLSLYVSASLSYLLYSDILLKFSPKEITAPTMPLNCANASNVQAVNRSATKGVTFPLPEPEWTYPRLSCPGSTFQKALLISPHRFGETKGNSAPLIIREPFIACGPKECKHFALTHYAAQPGGYYNGTREDRNKLRHLISVKLGKIPTVENSIFHMAAWSGSACHDGREWTYIGVDGPDSNALLKIKYGEAYTDTYHSYANNILRTQESACNCIGGDCYLMITDGSASGISECRFLKIREGRIIKEIFPTGRVEHTEECTCGFASNKTIECACRDNSYTAKRPFVKLNVETDTAEIRLMCTETYLDTPRPDDGSITGPCESNGDKGSGGIKGGFVHQRMASKIGRWYSRTMSKTKRMGMGLYVKYDGDPWIDSDALTLSGVMVSVEEPGWYSFGFEIKDKKCDVPCIGIEMVHDGGKTTWHSAATAIYCLMGSGQLLWDTVTGVDMAL |
| B/Temple/B8/1999 | 1999 | LPSTIQTLTLFLTSGGVLLSLYVSASLSYLLYSDILLKFSPKEITTPTMPLNCANASNVQAVNRSATKGVTLLLPEPEWTYPRLSCPGSTFQKALLISPHRFGETKGNSAPLIIREPFIACGPKECKHFALTHYAAQPGGYYNGTREDRNKLRHLISVKLGKIPTVENSIFHMAAWSGSACHDGREWTYIGVDGPDSNALLKIKYGEAYTDTYHSYANNILRTQESACNCIGGDCYLMITDGSASGISECRFLKIREGRIIKEIFPTGRVEHTEECTCGFASNKTIECACRDNSYTAKRPFVKLNVETDTAEIRLMCTETYLDTPRPDDGSITGPCESNGDKGSGGIKGGFVHQRMASKIGRWYSRTMSKTKRMGMGLYVKYDGDPWIDSDALTLSGVMVSMEEPGWYSFGFEIKDKKCDVPCIGIEMVHDGGKTTWHSAATAIYCLMGSGQLLWDTVTGVDMAL |
| B/Temple/B9/1999 | 1999 | LPSTIQTLTLFLTSGGVLLSLYVSASLSYLLYSDILLKFSPTEITAPTMPLNCANASNVQAVNRSATKGVTLPLPEPEWTYPRLSCPGSTFQKALLISPHRFGETKGNSAPLIIREPFIACGPKECKHFALTHYAAQPGGYYNGTREDRNKLRHLISVKLGKIPTVENSIFHMAAWSGSACHDGREWTYIGVDGPDSNALLKIKYGEAYTDTYHSYANNILRTQESACNCIGGDCYLMITDGSASGISECRFLKIREGRIIKEIFPTGRVEHTEECTCGFASNKTIECACRDNSYTAKRPFVKLNVETDTAEIRLMCTETYLDTPRPDDGSITGPCESNGDKGSGGIKGGFVHQRMASKIGRWYSRTMSKTKRMGMGLYVKYDGDPWIDSDALTLSGVMVSMEEPGWYSFGFEIKDKKCDVPCIGIEMVHDGGKTTWHSAATAIYCLMGSGQLLWDTVTGVDMAL |
| B/Vienna/1/99 | 1999 | LPSTIQTLTLFLTSGGVLLSLYVSASLSYLLYSDILLKFSPTEITAPTMPLDCANASNVQAVNRSATKGVTLLLPEPEWTYPRLSCPGSTFQKALLISPHRFGETKGNSAPLIIREPFIACGPKECKHFALTHYAAQPGGYYNGTREDRNKLRHLISVKLGKIPTVENSIFHMAAWSGSACHDGKEWTYIGVDGPDSNALLKIKYGEAYTDTYHSYANNILRTQESACNCIGGNCYLMITDGSASGISECRFLKIREGRIIKEIFPTGRVEHTEECTCGFASNKTIECACRDNSYTAKRPFVKLNVETDTAEIRLMCTETYLDTPRPDDGSITGPCESNGDKGSGGIKGGFVHQRMASKIGRWYSRTMSKTKRMGMGLYVKYDGDPWTDSDALALSGVMVSMEEPGWYSFGFEIKDKKCDVPCIGIEMVHDGGKETWHSAATAIYCLMGSGQLLWDTVTGVNMAL |
| B/Alaska/16/2000 | 2000 | LPSTIQTLTLFLTSGGVLLSLYVSASLSYLLYSDILLKLSPTEITAPTMPLDCANASNVQAVNRSATKGVTLLLPEPEWTYPRLSCPGSTFQKALLISPHRFGETKGNSAPLIIREPFIACGPKECKHFALTHYAAQPGGYYNGTREDRNKLRHLISVKLGKIPTVENSIFHMAAWSGSACHDGKEWTYIGVDGPDSNALLKIKYGEAYTDTYHSYANNILRTQESACNCIGGNCYLMITDGSASGISECRFLKIREGRIIKEIFPTGRVKHTEECTCGFASNKTIECACRDNSYTAKRPFVKLNVETDTAEIRLMCTETYLDTPRPDDGSITGPCESNGDKGSGGIKGGFVHQRMESKIGRWYSRTMSKTKRMGMGLYVKYDGDPWTDSDALALSGVMVSIEEPGWYSFGFEIKDKKCDVPCIGIEMVHDGGKETWHSAATAIYCLMGSGQLLWDTVTGVDMAL |
| B/Auckland/01/2000 | 2000 | LPSTIQTLTLFLTSGGVLLSLYVSASLSYLLYSGILLKFSPTEITAPTMPLNCANASNVQAVNRSATKGVTLLLPEPEWTYPRLSCPGSTFQKALLISPHRFGETKGNSAPLIIREPFIACGPKECKHFALTHYAAQPGGYYNGTREDRNKLRHLISVKLGKIPTVENSIFHMAAWSGSACHDGREWTYIGVDGPDSNALLKIKYGEAYTDTYHSYANNILRTQESACNCIGGDCYLMITDGSASGISECRFLKIREGRIIKEIFPTGRVEHTEECTCGFASNKTIECACRDNSYTAKRPFVKLNVETDTAEIRLMCTETYLDTPRPDDGSITGPCESHGDKGSGGIKGGFVHQRMASKIGRWYSRTMSKTKRMGMGLYVKYDGDPWIDSGALTLSGVMVSMEEPGWYSFGFEIKDKKCDVPCIGIEMVHDGGKKTWHSAATAIYCLMGSGQLLWDTVTGVDMAL |
| B/Canada/16188/2000 | 2000 | LPSTIQTLTLFLTSGGVLLSLYVSASLSYLLYSDILLKFSPTEITAPTMPLDCANASNVQAVNSSATKGVTLLLPEPEWTYPRLSCPGSTFQKALLISPHRFGETKGNSAPLIIREPFIACGPKECKHFALTHYAAQPGGYYNGTRGDRNKLRHLISVKLGKIPTVENSIFHMAAWSGSACHDGKEWTYIGVDGPDSNALLKIKYGEAYTDTYHSYANNILRTQESACNCIGGNCYLMITDGSASGISECRFLKIREGRIIKEIFPTGRVKHTEECTCGFASNKTIECACRDNSYTAKRPFVKLNVETDTAEIKLMCTETYLDTPRPDDGSITGPCESNGDKGSGGIKGGFVHQRMASKIGRWYSRTMSKTKRMGMGLYVKYDGDPWTDSDALALSGVMVSMEEPGWYSFGFEIKDKKCDVPCIGIEMVHDGGKETWHSAATAIYCLMGSGQLLWDTVTGVDMAL |
| B/Hong Kong/542/2000 | 2000 | LPSTIQTLTLFLTSGGVLLSLYVSASLSYLLYSDILLKFSQTEITAPTMPLDYANASNVQAVNRSATKGVTLLLPEPEWTYPRLSCPGSTFQKALLISPHRFGETKGNSAPLIIREPFIACGPKECKHFALTHYAAQPGGYYNGTREDRNKLRHLISVKLGKIPTVENSIFHMAAWSGSACHDGREWTYIGVDGPDSNALLKIKYGEAYTDTYHSYAKNILRTQESACNCIGGDCYLMITDGSASGISECRFLKIREGRIIKEIFPTGRVKHTEECTCGFASNKTIECACRDNSYTAKRPFVKLNVETDTAEIRLMCTETYLDTPRPDDGSITGPCESNGDKGSGGIKGGFVHQRMASKIGRWYSRTMSKTKRMGMGLYVKYDGDPWTDSEALALSGVMVSMEEPGWYSFGFEIKDKKCDVPCIGIEMVHDGGKTTWHSAATAIYCLMGSGQLLWDTVTGVDMAL |
| B/Hong Kong/548/2000 | 2000 | LPSTIQTLTLFLTSGGVLLSLYVSASLSYLLYSDILLKFSPTEITAPTMPLDCANASNVQAVNRSATKGVTLLLPEPEWTYPRLSCPGSIFQKALLISPHRFGETKGNSAPLIIREPFIACGPKECKHFALTHYAAQPGGYYNGTRGDRNKLRHLISVKLGKIPTVENSIFHMAAWSGSACHDGKEWTYIGVDGPDSNALLKIKYGEAYTDTYHSYANNILRTQESACNCIGGNCYLMITDGSASGISECRFLKIREGRIIKEIFPTGRVKHTEECTCGFASNKTIECACRDNSYTAKRPFVKLNVETDTAEIRLMCTETYLDTPRPDDGSITGPCESNGDKGSGGIKGGFVHQRMASKIGRWYSRTMSKTKRMGMGLYVKYDGDPWTDSDALALSGVMVSMEEPGWYSFGFEIKDKKCDVPCIGIEMVHDGGKETWHSAATAIYCLMGSGQLLWDTVTGVDMAL |
| B/Hong Kong/557/2000 | 2000 | LPSTIQTLTLFLTSGGVLLSLYVSASLSYLLYSDILLKFSQTEITAPTMPLDCANASNVQAVNRSATKGVTLLLPEPEWMYPRLSCPGSTFQKALLISPHRFGETKGNSAPLIIREPFIACGPKECKHFALTHYAAQPGGYYNGTREDRNKLRHLISVKLGKIPTVENSIFHMAAWSGSACHDGREWTYIGVDGPDSNALLKIKYGEAYTDTYHSYAKNILRTQESACNCIGGDCYLMITDGSASGISESRFLKIREGRIIKEIFPTGRVKHTEECTCGFASNKTIECACRDNSYTAKRPFVKLNVETDTAEIRLMCTETYLDTPRPDDGSITGPCESNGDKGSGGIKGGFVHQRMASKIGRWYSRTMSKTKRMGMGLYVKYDGDPWTDSEALALSGVMVSMEEPGWYSFGFEIKDKKCDVPCIGIEMVHDGGKTTWHSAATAIYCLMGSGQLLWDAVTGVDMAL |
| B/Houston/B66/2000 | 2000 | LPSTIQTLTLFLTSGGVLLSLYVSASLSYLLYSDILLKFSPTEITAPTMPLDCANASNVQAVNRSATKGVTLLLPEPEWTYPRLSCPGSTFQKALLISPHRFGETKGNSAPLIIREPFIACGPKECKHFALTHYAAQPGGYYNGTREDRNKLRHLISVKLGKIPTVENSIFHMAAWSGSACHDGKEWTYIGVDGPDSNALLKIKYGEAYTDTYHSYANNILRTQESACNCIGGNCYLMITDGSASGISECRFLKIREGRIIKEIFPTGRVKHTEECTCGFASNKTIECACRDNSYTAKRPFVKLNVETDTAEIRLMCTETYLDTPRPDDGSITGPCESNGDKGSGGIKGGFVHQRMASKIGRWYSRTMSKTKRMGMGLYVKYDGDPWTDSGALALSGVMVSMEEPGWYSFGFEIKDKKCDVPCIGIEMVHDGGKETWHSAATAIYCLMGSGQLLWDTVTGVDMAL |
| B/Houston/B67/2000 | 2000 | LPSTIQTLTLFLTSGGVLLSLYVSASLSYLLYSDILLKFSPTEITAPTMPLDCANASNVQAVNRSATKGVTFLLPEPEWTYPRLSCPGSTFQKALLISPHRFGETKGNSAPLIIREPFIACGPKECKHFALTHYAAQPGGYYNGTRGDRNKLRHLISVKLGKIPTVENSIFHMAAWSGSACHDGKEWTYIGVDGPDSNALLKIKYGEAYTDTYHSYANNILRTQESACNCIGGNCYLMITDGSASGISECRFLKIREGRIIKEIFPTGRVKHTEECTCGFASNKTIECACRDNSYTAKRPFVKLNVETDTAEIRLMCTETYLDTPRPDDGSITGPCESNGDKGSGGIKGGFVHQRMASKIGRWYSRTMSKTKRMGMGLYVKYDGDPWTDSDALALSGVMVSMEEPGWYSFGFEIKDKKCDVPCIGIEMVHDGGKETWHSAATAIYCLMGSGQLLWDTVTGVDMAL |
| B/Houston/B68/2000 | 2000 | LPSTIQTLTLFLTSGGVLLSLYVSASLSYLLYSDILLKFSPTEITAPTMPLDCANASNVQAVNRSSTKGVTFLLPEPEWTYPRLSCPGSTFQKALLISPHRFGETKGNSAPLIIREPFIACGPKECKHFALTHYAAQPGGYYNGTREDRNKLRHLISVKLGKIPTVENSIFHMAAWSGSACHDGKEWTYIGVDGPDSNALLKIKYGEAYTDTYHSYANNILRTQESACNCIGGNCYLMITDGSASGISECRFLKIREGRIIKEIFPTGRVKHTEECTCGFASNKTIECACRDNSYTAKRPFVKLNVETDTAEIRLMCTETYLDTPRPDDGSITGSCESDGDKGSGGIKGGFVHQRMASKIGRWYSRTMSKTKRMGMGLYVRYDGDPWTDSSALALSGVMVSMEEPGWYSFGFEIKDKKCDVPCIGIEMVHDGGKETWHSAATAIYCLMGSGQLLWDTVTGVDMAL |
| B/Mexico/84/2000 | 2000 | LPSTIQTLTLFLTSGGVLLSLYVSASLSYLLYSDILLKFSPTEITAPTMPLDCANASNVQAVNRSATKGVTLLLPEPEWTYPRLSCPGSTFQKALLISPHRFGETKGNSAPLIIREPFIACGPKECKHFALTHYAAQPGGYYNGTREDRNKLRHLISVKLGKIPTVENSIFHMAAWSGSACHDGKEWTYIGVDGPDSNALLKIKYGEAYTDTYHSYANNILRTQESACNCIGGNCYLMITDGSASGISECRFLKIREGRIIKEIFPTGRVKHTEECTCGFASNKTIECACRDNSYTAKRPFVKLNVETDTAEIRLMCTETYLDTPRPDDGSITGPCESNGDKGSGGIKGGFVHQRMASKIGRWYSRTMSKTKRMGMGLYVKYDGDPWTDSDALALSGVMVSMEEPGWYSFGFEIKDKKCDVPCIGIEMVHDGGKETWHSAATAIYCLMGSGQLLWDTVTGVDMAL |
| B/Nanchang/1/2000 | 2000 | LPSTIQTLTLFLTSGGVLLSLYVSALLSYLLYSDILLKFSPTKVIAPTMPLDCANASNVQAVNHSATEEMTFLLPEPEWTYPRLSCQGSTFQKALLISPHRFGEAKGNSAPLIIREPFIACGPKECKHFALTHYAAQPGGYYNGTREDRNKLRHLISVNLGKIPTVENSIFHMAAWSGSACHDGREWTYIGVDGPDSNALIKIKYGEAYTDTYHSYANNILRTQESACNCIGGDCYLMITDGSASGISRCRFLKIREGRIIKEIFPTGRVEHTEECTCGFASNKTIECACRDNSYTAKRPFVKLNVETDTAEIRLMCTETYLDTPRPDDGSITGPCESNGDKGNGGIKGGFVHQRMASRIGRWYSRTMSKTKRMGMELYVKYDGDPWTDSDALAPSGVMVSMEEPGWYSFGFEIKDKKCDVPCIGIEMVHDGGKKTWHSAATAIYCLMGSGQLLWDTVTGVNMAL |
| B/New York/1022/2000 | 2000 | LPSTIQTLTLFLTSGGVLLSLYVSASLSYLLYSDILLKFSPTEITAPTMPLDCANASNVQAVNRSATKGMTLLLPEPEWTYPRLSCPGSTFQKALLISPHRFGETKGNSAPLIIREPFIACGPKECKHFALTHYAAQPGGYYNGTREDRNKLRHLISVKLGKIPTVENSIFHMAAWSGSACHDGKEWTYIGVDGPDSNALLKIKYGEAYTDTYHSYANNILRTQESACNCIGGNCYLMITDGSASGISECRFLKIREGRIIKEIFPTGRVEHTEECTCGFASNKTIECACRDNSYTAKRPFVKLNVETDTAEIRLMCTETYLDTPRPDDGSITGPCESNGGKGSGGIKGGFVHQRMESKIGRWYSRTMSKTKRMGMGLYVKYDGDPWTDSDALALSGVMVSMEEPGWYSFGFEIKDKECDVPCIGIEMVHDGGKETWHSAATAIYCLMGSGQLLWDTVTGVNMAL |
| B/shenfang/77/2000 | 2000 | LPSTIQTLTLFLTSGGVLLSLYVSASLSYLLYSDILLKFSTTEITAPTMPLDCANASNVQAVNRSATKGVTLLLPEPEWTYPRLSCPGSTFQKALLISPHRFGETKGNSAPLIIREPFIACGPKECKHFALTHYAAQPGGYYNGTREDRNKLRHLISVKLGKIPTVENSIFHMAAWSGSACHDGKEWTYIGVDGPDSNALLKIKYGEAYTDTYHSYANNILRTQESACNCIGGNCYLMITDGSASGISECRFLKIREGRIIKEIFPTGRVKHTEECTCGFASNKTIECACRDNSYTAKRPFVKLNVETDTAEIRLMCTETYLDTPRPDDGSITGPCESNGDKGSGGIKGGFVHQRMASKIGRWYSRTMSKTKRMGMGLYVKYDGDPWTDSDALALSGVMVSMEEPGWYSFGFEIKDKKCDVPCIGIEMVHDGGKGTWHSAATAIYCLMGSGQLLWDTVTGVDMAL |
| B/Shizuoka/480/2000 | 2000 | LPSTIQTLTLFLTSGGVLLSLYVSASLSYLLYSDILLKFSSTEITAPTMPLDCANASNVQAVNRSATEGVTLLLPEPEWTYPRLSCPGSTFQKALLISPHRFGETKGNSAPLIIREPFIACGPKECKHFALTHYAAQPGGYYNGTREDRNKLRHLISVKLGKIPTVENSIFHMAAWSGSACHDGKEWTYIGVDGPDSNALLKIKYGEAYTDTYHSYANNILRTQESACNCIGGNCYLMITDGSASGISECRFLKIREGRIIKEIFPTGRVEHTEECTCGFASNKTIECACRDNSYTAKRPFVKLNVETDTAEIRLMCTETYLDTPRPDDGSITGPCESNGDKGSGGIKGGFVHQRMASKIGRWYSRTMSKTKRMGMGLYVKYDGDPWTDSDALALSGVMVSMEEPGWYSFGFEIKDKKCDVPCIGIEMVHDGGKETWHSAATAIYCLMGSGQLLWDTVTGVDMAL |
| B/South Carolina/NHRC0001/2000 | 2000 | LPSTIQTLTLFLTSGGVLLSLYVSASLSYLLYSDILLKFSPTEITAPTMPLDCANASNVQAVNRSATKGVTLLLPEPEWTYPRLSCPGSTFQKALLISPHRFGETKGNSAPLIIREPFIACGPKECKHFALTHYAAQPGGYYNGTRGDRNKLRHLISVKLGKIPTVENSIFHMAAWSGSACHDGKEWTYIGVDGPDSNALLKIKYGEAYTDTYHSYANNILRTQESACNCIGGNCYLMITDGSASGISECRFLKIREGRIIKEIFPTGRVKHTEECTCGFASNKTIECACRDNSYTAKRPFVKLNVETDTAEIRLMCTETYLDTPRPDDGSITGPCESNGDKGSGGIKGGFVHQRMASKIGRWYSRTMSKTKRMGMGLYVKYDGDPWTDSDALALSGVMVSMEEPGWYSFGFEIKDKKCDVPCIGIEMVHDGGKETWHSAATAIYCLMGSGQLLWDTVTGVDMAL |
| B/South Carolina/NHRC0002/2000 | 2000 | LPSTIQTLTLFLTSGGVLLSLYVSASLSYLLYSDILLKFSPTEITAPTMPLDCANASNVQAVNRSATKGVTLLLPEPEWTYPRLSCPGSTFQKALLISPHRFGETKGNSAPLIIREPFIACGPKECKHFALTHYAAQPGGYYNGTRGDRNKLRHLISVKLGKIPTVENSIFHMAAWSGSACHDGKEWTYIGVDGPDSNALLKIKYGEAYTDTYHSYANNILRTQESACNCIGGNCYLMITDGSASGISECRFLKIREGRIIKEIFPTGRVKHTEECTCGFASNKTIECACRDNSYTAKRPFVKLNVETDTAEIRLMCTETYLDTPRPDDGSITGPCESNGDKGSGGIKGGFVHQRMASKIGRWYSRTMSKTKRMGMGLYVKYDGDPWTDSDALALSGVMVSMEEPGWYSFGFEIKDKKCDVPCIGIEMVHDGGKETWHSAATAIYCLMGSGQLLWDTVTGVDMAL |
| B/Texas/06/2000 | 2000 | LPSTIQTLTLFLTSGGVLLSLYVSASLSYLLYSDILLKFSPTEITAPTMPLDCANASNVQAVNRSATKGVTLLLPEPEWTYPRLSCPGSTFQKALLISPHRFGETKGNSAPLIIREPFIACGPKECKHFALTHYAAQPGGYYNGTREDRNKLRHLISVKLGKIPTVENSIFHMAAWSGSACHDGKEWTYIGVDGPDSNALLKIKYGEAYTDTYHSYANNILRTQESACNCIGGNCYLMITDGSASGISECRFLKIREGRIIKEIFPTGRVKHTEECTCGFASNKTIECACRDNSYTAKRPFVKLNVETDTAEIRLMCTETYLDTPRPDDGSITGPCESNGDKGSGGIKGGFVHQRMASKIGRWYSRTMSKTKRMGMGLYVKYDGDPWTDSDALALSGVMVSMEEPGWYSFGFEIKDKKCDVPCIGIEMVHDGGKETWHSAATAIYCLMGSGQLLWDTVTGVDMAM |
| B/Texas/1/2000 | 2000 | LPSTIQTLTLFLTSGGVLLSLYVSASLSYLLYSDILLKFSPTEITAPTMPLDCANASNVQAVNRSATKGVTLLLPEPEWTYPRLSCPGSTFQKALLISPHRFGETKGNSAPLIIREPFIACGPKECKHFALTHYAAQPGGYYNGTREDRNKLRHLISVKLGKIPTVENSIFHMAAWSGSACHDGKEWTYIGVDGPDSNALLKIKYGEAYTDTYHSYANNILRTQESACNCIGGNCYLMITDGSASGISECRFLKIREGRIIKEIFPTGRVKHTEECTCGFASNKTIECACRDNSYTAKRPFVKLNVETDTAEIRLMCTETYLDTPRPDDGSITGPCESNGDKGSGGIKGGFVHQRMASKIGRWYSRTMSKTKRMGMGLYVKYDGDPWTDSDALALSGVMVSMEEPGWYSFGFEIKDKKCDVPCIGIEMVHDGGKETWHSAATAIYCLMGSGQLLWDTVTGVDMAL |
| B/Texas/12/2000 | 2000 | LPSTIQTLTLFLTSGGVLLSLYVSASLSYLLYSDILLKFSQTEITAPTMPLDCANASNVQAANRSATKGVTLLLPEPEWTYPRLSCPGSTFQKALLISPHRFGETKGNSAPLIIREPFIACGPKECKHFALTYYAAQPGGYYNGTREDRNKLRHLISVKLGKIPTVENSIFHMAAWSGSACHDGREWTYIGVDGPDSNALLKIKYGEAYTDTYHSYAKNILRTQESACNCIGGDCYLMITDGSASGISECRFLKIREGRIIKEIFPTGRVKHTEECTCGFASNKTIECACRDNSYTAKRPFVKLNVETDTAEIRLMCTETYLDTPRPNDGSITGPCESDGDKGSGGIKGGFVHQRMASKIGRWYSRTMSKTKRMGMGLYVKYDGDPWTDSEALALSGVMVSMEEPGWYSFGFEIKDKKCDVPCIGIEMVHDGGKTTWHSAATAIYCLMGSGQLLWDTVTGVDMAL |
| B/Wuhan/356/2000 | 2000 | LPSTIQTLTLFLTSGGVLLSLYVSASLSYLLYSDILLKFSQTEITAPTMPLDCANASNVQAVNRSATKGVTLLLPEPEWTYPRLSCPGSTFQKALLISPHRFGETKGNSAPLIIREPFIACGPKECKHFALTHYAAQPGGYYNGTREDRNKLRHLISVKLGKIPTVENSIFHMAAWSGSACHDGREWTYIGVDGPDSNALLKIKYGEAYTDTYHSYAKNILRTQESACNCIGGDCYLMITDGSASGISECRFLKIREGRIIKEIFPTGRIKHTEECTCGFASNKTIECACRDNSYTAKRPFVKLNVETDTAEIRLMCTETYLDTPRPDDGSITGPCESNGDKGSGGIKGGFVHQRMASKIGRWYSRTMSKTKRMGMGLYVKYDGDPWTDSEALALSGVMVSMEEPGWYSFGFEIKDKKCDVPCIGIEMVHDGGKTTWHSAATAIYCLMGSGQLLWDTVTGVDMAL |
| B/Akita/27/2001 | 2001 | LPSTIQTLTLFLTSGGVLLSLYVSALLSYLLYSDILLKFSPTKIIAPTTSLDSANASNFQAVNHSATKEMTFLLPEPEWTYPRLSCQGSTFQKALLISPHRFGEAKGNSAPLIIREPFIACGPKECKHFALTHYAAQPGGYYNGTREDRNKLRHLISVNLGKIPTVENSIFHMAAWSGSACHDGREWTYIGVDGPDSNALIKIKYGEAYTDTYHSYANNILRTQESACNCIGGDCYLMITDGSASGISKCRFLKIREGRIIKEIFPTGRVEHTEECTCGFASNKTIECACRDNSYTAKRPFVKLNVETDTAEIRLMCTETYLDTPRPDDGSITGPCESNGDKGSGGIKGGFVHQRMASKIGRWYSRTMSKTKRMGMELYVKYDGDPWTDSDALAPSGVMVSIEEPGWYSFGFEIKDKKCDVPCIGIEMVHDGGKTTWYSAATAIYCLMGSGQLLWDTVTGVDMAL |
| B/Akita/34/2001 | 2001 | LPSTIQTLTLFLTSGGVLLSLYVSASLSYLLYSDILLKFSPTEITAPTMPLDCANASNVQAVNRSATKGVTLLLPEPEWTYPRLSCPGSAFQKALLISPHRFGETKGNSAPLIIREPFIACGPKECKHFALTHYAAQPGGYYNGTRGDRNKLRHLISVKLGKIPTVENSIFHMAAWSGSACHDGKEWTYIGVDGPDNNALLKIKYGEAYTDTYHSYANNILRTQESACNCIGGNCYLMITDGSASGISECRFLKIREGRIIKEIFPTGRVKHTEECTCGFASNKTIECACRDNSYTAKRPFVKLNVETDTAEIRLMCTETYLDTPRPDDGSITGPCESNGDKGSGGIKGGFVHQRMASKIGRWYSRTMSKTKRMGMGLYVKYDGDPWTDSDALALSGVMVSMEEPGWYSFGFEIKDKKCDVPCIGIEMVHDGGKETWHSAATAIYCLMGSGQLLWDTVTGVDMAL |
| B/Argentina/132/2001 | 2001 | LPSTIQTLTLFLTSGGVLLSLYVSASLSYLLYSDILLKFSQTEITAPTMPLDCANASNVQAVNRSATKGVTLLLPEPEWTYPRLSCPGSTFQKALLISPHRFGETKGNSAPLIIREPFIACGPKECKHFALTHYAAQPGGYYNGTREDRNKLRHLISVKLGKIPTVENSIFHMAAWSGSACHDGREWTYIGVDGPDSNALLKIKYGEAYTDTYHSYAKNILRTQESACNCIGGDCYLMITDGSASGISECRFLKIREGRIIKEIFPTGRVKHTEECTCGFASNKTIECACRDNSYTAKRPFVKLNVETDTAEIRLMCTETYLDTPRPNDGSITGPCESNGDKGSGGIKGGFVHQRMASKIGRWYSRTMSKTKRMGMGLYVKYDGDPWTDSEALALSGVMVSMEEPGWYSFGFEIKDKKCDVPCIGIEMVHDGGKTTWHSAATAIYCLMGSGQLLWDTVTGVDMAL |
| B/Argentina/69/2001 | 2001 | LPSTIQTLTLFLTSGGVLLSLYVSASLSYLLYSDILLKFSQTEITAPTMPLDCANASNVQAVNRSATKGVTLLLPEPEWTYPRLSCPGSTFQKALLISPHRFGETKGNSAPLIIREPFIACGPKECKHFALTHYAAQPGGYYNGTREDRNKLRHLISVKLGKIPTVENSIFHMAAWSGSACHDGREWTYIGVDGPDSNALLKIKYGEAYTDTYHSYAKNILRTQESACNCIGGDCYLMITDGSASGISECRFLKIREGRIIKEIFPTGRVKHTEECTCGFASNKTIECACRDNSYTAKRPFVKLNVETDTAEIRLMCTETYLDTPRPNDGSITGPCESNGDKGSGGIKGGFVHQRMASKIGRWYSRTMSKTKRMGMGLYVKYDGDPWTDSEALALSGVMVSMEEPGWYSFGFEIKDKKCDVPCIGIEMVHDGGKTTWHSAATAIYCLMGSGQLLWDTVTGVDMAL |
| B/Auckland/2/2001 | 2001 | LPSTIQTLTLFLTSGGVLLSLYVSASLSYLLYSDILLKFSQTEITAPTMPLDCANASNVQAANRSATKGVTLLLPEPEWTYPRLSCPGSTFQKALLISPHRFGETKGNSAPLIIREPFIACGPKECKHFALTYYAAQPGGYYNGTREDRNKLRHLISVKLGKIPTVENSIFHMAAWSGSACHDGREWTYIGVDGPDSNALLKIKYGEAYTDTYHSYAKNILRTQESACNCIGGDCYLMITDGSASGISECRFLKIREGRIIKEIFPTGRVKHTEECTCGFASNKTIECACRDNSYTAKRPFVKLNVETDTAEIRLMCTETYLDTPRPNDGSITGPCESDGDKGSGGIKGGFVHQRMASKIGRWYSRTMSKTKRMGMGLYVKYDGDPWTDSEALALSGVMVSMEEPGWYSFGFEIKDKKCDVPCIGIEMVHDGGKTTWHSAATAIYCLMGSGQLLWDTVTGVDMAL |
| B/Hawaii/36/2001 | 2001 | LPSTIQTLTLFLTSGGVLLSLYVSALLSYLLYSDILLKFSPTKIIAPTTSLDSANASNFQAVNHSATKEMTFLLPEPEWTYPRLSCQGSTFQKALLISPHRFGEAKGNSAPLIIREPFIACGPKECKHFALTHYAAQPGGYYNGTREDRNKLRHLISVNLGKIPTVENSIFHMAAWSGSACHDGREWTYIGVDGPDSNALIKIKYGEAYTDTYHSYANNILRTQESACNCIGGDCYLMITDGSASGISKCRFLKIREGRIIKEIFPTGRVEHTEECTCGFASNKTIECACRDNSYTAKRPFVKLNVETDTAEIRLMCTETYLDTPRPDDGSITGPCESNGDKGSGGIKGGFVHQRMASKIGRWYSRTMSKTKRMGMELYVKYDGDPWTDSDALAPSGVMVSIEEPGWYSFGFEIKDKKCDVPCIGIEMVHDGGKTTWHSAATAIYCLMGSGQLLWDTVTGVDMAL |
| B/Hong Kong/123/2001 | 2001 | LPSTIQTLTLFLTSGGVLLSLYVSALLSYLLYSDILLKFSPTKIIAPTTSLDSANASNFQAVNHSATKEMTFLLPEPEWTYPRLSCQGSTFQKALLISPHRFGEAKGNSAPLIIREPFIACGPKECKHFALTHYAAQPGGYYNGTREDRNKLRHLISVNLGKIPTVENSIFHMAAWSGSACHDGREWTYIGVDGPDSNALIKIKYGEAYTDTYHSYANNILRTQESACNCIGGDCYLMITDGSASGISKCRFLKIREGRIIKEIFPTGRVEHTEECTCGFASNKTIECACRDNSYTAKRPFVKLNVETDTAEIRLMCTETYLDTPRPDDGSITGPCESNGDKGSGGIKGGFVHQRMASKIGRWYSRTMSKTKRMGMELYVKYDGDPWTDSDALAPSGVMVSIEEPGWYSFGFEIKDKKCDVPCIGIEMVHDGGKTTWHSAATAIYCLMGSGQLLWDTVTGVDMAL |
| B/Hong Kong/22/2001 | 2001 | LPSTIQTLTLFLTSGGVLLSLYVSALLSYLLYSDILLKFSPTKIIAPTTSLDSANASNFQAVNHSATKEMTFLLPEPEWTYPRLSCQGSTFQKALLISPHRFGEAKGNSAPLIIREPFIACGPKECKHFALTHYAAQPGGYYNGTREDRNKLRHLISVNLGKIPTVENSIFHMAAWSGSACHDGREWTYIGVDGPDSNALIKIKYGEAYTDTYHSYANNILRTQESACNCIGGDCYLMITDGSASGISKCRFLKIREGRIIKEIFPTGRVEHTEECTCGFASNKTIECACRDNSYTAKRPFVKLNVETDTAEIRLMCTETYLDTPRPDDGSITGPCESNGDKGSGGIKGGFVHQRMASKIGRWYSRTMSKTKRMGMELYVKYDGDPWTDSDALAPSGVMVSIEEPGWYSFGFEIKDKKCDVPCIGIEMVHDGGKTTWHSAATAIYCLMGSGQLLWDTVTGVDMAL |
| B/Hong Kong/330/2001 | 2001 | LPSTIQTLTLFLTSGGVLLSLYVSALLSYLLYSDILLKFSPTKIIAPTTSLDSANASNFQAVNHSATKEMTFLLPEPEWTYPRLSCQGSTFQKALLISPHRFGEAKGNSAPLIIREPFIACGPKECKHFALTHYAAQPGGYYNGTREDRNKLRHLISVNFGKIPTVENSIFHMAAWSGSACHDGREWTYIGVDGPDSNALIKIKYGEAYTDTYHSYANNILRTQESACNCIGGDCYLMITDGSASGISKCRFLKIREGRIVKEIFPTGRVEHTEECTCGFASNKTIECACRDNSYTAKRPFVKLNVETDTAEIRLMCTETYLDTPRPDDGSITGPCESNGDKGSGGIKGGFVHQRMASKIGRWYSRTMSKTKRMGMELYVKYDGDPWTDSDALAPSGVMVSIEEPGWYSFGFEIKDKKCDVPCIGIEMVHDGGKTTWHSAATAIYCLMGSGQLLWDTITGVDMAL |
| B/Iwate/1038/2001 | 2001 | LPSTIQTLTLFLTSGGVLLSLYVSASLSYLLYSDILLKFSPTEITAPTMPLDCANASNVQAVNRSATKGVTLLLPEPEWTYPRLSCPGSIFQKALLISPHRFGETKGNSAPLIIREPFIACGPKECKHFALTHYAAQPGGYYNGTRGDRNKLRHLISVKLGKIPTVENSIFHMAAWSGSACHDGKEWTYIGVDGPDSNALLKIKYGEAYTDTYHSYANNILRTQESACNCIGGNCYLMITDGSASGISECRFLKIREGRIIKEIFPTGRVKHTEECTCGFASNKTIECACRDNSYTAKRPFVKLNVETDTAEIRLMCTETYLDTPRPDDGSITGPCESNGDKGSGGIKGGFVHQRMASKIGRWYSRTMSKTKRMGMGLYVKYDGDPWTDSDALALSGVMVSMEEPGWYSFGFEIKDKKCDVPCIGIEMVHDGGKETWHSAATAIYCLMGSGQLLWDTVTGVDMAL |
| B/Kagoshima/2/2001 | 2001 | LPSTIQTLTLFLTSGGVLLSLYVSASLSYLLYSDILLKFSPTEITAPTMPLDCANASNVQAVNRSATKGVTLLLPEPEWTYPRLSCPGSTFQKALLISPHRFGETKGNSAPLIIREPFIAWGPKECKHFALTHYAAQPGGYYNGTRGDRNKLRHLISVKLGKIPTVENSIFHMAAWSGSACHDGKEWTYIGVDGPDSNALLKIKYGEAYTDTYHSYANNILRTQESACNCIGGNCYLMITDGSASGISECRFLKIREGRIIKEIFPAGRVKHTEECTCGFASNKTIECACRDNSYTAKRPFVKINVETDTAEIRLMCTETYLDTPRPDDGSITGPCESNGDKGSGGIKGGFVHQRMASKIGRWYSRTMSKTKRMGMGLYVKYDGDPWTDSDALALSGVMVSMEEPGWYSFGFEIKDKKCDVPCIGIEMVHDGGKETWHSAATAIYCLMGSGQLLWDTVTGVDMAL |
| B/Malaysia/83077/2001 | 2001 | LPSTIQTLTLFLTSGGVLLSLYVSALLSYLLYSDILLKFSPTKVIAPTMSLDYANASNVQAVNHSATEEMTFLLPEPEWTYPRLSCQGSTFQKALLISPHRFGEAKGNSAPLIIREPFIACGPKECKHFALTHYAAQPGGYYNGTREDRNKLRHLISVNLGKIPTVENSIFHMAAWSGSACHDGREWTYIGVDGPDSNALIKIKYGEAYTDTYHSYANNILRTQESACNCIGGDCYLMITDGSASGISRCRFLKIREGRIIKEIFPTGRVEHTEECTCGFASNKTIECACRDNSYTAKRPFVKLNVETDTAEIRLMCTETYLDTPRPDDGSITGPCESNGDKGNGGIKGGFVHQRMASRIGRWYSRTMSKTKRMGMELYVKYDGDPWTDSDALAPSGVMVSMEEPGWYSFGFEIKDKKCDVPCIGIEMVHDGGKKTWHSAATAIYCLMGSGQLLWDTVTGVDMAL |
| B/Memphis/1/01 | 2001 | LPSTIQTLTLFLTSGGVLLSLYVSASLSYLLYSDILLKFSPTEITAPTMPLDCANASNVQAVNRSATKGVTLLLPEPEWTYPRLSCPGSTFQKALLISPHRFGETKGNSAPLIIREPFIACGPKECKHFALTHYAAQPGGYYNGTRGDRNKLRHLISVKLGKIPTVENSIFHMAAWSGSACHDGKEWTYIGVDGPDSNALLKIKYGEAYTDTYHSYANNILRTQESACNCIGGNCYLMITDGSASGISECRFLKIREGRIIKEIFPTGRVKHTEECTCGFASNKTIECACRDNSYTAKRPFVKLNVETDTAEIRLMCTETYLDTPRPDDGSITGPCESNGDKGSGGIKGGFVHQRMASKIGRWYSRTMSKTKRMGMGLYVKYDGDPWTDSDALALSGVMVSMEEPGWYSFGFEIKDKKCDVPCIGIEMVHDGGKETWHSAATAIYCLMGSGQLLWDTVTGVDMAL |
| B/Memphis/3/01 | 2001 | LPSTIQTLTLFLTSGGVLLSLYVSASLSYLLYSDILLKFSPTEITAPTMPLDCANASNVQAVNRSATKGVTLLLPEPEWTYPRLSCPGSTFQKALLISPHRFGETKGNSAPLIIREPFIACGPKECKHFALTHYAAQPGGYYNGTREDRNKLRHLISVKLGKIPTVENSIFHMAAWSGSACHDGKEWTYIGVDGPDSNALLKIKYGEAYTDTYHSYANNILRTQESACNCIGGNCYLMITDGSASGISECRFLKIREGRIIKEIFPTGRVEHTEECTCGFASNKTIECACRDNSYTAKRPFVKLNVETDTAEIRLMCTETYLDTPRPDDGSITGPCESNGDKGSGGIKGGFVHQRMASKIGRWYSRTMSKTKRMGMGLYVKYDGDPWTDSDALALSGVMVSVGEPGWYSFGFEIKDKKCDVPCIGIEMVHDGGKETWHSAATAIYCLMGSGQLLWDTVTGVDMAL |
| B/Missouri/NHRC0001/2001 | 2001 | LPSTIQTLTLFLTSGGVLLSLYVSASLSYLLYSDILLKFSPTEITAPTMPLDCANASNVQAVNRSATKGVTLLLPEPEWTYPRLSCPGSTFQKALLISPHRFGETKGNSAPLIIREPFIACGPKECKHFALTHYAAQPGGYYNGTRGDRNKLRHLISVKLGKIPTVENSIFHMAAWSGSACHDGKEWTYIGVDGPDSNALLKIKYGEAYTDTYHSYANNILRTQESACNCIGGNCYLMITDGSASGISECRFLKIREGRIIKEIFPTGRVKHTEECTCGFASNKTIECACRDNSYTAKRPFVKLNVETDAAEIRLMCTETYLDTPRPDDGSITGPCESNGDKGSGGIKGGFVHQRMASKIGRWYSRTMSKTKRMGMGLYVKYDGDPWTDSDALALSGVMVSMEEPGWYSFGFEIKDKKCDVPCIGIEMVHDGGKETWHSAATAIYCLMGSGQLLWDTVTGVDMAL |
| B/Nebraska/1/01 | 2001 | LPSTIQTLTLFLTSGGVLLSLYVSASLSYLLYSDILLKFSPTEITAPTMPLDCANASNVQAVNRSATKGVALLLPEPEWTYPRLSCPGSTFQKALLISPHRFGETKGNSAPLIIREPFIACGPKECKHFALTHYAAQPGGYYNGTRGDRNKLRHLISVKLGKIPTVENSIFHMAAWSGSACHDGKEWTYIGVDGPDSNALLKIKYGEAYTDTYHSYANNILRTQESACNCIGGNCYLMITDGSASGISECRFLKIREGRIIKEIFPTGRVKHTEECTCGFASNKTIECACRDNSYTAKRPFVKLNVETDTAEIRLMCTETYLDTPRPDDGSITGPCESNGDKGSGGIKGGFVHQRMASKIGRWYSRTMSKTKRMGMGLYVKYDGDPWTDSDALALSGVMVSMEEPGWYSFGFEIKDKKCDVPCIGIEMVHDGGKETWHSAATAIYCLMGSGQLLWDTVTGVDMAL |
| B/New Jersey/02/2001 | 2001 | LPSTIQTLTLFLTSGGVLLSLYVSASLSYLLYSDILLKFSPTEITAPTMPLDCANASNVQAVNRSATKGVTLLLPKPEWTYPRLSCPGSTFQKALLISPHRFGETKGNSAPLIIREPFIACGPKECKHFALTHYAAQPGGYYNGTRGDRNKLRHLISVKLGKIPTVENSIFHMAAWSGSACHDGKEWTYIGVDGPDSNALLKIKYGEAYTDTYHSYANNILRTQESACNCIGGNCYLMITDGSASGISECRFLKIREGRIIKEIFPTGRVKHTEECTCGFASNKTIECACRDNSYTAKRPFVKLNVETDTAEIRLMCTETYLDTPRPDDGSITGPCESNGDKGSGGIKGGFVHQRMASKIGRWYSRTMSKTKRMGMGLYVKYDGDPWTDSDALALSGVMVSMEEPGWYSFGFEIKDKKCDVPCIGIEMVHDGGKETWHSAATAIYCLMGSGQLLWDTVTGVDMAL |
| B/New York/1024/2001 | 2001 | LPSTIQTLTLFLTSGGVLLSLYVSASLSYLLYSDILLKFSPTEITAPTMPLDCANASNVQAVNRSATKGMTLLLPEPEWTYPRLSCPGSTFQKALLISPHRFGETKGNSAPLIIREPFIACGPKECKHFALTHYAAQPGGYYNGTREDRNKLRHLISVKLGKIPTVENSIFHMAAWSGSACHDGKEWTYIGVDGPDSNALLKIKYGEAYTDTYHSYANNILRTQESACNCIGGNCYLMITDGSASGISECRFLKIREGRIIKEIFPTGRVEHTEECTCGFASNKTIECACRDNSYTAKRPFVKLNVETDTAEIRLMCTETYLDTPRPDDGSITGPCESNGGKGSGGIKGGFVHQRMESKIGRWYSRTMSKTKRMGMGLYVKYDGDPWTDSDALALSGVMVSMEEPGWYSFGFEIKDKECDVPCIGIEMVHDGGKETWHSAATAIYCLMGSGQLLWDTVTGVNMAL |
| B/New York/1025/2001 | 2001 | LPSTIQTLTLFLTSGGVLLSLYVSASLSYLLYSDILLKFSPTEITAPIMPLDCANASNVQAVNRSATKGVTLLLPEPEWTYPRLSCPGSTFQKALLISPHRFGETKGNSAPLIIREPFIACGPKECKHFALTHYAAQPGGYYNGTRGDRNKLRHLISVKLGKIPTVENSIFHMAAWSGSACHDGKEWTYIGVDGPDNNALLKIKYGEAYTDTYHSYANNTLRTQESACNCIGGNCYLMITDGSASGISECRFLKIREGRIIKEIFPTGRVKHTEECTCGFASNKTIECACRDNSYTAKRPFVKLNVETDTAEIRLMCTETYLDTPRPDDGSITGPCESNGNKGSGGIKGGFVHQRMASKIGRWYSRTMSKTKRMGMGLYVKYDGDPWTDSDALALSGVMVSMEEPGWYSFGFEIKDKKCDVPCIGIEMVHDGGKETWHSAATAIYCLMGSGQLLWDTVTGVDMAL |
| B/New York/1027/2001 | 2001 | LPSTIQTLTLFLTSGGVLLSLYVSASLSYLLYSDILLKFSPTEITAPTMPSDCANASNVQAVNRSATKGVTLLLPEPEWTYPRLSCPGSTFQKALLISPHRFGETKGNSAPLIIREPFIACGPKECKHFALTHYAAQPGGYYNGTRGDRNKLRHLISVKLGKIPTVENSIFHMAAWSGSACHDGKEWTYIGVDGPDSNALLKIKYGEAYTDTYHSYANNILRTQESACNCIGGNCYLMITDGSASGISECRFLKIREGRIIKEIFPTGRVKHTEECTCGFASNKTIECACRDNSYTAKRPFVKLNVETDTAEIRLMCTETYLDTPRPDDGSITGPCESNGDKGSGGIKGGFVHQRMASKIGRWYSRTMSKTKRMGMGLYVKYDGDPWTDSDALALSGVMVSMEEPGWYSFGFEIKDKKCDVPCIGIEMVHDGGKETWHSAATAIYCLMGSGQLLWDTVTGVDMAL |
| B/New York/1028/2001 | 2001 | LPSTIQTLTLFLTSGGVLLSLYVSASLSYLLYSDILLKFSPTEITAPTMPLDCANASNVQAVNRSATKGMTLLLPEPEWTYPRLSCPGSTFQKALLISPHRFGETKGNSAPLIIREPFIACGPKECKHFALTHYAAQPGGYYNGTREDRNKLRHLISVKLGKIPTVENSIFHMAAWSGSACHDGKEWTYIGVDGPDSNALLKIKYGEAYTDTYHSYANNILRTQESACNCIGGNCYLMITDGSASGISECRFLKIREGRIIKEIFPTGRVEHTEECTCGFASNKTIECACRDNSYTAKRPFVKLNVETDTAEIRLMCTETYLDTPRPDDGSITGPCESNGGKGSGGIKGGFVHQRMESKIGRWYSRTMSKTKRMGMGLYVKYDGDPWTDSDALALSGVMVSMEEPGWYSFGFEIKDKKCDVPCIGIEMVHDGGKETWHSAATAIYCLMGSGQLLWDTVTGVNMAL |
| B/New York/1029/2001 | 2001 | LPSTIQTLTLFLTSGGVLLSLYVSASLSYLLYSDILLKFSPTEITAPTMPLDCANASNVQAVNRSATKGMTLLLPEPEWTYPRLSCPGSTFQKALLISPHRFGETKGNSAPLIIREPFIACGPKECKHFALTHYAAQPGGYYNGTREDRNKLRHLISVKLGKIPTVENSIFHMAAWSGSACHDGKEWTYIGVDGPDSNALLKIKYGEAYTDTYHSYANNILRTQESACNCIGGNCYLMITDGSASGISECRFLKIREGRIIKEIFPTGRVEHTEECTCGFASNKTIECACRDNSYTAKRPFVKLNVETDTAEIRLMCTETYLDTPRPDDGSITGPCESNGGKGSGGIKGGFVHQRMESKIGRWYSRTMSKTKRMGMGLYVKYDGDPWTDSDALALSGVMVSMEEPGWYSFGFEIKDKKCDVPCIGIEMVHDGGKETWHSAATAIYCLMGSGQLLWDTVTGVNMAL |
| B/New York/1031/2001 | 2001 | LPSTIQTLTLFLTSGGVLLSLYVSASLSYLLYSDILLKFSPTEITAPTMPLDCANASNVQAVNRSATKGVTLLLPEPEWTYPRLSCPGSTFQKALLISPHRFGETKGNSAPLIIREPFIACGPKECKHFALTHYAAQPGGYYNGTRGDRNKLRHLISVKLGKIPTVENSIFHMAAWSGSACHDGKEWTYIGVDGPDNNALLKIKYGEAYTDTYHSYANNILRTQESACNCIGGNCYLMITDGSASGISECRFLKIREGRIIKEIFPTGRVKHTEECTCGFASNKTIECACRDNSYTAKRPFVKLNVETDTAEIRLMCTETYLDTPRPDDGSITGPCESNGDKGSGGIKGGFVHQRMASKIGRWYSRTMSKTKRMGMGLYVKYDGDPWTDSDALALSGVMVSMEEPGWYSFGFEIKDKKCDVPCIGIEMVHDGGKETWHSAATAIYCLMGSGQLLWDTVTGVDMAL |
| B/New York/1032/2001 | 2001 | LPSTIQTLTLFLTSGGVLLSLYVSASLSYLLYSDILLKFSPTEITAPTMPLDCANASNVQAVNRSATKGMTLLLPEPEWTYPRLSCPGSTFQKALLISPHRFGETKGNSAPLIIREPFIACGPKECKHFALTHYAAQPGGYYNGTREDRNKLRHLISVKLGKIPTVENSIFHMAAWSGSACHDGKEWTYIGVDGPDSNALLKIKYGEAYTDTYHSYANNILRTQESACNCIGGNCYLMITDGSASGISECRFLKIREGRIIKEIFPTGRVEHTEECTCGFASNKTIECACRDNSYTAKRPFVKLNVETDTAEIRLMCTETYLDTPRPDDGSITGPCESNGGKGSGGIKGGFVHQRMESKIGRWYSRTMSKTKRMGMGLYVKYDGDPWTDSDALALSGVMVSMEEPGWYSFGFEIKDKKCDVPCIGIEMVHDGGKETWHSAATAIYCLMGSGQLLWDTVTGVNMAL |
| B/New York/1034/2001 | 2001 | LPSTIQTLTLFLTSGGVLLSLYVSASLSYLLYSDILLKFSPTEITAPTMPLDCANASNVQAVNRSATKGMTLLLPEPEWTYPRLSCPGSTFQKALLISPHRFGETKGNSAPLIIREPFIACGPKECKHFALTHYAAQPGGYYNGTREDRNKLRHLISVKLGKIPTVENSIFHMAAWSGSACHDGKEWTYIGVDGPDSNALLKIKYGEAYTDTYHSYANNILRTQESACNCIGGNCYLMITDGSASGISECRFLKIREGRIIKEIFPTGRVEHTEECTCGFASNKTIECACRDNSYTAKRPFVKLNVETDTAEIRLMCTETYLDTPRPDDGSITGPCESNGGKGSGGIKGGFVHQRMESKIGRWYSRTMSKTKRMGMGLYVKYDGDPWTDSDALALSGVMVSMEEPGWYSFGFEIKDKKCDVPCIGIEMVHDGGKETWHSAATAIYCLMGSGQLLWDTVTGVNMAL |
| B/New York/1036/2001 | 2001 | LPSTIQTLTLFLTSGGVLLSLYVSASLSYLLYSDILLKFSPTEITAPTMPLDCANASNVQAVNRSATKGMTLLLPEPEWTYPRLSCPGSTFQKALLISPHRFGETKGNSAPLIIREPFIACGPKECKHFALTHYAAQPGGYYNGTREDRNKLRHLISVKLGKIPTVENSIFHMAAWSGSACHDGKEWTYIGVDGPDSNALLKIKYGEAYTDTYHSYANNILRTQESACNCIGGNCYLMITDGSASGISECRFLKIREGRIIKEIFPTGRVEHTEECTCGFASNKTIECACRDNSYTAKRPFVKLNVETDTAEIRLMCTETYLDTPRPDDGSITGPCESNGGKGSGGIKGGFVHQRMESKIGRWYSRTMSKTKRMGMGLYVKYDGDPWTDSDALALSGVMVSMEEPGWYSFGFEIKDKKCDVPCIGIEMVHDGGKETWHSAATAIYCLMGSGQLLWDTVTGVNMAL |
| B/New York/1039/2001 | 2001 | LPSTIQTLTLFLTTGGVLLSLYVSASLSYLLYSDILLKFSPTEITAPTMPLDCANASNVQAVNRSATKGVTFLLPEPEWTYPRLSCPGSTFQKALLISPHRFGETKGNSAPLIIREPFIACGPKECKHFALTHYAAQPGGYYNGTRGDRNKLRHLISVKLGKIPTVENSIFHMAAWSGSACHDGKEWTYIGVDGPDSNALLKIKYGEAYTDTYHSYANNILRTQESACNCIGGNCYLMITDGSASGISECRFLKIREGRIIKEIFPTGRVKHTEECTCGFASNKTIECACRDNSYTAKRPFVKLNVETDTAEIRLMCTETYLDTPRPDDGSITGPCESNGDKGSGGIKGGFVHQRMASKIGRWYSRTMSKTKRMGMGLYVKYDGDPWTDSDALALSGVMVSMEEPGWYSFGFEIKDKKCDVPCIGIEMVHDGGKETWHSAATAIYCLMGSGQLLWDTVTGVDMAL |
| B/New York/1040/2001 | 2001 | LPSTIQTLTLFLTSGGVLLSLYVSASLSYLLYSDILLKFSPTEITAPTMPLDCANASNVQAVNRSATKGMTLLLPEPEWTYPRLSCPGSTFQKALLISPHRFGETKGNSAPLIIREPFIACGPKECKHFALTHYAAQPGGYYNGTREDRNKLRHLISVKLGKIPTVENSIFHMAAWSGSACHDGKEWTYIGVDGPDSNALLKIKYGEAYTDTYHSYANNILRTQESACNCIGGNCYLMITDGSASGISECRFLKIREGRIIKEIFPTGRVEHTEECTCGFASNKTIECACRDNSYTAKRPFVKLNVETDTAEIRLMCTETYLDTPRPDDGSITGPCESNGGKGSGGIKGGFVHQRMESKIGRWYSRTMSKTKRMGMGLYVKYDGDPWTDSDALALSGVMVSMEEPGWYSFGFEIKDKKCDVPCIGIEMVHDGGKETWHSAATAIYCLMGSGQLLWDTVTGVNMAL |
| B/New York/1041/2001 | 2001 | LPSTIQTLTLFLTSGGVLLSLYVSASLSYLLYSDILLKFSPTEITAPTMPLDCANASNVQAVNRSATKGVTLLLPEPEWTYPRLSCPGSIFQKALLISPHRFGETKGNSAPLIIREPFIACGPKECKHFALTHYAAQPGGYYNGTRGDRNKLRHLISVKLGKIPTVENSIFHMAAWSGSACHDGKEWTYIGVDGPDSNALLKIKYGEAYTDTYHSYANNILRTQESACNCIGGNCYLMITDGSASGISECRFLKIREGRIIKEIFPTGRVKHTEECTCGFASNKTIECACRDNSYTAKRPFVKLNVETDTAEIRLMCTETYLDTPRPDDGSITGPCESNGDKGSGGIKGGFVHQRMASKIGRWYSRTMSKTKRMGMGLYVKYDGDPWTDSDALALSGVMVSMEEPGWYSFGFEIKDKKCDVPCIGIEMVHDGGKETWHSAATAIYCLMGSGQLLWDTVTGVDMAL |
| B/New York/1043/2001 | 2001 | LPSTIQTLTLFLTSGGVLLSLYVSASLSYLLYSDILLKFSPTEITAPTMPLDCANASNVQAVNRSATKEVTLLLPEPEWTYPRLSCPGSTFQKALLISPHRFGETKGNSAPLIIREPFIACGPKECKHFALTHYAAQPGGYYNGTRGDRNKLRHLISVKLGKIPTVENSIFHMAAWSGSACHDGKEWTYIGVDGPDSNALLKIKYGEAYTDTYHSYANNILRTQESACNCIGGNCYLMITDGSASGISECRFLKIREGRIIKEIFPTGRVKHTEECTCGFASNKTIECACRDNSYTAKRPFVKLNVETDTAEIRLMCTETYLDTPRPDDGSITGPCESNGDKGSGGIKGGFVHQRMASKIGRWYSRTMSKTKRMGMGLYVKYDGDPWTDSDALALSGVMVSMEEPGWYSFGFEIKDKKCDVPCIGIEMVHDGGKETWHSAATAIYCLMGSGQLLWDTVTGVDMAL |
| B/New York/1044/2001 | 2001 | LPSTIQTLTLFLTSGGVLLSLYVSASLSYLLYSDILLKFSPTEITAPTMPLDCANASNVQAVNRSATKGMTLLLPEPEWTYPRLSCPGSTFQKALLISPHRFGETKGNSAPLIIREPFIACGPKECKHFALTHYAAQPGGYYNGTREDRNKLRHLISVKLGKIPTVENSIFHMAAWSGSACHDGKEWTYIGVDGPDSNALLKIKYGEAYTDTYHSYANNILRTQESACNCIGGNCYLMITDGSASGISECRFLKIREGRIIKEIFPTGRVEHTEECTCGFASNKTIECACRDNSYTAKRPFVKLNVETDTAEIRLMCTETYLDTPRPDDGSITGPCESNGGKGSGGIKGGFVHQRMESKIGRWYSRTMSKTKRMGMGLYVKYDGDPWTDSDALALSGVMVSMEEPGWYSFGFEIKDKKCDVPCIGIEMVHDGGKETWHSAATAIYCLMGSGQLLWDTVTGVNMAL |
| B/New York/1047/2001 | 2001 | LPSTIQTLTLFLTSGGVLLSLYVSASLSYLLYSDILLKFSPTEITAPTMPLDCANASNVQAVNRSATKGVTLLLPEPEWTYPRLSCPGSTFQKALLISPHRFGETKGNSAPLIIREPFIACGPKECKHFALTHYAAQPGGYYNGTRGDRNKLRHLISVKLGKIPTVENSIFHMAAWSGSACHDGKEWTYIGVDGPDSNALLKIKYGEAYTDTYHSYANNILRTQESACNCIGGNCYLMITDGSASGISECRFLKIREGRIIKEIFPTGRVKHTEECTCGFASNKTIECACRDNSYTAKRPFVKLNVETDTAEIRLMCTETYLDTPRPDDGSITGPCESNGDKGSGGIKGGFVHQRMASKIGRWYSRTMSKTKRMGMGLYVKYDGDPWTDSDALALSGVMVSMEEPGWYSFGFEIKDKKCDVPCIGIEMVHDGGKETWHSAATAIYCLMGSGQLLWDTVTGVDMAL |
| B/South Carolina/NHRC0001/2001 | 2001 | LPSTIQTLTLFLTSGGVLLSLYVSASLSYLLYSDILLKFSPTEITAPTMPLDCANASNVQAVNRSATKGVTLLLPEPEWTYPRLSCPGSIFQKALLISPHRFGETKGNSAPLIIREPFIACGPKECKHFALTHYAAQPGGYYNGTRGDRNKLRHLISVKLGKIPTVENSIFHMAAWSGSACHDGKEWTYIGVDGPDSNALLKIKYGEAYTDTYHSYANNILRTQESACNCIGGNCYLMITDGSASGISECRFLKIREGRIIKEIFPTGRVKHTEECTCGFASNKTIECACRDNSYTAKRPFVKLNVETDTAEIRLMCTETYLDTPRPDDGSITGPCESNGDKGSGGIKGGFVHQRMASKIGRWYSRTMSKTKRMGMGLYVKYDGDPWTDSDALALSGVMVSMEEPGWYSFGFEIKDKKCDVPCIGIEMVHDGGKETWHSAATAIYCLMGSGQLLWDTVTGVDMAL |
| B/South Carolina/NHRC0002/2001 | 2001 | LPSTIQTLTLFLTSGGVLLSLYVSASLSYLLYSDILLKFSPTEITAPTMPLDCANASNVQAVNRSATKGVTLLLPEPEWTYPRLSCPGSTFQKALLISPHRFGETKGNSAPLIIREPFIACGPKECKHFALTHYAAQPGGYYNGTRGDRNKLRHLISVKLGKIPTVENSIFHMAAWSGSACHDGKEWTYIGVDGPDSNALLKIKYGEAYTDTYHSYANNILRTQESACNCIGGNCYLMITDGSASGISECRFLKIREGRIIKEIFPTGRVKHTEECTCGFASNKTIECACRDNSYTAKRPFVKLNVETDTAEIRLMCTETYLDTPRPDDGSITGPCESNGDKGSGGIKGGFVHQRMASKIGRWYSRTMSKTKRMGMGLYVKYDGDPWTDSDALALSGVMVSMEEPGWYSFGFEIKDKKCDVPCIGIEMVHDGGKETWHSAATAIYCLMGSGQLLWDTVTGVDMAL |
| B/Taiwan/1484/2001 | 2001 | LPSTIQTLTLFLTSGGALLSLYVSALLSYLLYSDILLKFSPTKVIAPTMSLDYTNASNVQAVNHSATEEMTFLLPEPEWTYPRLSCQGSTFQKALLISPHRFGEAKGNSAPLIIREPFIACGPKECKHFALTHYAAQPGGYYNGTREDRNKLRHLISVNLGKIPTVENSIFHMAAWSGSACHDGREWTYIGVDGPDSNALIKIKYGEAYTDTYHSYANNILRTQESACNCIGGDCYLMITDGSASGISRCRFLKIREGRIIKEIFPTGRVEHTEECTCGFASNKTIECACRDNSYTAKRPFVKLNVETDTAEIRLMCTETYLDTPRPDDGSITGPCESNGDKGNGGIKGGFVHQRMASRIGRWYSRTMSKTKRMGMELYVKYDGDPWTDSDALAPSGVMVSMEEPGWYSFGFEIKDKKCDVPCIGIEMVHDGGKKTWHSAATAIYCLMGSGQLLWDTVTGVDMAL |
| B/Taiwan/634/2001 | 2001 | LPSTIQTLTLFLTSGGVLLSLYVSASLSYLLYSDILLKFSTTEITAPTMPLDCANASNVQAVNRSATKGVTLLLPEPEWTYPRLSCPGSTFQKALLISPHRFGETKGNSAPLIIREPFIACGPKECKHFALTHYAAQPGGYYNGTREDRNKLRHLVSVKLGKIPTVENSIFHMAAWSGSACHDGKEWTYIGVDGPDSNALLKIKYGEAYTDTYHSYANNILRTQESACNCIGGNCYLMITDGSASGISECRFLKIREGRIIKEIFPTGRVKHTEECTCGFASNKTIECACRDNSYTAKRPFVKLNVETDTAEIRLMCTETYLDTPRPDDGSITGPCESNGDKGSGGIKGGFVHQRMASKIGRWYSRTMSKTKRMGMGLYVKYDGDPWTDSDALALSGVMVSMEEPGWYSFGFEIKDKKCDVPCIGIEMVHDGGKETWHSAATAIYCLMGSGQLLWDTVTGVDMAL |
| B/Temple/B1181/2001 | 2001 | LPSTIQTLTLFLTSGGVLLSLYVSASLSYLLYSDILLKFSPTEITAPTMPLDCANASNVQAVNRSATKGVTLLLPEPEWTYPRLSCPGSTFQKALLISPHRFGETKGNSAPLIIREPFIACGPKECKHFALTHYAAQPGGYYNGTRGDRNKLRHLISVKLGKIPTVENSIFHMAAWSGSACHDGKEWTYIGVDGPDSNALLKIKYGEAYTDTYHSYANNILRTQESACNCIGGNCYLMITDGSASGISECRFLKIREGRIIKEIFPTGRVKHTEECTCGFASNKTIECACRDNSYTAKRPFVKLNVETDTAEIRLMCTETYLDTPRPDDGSITGPCESNGDKGSGGIKGGFVHQRMASKIGRWYSRTMSKTKRMGMGLYVKYDGDPWTDSDALALSGVMVSMEEPGWYSFGFEIKDKKCDVPCIGIEMVHDGGKETWHSAATAIYCLMGSGQLLWDTVTGVDMAL |
| B/Temple/B1188/2001 | 2001 | LPSTIQTLTLFLTSGGVLLSLYVSASLSYLLYSDILLKFSPTEITAPTMPLDCANASNVQAVNRSATKGVTLLLPEPEWTYPRLSCPGSTFQKALLISPHRFGETKGNSAPLIIREPFIACGPKECKHFALTHYAAQPGGYYNGTRGDRNKLRHLISVKLGKIPTVENSIFHMAAWSGSACHDGKEWTYIGVDGPDSNALLKIKYGEAYTDTYHSYANNILRTQESACNCIGGNCYLMITDGSASGISECRFLKIREGRIIKEIFPTGRVKHTEECTCGFASNKTIECACRDNSYTAKRPFVKLNVETDTAEIRLMCTETYLDTPRPDDGSITGLCESNGDKGSGGIKGGFVHQRMASKIGRWYSRTMSKTKRMGMGLYVKYDGDPWTDSDALALSGVMVSMEEPGWYSFGFEIKDKKCDVPCIGIEMVHDGGKETWHSAATAIYCLMGSGQLLWDTVTGVDMAL |
| B/Temple/B1190/2001 | 2001 | LPSTIQTLTLFLTSGGVLLSLYVSASLSYLLYSDILLKFSPTEITAPTMPLDCANASNVQAVNRSATKGVTLLLPEPEWTYPRLSCPGSTFQKALLISPHRFGETKGNSAPLIIREPFIACGPKECKHFALTHYAAQPGGYYNGTRGDRNKLRHLISVKLGKIPTVENSIFHMAAWSGSACHDGKEWTYIGVDGPDSNALLKIKYGEAYTDTYHSYANNILRTQESACNCIGGNCYLMITDGSASGISECRFLKIREGRIIKEIFPTGRVKHTEECTCGFASNKTIECACRDNSYTAKRPFVKLNVETDTAEIRLMCTETYLDTPRPDDGSITGPCESNGDKGSGGIKGGFVHQRMTSKIGRWYSRTMSKTKRMGMGLYVKYDGDPWTDSDALALSGVMVSMEEPGWYSFGFEIKDKKCDVPCIGIEMVHDGGKETWHSAATAIYCLMGSGQLLWDTVTGVDMAL |
| B/Wuhan/2/2001 | 2001 | LPSTIQTLTLFLTSGGVLLSLYVSASLSYLLYSDILLKFSQTEITAPTMPLDCANASNVQAVNRSATKGVTLLLPEPEWTYPRLSCPGSTFQKALLISPHRFGETKGNSAPLIIREPFIACGPKECKHFALTHYAAQPGGYYNGTREDRNKLRHLISVKLGKIPTVENSIFHMAAWSGSACHDGREWTYIGVDGPDSNALLKIKYGEAYTDTYHSYAKNILRTQESACNCIGGDCYLMITDGSASGISECRFLKIREGRIIKEIFPTGRVKHTEECTCGFASNKTIECACRDNSYTAKRPFVKLNVETDTAEIRLMCTETYLDTPRPNDGSITGPCESNGDKGSGGIKGGFVHQRMASKIGRWYSRTMSKTKRMGMGLYVKYDGDPWTDSEALALSGVMVSMEEPGWYSFGFEIKDKKCDVPCIGIEMVHDGGKTTWHSAATAIYCLMGSGQLLWDTVTGVDMAL |
| B/Yamagata/K320/2001 | 2001 | LPSTIQTLTLFLTSGGVLLSLYVSASLSYLLYSGILLKFSPTEITAPTMPLNCANASNVQAVNRSATKGVTLLLPEPEWTYPRLSCPGSTFQKALLISPHRFGETKGNSAPLIIREPFIACGPKECKHFALTHYAAQPGGYYNGTREDRNKLRHLISVKLGKIPTVENSIFHMAAWSGSACHDGREWTYIGVDGPDSNALLKIKYGEAYTDTYHSYANNILRTQESACNCIGGDCYLMITDGSASGISECRFLKIREGRIIKEIFPTGRVEHTEECTCGFASNKTIECACRDNSYTAKRPFVKLNVETDTAEIRLMCTETYLDTPRPDDGSITGPCESHGDKGSGGIKGGFVHQRMASKIGRWYSRTMSKTKRMGMGLYVKYDGDPWIDSGALTLSGVMVSMEEPGWYSFGFEIKDKKCDVPCIGIEMVHDGGKKTWHSAATAIYCLMGSGQLLWDTVTGVDMAL |
| B/Yamagata/K461/2001 | 2001 | LPSTIQTLTLFLTSGGVLLSLYVSASLSYLLYSDILLKFSPTEITAPTMPLDCANASNVQAVNRSATKGVTLLLPEPEWTYPRLSCPGSTFQKALLISPHRFGETKGNSAPLIIREPFIACGPKECKHFALTHYAAQPGGYYNGTRGDRNKLRHLISVKLGKIPTVENSIFHMAAWSGSACHDGKEWTYIGVDGPDSNALLKIKYGEAYTDTYHSYANNILRTQESACNCIGGNCYLMITDGSASGISECRFLKIREGRIIKEIFPTGRVKHTEECTCGFASNKTIECACRDNSYTAKRPFVKLNVETDTAEIRLMCTETYLDTPRPDDGSITGPCESNGDKGSGGIKGGFVHQRMASKIGRWYSRTMSKTKRMGMGLYVKYDGDPWTDSDALALSGVMVSMEEPGWYSFGFEIKDKKCDVPCIGIEMVHDGGKETWHSAATAIYCLMGSGQLLWDTVTGVDMAL |
| B/Yamagata/K490/2001 | 2001 | LPSTIQTLTLFLTSGGVLLSLYVSASLSYLLYSDILLKFSQTEITAPTMPLDCANASNVQAVNRSATKGVTLLLPEPEWTYPRLSCPGSTFQKALLISPHRFGETKGNSAPLIIREPFIACGPKECKHFALTHYAAQPGGYYNGTREDRNKLRHLISVKLGKIPTVENSIFHMAAWSGSACHDGREWTYIGVDGPDSNALLKIKYGEAYTDTYHSYAKNILRTQESACNCIGGDCYLMITDGSASGISECRFLKIREGRIIKEIFPTGRVKHTEECTCGFASNKTIECACRDNSYTAKRPFVKLNVETDTAEIKLMCTETYLDTPRPDDGSITGPCESNGDKGSGGIKGGFVHQRMASKIGRWYSRTMSKTKRMGMGLYVKYDGDPWTDSEALALSGVMVSMEEPGWYSFGFEIKDKKCDVPCIGIEMVHDGGKTTWHSAATAIYCLMGSGQLLWDTVTGVDMAL |
| B/Yamagata/K500/2001 | 2001 | LPSTIQTLTLFLTSGGVLLSLYVSASLSYLLYSDILLKFSPTEITAPTMPLDCANASNVQAVNRSATKGVTLLLPEPEWTYPRLSCPGSTFQKALLISPHRFGETKGNSAPLIIREPFIACGPKECKHFALTHYAAQPGGYYNGTRGDRNKLRHLISVKLGKIPTVENSIFHMAAWSGSACHDGKEWTYIGVDGPDSNALLKIKYGEAYTDTYHSYANNILRTQESACNCIGGNCYLMITDGSASGISECRFLKIREGRIIKEIFPTGRVKHTEECTCGFASNKTIECACRDNSYTAKRPFVKLNVETDTAEIRLMCTETYLDTPRPDDGSITGPCESNGDKGSGGIKGGFVHQRMASKIGRWYSRTMSKTKRMGMGLYVKYDGDPWTDSDALALSGVMVSMEEPGWYSFGFEIKDKKCDVPCIGIEMVHDGGKETWHSAATAIYCLMGSGQLLWDTVTGVDMAL |
| B/Yamagata/K501/2001 | 2001 | LPSTIQTLTLFLTSGGVLLSLYVSASLSYLLYSGILLKFSPTEITAPTMPLNCANASNVQAVNRSATKGVTLLLPEPEWTYPRLSCPGSTFQKALLISPHRFGETKGNSAPLIIREPFIACGPKECKHFALTHYAAQPGGYYNGTREDRNKLRHLISVKLGKIPTVENSIFHMAAWSGSACHDGREWTYIGVDGPDSNALLKIKYGEAYTDTYHSYANNILRTQESACNCIGGDCYLMITDGSASGISECRFLKIREGRIIKEIFPTGRVEHTEECTCGFASNKTIECACRDNSYTAKRPFVKLNVETDTAEIRLMCTETYLDTPRPDDGSITGPCESHGDKGSGGIKGGFVHQRMASKIGRWYSRTMSKTKRMGMGLYVKYDGDPWIDSGALTLSGVMVSMEEPGWYSFGFEIKDKKCDVPCIGIEMVHDGGKKTWHSAATAIYCLMGSGQLLWDTVTGVDMAL |
| B/Yamagata/K515/2001 | 2001 | LPSTIQTLTLFLTSGGVLLSLYVSASLSYLLYSDILLKFSQTEITAPTMPLDCANASNVQAVNRSATKGVTLLPPEPEWTYPRLSCPGSTFQKALLISPHRFGETKGNSAPLIIREPFIACGPKECKHFALTHYAAQPGGYYNGTREDRNKLRHLISVKLGKIPTVENSIFHMAAWSGSACHDGREWTYIGVDGPDSNALLKIKYGEAYTDTYHSYAKNILRTQESACNCIGGDCYLMITDGSASGISECRFLKIREGRIIKEIFPTGRVKHTEECTCGFASNKTIECACRDNSYTAKRPFVKLNVETDTAEIRLMCTETYLDTPRPDDGSITGPCESNGNKGSGGIKGGFVHQRMASKIGRWYSRTMSKTKRMGMGLYVKYDGDPWTDSEALALSGVMVSMEEPGWYSFGFEIKDKKCDVPCIGIEMVHDGGKTTWHSAATAIYCLMGSGQLLWDTVTGVDMAL |
| B/Yamagata/K519/2001 | 2001 | LPSTIQTLTLFLTSGGVLLSLYVSASLSYLLYSDILLKFSPTEITAPTMPLDCANASNVQAVNRSATKGVTLLLPEPEWTYPRLSCPGSTFQKALLISPHRFGETKGNSAPLIIREPFIACGPKECKHFALTHYAAQPGGYYNGTRGDRNKLRHLISVKLGKIPTVENSIFHMAAWSGSACHDGKEWTYIGVDGPDSNALLKIKYGEAYTDTYHSYANNILRTQESACNCIGGNCYLMITDGSASGISECRFLKIREGRIIKEIFPTGRVKHTEECTCGFASNKTIECACRDNSYTAKRPFVKLNVETDTAEIRLMCTETYLDTPRPDDGSITGPCESNGDKGSGGIKGGFVHQRMASKIGRWYSRTMSKTKRMGMGLYVKYDGDPWTDSDALALSGVMVSMEEPGWYSFGFEIKDKKCDVPCIGIEMVHDGGKETWHSAATAIYCLMGSGQLLWDTVTGVDMAL |
| B/Yamagata/K520/2001 | 2001 | LPSTIQTLTLFLTSGGVLLSLYVSASLSYLLYSGILLKFSPTEITAPTMPLNCANASNVQAVNRSATKGVTLLLPEPEWTYPRLSCPGSTFQKALLISPHRFGETKGNSAPLIIREPFIACGPKECKHFALTHYAAQPGGYYNGTREDRNKLRHLISVKLGKIPTVENSIFHMAAWSGSACHDGREWTYIGVDGPDSNALLKIKYGEAYTDTYHSYANNILRTQESACNCIGGDCYLMITDGSASGISECRFLKIREGRIIKEIFPTGRVEHTEECTCGFASNKTIECACRDNSYTAKRPFVKLNVETDTAEIRLMCTETYLDTPRPDDGSITGPCESHGDKGSGGIKGGFVHQRMASKIGRWYSRTMSKTKRMGMGLYVKYDGDPWIDSGALTLSGVMVSMEEPGWYSFGFEIKDKKCDVPCIGIEMVHDGGKKTWHSAATAIYCLMGSGQLLWDTVTGVDMAL |
| B/Yamagata/K521/2001 | 2001 | LPSTIQTLTLFLTSGGVLLSLYVSASLSYLLYSDILLKFSPTEITAPTMPLDCANASNVQAVNRSATKGVTLLLPEPEWTYPRLSCPGSTFQKALLISPHRFGETKGNSAPLIIREPFIACGPKECKHFALTHYAAQPGGYYNGTRGDRNKLRHLISVKLGKIPTVENSIFHMAAWSGSACHDGKEWTYIGVDGPDSNALLKIKYGEAYTDTYHSYANNILRTQESACNCIGGNCYLMITDGSASGISECRFLKIREGRIIKEIFPTGRVKHTEECTCGFASNKTIECACRDNSYTAKRPFVKLNVETDTAEIRLMCTETYLDTPRPDDGSITGPCESNGDKGSGGIKGGFVHQRMASKIGRWYSRTMSKTKRMGMGLYVKYDGDPWTDSDALALSGVMVSMEEPGWYSFGFEIKDKKCDVPCIGIEMVHDGGKETWHSAATAIYCLMGSGQLLWDTVTGVDMAL |
| B/Yamagata/K535/2001 | 2001 | LPSTIQTLTLFLTSGGVLLSLYVSASLSYLLYSDILLKFSQTEITAPTMPLDCANASNVQAVNRSATKGVTLLLPEPEWTYPRLSCPGSTFQKALLISPHRFGETKGNSAPLIIREPFIACGPKECKHFALTHYAAQPGGYYNGTREDRNKLRHLISVKLGKIPTVENSIFHMAAWSGSACHDGREWTYIGVDGPDSNALLKIKYGEAYTDTYHSYAKNILRTQESACNCIGGDCYLMITDGSASGISECRFLKIREGRIIKEIFPTGRVKHTEECTCGFASNKTIECACRDNSYTAKRPFVKLNVETDTAEIKLMCTETYLDTPRPDDGSITGPCESNGDKGSGGIKGGFVHQRMASKIGRWYSRTMSKTKRMGMGLYVKYDGDPWTDSEALALSGVMVSMEEPGWYSFGFEIKDKKCDVPCIGIEMVHDGGKTTWHSAATAIYCLMGSGQLLWDTVTGVDMAL |
| B/Auckland/16/2002 | 2002 | LPSTIQTLTLFLTSGGVLLSLYVSASLSYLLYSDILLKFSPTEITAPTMPLDCANASNVQAVNRSATKGVTLLLPEPEWTYPRLSCPGSTFQKALLISPHRFGETKGNSAPLIIREPFIACGPKECKHFALTHYAAQPGGYYNGTRGDRNKLRHLISVKLGKIPTVENSIFHMAAWSGSACHDGKEWTYIGVDGPDNNALLKIKYGEAYTDTYHSYANNILRTQESACNCIGGNCYLMITDGSASGISECRFLKIREGRIIKEIFPTGRVKHTEECTCGFASNKTIECACRDNSYTAKRPFVKLNVETDTAEIRLMCTETYLDTPRPDDGSITGPCESNGDKGSGGIKGGFVHQRMASKIGRWYSRTMSKTKRMGMGLYVKYDGDPWADSDALALSGVMVSMEEPGWYSFGFEIRDKKCDVPCIGIEMVHDGGKETWHSAATAIYCLMGSGQLLWDTVTGVDMAL |
| B/Auckland/35/2002 | 2002 | LPSTIQTLTLFLTSGGVLLSLYVSASLSYLLYSDILLKFSPTEITAPTMPLDCANASNVQAVNRSATKGVTLLLPEPEWTYPRLSCPGSTFQKALLISPHRFGETKGNSAPLIIREPFIACGPKECKHFALTHYAAQPGGYYNGTRGDRNKLRHLISVKLGKIPTVENSIFHMAAWSGSACHDGKEWTYIGVDGPDNNALLKIKYGEAYTDTYHSYANNILRTQESACNCIGGNCYLMITDGSASGISECRFLKIREGRIIKEIFPTGRVKHTEECTCGFASNKTIECACRDNSYTAKRPFVKLNVETDTAEIRLMCTETYLDTPRPDDGSITGPCESNGDKGSGGIKGGFVHQRMASKIGRWYSRTMSKTKRMGMGLYVKYDGDPWADSDALALSGVMVSMEEPGWYSFGFEIRDKKCDVPCIGIEMVHDGGKETWHSAATAIYCLMGSGQLLWDTVTGVDMAL |
| B/Auckland/36/2002 | 2002 | LPSTIQTLTLFLTSGGVLLSLYVSASLSYLLYSDILLKFSPTEITAPTMPLDCANASNVQAVNRSATKGVTFLLPEPEWTYPRLSCPGSTFQKALLISPHRFGETKGNSAPLIIREPFIACGPKECKHFALTHYAAQPGGYYNGTRGDRNKLRHLISVKLGKIPTVENSIFHMAAWSGSACHDGKEWTYIGVDGPDNNALLKIKYGEAYTDTYHSYANNILRTQESACNCIGGNCYLMITDGSASGISECRFLKIREGRIIKEIFPTGRVKHTEECTCGFASNKTIECACRDNSYTAKRPFVKLNVETDTAEIRLMCTETYLDTPRPDDGSITGPCESNGDKGSGGIKGGFVHQRMASKIGRWYSRTMSKTKRMGMGLYVKYDGDPWADSDALALSGVMVSMEEPGWYSFGFEIKDKKCDVPCIGIEMVHDGGKETWHSAATAIYCLMGSGQLLWDTVTGVDMAL |
| B/Brisbane/25/2002 | 2002 | LPSTIQTLTLFLTSGGVLLSLYVSASLSYLLYSDILLKFSPTEITAPTMPLDCANASNVQAVNRSATKGVTFLLPEPEWTYPRLSCPGSTFQKALLISPHRFGETKGNSAPLIIREPFIACGPKECKHFALTHYAAQPGGYYNGTRGDRNKLRHLISVKLGKIPTVENSIFHMAAWSGSACHDGKEWTYIGVDGPDNNALLKIKYGEAYTDTYHSYANNILRTQESACNCIGGNCYLMITDGSASGISECRFLKIREGRIIKEIFPTGRVKHTEECTCGFASNKTIECACRDNSYTAKRPFVKLNVETDTAEIRLMCTETYLDTPRPDDGSITGPCESNGDKGSGGIKGGFVHQRMASKIGRWYSRTMSKTKRMGMGLYVKYDGDPWADSDALALSGVMVSMEEPGWYSFGFEIKDKKCDVPCIGIEMVHDGGKETWHSAATAIYCLMGSGQLLWDTVTGVDMAL |
| B/Brisbane/30/2002 | 2002 | LPSTIQTLTLFLTSGGVLLSLYVSASLSYLLYSDILLKFSPTEITAPTMPLDCANASNVQAVNRSATKGVTLLLPEPEWTYPRLSCPGSTFQKALLISPHRFGETKGNSAPLIIREPFIACGPKECKHFALTHYAAQPGGYYNGTRGDRNKLRHLISVKLGKIPTVENSIFHMAAWSGSACHDGKEWTYIGVDGPDNNALLKIKYGEAYTDTYHSYANNILRTQESACNCIGGNCYLMITDGSASGISECRFLKIREGRIIKEIFPTGRVKHTEECTCGFASNKTIECACRDNSYTAKRPFVKLNVETDTAEIRLMCTETYLDTPRPDDGSITGPCESNGDKGSGGIKGGFVHQRMASKIGRWYSRTMSKTKRMGMGLYVKYDGDPWADSDALALSGVMVSMEEPGWYSFGFEIKDKKCDVPCIGIEMVHDGGKETWHSAATAIYCLMGSGQLLWDTVTGVDMAL |
| B/Brisbane/34/2002 | 2002 | LPSTIQTLTLFLTSGGVLLSLYVSASLSYLLYSDILLKFSPTEITAPTMPLDCANASNVQAVNRSATKGVTLLLPEPEWTYPRLSCPGSTFQKALLISPHRFGETKGNSAPLIIREPFIACGPKECKHFALTHYAAQPGGYYNGTRGDRNKLRHLISVKLGKIPTVENSIFHMAAWSGSACHDGKEWTYIGVDGPDNNALLKIKYGEAYTDTYHSYANNILRTQESACNCIGGNCYLMITDGSASGISECRFLKIREGRIIKEIFPTGRVKHTEECTCGFASNKTIECACRDNSYTAKRPFVKLNVETDTAEIRLMCTETYLDTPRPDDGSITGPCESNGDKGSGGIKGGFVHQRMASKIGRWYSRTMSKTKRMGMGLYVKYDGDPWADSDALALSGVMVSMEEPGWYSFGFEIKDKKCDVPCIGIEMVHDGGKETWHSAATAIYCLMGSGQLLWDTVTGVDMAL |
| B/Brisbane/47/2002 | 2002 | LPSTIQTLTLFLTSGGVLLSLYVSASLSYLLYSDILLKFSPTEITAPTMPLDCANASNVQAVNRSATKGVTLLLPELEWTYPRLSCPGSTFQKALLISPHRFGETKGNSAPLIIREPFIACGPKECKHFALTYYAAQPGGYYNGTRGDRNKLRHLISVKLGKIPTVENSIFHMAAWSGSACHDGKEWTYIGVDGPDNNALLKIKYGEAYTDTYHSYANNILRTQESACNCIGGNCYLMITDGSASGISECRFLKIREGRIIKEIFPTGRVKHTEECTCGFASNKTIECACRDNSYTAKRPFVKLNVETDTAEIRLMCTETYLDTPRPADGSITGPCESNGDKGSGGIKGGFVHQRMASKIGRWYSRTMSKTKRMGMGLYVKYDGDPWADSDALALSGVMVSMEEPGWYSFGFEIKDKKCDVPCIGIEMVHDGGKETWHSAATAIYCLMGSGQLLWDTVTGVDMAL |
| B/Brisbane/52/2002 | 2002 | LPSTIQTLTLFLTSGGVLLSLYVSASLSYLLYSDILLKFSPTEINAPTMPLDCANASNVQAVNRSATKGVTLLLPEPEWTYPRLSCPGSTFQKALLISPHRFGETKGNSAPLIIREPFIACGPKECKHFALTHYAAQPGGYYNGTRGDRNKLRHLISVKLGKIPTVENSIFHMAAWSGSACHDGKEWTYIGVDGPDNNALLKIKYGEAYTDTYHSYANNILRTQESACNCIGGNCYLMITDGSASGISECRFLKIREGRIIKEIFPTGRVKHTEECTCGFASNKTIECACRDNSYTAKRPFVKLNVETDTAEIRLMCTETYLDTPRPDDGSITGPCESNGDKGSGGIKGGFVHQRMASKIGRWYSRTMSKTKRMGMGLYVKYDGDPWADSDALALSGVMVSMEEPGWYSFGFEIKDKKCDVPCIGIEMVHDGGKETWHSAATAIYCLMGSGQLLWDTVTGVDMAL |
| B/Brisbane/59/2002 | 2002 | LPSTIQTLTLFLTSGGVLLSLYVSASLSYLLYSDILLKFSPTEIIAPTMPLDCANASNVQAVNRSATKGVTLLLPEPEWTYPRLSCPGSTFQKALLISPHRFGETKGNSAPLIIREPFIACGPKECKHFALTHYAAQPGGYYNGTRGDRNKLRHLISVKLGKIPTVENSIFHMAAWSGSACHDGKEWTYIGVDGPDNNALLKIKYGEAYTDTYHSYANNILRTQESACNCIGGNCYLMITDGSASGISECRFLKIREGRIIKEIFPTGRVKHTEECTCGFASNKTIECACRDNSYTAKRPFVKLNVETDTAEIRLMCTETYLDTPRPDDGSITGPCESNGDKGSGGIKGGFVHQRMASKIGRWYSRTMSKTKRMGMGLYVKYDGDPWADSDALALSGVMVSMEEPGWYSFGFEIKDKKCDVPCIGIEMVHDGGKETWHSAATAIYCLMGSGQLLWDTVTGVDMAL |
| B/Brisbane/72/2002 | 2002 | LPSTIQTLTLFLTSGGVLLSLYVSVSLSYLLYSDILLKFSPTEITAQTMPLDFANASNVQAVNRSATKGVTLLLPEPEWTYPRLSCPGSTFQKALLISPHRFGETKGNSAPLIIREPFIACGPKECKHFALTHYAAQPGGYYNGTRGDRNKLRHLISVKLGKIPTVENSIFHMAAWSGSACHDGKEWTYIGVDGPDNNALLKIKYGEAYTDTYHSYANNILRTQESACNCIGGNCYLMITDGSASGISECRFLKIREGRIIKEIFPTGRVKHTEECTCGFASNKTIECACRDNSYTAKRPFVKLNVETDTAEIRLMCTETYLDTPRPDDGSITGPCESNGDKGSGGIKGGFVHQRMASKIGRWYSRTMSKTKRMGMGLYVKYDGDPWADSDALALSGVMVSMEEPGWYSFGFEIRDKKCDVPCIGIEMVHDGGKETWHSAATAIYCLMGSGQLLWDTVTGVDMAL |
| B/Christchurch/133/2002 | 2002 | LPSTIQTLTLFLTSGGVLLSLYVSASLSYLLYSDILLKFSPTEITAPTMPLDCANASNVQAVNRSATKGVTLLLPEPEWTYPRLSCPGSTFQKALLISPHRFGETKGNSAPLIIREPFIACGPKECKHFALTHYAAQPGGYYNGKRGDRNKLRHLISVKLGKIPTVENSIFHMAAWSGSACHDGKEWTYIGVDGPDNNALLKIKYGEAYTDTYHSYANNILRTQESACNCIGGNCYLMITDGSASGISECRFLKIREGRIIKEIFPTGRVKHTEECTCGFASNKTIECACRDNSYTAKRPFVKLNVETDTAEIRLMCTETYLDTPRPDDGSITGPCESNGDKGSGGIKGGFVHQRMASKIGRWYSRTMSKTKRMGMGLYVKYDGDPWADSDALALSGVMVSMEEPGWYSFGFEIRDKKCDVPCIGIEMVHDGGKETWHSAATAIYCLMGSGQLLWDTVTGVDMAL |
| B/Christchurch/84/2002 | 2002 | LPSTIQTLTLFLTSGGVLLSLYVSASLSYLLYSDILLKFSPTEITAPTMPLDCANASNVQAVNRSATKGVTLLLPEPEWTYPRLSCPGSTFQKALLISPHRFGETKGNSAPLIIREPFIACGPKECKHFALTHYAAQPGGYYNGTRGDRNKLRHLISVKLGKIPTVENSIFHMAAWSGSACHDGKEWTYIGVDGPDNNALLKIKYGEAYTDTYHSYANNILRTQESACNCIGGNCYLMITDGSASGISECRFLKIREGRIIKEIFPTGRVKHTEECTCGFASNKTIECACRDNSYTAKRPFVKLNVETDTAEIRLMCTETYLDTPRPDDGSITGPCESNGDKGSGGIKGGFVHQRMASKIGRWYSRTMSKTKRMGMGLYVKYDGDPWADSDALALSGVMVSMEEPGWYSFGFEIRDKKCDVPCIGIEMVHDGGKETWHSAATAIYCLMGSGQLLWDTVTGVDMAL |
| B/Christchurch/90/2002 | 2002 | LPSTIQTLTLFLTSGGVLLSLYVSASLSYLLYSDILLKFSPTEITAPTMPLDCANASNVQAVNRSATKGVTLLLPEPEWTYPRLSCPGSTFQKALLISPHRFGETKGNSAPLIIREPFIACGPKECKHFALTHYAAQPGGYYNGTRGDRNKLRHLISVKLGKIPTVENSIFHMAAWSGSACHDGKEWTYIGVDGSDNNALLKIKYGEAYTDTYHSYANNILRTQESACNCIGGNCYLMITDGSASGISECRFLKIREGRIIKEIFPTGRVKHTEECTCGFASNKTIECACRDNSYTAKRPFVKLNVETDTAEIRLMCTETYLDTPRPDDGSITGPCESNGDKGSGGIKGGFVHQRMASKIGRWYSRTMSKTKRMGMGLYVKYDGDPWADSDALALSGIMVSMEEPGWYSFGFEIKDKKCDVPCIGIEMVHDGGKETWHSAATAIYCLMGSGQLLWDTVTGVDMAL |
| B/Dunedin/1/2002 | 2002 | LPSTIQTLTLFLTSGGVLLSLYVSASLSYLLYSDILLKFSPTEITAPTMPLDCANASNVQAVNRSATKGVTLLLPEPEWTYPRLSCPGSTFQKALLISPHRFGETKGNSAPLIIREPFIACGPKECKHFALTHYAAQPGGYYNGTRGDRNKLRHLISVKLGKIPTVENSIFHMAAWSGSACHDGKEWTYIGVDGPDNNALLKIKYGEAYTDTYHSYANNILRTQESACNCIGGNCYLMITDGSASGISECRFLKIREGRIIKEIFPTGRVKHTEECTCGFASNKTIECACRDNSYTAKRPFVKLNVETDTAEIRLMCTETYLDTPRPDDGSITGPCESNGDKGSGGIKGGFVHQRMASKIGRWYSRTMSKTKRMGMGLYVKYDGDPWADSDALALSGVMVSMEEPGWYSFGFEIRDKKCDVPCIGIEMVHDGGKETWHSAATAIYCLMGSGQLLWDTVTGVDMAL |
| B/Hong Kong/167/2002 | 2002 | LPSTIQTLTLFLTSGGVLLSLYVSALLSYLLYSDILLKFSPTKIIAPTTSLDSANASNFQAVNHSATKEMTFLLPEPEWTYPRLSCQGSTFQKALLISPHRFGEAKGNSAPLIIREPFIACGPKECKHFALTHYAAQPGGYYNGTREDRNKLRHLISVNLGKIPTVENSIFHMAAWSGSACHDGREWTYIGVDGPDSNALIKIKYGEAYTDTYHSYANNILRTQESACNCIGGDCYLMITDGSASGISKCRFLKIREGRIIKEIFPTGRVEHTEECTCGFASNKTIECACRDNSYTAKRPFVKLNVETDTAEIRLMCTETYLDTPRPDDGSITGPCESNGDKGSGGIKGGFVHQRMASKIGRWYSRTMSKTKRMGMELYVKYDGDPWTDSDALAPSGVMVSIEEPGWYSFGFEIKDKKCDVPCIGIEMVHDGGKTTWHSAATAIYCLMGSGQLLWDTVTGVDMAL |
| B/Houston/B69/2002 | 2002 | LPSTIQTLTLFLTSGGVLLSLYVSASLSYLLYSDILLKFSPTEITAPTMPLDCANASNVQAVNHSATKGVTLLLPEPEWTYPRLSCPGSTFQKALLISPHRFGETKGNSAPLIIREPFIACGPKECKHFALTHYAAQPGGYYNGTRGDRNKLRHLISVKLGKIPTVENSIFHMAAWSGSACHDGKEWTYIGVDGPDSNALLKIKYGEAYTDTYHSYANNILRTQESACNCIGGNCYLMITDGSASGISECRFLKIREGRIIKEIFPTGRVKHTEECTCGFASNKTIECACRDNSYTAKRPFVKLNVETDTAEIRLMCTETYLDTPRPDDGSITGPCESNGDKGSGGIKGGFVHQRMASKIGRWYSRTMSKTKRMGMGLYVKYDGDPWTDSDALAFSGVMVSMEEPGWYSFGFEIKDKKCDVPCIGIEMVHDGGKETWHSAATAIYCLMGSGQLLWDTVTGVDMAL |
| B/Houston/B71/2002 | 2002 | LPSTIQTLTLFLTSGGVLLSLYVSASLSYLLYSDILLKFSPTEITAPTMPLDCANASNVQAVNRSATKGVKLLLPEPEWTYPRLSCPGSTFQKALLISPHRFGETKGNSAPLIIREPFIACGPKECKHFALTHYAAQPGGYYNGTRGDRNKLRHLISVKLGKIPTVENSIFHMAAWSGSACHDGKEWTYIGVDGPDNNALLKIKYGEAYTDTYHSYANNILRTQESACNCIGGNCYLMITDGSASGVSECRFLKIREGRIIKEIFPTGRVKHTEECTCGFASNKTIECACRDNSYTAKRPFVKLNVETDTAEIRLMCTETYLDTPRPDDGSITGPCESNGDKGSGGIKGGFVHQRMASKIGRWYSRTMSKTKRMGMGLYVKYDGDPWADSDALALSGVMVSMEEPGWYSFGFEIKDKKCDVPCIGIEMVHDGGKETWHSAATAIYCLMGSGQLLWDTVTGVDMAL |
| B/Houston/B75/2002 | 2002 | LPSTIQTLTLFLTSGGVLLSLYVSASLSYLLYSDILLKFSPTEITAPTMPLDCANASNVQAVNRSATKGVTLLLPEPEWTYPRLSCPGSTFQKALLISPHRFGETKGNSAPLIIREPFIACGPKECKHFALTHYAAQPGGYYNGTRGDRNKLRHLISVKLGKIPTVENSIFHMAAWSGSACHDGKEWTYIGVDGPDNNALLKIKYGEAYTDTYHSYANNILRTQESACNCIGGNCYLMITDGSASGISECRFLKIREGRIIKEIFPTGRVKHTEECTCGFASNKTIECACRDNSYTAKRPFVKLNVETDTAEIRLMCTETYLDTPRPDDGSITGPCESNGDKGSGGIKGGFVHQRMASKIGRWYSRTMSKTKRMGMGLYVKYDGDPWADSDALALSGVMVSMEEPGWYSFGFEIKDKKCDVPCIGIEMVHDGGKETWHSAATAIYCLMGSGQLLWDTVTGVDMAL |
| B/Maryland/1/2002 | 2002 | LPSTIQTLTLFLTSGGVLLSLYVSASLSYLLYSDILLKFSPTEITAPTMPLDCANASNVQAVNRSATKGVTLLLPEPEWTYPRLSCPGSTFQKALLISPHRFGETKGNSAPLIIREPFIACGPKECKHFALTHYAAQPGGYYNGTRGDRNKLRHLISVKLGKIPTVENSIFHMAAWSGSACHDGKEWTYIGVDGPDNNALLKIKYGEAYTDTYHSYANNILRTQESACNCIGGNCYLMITDGSASGISECRFLKIREGRIIKEIFPTGRVKHTEECTCGFASNKTIECACRDNSYTAKRPFVKLNVETDTAEIRLMCTETYLDTPRPDDGSITGPCESNGDKGSGGIKGGFVHQRMASKIGRWYSRTMSKTKRMGMGLYVKYDGDPWADSDALALSGVMVSMEEPGWYSFGFEIRDKKCDVPCIGIEMVHDGGKETWHSAATAIYCLMGSGQLLWDTVTGVDMAL |
| B/New York/1051/2002 | 2002 | LPSTIQTLTLFLTSGGVLLSLYVSASLSYLLYSDILLKFSPTEITAPTMPLDCANASNVQAVNRSATKGVTLLLPEPEWTYPRLSCPGSTFQKALLISPHRFGETKGNSAPLIIREPFIACGPKECKHFALTHYAAQPGGYYNGTRGDRNKLRHLISVKLGKIPTVENSIFHMAAWSGSACHDGKEWTYIGVDGPDNNALLKIKYGEAYTDTYHSYANNILRTQESACNCIGGNCYLMITDGSASGISECRFLKIREGRIIKEIFPTGRVKHTEECTCGFASNKTIECACRDNSYTAKRPFVKLNVETDTAEIRLMCTETYLDTPRPDDGSITGPCESNGDKGSGGIKGGFVHQRMASKIGRWYSRTMSKTKRMGMGLYVKYDGDPWADSDALALSGVMVSMEEPGWYSFGFEIRDKKCDVPCIGIEMVHDGGKETWHSAATAIYCLMGSGQLLWDTVTGVDMAL |
| B/New York/1052/2002 | 2002 | LPSTIQTLTLLLTSGGVLLSLYVSASLSYLLYSDILLKFSPTEITAPTMPLDCANASNVQAVNRSATKGVTLLLPEPEWTYPRLSCPGSTFQKALLISPHRFGETKGNSAPLIIREPFIACGPKECKHFALTHYAAQPGGYYNGTRGDRNKLRHLISVKLGKIPTVENSIFHMAAWSGSACHDGKEWTYIGVDGPDSNALLKIKYGEAYTDTYHSYANNILRTQESACNCIGGNCYLMITDGSASGISECRFLKIREGRIIKEIFPTGRVKHTEECTCGFASNKTIECACRDNSYTAKRPFVKLNVETDTAEIRLMCTETYLDTPRPDDGSITGPCESNGDKGSGGIKGGFVHQRMASKIGRWYSRTMSKTKRMGMGLYVKYDGDPWTDSDALALSGVMVSMEEPGWYSFGFEIKDKKCDVPCIGIEMVHDGGKETWHSAATAIYCLMGSGQLLWDTVTGVDMAL |
| B/Sydney/12/2002 | 2002 | LPSTIQTLTLFLTSGGVLLSLYVSASLSYLLYSDILLKFSPTEITAPTMPLDCANASNVQAVNRSATKGVTLLLPEPEWTYPRLSCPGSTFQKALLISPHRFGETKGNSAPLIIREPFIACGPKECKHFALTHYAAQPGGYYNGTRGDRNKLRHLISVKLGKIPTVENSIFHMAAWSGSACHDGKEWTYIGVDGPDNNALLKIKYGEAYTDTYHSYANNILRTQESACNCIGGNCYLMITDGSASGISECRFLKIREGRIIKEIFPTGRVKHTEECTCGFASNKTIECACRDNSYTAKRPFVKLNVETDTAEIRLMCTETYLDTPRPDDGSITGPCESNGDKGSGGIKGGFVHQRMASKIGRWYSRTMSKTKRMGMGLYVKYDGDPWADSDALALSGVMVSMEEPGWYSFGFEIKDKKCDVPCIGIEMVHDGGKETWHSAATAIYCLMGSGQLLWDTVTGVDMAL |
| B/Sydney/200/2002 | 2002 | LPSTIQTLTLFLTSGGVLLSLYVSASLSYLLYSDILLKFTPTEITVPTMPLDCANASNVQAVNRSATKGVTLLLPEPEWTYPRLSCPGSTFQKALLISPHRFGETKGNSAPLIIREPFIACGPKECKHFALTHYAAQPGGYYNGTREDRNKLRHLISVKLGKIPTVENSIFHMAAWSGSACHDGKEWTYIGVDGPDSNALLKIKYGEAYTDTYHSYANNILRTQESACNCIGGNCYLMITDGSASGISECRFLKIREGRIIKEIFPTGRVKHTEECTCGFASNKTIECACRDNSYTAKRPFVKLNVETDTAEIRLMCTETYLDTPRPDDGSITGPCESNGNKGSGGIKGGFVHQRMASKIGRWYSRTMSKTKRMGMGLYVKYDGDPWTDSDALALSGVMVSMEEPGWYSFGFEIKDKKCDVPCIGIEMVHDGGKETWHSAATAIYCLMGSGQLLWDTVTGVDMAL |
| B/Sydney/213/2002 | 2002 | LPSTIQTLTLFLTSGGVLLSLYVSASLSYLLYSDILLKFSPTEITAPTMPLDCANASNVQAVNRSATKGVTLLLPEPEWTYPRLSCPGSTFQKALLISPHRFGETKGNSAPLIIREPFIACGPKECKHFALTHYAAQPGGYYNGTRGDRNKLRHLISVKLGKIPTVENSIFHMAAWSGSACHDGKEWTYIGVDGPDNNALLKIKYGEAYTDTYHSYANNILRTQESACNCIGGNCYLMITDGSASGISECRFLKIREGRIIKEIFPTGRVKHTEECTCGFASNKTIECACRDNSYTAKRPFVKLNVETDTAEIRLMCTETYLDTPRPDDGSITGPCESNGDKGSGGIKGGFVHQRMASKIGRWYSRTMSKTKRMGMGLYVKYDGDPWADSDALALSGVMVSMEEPGWYSFGFEIKDKKCDVPCIGIEMVHDGGKETWHSAATAIYCLMGSGQLLWDTVTGVDMAL |
| B/Sydney/3/2002 | 2002 | LPSTIQTLTLFLTSGGVLLSLYVSASLSYLLYSDILLKFTPTEITVPTMPLDCANASNVQAVNRSATKGVTLLLPEPEWTYPRLSCPGSTFQKALLISPHRFGETKGNSAPLIIREPFIACGPKECKHFALTHYAAQPGGYYNGTREDRNKLRHLISVKLGKIPTVENSIFHMAAWSGSACHDGKEWTYIGVDGPDSNALLKIKYGEAYTDTYHSYANNILRTQESACNCIGGNCYLMITDGSASGISECRFLKIREGRIIKEIFPTGRVKHTEECTCGFASNKTIECACRDNSYTAKRPFVKLNVETDTAEIRLMCTETYLDTPRPDDGSITGPCESNGNKGSGGIKGGFVHQRMASKIGRWYSRTMSKTKRMGMGLYVKYDGDPWTDSDALALSGVMVSMEEPGWYSFGFEIKDKKCDVPCIGIEMVHDGGKETWHSAATAIYCLMGSGQLLWDTVTGVDMAL |
| B/Sydney/64/2002 | 2002 | LPSTIQTLTLFLTSGGVLLSLYVSASLSYLLYSDILLKFSPTEIIAPTMPLDCANASNVQAVNRSATKGVTLLLPEPEWTYPRLSCPGSTFQKALLISPHRFGETKGNSAPLIIREPFIACGPKECKHFALTHYAAQPGGYYNGTRGDRNKLRHLISVKLGKIPTVENSIFHMAAWSGSACHDGKEWTYIGVDGPDNNALLKIKYGEAYTDTYHSYANNILRTQESACNCIGGNCYLMITDGSASGISECRFLKIREGRIIKEIFPTGRVKHTEECTCGFASNKTIECACRDNSYTAKRPFVKLNVETDTAEIRLMCTETYLDTPRPDDGSITGPCESNGDKGSGGIKGGFVHQRMASKIGRWYSRTMSKTKRMGMGLYVKYDGDPWADSDALALSGVMVSMEEPGWYSFGFEIKDKKCDVPCIGIEMVHDGGKETWHSAATAIYCLMGSGQLLWDTVTGVDMAL |
| B/Sydney/7/2002 | 2002 | LPSTIQTLTLFLTSGGVLLSLYVSASLSYLLYSDILLKFSPTEIIAPTMPLDCANASNVQAVNRSATKGVTLLLPEPEWTYPRLSCPGSTFQKALLISPHRFGETKGNSAPLIIREPFIACGPKECKHFALTHYAAQPGGYYNGTRGDRNKLRHLISVKLGKIPTVENSIFHMAAWSGSACHDGKEWTYIGVDGPDNNALLKIKYGEAYTDTYHSYANNILRTQESACNCIGGNCYLMITDGSASGISECRFLKIREGRIIKEIFPTGRVKHTEECTCGFASNKTIECACRDNSYTAKRPFVKLNVETDTAEIRLMCTETYLDTPRPDDGSITGPCESNGDKGSGGIKGGFVHQRMASKIGRWYSRTMSKTKRMGMGLYVKYDGDPWADSDALALSGVMVSMEEPGWYSFGFEIRDKKCDVPCIGIEMVHDGGKETWHSAATAIYCLMGSGQLLWDTVTGVDMAL |
| B/Sydney/70/2002 | 2002 | LPSTIQTLTLFLTSGGVLLSLYVSASLSYLLYSDILLKFSPTEINAPTMPLDCANASNVQAVNRSATKGVTLLLPEPEWTYPRLSCPGSTFQKALLISPHRFGETKGNSAPLIIREPFIACGPKECKHFALTHYAAQPGGYYNGTRGDRNKLRHLISVKLGKIPTVENSIFHMAAWSGSACHDGKEWTYIGVDGPDNNALLKIKYGEAYTDTYHSYANNILRTQESACNCIGGNCYLMITDGSASGISECRFLKIREGRIIKEIFPTGRVKHTEECTCGFASNKTIECACRDNSYTAKRPFVKLNVETDTAEIRLMCTETYLDTPRPDDGSITGPCESNGDKGSGGIKGGFVHQRMASKIGRWYSRTMSKTKRMGMGLYVKYDGDPWADSDALALSGVMVSMEEPGWYSFGFEIKDKKCDVPCIGIEMVHDGGKETWHSAATAIYCLMGSGQLLLDTVTGVDMAL |
| B/Sydney/72/2002 | 2002 | LPSTIQTLTLFLTSGGVLLSLYVSASLSYLLYSDILLKFSPTEITAPTMPLDCANASNVQAVNRSATKGVTLLLPEPEWTYPRLSCPGSTFQKALLISPHRFGETKGNSAPLIIREPFIACGPKECKHFALTHYAAQPGGYYNGTRGDRNKLRHLISVKLGKIPTVENSIFHMAAWSGSACHDGKEWTYIGVDGPDNNALLKIKYGEAYTDTYHSYANNILRTQESACNCIGGNCYLMITDGSASGISECRFLKIREGRIIKEIFPTGRVKHTEECTCGFASNKTIECACRDNSYTAKRPFVKLNVETDTAEIRLMCTETYLDTPRPDDGSITGPCESNGDNGSGGIKGGFVHQRMASKIGRWYSRTMSKTKRMGMGLYVKYDGDPWADSDALALSGVMVSMEEPGWYSFGFEIKDKKCDVPCIGIEMVHDGGKETWHSAATAIYCLMGSGQLLWDTVTGVDMAL |
| B/Sydney/86/2002 | 2002 | LPSTIQTLTLFLTSGGVLLSLYVSASLSYLLYSDILLKFSPTEITAPTIPLDCANASNIQAVNRSATKGVTLLLPEPEWTYPRLSCPGSTFQKALLISPHRFGETKGNSAPLIIREPFIACGPKECKHFALTHYAAQPGGYYNGTRGDRNKLRHLISVKLGKIPTVENSIFHMAAWSGSACHDGKEWTYIGVDGPDNNALLKIKYGEAYTDTYHSYANNILRTQESACNCIGGNCYLMITDGSASGISECRFLKIREGRIIKEIFPTGRVKHTEECTCGFASNKTIECACRDNSYTAKRPFVKLNVETDTAEIRLMCTETYLDTPRPDDGSITGPCESNGDKGSGGIKGGFVHQRMASKIGRWYSRTMSKTKRMGMGLYVKYDGDPWADSDALALSGVMVSMEEPGWYSFGFEIKDKKCDVPCIGIEMVHDGGKETWHSAATAIYCLMGSGQLLWDTVTGVDMAL |
| B/Tehran/80/02 | 2002 | LPSTIQTLTLFLTSGGVLLSLYVSASLSYLLYSDILLKFSPTEITAPTMPLDCANASNVQAVNRSATKGVTLLLPEPEWTYPRLSCPGSTFQKALLISPHRFGETKGNSAPLIIREPFIACGPKECKHFALTHYAAQPGGYYNGTRGDRNKLRHLISVKLGKIPTVENSIFHMAAWSGSACHDGKEWTYIGVDGPDNNALLKIKYGEAYTDTYHSYANNILRTQESACNCIGGNCYLMITDGSASGISECRFLKIREGRIIKEIFPTGRVKHTEECTCGFASNKTIECACRDNSYTAKRPFVKLNVETDTAEIRLMCTETYLDTPRPDDGSITGPCESNGDKGSGGIKGGFVHQRMASKIGRWYSRTMSKTKRMGMGLYVKYDGDPWADSDALALSGVMVSMEEPGWYSFGFEIKDKKCDVPCIGIEMVHDGGKETWHSAATAIYCLMGSGQLLWDTVTGVDMAL |
| B/Townsville/11/2002 | 2002 | LPSTIQTLTLFLTSGGVLLSLYVSASLSYLLYSDILLKFSPTEITAPTMPLDCANASNVQAVNRSATKGVTFLLPEPEWTYPRLSCPGSTFQKALLISPHRFGETKGNSAPLIIREPFIACGPKECKHFALTHYAAQPGGYYNGTRGDRNKLRHLISVKLGKIPTVENSIFHMAAWSGSACHDGKEWTYIGVDGPDNNALLKIKYGEAYTDTYHSYANNILRTQESACNCIGGNCYLMITDGSASGISECRFLKIREGRIIKEIFPTGRVKHTEECTCGFASNKTIECACRDNSYTAKRPFVKLNVETDTAEIRLMCTETYLDTPRPDDGSITGPCESNGDKGSGGIKGGFVHQRMASKIGRWYSRTMSKTKRMGMGLYVKYDGDPWADSDALALSGVMVSMEEPGWYSFGFEIRDKKCDVPCIGIEMVHDGGKETWHSAATAIYCLMGSGQLLWDTVTGVDMAL |
| B/Ulan Ude/4/02 | 2002 | LPSTIQTLTLFLTSGGVLLSLYVSASLSYLLYSDILLKFSQTEITAPTMPLDCANASNVQAVNRSATKGVTLLLPEPEWTYPRLSCPGSTFQKALLISPHRFPETKGNSAPLIIREPFIACGPKECKHFALTHYAAQPGGYYNGTREDRNKLRHLISVKLGKIPTVENSIFHMAAWSGSACHDGREWTYIGVDGPDSNALLKIKYGEAYTDTYHSYAKNILRTQESACNCIGGDCYLMITDGSASGISECRFLKIREGRIIKEIFPTGRVKHTEECTCGFASNKTIECACRDNSYTAKRPFVKLNVETDTAEIRLMCTETYLDTPRPNDGSITGPCESNGDKGSGGIKGGFVHQRMASKIGRWYSRTMSKTKRMGMGLYVKYDGDPWTDSEALALSGVMVSMEEPGWYSFGFEIKDKKCDVPCIGIEMVHDGGKTTWHSAATAIYCLMGSGQLLWDTVTGVDMAL |
| B/Victoria/108/2002 | 2002 | LPSTIQTLTLFLTSGGVLLSLYVSASLSYLLYSDILLKFSQTEITAPTMPLDCANASNVQAVNRSATKGVTLLLPEPEWTYPRLSCPGSTFQKALLISPHRFGETKGNSAPLIIREPFIACGPKECKHFALTHYAAQPGGYYNGTREDRNKLRHLISVKLGKIPTVENSIFHMAAWSGSACHDGREWTYIGVDGPDSNALLKIKYGEAYTDTYHSYAKNILRTQESACNCIGGDCYLMITDGSASGISECRFLKIREGRIIKEIFPTGRVKHTEECTCGFASNKTIECACRDNSYTAKRPFVKLNVETDTAEIRLMCTETYLDTPRPNDGSITGPCESNGDKGSGGIKGGFVHQRMESKIGRWYSRTMSKTKRMGMGLYVKYDGDPWTDSEALALSGVMVSMEEPGWYSFGFEIKDKKCDVPCIGIEMVHDGGKTTWHSAATAIYCLMGSGQLLWDTVTGVDMTL |
| B/Victoria/119/2002 | 2002 | LPSTIQTLTLFLTSGGVLLSLYVSASLSYLLYSDILLKFSPTEITAPTMPLDCANASNVQAVNRSATKGVTLLLPEPEWTYPRLSCPGSIFQKALLISPHRFGETKGNSAPLIIREPFIACGPKECKHFALTHYAAQPGGYYNGTRGDRNKLRHLISVKLGKIPTVENSIFHMAAWSGSACHDGKEWTYIGVDGPDNNALLKIKYGEAYTDTYHSYANNILRTQESACNCIGGNCYLMITDGSASGISECRFLKIREGRIIKEIFPTGRVKHTEECTCGFASNKTIECACRDNSYTAKRPFVKLNVETDTAEIRLMCTETYLDTPRPDDGSITGPCESNGDKGSGGIKGGFVHQRMASKIGRWYSRTMSKTKRMGMGLYVKYDGDPWADSDALALSGVMVSMEEPGWYSFGFEIRDKKCDVPCIGIEMVHDGGKETWHSAATAIYCLMGSGQLLWDTVTGVDMAL |
| B/Victoria/142/2002 | 2002 | LPSTIQTLTLFLTSGGVLLSLYVSASLSYLLYSDILLKFSQTEITAPTMPLDCANASNVQAANRSATKGVTLLLPEPEWTYPRLSCPGSTFQKALLISPHRFGETKGNSAPLIIREPFIACGPKECKHFALTHYAAQPGGYYNGTREDRNKLRHLISVKLGKIPTVENSIFHMAAWSGSACHDGREWTYIGVDGPDSNALLKIKYGEAYTDTYHSYAKNILRTQESACNCIGGDCYLMITDGSASGISECRFLKIREGRIIKEIFPTGRVKHTEECTCGFASNKTIECACRDNSYTAKRPFVKLNVETDTAEIRLMCTETYLDTPRPNDGSITGPCESDGDKGSGGIKGGFVHQRMASKIGRWYSRTMSKTKRMGMGLYVKYDGDPWTDSEALALSGVMVSMEEPGWYSFGFEIKDKKCDVPCIGIEMVHDGGKTTWHSAATAIYCLMGSGQLLWDTVTGVDMAL |
| B/Victoria/143/2002 | 2002 | LPSTIQTLTLFLTSGGVLLSLYVSASLSYLLYSDILLKFSPTEITAPTMPLDCANASNVQAVNRSATKGVTLLLPEPEWTYPRLSCPGSTFQKALLISPHRFGETKGNSAPLIIREPFIACGPKECKHFALTHYAAQPGGYYSGTRGDRNKLRHLISVKLGKIPTVENSIFHMAAWSGSACHDGKEWTYIGVDGPDNNALLKIKYGEAYTDTYHSYANNILRTQESACNCIGGNCYLMITDGSASGISECRFLKIREGRIIKEIFPTGRVKHTEECTCGFASNKTIECACRDNNYTAKRPFVKLNVETDTAEIRLMCTETYLDTPRPDDGSITGPCESNGDKGSGGIKGGFVHQRMASKIGRWYSRTMSKNKRMGMGLYVKYDGDPWADSDALALSGVMVSMEEPGWYSFGFEIKDKKCDVPCIGIEMVHDGGKETWHSAATAIYCLMGSGQLLWDTVTGVDMAL |
| B/Victoria/149/2002 | 2002 | LPSTIQTLTLFLTSGGVLLSLYVSASLSYLLYSDILLKFSPTEITAPTMPLDCANASNVQAVNRSATKGVTLLLPEPEWTYPRLSCPGSTFQKALLISPHRFGETKGNSAPLIIREPFIACGPKECKHFALTHYAAQPGGYYNGTRGDRNKLRHLISVKLGKIPTVENSIFHMAAWSGSACHDGKEWTYIGVDGPDNNALLKIKYGEAYTDTYHSYANNILRTQESACNCIGGNCYLMITDGSASGISECRFLKIREGRIIKEIFPTGRVKHTEECTCGFASNKTIECACRDNSYTAKRPFVKLNVETDTAEIRLMCTETYLDTPRPDDGSITGPCESNGDKGSGGIKGGFVHQRMASKIGRWYSRTMSKTKRMGMGLYVKYDGDPWADSDALALSGVMVSMEEPGWYSFGFEIKDKKCDVPCIGIEMVHDGGKETWHSAATAIYCLMGSGQLLWDTVTGVDMAL |
| B/Victoria/156/2002 | 2002 | LPSTIQTLTLFLTSGGVLLSLYVSASLSYLLYSDILLKFSPTEIIAPTMPLDCANASNVQAVNRSATKGVTLLLPEPEWTYPRLSCPGSTFQKALLISPHRFGETKGNSAPLIIREPFIACGPKECKHFALTHYAAQPGGYYNGTRGDRNKLRHLISVKLGKIPTVENSIFHMAAWSGSACHDGKEWTYIGVDGPDNNALLKIKYGEAYTDTYHSYANNILRTQESACNCIGGNCYLMITDGSASGISECRFLKIREGRIIKEIFPTGRVKHTEECTCGFASNKTIECACRDNSYTAKRPFVKLNVETDTAEIRLMCTETYLDTPRPDDGSITGPCESNGDKGSGGIKGGFVHQRMASKIGRWYSRTMSKTKRMGMGLYVKYDGDPWADSDALALSGVMVSMEEPGWYSFGFEIKDKKCDVPCIGIEMVHDGGKETWHSAATAIYCLMGSGQLLWDTVTGVDMAL |
| B/Victoria/158/2002 | 2002 | LPSTIQTLTLFLTSGGVLLSLYVSASLSYLLYSDILLKFSPTEIIAPTMPLDCANASNVQAVNRSATKGVTLLLPEPEWTYPRLSCPGSTFQKALLISPHRFGETKGNSAPLIIREPFIACGPKECKHFALTHYAAQPGGYYNGTRGDRNKLRHLISVKLGKIPTVENSIFHMAAWSGSACHDGKEWTYIGVDGPDNNALLKIKYGEAYTDTYHSYANNILRTQESACNCIGGNCYLMITDGSASGISECRFLKIREGRIIKEIFPTGRVKHTEECTCGFASNKTIECACRDNSYTAKRPFVKLNVETDTAEIRLMCTETYLDTPRPDDGSITGPCESNGDKGSGGIKGGFVHQRMASKIGRWYSRTMSKTKRMGMGLYVKYDGDPWADSDALALSGVMVSMEEPGWYSFGFEIKDKKCDVPCIGIEMVHDGGKETWHSAATAIYCLMGSGQLLWDTVTGVDMAL |
| B/Victoria/160/2002 | 2002 | LPSTIQTLTLFLTSGGVLLSLYVSASLSYLLYSDILLKFSPTEITAPTMPLDCANASNVQAVNRSATKGVTLLLPEPEWTYPRLSCPGSTFQKALLISPHRFGETKGNSAPLIIREPFIACGPKECKHFALTHYAAQPGGYYNGTRGDRNKLRHLISVKLGKIPTVENSIFHMAAWSGSACHDGKEWTYIGVDGPDNNALLKIKYGEAYTDTYHSYANNILRTQESACNCIGGNCYLMITDGSASGISECRFLKIREGRIIKEIFPTGRVKHTEECTCGFASNKTIECACRDNSYTAKRPFVKLNVETDTAEIRLMCTETYLDTPRPDDGSITGPCESNGDKGSGGIKGGFVHQRMASKIGRWYSRTMSKTKRMGMGLYVKYDGDPWADSDALALSGVMVSMEEPGWYSFGFEIKDKKCDVPCIGIEMVHDGGKETWHSAATAIYCLMGSGQLLWDTVTGVDMAL |
| B/Victoria/162/2002 | 2002 | LPSTIQTLTLFLTSGGVLLSLYVSASLSYLLYSDMLLKFSPTEITAPTMPLDCANASNVQAVNRSATKGVTLLLPEPEWTYPRLSCPGSTFQKALLISPHRFGETKGNSAPLIIREPFIACGPKECKHFALTHYAAQPGGYYSGTRGDRNKLRHLISVKLGKIPTVENSIFHMAAWSGSACHDGKEWTYIGVDGPDNNALLKIKYGEAYTDTYHSYANNILRTQESACNCIGGNCYLMITDGSASGISECRFLKIREGRIIKEIFPTGRVKHTEECTCGFASNKTIECACRDNSYTAKRPFVKLNVETDTAEIRLMCTETYLDTPRPDDGSITGPCESNGDKGSGGIKGGFVHQRMASKIGRWYSRTMSKNKRMGMGLYVKYDGDPWADSDALALSGVMVSMEEPGWYSFGFEIKDKKCDVPCIGIEMVHDGGKETWHSAATAIYCLMGSGQLLWDTVTGVDMAL |
| B/Victoria/349/2002 | 2002 | LPSTIQTLTLFLTSGGVLLSLYVSASLSYLLYSDILLKFSPTEIIAPTMPLDCANASNVQAVNRSATKGVTLLLPEPEWTYPRLSCPGSTFQKALLISPHRFGETKGNSAPLIIREPFIACGPKECKHFALTHYAAQPGGYYNGTRGDRNKLRHLISVKLGKIPTVENSIFHMAAWSGSACHDGKEWTYIGVDGPDNNALLKIKYGEAYTDTYHSYANNILRTQESACNCIGGNCYLMITDGSASGISECRFLKIREGRIIKEIFPTGRVKHTEECTCGFASNKTIECACRDNSYTAKRPFVKLNVETDTAEIRLMCTETYLDTPRPDDGSITGPCESNGDKGSGGIKGGFVHQRMASKIGRWYSRTMSKTKRMGMGLYVKYDGDPWADSDALALSGVMVSMEEPGWYSFGFEIKDKKCDVPCIGIEMVHDGGKETWHSAATAIYCLMGSGQLLWDTVTGVDMAL |
| B/Victoria/351/2002 | 2002 | LPSTIQTLTLFLTSGGVLLSLYVSASLSYLLYSDILLKFSPTEIIAPTMPLDCANASNVQAVNRSATKGVTLLLPEPEWTYPRLSCPGSTFQKALLISPHRFGETKGNSAPLIIREPFIACGPKECKHFALTHYAAQPGGYYNGTRGDRNKLRHLISVKLGKIPTVENSIFHMAAWSGSACHDGKEWTYIGVDGPDNNALLKIKYGEAYTDTYHSYANNILRTQESACNCIGGNCYLMITDGSASGISECRFLKIREGRIIKEIFPTGRVKHTEECTCGFASNKTIECACRDNSYTAKRPFVKLNVETDTAEIRLMCTETYLDTPRPDDGSITGPCESNGDKGSGGIKGGFVHQRMASKIGRWYSRTMSKTKRMGMGLYVKYDGDPWADSDALALSGVMVSMEEPGWYSFGFEIKDKKCDVPCIGIEMVHDGGKETWHSAATAIYCLMGSGQLLWDTVTGVDMAL |
| B/Victoria/354/2002 | 2002 | LPSTIQTLTLFLTSGGVLLSLYVSASLSYLLYSDILLKFSPTEIIAPTMPLDCANASNVQAVNRSATKGVTLLLPEPEWTYPRLSCPGSTFQKALLISPHRFGETKGNSAPLIIREPFIACGPKECKHFALTHYAAQPGGYYNGTRGDRNKLRHLISVKLGKIPTVENSIFHMAAWSGSACHDGKEWTYIGVDGPDNNALLKIKYGEAYTDTYHSYANNILRTQESACNCIGGNCYLMITDGSASGISECRFLKIREGRIIKEIFPTGRVKHTEECTCGFASNKTIECACRDNSYTAKRPFVKLNVETDTAEIRLMCTETYLDTPRPDDGSITGPCESNGDKGSGGIKGGFVHQRMASKIGRWYSRTMSKTKRMGMGLYVKYDGDPWADSDALALSGVMVSMEEPGWYSFGFEIKDKKCDVPCIGIEMVHDGGKETWHSAATAIYCLMGSGQLLWDTVTGVDMAL |
| B/Victoria/502/2002 | 2002 | LPSTIQTLTLFLTSGGVLLSLYVSASLSYLLYSDILLKFSPTEITAPTVPLDCANASNVQAVNRSATKGVTLLLPEPEWTYPRLSCPGSTFQKALLISPHRFGETKGNSAPLIIREPFIACGPKECKHFALTHYAAQPGGYYSGTRGDRNKLRHLISVKLGKIPTVENSIFHMAAWSGSACHDGKEWTYIGVDGPDNNALLKIKYGEAYTDTYHSYANNILRTQESACNCIGGNCYLMITDGSASGISECRFLKIREGRIIKEIFPTGRVKHTEECTCGFASNKTIECACRDNSYTAKRPFVKLNVETDTAEIRLMCTETYLDTPRPDDGSITGPCESNGDKGSGGIKGGFVHQRMASKIGRWYSRTMSKNKRMGMGLYVKYDGDPWADSDALALSGVMVSMEEPGWYSFGFEIKDKKCDVPCIGIEMVHDGGKETWHSAATAIYCLMGSGQLLWDTVTGVDMAL |
| B/Victoria/503/2002 | 2002 | LPSTIQTLTLFLTTGGVLLSLYVSASLSYLLYSDILLKFSPTEIIAPTMPLDCANASNVQAVNRSATKGVTLLLPEPEWTYPRLSCPGSTFQKALLISPHRFGETKGNSAPLIIREPFIACGPKECKHFALTHYAAQPGGYYNGTRGDRNKLRHLISVKLGKIPTVENSIFHMAAWSGSACHDGKEWTYIGVDGPDNNALLKIKYGEAYTDTYHSYANNILRTQESACNCIGGNCYLMITDGSASGISECRFLKIREGRIIKEIFPTGRVKHTEECTCGFASNKTIECACRDNSYTAKRPFVKLNVETDTAEIRLMCTETYLDTPRPDDGSITGPCESNGDKGSGGIKGGFVHQRMASKIGRWYSRTMSKTKRMGMGLYVKYDGDPWADSDALALSGVMVSMEEPGWYSFGFEIKDKKCDVPCIGIEMVHDGGKETWHSAATAIYCLMGSGQLLWDTVTGVDMAL |
| B/Waikato/11/2002 | 2002 | LPSTIQTLTLFLTSGGVLLSLYVSASLSYLLYSDILLKFSPTEITAPTMPLDCANASNVQAVNRSATKGVTLLLPEPEWTYPRLSCPGSTFQKALLISPHRFGETKGNSAPLIIREPFIACGPKECKHFALTHYAAQPGGYYNGTRGDRNKLRHLISVKLGKIPTVENSIFHMAAWSGSACHDGKEWTYIGVDGPDNNALLKIKYGEAYTDTYHSYANNILRTQESACNCIGGNCYLMITDGSASGISECRFLKIREGRIIKEIFPTGRVKHTEECTCGFASNKTIECACRDNSYTAKRPFVKLNVETDTAEIRLMCTETYLDTPRPDDGSITGPCESNGDKGSGGIKGGFVHQRMASKIGRWYSRTMSKTKRMGMGLYVKYDGDPWADSDALALSGVMVSMEEPGWYSFGFEIRDKKCDVPCIGIEIVHDGGKETWHSAATAIYCLMGSGQLLWDTVTGVDMAL |
| B/Waikato/15/2002 | 2002 | LPSTIQTLTLFLTSGGVLLSLYVSASLSYLLYSDILLKFSPTEITAPTMPLDCANASNVQAVNRSATKGVTLLLPEPEWTYPRLSCPGSTFQKALLISPHRFGETKGNSAPLIIREPFIACGPKECKHFALTHYAAQPGGYYNGTRGDRNKLRHLISVKLGKIPTVENSIFHMAAWSGSACHDGKEWTYIGVDGPDNNALLKIKYGEAYTDTYHSYANNILRTQESACNCIGGNCYLMITDGSASGISECRFLKIREGRIIKEIFPTGRVKHTEECTCGFASNKTIECACRDNSYTAKRPFVKLNVETDTAEIRLMCTETYLDTPRPDDGSITGPCESNGDKGSGGIKGGFVHQRMASKIGRWYSRTMSKTKRMGMGLYVKYDGDPWADSDALALSGVMVSMEEPGWYSFGFEIKDKKCDVPCIGIEMVHDGGKETWHSAATAIYCLMGSGQLLWDTVTGVDMAL |
| B/Wellington/2/2002 | 2002 | LPSTIQTLTLFLTSGGVLLSLYVSASLSYLLYSDILLKFSPTEITAPTMPLDCANASNVQAVNRSATKGVTLLLPEPEWTYPRLSCPGSTFQKALLISPHRFGETKGNSAPLIIREPFIACGPKECKHFALTHYAAQPGGYYNGTRGDRNKLRHLISVKLGKIPTVENSIFHMAAWSGSACHDGKEWTYIGVDGPDNNALLKIKYGEAYTDTYHSYANNILRTQESACNCIGGNCYLMITDGSASGISECRFLKIREGRIIKEIFPTGRVKHTEECTCGFASNKTIECACRDNSYTAKRPFVKLNVETDTAEIRLMCTETYLDTPRPDDGSITGPCESNGDKGSGGIKGGFVHQRMASKIGRWYSRTMSKTKRMGMGLYVKYDGDPWADSDALALSGVMVSMEEPGWYSFGFEIRDKKCDVPCIGIEMVHDGGKETWHSAATAIYCLMGSGQLLWDTVTGVDMAL |
| B/Auckland/1/2003 | 2003 | LPSTIQTLTLFLTSGGVLLSLYVSASLSYLLYSDVLLKFSPAEITAPTMPLDCANASNVQAVNRSATKGVTLLLPEPEWTYPRLSCPGSTFQKALLISPHRFGETKGNSAPLIIREPFIACGPKECKHFALTHYAAQPGGYYNGTRGDRNKLRHLISVKLGKIPTVENSIFHMAAWSGSACHDGKEWTYIGVDGPDNNALLKIKYGEAYTDTYHSYANNILRTQESACNCIGGNCYLMITDGSASGVSECRFLKIREGRIIKEIFPTGRVKHTEECTCGFASNKTIECACRDNSYTAKRPFVKLNVETDTAEIRLMCTETYLDTPRPDDGSITGPCESNGDKGSGGIKGGFVHQRMASKIGRWYSRTMSKTKRMGMGLYVKYDGDPWADSDALALSGVMVSMEEPGWYSFGFEIKDKKCDVPCIGIEMVHDGGKETWHSAATAIYCLMGSGQLLWDTVTGVDMAL |
| B/Chanthaburi/218/2003 | 2003 | LPSTIQTLTLFLTSGGVLLSLYVSASLSYLLYSDILLKFSTTEITAPTMPLDCANASNVQAVNRSATKGVTLLLPEPEWTYPRLSCPGSTFQKALLISPHRFGETKGNSAPLIIREPFIACGPKECKHFALTHYAAQPGGYYNGTREDRNKLRHLISVKLGKIPTVENSIFHMAAWSGSACHDGKEWTYIGVDGPDSNALLKIKYGEAYTDTYHSYANNILRTQESACNCIGGNCYLMITDGSASGISECRFLKIREGRIIKEIFPTGRVKHTEECTCGFASNKTIECACRDNSYTAKRPFVKLNVETDTAEIRLMCTETYLDTPRPDDGSITGPCESNGNKGSGGIKGGFVHQRMASKIGRWYSRTMSKTKRMGMGLYVKYDGDPWTDSDALALSGVMVSMEEPGWYSFGFEIKDKKCDVPCIGIEMVHDGGKETWHSAATAIYCLMGSGQLLWDTVTGVDMAL |
| B/Finland/162/03 | 2003 | LPSTIQTLTLFLTSGGVLLSLYVSASLSYLLYSDILLKFSPTEITAPTMPLDCANASNVQAVNHSATKGVTLLLPEPEWTYPRLSCPGSTFQKALLISPHRFGETKGNSAPLIIREXFIACGPKECKHFALTHYAAQPGGYYNGTRGDRNKLRHLISVKLGKIPTVENSIFHMAAWSGSACHDGKEWTYIGVDGPDNDALLKIKYGEAYTDTYHSYANNILRTQESACNCIGGNCYLMITDGSASGISECRFLKIREGRIIKEIFPTGRVKHTEECTCGFASNKTIECACRDNSYTAKRPFVKLNVETDTAEIRLMCTETYLDTPRPDDGSITGPCESNGDKGSGGIKGGFVHQRMASKIGRWYSRTMSKTKRMGMGLYVKYDGDPWADSDALALSGVMVSMEEPGWYSFGFEIKDKKCDVPCIGIEMVHDGGKETWHSAATAIYCLMGSGQLLWDTVTGVDMAL |
| B/Finland/164/2003 | 2003 | LPSTIQTLTLFLTSGGVLLSLYMSASLSYLLYSDILLKFSPTEITAPTMPLDCANASNVQAVNRSATKGVTLLLPEPEWTYPRLSCPGSTFQKALLISPHRFGETKGNSAPLIIREPFIACGPKECKHFALTHYAAQPGGYYNGTRGDRNKLRHLISVKLGKIPTVENSIFHMAAWSGSACHDGKEWTYIGVDGPDNNALLKIKYGEAYTDTYHSYANNILRTQESACNCIGGNCYLMITDGSASGVSECRFLKIREGRIIKEIFPTGRVKHTEECTCGFASNKTIECACRDNSYTAKRPFVKLNMETDTAEIRLMCTETYLDTPRPDDGSITGPCESNGDKGSGGIKGGFVHQRMASKIGRWYSRTMSKTERMGMGLYVKYDGDPWADSDALALSGVMVSMEEPGWYSFGFEIKDKKCDVPCIGIEMVHDGGKETWHSAATAIYCLMGSGQLLWDTVTGVDMAL |
| B/Finland/190/2003 | 2003 | LPSTIQTLTLFLTSGGVLLSLYXSASLSYLLYSDILLKFSPTEITAPTMPLDCANASNVQAVNRSATKGVTLLLPEPEWTYPRLSCPGSTFQKALLISPHRFGETKGNSAPLIIREPFIACGPKECKHFALTHYAAQPGGYYNGTRGDRNKLRHLISVKLGKIPTVENSIFHMAAWSGSACHDGKEWTYIGVDGPDNNALLKIKYGEAYTDTYHSYANNILRTQESACNCIGGNCYLMITDGSASGVSECRFLKIREGRIIKEIFPTGRVKHTEECTCGFASNKTIECACRDNSYTAKRPFVKLNMETDTAEIRLMCTETYLDTPRPDDGSITGPCESNGDKGSGGIKGGFVHQRMASKIGRWYSRTMSKTERMGMGLYVKYDGDPWADSDALALSGVMVSMEEPGWYSFGFEIKDKKCDVPCIGIEMVHDGGKETWHSAATAIYCLMGSGQLLWDTVTGVDMAL |
| B/Finland/191/2003 | 2003 | LPSTIQTLTLFLTSGGVLLSLYMSASLSYLLYSDILLKFSPTEITAPTMPLDCANASNVQAVNRSATKGVTLLLPEPEWTYPRLSCPGSTFQKALLISPHRFGETKGNSAPLIIREPFIACGPKECKHFALTHYAAQPGGYYNGTRGDRNKLRHLISVKLGKIPTVENSIFHMAAWSGSACHDGKEWTYIGVDGPDNNALLKIKYGEAYTDTYHSYANNILRTQESACNCIGGNCYLMITDGSASGVSECRFLKIREGRIIKEIFPTGRVKHTEECTCGFASNKTIECACRDNSYTAKRPFVKLNMETDTAEIRLMCTETYLDTPRPDDGSITGPCESNGDKGSGGIKGGFVHQRMASKIGRWYSRTMSKTERMGMGLYVKYDGDPWADSDALALSGVMVSMEEPGWYSFGFEIKDKKCDVPCIGIEMVHDGGKETWHSAATAIYCLMGSGQLLWDTVTGVDMAL |
| B/Finland/193/2003 | 2003 | LPSTIQTLTLFLTSGGVLLSLYMSASLSYLLYSDILLKFSPTEITAPTMPLDCANASNVQAVNRSATKGVTLLLPEPEWTYPRLSCPGSTFQKALLISPHRFGETKGNSAPLIIREPFIACGPKECKHFALTHYAAQPGGYYNGTRGDRNKLRHLISVKLGKIPTVENSIFHMAAWSGSACHDGKEWTYIGVDGPDNNALLKIKYGEAYTDTYHSYANNILRTQESACNCIGGNCYLMITDGSASGVSECRFLKIREGRIIKEIFPTGRVKHTEECTCGFASNKTIECACRDNSYTAKRPFVKLNMETDTAEIRLMCTETYLDTPRPDDGSITGPCESNGDKGSGGIKGGFVHQRMASKIGRWYSRTMSKTERMGMGLYVKYDGDPWADSDALALSGVMVSMEEPGWYSFGFEIKDKKCDVPCIGIEMVHDGGKETWHSAATAIYCLMGSGQLLWDTVTGVDMAL |
| B/Finland/203/2003 | 2003 | LPSTIQTLTLFLTSGGVLLSLYVSASLSYLLYSDIMLKFSPTEITAPTMPLDCANASNVQAVNRSSTKGVTLLLPEPEWTYPRLSCPGSTFQKALLISPHRFGETKGNSAPLIIREPFIACGPKECKHFALTHYAAQPGGYYNGTRGDRNKLRHLISVKLGKIPTVENSIFHMAAWSGSACHDGKEWTYIGVDGPDNNALLKIKYGEAYTDTYHSYANNILRTQESACNCIGGNCYLMITDGSASGVSECRFLKIREGRIIKEIFPTGRVKHTEECTCGFASNKTIECACRDNSYTAKRPFVKLNVETDTAEIRLMCTETYLDTPRPDDGSITGPCESNGDKGSGGIKGGFVHQRMASKIGRWYSRTMSKTERMGMGLYVKYDGDPWADSDALALSGVMVSMEEPGWYSFGFEIKDKKCDVPCIGIEMVHDGGKETWHSAATAIYCLMGSGQLLWDTVTGVDMAL |
| B/Finland/204/2003 | 2003 | LPSTIQTLTLFLTSGGVLLSLYVSASLSXLLXSDILLKFSPTEITAPTMPLDCANASNVQAVNHSATKGVTLLLPEPEWTYPRLSCPGSTFQKALLISPHRFGETKGNSAPLIIREPFIACGPKECKHFALTHYAAQPGGYYNGTRGXRNKLRHLISVKLGKIPTVENSIFHMAAWSGSACHDGKEWTYIGVDGPDNDALLKIKYGEAYTDTYHSYANNILRTQESACNCIGGNCYLMITDGSASGISECRFLKIREGRIIKEIFPTGRVKHTEECTCGFASNKTIECACRDNSYTAKRPFVKLNVETDTAEIRLMCTETYLDTPRPDDGSITGPCESNGDKGSGGIKGGFVHQRMASKIGRWYSRTMSKTKRMGMGLYVKYDGDPWADSDALALSGVMVSMEEPGWYSFGFEIKDKKCDVPCIGIEMVHDGGKETWHSAATAIYCLMGSGQLLWDTVTGVDMAL |
| B/Finland/223/2003 | 2003 | LPSTIQTLTLFLTSGGVLLSLYVSASLSYLLYSDILLKFSTTEINAPTMPLDCANASNVQAVNRSATKGVTFLLPEPEWTYPRLSCPGSTFQKALLISPHRFGETKGNSAPLIIREPFIACGPKECKHFALTHYAAQPGGYYNGTREDRNKLRHLISVKLGKIPTVENSIFHMAAWSGSACHDGKEWTYIGVDGPDSNALLKIKYGEAYTDTYHSYANNILRTQESACNCIGGNCYLMITDGSASGISECRFLKIREGRIIKEIFPTGRVKHTEECTCGFASNKTIECACRDNSYTAKRPFVKLNVETDTAEIRLMCTETYLDTPRPDDGSITGPCESNGNKGSGGIKGGFVHQRMASKIGRWYSRTMSKTKRMGMGLYVKYDGDPWTDSDALALSGVMVSMEEPGWYSFGFEIKDKKCDVPCIGIEMVHDGGKETWHSAATAIYCLMGSGQLLWDTVTGVDMAL |
| B/Finland/227/2003 | 2003 | LPSTIQTLTLFLTSGGVLLSLYVSASLSYLLYSDILLKFSPTEITAPTMPLDCANASNVQAVNRSATKGVTLLLPEPEWTYPRLSCPGSTFQKALLISPHRFGETKGNSAPLIIREPFIACGPKECKHFALTHYAAQPGGYYNGTRGDRNKLRHLISVKLGKIPTVENSIFHMAAWSGSACHDGKEWTYIGVDGPDNNALLKIKYGEAYTDTYHSYANNILRTQESACNCIGGNCYLMITDGSASGISECRFLKIREGRIIKEIFPTGRVKHTEECTCGFASNKTIECACRDNSYTAKRPFVKLNVETDTAEIRLMCTETYLDTPRPDDGSITGPCESNGDKGSGGIKGGFVHQRMASKIGRWYSRTMSKTKRMGMGLYVKYDGDPWADSDALALSGVMVSMEEPGWYSFGFEIKDKKCDVPCIGIEMVHDGGKETWHSAATAIYCLMGSGQLLWDTVTGVDMAL |
| B/Finland/231/2003 | 2003 | LPSTIQTLTLFLTSGGVLLSLYVSASLSYLLYSDILLKFSTTEINAPTMPLDCANASNVQAVNRSATKGVTFLLPEPEWTYPRLSCPGSTFQKALLISPHRFGETKGNSAPLIIREPFIACGPKECKHFALTHYAAQPGGYYNGTREDRNKLRHLISVKLGKIPTVENSIFHMAAWSGSACHDGKEWTYIGVDGPDSNALLKIKYGEAYTDTYHSYANNILRTQESACNCIGGNCYLMITDGSASGISECRFLKIREGRIIKEIFPTGRVKHTEECTCGFASNKTIECACRDNSYTAKRPFVKLNVETDTAEIRLMCTETYLDTPRPDDGSITGPCESNGNKGSGGIKGGFVHQRMASKIGRWYSRTMSKTKRMGMGLYVKYDGDPWTDSDALALSGVMVSMEEPGWYSFGFEIKDKKCDVPCIGIEMVHDGGKETWHSAATAIYCLMGSGQLLWDTVTGVDMAL |
| B/Finland/235/2003 | 2003 | LPSTIQTLTLFLTSGGVLLSLYVSASLSYLLYSDILLKFSPTEITAPTMPLDCANASNVQAVNHSATKGVTLLLPEPEWTYPRLSCPGSTFQKALLISPHRFGETKGNSAPLIIREPFIACGPKECKHFALTHYAAQPGGYYNGTRGDRNKLRHLISVKLGKIPTVENSIFHMAAWSGSACHDGKEWTYIGVDGPDNDALLKIKYGEAYTDTYHSYANNILRTQESACNCIGGNCYLMITDGSASGISECRFLKIREGRIIKEIFPTGRVKHTEECTCGFASNKTIECACRDNSYTAKRPFVKLNVETDTAEIRLMCTETYLDTPRPDDGSITGPCESNGDKGSGGIKGGFVHQRMASKIGRWYSRTMSKTKRMGMGLYVKYDGDPWADSDALALSGVMVSMEEPGWYSFGFEIKDKKCDVPCIGIEMVHDGGKETWHSAAXAIYCLMGSGQLLWDTVTGVDMAL |
| B/Finland/244/2003 | 2003 | LPSTIQTLTLFLTSGGVLLSLYVSASLSYLLYSDILLKFSPTEITAPTMPLDCANASNVQAVNHSATKGVTLLLPEPEWTYPRLSCPGSTFQKALLISPHRFGETKGNSAPLIIREPFIACGPKECKHFALTHYAAQPGGYYNGXRGDRNKLRHLISVKLGKIPTVENSIFHMAAWSGSACHDGKEWTYIGVDGPDNDALLKIKYGEAYTDTYHSYANNILRTQESACNCIGGNCYLMITDGSASGISECRFLKIREGRIIKEIFPTGRVKHTEECTCGFASNKTIECACRDNSYTAKRPFVKLNVETDTAEIRLMCTETYLDTPRPDDGSITGPCESNGDKGSGGIKGGFVHQRMASKIGRWYSRTMSKTKRMGMGLYVKYDGDPWADSDALALSGVMVSMEEPGWYSFGFEIKDKKCDVPCIGIEMVHDGGKETWHSAATAIYCLMGSGQLLWDTVTGVDMAL |
| B/Fujian/203/2003 | 2003 | LPSTIQTLTLFLTSGGVLLSLYVSASLSYLLYSDILLKFSPTEITAPTMPLDCANASNVQAVNRSATKGVTLLIPEPEWTYPRLSCPGSTFQKALLISPHRFGETKGNSAPLIIREPFIACGPKECKHFALTHYAAQPGGYYNGTRGDRNKLRHLISVKLGKIPTVENSIFHMAAWSGSACHDGKEWTYIGVDGPDNNALLKIKYGEAYTDTYHSYANNILRTQESACNCIGGNCYLMITDGSASGVSECRFLKIREGRIIKEIFPTGRVKHTEECTCGFANNKTIECACRDNSYTAKRPFVKLNVETDTAEIRLMCTETYLDTPRPDDGSITGPCESNGDKGSGGIKGGFVHQRMASKIGRWYSRTMSKTKRMGMGLYVKYDGDPWADSDALALSGVMVSMEEPGWYSFGFEIKDKKCDVPCIGIEMVHDGGKETWHSAATAIYCLMGSGQLLWDTVTGVNMAL |
| B/Georgia/NHRC0001/2003 | 2003 | LPSTIQTLTLFLTSGGVLLSLYVSASLSYLLYSDILLKFSPTEITAPTMPLDCANASNVQAVNRSATKGVTLLLPEPEWTYPRLSCPGSTFQKALLISPHRFGETKGNSAPLIIREPFIACGPKECKHFALTHYAAQPGGYYNGTRGDRNKLRHLISVKLGKIPTVETSIFHMAAWSGSACHDGKEWTYIGVDGPDNNALLKIKYGEAYTDTYHSYANNILRTQESACNCIGGNCYLMITDGSASGVSECRFLKIREGRIIKEIFPTGRVKHTEECTCGFASNKTIECACRDNSYTAKRPFVKLNVETDTAEIRLMCTETYLDTPRPDDGSITGPCESNGDKGSGGIKGGFVHQRMASKIGRWYSRTMSKTERMGMGLYVKYDGDPWADSDALALSGVMVSMEEPGWYSFGFEIKDKKCDVPCIGIEMVHDGGKETWHSAATAIYCLMGSGQLLWDTVTGVDMAL |
| B/Hawaii/NHRC0001/2003 | 2003 | LPSTIQTLTLFLTSGGVLLSLYVSASLSYLLYSDILLKFSPTEITAPTMPLDCANASNVQAVNRSATKGVTLLLPEPEWTYPRLSCPGSTFQKALLISPHRFGETKGNSAPLIIREPFIACGPKECKHFALTHYAAQPGGYYNGTRGDRNKLRHLISVKLGKIPTVENSIFHMAAWSGSACHDGKEWTYIGVDGPDNNALLKIKYGEAYTDTYHSYANNILRTQESACNCIGGNCYLMITDGSASGVSECRFLKIREGRIIKEIFPTGRVKHTEECTCGFASNKTIECACRDNSYTAKRPFVKLNVETDTAEIRLMCTETYLDTPRPDDGSITGPCESNGDKGSGGIKGGFVHQRMASKIGRWYSRTMSKTKRMGMGLYVKYDGDPWADSDALALSGVMVSMEEPGWYSFGFEIKDKKCDVPCIGIEMVHDGGKETWHSAATAIYCLMGSGQLLWDTVTGVDMAL |
| B/Hong Kong/553a/2003 | 2003 | LPSTIQTLTLFLTSGGVLLSLYVSASLSYLLYSDILLKFSPTKITAPTMPLDCANASNVQAVNRSATKGVTLLLPEPEWTYPRLSCPGSTFQKALLISPHRFGETKGNSAPLIIREPFIACGPKECKHFALTHYAAQPGGYYNGTRGDRNKLRHLISVKLGKIPTVENSIFHMAAWSGSACHDGKEWTYIGVDGPDNNALLKIKYGEAYTDTYHSYANNILRTQESACNCIGGNCYLMITDGSASGVSECRFLKIREGRIIKEIFPTGRVKHTEECTCGFASNKTIECACRDNSYTAKRPFVKLNVETDTAEIRLMCTKTYLDTPRPDDGSITGPCESNGDKGSGGIKGGFVHQRMASKIGRWYSRTMSKTKRMGMGLYVKYDGDPWADSDALALSGVMVSMEEPGWYSFGFEIKDKKCDVPCIGIEMVHDGGKETWHSAATAIYCLMGSGQLLWDTVTGVDMAL |
| B/Houston/B79/2003 | 2003 | LPSTIQTLTLFLTSGGVLLSLYVSASLSYLLYSDILLKFSPTEITAPTMPLDCANASNVQAVNRSATKGVTLLLPEPEWTYPRLSCPGSTFQKALLISPHRFGETKGNSAPLIIREPFIACGPNECKHFALTHYAAQPGGYYNGKRGDRNKLRHLISVKLGKIPTVENSIFHMAAWSGSACHDGKEWTYIGVDGPDNNALLKIKYGEAYTDTYHSYANNILRTQESACNCIGGNCYLMITDGSASGVSECRFLKIREGRIIKEIFPTGRVKHTEECTCGFASNKTIECACRDNSYTAKRPFVKLNVETDTAEIRLMCTETYLDTPRPDDGSITGPCESNGDKGSGGIKGGFVHQRMASKIGRWYSRTMSKTERMGMGLYVKYDGDPWADSDALALSGVMVSMEEPGWYSFGFEIKDKKCDVPCIGIEMVHDGGKETWHSAATAIYCLMGSGQLLWDTVTGVDMAL |
| B/Houston/B81/2003 | 2003 | LPSTIQTLTLFLTSGGVLLSLYVSASLSYLLYSDILLKFSPTEITAPTMPLDYANASNVQAVNRSATKGVTLLLPEPEWTYPRLSCPGSTFQKALLISPHRFGETKGNSAPLIIREPFIACGPNECKHFALTHYAAQPGGYYNGTRGDRNKLRHLISVKLGKIPTVENSIFHMAAWSGSACHDGKEWTYIGVDGPDNNALLKIKYGEAYTDTYHSYANNILRTQESACNCIGGNCYLMITDGSASGVSECRFLKIREGRIIKEIFPTGRVKHTEECTCGFASNKTIECACRDNSYTAKRPFVKLNVETDTAEIRLMCTETYLDTPRPDDGSITGPCESNGDKGSGGIKGGFVHQRMASKIGRWYSRTMSKTERMGMGLYVKYDGDPWADSDALALSGVMVSMEEPGWYSFGFEIKDKKCDVPCIGIEMVHDGGKETWHSAATAIYCLMGSGQLLWDTVTGVDMAL |
| B/Houston/B86/2003 | 2003 | LPSTIQTLTLFLTSGGVLLSLYVSASLSYLLYSDILLKFSPTEITAPTMPLDCANASNVQAVNRSATKGVTLLLPEPEWTYPRLSCPGSTFQKALLISPHRFGETKGNSAPLIIREPFIACGPNECKHFALTHYAAQPGGYYNGTRGDRNKLRHLISVKLGKIPTVENSIFHMAAWSGSACHDGKEWTYIGVDGPDNNALLKIKYGEAYTDTYHSYANNILRTQESACNCIGGNCYLMITDGSASGVSECRFLKIREGRIIKEIFPTGRVKHTEECTCGFASNKTIECACRDNSYTAKRPFVKLNVETDTAEIRLMCTETYLDTPRPDDGSITGPCESNGDKGSGGIKGGFVHQRMASKIGRWYSRTMSKTERMGMGLYVKYDGDPWADSDALALSGVMVSMEEPGWYSFGFEIKDKKCDVPCIGIEMVHDGGKETWHSAATAIYCLMGSGQLLWDTVTGVDMAL |
| B/Houston/B87/2003 | 2003 | LPSTIQTLTLFLTSGGVLLSLYVSASLSYLLYSDILLKFSPTEITAPTMPLDCANASNVQAVNRSATKGVTLLLPEPEWTYPRLSCPGSTFQKALLISPHRFGETKGNSAPLIIREPFIACGPNECKHFALTHYAAQPGGYYNGTRGDRNKLRHLISVKLGKIPTVENSIFHMAAWSGSACHDGKEWTYIGVDGPDNNALLKIKYGEAYTDTYHSYANNILRTQESACNCIGGNCYLMITDGSASGVSECRFLKIREGRIIKEIFPTGRVKHTEECTCGFASNKTIECACRDNSYTAKRPFVKLNVETDTAEIRLMCTETYLDTPRPDDGSITGPCESNGDKGSGGIKGGFVHQRMASKIGRWYSRTMSKTERMGMGLYVKYDGDPWADSDALALSGVMVSMEEPGWYSFGFEIKDKKCDVPCIGIEMVHDGGKETWHSAATAIYCLMGSGQLLWDTVTGVDMAL |
| B/Indonesia/918099/2003 | 2003 | LPSTIQTLTLFLTSGGVLLSLYVSASLSYLLYSDILLKFSQTEITAPIMPLDCANASNVQAVNRSATKGVTLLLPEPEWTYPRLSCPGSTFQKALLISPHRFGETKGNSAPLIIREPFIACGPKECKHFALTHYAAQPGGYYNGTREDRNKLRHLISVKLGKIPTVENSIFHMAAWSGSACHDGKEWTYIGVDGPDSNALLKIKYGEAYTDTYHSYAKNILRTQESACNCIGGDCYLMITDGPASGISECRFLKIREGRIIKEIFPTGRVKHTEECTCGFASNKTIECACRDNSYTAKRPFVKLNVETDTAEIRLMCTETYLDTPRPNDGSITGPCESDGDKGSGGIKGGFVHQRMASKIGRWYSRTMSKTKRMGMGLYVKYDGDPWTDSEALALSGVMVSMEEPGWYSFGFEIKDKKCDVPCIGIEMVHDGGKTTWHSAATAIYCLMGSGQLLWDTVTGVDMAL |
| B/Jiangsu/10/2003 | 2003 | LPSTIQTLTLFLTSGGVLLSLYVSASLSYLLYSDILLKFSTTETTAPTMPLDCANASNVQAVNRSATKGVTLLLPEPEWTYPRLSCPGSTFQKALLISPHRFGETKGNSAPLIIREPFIACGPKECKHFALTHYAAQPGGYYNGTREDRNKLRHLISVKLGKIPTVENSIFHMAAWSGSACHDGKEWTYIGVDGPDSNALLKIKYGEAYTDTYHSYANNILRTQESACNCIGGNCYLMITDGSASGISECRFLKIREGRIIKEIFPTGRVKHTEECTCGFASNKTIECACRDNSYTAKRPFVKLNVETDTAEIRLMCTETYLDTPRPDDGSITGPCESNGNKGSGGIKGGFVHQRMASKIGRWYSRTMSKTKRMGMGLYVKYDGDPWTDSDALALSGVMVSMEEPGWYSFGFEIKDKKCDVPCIGIEMVHDGGKETWHSAATAIYCLMGSGQLLWDTVTGVDMAL |
| B/Jilin/20/2003 | 2003 | LPSTIQTLTLFLTSGGVLLSLYVSASLSYLLYSDILLKFSTTEITAPTMPLDCANASNVQAVNRSATKGVTLLLPEPEWTYPRLSCPGSTFQKALLISPHRFGETKGNSAPLIIREPFIACGPKECKHFALTHYAAQPGGYYNGTKEDRNKLRHLISVKLGKIPTVENSIFHMAAWSGSACHDGKEWTYIGVDGPDSNALLKIKYGEAYTDTYHSYANNILRTQESACNCIGGNCYLMITDGSASGISECRFLKIREGRIIKEIFPTGRVKHTEECTCGFASNKTIECACRDNSYTAKRPFVKLNVETDTAEIRLMCTETYLDTPRPDDGSITGPCESNGNKGSGGIKGGFVHQRMASKIGRWYSRTMSKTKRMGMGLYVKYDGDPWTDSDALALSGVMVSMEEPGWYSFGFEIKDKKCDVPCIGIEMVHDGGKETWHSAATAIYCLMGSGQLLWDTVTGVDMAL |
| B/Malaysia/234/2003 | 2003 | LPSTIQTLTLFLTSGGVLLSLYVSASLSYLLYSDILLKFSQTEITAPIMPLDCANASNVQAVNRSATKGVTLLLPEPEWTYPRLSCPGSTFQKALLISPHRFGETKGNSAPLIIREPFIACXXKECKHFALTHYAAQPGGYYNGTREDRNKLRHLISVKLGKIPTVENSIFHMAAWSGSACHDGREWTYIGVDGPDSNALLKIKYGEAYTDTYHSYAKNILRTQESACNCIGGDCYLMITDGPASGISECRFLKIREGRIIKEIFPTGRVKHTEECTCGFASNKTIECACRDNSYTAKRPFVKLNVETDTAEIRLMCTETYLDTPRPNDGSITGPCESNGDKGSGGIKGGFVHQRMASKIGRWYSRTMSKTKRMGMGLYVKYDGDPWTDSEALALSGVMVSMEEPGWYSFGFEIKDKKCDVPCIGIEMVHDGGKTTWHSAATAIYCLMGSGQLLWDTVTGVDMAL |
| B/Malaysia/24157/2003 | 2003 | LPSTIQTLTLFLTSGGVLLSLYVSASLSYLLYSDILLKFSQTEITAPTMPLDCANASNVQAVNRSATKGVTLLLPEPEWTYPRLSCPGSTFQKALLISPHRFGETKGNSAPLIIREPFIACGPKECKHFALTHYAAQPGGYYNGTREDRNKLRHLISVKLGKIPTVENSIFHMAAWSGSACHDGREWTYIGVDGPDSNALLKIKYGEAYTDTYHSYAKNILRTQESACNCIGGDCYLMITDGSASGISECRFLKIREGRIIKEIFPTGRVKHTEECTCGFASNKTIECACRDNSYTAKRPFVKLNVETDTAEIRLMCTETYLDTPRPNDGSITGPCESNGDRGSGGIKGGFVHQRMASKIGRWYSRTMSKTKRMGMGLYVKYDGDPWTDSEALALSGVMVPMEEPGWYSFGFEIKDKKCDVPCIGIEMVHDGGKTTWHSAATAIYCLMGSGQLLWDTVTGVDMAL |
| B/Malaysia/24296/2003 | 2003 | LPSTIQTLTLFLTSGGVLLSLYVSASLSYLLYSDILLKFSQTEITAPTMPLDCANASNVQAVNRSATKGVTLLLPEPEWTYPRLSCPGSTFQKALLISPHRFGETKGNSAPLIIREPFIACGPKECKHFALTHYAAQPGGYYNGTREDRNKLRHLISVKLGKIPTVENSIFHMAAWSGSACHDGREWTYIGVDGPDSNALLKIKYGEAYTDTYHSYAKNILRTQESACNCIGGDCYLMITDGSASGISECRFLKIREGRIIKEIFPTGRVKHTEECTCGFASNKTIECACRDNSYTAKRPFVKLNVETDTAEIRLMCTETYLDTPRPNDGSITGPCESNGDRGSGGIKGGFVHQRMASKIGRWYSRTMSKTKRMGMGLYVKYDGDPWTDSEALALSGVMVPMEEPGWYSFGFEIKDKKCDVPCIGIEMVHDGGKTTWHSAATAIYCLMGSGQLLWDTVTGVDMAL |
| B/Malaysia/24651/2003 | 2003 | LPSTIQTLTLFLTSGGVLLSLYVSASLSYLLYSDILLKFSQTEITAPTMPLDCANASNVQAVNRSATKGVTLLLPEPEWTYPRLSCPGSTFQKALLISPHRFGETKGNSAPLIIREPFIACGPKECKHFALTHYAAQPGGYYNGTREDRNKLRHLISVKLGKIPTVENSIFHMAAWSGSACHDGREWTYIGVDGPDSNALLKIKYGEAYTDTYHSYAKNILRTQESACNCIGGDCYLMITDGSASGISECRFLKIREGRIIKEIFPTGRVKHTEECTCGFASNKTIECACRDNSYTAKRPFVKLNVETDTAEIRLMCTETYLDTPRPNDGSITGPCESNGDRGGGGIKGGFVHQRMASKIGRWYSRTMSKTKRMGMGLYVKYDGDPWTDSEALALSGVMVPMEEPGWYSFGFEIKDKKCDVPCIGIEMVHDGGKTTWHSAATAIYCLMGSGQLLWDTVTGVDMAL |
| B/Malaysia/915/2003 | 2003 | LPSTIQTLTLFLTLGGVLLSLYVSASLSYLLYSDILLKFSQTEITAPIMPLDCANASNVQAVNRSATKGVTLLLPEPEWTYPRLSCPGSTFQKALLISPHRFGETKGNSAPLIIREPFIACGPQECKHFALTHYAAQPGGYYNGTREDRNKLRHLISVKLGKIPTVENSIFHMAAWSGSACHDGREWTYIGVDGPDSNALLKIKYGEAYTDTYHSYAKNILRTQESACNCIGGNCYLMITDGPASGISECRFLKIREGRIIKEIFPTGRVKHTEECTCGFASNKTIECACRDNSYTAKRPFVKLNVETDTAEIRLMCTETYLDTPRPNDGSITGPCESNGDKGSGGIKGGFVHQRMASKIGRWYSRTMSKTKRMGMGLYVKYDGDPWTDSEALALSGVMVSMEEPGWYSFGFEIKDKKCDVPCIGIEMVHDGGKKTWHSAATAIYCLMGSGQLLWDTVTGVDMAL |
| B/Memphis/1/2003 | 2003 | LPSTIQTLTLFLTSGGVLLSLYVSASLSYLLYSDILLKFSPTEITAPTMPLDCANASNVQAVNRSATKGVTLLLPEPEWTYPRLSCPGSTFQKALLISPHRFGETKGNSAPLIIREPFIACGPNECKHFALTHYAAQPGGYYNGTRGDRNKLRHLISVKLGKIPTVENSIFHMAAWSGSACHDGKEWTYIGVDGPDNNALLKIKYGEAYTDTYHSYANNILRTQESACNCIGGNCYLMITDGSASGVSECRFLKIREGRIIKEIFPTGRVKHTEECTCGFASNKIIECACRDSSYTAKRPFVKLNVETDTAEIRLMCTETYLDTPRPNDGSITGPCESNGDKGSGGIKGGFVHQRMASKIGRWYSRTMSKTERMGMGLYVKYDGDPWADSDALALSGVMVSMEEPGWYSFGFEIKDKKCDVPCIGIEMVHDGGKETWHSAATAIYCLMGSGQLLWDTVTGVDMAL |
| B/Missouri/NHRC0001/2003 | 2003 | LPSTIQTLTLFLTSGGVLLSLYVSASLSYLLYSDILLKFSPTEITAPTMPLDCANASNVQAVNRSTTKGVTLLLPEPEWTYPRLSCPGSTFQKALLISPHRFGETKGNSAPLIIREPFIACGPNECKHFALTHYAAQPGGYYNGTRGDRNKLRHLISVKLGKIPTVENSIFHMAAWSGSACHDGKEWTYIGVDGPDNNALLKIKYGEAYTDTYHSYANNILRTQESACNCIGGNCYLMITDGSASGVSECRFLKIREGRIIKEIFPTGRVKHTEECTCGFASNKTIECACRDNSYTAKRPFVKLNVETDTAEIRLMCTETYLDTPRPDDGSITGPCESNGDKGSGGIKGGFVHQRMASKIGRWYSRTMSKTERMGMGLYVKYDGDPWADSDALALSGVMVSMEEPGWYSFGFEIKDKKCDVPCIGIEMVHDGGKETWHSAATAIYCLMGSGQLLWDTVTGVDMAL |
| B/New York/1055/2003 | 2003 | LPSTIQTLTLFLTSGGVLLSLYVSASLSYLLYSDILLKFSPTEITAPTMPLDCANASNVQAVNRSATKGVTLLLPEPEWTYPRLSCPGSTFQKALLISPHRFGETKGNSAPLIIREPFIACGPKECKHFALTHYAAQPGGYYNGTRGDRNKLRHLISVKLGKIPTVETSIFHMAAWSGSACHDGKEWTYIGVDGPDNNALLKIKYGEAYTDTYHSYANNILRTQESACNCIGGNCYLMITDGSASGVSECRFLKIREGRIIKEIFPTGRVKHTEECTCGFASNKTIECACRDNSYTAKRPFVKLNVETDTAEIRLMCTETYLDTPRPDDGSITGPCESNGDKGSGGIKGGFVHQRMASKIGRWYSRTMSKTERMGMGLYVKYDGDPWADSDALALSGVMVSMEEPGWYSFGFEIKDKKCDVPCIGIEMVHDGGKETWHSAATAIYCLMGSGQLLWDTVTGVDMAL |
| B/New York/1056/2003 | 2003 | LPSTIQTLTLFLTSGGVLLSLYVSASLSYLLYSDILLKFSPTEITAPTMPLDCANASNVQAVNRSATKGVTLLLPEPEWTYPRLSCPGSTFQKALLISPHRFGETKGNSAPLIIREPFIACGPKECKHFALTHYAAQPGGYYNGTRGDRNKLRHLISVKLGKIPTVETSIFHMAAWSGSACHDGKEWTYIGVDGPDNNALLKIKYGEAYTDTYHSYANNILRTQESACNCIGGNCYLMITDGSASGVSECRFLKIREGRIIKEIFPTGRVKHTEECTCGFASNKTIECACRDNSYTAKRPFVKLNVETDTAEIRLMCTETYLDTPRPDDGSITGPCESNGDKGSGGIKGGFVHQRMASKIGRWYSRTMSKTERMGMGLYVKYDGDPWADSDALALSGVMVSMEEPGWYSFGFEIKDKKCDVPCIGIEMVHDGGKETWHSAATAIYCLMGSGQLLWDTVTGVDMAL |
| B/New York/1057/2003 | 2003 | LPSTIQTLTLFLTSGGVLLSLYVSASLSYLLYSDILLKFSPTEITAPTMPLDCANASNVQAVNRSATKGVTLLLPEPEWTYPRLSCPGSTFQKALLISPHRFGETKGNSAPLIIREPFIACGPKECKHFALTHYAAQPGGYYNGTRGDRNKLRHLISVKLGKIPTVETSIFHMAAWSGSACHDGKEWTYIGVDGPDNNALLKIKYGEAYTDTYHSYANNILRTQESACNCIGGNCYLMITDGSASGVSECRFLKIREGRIIKEIFPTGRVKHTEECTCGFASNKTIECACRDNSYTAKRPFVKLNVETDTAEIRLMCTETYLDTPRPDDGSITGPCESNGDKGSGGIKGGFVHQRMASKIGRWYSRTMSKTERMGMGLYVKYDGDPWADSDALALSGVMVSMEEPGWYSFGFEIKDKKCDVPCIGIEMVHDGGKETWHSAATAIYCLMGSGQLLWDTVTGVDMAL |
| B/New York/1058/2003 | 2003 | LPSTIQTLTLFLTSGGVLLSLYVSASLSYLLYSDILLKFSPTEITAPTMPLDCANASNVQAVNRSATKGVTLLLPEPEWTYPRLSCPGSTFQKALLISPHRFGETKGNSAPLIIREPFIACGPNECKHFALTHYAAQPGGYYNGTRGDRNKLRHLISVKLGKIPTVENSIFHMAAWSGSACHDGKEWTYIGVDGPDNNALLKIKYGEAYTDTYHSYANNILRTQESACNCIGGNCYLMITDGSASGVSECRFLKIREGRIIKEIFPTGRVKHTEECTCGFASNKTIECACRDNSYTAKRPFVKLNVETDTAEIRLMCTETYLDTPRPDDGSITGPCESNGDKGSGGIKGGFVHQRMASKIGRWYSRTMSKTERMGMGLYVKYDGDPWADSDALALSGVMVSMEEPGWYSFGFEIKDKKCDVPCIGIEMVHDGGKETWHSAATAIYCLMGSGQLLWDTVTGVDMAL |
| B/New York/1059/2003 | 2003 | LPSTIQTLTLFLTSGGVLLSLYVSASLSYLLYSDILLKFSPTEITAPTMPLDCANASNVQAVNRSATKGVTLLLPEPEWTYPRLSCPGSTFQKALLISPHRFGETKGNSAPLIIREPFIACGPNECKHFALTHYAAQPGGYYNGTRGDRNKLRHLISVKLGKIPTVENSIFHMAAWSGSACHDGKEWTYIGVDGPDNNALLKIKYGEAYTDTYHSYANNILRTQESACNCIGGNCYLMVTDGSASGVSECRFLKIREGRIIKEIFPTGRVKHTEECTCGFASNKTIECACRDNSYTAKRPFVKLNVETDTAEIRLMCTETYLDTPRPDDGSITGPCESNGDKGSGGIKGGFVHQRMASKIGRWYSRTMSKTERMGMGLYVKYDGDPWADSDALALSGVMVSMEEPGWYSFGFEIKDKKCDVPCIGIEMVHDGGKETWHSAATAIYCLMGSGQLLWDTVTGVDMAL |
| B/New York/1394/2003 | 2003 | LPSTIQTLTLFLTSGGVLLSLYVSASLSYLLYSDILLKFSPTEITAPTMPLDCANASNVQAVNRSATKGVTLLLPEPEWTYPRLSCPGSTFQKALLISPHRFGETKGNSAPLIIREPFIACGPNECKHFALTHYAAQPGGYYNGTRGDRNKLRHLISVKLGKIPTVENSIFHMAAWSGSACHDGKEWTYIGVDGPDNNALLKIKYGEAYTDTYHSYANNILRTQESACNCIGGNCYLMITDGSASGVSECRFLKIREGRIIKEIFPTGRVKHTEECTCGFASNKTIECACRDNSYTAKRPFVKLNVETDTAEIRLMCTETYLDTPRPDDGSITGPCESNGDKGSGGIKGGFVHQRMTSKIGRWYSRTMSKTERMGMGLYVKYDGDPWADSDALALSGVMVSMEEPGWYSFGFEIKDKKCDVPCIGIEMVHDGGKETWHSAATAIYCLMGSGQLLWDTVTGVDMAL |
| B/Paraguay/636/2003 | 2003 | LPSTIQTLTLFLTSGGVLLSLYVSASLSYLLYSDILLKFSPTEITAPTMPLDCANASNVQAVNRSATKGVTLLIPEPEWTYPRLSCPGSTFQKALLISPHRFGETKGNSAPLIIREPFIACGPKECKHFALTHYAAQPGGYYNGTRGDRNKLRHLISVKLGKIPTVENSIFHMAAWSGSACHDGKEWTYIGVDGPDNSALLKIKYGEAYTDTYHSYANNILRTQESACNCIGGNCYLMITDGSASGVSECRFLKIREGRIIKEIFPTGRVKHTEECTCGFASNKTIECACRDNSYTAKRPFVKLNVETDTAEIRLMCTETYLDTPRPDDGSITGPCESNGDKGSGGIKGGFVHQRMASKIGRWYSRTMSKTKRMGMGLYVKYDGDPWADSDALALSGVMVSMEEPGWYSFGFEIKDKKCDVPCIGIEMVHDGGKETWHSAATAIYCLMGSGQLLWDTVTGVDMAL |
| B/Temple/B17/2003 | 2003 | LPSTIQTLTLFLTSGGVLLSLYVSASLSYLLYSDILLKFSPTEITAPTMPLDCANASNVQAVNRSATKGVTLLLPEPEWTYPRLSCPGSTFQKALLISPHRFGETKGNSAPLIIREPFIACGPNECKHFALTHYAAQPGGYYNGKRGDRNKLRHLISVKLGKIPTVENSIFHMAAWSGSACHDGKEWTYIGVDGPDNNALLKIKYGEAYTDTYHSYANNILRTQESACNCIGGNCYLMITDGSASGVSECRFLKIREGRIIKEIFPTGRVKHTEECTCGFASNKTIECACRDNSYTAKRPFVKLNVETDTAEIRLMCTETYLDTPRPDDGSITGPCESNGDKGSGGIKGGFVHQRMASKIGRWYSRTMSKTERMGMGLYVKYDGDPWADSDALALSGVMVSMEEPGWYSFGFEIKDKKCDVPCIGIEMVHDGGKETWHSAATAIYCLMGSGQLLWDTVTGVDMAL |
| B/Temple/B18/2003 | 2003 | LPSTIQTLTLFLTSGGVLLSLYVSASLSYLLYSDILLKFSPTEITAPTMPLDCANASNVQAVNRSATKGVTLLLPEPEWTYPRLSCPGSTFQKALLISPHRFGETKGNSAPLIIREPFIACGPNECKHFALTHYAAQPGGYYNGXRGXRNKLRHLISVKLGKIPTVENSIFHMAAWSGSACHDGKEWTYIGVDGPDNNALLKIKYGEAYTDTYHSYANNILRTQESACNCIGGNCYLMITDGSASGVSECRFLKIREGRIIKEIFPTGRVKHTEECTCGFASNKTIECACRDNSYTAKRPFVKLNVETDTAEIRLMCTETYLDTPRPDDGSITGPCESNGDKGSGGIKGGFVHQRMASKIGRWYSRTMSKTERMGMGLYVKYDGDPWADSDALALSGVMVSMEEPGWYSFGFEIKDKKCDVPCIGIEMVHDGGKETWHSAATAIYCLMGSGQLLWDTVTGVDMAL |
| B/Temple/B19/2003 | 2003 | LPSTIQTLTLFLTSGGVLLSLYVSASLSYLLYSDILLKFSPTEITAPTMPLDCANASNVQAVNRSATKGVTLLLPEPEWTYPRLSCPGSTFQKALLISPHRFGETKGNSAPLIIREPFIACGPNECKHFALTHYAAQPGGYYNGTRGDRNKLRHLISVKLGKIPTVENSIFHMAAWSGSACHDGKEWTYIGVDGPDNNALLKIKYGEAYTDTYHSYANNILRTQESACNCIGGNCYLMITDGSASGVSECRFLKIREGRIIKEIFPTGRVKHTEECTCGFASNKTIECACRDNSYTAKRPFVKLNVETDTAEIRLMCTETYLDTPRPDDGSITGPCESNGDKGSGGIKGGFVHQRMASKIGRWYSRTMSKTERMGMGLYVKYDGDPWADSDALALSGVMVSMEEPGWYSFGFEIKDKKCDVPCIGIEMVHDGGKETWHSAATAIYCLMGSGQLLWDTVTGVDMAL |
| B/Temple/B20/2003 | 2003 | LPSTIQTLTLFLTSGGVLLSLYVSASLSYLLYSDILLKFSPTEITAPTMPLDCANASNVQAVNRSATKGVTLLLPEPEWTYPRLSCPGSTFQKALLISPHRFGETKGNSAPLIIREPFIACGPNECKHFALTHYAAQPGGYYNGTRGDRNKLRHLISVKLGKIPTVENSIFHMAAWSGSACHDGKEWTYIGVDGPDNNALLKIKYGEAYTDTYHSYANNILRTQESACNCIGGNCYLMITDGSASGVSECRFLKIREGRIIKEIFPTGRVKHTEECTCGFASNKTIECACRDNSYTAKRPFVKLNVETDTAEIRLMCTETYLDTPRPDDGSITGPCESNGDKGSGGIKGGFVHQRMASKIGRWYSRTMSKTERMGMGLYVKYDGDPWADSDALALSGVMVSMEEPGWYSFGFEIKDKKCDVPCIGIEMVHDGGKETWHSAATAIYCLMGSGQLLWDTVTGVDMAL |
| B/Temple/B21/2003 | 2003 | LPSTIQTLTLFLTSGGVLLSLYVSASLSYLLYSDILLKFSPTEITAPTMPLDCANASNVQAVNRSATKGVTLLLPEPEWTYPRLSCPGSTFQKALLISPHRFGETKGNSAPLIIREPFIACGPNECKHFALTHYAAQPGGYYNGKRGDRNKLRHLISVKLGKIPTVENSIFHMAAWSGSACHDGKEWTYIGVDGPDNNALLKIKYGEAYTDTYHSYANNILRTQESACNCIGGNCYLMITDGSASGVSECRFLKIREGRIIKEIFPTGRVKHTEECTCGFASNKTIECACRDNSYTAKRPFVKLNVETDTAEIRLMCTETYLDTPRPDDGSITGPCESNGDKGSGGIKGGFVHQRMASKIGRWYSRTMSKTERMGMGLYVKYDGDPWADSDALALSGVMVSMEEPGWYSFGFEIKDKKCDVPCIGIEMVHDGGKETWHSAATAIYCLMGSGQLLWDTVTGVDMAL |
| B/Temple/B24/2003 | 2003 | LPSTIQTLTLFLTSGGVLLSLYVSASLSYLLYSDILLKFSPTEITAPTMPLDCANASNVQAVNRSATKGVTLLLPEPEWTYPRLSCPGSTFQKALLISPHRFGETKGNSAPLIIREPFIACGPNECKHFALTHYAAQPGGYYNGTRGERNKLRHLISVKLGKIPTVENSIFHMAAWSGSACHDGKEWTYIGVDGPDNNALLKIKYGEAYTDTYHSYANNILRTQESACNCIGGNCYLMITDGSASGVSECRFLKIREGRIIKEIFPTGRVKHTEECTCGFASNKTIECACRDNSYTAKRPFVKLNVETDTAEIRLMCTETYLDTPRPDDGSITGPCESNGDKGSGGIKGGFVHQRMASKIGRWYSRTMSKTERMGMGLYVKYDGDPWADSDALALSGVMVSMEEPGWYSFGFEIKDKKCDVPCIGIEMVHDGGKETWHSAATAIYCLMGSGQLLWDTVTGVDMAL |
| B/Temple/B30/2003 | 2003 | LPSTIQTLTLFLTSGGVLLSLYVSASLSYLLYSDILLKFSPTEITAPTMPLDCANASNVQAVNRSATKGVTLLLPEPEWTYPRLSCPGSTFQKALLISPHRFGETKGNSAPLIIREPFIACGPNECKHFALTHYAAQPGGYYNGTRGDRNKLRHLISVKLGKIPTVENSIFHMAAWSGSACHDGKEWTYIGVDGPDNNALLKIKYGEAYTDTYHSYANNILRTQESACNCIGGNCYLMITDGSASGVSECRFLKIREGRIIKEIFPTGRVKHTEECTCGFASNKIIECACRDNSYTAKRPFVKLNVETDTAEIRLMCTETYLDTPRPDDGSITGPCESNGDKGSGGIKGGFVHQRMASKIGRWYSRTMSKTERMGMGLYVKYDGDPWADSDALALSGVMVSMEEPGWYSFGFEIKDKKCDVPCIGIEMVHDGGKETWHSAATAIYCLMGSGQLLWDTVTGVDMAL |
| B/Townsville/1/2003 | 2003 | LPSTIQTLTLFLTSGGVLLSLYVSASLSYLLYSDILLKFSQTEITAPIMPLDCANASNVQAVNRSATKGVTLLLPEPEWTYPRLSCPGSTFQKALLISPHRFGETKGNSAPLIIREPFIACGPKECKHFALTHYAAQPGGYYNGTREDRNKLRHLISVKLGKIPTVENSIFHMAAWSGSACHDGREWTYIGVDGPDSNALLKIKYGEAYTDTYHSYAKNILRTQESACNCIGGDCYLMITDGPASGISECRFLKIREGRIIKEIFPTGRVKHTEECTCGFASNKTIECACRDNSYTAKRPFVKLNVETDTAEIRLMCTETYLDTPRPNDGSITGPCESNGDKGSGGIKGGFVHQRMASKIGRWYSRTMSKTKRMGMGLYVKYDGDPWTDSEALALSGVMVSMEEPGWYSFGFEIKDKKCDVPCIGIEMVHDGGKTTWHSAATAIYCLMGSGQLLWDTVTGVDMAL |
| B/Ulan-Ude/6/2003 | 2003 | LPSTIQTLTLFLTSGGVLLSLYVSASLSYLLYSDILLKFSQTEITAPTMPLDCANASNVQAVNRSATKGVTLLLPEPEWTYPRLSCPGSTFQKALLISPHRFGETKGNSAPLIIREPFIACGPKECKHFALTHYAAQPGGYYNGTREDRNKLRHLISVKLGKIPTVENSIFHMAAWSGSACHDGREWTYIGVDGPDSNALLKIKYGEAYTDTYHSYAKNILRTQESACNCIGGDCYLMITDGSASGISECRFLKIREGRIIKEIFPTGRVKHTEECTCGFASNKTIECACRDNSYTAKRPFVKLNVETDTAEIRLMCTETYLDTPRPNDGSITGPCESNGDKGSGGIKGGFVHQRMASKIGRWYSRTMSKTKRMGMGLYVKYDGDPWTDSEALALSGVMVSMEEPGWYSFGFEIKDKKCDVPCIGIEMVHDGGKTTWHSAATAIYCLMGSGQLLWDTVTGVDMAL |
| B/Yamagata/1246/2003 | 2003 | LPSTIQTLTLFLTSGGVLLSLYVSASLSYLLYSDILLKFSTTEITAPTMPLDCANASNVQAVNRSATKGVTLLLPEPEWTYPRLSCPGSTFQKALLISPHRFGETKGNSAPLIIREPFIACGPKECKHFALTHYAAQPGGYYNGTREDRNKLRHLISVKLGKIPTVENSIFHMAAWSGSACHDGKEWTYIGVDGPDSNALLKIKYGEAYTDTYHSYANNILRTQESACNCIGGNCYLMITDGSASGISECRFLKIREGRIIKEIFPTGRVKHTEECTCGFASNKTIECACRDNSYTAKRPFVKLNVETDTAEIRLMCTETYLDTPRPDDGSITGPCESNGNKGSGGIKGGFVHQRMASKIGRWYSRTMSKTKRMGMGLYVKYDGDPWTDSDALALSGVMVSMEEPGWYSFGFEIKDKKCDVPCIGIEMVHDGGKETWHSAATAIYCLMGSGQLLWDTVTGVDMAL |
| B/Yamagata/1311/2003 | 2003 | LPSTIQTLTLFLTSGGVLLSLYVSASLSYLLYSDILLKFSQTEITAPIMPLDCANASNVQAVNRSATKGVTLLLPEPEWTYPRLSCPGSTFQKALLISPHRFGETKGNSAPLIIREPFIACGPKECKHFALTHYAAQPGGYYNGTREDRNKLRHLISVKLGKIPTVENSIFHMAAWSGSACHDGREWTYIGVDGPDSNALLKIKYGEAYTDTYHSYAKNILRTQESACNCIGGDCYLMITDGPASGISECRFLKIREGRIIKEIFPTGRVKHTEECTCGFASNKTIECACRDNSYTAKRPFVKLNVETDTAEIRLMCTETYLDTPRPNDGSITGPCESNGDKGSGGIKGGFVHQRMASKIGRWYSRTMSKTKRMGMGLYVKYDGDPWTDSEALALSGVMVSMEEPGWYSFGFEIKDKKCDVPCIGIEMVHDGGKTTWHSAATAIYCLMGSGQLLWDTVTGVDMAL |
| B/Auckland/1/2004 | 2004 | LPSTIQTLTLFLTSGGVLLSLYVSASLSYLLYSDILLKFSQTEITAPIMPLDCTNASNVQAVNRSATKGVTLLLPEPEWTYPRLSCPGSTFQKALLISPHRFGETKGNSAPLIIREPFIACGPKECKHFALTHYAAQPGGYYNGTREDRNKLRHLISVKLGKIPTVENSIFHMAAWSGSACHDGREWTYIGVDGPDSNALLKIKYGEAYTDTYHSYAKNILRTQESACNCIGGDCYLMITDGPASGISECRFLKIREGRIIKEIFPTGRVKHTEECTCGFASNKTIECACRDNSYTAKRPFVKLNVETDTAEIRLMCTETYLDTPRPNDGSITGPCESNGDKGSGGIKGGFVHQRMASKIGRWYSRTMSKTKRMGMGLYVKYDGDPWTDSEALALSGVMVSMEEPGWYSFGFEIKDKKCDVPCIGIEMVHDGGKTTWHSAATAIYCLMGSGQLLWDTVTGVDMAL |
| B/Brisbane/1/2004 | 2004 | LPSTIQTLTLFLTSGGVLLSLYVSASLSYLLYSDILLKFSQTEIIAPIMPLDCANASNVQAVNHSATKGVTLLLPEPEWTYPRLSCPGSTFQKALLISPHRFGETKGNSAPLIIREPFIACGPKECKHFALTHYAAQPGGYYNGTREDRNKLRHLISVKLGKIPTVENSIFHMAAWSGSACHDGREWTYIGVDGPDSNALLKIKYGEAYTDTYHSYAKNILRTQESACNCIGGDCYLMITDGPASGISECRFLKIREGRIIKEIFPTGRVKHTEECTCGFASNKTIECACRDNSYTAKRPFVKLNVETDTAEIRLMCTETYLDTPRPNDGSITGPCESNGDKGSGGIKGGFVHQRMASKIGRWYSRTMSKTKRMGMGLYVKYDGDPWTDSEALALSGVMVSMEEPGWYSFGFEIKDKKCDVPCIGIEMVHDGGKTTWHSAATAIYCLMGSGQLLWDTVTGVDMAL |
| B/Brisbane/10/2004 | 2004 | LPSTIQTLTLFLTSGGVLLSLYVSASLSYLLYSDILLKFSQTEITAPIMPLDCANASNVQAVNRSATKGVTLLLPEPEWTYPRLSCPGSTFQKALLISPHRFGETKGNSAPLIIREPFIACGPKECKHFALTHYAAQPGGYYNGTREDRNKLRHLISVKLGKIPTVENSIFHMAAWSGSACHDGREWTYIGVDGPDSNALLKIKYGEAYTDTYHSYAKNILRTQESACNCIGGDCYLMITDGPASGISECRFLKIREGRIIKEIFPTGRVKHTEECTCGFASNKTIECACRDNSYTAKRPFVKLNVETDTAEIRLMCTETYLDTPRPNDGSITGPCESNGDKGSGGIKGGFVHQRMASKIGRWYSRTISKTKRMGMGLYVKYDGDPWTDSEALALSGVMVSMEEPGWYSFGFEIKDKKCDVPCIGIEMVHDGGKTTWHSAATAIYCLMGSGQLLWDTVTGVDMAL |
| B/Brisbane/13/2004 | 2004 | LPSTIQTLTLFLTSGGVLLSLYVSASLSYLLYSDILLKFSQTEITAPIMPLDCANASNVQAVNRSATKGVTLLLPEPEWTYPRLSCPGSTFQKALLISPHRFGETKGNSAPLIIREPFIACGPKECKHFALTHYAAQPGGYYNGTREDRNKLRHLISVKLGKIPTVENSIFHMAAWSGSACHDGREWTYIGVDGPDSNALLKIKYGEAYTDTYHSYAKNILRTQESACNCIGGDCYLMITDGPASGISECRFLKIREGRIIKEIFPTGRVKHTEECTCGFASNKTIECACRDNSYTAKRPFVKLNVETDTAEIRLMCTETYLDTPRPNDGSITGPCESNGDKGSGGIKGGFVHQRMASKIGRWYSRTMSKTKRMGMGLYVKYDGDPWTDSEALALSGVMVSMEEPGWYSFGFEIKDKKCDVPCIGIEMVHDGGKTTWHSAATAIYCLMGSGQLLWDTVTGVDMAL |
| B/Brisbane/14/2004 | 2004 | LPSTIQTLTLFLTSGGVLLSLYVSASLSYLLYSDILLKFSQTEITAPIMPLDCANASNVQAVNRSATKGVTLLLPEPEWTYPRLSCPGSTFQKALLISPHRFGETKGNSAPLIIREPFIACGPKECKHFALTHYAAQPGGYYNGTREDRNKLRHLISVKLGKIPTVENSIFHMAAWSGSACHDGREWTYIGVDGPDSNALLKIKYGEAYTDTYHSYAKNILRTQESACNCIGGDCYLMITDGPASGISECRFLKIREGRIIKEIFPTGRVKHTEECTCGFASNKTIECACRDNNYTAKRPFVKLNVETDTAEIRLMCTETYLDTPRPNDGSITGPCESNGDKGSGGIKGGFVHQRMASKIGRWYSRTMSKTKRMGMGLYVKYDGDPWTDSEALALSGVMVSMEEPGWYSFGFEIKDKKCDVPCIGIEMVHDGGKTTWHSAATAIYCLMGSGQLLWDTVTGVDMAL |
| B/Brisbane/19/2004 | 2004 | LPSTIQTLTLFLTSGGVLLSLYVSASLSYLLYSDILLKFSQTEITAPIMPLDCANASNVQAVNRSATKGVTLLLPEPEWTYPRLSCPGSTFQKALLISPHRFGETKGNSAPLIIREPFIACGPKECKHFALTHYAAQPGGYYNGTREDRNKLRHLISVKLGKIPTVENSIFHMAAWSGSACHDGREWTYIGVDGPDSNALLKIKYGEAYTDTYHSYAKNILRTQESACNCIGGDCYLMITDGPASGISECRFLKIREGRIIKEIFPTGRVKHTEECTCGFASNKTIECACRDNSYTAKRPFVKLNVETDTAEIRLMCTETYLDTPRPNDGSITGPCESNGDKGSGGIKGGFVHQRMASKIGRWYSRTMSKTKRMGMGLYVKYDGDPWTDSEALALSGVMVSMEEPGWYSFGFEIKDKKCDVPCIGIEMVHDGGKTTWHSAATAIYCLMGSGQLLWDTVTGVDMAL |
| B/Brisbane/21/2004 | 2004 | LPSTIQTLTLFLTSGGVLLSLYVSASLSYLLYSDILLKFSQTEITAPIMPLDCANASNVQAVNRSAAKGVTLLLPEPEWTYPRLSCPGSTFQKALLISPHRFGETKGNSAPLIIREPFIACGPTECKHFALTHYAAQPGGYYNGTREDRNKLRHLISVKLGKIPTVENSIFHMAAWSGSACHDGKEWTYIGVDGPDSNALLKIKYGEAYTDTYHSYAKNILRTQESACNCIGGDCYLMITDGPASGISECRFLKIREGRIIKEIFPTGRVKHTEECTCGFASNKTIECACRDNSYTAKRPFVKLNVETDTAEIRLMCTETYLDTPRPNDGSITGPCESDGDKGSGGIKGGFVHQRMASKIGRWYSRTMSKTKRMGMGLYVKYDGDPWTDSEALALSGVMVSMEEPGWYSFGFEIKDKKCDVPCIGIEMVHDGGKTTWHSAATAIYCLMGSGQLLWDTVTGVDMAL |
| B/Brisbane/5/2004 | 2004 | LPSTIQTLTLFLTSGGVLLSLYVSASLSYLLYSDILLKFSQTEITAPIMPLDCANASNVQAVNRSATKGVTLLLPEPEWTYPRLSCPGSTFQKALLISPHRFGETKGNSAPLIIREPFIACGPKECKHFALTHYAAQPGGYYNGTREDRNKLRHLISVKLGKIPTVENSIFHMAAWSGSACHDGREWTYIGVDGPDSNALLKIKYGEAYTDTYHSYAKNILRTQESACNCIGGDCYLMITDGPASGISECRFLKIREGRIIKEIFPTGRVKHTEECTCGFASNKTIECACRDNSYTAKRPFVKLNVETDTAEIRLMCTETYLDTPRPNDGSITGPCESNGDKGSGGIKGGFVHQRMASKIGRWYSRTMSKTKRMGMGLYVKYDGDPWTDSEALALSGVMVSMEEPGWYSFGFEIKDKKCDVPCIGIEMVHDGGKTTWHSAATAIYCLMGSGQLLWDTVTGVDMAL |
| B/Brisbane/9/2004 | 2004 | LPSTIQTLTLFLTSGGVLLSLYVSASLSYLLYSDILLKFSQTEITAPIMPLDCANASNVQAVNRSATKGVTLLLPEPEWTYPRLSCPGSTFQKALLISPHRFGETKGNSAPLIIREPFIACGPKECKHFALTHYAAQPGGYYNGTREDRNKLRHLISVKLGKIPTVENSIFHMAAWSGSACHDGREWTYIGVDGPDSNALLKIKYGEAYTDTYHSYAKNILRTQESACNCIGGDCYLMITDGPASGISECRFLKIREGRIIKEIFPTGRVKHTEECTCGFASNKTIECACRDNSYTAKRPFVKLNVETDTAEIRLMCTETYLDTPRPNDGSITGPCESNGDKGSGGIKGGFVHQRMASKIGRWYSRTISKTKRMGMGLYVKYDGDPWTDSEALALSGVMVSMEEPGWYSFGFEIKDKKCDVPCIGIEMVHDGGKTTWHSAATAIYCLMGSGQLLWDTVTGVDMAL |
| B/Christchurch/241/2004 | 2004 | LPSTIQTLTLFLTSGGVLLSLYVSASLSYLLYSDILLKFSTTEITAPTMPLDCANASNVQAVNRSATKGVTFLLPEPEWTYPRLSCPGSTFQKALLISPHRFGETKGNSAPLIIREPFIACGPKECKHFALTHYAAQPGGYYNGTREDRNKLRHLISVKLGKIPTVENSIFHMAAWSGSACHDGKEWTYIGVDGPDSNALLKIKYGEAYTDTYHSYANNILRTQESACNCIGGNCYLMITDGSASGISECRFLKIREGRIIKEIFPTGRVKHTEECTCGFASNKTIECACRDNSYTAKRPFVKLNVETDTAEIRLMCTETYLDTPRPDDGSITGPCESNGNKGSGGIKGGFVHQRMASKIGRWYSRTMSKTKRMGMGLYVKYDGDPWTDSDALALSGVMVSMEEPGWYSFGFEIKDKKCDVPCIGIEMVHDGGKETWHSAATAIYCLMGSGQLLWDTVTGVDMAL |
| B/Christchurch/335/2004 | 2004 | LPSTIQTLTLFLTSGGVLLSLYVSASLSYLLYSDILLKFSQTEITAPTMPLDCANASNVQAVNRSATKGVTLLLPELEWTYPRLSCPGSTFQKALLISPHRFGETKGNSAPLIIREPFIACGPKECKHFALTHYAAQPGGYYNGTREDRNKLRHLISVKLGKIPTVENSIFHMAAWSGSACHDGREWTYVGVDGPDSNALLKIKYGEAYTDTYHSYAKNILRTQESACNCIGGDCYLMITDGPASGISECRFLKIREGRIIKEIFPTGRVKHTEECTCGFASNKTIECACRDNSYTAKRPFVKLDVETDTAEIRLMCTETYLDTPRPNDGSITGPCEANGDKGSGGIKGGFVHQRMASKIGRWYSRTMSKTKRMGMGLYVKYDGDPWTDSEALALSGVMVSMEEPGWYSFGFEIKDKKCDVPCIGIEMVHDGGKTTWHSAATAIYCLMGSGQLLWDTVTGVDMAL |
| B/Christchurch/402/2004 | 2004 | LPSTIQTLTLFLTSGGVLLSLYVSASLSYLLYSDILLKFSTTEITAPTMPLDCANASNVQAVNRSATKGVTFLLPEPEWTYPRLSCPGSTFQKALLISPHRFGETKGNSAPLIIREPFIACGPKECKHFALTHYAAQPGGYYNGTREDRNKLRHLISVKLGKIPTVENSIFHMAAWSGSACHDGKEWTYIGVDGPDSNALLKIKYGEAYTDTYHSYANNILRTQESACNCIGGNCYLMITDGSASGISECRFLKIREGRIIKEIFPTGRVKHTEECTCGFASNKTIECACRDNSYTAKRPFVKLNVETDTAEIRLMCTETYLDTPRPDDGSITGPCESNGNKGSGGIKGGFVHQRMASKIGRWYSRTMSKTKRMGMGLYVKYDGDPWTDSDALALSGVMVSMEEPGWYSFGFEIKDKKCDVPCIGIEMVHDGGKETWHSAATAIYCLMGSGQLLWDTVTGVDMAL |
| B/Colorado/13/2004 | 2004 | LPSTIQTLTLFLTSGGVLLSLYVSASLSYLLYSDILLKFSQTEITAPIMPLDCANASNVQAVNRSATKGVTLLLPEPEWTYPRLSCPGSTFQKALLISPHRFGETKGNSAPLIIREPFIACGPTECKHFALTHYAAQPGGYYNGTREDRNKLRHLISVKLGKIPTVENSIFHMAAWSGSACHDGKEWTYIGVDGPDSNALLKIKYGEAYTDTYHSYAKNILRTQESACNCIGGDCYLMITDGPASGISECRFLKIREGRIIKEIFPTGRVKHTEECTCGFASNKTIECACRDNSYTAKRPFVKLNVETDTAEIRLMCTETYLDTPRPNDGSITGPCESDGDKGSGGIKGGFVHQRMASKIGRWYSRTMSKTKRMGMGLYVKYDGDPWTDSEALALSGVMVSMEEPGWYSFGFEIKDKKCDVPCIGIEMVHDGGKTTWHSAATAIYCLMGSGQLLWDTVTGVDMAL |
| B/Egypt/2040/2004 | 2004 | LPSTIQTLTLFLTSGGVLLSLYVSASLSYLLYSDILLKFSQTEITAPIMPLDCANASNVQAVNRSATKGVTLLLPEPEWTYPRLSCPGSTFQKALLISPHRFGETTGNSAPLIIREPFIACGPKECKHFALTHYAAQPGGYYNGTREDRNKLRHLISVKLGKIPTVENSIFHMAAWSGSACHDGREWTYIGVDGPDNNALLKIKYGEAYTDTYHSYAKNILRTQESACNCIGGDCYLMITDGPASGISECRFLKIREGRIIKEIFPTGRVKHTEECTCGFASNKTIECACRDNSYTAKRPFVKLNVETDTAEIRLMCTETYLDTPRPNDGSITGPCESNGDKGSGGIKGGFVHQRMASKIGRWYSRTMSKTKRMGMGLYVKYDGDPWTDSEALALSGVMVSMEEPGWYSFGFEIKDKKCDVPCIGIEMVHDGGKTTWHSAATAIYCLMGSGQLLWDTVTGVDMAL |
| B/Hawaii/13/2004 | 2004 | LPSTIQTLTLFLTSGGVLLSLYVSASLSYLLYSDILLKFSPTEITAPTMPLDCANASNVQAVNRSATKGVTLLLPEPEWTYPRLSCPGSTFQKALLISPHRFGETKGNSAPLIIREPFIACGPNECKHFALTHYAAQPGGYYNGTRGDRNKLRHLISVKLGKIPTVENSIFHMAAWSGSACHDGKEWTYIGVDGPDNNALLKIKYGEAYTDTYHSYANNILRTQESACNCIGGNCYLMITDGSASGVSECRFLKIREGRIIKEIFPTGRVKHTEECTCGFASNKTIECACRDNSYTAKRPFVKLNVETDTAEIRLMCTETYLDTPRPDDGSITGPCESNGDKGSGGIKGGFVHQRMASKIGRWYSRTMSKTERMGMGLYVKYDGDPWADSDALALIGVMVSMEEPGWYSFGFEIKDKKCDVPCIGIEMVHDGGKETWHSAATAIYCLMGSGQLLWDTVTGVNMAL |
| B/Hawaii/33/2004 | 2004 | LPSTIQTLTLFLTSGGVLLSLYVSASLSYLLYSDILLKFSPTEITAPTMPLDCANASNVQAVNRSATKGVTLLLPEPEWTYPRLSCPGSTFQKALLISPHRFGETKGNSAPLIIREPFIACGPNECKHFALTHYAAQPGGYYNGTRGDRNKLRHLISVKLGKIPTVENSIFHMAAWSGSACHDGKEWTYIGVDGPDNNALLKIKYGEAYTDTYHSYANNILRTQESACNCIGGNCYLMITDGSASGVSECRFLKIREGRIIKEIFPTGRVKHTEECTCGFASNKTIECACRDNSYTAKRPFVKLNVETDTAEIRLMCTETYLDTPRPDDGSITGPCESNGDKGSGGIKGGFVHQRMASKIGRWYSRTMSKTERMGMGLYVKYDGDPWADSDALALIGVMVSMEEPGWYSFGFEIKDKKCDVPCIGIEMVHDGGKETWHSAATAIYCLMGSGQLLWDTVTGVNMAL |
| B/Malaysia/24666/2004 | 2004 | LPSTIQTLTLFLTSGGVLLSLYVSASLSYLLYSDILLKFSQTEITAPTMPLDCANASNVQAVNRSATKGVTLLLPEPEWTYPRLSCPGSTFQKALLISPHRFGETKGNSAPLIIREPFIACGPKECKHFALTHYAAQPGGYYNGTREDRNKLRHLISVKLGKIPTVENSIFHMAAWSGSACHDGREWTYIGVDGPDSNALLKIKYGEAYTDTYHSYAKNILRTQESACNCIGGDCYLMITDGSASGISECRFLKIREGRIIKEIFPTGRVKHTEECTCGFASNKTIECACRDNSYTAKRPFVKLNVETDTAEIRLMCTETYLDTPRPNDGSITGPCESNGDRGGGGIKGGFVHQRMASKIGRWYSRTMSKTKRMGMGLYVKYDGDPWTDSEALALSGVMVPMEEPGWYSFGFEIKDKKCDVPCIGIEMVHDGGKTTWHSAATAIYCLMGSGQLLWDTVTGVDMAL |
| B/Malaysia/29570/2004 | 2004 | LPSTIQTLTLFLTSGGVLLSLYVSASLSYLLYSDILLKFPPTEITAPTMPLDCANASNVQAVNRSATKGVTLLLPEPEWTYPRLSCPGSTFQKALLISPHRFGETKGNSAPLIIREPFIACGPKECKHFALTHYAAQPGGYYNGTRGDRNKLRHLISVKLGKIPTVENSIFHMAAWSGSACHDGKEWTYIGVDGPDNNALLKIKYGEAYTDTYHSYANNILRTQESACNCIGGNCYLMITDGSASGVSECRFLKIREGRIIKEIFPTGRVKHTEECTCGFASNKTIECACRDNSYTAKRPFVKLNVETDTAEIRLMCTETYLDTPRPDDGSITGPCESNGDKGSGGIKGGFVHQRMASKIGRWYSRTMSKTKRMGMGLYVKYDGDPWADSDALAFSGVMVSMEEPGWYSFGFEIKDKKCDVPCIGIEMVHDGGKETWHSAATAIYCLMGSGQLLWDTVTGVNMAL |
| B/Malaysia/30474/2004 | 2004 | LPSTIQTLTLFLTSGGVLLSLYVSASLSYLLYSDILLKFPSTEITAPTMPLDCANASNVQAVNRSATKGVTLLLPEPEWTYPRLSCPGSTFQKALLISPHRFGETKGNSAPLIIREPFIACGPKECKHFALTHYAAQPGGYYNGTRGDRNKLRHLISVKLGKIPTVENSIFHMAAWSGSACHDGKEWTYIGVDGPDNNALLKIKYGEAYTDTYHSYANNILRTQESACNCIGGNCYLMITDGSASGVSECRFLKIREGRIIKEIFPTGRIKHTEECTCGFASNKTIECACRDNSYTAKRPFVKLNVETDTAEIRLMCTETYLDTPRPDDGSITGPCESNGDKGSGGIKGGFVHQRMASKIGRWYSRTMSKTKRMGMGLYVKYDGDPWADSDALAFSGVMVSMEEPGWYSFGFEIKDKKCDVPCIGIEMVHDGGKETWHSAATAIYCLMGSGQLLWDTVTGVNMAL |
| B/New York/1062/2004 | 2004 | LPSTIQTLTLFLTSGGVLLSLYVSASLSYLLYSDILLKFSTTEITAPTMPSDCANASNVQAVNRSATKGVTLLLPEPEWTYPRLSCPGSTFQKALLISPHRFGETKGNSAPLIIREPFIACGPKECKHFALTHYAAQPGGYYNGTREDRNKLRHLISVKLGKIPTVENSIFHMAAWSGSACHDGKEWTYIGVDGPDSNALLKIKYGEAYTDTYHSYANNILRTQESACNCIGGNCYLMITDGSASGISECRFLKIREGRIIKEIFPTGRVKHTEECTCGFASNKTIECACRDNSYTAKRPFVKLNVETDTAEIRLMCTETYLDTPRPDDGSITGPCESNGNKGSGGIKGGFVHQRMASKIGRWYSRTMSKTKRMGMGLYVKYDGDPWTDSDALALSGVMVSMEEPGWYSFGFEIKDKKCDVPCIGIEMVHDGGKETWHSAATAIYCLMGSGQLLWDTVTGVDMAL |
| B/New York/1067/2004 | 2004 | LPSTIQTLTLFLTSGGVLLSLYVSASLSYLLYSDILLKFSQTEITAPIMPLDCANASNVQAVNRSATKGVTLLLPEPEWTYPRLSCPGSTFQKALLISPHRFGETKGNSAPLIIREPFIACGPTECKHFALTHYAAQPGGYYNGTREDRNKLRHLISVKLGKIPTVENSIFHMAAWSGSACHDGKEWTYIGVDGPDSNALLKIKYGEAYTDTYHSYAKNILRTQESACNCIGGDCYLMITDGPASGISECRFLKIREGRIIKEIFPTGRVKHTEECTCGFASNKTIECACRDNSYTAKRPFVKLNVETDTAEIRLMCTETYLDTPRPNDGSITGPCESDGDKGSGGIKGGFVHQRMASKIGRWYSRTMSKTKRMGMGLYVKYDGDPWTDSEALALSGVMVSMEEPGWYSFGFEIKDKKCDVPCIGIEMVHDGGKTTWHSAATAIYCLMGSGQLLWDTVTGVDMAL |
| B/Otago/02/2004 | 2004 | LPSTIQTLTLFLTSGGVLLSLYVSASLSYLLYSDILLKFSTTEITAPTMPLDCANASNVQAVNRSATKGVTFLLPEPEWTYPRLSCPGSTFQKALLISPHRFGETKGNSAPLIIREPFIACGPKECKHFALTHYAAQPGGYYNGTREDRNKLRHLISVKLGKIPTVENSIFHMAAWSGSACHDGKEWTYIGVDGPDSNALLKIKYGEAYTDTYHSYANNILRTQESACNCIGGNCYLMITDGSASGISECRFLKIREGRIIKEIFPTGRVKHTEECTCGFASNKTIECACRDNSYTAKRPFVKLNVETDTAEIRLMCTETYLDTPRPDDGSITGPCESNGNKGSGGIKGGFVHQRMASKIGRWYSRTMSKTKRMGMGLYVKYDGDPWTDSDALALSGVMVSMEEPGWYSFGFEIKDKKCDVPCIGIEMVHDGGKETWHSAATAIYCLMGSGQLLWDTVTGVDMAL |
| B/Otago/03/2004 | 2004 | LPSTIQTLTLFLTSGGVLLSLYVSASLSYLLYSDILLKFSTTEITAPTMPLDCANASNVQAVNRSATKGVTFLLPEPEWTYPRLSCPGSTFQKALLISPHRFGETKGNSAPLIIREPFIACGPKECKHFALTHYAAQPGGYYNGTREDRNKLRHLISVKLGKIPTVENSIFHMAAWSGSACHDGKEWTYIGVDGPDSNALLKIKYGEAYTDTYHSYANNILRTQESACNCIGGNCYLMITDGSASGISECRFLKIREGRIIKEIFPTGRVKHTEECTCGFASNKTIECACRDNSYTAKRPFVKLNVETDTAEIRLMCTETYLDTPRPDDGSITGPCESNGNKGSGGIKGGFVHQRMASKIGRWYSRTMSKTKRMGMGLYVKYDGDPWTDSDALALSGVMVSMEEPGWYSFGFEIKDKKCDVPCIGIEMVHDGGKETWHSAATAIYCLMGSGQLLWDTVTGVDMAL |
| B/Sydney/2/2004 | 2004 | LPSTIQTLTLFLTSGGVLLSLYVSASLSYLLYSDILLKFSQTEIIAPIMPLDCANASNVQAVNHSATKGVTLLLPEPEWTYPRLSCPGSTFQKALLISPHRFGETKGNSAPLIIREPFIACGPKECKHFALTHYAAQPGGYYNGTREDRNKLRHLISVKLGKIPTVENSIFHMAAWSGSACHDGREWTYIGVDGPDSNALLKIKYGEAYTDTYHSYAKNILRTQESACNCIGGDCYLMITDGPASGISECRFLKIREGRIIKEIFPTGRVKHTEECTCGFASNKTIECACRDNSYTAKRPFVKLNVETDTAEIRLMCTETYLDTPRPNDGSITGPCESNGDKGSGGIKGGFVHQRMASKIGRWYSRTMSKTKRMGMGLYVKYDGDPWTDSEALALSGVMVSMEEPGWYSFGFEIKDKKCDVPCIGIEMVHDGGKTTWHSAATAIYCLMGSGQLLWDTVTGVDMAL |
| B/Sydney/203/2004 | 2004 | LPSTIQTLTLFLTSGGVLLSLYVSASLSYLLYSDILLKFSQTEIIAPIMPLDCANASNVQAVNHSATKGVTLLLPEPEWTYPRLSCPGSTFQKALLISPHRFGETKGNSAPLIIREPFIACGPKECKHFALTHYAAQPGGYYNGTREDRNKLRHLISVKLGKIPTVENSIFHMAAWSGSACHDGREWTYIGVDGPDSNALLKIKYGEAYTDTYHSYAKNILRTQESACNCIGGDCYLMITDGPASGISECRFLKIREGRIIKEIFPTGRVKHTEECTCGFASNKTIECACRDNSYTAKRPFVKLNVETDTAEIRLMCTETYLDTPRPNDGSITGPCESNGDKGSGGIKGGFVHQRMASKIGRWYSRTMSKTKRMGMGLYVKYDGDPWTDSEALALSGVMVSMEEPGWYSFGFEIKDKKCDVPCIGIEMVHDGGKTTWHSAATAIYCLMGSGQLLWDTVTGVDMAL |
| B/Sydney/3/2004 | 2004 | LPSTIQTLTLFLTSGGVLLSLYVSASLSYLLYSDILLKFSQTEIIAPIMPLDCANASNVQAVNHSATKGVTLLLPEPEWTYPRLSCPGSTFQKALLISPHRFGETKGNSAPLIIREPFIACGPKECKHFALTHYAAQPGGYYNGTREDRNKLRHLISVKLGKIPTVENSIFHMAAWSGSACHDGREWTYIGVDGPDSNALLKIKYGEAYTDTYHSYAKNILRTQESACNCIGGDCYLMITDGPASGISECRFLKIREGRIIKEIFPTGRVKHTEECTCGFASNKTIECACRDNSYTAKRPFVKLNVETDTAEIRLMCTETYLDTPRPNDGSITGPCESNGDKGSGGIKGGFVHQRMASKIGRWYSRTMSKTKRMGMGLYVKYDGDPWTDSEALALSGVMVSMEEPGWYSFGFEIKDKKCDVPCIGIEMVHDGGKTTWHSAATAIYCLMGSGQLLWDTVTGVDMAL |
| B/Sydney/4/2004 | 2004 | LPSTIQTLTLFLTSGGVLLSLYVSASLSYLLYSDILLKFSTTEITAPTMPLDCANASNVQAVNRSATKGVTFLLPEPEWTYPRLSCPGSTFQKALLISPHRFGETKGNSAPLIIREPFIACGPKECKHFALTHYAAQPGGYYNGTREDRNKLRHLISVKLGKIPTVENSIFHMAAWSGSACHDGKEWTYIGVDGPDSNALLKIKYGEAYTDTYHSYANNILRTQESACNCIGGNCYLMITDGSASGISECRFLKIREGRIIKEIFPTGRVKHTEECTCGFASNKTIECACRDNSYTAKRPFVKLNVETDTAEIRLMCTETYLDTPRPDDGSITGPCESNGNKGSGGIKGGFVHQRMASKIGRWYSRTMSKTKRMGMGLYVKYDGDPWTDSDALALSGVMVSMEEPGWYSFGFEIKDKKCDVPCIGIEMVHDGGKETWHSAATAIYCLMGSGQLLWDTVTGVDMAL |
| B/Sydney/5/2004 | 2004 | LPSTIQTLTLFLTSGGVLLSLYVSASLSYLLYSDILLKFSQTEIIAPIMPLDCANASNVQAVNHSATKGVTLLLPEPEWTYPRLSCPGSTFQKALLISPHRFGETKGNSAPLIIREPFIACGPKECKHFALTHYAAQPGGYYNGTREDRNKLRHLISVKLGKIPTVENSIFHMAAWSGSACHDGREWTYIGVDGPDSNALLKIKYGEAYTDTYHSYAKNILRTQESACNCIGGDCYLMITDGPASGISECRFLKIREGRIIKEIFPTGRVKHTEECTCGFASNKTIECACRDNSYTAKRPFVKLNVETDTAEIRLMCTETYLDTPRPNDGSITGPCESNGDKGSGGIKGGFVHQRMASKIGRWYSRTMSKTKRMGMGLYVKYDGDPWTDSEALALSGVMVSMEEPGWYSFGFEIKDKKCDVPCIGIEMVHDGGKTTWHSAATAIYCLMGSGQLLWDTVTGVDMAL |
| B/Taiwan/125/2004 | 2004 | LPSTIQTLTLFLTSGGVLLSLYVSASLSYLLYSDILLKFSTTEITAPTMPLDCANASNVQAVNRSATKGVTLLLPEPEWTYPRLSCPGSTFQKALLISPHRFGETKGNSAPLIIREPFIACGPKECKHFALTHYAAQPGGYYNGTREDRNKLRHLISVKLGKIPTVENSIFHMAAWSGSACHDGKEWTYIGVDGPDSNALLKIKYGEAYTDTYHSYANNILRTQESACNCIGGNCYLMITDGSASGISECRFLKIREGRIIKEIFPTGRVKHTEECTCGFASNKTIECACRDNSYTAKRPFVKLNVETDTAEIRLMCTETYLDTPRPDDGSITGPCESNGNKGSGGIKGGFVHQRMASKIGRWYSRTMSKTKRMGMGLYVKYDGDPWTDSDALALSGVMVSMEEPGWYSFGFEIKDKKCDVPCIGIEMVHDGGKETWHSAATAIYCLMGSGQLLWDTVTGVDMAL |
| B/Taiwan/2171/2004 | 2004 | LPSTIQTLTLFLTSGGVLLSLYVSASLSYLLYSDILLKFSQTEITAPIMPLDCANASNVQAVNRSATKGVTLLLPEPEWTYPRLSCPGSTFQKALLISPHRFGETKGNSAPLIIREPFIACGPKECKHFALTHYAAQPGGYYNGTREDRNKLRHLISVKLGKIPTVENSIFHMAAWSGSACHDGREWTYIGVDGPDSNALLKIKYGEAYTDTYHSYAKNILRTQESACNCIGGDCYLMITDGPASGISECRFLKIREGRIIKEIFPTGRVKHTEECTCGFASNKTIECACRDNSYTAKRPFVKLNVETDTAEIRLMCTETYLDTPRPNDGSITGPCESNGDKGSGGIKGGFVHQRMASKIGRWYSRTMSKTKRMGMGLYVKYDGDPWTDSEALALSGVMVSMEEPGWYSFGFEIKDKKCDVPCIGIEMVHDGGKTTWHSAATAIYCLMGSGQLLWDTVTGVDMAL |
| B/Taiwan/3395/2004 | 2004 | LPSTIQTLTLFLTSGGVLLSLYVSASLSYLLYSDILLKFSTTEITAPTMPLDCANASNVQAVNRSATKGVTFLLPEPEWTYPRLSCPGSTFQKALLISPHRFGETKGNSAPLIIREPFIACGPKECKHFALTHYAAQPGGYYNGTREDRNKLRHLISVELGKIPTVENSIFHMAAWSGSACHDGKEWTYIGVDGPDSNALLKIKYGEAYTDTYHSYANNILRTQESACNCIGGNCYLMITDGSASGISECRFLKIREGRIIKEIFPTGRVKHTEECTCGFASNKTIECACRDNSYTAKRPFVKLNVETDTAEIRLMCTETYLDTPRPDDGSITGPCESNGNKGSGGIKGGFVHQRMASKIGRWYSRTMSKTKRMGMGLYVKYDGDPWTDSDALALSGVMVSMEEPGWYSFGFEIKDKKCDVPCIGIEMVHDGGKETWHSAATAIYCLMGSGQLLWDTVTGVDMAL |
| B/Taiwan/70303/2004 | 2004 | LPSTIQTLTLFLTSGGVLLSLYVSASLSYLLYSDILLKFPSTEITAPTMPLDCANASNVQAVNRSATKGVTLLLPEPEWTYPRLSCPGSTFQKALLISPHRFGETKGNSAPLIIREPFIACGPKECKHFALTHYAAQPGGYYNGTRGDRNKLRHLISVKLGKIPTVENSIFHMAAWSGSACHDGKEWTYIGVDGPDNNALLKIKYGEAYTDTYHSYANNILRTQESACNCIGGNCYLMITDGSASGVSECRFLKIREGRIIKEIFPTGRIQHTEECTCGFASNKTIECACRDNSYTAKRPFVKLNVETDTAEIRLMCTETYLDTPRPNDGSITGPCESNGDKGSGGIKGGFVHQRMASKIGRWYSRTMSKTKRMGMGLYVKYDGDPWTDSEALALSGVMVSMEEPGWYSFGFEIKDKKCDVPCIGIEMVHDGGKTTWHSAATAIYCLMGSGQLLWDTVTGVNMAL |
| B/Taiwan/72068/2004 | 2004 | LPSTIQTLTLFLTSGGVLLSLYVSASLSYLLYSDILLKFSTTEITAPTIPLDCANASNVQAVNRSATKGVTFLLPEPEWTYPRLSCPGSTFQKALLISPHRFGETKGNSAPLIIREPFIACGPKECKHFALTHYAAQPGGYYNGTREDRNKLRHLISVKLGKIPTVENSIFHMAAWSGSACHDGKEWTYIGVDGPDSNALLKIKYGEAYTDTYHSYANNILRTQESACNCIGGNCYLMITDGSASGISECRFLKIREGRIIKEIFPTGRVKHTEECTCGFASNKTIECACRDNSYTAKRPFVKLNVETDTAEIRLMCTETYLDTPRPDDGSITGPCESNGNKGSGGIKGGFVHQRMASKIGRWYSRTMSKTKRMGMGLYVKYDGDPWTDSDALALSGVMVSMEEPGWYSFGFEIKDKKCDVPCIGIEMVHDGGKETWHSAATAIYCLMGSGQLLWDTVTGVDMAL |
| B/Taiwan/72168/2004 | 2004 | LPSTIQTLTLFLTSGGVLLSLYVSASLSYLLYSDILLKFPSTEITAPTMPLDCANASNVQAVNRSATKGVTLLLPEPEWTYPRLSCPGSTFQKALLISPHRFGETKGNSAPLIIREPFIACGPKECKHFALTHYAAQPGGYYNGTRGDRNKLRHLISVKLGKIPTVENSIFHMAAWSGSACHDGKEWTYIGVDGPDNNALLKIKYGEAYTDTYHSYANNILRTQESACNCIGGNCYLMITDGSASGVSECRFLKIREGRIIKEIFPTGRIQHTEECTCGFASNKTIECACRDNSYTAKRPFVKLNVETDTAEIRLMCTKTYLDTPRPDDGSITGPCESNGGKGSGGIKGGFVHQRMASKIGRWYSRTMSKTERKGMGLYVKYNGDPWADSDALAFSGVMISMEEPGWYSFGFEIKDKKCDVPCIGIEMVHDGGKETWHSAATAIYCLMGSGQLLWDTVTGVNMTL |
| B/Victoria/101/2004 | 2004 | LPSTIQTLTLFLTSGGVLLSLYVSASLSYLLYSDILLKFSQTEITAPIMPLDCANASNVQAVNRSATKGVTLLLPEPEWTYPRLSCPGSTFQKALLISPHRFGETKGNSAPLIIREPFIACGPKECKHFALTHYAAQPGGYYNGTREDRNKLRHLISVKLGKIPTVENSIFHMAAWSGSACHDGREWTYIGVDGPDSNALLKIKYGEAYTDTYHSYAKNILRTQESACNCIGGDCYLMITDGPASGISECRFLKIREGRIIKEIFPTGRVKHTEECTCGFASNKTIECACRDNSYTAKRPFVKLNVETDTAEIRLMCTETYLDTPRPNDGSITGPCESNGDKGSGGIKGGFVHQRMASKIGRWYSRTMSKTKRMGMGLYVKYDGDPWTDSEALALSGVMVSMEEPGWYSFGFEIKDKKCDVPCIGIEMVHDGGKTTWHSAATAIYCLMGSGQLLWDTVTGVDMAL |
| B/Victoria/108/2004 | 2004 | LPSTIQTLTLFLTSGGVLLSLYVSASLSYLLYSDILLKFSQTEITAPIMPLDCANASNVQAVNRSATKGVTLLLPEPEWTYPRLSCPGSTFQKALLISPHRFGETKGNSAPLIIREPFIACGPKECKHFALTHYAAQPGGYYNGTREDRNKLRHLISVKLGKIPTVENSIFHMAAWSGSACHDGREWTYIGVDGPDSNALLKIKYGEAYTDTYHSYAKNILRTQESACNCIGGDCYLMITDGPASGISECRFLKIREGRIIKEIFPTGRVKHTEECTCGFASNKTIECACRDNSYTAKRPFVKLNVETDTAEIRLMCTETYLDTPRPNDGSITGPCESNGDKGSGGIKGGFVHQRMASKIGRWYSRTMSKTKRMGMGLYVKYDGDPWTDSEALALSGVMVSMEEPGWYSFGFEIKDKKCDVPCIGIEMVHDGGKTTWHSAATAIYCLMGSGQLLWDTVTGVDMAL |
| B/Victoria/109/2004 | 2004 | LPSTIQTLTLFLTSGGVLLSLYVSASLSYLLYSDILLKFSQTEITAPIMPLDCANASNVQAVNRSATKGVTLLLPEPEWTYPRLSCPGSTFQKALLISPHRFGETKGNSAPLIIREPFIACGPKECKHFALTHYAAQPGGYYNGTREDRNKLRHLISVKLGKIPTVENSIFHMAAWSGSACHDGREWTYIGVDGPDSNALLKIKYGEAYTDTYHSYAKNILRTQESACNCIGGDCYLMITDGPASGISECRFLKIREGRIIKEIFPTGRVKHTEECTCGFASNKTIECACRDNSYTAKRPFVKLNVETDTAEIRLMCTETYLDTPRPNDGSITGPCESNGDKGSGGIKGGFVHQRMASKIGRWYSRTMSKTKRMGMGLYVKYDGDPWTDSEALALSGVMVSMEEPGWYSFGFEIKDKKCDVPCIGIEMVHDGGKTTWHSAATAIYCLMGSGQLLWDTVTGVDMAL |
| B/Victoria/110/2004 | 2004 | LPSTIQTLTLFLTSGGVLLSLYVSASLSYLLYSDILLKFSQTEITAPIMPLDCANASNVQAVNRSATKGVTLLLPEPEWTYPRLSCPGSTFQKALLISPHRFGETKGNSAPLIIREPFIACGPKECKHFALTHYAAQPGGYYNGTREDRNKLRHLISVKLGKIPTVENSIFHMAAWSGSACHDGREWTYIGVDGPDSNALLKIKYGEAYTDTYHSYAKNILRTQESACNCIGGDCYLMITDGPASGISECRFLKIREGRIIKEIFPTGRVKHTEECTCGFASNKTIECACRDNNYTAKRPFVKLNVETDTAEIRLMCTETYLDTPRPNDGSITGPCESNGDKGSGGIKGGFVHQRMASKIGRWYSRTMSKTKRMGMGLYVKYDGDPWTDSEALALSGVMVSMEEPGWYSFGFEIKDKKCDVPCIGIEMVHDGGKTTWHSAATAIYCLMGSGQLLWDTVTGVDMAL |
| B/Victoria/113/2004 | 2004 | LPSTIQTLTLFLTSGGVLLSLYVSASLSYLLYSDILLKFSQTEITAPIMPLDCANASNVQAVNRSATKGVTLLLPEPEWTYPRLSCPGSTFQKALLISPHRFGETKGNSAPLIIREPFIACGPKECKHFALTHYAAQPGGYYNGTREDRNKLRHLISVKLGKIPTVENSIFHMTAWSGSACHDGKEWTYIGVDGPDSNALLKIKYGEAYTDTYHSYAKNILRTQESACNCIGGDCYLMITDGPASGISECRFLKIREGRIIKEIFPTGRVKHTEECTCGFASNKTIECACRDNSYTAKRPFVKLNVETDTAEIRLMCTETYLDTPRPNDGSITGPCESDGDKGSGGIKGGFVHQRMATKIGRWYSRTMSKTKRMGMGLYVKYDGDPWTDSEALALSGVMVSMEEPGWYSFGFEIKDKKCDVPCIGIEMVHDGGKTTWHSAATAIYCLMGSGQLLWDTVTGVDMAL |
| B/Victoria/302/2004 | 2004 | LPSTIQTLTLFLTSGGVLLSLYVSASLSYLLYSDILLKFSQTEITAPIMPLDCANASNVQAVNRSATKGVTLLLPEPEWTYPRLSCPGSTFQKALLISPHRFGETKGNSAPLIIREPFIACGPKECKHFALTHYAAQPGGYYNGTREDRNKLRHLISVKLGKIPTVENSIFHMAAWSGSACHDGREWTYIGVDGPDSNALLKIKYGEAYTDTYHSYAKNILRTQESACNCIGGDCYLMITDGPASGISECRFLKIREGRIIKEIFPTGRVKHTEECTCGFASNKTIECACRDNNYTAKRPFVKLNVETDTAEIRLMCTETYLDTPRPNDGSITGPCESNGDKGSGGIKGGFVHQRMASKIGRWYSRTMSKTKRMGMGLYVKYDGDPWTDSEALALSGVMVSMEEPGWYSFGFEIKDKKCDVPCIGIEMVHDGGKTTWHSAATAIYCLMGSGQLLWDTVTGVDMAL |
| B/Victoria/304/2004 | 2004 | LPSTIQTLTLFLTSGGVLLSLYVSASLSYLLYSDILLKFSQTEITAPIMPLDCANASNVQAVNRSATKGVTLLLPEPEWTYPRLSCPGSTFQKALLISPHRFGETKGNSAPLIIREPFIACGPKECKHFALTHYAAQPGGYYNGTREDRNKLRHLISVKLGKIPTVENSIFHMAAWSGSACHDGREWTYIGVDGPDSNALLKIKYGEAYTDTYHSYAKNILRTQESACNCIGGDCYLMITDGPASGISECRFLKIREGRIIKEIFPTGRVKHTEECTCGFASNKTIECACRDNNYTAKRPFVKLNVETDTAEIRLMCTETYLDTPRPNDGSITGPCESNGDKGSGGIKGGFVHQRMASKIGRWYSRTMSKTKRMGMGLYVKYDGDPWTDSEALALSGVMVSMEEPGWYSFGFEIKDKKCDVPCIGIEMVHDGGKTTWHSAATAIYCLMGSGQLLWDTVTGVDMAL |
| B/Victoria/305/2004 | 2004 | LPSTIQTLTLFLTSGGVLLSLYVSASLSYLLYSDILLKFSQTEITAPIMPLDCANASNVQAVNRSATKGVTLLLPEPEWTYPRLSCPGSTFQKALLISPHRFGETKGNSAPLIIREPFIACGPKECKHFALTHYAAQPGGYYNGTREDRNKLRHLISVKLGKIPTVENSIFHMAAWSGSACHDGREWTYIGVDGPDSNALLKIKYGEAYTDTYHSYAKNILRTQESACNCIGGDCYLMITDGPASGISECRFLKIREGRIIKEIFPTGRVKHTEECTCGFASNKTIECACRDNNYTAKRPFVKLNVETDTAEIRLMCTETYLDTPRPNDGSITGPCESNGDKGSGGIKGGFVHQRMASKIGRWYSRTMSKTKRMGMGLYVKYDGDPWTDSEALALSGVMVSMEEPGWYSFGFEIKDKKCDVPCIGIEMVHDGGKTTWHSAATAIYCLMGSGQLLWDTVTGVDMAL |
| B/Victoria/508/2004 | 2004 | LPSTIQTLTLFLTSGGVLLSLYVSASLSYLLYSDILLKFSTTEITAPTMPLDCANASNVQAVNRSATKGVTFLLPEPEWTYPRLSCPGSTFQKALLISPHRFGETKGNSAPLIIREPFIACGPKECKHFALTHYAAQPGGYYNGTREDRNKLRHLISVKLGKIPTVENSIFHMAAWSGSACHDGKEWTYIGVDGPDSNALLKIKYGEAYTDTYHSYANNILRTQESACNCIGGNCYLMITDGSASGISECRFLKIREGRIIKEIFPTGRVKHTEECTCGFASNKTIECACRDNSYTAKRPFVKLNVETDTAEIRLMCTETYLDTPRPDDGSITGPCESNGNKGSGGIKGGFVHQRMASKIGRWYSRTMSKTKRMGMGLYVKYDGDPWTDSDALALSGVMVSMEEPGWYSFGFEIKDKKCDVPCIGIEMVHDGGKETWHSAATAIYCLMGSGQLLWDTVTGVDMAL |
| B/Victoria/515/2004 | 2004 | LPSTIQTLTLFLTSGGVLLSLYVSASLSYLLYSDILLKFSTTEITAPTIPLDCANASNVQAVNRSATKGVTLLLPEPEWTYPRLSCPGSTFQKALLISPHRFGETKGNSAPLIIREPFIACGPKECKHFALTHYAAQPGGYYNGTREDRNKLRHLISVKLGKIPTVENSIFHMAAWSGSACHDGKEWTYIGVDGPDSNALLKIKYGEAYTDTYHSYANNILRTQESACNCIGGNCYLMITDGSASGISECRFLKIREGRIIKEIFPTGRVKHTEECTCGFASNKTIECACRDNSYTAKRPFVKLNVETDTAEIRLMCTETYLDTPRPDDGSITGPCESNGNKGSGGIKGGFVHQRMASKIGRWYSRTMSKTKRMGMGLYVKYDGDPWTDSDALALSGVMVSMEEPGWYSFGFEIKDKKCDVPCIGIEMVHDGGKETWHSAATAIYCLMGSGQLLWDTVTGVDMAL |
| B/Victoria/517/2004 | 2004 | LPSTIQTLTLFLTSGGVLLSLYVSASLSYLLYSDILLKFSQTEITAPIMPLDCANASNVQAVNRSATKGVTLLLPEPEWTYPRLSCPGSTFQKALLISPHRFGETKGNSAPLIIREPFIACGPKECKHFALTHYAAQPGGYYNGTREDRNKLRHLISVKLGKIPTVENSIFHMAAWSGSACHDGREWTYIGVDGPDSNALLKIKYGEAYTDTYHSYAKNILRTQESACNCIGGDCYLMITDGPASGISECRFLKIREGRIIKEIFPTGRVKHTEECTCGFASNKTIECACRDNNYTAKRPFVKLNVETDTAEIRLMCTETYLDTPRPNDGSITGPCESNGDKGSGGIKGGFVHQRMASKIGRWYSRTMSKTKRMGMGLYVKYDGDPWTDSEALALSGVMVSMEEPGWYSFGFEIKDKKCDVPCIGIEMVHDGGKTTWHSAATAIYCLMGSGQLLWDTVTGVDMAL |
| B/Waikato/01/2004 | 2004 | LPSTIQTLTLFLTSGGVLLSLYVSASLSYLLYSDILLKFSQTEITAPIMPLDCANTSNIQAVNRSATKGVTLLLPEPEWTYPRLSCPGSTFQKALLISPHRFGETKGNSAPLIIREPFIACGPKECKHFALTHYAAQPGGYYNGTREDRNKLRHLISVKLGKIPTVENSIFHMAAWSGSACHDGREWTYIGVDGPDSNALLKIKYGEAYTDTYHSYAKNILRTQESACNCIGGDCYLMITDGPASGISECRFLKIREGRIIKEIFPTGRVKHTEECTCGFASNKTIECACRDNSYTAKRPFVKLNVETDTAEIRLMCTETYLDTPRPNDGSITGPCESNGDKGSGGIKGGFVHQRMASKIGRWYSRTMSKTKRMGMGLYVKYDGDPWTDSEALALSGVMVSMEEPGWYSFGFEIKDKKCDVPCIGIEMVHDGGKTTWHSAATAIYCLMGSGQLLWDTVTGVDMAL |
| B/Waikato/02/2004 | 2004 | LPSTIQTLTLFLTSGGVLLSLYVSASLSYLLYSDILLKFSTTEITAPTMPLDCANASNVQAVNRSATKGVTFLLPEPEWTYPRLSCPGSTFQKALLISPHRFGETKGNSAPLIIREPFIACGPKECKHFALTHYAAQPGGYYNGTREDRNKLRHLISVKLGKIPTVENSIFHMAAWSGSACHDGKEWTYIGVDGPDSNALLKIKYGEAYTDTYHSYANNILRTQESACNCIGGNCYLMITDGSASGISECRFLKIREGRIIKEIFPTGRVKHTEECTCGFASNKTIECACRDNSYTAKRPFVKLNVETDTAEIRLMCTETYLDTPRPDDGSITGPCESNGNKGSGGIKGGFVHQRMASKIGRWYSRTMSKTKRMGMGLYVKYDGDPWTDSDALALSGVMVSMEEPGWYSFGFEIKDKKCDVPCIGIEMVHDGGKETWHSAATAIYCLMGSGQLLWDTVTGVDMAL |
| B/Wellington/01/2004 | 2004 | LPSTIQTLTLFLTSGGVLLSLYVSASLSYLLYSDILLKFSTTEITAPTMPLDCANASNVQAVNRSATKGVTFLLPEPEWTYPRLSCPGSTFQKALLISPHRFGETKGNSAPLIIREPFIACGPKECKHFALTHYAAQPGGYYNGTREDRNKLRHLISVKLGKIPTVENSIFHMAAWSGSACHDGKEWTYIGVDGPDSNALLKIKYGEAYTDTYHSYANNILRTQESACNCIGGNCYLMITDGSASGISECRFLKIREGRIIKEIFPTGRVKHTEECTCGFASNKTIECACRDNSYTAKRPFVKLNVETDTAEIRLMCTETYLDTPRPDDGSITGPCESNGNKGSGGIKGGFVHQRMASKIGRWYSRTMSKTKRMGMGLYVKYDGDPWTDSDALALSGVMVSMEEPGWYSFGFEIKDKKCDVPCIGIEMVHDGGKETWHSAATAIYCLMGSGQLLWDTVTGVDMAL |
| B/Wellington/02/2004 | 2004 | LPSTIQTLTLFLTSGGVLLSLYVSASLSYLLYSDILLKFSQTEIIAPIMPLDCANASNVQAVNHSATKGVTLLLPEPEWTYPRLSCPGSTFQKALLISPHRFGETKGNSAPLIIREPFIACGPKECKHFALTHYAAQPGRYYNGTREDRNKLRHLISVKLGKIPTVENSIFHMAAWSGSACHDGREWTYIGVDGPDSNALLKIKYGEAYTDTYHSYAKNILRTQESACNCIGGDCYLMITDGPASGISECRFLKIREGRIIKEIFPTGRVKHTEECTCGFASNKTIECACRDNSYTAKRPFVKLNVETDTAEIRLMCTETYLDTPRPNDGSITGPCESNGDKGSGGIKGGFVHQRMASKIGRWYSRTMSKTKRMGMGLYVKYDGDPWTDSEALALSGVMVSMEEPGWYSFGFEIKDKKCDVPCIGIEMVHDGGKTTWHSAATAIYCLMGSGQLLWDTVTGVDMAL |
| B/Wellington/05/2004 | 2004 | LPSTIQTLTLFLTSGGVLLSLYVSASLSYLLYSDILLKFSQTEITAPIMPLDCANASNVQAVNRSATKGVTLLLPEPEWTYPRLSCPGSTFQKALLISPHRFGETKGNSAPLIIREPFIACGPKECKHFALTHYAAQPGGYYNGTREDRNKLRHLISVKLGKIPTVENSIFHMAAWSGSACHDGREWTYIGVDGPDSNALLKIKYGEAYTDTYHSYAKNILRTQESACNCIGGDCYLMITDGPASGISECRFLKIREGRIIKEIFPTGRVKHTEECTCGFASNKTIECACRDNNYTAKRPFVKLNVETDTAEIRLMCTETYLDTPRPNDGSITGPCESNGDKGSGGIKGGFVHQRMASKIGRWYSRTMSKTKRMGMGLYVKYDGDPWTDSEALALSGVMVSMEEPGWYSFGFEIKDKKCDVPCIGIEMVHDGGKTTWHSAATAIYCLMGSGQLLWDTVTGVDMAL |
| B/Arizona/162/2005 | 2005 | LPSTIQTLTLFLTSGGVLLSLYVSASLSYLLYSDILLKFSPTEITAPTMPLDCANASNVQALNRSATKGVTLLLPEPEWTYPRLSCPGSTFQKALLISPHRFGETKGNSAPLIIREPFIACGPNECKHFALTHYAAQPGGYYNGTRGDRNKLRHLISVKLGKIPTVENSIFHMAAWSGSACHDGKEWTYIGVDGPDNNALLKIKYGEAYTDTYHSYANNILRTQESACNCIGGNCYLMITDGSASGVSECRFLKIREGRIIKEIFPTGRVKHTEECTCGFASNKTIECACRDNSYTAKRPFVKLNVETDTAEIRLMCTDTYLDTPRPDDGSITGPCESNGDKGSGGIKGGFVHQRMASKVGRWYSRTMSKTERMGMGLYVKYDGDPWADSDALAFSGVMVSMKEPGWYSFGFEIKDKKCDVPCIGIEMVHDGGKETWHSAATAIYCLMGSGQLLWDTVTGVDMAL |
| B/Auckland/107/2005 | 2005 | LPSTIQTLTLFLTSGGVLLSLYVSASLSYLLYSDILLKFSQTEITAPIMPLDCANASNVQAVNRSAAKGVTLLLPEPEWTYPRLSCPGSTFQKALLISPHRFGETKGNSAPLIIREPFIACGPTECKHFALTHYAAQPGGYYNGTREDRNKLRHLISVKLGKIPTVENSIFHMAAWSGSACHDGKEWTYIGVDGPDSNALLKIKYGEAYTDTYHSYAKNILRTQESACNCIGGDCYLMITDGPASGISECRFLKIREGRIIKEIFPTGRVKHTEECTCGFASNKTIECACRDNSYTAKRPFVKLNVETDTAEIRLMCTETYLDTPRPNDGSITGPCESDGDKGSGGIKGGFVHQRMASKIGRWYSRTMSKTKRMGMGLYVKYDGDPWTDSEALALSGVMVSMEEPGWYSFGFEIKDKKCDVPCIGIEMVHDGGKTTWHSAATAIYCLMGSGQLLWDTVTGVDMAL |
| B/Auckland/21/2005 | 2005 | LPSTIQTLTLFLTSGGVLLSLYVSASLSYLLYSDILLKFSPTEITAPTMPLDCANASNVHAVNRSATKGVTLLLPEPEWTYPRLSCPGSTFQKALLISPHRFGETKGNSAPLIIREPFIACGPNECKHFALTHYAAQPGGYYNGTRGDRNKLRHLISVKLGKIPTVENSIFHMAAWSGSACHDGKEWTYIGVDGPDNNALLKIKYGEAYTDTYHSYANNILRTQESACNCIGGNCYLMITDGSASGVSECRFLKIREGRIIKEIFPTGRVKHTEECTCGFASNKTIECACRDNSYTAKRPFVKLNVETDTAEIRLMCTDTYLDTPRPDDGSITGPCESNGDKGSGGIKGGFVHQRMASKIGRWYSRTMSKTERMGMGLYVKYDGDPWADSDALAFSGVMVSMKEPGWYSFGFEIKDKKCDVPCIGIEMVHDGGKETWHSAATAIYCLMGSGQLLWDTVTGVDMAL |
| B/Auckland/98/2005 | 2005 | LPSTIQTLTLFLTSGGVLLSLYVSASLSYLLYSDILLKFSPTEITAPTMPLDCANASNVHAVNRSATKGVTLLLPEPEWTYPRLSCPGSTFQKALLISPHRFGETKGNSAPLIIREPFIACGPNECKHFALTHYAARPGGYYNGTRGDRNKLRHLISVKLGKIPTVENSIFHMAAWSGSACHDGKEWTYIGVDGPDNNALLKIKYGEAYTDTYHSYANNILRTQESACNCIGGNCYLMITDGSASGVSECRFLKIREGRIIKEIFPTGRVKHTEECTCGFASNKTIECACRDNSYTAKRPFVKLNVETDTAEIRLMCTDTYLDTPRPDDGSITGPCESNGDKGSGGIKGGFVHQRMASKIGRWYSRTMSKTERMGMGLYVKYDGDPWADSDALAFSGVMVSMKEPGWYSFGFEIKDKKCDVPCIGIEMVHDGGKETWHSAATAIYCLMGSGQLLWDTVTGVDMAL |
| B/Auckland/99/2005 | 2005 | LPSTIQTLTLFLTSGGVLLSLYVSASLSYLLYSDILLKFSQTEITAPIMPLDCANASNVQAVNRSAAKGVTLLLPEPEWTYPRLSCPGSTFQKALLISPHRFGETKGNSAPLIIREPFIACGPTECKHFALTHYAAQPGGYYNGTREDRNKLRHLISVKLGKIPTVENSIFHMAAWSGSACHDGKEWTYIGVDGPDSNALLKIKYGEAYTDTYHSYAKNILRTQESACNCIGGDCYLMITDGPASGISECRFLKIREGRIIKEIFPTGRVKHTEECTCGFASNKTIECACRDNSYTAKRPFVKLNVETDTAEIRLMCTETYLDTPRPNDGSITGPCESDGDKGSGGIKGGFVHQRMASKIGRWYSRTMSKTKRMGMGLYVKYDGDPWTDSEALALSGVMVSMEEPGWYSFGFEIKDKKCDVPCIGIEMVHDGGKTTWHSAATAIYCLMGSGQLLWDTVTGVDMAL |
| B/Brisbane/100/2005 | 2005 | LPSTIQTLTLFLTSGGVLLSLYVSASLSYLLYSDILLKFSQTEITAPIMPLDCANASNVQAVNRSAAKGVTLLLPEPEWTYPRLSCPGSTFQKALLISPHRFGETKGNSAPLIIREPFIACGPTECKHFALTHYAAQPGGYYNGTREDRNKLRHLISVKLGKIPTVENSIFHMAAWSGSACHDGKEWTYIGVDGPDSNALLKIKYGEAYTDTYHSYAKNILRTQESACNCIGGDCYLMITDGPASGISECRFLKIREGRIIKEIFPTGRVKHTEECTCGFASNKTIECACRDNSYTAKRPFVKLNVETDTAEIRLMCTETYLDTPRPNDGSITGPCESDGDKGSGGIKGGFVHQRMASKIGRWYSRTMSKTKRMGMGLYVKYDGDPWTDSEALALSGVMVSMEEPGWYSFGFEIKDKKCDVPCIGIEMVHDGGKTTWHSAATAIYCLMGSGQLLWDTVTGVDMAL |
| B/Brisbane/11/2005 | 2005 | LPSTIQTLTLFLTSGGVLLSLYVSASLSYLLYSDILLKFSPTEITAPTMPLDCANASNVQAVNRSATKGVTLLLPEPEWTYPRLSCPGSTFQKALLISPHRFGETKGNSAPLIIREPFIACGPNECKHFALTHYAAQPGGYYNGTRGDRNKLRHLISVKLGKIPTVENSIFHMAAWSGSACHDGKEWTYIGVDGPDNNALLKIKYGEAYTDTYHSYANNILRTQESACNCIGGNCYLMITDGSASGVSECRFLKIREGRIIKEIFPTGRVKHTEECTCGFASNKTIECACRDNSYTAKRPFVKLNVETDTAEIRLMCTDTYLDTPRPDDGSITGPCESNGDKGSGGIKGGFVHQRMASKVGRWYSRTMSKTERMGMGLYVKYDGDPWADSDALAFSGVMVSMKEPGWYSFGFEIKDKKCDVPCIGIEMVHDGGKETWHSAATAIYCLMGSGQLLWDTVTGVDMAL |
| B/Brisbane/2/2005 | 2005 | LPSTIQTLTLFLTSGGVLLSLYVSASLSYLLYSDILLKFSQTEITAPIMPLDCANASNVQAVNRSAAKGVTLLLPEPEWTYPRLSCPGSTFQKALLISPHRFGETKGNSAPLIIREPFIACGPTECKHFALTHYAAQPGGYYNGTREDRNKLRHLISVKLGKIPTVENSIFHMAAWSGSACHDGKEWTYIGVDGPDSNALLKIKYGEAYTDTYHSYAKNILRTQESACNCIGGDCYLMITDGPASGISECRFLKIREGRIIKEIFPTGRVKHTEECTCGFASNKTIECACRDNSYTAKRPFVKLNVETDTAEIRLMCTETYLDTPRPNDGSITGPCESDGDKGSGGIKGGFVHQRMASKIGRWYSRTMSKTKRMGMGLYVKYDGDPWTDSEALALSGVMVSMEEPGWYSFGFEIKDKKCDVPCIGIEMVHDGGKTTWHSAATAIYCLMGSGQLLWDTVTGVDMAL |
| B/Brisbane/3/2005 | 2005 | LPSTIQTLTLFLTSGGVLLSLYVSASLSYLLYSDILLKFSQTEITAPIMPLDCANASNVQAVNRSAAKGVALLLPEPEWTYPRLSCPGSTFQKALLISPHRFGETKGNSAPLIIREPFIACGPTECKHFALTHYAAQPGGYYNGTREDRNKLRHLISVKLGKIPTVENSIFHMAAWSGSACHDGKEWTYIGVDGPDSNALLKIKYGEAYTDTYHSYAKNILRTQESACNCIGGDCYLMITDGPASGISECRFLKIREGRIIKEIFPTGRVKHTEECTCGFASNKTIECACRDNSYTAKRPFVKLNVETDTAEIRLMCTETYLDTPRPNDGSITGPCESDGDKGSGGIKGGFVHQRMASKIGRWYSRTMSKTKRMGMGLYVKYDGDPWTDSEALALSGVMVSMEEPGWYSFGFEIKDKKCDVPCIGIEMVHDGGKTTWHSAATAIYCLMGSGQLLWDTVTGVDMAL |
| B/Brisbane/42/2005 | 2005 | LPSTIQTLTLFLTSGGVLLSLYVSASLSYLLYSDILLKFSQTEITAPIMPLDCANASNVQAVNRSAAKGVTLLLPEPEWAYPRLSCPGSTFQKALLISPHRFGETKGNSAPLIIREPFIACGPTECKHFALTHYAAQPGGYYNGTREDRNKLRHLISVKLGKIPTVENSIFHMAAWSGSACHDGKEWTYIGVDGPDSNALLKIKYGEAYTDTYHSYAKNILRTQESACNCIGGDCYLMITDGPASGISECRFLKIREGRIIKEIFPTGRVKHTEECTCGFASNKTIECACRDNSYTAKRPFVKLNVETDTAEIRLMCTETYLDTPRPNDGSITGPCESDGDKGSGGIKGGFVHQRMASKIGRWYSRTMSKTKRMGMGLYVKYDGDPWTDSEALALSGVMVSMEEPGWYSFGFEIKDKKCDVPCIGIEMVHDGGKTTWHSAATAIYCLMGSGQLLWDTVTGVDMAL |
| B/Brisbane/6/2005 | 2005 | LPSTIQTLTLFLTSGGVLLSLYVSASLSYLLYSDILLKFSPTEITAPTMPLDCANASNVQAVNRSATKGVTLLLPEPEWTYPRLSCPGSTFQKALLISPHRFGETKGNSAPLIIREPFIACGPNECKHFALTHYAAQPGGYYNGTRGDRNKLRHLISVKLGKIPTVENSIFHMAAWSGSACHDGKEWTYIGVDGPDNNALLKIKYGEAYTDTYHSYANNILRTQESACNCIGGNCYLMITDGSASGVSECRFLKIREGRIIKEIFPTGRVKHTEECTCGFASNKTIECACRDNSYTAKRPFVKLNVETDTAEIRLMCTDTYLDTPRPDDGSITGPCESNGDKGSGGIKGGFVHQRMASKVGRWYSRTMSKTERMGMGLYVKYDGDPWADSDALAFSGVMVSMKEPGWYSFGFEIKDKKCDVPCIGIEMVHDGGKETWHSAATAIYCLMGSGQLLWDTVTGVDMAL |
| B/Brisbane/65/2005 | 2005 | LPSTIQTLTLFLTSGGVLLSLYVSASLSYLLYSDILLKLSPTEITAPTMPLDCANTSNVQAVNRSATKGVTLLLPEPEWTYPRLSCPGSTFQKALLISPHRFGETKGNSAPLIIREPFIACGPNECKHFALTHYAAQPGGYYNRTRGDRNKLRHLISVKLGKIPTVENSIFHMAAWSGSACHDGKEWTYIGVDGPDNNALLKIKYGEAYTDTYHSYANNILRTQESACNCIGGNCYLMITDGSASGVSECRFLKIREGRIIKEIFPTGRVKHTEECTCGFASNKTIECACRDNSYTAKRPFVKLNMETDTAEIRLMCTDTYLDTPRPDDGSITGPCESNGDKGSGGIKGGFVHQRMASKIGRWYSRTMSKTERMGMGLYVKYDGDPWADSDALAFSGVMVSMKEPGWYSFGFEIKDKKCDVPCIGIEMVHDGGKETWHSAATAIYCLMGSGQLLWDTVTGVDMAL |
| B/Brisbane/95/2005 | 2005 | LPSTIQTLTLFLTSGGVLLSLYVSASLSYLLYSDILLKFSQTEITAPIMPLDCANASNVQAVNRSAAKGVTLLLPEPEWTYPRLSCPGSTFQKALLISPHRFGETKGNSAPLIIREPFIACGPTECKHFALTHYAAQPGEYYNGTREDRNKLRHLISVKLGKIPTVENSIFHMAAWSGSACHDGKEWTYIGVDGPDSNALLKIKYGEAYTDTYHSYAKNILRTQESACNCIGGDCYLMITDGPSSGISECRFLKIREGRIIKEIFPTGRVKHTEECTCGFASNKTIECACRDNSYTAKRPFVKLNVETDTAEIRLMCTETYLDTPRPNDGSITGPCESDGDKGSGGIKGGFVHQRMASKIGRWYSRTMSKTKRMGMGLYVKYDGDPWTDSEALALSGVMVSMEEPGWYSFGFEIKDKKCDVPCIGIEMVHDGGKTTWHSAATAIYCLMGSGQLLWDTVTGVDMAL |
| B/California/NHRC0004/2005 | 2005 | LPSTIQTLTLFLTSGGVLLSLYVSASLSYLLYSDILLKFSQTEITAPIMPLDCANASNVQAVNRSATKGVTLLLPEPEWTYPRLSCPGSTFQKALLISPHRFGETKGNSAPLIIREPFIACGPTECKHFALTHYAAQPGGYYNGTREDRNKLRHLISVKLGKIPTVENSIFHMAAWSGSACHDGKEWTYIGVDGPDSNALLKIKYGEAYTDTYHSYAKNILRTQESACNCIGGDCYLMITDGPASGISECRFLKIREGRIIKEIFPTGRVKHTEECTCGFASNKTIECACRDNSYTAKRPFVKLNVETDTAEIRLMCTETYLDTPRPNDGSITGPCESDGDKGSGGIKGGFVHQRMASKIGRWYSRTMSKTKRMGMGLYVKYDGDPWTDSEALALSGVMVPMEEPGWYSFGFEIKDKKCDVPCIGIEMVHDGGKTTWHSAATAIYCLMGSGQLLWDTVTGVDMAL |
| B/California/NHRC0008/2005 | 2005 | LPSTIQTLTLFLTSGGVLLSLYVSASLSYLLYSDILLKFSQTEITAPIMPLDCANASNVQAVNRSAAKGVTLLLPEPEWTYPRLSCPGSTFQKALLISPHRFGETKGNSAPLIIREPFIACGPTECKHFALTHYAAQPGGYYNGTREDRNKLRHLISVKLGKIPTVENSIFHMAAWSGSACHDGKEWTYIGVDGPDSNALLKIKYGEAYTDTYHSYAKNILRTQESACNCIGGDCYLMITDGPASGISECRFLKIREGRIIKEIFPTGRVKHTEECTCGFASNKTIECACRDNSYTAKRPFVKLNVETDTAEIRLMCTETYLDTPRPNDGSITGPCESDGDKGSGGIKGGFVHQRMASKIGRWYSRTMSKTKRMGMGLYVKYDGDPWTDSEALALSGVMVSMEEPGWYSFGFEIKDKKCDVPCIGIEMVHDGGKTTWHSAATAIYCLMGSGQLLWDTVTGVDMAL |
| B/Christchurch/243/2005 | 2005 | LPSTIQTLTLFLTSGGVLLSLYVSASLSYLLYSDILLKFSQTEITAPIMPLDCANASNVQAVNRSAAKGVTLLLPEPEWTYPRLSCPGSTFQKALLISPHRFGETKGNSAPLIIREPFIACGPTECKHFALTHYAAQPGGYYNGTREDRNKLRHLISVKLGKIPTVENSIFHMAAWSGSACHDGKEWTYIGVDGPDSNALLKIKYGEAYTDTYHSYAKNILRTQESACNCIGGDCYLMITDGPASGISECRFLKIREGRIIKEIFPTGRVKHTEECTCGFASNKTIECACRDNSYTAKRPFVKLNVETDTAEIRLMCTETYLDTPRPNDGSITGPCESDGDKGSGGIKGGFVHQRMASKIGRWYSRTMSKTKRMGMGLYVKYDGDPWTDSEALALSGVMVSMEEPGWYSFGFEIKDKKCDVPCIGIEMVHDGGKTTWHSAATAIYCLMGSGQLLWDTVTGVDMAL |
| B/Christchurch/28/2005 | 2005 | LPSTIQTLTLFLTSGGVLLSLYVSASLSYLLYSDILLKFSQTEITAPIMPLDCANASNVQAVNRSAAKGVTLLLPEPEWTYPRLSCPGSTFQKALLISPHRFGETKGNSAPLIIREPFIACGPTECKHFALTHYAAQPGGYYNGTREDRNKLRHLISVKLGKIPTVENSIFHMAAWSGSACHDGKEWTYIGVDGPDSNALLKIKYGEAYTDTYHSYAKNILRTQESACNCIGGDCYLMITDGPASGISECRFLKIREGRIIKEIFPTGRVKHTEECTCGFASNKTIECACRDNSYTAKRPFVKLNVETDTAEIRLMCTETYLDTPRPNDGSITGPCESDGDKGSGGIKGGFVHQRMASKIGRWYSRTMSKTKRMGMGLYVKYDGDPWTDSEALALSGVMVSMEEPGWYSFGFEIKDKKCDVPCIGIEMVHDGGKTTWHSAATAIYCLMGSGQLLWDTVTGVDMAL |
| B/Christchurch/4/2005 | 2005 | LPSTIQTLTLFLTSGGVLLSLYVSASLSYLLYSDILQKFSQTEITAPIMPLDCANASNVQAVNRSATKGVTLLLPEPEWTYPRLSCPGSTFQKALLISPHRFGETKGNSAPLIIREPFIACGPTECKHFALTHYAAQPGGYYNGTREDRNKLRHLISVKLGKIPTVENSIFHMAAWSGSACHDGKEWTYIGVDGPDSNALLKIKYGEAYTDTYHSYAKNILRTQESACNCIGGDCYLMITDGPASGISECRFLKIREGRIIKEIFPTGRVKHTEECTCGFASNKTIECACRDNSYTAKRPFVKLNVETDTAEIRLMCTETYLDTPRPNDGSITGPCESDGDKGSGGIKGGFVHQRMASKIGRWYSRTMSKTKRMGMGLYVKYDGDPWTDSEALALSGVMVSMEEPGWYSFGFEIKDKKCDVPCIGIEMVHDGGKTTWHSAATAIYCLMGSGQLLWDTVTGVDMAL |
| B/Georgia/09/2005 | 2005 | LPSTIQTLTLFLTSGGVLLSLYVSASLSYLLYSDILLKFSQTEITAPTMPLDCANASNVQAVNRSATKGVTLLLPEPEWTYPRLSCPGSTFQKALLISPHRFGETKGNSAPLIIREPFIACGPNECKHFALTHYAAQPGGYYNGTREDRNKLRHLISVKLGKIPTVENSIFHMAAWSGSACHDGREWTYIGVDGPDSNALLKIKYGEAYTDTYHSYAKNILRTQESACNCIGGDCYLMITDGPASGISECRFLKIREGRIIKEIFPTGRVKHTEECTCGFASNKTIECACRDNSYTAKRPFVKLDVETDTAEIRLMCTETYLDTPRPNDGSITGPCESNGDKGSGGIKGGFVHQRMASKIGRWYSRTLSKTKRMGMGLYVKYDGDPWTDSEALALSGVMVSMEEPGWYSFGFEIKDKKCDVPCIGIEMVHDGGKTTWHSAATAIYCLMGSGQLLWDTVTGVDMAL |
| B/Houston/B846/2005 | 2005 | LPSTIQTLTLFLTSGGVLLSLYVSASLSYLLYSDILLKFSQTGITAPIMPLDCANASNVQAVNRSATKGVTLLLPEPEWTYPRLSCPGSTFQKALLISPHRFGETKGNSAPLIIREPFIACGPKECKHFALTHYAAQPGGYYNGTREDRNKLRHLISVKLGKIPTVENSIFHMAAWSGSACHDGREWTYIGVDGPDSNALLKIKYGEAYTDTYHSYAKNILRTQESACNCIGGDCYLMITDGPASGISECRFLKIREGRIIKEIFPTGRVKHTEECTCGFASNKTIECACRDNSYTAKRPFVKLNVETDTAEIRLMCTETYLDTPRPNDGSITGPCESNGDKGSGGIKGGFVHQRMASKIGRWYSRTMSKTKRMGMGLYVKYDGDPWTDSEALALSGVMVSMEEPGWYSFGFEIKDKKCDVPCIGIEMVHDGGKTTWHSAATAIYCLMGSGQLLWDTVTGVDMAL |
| B/Jiangxi/568/2004 | 2005 | LPSTIQTLTLFLTSGGVLLSLYVSASLSYLLYSDILLKFSTTEITAPTIPLDCANASNVQAVNRSATKGVTFLLPEPEWTYPRLSCPGSTFQKALLISPHRFGETKGNSAPLIIREPFIACGPKECKHFALTHYAAQPGGYYNGTREDRNKLRHLISVKLGKIPTVENSIFHMAAWSGSACHDGKEWTYIGVDGPDSNALLKIKYGEAYTDTYHSYANNILRTQESACNCIGGNCYLMITDGSASGISECRFLKIREGRIIKEIFPTGRVKHTEECTCGFASNKTIECACRDNSYTAKRPFVKLNVETDTAEIRLMCTETYLDTPRPDDGSITGPCESNGNKGSGGIKGGFVHQRMASKIGRWYSRTMSKTKRMGMGLYVKYDGDPWTDSDALALSGVMVSMEEPGWYSFGFEIKDKKCDVPCIGIEMVHDGGKETWHSAATAIYCLMGSGQLLWDTVTGVDMAL |
| B/Nepal/1103/2005 | 2005 | LPSTIQTLTLFLTSGGVLLSLYVSASLSYLLYSDILLKFPSTEITAPTMPLDCANASNVQAVNRSATKRVTLLLPEPEWTYPRLSCPGSTFQKALLISPHRFGETKGNSAPLIIREPFIACGPKECKHFALTHYAAQPGGYYNGTRGDRNKLRHLISVKLGKIPTVENSIFHMAAWSGSACHDGKEWTYIGVDGPDNNALLKIKYGEAYTDTYHSYANNILRTQESACNCIGGNCYLMITDGSASGVSECRFLKIREGRIIKEIFPTGRIKHTEECTCGFASNKTIECACRDNSYTAKRPFVKLNVETDTAEIRLMCTETYLDTPRPDDGSITGPCESNGDKGSGGIKGGFVHQRMASKIGRWYSRTMSKTKRMGMGLYVKYDGDPWADSDALAFSGVMVSMEEPGWYSFGFEIKDKKCDVPCIGIEMVHDGGKETWHSAATAIYCLMGSGQLLWDTVTGVNMAL |
| B/Nepal/1106/2005 | 2005 | LPSTIQTLTLFLTSGGVLLSLYVSASLSYLLYSDILLKFSPTEIIAPTMPLDCANASNVQAVNRSTTKGVTLLLPEPEWTYPRLSCPGSTFQKALLISPHRFGETKGNSAPLIIREPFIACGPKECKHFALTHYAAQPGGYYNGTRGDRNKLRHLISVKLGKIPTVENSIFHMAAWSGSACHDGKEWTYIGVDGPDNNALLKIKYGEAYTDTYHSYANNILRTQESACNCIGGNCYLMITDGSASGISECRFLKIREGRIIKEIFPTGRVKHTEECTCGFASNKTIECACRDNSYTAKRPFVKLNVETDTAEIRLMCTETYLDTPRPDDGSITGPCESNGDKGSGGIKGGFVHQRMASKIGRWYSRTMSKTKRMGMGLYVKYDGDPWADSDALALSGVMVSMEEPGWYSFGFEIKDKKCDVPCIGIEMVHDGGKETWHSAATAIYCLMGSGQLLWDTVTGVDMAL |
| B/Nepal/1114/2005 | 2005 | LPSTIQTLTLFLTSGGVLLSLYVSASLSYLLYSDILLKFPPTEITAPTMPLDCANASNVQAVNRSATKGVTLLLPEPEWTYPRLSCPGSTFQKALLISPHRFGETKGNSAPLIIREPFIACGPKECKHFALTHYAAQPGGYYNGTRGDRNKLRHLISVKLGKIPTVENSIFHMAAWSGSACHDGKEWTYIGVDGPDNNALLKIKYGEAYTDTYHSYANNILRTQESACNCIGGNCYLMITDGSASGVSECRFLKIREGRIIKEIFPTGRVKHTEECTCGFASNKTIECACRDNSYTAKRPFVKLNVETDTAEIRLMCTETYLDTPRPDDGSITGPCESNGDKGSGGIKGGFVHQRMASKIGRWYSRTMSKTKRMGMGLYVKYDGDPWADSDALAFSGVMISMEEPGWYSFGFEIKDKKCDVPCIGIEMVHDGGKETWHSAATAIYCLMGSGQLLWDTVTGVNMAL |
| B/Nepal/1120/2005 | 2005 | LPSTIQTLTLFLTSGGVLLSLYVSASLSYLLYSDILLKFPSTEITAPTMPLDCANASNVQAVNRSATKGMTLLLPEPEWTYPRLSCPGSTFQKALLISPHRFGETKGNSAPLIIREPFIACGPKECKHFALTHYAAQPGGYYNGTRGDRNKLRHLISVKLGKIPTVENSIFHMAAWSGSACHDGKEWTYIGVDGPDNNALLKIKYGEAYTDTYHSYANNILRTQESACNCIGGNCYLMITDGSASGVSECRFLKIREGRIIKEIFPTGRIKHTEECTCGFASNKTIECACRDNSYTAKRPFVKLNVETDTAEIRLMCTDTYLDTPRPDDGSITGPCESNGDKGSGGIKGGFVHQRMASKIGRWYSRTMSKTKRMGMGLYVKYDGDPWADSDALAFSGVMVSMEEPGWYSFGFEIKDKKCDVPCIGIEMVHDGGKETWHSAATAIYCLMGSGQLLWDTVTGVNMAL |
| B/Nepal/1122/2005 | 2005 | LPSTIQTLTLFLTSGGVLLSLYVSASLSYLLYSDILLKFPSTEITAPTMPLDCANASNVQAVNRSATKGMTLLLPEPEWTYPRLSCPGSTFQKALLISPHRFGETKGNSAPLIIREPFIACGPKECKHFALTHYAAQPGGYYNGTRGDRNKLRHLISVKLGKIPTVENSIFHMAAWSGSACHDGKEWTYIGVDGPDNNALLKIKYGEAYTDTYHSYANNILRTQESACNCIGGNCYLMITDGSASGVSECRFLKIREGRIIKEIFPTGRIKHTEECTCGFASNKTIECACRDNSYTAKRPFVKLNVETDTAEIRLMCTDTYLDTPRPDDGSITGPCESNGDKGSGGIKGGFVHQRMASKIGRWYSRTMSKTKRMGMGLYVKYDGDPWADSDALAFSGVMVSMEEPGWYSFGFEIKDKKCDVPCIGIEMVHDGGKETWHSAATAIYCLMGSGQLLWDTVTGVNMAL |
| B/Nepal/1131/2005 | 2005 | LPSTIQTLTLFLTSGGVLLSLYVSASLSYLLYSDILLKFPSTEITAPTMPLDCANASNVQAVNRSATKRVTLLLPEPEWTYPRLSCPGSTFQKALLISPHRFGETKGNSAPLIIREPFIACGPKECKHFALTHYAAQPGGYYNGTRGDRNKLRHLISVKLGKIPTVENSIFHMAAWSGSACHDGKEWTYIGVDGPDNNALLKIKYGEAYTDTYHSYANNILRTQESACNCIGGNCYLMITDGSASGVSECRFLKIREGRIIKEIFPTGRIKHTEECTCGFASNKTIECACRDNSYTAKRPFVKLNVETDTAEIRLMCTETYLDTPRPDDGSITGPCESNGDKGSGGIKGGFVHQRMASKIGRWYSRTMSKTKRMGMGLYVKYDGDPWADSDALAFSGVMVSMEEPGWYSFGFEIKDKKCDVPCIGIEMVHDGGKETWHSAATAIYCLMGSGQLLWDTVTGVNMAL |
| B/New York/1072/2005 | 2005 | LPSTIQTLTLFLTSGGVLLSLYVSASLSYLLYSDILLKFSQTEITAPIMPLDCANASNVQAVNRSATKGVTLLLPEPEWTYPRLSCPGSTFQKALLISPHRFGETKGNSAPLIIREPFIACGPTECKHFALTHYAAQPGGYYNGTREDRNKLRHLISVKLGKIPTVENSIFHMAAWSGSACHDGKEWTYIGVDGPDSNALLKIKYGEAYTDTYHSYAKNILRTQESACNCIGGDCYLMITDGPASGISECRFLKIREGRIIKEIFPTGRVKHTEECTCGFASNKTIECACRDNSYTAKRPFVKLNVETDTAEIRLMCTETYLDTPRPNDGSITGPCESDGDKGSGGIKGGFVHQRMASKIGRWYSRTMSKTKRMGMGLYVKYDGDPWTDSEALALSGVMVSMEEPGWYSFGFEIKDKKCDVPCIGIEMVHDGGKTTWHSAATAIYCLMGSGQLLWDTVTGVDMAL |
| B/Ohio/1/2005 | 2005 | LPSTIQTLTLFLTSGGVLLSLYVSASLSYLLYSDILLKFSPTEITAPTMPLDCANASNVQAVNRSATKGVTLLLPEPEWTYPRLSCPGSTFQKALLISPHRFGETKGNSAPLIIREPFIACGPNECKHFALTHYAAQPGGYYNGTRGDRNKLRHLISVKLGKIPTVENSIFHMAAWSGSACHDGKEWTYIGVDGPDNNALLKIKYGEAYTDTYHSYANNILRTQESACNCIGGNCYLMITDGSASGVSECRFLKIREGRIIKEIFPTGRVKHTEECTCGFASNKTIECACRDNSYTAKRPFVKLNVETDTAEIRLMCTDTYLDTPRPDDGSITGPCESNGDKGSGGIKGGFVHQRMASKIGRWYSRTMSKTERMGMGLYVKYDGDPWADSDALAFSGVMVSMKEPGWYSFGFEIKDKKCDVPCIGIEMVHDGGKETWHSAATAIYCLMGSGQLLWDTVTGVDMAL |
| B/South Carolina/NHRC0001/2005 | 2005 | LPSTIQTLTLFLTSGGVLLSLYVSASLSYLLYSDILLKFSQTEITAPIMPLDCANASNVQAVNRSAAKGVTLLLPEPEWTYPRLSCPGSTFQKALLISPHRFGETKGNSAPLIIREPFIACGPTECKHFALTHYAAQPGGYYNGTREDRNKLRHLISVKLGKIPTVENSIFHMAAWSGSACHDGKEWTYIGVDGPDSNALLKIKYGEAYTDTYHSYAKNILRTQESACNCIGGDCYLMITDGPASGISECRFLKIREGRIIKEIFPTGRVKHTEECTCGFASNKTIECACRDNSYTAKRPFVKLNVETDTAEIRLMCTETYLDTPRPNDGSITGPCESDGDKGSGGIKGGFVHQRMASKIGRWYSRTMSKTKRMGMGLYVKYDGDPWTDSEALTLSGVMVSMEEPGWYSFGFEIKDKKCDVPCIGIEMVHDGGKTTWHSAATAIYCLMGSGQLLWDTVTGVDMAL |
| B/Sydney/15/2005 | 2005 | LPSTIQTLTLFLTSGGVLLSLYVSASLSYLLYSDILLKFSPTEITAPTMPLDCANASNVHAVNRSATKGVTPLLPEPEWTYPRLSCPGSTFQKALLISPHRFGETKGNSAPLIIREPFIACGPNECKHFALTHYAAQPGGYYNGTRGDRNKLRHLISVKLGKIPTVENSIFHMAAWSGSACHDGKEWTYIGVDGPDNNALLKIKYGEAYTDTYHSYANNILRTQESACNCIGGNCYLMITDGSASGVSECRFLKIREGRIIKEIFPTGRVKHTEECTCGFASNKTIECACRDNSYTAKRPFVKLNVETDTAEIRLMCTDTYLDTPRPDDGSITGPCESNGDKGSGGIKGGFVHQRMASKIGRWYSRTMSKTERMGMGLYVKYDGDPWADSDALAFSGVMVSMKEPGWYSFGFEIKDKKCDVPCIGIEMVHDGGKETWHSAATAIYCLMGSGQLLWDTVTGVDMAL |
| B/Sydney/19/2005 | 2005 | LPSTIQTLTLFLTSGGVLLSLYVSASLSYLLYSDILLKFSPTEITAPTMPLDCANASNVHAVNRSATKGVTPLLPEPEWTYPRLSCPGSTFQKALLISPHRFGETKGNSAPLIIREPFIACGPNECKHFALTHYAAQPGGYYNGTRGDRNKLRHLISVKLGKIPTVENSIFHMAAWSGSACHDGKEWTYIGVDGPDNNALLKIKYGEAYTDTYHSYANNILRTQESACNCIGGNCYLMITDGSASGVSECRFLKIREGRIIKEIFPTGRVKHTEECTCGFASNKTIECACRDNSYTAKRPFVKLNVETDTAEIRLMCTDTYLDTPRPDDGSITGPCESNGDKGSGGIKGGFVHQRMASKIGRWYSRTMSKTERMGMGLYVKYDGDPWADSDALAFSGVMVSMKEPGWYSFGFEIKDKKCDVPCIGIEMVHDGGKETWHSAATAIYCLMGSGQLLWDTVTGVDMAL |
| B/Sydney/2/2005 | 2005 | LPSTIQTLTLFLTSGGVLLSLYVSASLSYLLYSDILLKFSQTEITAPIMPLDCANASNVQAVNRSAAKGVTLLLPEPEWTYPRLSCPGSTFQKALLISPHRFGETKGNSAPLIIREPFIACGPTECKHFALTHYAAQPGGYYNGTREDRNKLRHLISVKLGKIPTVENSIFHMAAWSGSACHDGKEWTYIGVDGPDSNALLKIKYGEAYTDTYHSYAKNILRTQESACNCIGGDCYLMITDGPASGISECRFLKIREGRIIKEIFPTGRVKHTEECTCGFASNKTIECACRDNSYTAKRPFVKLNVETDTAEIRLMCTETYLDTPRPNDGSITGPCESDGDKGSGGIKGGFVHQRMASKIGRWYSRTMSKTKRMGMGLYVKYDGDPWTDSEALALSGVMVSMEEPGWYSFGFEIKDKKCDVPCIGIEMVHDGGKTTWHSAATAIYCLMGSGQLLWDTVTGVDMAL |
| B/Taiwan/00748/2005 | 2005 | LPSTIQTLTLFLTSGGVLLSLYVSASLSYLLYSDILLKFSQTEITAPTMPLDCANASNVQAVNRSATKGVTLLLPEPEWTYPRLSCPGSTFQKALLISPHRFGETKGNSAPLIIREPFIACGPNECKHFALTHYAAQPGGYYNGTREDRNKLRHLISVKLGKIPTVENSIFHMAAWSGSACHDGREWTYIGVDGPDSNALLKIKYGEAYTDTYHSYAKNILRTQESACNCIGGDCYLMITDGPASGISECRFLKIREGRIIKEIFPTGRVKHTEECTCGFASNKTIECACRDNSYTAKRPFVKLDVETDTAEIRLMCTETYLDTPRPNDGSITGPCESNGDKGSGGIKGGFVHQRMASKIGRWYSRTLSKTKRMGMGLYVKYDGDPWTDSEALALSGVMVSMEEPGWYSFGFEIKDKKCDVPCIGIEMVHDGGKTTWHSAATAIYCLMGSGQLLWDTVTGVDMAL |
| B/Taiwan/539/2005 | 2005 | LPSTIQTLTLFLTSGGVLLSLYVSASLSYLLYSDILLKFSTTEITAPTIPLDCANASNVQAVNRSATKGVTLLLPEPEWTYPRLSCPGSTFQKALLISPHRFGETKGNSAPLIIREPFIACGPKECKHFALTHYAAQPGGYYNGTREDRNKLRHLISVKLGKIPTVENSIFHMAAWSGSACHDGKEWTYIGVDGPDSNALLKIKYGEAYTDTYHSYANNILRTQESACNCIGGNCYLMITDGSASGISECRFLKIREGRIIKEIFPTGRVKHTEECTCGFASNKTIECACRDNSYTAKRPFVKLNVETDTAEIRLMCTETYLDTPRPDDGSITGPCESNGNKGSGGIKGGFVHQRMASKIGRWYSRTMSKTKRMGMGLYVKYDGDPWTDSDALALSGVMVSMEEPGWYSFGFEIKDKKCDVPCIGIEMVHDGGKETWHSAATAIYCLMGSGQLLWDTVTGVDMAL |
| B/Taiwan/70864/2005 | 2005 | LPSTIQTLTLFLTSGGVLLSLYVSASLSYLLYSDILLKFSQTEITAPIMPLDCANASNVQAVNRSAAKGVTLLLPEPEWTYPRLSCPGSTFQKALLISPHRFGETKGNSAPLIIREPFIACGPTECKHFALTHYAAQPGGYYNGTREDRNKLRHLISVKLGKIPTVENSIFHMAAWSGSACHDGKEWTYIGVDGPDSNALLKIKYGEAYTDTYHSYAKNILRTQESACNCIGGDCYLMITDGPASGISECRFLKIREGRIIKEIFPTGRVKHTEECTCGFASNKTIECACRDNSYTAKRPFVKLNVETDTAEIRLMCTETYLDTPRPNDGSITGPCESDGDKGSGGIKGGFVHQRMASKIGRWYSRTMSKTKRMGMGLYVKYDGDPWTDSEALALSGVMVSMEEPGWYSFGFEIKDKKCDVPCIGIEMVHDGGKTTWHSAATAIYCLMGSGQLLWDTVTGVDMAL |
| B/Taiwan/91105/2005 | 2005 | LPSTIQTLTLFLTSGGVLLSLYVSASLSYLLYSDILLKFSQTEITAPIMPLDCANASNVQAVNRSAAKGVTLLLPEPEWTYPRLSCPGSTFQKALLISPHRFGETKGNSAPLIIREPFIACGPTECKHFALTHYAAQPGGYYNGTREDRNKLRHLISVKLGKIPTVENSIFHMAAWSGSACHDGKEWTYIGVDGPDSNALLKIKYGEAYTDTYHSYAKNILRTQESACNCIGGDCYLMITDGPASGISECRFLKIREGRIIKEIFPTGRVKHTEECTCGFASNKTIECACRDNSYTAKRPFVKLNVETDTAEIRLMCTETYLDTPRPNDGSITGPCESDGDKGSGGIKGGFVHQRMASKIGRWYSRTMSKTKRMGMGLYVKYDGDPWTDSEALALSGVMVSMEEPGWYSFGFEIKDKKCDVPCIGIEMVHDGGKTTWHSAATAIYCLMGSGQLLWDTVTGVDMAL |
| B/Townsville/11/2005 | 2005 | LPSTIQTLTLFLTSGGVLLSLYVSASLSYLLYSDILLKFSQTEITAPIMPLDCANASNVQAVNRSAAKGVTLLLPEPEWTYPRLSCPGSTFQKALLISPHRFGETKGNSAPLIIREPFIACGPTECKHFALTHYAAQPGGYYNGTREDRNKLRHLISVKLGKIPTVENSIFHMAAWSGSACHDGKEWTYIGVDGPDSNALLKIKYGEAYTDTYHSYAKNILRTQESACNCIGGDCYLMITDGPASGISECRFLKIREGRIIKEIFPTGRVKHTEECTCGFASNKTIECACRDNSYTAKRPFVKLNVETDTAEIRLMCTETYLDTPRPNDGSITGPCESDGDKGSGGIKGGFVHQRMASKIGRWYSRTMSKTKRMGMGLYVKYDGDPWTDSEALALSGVMVSMEEPGWYSFGFEIKDKKCDVPCIGIEMVHDGGKTTWHSAATAIYCLMGSGQLLWDTVTGVDMAL |
| B/Townsville/15/2005 | 2005 | LPSTIQTLTLFLTSGGVLLSLYVSASLSYLLYSDILLKFSQTEIIAPIMPLDCANASNVQAVNRSAAKGVTLLLPEPEWTYPRLSCPGSTFQKALLISPHRFGETKGNSAPLIIREPFIACGPTECKHFALTHYAAQPGGYYNGTREDRNKLRHLISVKLGKIPTVENSIFHMAAWSGSACHDGKEWTYIGVDGPDSNALLKIKYGEAYTDTYHSYAKNILRTQESACNCIGGDCYLMITDGPASGISECRFLKIREGRIIKEIFPTGRVKHTEECTCGFASNKTIECACRDNSYTAKRPFVKLNVETDTAEIRLMCTETYLDTPRPNDGSITGPCESDGDKGSGGIKGGFVHQRMASKIGRWYSRTMSKTKRMGMGLYVKYDGDPWTDSEALALSGVMVSMEEPGWYSFGFEIKDKKCDVPCIGIEMVHDGGKTTWHSAATAIYCLMGSGQLLWDTVTGVDMAL |
| B/Townsville/17/2005 | 2005 | LPSTIQTLTLFLTSGGVLLSLYVSASLSYLLYSDILLKFSQTEITAPIMPLDCANASNVQAVNRSAAKGVTLLLPEPEWTYPRLSCPGSTFQKALLISPHRFGETKGNSAPLIIREPFIACGPTECKHFALTHYAAQPGGYYNGTREDRNKLRHLISVKLGKIPTVENSIFHMAAWSGSACHDGKEWTYIGVDGPDSNALLKIKYGEAYTDTYHSYAKNILRTQESACNCIGGDCYLMITDGPASGISECRFLKIREGRIIKEIFPTGRVKHTEECTCGFASNKTIECACRDNSYTAKRPFVKLNVETDTAEIRLMCTETYLDTPRPNDGSITGPCESDGDKGSGGIKGGFVHQRMASKIGRWYSRTMSKTKRMGMGLYVKYDGDPWTDSEALALSGVMVSMEEPGWYSFGFEIKDKKCDVPCIGIEMVHDGGKTTWHSAATAIYCLMGSGQLLWDTVTGVDMAL |
| B/Victoria/310/2005 | 2005 | LPSTIQTLTLFLTSGGVLLSLYVSASLSYLLYSDILLKFSPTEITAPTMPLDCANASNVQAVNRSATKGVTLLLPEPEWTYPRLSCPGSTFQKALLISPHRFGETKGNSAPLIIREPFIACGPNECKHFALTHYAAQPGGYYNGTRGDRNKLRHLISVKLGKIPTVENSIFHMAAWSGSACHDGKEWTYIGVDGPDNNALLKIKYGEAYTDTYHSYANNILRTQESACNCIGGNCYLMITDGSASGVSECRFLKIREGRIIKEIFPTGRVKHTEECTCGFASNKTIECACRDNSYTAKRPFVKLNVEIDTAEIRLMCTDTYLDTPRPDDGSITGSCESNGDKGSGGIKGGFVHQRMASKIGRWYSRTMSKTERMGMGLYVKYDGDPWADSDALAFSGVMVSMKEPGWYSFGFEIKDKKCDVPCIGIEMVHDGGKETWHSAATAIYCLMGSGQLLWDTVTGVDMAL |
| B/Victoria/507/2005 | 2005 | LPSTIQTLTLFLTSGGVLLSLYVSASLSYLLYSDILLKFSQTEITAPIMPLDCANASNVQAVNRSATKGVTLLLPEPEWTYPRLSCPGSTFQKALLISPHRFGETKGNSAPLIIREPFIACGPKECKHFALTHYAAQPGGYYNGTREDRNKLRHLISVKLGKIPTVENSIFHMAAWSGSACHDGREWTYIGVDGPDSNALLKIKYGEAYTDTYHSYAKNILRTQESACNCIGGDCYLMITDGPASGISECRFLKIREGRIIKEIFPTGRVKHTEECTCGFASNKTIECACRDNNYTAKRPFVKLNVETDTAEIRLMCTETYLDTPRPNDGSITGPCESNGDKGSGGIKGGFVHQRMASKIGRWYSRTMSKTKRMGMGLYVKYDGDPWTDSEALALSGVMVSMEEPGWYSFGFEIKDKKCDVPCIGIEMVHDGGKTTWHSAATAIYCLMGSGQLLWDTVTGVDMAL |
| B/Victoria/510/2005 | 2005 | LPSTIQTLTLFLTSGGVLLSLYVSASLSYLLYSDILLKFSPTEITAPTMPLDCANASNVQAVNRSATKGVTLLLPEPEWTYPRLSCPGSTFQKALLISPHRFGETKGNSAPLIIREPFIACGPNECKHFALTHYAAQPGGYYNGTRGDRNKLRHLISVKLGKIPTVENSIFHMAAWSGSACHDGKEWTYIGVDGPDNNALLKIKYGEAYTDTYHSYANNILRTQESACNCIGGNCYLMITDGSASGVSECRFLKIREGRIIKEIFPTGRVKHTEECTCGFASNKTIECACRDNSYTAKRPFVKLNVEIDTAEIRLMCTDTYLDTPRPDDGSITGPCESNGDKGSGGIKGGFVHQRMASKIGRWYSRTMSKTERMGMGLYVKYDGDPWADSDALAFSGVMVSMKEPGWYSFGFEIKDKKCDVPCIGIEMVHDGGKETWHSAATAIYCLMGSGQLLWDTVTGVDMAL |
| B/Victoria/522/2005 | 2005 | LPSTIQTLTLFLTSGGVLLSLYVSASLSYLLYSDILLKFSPTEITAPTMPLDCANASNVQAVNRSATKGVTLLLPEPEWTYPRLSCPGSTFQKALLISPHRFGETKGNSAPLIIREPFIACGPNECKHFALTHYAAQPGGYYNGTRGDRNKLRHLISVKLGKIPTVENSIFHMAAWSGSACHDGKEWTYIGVDGPDNNALLKIKYGEAYTDTYHSYANNILRTQESACNCIGGNCYLMITDGSASGVSECRFLKIREGRIIKEIFPTGRVKHTEECTCGFASNKTIECACRDNSYTAKRPFVKLNVEIDTAEIRLMCTDTYLDTPRPDDGSITGPCESNGDKGSGGIKGGFVHQRMASKIGRWYSRTMSKTERMGMGLYVKYDGDPWADSDALAFSGVMVSMKEPGWYSFGFEIKDKKCDVPCIGIEMVHDGGKETWHSAATAIYCLMGSGQLLWDTVTGVDMAL |
| B/Victoria/530/2005 | 2005 | LPSTIQTLTLFLTSGGVLLSLYVSASLSYLLYSDILLKFSQTEITAPIMPLDCANASNVQAVNRSAAKGVTLLLPEPEWTYPRLSCPGSTFQKALLISPHRFGETKGNSAPLIIREPFIACGPTECKHFALTHYAAQPGGYYNGTREDRNKLRHLISVKLGKIPTVENSIFHMAAWSGSACHDGKEWTYIGVDGPDSNALLKIKYGEAYTDTYHSYAKNILRTQESACNCIGGDCYLMITDGPASGISECRFLKIREGRIIKEIFPTGRVKHTEECTCGFASNKTIECACRDNSYTAKRPFVKLNVETDTAEIRLMCTETYLDTPRPNDGSITGPCESDGDKGSGGIKGGFVHQRMASKIGRWYSRTMSKTKRMGMGLYVKYDGDPWTDSEALALSGVMVSMEEPGWYSFGFEIKDKKCDVPCIGIEMVHDGGKTTWHSAATAIYCLMGSGQLLWDTVTGVDMAL |
| B/Victoria/531/2005 | 2005 | LPSTIQTLTLFLTSGGVLLSLYVSASLSYLLYSDILLKFSQTEITAPIMPLDCANASNVQAVNRSAAKGVTLLLPEPEWTYPRLSCPGSTFQKALLISPHRFGETKGNSAPLIIREPFIACGPTECKHFALTHYAAQPGGYYNGTREDRNKLRHLISVKLGKIPTVENSIFHMAAWSGSACHDGKEWTYIGVDGPDSNALLKIKYGEAYTDTYHSYAKNILRTQESACNCIGGDCYLMITDGPASGISECRFLKIREGRIIKEIFPTGRVKHTEECTCGFASNKTIECACRDNSYTAKRPFVKLNVETDTAEIRLMCTETYLDTPRPNDGSITGPCESDGDKGSGGIKGGFVHQRMASKIGRWYSRTMSKTKRMGMGLYVKYDGDPWTDSEALALSGVMVSMEEPGWYSFGFEIKDKKCDVPCIGIEMVHDGGKTTWHSAATAIYCLMGSGQLLWDTVTGVDMAL |
| B/Waikato/146/2005 | 2005 | LPSTIQTLTLFLTSGGVLLSLYVSASLSYLLYSDILLKFSQTEITAPIMPLDCANASNVQAVNRSAAKGVTLLLPEPEWTYPRLSCPGSTFQKALLISPHRFGETKGNSAPLIIREPFIACGPTECKHFALTHYAAQPGGYYNGTREDRNKLRHLISVKLGKIPTVENSIFHMAAWSGSACHDGKEWTYIGVDGPDSNALLKIKYGEAYTDTYHSYAKNILRTQESACNCIGGDCYLMITDGPASGISECRFLKIREGRIIKEIFPTGRVKHTEECTCGFASNKTIECACRDNSYTAKRPFVKLNVETDTAEIRLMCTETYLDTPRPNDGSITGPCESDGDKGSGGIKGGFVHQRMASKIGRWYSRTMSKTKRMGMGLYVKYDGDPWTDSEALALSGVMVSMEEPGWYSFGFEIKDKKCDVPCIGIEMVHDGGKTTWHSAATAIYCLMGSGQLLWDTVTGVDMAL |
| B/Waikato/86/2005 | 2005 | LPSTIQTLTLFLTSGGVLLSLYVSASLSYLLYSDILLKFSQTEITAPIMPLDCANASNVQAVNRSAAKGVTLLLPEPEWTYPRLSCPGSTFQKALLISPHRFGETKGNSAPLIIREPFIACGPTECKHFALTHYAAQPGGYYNGTREDRNKLRHLISVKLGKIPTVENSIFHMAAWSGSACHDGKEWTYIGVDGPDSNALLKIKYGEAYTDTYHSYAKNILRTQESACNCIGGDCYLMITDGPASGISECRFLKIREGRIIKEIFPTGRVKHTEECTCGFASNKTIECACRDNSYTAKRPFVKLNVETDTAEIRLMCTETYLDTPRPNDGSITGPCESDGDKGSGGIKGGFVHQRMASKIGRWYSRTMSKTKRMGMGLYVKYDGDPWTDSEALALSGVMVSMEEPGWYSFGFEIKDKKCDVPCIGIEMVHDGGKTTWHSAATAIYCLMGSGQLLWDTVTGVDMAL |
| B/Wellington/16/2005 | 2005 | LPSTIQTLTLFLTSGGVLLSLYVSASLSYLLYSDILLKFSQTEITAPIMPLDCANASNVQAVNRSATKGVTLLLPEPEWTYPRLSCPGSTFQKALLISPHRFGETKGNSAPLIIREPFIACGPTECKHFALTHYAAQPGGYYNRTREDRNKLRHLISVKLGKIPTVENSIFHMAAWSGSACHDGKEWTYIGVDGPDSNALLKIKYGEAYTDTYHSYAKNILRTQESACNCIGGDCYLMITDGPASGISECRFLKIREGRIIKEIFPTGRVKHTEECTCGFASNKTIECACRDNSYTAKRPFVKLNVETDTAEIRLMCTETYLDTPRPNDGSITGPCESDGDKGSGGIKGGFVHQRMASKIGRWYSRTMSKTKRMGMGLYVKYDGDPWTDSEALALSGVMVSMEEPGWYSFGFEIKDKKCDVPCIGIEMVHDGGKTTWHSAATAIYCLMGSGQLLWDTVTGVDMAL |
| B/Wellington/87/2005 | 2005 | LPSTIQTLTLFLTSGGVLLSLYVSASLSYLLYSDILLKFPPTEITAPTMPLDCANASNVQAVNRSATKGVTLLLPEPEWTYPRLSCPGSTFQKALLISPHRFGETKGNSAPLIIREPFIACGPKECKHFALTHYAAQPGGYYNGTRGDRNKLRHLISVRLGKIPTVENSIFHMAAWSGSACHDGKEWTYIGVDGPDNNALLKIKYGEAYTDTYHSYANNILRTQESACNCIGGNCYLMITDGSASGVSECRFLKIREGRIIKEIFPTGRVKHTEECTCGFASNKTIECACRDNSYTAKRPFVKLNVETDTAEIRLMCTETYLDTPRPDDGSITGPCESNGDKGSGGIKGGFVHQRMASKIGRWYSRTMSKTKRMGMGLYVKYDGDPWADSDALAFSGVMVSMEEPGWYSFGFEIKDKKCDVPCIGIEMVHDGGKETWHSAATAIYCLMGSGQLLWDTVTGVNMAL |
| B/Brisbane/13/2006 | 2006 | LPSTIQTLTLFLTSGGVLLSLYVSASLSYLLYSDILLKFSPTEITAPTMPLDCANASNVHAVNRSATKGVTLLLPEPEWTYPRLSCPGSTFQKALLISPHRFGETKGNSAPLIIREPFIACGPNECKHFALTHYAAQPGGYYNRTRGDRNKLRHLISVKLGKIPTVENSIFHMAAWSGSACHDGKEWTYIGVDGPDNNALLKIKYGEAYTDTYHSYANNILRTQESACNCIGGNCYLMITDGSASGVSECRFLKIREGRIIKEIFPTGRVKHTEECTCGFASNKTIECACRDNSYTAKRPFVKLNVETDTAEIRLMCTDTYLDTPRPDDGSITGPCESNGDKGSGGIKGGFVHQRMASKIGRWYSRTMSKTERMGMGLYVKYDGDPWADSDALAFSGVMVSMKEPGWYSFGFEIKDKKCDVPCIGIEMVHDGGKETWHSAATAIYCLMGSGQLLWDTVTGVDMAL |
| B/Brisbane/16/2006 | 2006 | LPSTIQTLTLFLTSGGVLLSLYVSASLSYLLYSDILLKFSPTEITAPTMPLDCANASNVQAVNRSATKGVTLLLPEPEWTYPRLSCPGSTFQKALLISPHRFGETKGNSAPLIIREPFIACGPNECKHFALTHYAAQPGGYYNRTRGDRNKLRHLISVKLGKIPTVENSIFHMAAWSGSACHDGKEWIYIGVDGPDNNALLKIKYGEAYTDTYHSYANKILRTQESACNCIGGNCYLMITDGSASGVSECRFLKIREGRIIKEIFPTGRVKHTEECTCGFASNKTIECACRDNSYTAKRPFVKLNVETDTAEIRLMCTDTYLDTPRPDDGSITGPCESNGDKGSGGIKGGFVHQRMASKIGRWYSRTMSKTERMGMGLYVKYDGDPWADSDALAFSGVMVSMKEPGWYSFGFEIKDKKCDVPCIGIEMVHDGGKETWHSAATAIYCLMGSGQLLWDTVTGVDMAL |
| B/Brisbane/2/2006 | 2006 | LPSTIQTLTLFLTSGGVLLSLYVSASLSYLLYSDILLKFSPTEITAPTMPLDCANASNVQAVNRSATKGVTLLLPEPEWTYPRLSCPGSTFQKALLISPHRFGETKGNSAPLIIREPFIACGPNECKHFALTHYAAQPGGYYNGTRGDRNKLRHLISVKLGKIPTVENSIFHMAAWSGSACHDGKEWTYIGVDGPDNNALLKIKYGEAYTDTYHSYANKILRTQESACNCIGGNCYLMITDGSASGVSECRFLKIREGRIIKEIFPTGRVKHTEECTCGFASNKTIECACRDNSYTAKRPFVKLNVETDTAEIRLMCTDTYLDTPRPDDGSITGPCESNGDKGSGGIKGGFVHQRMASKIGRWYSRTISKTERMGMGLYVKYDGDPWADSDALAFSGVMVSMKEPGWYSFGFEIKDKKCDVPCIGIEMVHDGGKETWHSAATAIYCLMGSGQLLWDTVTGVDMAL |
| B/Brisbane/23/2006 | 2006 | LPSTIQTLTLFLTSGGVLLSLYVSASLSYLLYSDILLKFSPTEITAPTMPLDCANASNVQAVNRSATKGVTLLLPEPEWTYPRLSCPGSTFQKALLISPHRFGETKGNSAPLIIREPFIACGPNECKHFALTHYAAQPGGYYNGTRGDRNKLRHLISVKLGKIPTVENSIFHVAAWSGSACHDGKEWTYIGVDGPDNNALLKIKYGEAYTDTYHSYANKILRTQESACNCIGGNCYLMITDGSASGVSECRFLKIREGRIIKEIFPTGRVKHTEECTCGFASNKTIECACRDNSYTAKRPFVKLNVETDTAEIRLMCTDTYLDTPRPDDGSITGPCESNGDKGSGGIKGGFVHQRMASKIGRWYSRTMSKTERMGMGLYVKYDGDPWADSDALAFSGVMVSMKEPGWYSFGFEIKDKKCDVPCIGIEMVHDGGKETWHSAATAIYCLMGSGQLLWDTVTGVDMAL |
| B/Brisbane/31/2006 | 2006 | LPSTIQTLTLFLTSGGVLLSLYVSASLSYLLYSDILLKFSPTEITAPTMPLDCANASNVHAVNRSATKGVTLLLPEPEWTYPRLSCPGSTFQKALLISPHRFGETKGNSAPLIIREPFIACGPNECKHFALTYYAAQPGGYYNGTRGDRNKLRHLISVKLGKIPTVENSIFHMAAWSGSACHDGKEWTYIGVDGPDNNALLKIKYGEAYTDTYHSYANNILRTQESACNCIGGNCYLMITDGSASGVSECRFLKIREGRIIKEIFPTGRVKHTEECTCGFASNKTIECACRDNSYTAKRPFVKLNVETDTAEIRLMCTDTYLDTPRPDDGSITGPCESNGDKGSGGIKGGFVHQRMASKIGRWYSRTMSKTERMGMGLYVKYDGDPWADSDALAFSGVMVSMKEPGWYSFGFEIKDKKCDVPCIGIEMVHDGGKETWHSAATAIYCLMGSGQLLWDTVTGVDMAL |
| B/Brisbane/43/2006 | 2006 | LPSTIQTLTLFLTSGGVLLSLYVSASLSYLLYSDILLKFTPTEITAPTMPLDCANASNVQAVNRSATKGVTPLLPEPEWTYPRLSCPGSTFQKALLISPHRFGETKGNSAPLIIREPFIACGPNECKHFALTHYAAQPGGYYNGTRGDRNKLRHLISVKLGKIPTVENSIFHMAAWSGSACHDGKEWTYIGVDGPDNNALLKIKYGEAYTDTYHSYANKILRTQESACNCIGGNCYLMITDGSASGVSECRFLKIREGRIIKEIFPTGRVKHTEECTCGFASNKTIECACRDNSYTAKRPFVKLNVETDTAEIRLMCTDTYLDTPRPDDGSITGPCESNGDKGSGGIKGGFVHQRMASKIGRWYSRTMSKTERMGMGLYVKYDGDPWADSDALAFSGVMVSMKEPGWYSFGFEIKDKKCDVPCIGIEMVHDGGKETWHSAATAIYCLMGSGQLLWDTVTGVDMAL |
| B/Brisbane/5/2006 | 2006 | LPSTIQTLTLFLTSGGVLLSLYVSASLSYLLYSDILLKFSQTEITAPIMPLDCANASNVQAVNRSAAKGVTLLLPEPEWTYPRLSCPGSTFQKALLISPHRFGETKGNSAPLIIREPFIACGPTECKHFALTHYAAQPGGYYNGTREDRNKLRHLISVKLGKIPTVENSIFHMAAWSGSACHDGKEWTYVGVDGPDSNALLKIKYGEAYTDTYHSYAKNILRTQESACNCIGGDCYLMITDGPASGISECRFLKIREGRIIKEIFPTGRVKHTEECTCGFASNKTIECACRDNSYTAKRPFVKLNVETDTAEIRLMCTETYLDTPRPNDGSITGPCESDGDKGSGGIKGGFVHQRMASKIGRWYSRTMSKTKRMGMGLYVKYDGDPWTDSEALALSGVMVSMEEPGWYSFGFEIKDKKCDVPCIGIEMVHDGGKTTWHSAATAIYCLMGSGQLLWDTVTGVDMAL |
| B/Brisbane/67/2006 | 2006 | LPSTIQTLTLFLTSGGVLLSLYVSASLSYLLYSDILLKFSPTEITAPTMPLDCANASNVQAVNRSATKGVTLLLPEPEWTYPRLSCPGSTFQKALLISPHRFGETKGNSAPLIIREPFIACGPNECKHFALTHYAAQPGGYYNGTRGDRNKLRHLISVKLGKIPTVENSIFHMAAWSGSACHDGKEWTYIGVDGPDNNALLKIKYGEAYTDTYHSYANKILRTQESACNCIGGNCYLMITDGSASGVSECRFLKIREGRIIKEIFPTGRVKHTEECTCGFASNKTIECACRDNSYTAKRPFVKLNVETDTAEIRLMCTDTYLDTPRPDDGSITGPCESNGDKGSGGIKGGFVHQRMASKIGRWYSRTMSKTERMGMGLYVKYDGDPWADSDALAFSGVMVSMKEPGWYSFGFEIKDKKCDVPCIGIEMVHDGGKETWHSAATAIYCLMGSGQLLWDTVTGVDMAL |
| B/Brisbane/74/2006 | 2006 | LPSTIQTLTLFLTSGGVLLSLYVSASLSYLLYSDILLKFSPTEITAPTMPLDCANASNVHAVNRSATKGVTLLLPEPEWTYPRLSCPGSTFQKALLISPHRFGETKGNSAPLIIREPFIACGPNECKHFALTHYAAQPGGYYNGTRGDRNKLRHLISVKLGKIPTVENSIFHMAAWSGSACHDGKEWTYIGVDGPDNNALLKIKYGEAYTDTYHSYANNILRTQESACNCIGGNCYLMITDGSASGVSECRFLKIREGRIIKEIFPTGRVKHTEECTCGFASNKTIECACRDNSYTAKRPFVKLNVETDTAEIRLMCTDTYLDTPRPDDGSITGPCESNGDKGSGGIKGGFVHQRMASKIGRWYSRTMSKTERMGMGLYVKYDGDPWAESDALAFSGVMVSMKEPGWYSFGFEIKDKKCDVPCIGIEMVHDGGKETWHSAATAIYCLMGSGQLLWDTVTGVDMAL |
| B/Brisbane/77/2006 | 2006 | LPSTIQTLTLFLTSGGVLLSLYVSASLSYLLYSNILLKFSPTEITAPTMPLDCANASNVHAVNRSATKGVTLLLPEPEWTYPRLSCPGSTFQKALLISPHRFGETKGNSAPLIIREPFIACGPNECKHFALTHYAAQPGGYYNGTRGDRNKLRHLISVKLGKIPTVENSIFHMAAWSGSACHDGKEWTYIGVDGPDNNALLKIKYGEAYTDTYHSYANNILRTQESACNCIGGNCYLMITDGSASGVSECRFLKIREGRIIKEIFPTGRVKHTEECTCGFASNKTIECACRDNSYTAKRPFVKLNVETDTAEIRLMCTDTYLDTPRPDDGSITGPCESNGDKGSGGIKGGFVHQRMASKIGRWYSRTMSKTERMGMGLYVKYDGDPWADSDALAFSGVMVSMKEPGWYSFGFEIKDKKCDVPCIGIEMVHDGGKETWHSAATAIYCLMGSGQLLWDTVTGVDMAL |
| B/Brisbane/84/2006 | 2006 | LPSTIQTLTLFLTSGGVLLSLYVSASLSYLLYSDILLKFSPTEITAPTMPLDCANASNVQAVNRSATKGVTLLLPEPEWTYPRLSCPGSTFQKALLISPHRFGETKGNSAPLIIREPFIACGPNECKHFALTHYAAQPGGYYNGTRGDRNKLRHLISVKLGKIPTVENSIFHMAAWSGSACHDGKEWTYIGVDGPDNNALLKIKYGEAYTDTYHSYANKILRTQESACNCIGGNCYLMITDGSASGVSECRFLKIREGRIIKEIFPTGRVKHTEECTCGFASNKTIECACRDNSYTAKRPFVKLNVETDTAEIRLMCTDTYLDTPRPDDGSITGPCESNGDKGSGGIKGGFVHQRMASKIGRWYSRTMSKTERMGMGLYVKYDGDPWADSDALAFSGVMVSMKEPGWYSFGFEIKDKKCDVPCIGIEMVHDGGKETWHSAATAIYCLMGSGQLLWDTVTGVDMAL |
| B/Brisbane/9/2006 | 2006 | LPSTIQTLTLFLTSGGVLLSLYVSASLSYLLYSDILLKFSPTEITAPTMPLDCANASNVQAVNRSATKGVTLLLPEPEWTYPRLSCPGSTFQKALLISPHRFGETKGNSAPLIIREPFIACGPNECKHFALTHYAAQPGGYYNGTRGDRNKLRHLISVKLGKIPTVENSIFHMAAWSGSACHDGKEWTYIGVDGPDNNALLKIKYGEAYTDTYHSYANKILRTQESACNCIGGNCYLMITDGSASGVSECRFLKIREGRIIKEIFPTGRVKHTEECTCGFASNKTIECACRDNSYTAKRPFVKLNVETDTAEIRLMCTDTYLDTPRPDDGSITGPCESNGDKGSGGIKGGFVHQRMASKIGRWYSRTMSKTERMGMGLYVKYDGDPWADSDALAFSGVMVSMKEPGWYSFGFEIKDKKCDVPCIGIEMVHDGGKETWHSAATAIYCLMGSGQLLWDTVTGVDMAL |
| B/Brisbane/95/2006 | 2006 | LPSTIQTLTLFLTSGGVLLSLYVSASLSYLLYSDILLKFSPTEITAPTMSLDCANASNVQAVNRSATKGVTLLLPEPEWTYPRLSCPGSTFQKALLISPHRFGETKGNSAPLIIREPFIACGPNECKHFALTHYAAQPGGYYNGTRGDRNKLRHLISVKLGKIPTVENSIFHMAAWSGSACHDGKEWTYIGVDGPDNNALLKIKYGEAYTDTYHSYANKILRTQESACNCIGGNCYLMITDGSASGVSECRFLKIREGRIIKEIFPTGRVKHTEECTCGFASNKTIECACRDNSYTAKRPFVKLNVETDTAEIRLMCTDTYLDTPRPDDGSITGPCESNGDRGSGGIKGGFVHQRMASKIGRWYSRTMSKTERMGMGLYVKYDGDPWADSDALAFSGVMVSMKEPGWYSFGFEIKDKKCDVPCIGIEMVHDGGKETWHSAATAIYCLMGSGQLLWDTVTGVDMAL |
| B/California/NHRC0001/2006 | 2006 | LPSTIQTLTLFLTSGGVLLSLYVSASLSYLLYSDILLKFSPTEITAPTMPLDCANASNVHAVNRSATKGVTLLLPEPEWTYPRLSCPGSTFQKALLISPHRFGETKGNSAPLIIREPFIACGPNECKHFALTHYAAQPGGYYNGTRGDRNKLRHLISVKLGKIPTVENSIFHMAAWSGSACHDGKEWTYIGVDGPDNNALLKIKYGEAYTDTYHSYANNILRTQESACNCIGGNCYLMITDGSASGVSECRFLKIREGRIIKEIFPTGRVKHTEECTCGFASNKTIECACRDNSYTAKRPFVKLNVETDTAEIRLMCTDTYLDTPRPDDGSITGPCESNGDKGSGGIKGGFVHQRMASKIGRWYSRTMSKTERMGMGLYVKYDGDPWADSDALAFSGVMVSVKEPGWYSFGFEIKDKKCDVPCIGIEMVHDGGKETWHSAATAIYCLMGSGQLLWDTVTGVDMAL |
| B/California/NHRC0005/2006 | 2006 | LPSTIQTLTLFLTSGGVLLSLYVSASLSYLLYSDILLKFSPTEITAPTMPLDCANASNVQAVNRSATKGVTLLLPEPEWTYPRLSCPGSTFQKALLISPHRFGETKGNSAPLIIREPFIACGPNECKHFALTHYAAQPGGYYNGTRGDRNKLRHLISVKLGKIPTVENSIFHMAAWSGSACHDGKEWTYIGVDGPDNNALLKIKYGEAYTDTYHSYANNILRTQESACNCIGGNCYLMITDGSASGVSECRFLKIREGRIIKEIFPTGRVKHTEECTCGFASNKTIECACRDNSYTAKRPFVKLNVETDTAEIRLMCTDTYLDTPRPDDGSITGPCESNGDKGSGGIKGGFVHQRMASKVGRWYSRTMSKTERMGMGLYVKYDGDPWADSDALAFSGVMVSMKEPGWYSFGFEIKDKKCDVPCIGIEMVHDGGKETWHSAATAIYCLMGSGQLLWDTVTGVDMAL |
| B/California/NHRC0006/2006 | 2006 | LPSTIQTLTLFLTSGGVLLSLYVSASLSYLLYSDILLKFSPTEITAPTMPLDCANASNVQAVNRSATKGVTLLLPEPEWTYPRLSCPGSTFQKALLISPHRFGETKGNSAPLIIREPFIACGPNECKHFALTHYAAQPGGYYNGTRGDRNRLRHLISVKLGKIPTVENSIFHMAAWSGSACHDGKEWTYIGVDGPDNNALLKIKYGEAYTDTYHSYANNILRTQESACNCIGGNCYLMITDGSASGVSECRFLKIREGRIIKEIFPTGRVKHTEECTCGFASNKTIECACRDNSYTAKRPFVKLNVETDTAEIRLMCTDTYLDTPRPDDGSITGPCESNGDKGSGGIKGGFVHQRMASKVGRWYSRTMSKTERMGMGLYVKYDGDPWADSDALAFSGVMVSMKEPGWYSFGFEIKDKKCDVPCIGIEMVHDGGKETWHSAATAIYCLMGSGQLLWDTVTGVDMAL |
| B/Christchurch/4/2006 | 2006 | LPSTIQTLTLFLTSGGVLLSLYVSASLSYLLYSDILLKFSPTEITAPTMPLDCANASNVQAVNRSATKGVTLLLPEPEWTYPRLSCPGSTFQKALLISPHRFGETKGNSAPLIIREPFIACGPNECKHFALTHYAAQPGGYYNGTRGDRNKLRHLISVKLGKIPTVENSIFHMAAWSGSACHDGKEWTYIGVDGPDNNALLKIKYGEAYTDTYHSYANKILRTQESACNCIGGNCYLMITDGSASGVSECRFLKIREGRIIKEIFPTGRVKHTEECTCGFASNKTIECACRDNSYTAKRPFVKLNVETDTAEIRLMCTDTYLDTPRPDDGSITGPCESNGDKGSGGIKGGFVHQRMASKIGRWYSRTMSKTERMGMGLYVKYDGDPWADSDALAFSGVMVSMKEPGWYSFGFEIKDKKCDVPCIGIEMVHDGGKETWHSAATAIYCLMGSGQLLWDTVTGVDMAL |
| B/Indonesia/2886/2006 | 2006 | LPSTIQMLTLFLTSGGVLLSLYVSASLSYLLYSDILLKFPLTEITAPTMPLDCANASNVQAVNRSATKGVTLLLPEPEWTYPRLSCPGSTFQKALLISPHRFGETKGNSAPLIIREPFIACGPKECKHFALTHYAAQPGGYYNGTRGDRNKMRHLISVKLGKIPTVENSIFHMAAWSGSACHDGKEWTYIGVDGPDNNALLKIKYGEAYTDTYHSYANNILRTQESACNCIGGNCYLMITDGSASGVSECRFLKIREGRIIKEIFPTGRVKHTEECTCGFASNKTIECACRDNSYTAKRPFVKLNVETDTAEIRLMCTETYLDTPRPEDGSITGPCESNGDKGSGGIKGGFVHQRMASKIGRWYSRTMSKTKRMGMGLYVKYDGDPWADSDALAFSGVMVSMEEPGWYSFGFEIKDKKCDVPCIGIEMVHDGGKETWHSAATAIYCLMGSGQLLWDTVTGVNMAL |
| B/New York/1088/2006 | 2006 | LPSTIQTLTLFLTSGGVLLSLYVSASLSYLLYSDILLKFSPTEITAPTMPLDCANASNVQAVNRSATKGVTLLLPEPEWTYPRLSCPGSTFQKALLISPHRFGETKGNSAPLIIREPFIACGPNECKHFALTHYAAQPGGYYNGTRGDRNKLRHLISVKLGKIPTVENSIFHMAAWSGSACHDGKEWTYIGVDGPDNNALLKIKYGEAYTDTYHSYANKILRTQESACNCIGGNCYLMITDGSASGVSECRFLKIREGRIIKEIFPTGRVKHTEECTCGFASNKTIECACRDNSYTAKRPFVKLNVETDTAEIRLMCTDTYLDTPRPDDGSITGPCESNGDKGSGGIKGGFVHQRMASKIGRWYSRTMSKTERMGMGLYVKYDGDPWADSDALAFSGVMVSMKEPGWYSFGFEIKDKKCDVPCIGVEMVHDGGKETWHSAATAIYCLMGSGQLLWDTVTGVDMAL |
| B/New York/1091/2006 | 2006 | LPSTIQTLTLFLTSGGVLLSLYVSASLSYLLYSDILLKFSPTEITAPTMPLDCANASNVHAVNRSATKGVTLLLPEPEWIYPRLSCPGSTFQKALLISPHRFGETKGNSAPLIIREPFIACGPNECKHFALTHYAAQPGGYYNGTRGDRNKLRHLISVKLGKIPTVENSIFHMAAWSGSACHDGKEWTYIGVDGPDNNALLKIKYGEAYTDTYHSYANNILRTQESACNCIGGNCYLMITDGSASGVSECRFLKIREGRIIKEIFPTGRVKHTEECTCGFASNKTIECACRDNSYTAKRPFVKLNVETDTAEIRLMCTDTYLDTPRPDDGSITGPCESNGDKGSGGIKGGFVHQRMASKIGRWYSRTMSKTERMGMGLYVKYNGDPWADSDALAFSGVMVSMKEPGWYSFGFEIKDKKCDVPCIGIEMVHDGGKETWHSAATAIYCLMGSGQLLWDTVTGVDMAL |
| B/New York/1092/2006 | 2006 | LPSTIQTLTLFLTSGGVLLSLYVSASLSYLLYSDILLKFSPTEITAPTMPLDCANASNVQAVNRSATKGVTLLLPEPEWTYPRLSCPGSTFQKALLISPHRFGETKGNSAPLIIREPFIACGPNECKHFALTHYAAQPGGYYNGTRGDRNKLRHLISVKLGKIPTVENSIFHMAAWSGSACHDGKEWTYIGVDGPDNNALLKIKYGEAYTDTYHSYANKILRTQESACNCIGGNCYLMITDGSASGVSECRFLKIREGRIIKEIFPTGRVKHTEECTCGFASNKTIECACRDNSYTAKRPFVKLNVETDTAEIRLMCTDTYLDTPRPDDGSITGPCESNGDKGSGGIKGGFVHQRMASKIGRWYSRTMSKTERMGMGLYVKYDGDPWADSDALAFSGVMVSMKEPGWYSFGFEIKDKKCDVPCIGIEMVHDGGKETWHSAATAIYCLMGSGQLLWDTVTGVDMAL |
| B/New York/1093/2006 | 2006 | LPSTTQTLTLFLTSGGVLLSLYVSASLSYLLYSDILLKFSPTEITAPTMPLDCANASNVQAVNRSATKGVTLLLPEPEWTYPRLSCPGSTFQKALLISPHRFGETKGNSAPLIIREPFIACGPNECKHFALTHYAAQPGGYYNGTRGDRNKLRHLISVKLGKIPTVENSIFHMAAWSGSACHDGKEWTYIGVDGPDNNALLKIKYGEAYTDTYHSYANKILRTQESACNCIGGNCYLMITDGSASGVSECRFLKIREGRIIKEIFPTGRVKHTEECTCGFASNKTIECACRDNSYTAKRPFVKLNVETDTAEIRLMCTDTYLDTPRPDDGSITGPCESNGDKGSGGIKGGFVHQRMASKIGRWYSRTMSKTERMGMGLYVKYDGDPWADSDALAFSGVMVSMKEPGWYSFGFEIKDKKCDVPCIGIEMVHDGGKETWHSAATAIYCLMGSGQLLWDTVTGVDMAL |
| B/New York/1095/2006 | 2006 | LPSTIQTLTLFLTSGGVLLSLYVSASLSYLLYSDILLKFSPTEITAPTMPLDCANASNVQAVNRSATKGVTLLLPEPEWTYPRLSCPGSTFQKALLISPHRFGETKGNSAPLIIREPFIACGPNECKHFALTHYAAQPGGYYNGTRGDRNKLRHLISVKLGKIPTVENSIFHMAAWSGSACHDGKEWTYIGVDGPDNNALLKIKYGEAYTDTYHSYANKILRTQESACNCIGGNCYLMITDGSASGVSECRFLKIREGRIIKEIFPTGRVKHTEECTCGFASNKTIECACRDNSYTAKRPFVKLNVETDTAEIRLMCTDTYLDTPRPDDGSITGPCESNGDKGSGGIKGGFVHQRMASKIGRWYSRTMSKTERMGMGLYVKYDGDPWADSDALAFSGVMVSMKEPGWYSFGFEIKDKKCDVPCIGIEMVHDGGKETWHSAATAIYCLMGSGQLLWDTVTGVDMAL |
| B/New York/1099/2006 | 2006 | LPSTIQTLTLFLTSGGVLLSLYVSASLSYLLYSDILLKFSPTEITAPTMPLDCANASNVQAVNRSATKGVTLLLPEPEWTYPRLSCPGSTFQKALLISPHRFGETKGNSAPLIIREPFIACGPNECKHFALTHYAAQPGGYYNGTRGDRNKLRHLISVKLGKIPTVENSIFHMAAWSGSACHDGKEWTYIGVDGPDNNALLKIKYGEAYTDTYHSYANKILRTQESACNCIGGNCYLMITDGSASGVSECRFLKIREGRIIKEIFPTGRVKHTEECTCGFASNKTIECACRDNSYTAKRPFVKLNVETDTAEIRLMCTDTYLDTPRPDDGSITGPCESNGDKGSGGIKGGFVHQRMASKIGRWYSRTMSKTERMGMGLYVKYDGDPWADSDALAFSGVMVSMKEPGWYSFGFEIKDKKCDVPCIGIEMVHDGGKETWHSAATAIYCLMGSGQLLWDTVTGVDMAL |
| B/South Carolina/NHRC0001/2006 | 2006 | LPSTIQTLTLFLTSGGVLLSLYVSASLSYLLYSDILLKFSPTEITAPTMPLDCANASNVHAVNRSATKGVTLLLPEPEWTYPRLSCPGSTFQKALLISPHRFGETKGNSAPLIIREPFIACGPNECKHFALTHYAAQPGGYYNGTRGDRNKLRHLISVKLGKIPTVENSIFHMAAWSGSACHDGKEWTYIGVDGPDNNALLKIKYGEAYTDTYHSYANNILRTQESACNCIGGNCYLMITDGSASGVSECRFLKIREGRIIKEIFPTGRVKHTEECTCGFASNKTIECACRDNSYTAKRPFVKLNVETDTAEIRLMCTDTYLDTPRPDDGSITGPCESNGDKGSGGIKGGFVHQRMASKIGRWYSRTMSKTERMGMGLYVKYDGDPWADSDALAFSGVMVSMKEPGWYSFGFEIKDKKCDVPCIGIEMVHDGGKETWHSAATAIYCLMGSGQLLWDTVTGVDMAL |
| B/St. Petersburg/14/2006 | 2006 | LPSTIQTLTLFLTSGGVLLSLYVSASLSYLLYSDILLKFSPTEITAPTMPLDCANASNVQAVNRSATKGVTLLLPEPEWTYPRLSCPGSTFQKALLISPHRFGETKGNSAPLIIREPFIACGPNECKHFALTHYAAQPGGYYNGTRGDRNKLRHLISVKLGKIPTVENSIFHMAAWSGSACHDGKEWTYIGVDGPDNNALLKIKYGEAYTDTYHSYANKILRTQESACNCIGGNCYLMITDGSASGVSECRFLKIREGRIIKEIFPTGRVKHTEECTCGFASNKTIECACRDNSYTAKRPFVKLNVETDTAEIRLMCTDTYLDTPRPDDGSITGPCESNGDKGSGGIKGGFVHQRMASKIGRWYSRTMSKTERMGMGLYVKYDGDPWADSDALAFSGVMVSMKEPGWYSFGFEIKDKKCDVPCIGIEMVHDGGKETWHSAATAIYCLMGSGQLLWDTVTGVDMAL |
| B/Sydney/10/2006 | 2006 | LPSTIQTLTLFLTSGGVLLSLYVSASLSYLLYSDILLKFPSTEITAPTMPLDCANASNVQAVNRSATKGVTLLLPEPEWTYPRLSCPGSTFQKALLISPHRFGETKGNSAPLIIREPFIACGPKECKHFALTHYAAQPGGYYNGTRGDRNKLRHLISVKLGKIPTVENSIFHMAAWSGSACHDGKEWTYIGVDGPDNNALLKIKYGEAYTDTYHSYANNILRTQESACNCIGGNCYLMITDGSASGVSECRFLKIREGRIIKEIFPTGRIKHTEECTCGFASNKTIECACRDNSYTAKRPFVKLNVETDTAEIRLMCTETYLDTPRPDDGSITGPCESNGDKGSGGIKGGFVHQRMASKIGRWYSRTMSKTERMGMGLYVKYDGDPWADSDALAFSGVMVSMEEPGWYSFGFEIKDKKCDVPCIGIEMVHDGGKETWHSAATAIYCLMGSGQLLWDTVTGVNMAL |
| B/Sydney/11/2006 | 2006 | LPSTIQTLTLFLTSGGVLLSLYVSASLSYLLYSDILLKFPSTEITAPTMPLDCANASNVQAVNRSATKGVTLLLPEPEWTYPRLSCPGSTFQKALLISPHRFGETKGNSAPLIIREPFIACGPKECKHFALTHYAAQPGGYYNGTRGDRNKLRHLISVKLGKIPTVENSIFHMAAWSGSACHDGKEWTYIGVDGPDNNALLKIKYGEAYTDTYHSYANNILRTQESACNCIGGNCYLMITDGSASGVSECRFLKIREGRIIKEIFPTGRIKHTEECTCGFASNKTIECACRDNSYTAKRPFVKLNVETDTAEIRLMCTETYLDTPRPDDGSITGPCESNGDKGSGGIKGGFVHQRMASKIGRWYSRTMSKTERMGMGLYVKYDGDPWADSDALAFSGVMVSMEEPGWYSFGFEIKDKKCDVPCIGIEMVHDGGKETWHSAATTIYCLMGSGQLLWDTVTGVNMAL |
| B/Sydney/204/2006 | 2006 | LPSTIQTLTLFLTSGGVLLSLYVSASLSYLLYSDILLKFSQTEITAPIMPLDCANASNVQAVNRSAAKGVTLLLPEPEWTYPRLSCPGSTFQKALLISPHRFGETKGNSAPLIIREPFIACGPTECKHFALTHYAAQPGGYYNGTREDRNKLRHLISVKLGKIPTVENSIFHMAAWSGSACHDGKEWTYIGVDGPDSNALLKIKYGEAYTDTYHSYAKNILRTQESACNCIGGDCYLMITDGPASGISECRFLKIREGRIIKEIFPTGRVKHTEECTCGFASNKTIECACRDNSYTAKRPFVKLNVETDTAEIRLMCTETYLDTPRPNDGSITGPCESDGDKGSGGIKGGFVHQRMASKIGRWYSRTMSKTKRMGMGLYVKYDGDPWTDSEALALSGVMVSMEEPGWYSFGFEIKDKKCDVPCIGIEMVHDGGKTTWHSAATAIYCLMGSGQLLWDTVTGVDMAL |
| B/Sydney/207/2006 | 2006 | LPSTIQTLTLFLTSGGVLLSLYVSASLSYLLYSDILLKFSPTEITAPTMPLDCANASNVQAVNRSATKGVTLLLPEPEWTYPRLSCPGSTFQKALLISPHRFGETKGNSAPLIIREPFIACGPNECKHFALTHYAAQPGGYYNGTRGDRNKLRHLISVKLGKIPTVENSIFHMAAWSGSACHDGKEWTYIGVDGPDNNALLKIKYGEAYTDTYHSYANKILRTQESACNCIGGNCYLMITDGSASGVSECRFLKIREGRIIKEIFPTGRVKHTEECTCGFASNKTIECACRDNSYTAKRPFVKLNVETDTAEIRLMCTDTYLDTPRPDDGSITGPCESNGDKGSGGIKGGFVHQRMASKIGRWYSRTMSKTERMGMGLYVKYDGDPWADSDALAFSGVMVSMKEPGWYSFGFEIKDKKCDVPCIGIEMVHDGGKETWHSAATAIYCLMGSGQLLWDTVTGVDMAL |
| B/Sydney/507/2006 | 2006 | LPSTIQTLTLFLTSGGVLLSLYVSASLSYLLYSDILLKFSQTEITAPIMPLDCANASNVQAVNRSAAKGVTFLLPEPEWTYPRLSCPGSTFQKALLISPHRFGETKGNSAPLIIREPFIACGPTECKHFALTHYAAQPGGYYNGTREDRNKLRHLISVKLGKIPTVENSIFHMAAWSGSACHDGKEWTYIGVDGPDSNALLKIKYGEAYTDTYHSYAKNILRTQESACNCIGGDCYLMITDGPASGISECRFLKIREGRIIKEIFPTGRVKHTEECTCGFASNKTIECACRDNSYTAKRPFVKLNVETDTAEIRLMCTETYLDTPRPNDGSITGPCESDGDKGSGGIKGGFVHQRMASKIGRWYSRTMSKTKRMGMGLYVKYDGDPWTDSEALALSGVMVSMEEPGWYSFGFEIKDKKCDVPCIGIEMVHDGGKTTWHSAATAIYCLMGSGQLLWDTVTGVDMAL |
| B/Sydney/508/2006 | 2006 | LPSTIQTLTLFLTSGGVLLSLYVSASLSYLLYSDILLKFSPTEITAPTMPLDCANASNVQAVNRSATKGVTLLLPEPEWTYPRLSCPGSTFQKALLISPHRFGETKGNSAPLIIREPFIACGPNECKHFALTHYAAQPGGYYNGTRGDRNKLRHLISVKLGKIPTVENSIFHMAAWSGSACHDGKEWTYIGVDGPDNNALLKIKYGEAYTDTYHSYANKILRTQESACNCIGGNCYLMITDGSASGVSECRFLKIREGRIIKEIFPTGRVKHTEECTCGFASNKTIECACRDNSYTAKRPFVKLNVETDTAEIRLMCTDTYLDTPRPDDGSITGPCESNGDKGSGGIKGGFVHQRMASKIGRWYSRTMSKTERMGMGLYVKYDGDPWADSDALAFSGVMVSMKEPGWYSFGFEIKDKKCDVPCIGIEMVHDGGKETWHSAATAIYCLMGSGQLLWDTVTGVDMAL |
| B/Sydney/510/2006 | 2006 | LPSTIQTLTLFLTSGGVLLSLYVSASLSYLLYSDILLKFSPTEITAPTMPLDCANASNVQAVNRSATKGVTLLLSEPEWTYPRLSCPGSTFQKALLISPHRFGETKGNSAPLIIREPFIACGPNECKHFALTHYAAQPGGYYNGTRGDRNKLRHLISVKLGKIPTVENSIFHMAAWSGSACHDGKEWTYIGVDGPDNNALLKIKYGEAYTDTYHSYANKILRTQESACNCIGGNCYLMITDGSASGVSECRFLKIREGRIIKEIFPTGRVKHTEECTCGFASNKTIECACRDNSYTAKRPFVKLNVETDTAEIRLMCTDTYLDTPRPDDGSITGPCESNGDKGSGGIKGGFVHQRMASKIGRWYSRTMSKTERMGMGLYVKYDGDPWADSDALAFSGVMVSMKEPGWYSFGFEIKDKKCDVPCIGIEMVHDGGKETWHSAATAIYCLMGSGQLLWDTVTGVDMAL |
| B/Sydney/71/2006 | 2006 | LPSTIQTLTLFLTSGGVLLSLYVSASLSYLLYSDILLKFSPTEITAPTMPLDCANASNVQAVNRSATKGVTLLLPEPEWTYPRLSCPGSTFQKALLISPHRFGETKGNSAPLIIREPFIACGPNECKHFALTHYAAQPGGYYNGTRGDRNKLRHLISVKLGKIPTVENSIFHMAAWSGSACHDGKEWTYIGVDGPDNNALLKIKYGEAYTDTYHSYANKILRTQESACNCIGGNCYLMITDGSASGVSECRFLKIREGRIIKEIFPTGRVKHTEECTCGFASNKTIECACRDNSYTAKRPFVKLNVETDTAEIRLMCTDTYLDTPRPDDGSITGPCESNGDKGSGGIKGGFVHQRMASKIGRWYSRTMSKTERMGMGLYVKYDGDPWADSDALAFSGVMVSMKEPGWYSFGFEIKDKKCDVPCIGIEMVHDGGKETWHSAATAIYCLMGSGQLLWDTVTGVDMAL |
| B/Sydney/83/2006 | 2006 | LPSTIQTLTLFLTSGGVLLSLYVSASLSYLLYSDILLKFSPTEITAPTMPLDYANASNVQAVNRSATKGVTLLLPEPEWTYPRLSCPGSTFQKALLISPHRFGETKGNSAPLIIREPFIACGPNECKHFALTHYAAQPGGYYNGTRGDRNKLRHLISVKLGKIPTVENSIFHMAAWSGSACHDGKEWTYIGVDGPDNNALLKIKYGEAYTDTYHSYANKILRTQESACNCIGGNCYLMITDGSASGVSECRFLKIREGRITKEIFPTGRVKHTEECTCGFASNKTIECACRDNSYTAKRPFVKLNVETDTAEIRLMCTDTYLDTPRPDDGSITGPCESNGDKGSGGIKGGFVHQRMASKIGRWYSRTMSKTERMGMGLYVKYDGDPWADSDALAFSGVMVSMKEPGWYSFGFEIKDKKCDVPCIGIEMVHDGGKETWHSAATAIYCLMGSGQLLWDTVTGVDMAL |
| B/Sydney/9/2006 | 2006 | LPSTIQTLTLFLTSGGVLLSLYVSASLSYLLYSDILLKFPSTEITAPTMPLDCANASNVQAVNRSATKGVTLLLPEPEWTYPRLSCPGSTFQKALLISPHRFGETKGNSAPLIIREPFIACGPKECKHFALTHYAAQPGGYYNGTRGDRNKLRHLISVKLGKIPTVENSIFHMAAWSGSACHDGKEWTYIGVDGPDNNALLKIKYGEAYTDTYHSYANNILRTQESACNCIGGNCYLMITDGSASGVSECRFLKIREGRIIKEIFPTGRIKHTEECTCGFASNKTIECACRDNSYTAKRPFVKLNVETDTAEIRLMCTETYLDTPRPDDGSITGPCESNGDKGSGGIKGGFVHQRMASKIGRWYSRTMSKTERMGMGLYVKYDGDPWADSDALAFSGVMVSMEEPGWYSFGFEIKDKKCDVPCIGIEMVHDGGKETWHSAATAIYCLMGSGQLLWDTVTGVNMAL |
| B/Taiwan/2755/2006 | 2006 | LPSTIQTLTLFLTSGGVLLSLYVSASLSYLLYSDILLKFSPTEITAPTMPLDCANASNVQAVNRSATKGVTLLLPEPEWTYPRLSCPGSTFQKALLISPHRFGETKGNSAPLIIREPFIACGPNECKHFALTHYAAQPGGYYNGTRGDRNKLRHLISVKLGKIPTVENSIFHMAAWSGSACHDGKEWTYIGVDGPDNNALLKIKYGEAYTDTYHSYANKILRTQESACNCIGGNCYLMITDGSASGVSECRFLKIREGRIIKEIFPTGRVKHTEECTCGFASNKTIECACRDNSYTAKRPFVKLNVETDTAEIRLMCTDTYLDTPRPDDGSITGPCESNGDRGSGGIKGGFVHQRMASKIGRWYSRTMSKTERMGMGLYVKYDGDPWADSDALAFSGVMVSMKEPGWYSFGFEIKDKKCDVPCIGIEMVHDGGKETWHSAATAIYCLMGSGQLLWDTVTGVDMAL |
| B/Taiwan/2894/2006 | 2006 | LPSTIQTLTLFLTSGGVLLSLYVSASLSYLLYSDILLKFSPTEITAPTMPLDCANASNVQAVNRSATKGVTLLLPEPEWTYPRLSCPGSTFQKALLISPHRFGETKGNSAPLIIREPFIACGPNECKHFALTHYAAQPGGYYNGTRGDRNKLRHLISVKLGKIPTVENSIFHMAAWSGSACHDGKEWTYIGVDGPDNNALLKIKYGEAYTDTYHSYANKILRTQESACNCIGGNCYLMITDGSASGVSECRFLKIREGRIIKEIFPTGRVKHTEECTCGFASNKTIECACRDNSYTAKRPFVKLNVETDTAEIRLMCTDTYLDTPRPDDGSITGPCESNGDRGSGGIKGGFVHQRMASKIGRWYSRTMSKTERMGMGLYVKYDGDPWADSDALAFSGVMVSMKEPGWYSFGFEIKDKKCDVPCIGIEMVHDGGKETWHSAATAIYCLMGSGQLLWDTVTGVDMAL |
| B/Taiwan/3001/2006 | 2006 | LPSTIQTLTLFLTSGGVLLSLYVSASLSYLLYSDILLKFSQTEITAPIMPLDCANASNVQAVNRSAAKGVTLLLPEPEWTYPRLSCPGSTFQKALLINPHRFGETKGNSAPLIIREPFIACGPTECKHFALTHYAAQPGGYYNGTREDRNKLRHLISVKLGKIPTVENSIFHMAAWSGSACHDGKEWTYIGVDGPDSNALLKIKYGEAYTDTYHSYAKNILRTQESACNCIGGDCYLMITDGSASGISECRFLKIREGRIIKEIFPTGRVKHTEECTCGFASNKTIECACRDNSYTAKRPFVKLNVETDTAEIRLMCTETYLDTPRPNDGSITGPCESDGDKGSGGIKGGFVHQRMASKIGRWYSRTMSKTKRMGMGLYVKYDGDPWTDSEALALSGVMVSMEEPGWYSFGFEIKDKKCDVPCIGIEMVHDGGKTTWHSAATAIYCLMGSGQLLWDTVTGVDMAL |
| B/Taiwan/3290/2006 | 2006 | LPSTIQTLTLFLTSGGVLLSLYVSASLSYLLYSDILLKFSPTEITAPTMPLDCANASNVQAVNRSATKGVTLLLPEPEWTYPRLSCPGSTFQKALLISPHRFGETKGNSAPLIIREPFIACGPNECKHFALTHYAAQPGGYYNGTRGDRNKLRHLISVKLGKIPTVENSIFHMAAWSGSACHDGKEWTYIGVDGPDNNALLKIKYGEAYTDTYHSYANKILRTQESACNCIGGNCYLMITDGSASGVSECRFLKIREGRIIKEIFPTGRVKHTEECTCGFASNKTIECACRDNSYTAKRPFVKLNVETDTAEIRLMCTDTYLDTPRPDDGSITGPCESNGDRGSGGIKGGFVHQRMASKIGRWYSRTMSKTERMGMGLYVKYDGDPWADSDALAFSGVMVSMKEPGWYSFGFEIKDKKCDVPCIGIEMVHDGGKETWHSAATAIYCLMGSGQLLWDTVTGVDMAL |
| B/Victoria/100/2006 | 2006 | LPSTIQTLTLFLTSGGVLLSLYVSASLSYLLYSDILLKFSPTEITAPTMPLDCANASNVQAVNRSATKGVTLLLPEPEWTYPRLSCPGSTFQKALLISPHRFGETKGNSAPLIIREPFIACGPNECKHFALTHYAAQPGGYYNGTRGDRNKLRHLISVKLGKIPTVENSIFHMAAWSGSACHDGKEWTYIGVDGPDNNALLKIKYGEAYTDTYHSYANKILRTQESACNCIGGNCYLMITDGSASGVSECRFLKIREGRIIKEIFPTGRVKHTEECTCGFASNKTIECACRDNSYTAKRPFVKLNVETDTAEIRLMCTDTYLDTPRPDDGSITGPCESNGDKGSGGIKGGFVHQRMASKIGRWYSRTMSKTERMGMGLYVKYDGDPWADSDALAFSGVMVSMKEPGWYSFGFEIKDKKCDVPCIGIEMVHDGGKETWHSAATAIYCLMGSGQLLWDTVTGVDMAL |
| B/Victoria/303/2006 | 2006 | LPSTIQTLTLFLTSGGVLLSLYVLASLSYLLYSDILLKFSPTEITAPTMPLDCANASNVHAVNRSATKGVTLLLPEPEWAYPRLSCPGSTFQKALLISPHRFGETKGNSAPLIIREPFIACGPNECKHFALTHYAAQPGGYYNGTRGDRNKLRHLISVKLGKIPTVENSIFHMAAWSGSACHDGKEWTYIGVDGPDNNALLKIKYGEAYTDTYHSYANNILRTQESACNCIGGNCYLMITDGSASGVSECRFLKIREGRIIKEIFPTGRVKHTEECTCGFASNKTIECACRDNSYTAKRPFVKLNVETDTAEIRLMCTDTYLDTPRPDDGSITGPCESNGDKGSGGIKGGFVHQRMASKIGRWYSRTMSKTERMGMGLYVKYDGDPWADSDALAFSGVMVSMKEPGWYSFGFEIKDKKCDVPCIGIEMVHDGGKETWHSAATAIYCLMGSGQLLWDTVTGVDMAL |
| B/Victoria/309/2006 | 2006 | LPSTIQTLTLFLTSGGVLLSLYVSASLSYLLYSDILLKFSPTEITAPTMPLDCANASNVQAVNRSATKGVTLLLPEPEWTYPRLSCPGSTFQKALLISPHRFGETKGNSAPLIIREPFIACGPNECKHFALTHYAAQPGGYYNGTRGDRNKLRHLISVKLGKIPTVENSIFHMAAWSGSACHDGKEWTYIGVDGPDNNALLKIKYGEAYTDTYHSYANKILRTQESACNCIGGNCYLMITDGSASGVSECRFLKIREGRIIKEIFPTGRVKHTEECTCGFASNKTIECACRDNSYTAKRPFVKLNVETDTAEIRLMCTDTYLDTPRPDDGSITGPCESNGDKGSGGIKGGFVHQRMASKIGRWYSRTMSKTERMGMGLYVKYDGDPWADSDALAFSGVMVSMKEPGWYSFGFEIKDKKCDVPCIGIEMVHDGGKETWHSAATAIYCLMGSGQLLWDTVTGVDMAL |
| B/Victoria/329/2006 | 2006 | LPSTIQTLTLFLTSGGVLLSLYVSASLSYLLYSDILLKFSPTEITAPTMPLDCANASNVQAVNRSATKGVTPLLPEPEWTYPRLSCPGSTFQKALLISPHRFGETKGNSAPLIIREPFIACGPNECKHFALTHYAAQPGGYYNGTRGDRNKLRHLISVKLGKIPTVENSIFHMAAWSGSACHDGKEWTYIGVDGPDNNALLKIKYGEAYTDTYHSYANKILRTQESACNCIGGNCYLMITDGSASGVSECRFLKIREGRIIKEIFPTGRVKHTEECTCGFASNKTIECACRDNSYTAKRPFVKLNVETDTAEIRLMCTDTYLDTPRPDDGSITGPCESNGDKGSGGIKGGFVHQRMASKIGRWYSRTMSKTERMGMGLYVKYDGDPWADSDALAFSGVMVSMKEPGWYSFGFEIKDKKCDVPCIGIEMVHDGGKETWHSAATAIYCLMGSGQLLWDTVTGVDMAL |
| B/Victoria/330/2006 | 2006 | LPSTIQTLTLFLTSGGVLLSLYVSASLSYLLYSDILLKFSPTEITAPTMPLDCANASNVQAVNRSATKGVTLLLSEPEWTYPRLSCPGSTFQKALLISPHRFGETKGNSAPLIIREPFIACGPNECKHFALTHYAAQPGGYYNGTRGDRNKLRHLISVKLGKIPTVENSIFHMAAWSGSACHDGKEWTYIGVDGPDNNALLKIKYGEAYTDTYHSYANKILRTQESACNCIGGNCYLMITDGSASGVSECRFLKIREGRIIKEIFPTGRVKHTEECTCGFASNKTIECACRDNSYTAKRPFVKLNVETDTAEIRLMCTDTYLDTPRPDDGSITGPCESNGDKGSGGIKGGFVHQRMASKIGRWYSRTMSKTERMGMGLYVKYDGDPWADSDALAFSGVMVSMKEPGWYSFGFEIKDKKCDVPCIGIEMVHDGGKETWHSAATAIYCLMGSGQLLWDTVTGVDMAL |
| B/Victoria/332/2006 | 2006 | LPSTIQTLTLFLTSGGVLLSLYVSASLSYLLYSDILLKFSPTEITAPTMPLDCANASNVQAVNRSATKGVTLLLPEPEWTYPRLSCPGSTFQKALLISPHRFGETKGNSAPLIIREPFIACGPNECKHFALTHYAAQPGGYYNGTRGDRNKLRHLISVKLGKIPTVENSIFHMAAWSGSACHDGKEWTYIGVDGPDNNALLKIKYGEAYTDTYHSYANKILRTQESACNCIGGNCYLMITDGSASGVSECRFLKIREGRIIKEIFPTGRVKHTEECTCGFASNKTIECACRDNSYTAKRPFVKLNVETDTAEIRLMCTDTYLDTPRPDDGSITGPCESNGDKGSGGIKGGFVHQRMASKIGRWYSRTMSKTERMGMGLYVKYDGDPWADSDALAFSGVMVSMKEPGWYSFGFEIKDKKCDVPCIGIEMVHDGGKETWHSAATAIYCLMGSGQLLWDTVTGVDMAL |
| B/Victoria/333/2006 | 2006 | LPSTIQTLTLFLTSGGVLLSLYVSASLSYLLYSDILLKFSPTEITAPTMPLDCANASNVQAVNRSATKGVTLLLPEPEWTYPRLSCPGSTFQKALLISPHRFGETKGNSAPLIIREPFIACGPNECKHFALTHYAAQPGGYYNGTRGDRNKLRHLISVKLGKIPTVENSIFHMAAWSGSACHDGKEWTYIGVDGPDNNALLKIKYGEAYTDTYHSYANKILRTQESACNCIGGNCYLMITDGSASGVSECRFLKIREGRIIKEIFPTGRVKHTEECTCGFASNKTIECACRDNSYTAKRPFVKLNVETDTAEIRLMCTDTYLDTPRPDDGSITGPCESNGDKGSGGIKGGFVHQRMASKIGRWYSRTMSKTERMGMGLYVKYDGDPWADSDALAFSGVMVSMKEPGWYSFGFEIKDKKCDVPCIGIEMVHDGGKETWHSAATAIYCLMGSGQLLWDTVTGVDMAL |
| B/Victoria/501/2006 | 2006 | LPSTIQTLTLFLTSGGVLLSLYVSASLSYLLYSDILLKFSPTEITAPTMPLDCANASNVQAVNRSATKGVTLLLPEPEWTYPRLSCPGSTFQKALLISPHRFGETKGNSAPLIIREPFIACGPNECKHFALTHYAAQPGGYYNGTRGDRNKLRHLISVKLGKIPTVENSIFHMAAWSGSACHDGKEWTYIGVDGPDNNALLKIKYGEAYTDTYHSYANKILRTQESACNCIGGNCYLMITDGSASGVSECRFLKIREGRIIKEIFPTGRVKHTEECTCGFASNKTIECACRDNSYTAKRPFVKLNVETDTAEIRLMCTDTYLDTPRPDDGSITGPCESNGDKGSGGIKGGFVHQRMASKIGRWYSRTMSKTERMGMGLYVKYDGDPWADSDALAFSGVMVSMKEPGWYSFGFEIKDKKCDVPCIGIEMVHDGGKETWHSAATAIYCLMGSGQLLWDTVTGVDMAL |
| B/Victoria/505/2006 | 2006 | LPSTIQTLTLFLTSGGVLLSLYVSASLSYLLYSDILLKFSPTEITAPTMPLDCANASNVQAVNRSATKGVTLLLPEPEWTYPRLSCPGSTFQKALLISPHRFGETKGNSAPLIIREPFIACGPNECKHFALTHYAAQPGGYYNGTRGDRNKLRHLISVKLGKIPTVENSIFHMAAWSGSACHDGKEWTYIGVDGPDNNALLKIKYGEAYTDTYHSYANKILRTQESACNCIGGNCYLMITDGSASGVSECRFLKIREGRIIKEIFPTGRVKHTEECTCGFASNKTIECACRDNSYTAKRPFVKLNVETDTAEIRLMCTDTYLDTPRPDDGSITGPCESNGDKGSGGIKGGFVHQRMASKIGRWYSRTMSKTERMGMGLYVKYDGDPWADSDALAFSGVMVSMKEPGWYSFGFEIKDKKCDVPCIGIEMVHDGGKETWHSAATAIYCLMGSGQLLWDTVTGVDMAL |
| B/Waikato/1/2006 | 2006 | LPSTIQTLTLFLTSGGVLLSLYVSASLSYLLYSDILLKFSQTEITAPIMPLDCANASNVQAVNRSAAKGVTLLLPEPEWTYPRLSCPGSTFQKALLISPHRFGETKGNSAPLIIREPFIACGPTECKHFALTHYAAQPGGYYNGTREDRNKLRHLISVKLGKIPTVENSIFHMAAWSGSACHDGKEWTYVGVDGPDSNALLKIKYGEAYTDTYHSYAKNILRTQESACNCIGGDCYLMITDGPASGISECRFLKIREGRIIKEIFPTGRVKHTEECTCGFASNKTIECACRDNSYTAKRPFVKLNVETDTAEIRLMCTETYLDTPRPNDGSITGPCESDGDKGSGGIKGGFVHQRMASKIGRWYSRTMSKTKRMGMGLYVKYDGDPWTDSEALALSGVMVSMEEPGWYSFGFEIKDKKCDVPCIGIEMVHDGGKTTWHSAATAIYCLMGSGQLLWNTVTGVDMAL |
| B/beijingxicheng/514/2007 | 2007 | LPSTIQTLTLFLTSGGVLLSLYVSASLSYLLYSDILLKFSQTEIAAPIMPLDCANASNVQAVNRSAAKGVTLLLPEPEWTYPRLSCPGSTFQKALLISPHRFGETKGNSAPLIIREPFIACGPTECKHFALTHYAAQPGGYYNGTREDRNKLRHLISVKLGKIPTVENSIFHMAAWSGSACHDGKEWTYIGVDGPDSNALLKIKYGEAYTDTYHSYAKNILRTQESACNCIGGDCYLMITDGPASGVSECRFLKIREGRIIKEIFPTGRVKHTEECTCGFASNKTIECACRDNSYTAKRPFVKLNVETDTAEIRLMCTETYLDTPRPNDGSITGPCESDGDKGSGGIKGGFVHQRMASKIGRWYSRTMSKTKRMGMGLYVKYDGDPWTDSEALALSGVMVSMEEPGWYSFGFEIKDKKCDVPCIGIEMVHDGGKTTWHSAATAIYCLMGSGQLLWDTVTGVDMAL |
| B/Brisbane/1/2007 | 2007 | LPSTIQTLTLFLTSGGVLLSLYVSASLSYLLYSDILLKFSQTEITAPIMPLDCANASNVQAVNRSAAKGVTLLLPEPEWTYPRLSCPGSTFQKALLISPHRFGETKGNSAPLIIREPFIACGPTECKHFALTHYAAQPGGYYNGTREDRNKLRHLISVKLGKIPTVENSIFHMAAWSGSACHDGKEWTYIGVDGPDSNALLKIKYGEAYTDTYHSYAKNILRTQESACNCIGGDCYLMITDGPASGVSECRFLKIREGRIIKEIFPTGRVKHTEECTCGFASNKTIECACRDNSYTAKRPFVKLNVETDTAEIRLMCTETYLDTPRPNDGSITGPCESDGDKGSGGIKGGFVHQRMASKIGRWYSRTMSKTKRMGMGLYVKYDGDPWTDSEALALSGVMVSMEEPGWYSFGFEIKDKKCDVPCIGIEMVHDGGKTTWHSAATAIYCLMGSGQLLWDTVTGVDMAL |
| B/Brisbane/10/2007 | 2007 | LPSTIQTLTLFLTSGGVLLSLYVSASLSYLLYSDILLKFSPTEIIAPTMPLDCANASNVHAVNRSATKGVTLLLPEPEWTYPRLSCPGSTFQKALLISPHRFGETKGNSAPLIIREPFIACGPNECKHFALTHYAAQPGGYYNGTRGNRNKLRHLISVKLGKIPTVENSIFHMAAWSGSACHDGKEWTYIGVDGPDNNALLKIKYGEAYTDTYHSYANNILRTQESACNCIGGNCYLMITDGSASGVSECRFLKIREGRIIKEIFPTGRVKHTEECTCGFASNKTIECACRDNSYTAKRPFVKLNVETDTAEIRLMCTDTYLDTPRPDDGSITGPCESNGDKGSGGIKGGFVHQRMASKIGRWYSRTMSKTERMGMGLYVKYDGDPWADSDALAFSGVMVSMKEPGWYSFGFEIKDKKCDVPCIGIEMVHDGGKETWHSAATAIYCLMGSGQLLWDTVTGVDMAL |
| B/Brisbane/12/2007 | 2007 | LPSTIQTLTLFLTSGGVLLSLYVSASLSYLLYSDILLKFSQTEITAPIMPLDCANASNVQAVNRSAAKGVTLLLSEPEWTYPRLSCPGSTFQKALLISPHRFGETKGNSAPLIIREPFIACGPTECKHFALTHYAAQPGGYYNGTREDRNKLRHLISVKLGKIPTVENSIFHMAAWSGSACHDGKEWTYIGVDGPDSNALLKIKYGEAYTDTYHSYAKNILRTQESACNCIGGDCYLMITDGPASGVSECRFLKIREGRIIKEIFPTGRVKHTEECTCGFASNKTIECACRDNSYTAKRPFVKLNVETDTAEIRLMCTETYLDTPRPNDGSITGPCESDGDKGSGGIKGGFVHQRMASKIGRWYSRTMSKTKRMGMGLYVKYDGDPWTDSEALALSGVMVSMEEPGWYSFGFEIKDKKCDVPCIGIEMVHDGGKTTWHSAATAIYCLMGSGQLLWDTVTGVDMAL |
| B/Brisbane/24/2007 | 2007 | LPSTIQTLTLFLTSGGVLLSLYVSASLSYLLYSDILLKFSQTEITAPIMPLDYANASNVQAVNHSAAKGVTLLLPEPEWTYPRLSCPGSTFQKALLISPHRFGETKGNSAPLIIREPFIACGPTECKHFALTHYAAQPGGYYNGTREDRNKLRHLISVKLGKIPTVENSIFHMAAWSGSACHDGKEWTYIGVDGPDSNALLKIKYGEAYIDTYHSYAKNILRTQESACNCIGGDCYLMITDGPASGISECRFLKIREGRIIKEIFPTGRVKHTEECTCGFASNKTIECACRDNSYTAKRPFVKLNVETDTAEIRLMCTETYLDTPRPNDGSITGPCESDGDKGSGGIKGGFVHQRMASKIGRWYSRTMSKTKRMGMGLYVKYDGDPWTDSEALALSGVMVSMEEPGWYSFGFEIKDKKCDVPCIGIEMVHDGGKTTWHSAATAIYCLMGSGQLLWDTVTGVDMAL |
| B/Brisbane/25/2007 | 2007 | LPSTIQTLTLFLTSGGVLLSLYVSASLSYLLYSDILLKFSRTEITAPIMPLDCANASNVQAVNRSATKGVTLLLPEPEWTYPRLSCPGSTFQKALLISPHRFGETKGNSAPLIIREPFIACGPKECKHFALTHYAAQPGGYYNGTREDRNKLRHLISVKLGKIPTVENSIFHMAAWSGSACHDGREWTYIGVDGPDSNALLKIKYGEAYTDTYHSYAKNILRTQESACNCIGGDCYLMITDGPASGISECRFLKIREGRIIKEIFPTGRVKHTEECTCGFASNKTIECACRDNSYTAKRPFVKLNVETDTAEIRLMCTETYLDTPRPNDGSITGPCESNGDKGSGGIKGGFVHQRMASKIGRWYSRTMSKTKRMGMGLYVKYDGDPWTDSEALALSGVMVSMEEPGWYSFGFEIKDKKCDVPCIGIEMVHDGGKTTWHSAATAIYCLMGSGQLLWDTVTGVNMTL |
| B/Brisbane/7/2006 | 2007 | LPSTIQTLTLFLTSGGVLLSLYVSASLSYLLYSDILLKFSPTEITAPTMPLDCANASNVQAVNRSATKGVTPLLPEPEWTYPRLSCPGSTFQKALLISPHRFGETKGNSAPLIIREPFIACGPNECKHFALTHYAAQPGGYYNGTRGDRNKLRHLISVKLGKIPTVENSIFHMAAWSGSACHDGKEWTYIGVDGPDNNALLKIKYGEAYTDTYHSYANKILRTQESACNCIGGNCYLMITDGSASGVSECRFLKIREGRIIKEIFPTGRVKHTEECTCGFASNKTIECACRDNSYTAKRPFVKLNVETDTAEIRLMCTDTYLDTPRPDDGSITGPCESNGDKGSGGIKGGFVHQRMASKIGRWYSRTMSKTERMGMGLYVKYDGDPWADSDALAFSGVMVSMKEPGWYSFGFEIKDKKCDVPCIGIEMVHDGGKETWHSAATAIYCLMGSGQLLWDTVTGVDMAL |
| B/Brisbane/7/2007 | 2007 | IPSTIQTLTLFLTSGGVLLSLYVSASLSYLLYSDILLKFSPTEIIAPTMPLDCANASNVHAVNRSATKGVTLLLPEPEWTYPRLSCPGSTFQKALLISPHRFGETKGNSAPLIIREPFIACGPNECKHFALTHYAAQPGGYYNGTRGDRNKLRHLISVKLGKIPTVENSIFHMAAWSGSACHDGKEWTYIGVDGPDNNALLKIKYGEAYTDTYHSYANNILRTQESACNCIGGNCYLMITDGSASGVSECRFLKIREGRIIKEIFPTGRVKHTEECTCGFASNKTIECACRDNSYTAKRPFVKLNVETDTAEIRLMCTDTYLDTPRPDDGSITGPCESNGDKGSGGIKGGFVHQRMASKIGRWYSRTMSKTERMGMGLYVKYDGDPWADSDALAFSGVMVSMKEPGWYSFGFEIKDKKCDVPCIGIEMVHDGGKETWHSAATAIYCLMGSGQLLWDTVTGVDMAL |
| B/Christchurch/105/2007 | 2007 | LPSTIQTLTLFLTSGGVLLSLYVSASLSYLLYSDILLKFSQTEITAPIMPLDCANASNVQAVNRSAAKGVTLLLPEPEWTYPRLSCPGSTFQKALLISPHRFGETKGNSAPLIIREPFIACGPTECKHFALTHYAAQPGGYYNGTREDRNKLRHLISVKLGKIPTVENSIFHMAAWSGSACHDGKEWTYVGVDGPDSNALLKIKYGEAYTDTYHSYAKNILRTQESACNCIGGDCYLMITDGPASGISECRFLKIREGRIIKEIFPTGRVKHTEECTCGFASNKTIECACRDNSYTAKRPFVKLNVETDTAEIRLMCTETYLDTPRPNDGSITGPCGSDGDKGSGGIKGGFVHQRMASKIGRWYSRTMSKTKRMGMGLYVKYDGDPWTDSEALALSGVMVSMDEPGWYSFGFEIKDKKCDVPCIGIEMVHDGGKTTWHSAATAIYCLMGSGQLLWDTVTGVDMAL |
| B/Christchurch/18/2007 | 2007 | LPSTIQTLTLFLTSGGVLLSLYVSASLSYLLYSDILLKFSQTEITAPIMPLDCANASNVQAVNRSAAKGVTLLLPEPEWTYPRLSCPGSTFQKALLISPHRFGETKGNSAPLIIREPFIACGPTECKHFALTHYAAQPGGYYNGTREDRNKLRHLISVKLGKIPTVENSIFHMAAWSGSACHDGKEWTYVGVDGPDSNALLKIKYGEAYTDTYHSYAKNILRTQESACNCIGGDCYLMITDGPASGISECRFLKIREGRIIKEIFPTGRVKHTEECTCGFASNKTIECACRDNSYTAKRPFVKLNVETDTAEIRLMCTETYLDTPRPNDGSITGPCGSDGDKGSGGIKGGFVHQRMASKIGRWYSRTMSKTKRMGMGLYVKYDGDPWTDSEALALSGVMVSMDEPGWYSFGFEIKDKKCDVPCIGIEMVHDGGKTTWHSAATAIYCLMGSGQLLWDTVTGVDMAL |
| B/Christchurch/2/2007 | 2007 | LPSTIQTLTLFLTSGGVLLSLYVSASLSYLLYSDILLKFSRTEITAPIMPLDCANASNIQAVNRSATKGVTLLLPEPEWTYPRLSCPGSTFQKALLISPHRFGETKGNSAPLIIREPFIACGPKECKHFALTHYAAQPGGYYNGTREDRNKLRHLISVKLGKIPTVENSIFHMAAWSGSACHDGREWTYIGVDGPDSNALLKIKYGEAYTDTYHSYAKNILRTQESACNCIGGDCYLMITDGPASGISECRFLKIREGRIIKEIFPTGRVKHTEECTCGFASNKTIECACRDNSYTAKRPFVKLNVETDTAEIRLMCTETYLDTPRPNDGSITGPCESNGDKGSGGIKGGFVHQRMASKIGRWYSRTMSKTKRMGMGLYVKYDGDPWTDSEALALSGVMVSIEEPGWYSFGFEIKDKKCDVPCIGIEMVHDGGKTTWHSAATAIYCLMGSGQLLWDTVTGVDMAL |
| B/Christchurch/200/2007 | 2007 | LPSTIQTLTLFLTSGGVLLSLYVSASLSYLLYSDILLKFSQTEITAPIMPLDCANASNVQAVNRSAAKGVTLLLPEPEWTYPRLSCPGSTFQKALLISPHRFGETKGNSAPLIIREPFIACGPTECKHFALTHYAAQPGGYYNGTREDRNKLRHLISVKLGKIPTVENSIFHMAAWSGSACHDGKEWTYVGVDGPDSNALLKIKYGEAYTDTYHSYAKNILRTQESACNCIGGDCYLMITDGPASGISECRFLKIREGRIIKEIFPTGRVKHTEECTCGFASNKTIECACRDNSYTAKRPFVKLNVETDTAEIRLMCTETYLDTPRPNDGSITGPCGSDGDKGSGGIKGGFVHQRMASKIGRWYSRTMSKTKRMGMGLYVKYDGDPWTDSEALALSGVMVSMDEPGWYSFGFEIKDKKCDVPCIGIEMVHDGGKTTWHSAATAIYCLMGSGQLLWDTVTGVDMAL |
| B/Egypt/80/2007 | 2007 | LPSTIQMLTLFLTSGGVLLSLYVSASLSYLLYSDILLKFSPTEITAPTMPLDCANASNVQAVNRSATKGVTLLLPEPEWTYPRLSCPGSTFQKALLISPHRFGETKGNSAPLIIREPFIACGPNECKHFALTHYAAQPGGYYNGTRGDRNKLRHLISVKLGKIPTVENSIFHMAAWSGSACHDGKEWTYIGVDGPDNNALLKIKYGEAYTDTYHSYANKILRTQESACNCIGGNCYLMITDGSASGVSECRFLKIREGRIIKEIFPTGRVKHTEECTCGFASNKTIECACRDNSYTAKRPFVKLNVETDTAEIRLMCTDTYLDTPRPDDGSITGPCESNGDKGSGGIKGGFVHQRMASKIGRWYSRTMSKTERMGMGLYVKYDGDPWADSDALAFSGVMVSMKEPGWYSFGFEIKDKKCDVPCIGIEMVHDGGKETWHSAATAIYCLMGSGQLLWDTVTGVDMAL |
| B/heilongjiangdaoli/1189/2007 | 2007 | LPSTIQTLTLFLTSGGVLLSLYVSASLSYLLYSDILLKFSQTEITAPIMPLDCANASNVQAVNRSAAKGVTLLLPEPEWTYPRLSCPGSTFQKALLISPHRFGETKGNSAPLIIREPFIACGPTECKHFALTHYAAQPGGYYNGTREDRNKLRHLISVKLGKIPTVENSIFHMAAWSGSACHDGKEWTYIGVDGPDSNALLKIKYGEAYTDTYHSYAKNILRTQESACNCIGGDCYLMITDGPASGVSECRFLKIREGRIIKEIFPTGRVKHTEECTCGFASNKTIECACRDNSYTAKRPFVKLNVETDTAEIRLMCTETYLDTPRPNDGSITGPCESDGDKGSGGIKGGFVHQRMASKIGRWYSRTMSKTKRMGMGLYVKYDGDPWTDSEALALSGVMVSMEEPGWYSFGFEIKDKKCDVPCIGIEMVHDGGKTTWHSAATAIYCLMGSGQLLWDTVTGVDMAL |
| B/henanjinshui/1161/2007 | 2007 | LPSTIQTLTLFLTSGGVLLSLYVSASLSYLLYSDILLKFSQTEITAPIMSLDCANASNVQAVNRSAAKGVTLLLPEPEWTYPRLSCPGSTFQKALLISPHRFGETKGNSAPLIIREPFIACGPTECKHFALTHYAAQPGGYYNGTREDRNKLRHLISVKLGKIPTVENSIFHMAAWSGSACHDGKEWTYIGVDGPDSNALLKIKYGEAYTDTYHSYAKNILRTQESACNCIGGDCYLMITDGPASGVSECRFLKIREGRIIKEIFPTGRVKHTEECTCGFASNKTIECACRDNSYTAKRPFVKLNVETDTAEIRLMCTETYLDTPRPNDGSITGPCESDGDKGSGGIKGGFVHQRMASKIGRWYSRTMSKTKRMGMGLYVKYDGDPWTDSEALALSGVMVSMEEPGWYSFGFEIKDKKCDVPCIGIEMVHDGGKTTWHSAATAIYCLMGSGQLLWDTVTGVDMAL |
| B/hubeimaojian/1367/2007 | 2007 | LPSTIQTLTLFLTSGGVLLSLYVSASLSYLLYSDILLKFSQTEITAPIMPLDCANASNVQAVNRSAAKGVTLLLPEPEWTYPRLSCPGSTFQKALLISPHRFGETKGNSAPLIIREPFIACGPTECKHFALTHYAAQPGGYYNGTREDRNKLRHLISVKLGKIPTVENSIFHMAAWSGSACHDGKEWTYIGVDGPDSNALLKIKYGEAYTDTYHSYAKNILRTQESACNCIGGDCYLMITDGPASGVSECRFLKIREGRIIKEIFPTGRVKHTEECTCGFASNKTIECACRDNSYTAKRPFVKLNVETDTAEIRLMCTETYLDTPRPNDGSITGPCESDGDKGSGGIKGGFVHQRMASKIGRWYSRTMSKTKRMGMGLYVKYDGDPWTDSEALALSGVMVSMEEPGWYSFGFEIKDKKCDVPCIGIEMVHDGGKTTWHSAATAIYCLMGSGQLLWDTVTGVDMAL |
| B/jiangxidonghu/1782/2007 | 2007 | LPSTIQTLTLFLTSGGVLLSLYVSASLSYLLYSDILLKFPSTEITAPTMPLDCANASNVQAVNRSATKGVTFLLPEPEWTYPRLSCPGSTFQKALLISPHRFGETKGNSAPLIIREPFIACGPKECKHFALTHYAAQPGGYYNGTRGDRNKLRHLISVKLGKIPTVENSIFHMAAWSGSACHDGKEWTYIGVDGPDNNALLKIKYGEAYTDTYHSYANNILRTQESACNCIGGNCYLMITDGSASGVSECRFLKIREGRIIKEIFPTGRIKHTEECTCGFASNKTIECACRDNSYTAKRPFVKLNVETDTAEIRLMCTETYLDTPRPDDGSITGPCESNGGKGSGGIKGGFVHQRMASKIGRWYSRTMSKTERKGMGLYVKYDGDPWADSDALAFSGVMVPMEEPGWYSFGFEIKDKKCDVPCIGIEMVHDGGKETWHSAATAIYCLMGSGQLLWDTVTGVNMAL |
| B/jilinchaoyang/1320/2007 | 2007 | LPSTIQTLTLFLTSGGVLLSLYVSASLSYLLYSDILLKFPSTEITAPTMPLDCANASNVQAVNRSATKGVTFLLPEPEWTYPRLSCPGSTFQKALLISPHRFGETKGNSAPLIIREPFIACGPKECKHFALTHYAAQPGGYYNGTRGDRNKLRHLISVKLGKIPTVENSIFHMAAWSGSACHDGKEWTYIGVDGPDNNALLKIKYGEAYTDTYHSYANNILRTQESACNCIGGNCYLMITDGSASGVSECRFLKIREGRIIKEIFPTGRIKHTEECTCGFASNKTIECACRDNSYTAKRPFVKLNVETDTAEIRLMCTETYLDTPRPDDGSITGPCESNGGKGSGGIKGGFVHQRMASKIGRWYSRTMSKTERKGMGLYVKYDGDPWADSDALVFSGVMVSMEEPGWYSFGFEIKDKKCDVPCIGIEMVHDGGKETWHSAATAIYCLMGSGQLLWDTVTGVNMAL |
| B/Kol/1013/2007 | 2007 | LPSTIQTLTLFLTSGGVLLSLYVSASLSYLLYSDILLKFSRTEITAPIMPLDCANASNVQAVNRSATKGVTLLLPEPEWTYPRLSCPGSTFQKALLISPHRFGETKGNSAPLIIREPFIACGPKECKHFALTHYAAQPGGYYNGTREDRNKLRHLISVKLGKIPTVENSIFHMAAWSGSACHDGREWTYIGVDGPDSNALLKIKYGEAYTDTYHSYAKNILRTQESACNCIGGDCYLMITDGPASGISECRFLKIREGRIIKEIFPTGRVKHTEECTCGFASNKTIECACRDNSYTAKRPFVKLNVETDTAEIRLMCTETYLDTPRPNDGSITGPCESDGDKGSGGIKGGFVHQRMASKIGRWYSRTMSKTKRMGMGLYVKYDGDPWTDSEALALSGVMVSMEEPGWYSFGFEIKDKKCDVPCIGIEMVHDGGKTTWHSAATAIYCLMGSGQLLWDTVTGVDMAL |
| B/Malaysia/1723975/2007 | 2007 | LPSTIQTLTLFLTSGGVLLSLYVSASLSYLLYSDILLKFPSTEITAPTMPLDCANASNVQAVNRSATKGVTLLLPEPEWTYPRLSCPGSTFQKALLISPHRFGETKGNSAPLIIREPFIACGPKECKHFALTHYAAQPGGYYNGTRGDRNKLRHLISVKLGKIPTVENSIFHMAAWSGSACHDGKEWTYIGVDGPDNNALLKIKYGEAYTDTYHSYANNILRTQESACNCIGGNCYLMITDGSASGVSECRFLKIREGRIIKEIFPTGRIKHTEECTCGFASNKTIECACRDNSYTAKRPFVKLNVETDTAEIRLMCTETYLDTPRPDDGSITGPCESNGDKGSGGIKGGFVHQRMASKIGRWYSRTMSKTERMGMGLYVKYDGDPWADSDALTFSGVMVSMEEPGWYSFGFEIKDKKCDVPCIGIEMVHDGGKETWHSAATAIYCLMGSGQLLWDTVTGVNMAL |
| B/Malaysia/1759914/2007 | 2007 | LPSTIQTLTLFLTSGGVLLSLYVSASLSYLLYSDILLKFSQTEITAPIMPLDCANASNVQAVNRSAAKGVTLLLPEPEWTYPRLSCPGSTFQKALLISPHRFGETKGNSAPLIIREPFIACGPTECKHFALTHYAAQPGGYYNGTREDRNKLRHLISVKLGKIPTVENSIFHMAAWSGSACHDGKEWTYIGVDGPDSNALLKIKYGEAYTDTYHSYAKNILRTQESACNCIGGDCYLMITDGPASGISECRFLKIREGRIIKEIFPTGRVKHTEECTCGFASNKTIECACRDNSYTAKRPFVKLNVETDTAEIRLMCTETYLDTPRPNDGSITGPCESDGDKGSGGIKGGFVHQRMASKIGRWYSRTMSKTKRMGMGLYVKYDGDPWTDSEALALSGVMVSMEEPGWYSFGFEIKDKKCDVPCIGIEMVHDGGKTTWHSAATAIYCLMGSGQLLWDTVTGVDMAL |
| B/Malaysia/1819373/2007 | 2007 | LPSTIQTLTLFLTSGGVLLSLYVSTSLSYLLYSDILLKFSQTEITAPIMPLDCANASNVQAVNRSAAKGVTLLLPEPEWTYPRLSCPGSTFQKALLISPHRFGETKGNSAPLIIREPFIACGPTECKHFALTHYAAQPGGYYNGTREDRNKLRHLISVKLGKIPTVENSIFHMAAWSGSACHDGKEWTYIGVDGPDSNALLKIKYGEAYTDTYHSYAKNILRTQESACNCIGGDCYLMITDGPASGISECRFLKIREGRIIKEIFPTGRVKHTEECTCGFASNKTIECACRDNSYTAKRPFVKLNVETDTAEIRLMCTETYLDTPRPNDGSITGPCESDGDKGSGGIKGGFVHQRMASKIGRWYSRTMSKTKRMGMGLYVKYDGDPWTDSEALALSGVMVSMEEPGWYSFGFEIKDKKCDVPCIGIEMVHDGGKTTWHSAATAIYCLMGSGQLLWDTVTGVDMAL |
| B/Malaysia/1887865/2007 | 2007 | LPSTIQTLTLFLTSGGVLLSLYVSASLSYLLYSDILLKFSQTEITAPIVPLDCANASNVQAVNRSAAKGVTLLLPEPEWTYPRLSCPGSTFQKALLISPHRFGETKGNSAPLIIREPFIACGPTECKHFALTHYAAQPGEYYNGTREDRNKLRHLISVKLGKIPTVENSIFHMAAWSGSACHDGKEWTYIGVDGPDSNALLKIKYGEAYTDTYHSYAKNILRTQESACNCIGGDCYLMITDGPASGISECRFLKIREGRIIKEIFPTGRVKHTEECTCGFASNKTIECACRDNSYTAKRPFVKLNVETDTAEIRLMCTETYLDTPRPNDGSITGPCESDGDKGSGGIKGGFVHQRMASKIGRWYSRTMSKTKRMGMGLYVKYDGDPWTDSEALALSGVMVSMEEPGWYSFGFEIKDKKCDVPCIGIEMVHDGGKTTWHSAATAIYCLMGSGQLLWDTVTGVDMAL |
| B/Malaysia/1918984/2007 | 2007 | LPSTIQTLTLFLTSGGVLLSLYVSASLSYLLYSDILLKFSRTEITAPIMPLDCANASNVQAVNRSATKGVTLLLPEPEWTYPRLSCPGSTFQKALLISPHRFGETKGNSAPLIIREPFIACGPKECKHFALTHYAAQPGGYYNGTREDRNKLRHLISVKLGKIPTVENSIFHMAAWSGSACHDGREWTYIGVDGPDSNALLKIKYGEAYTDTYHSYAKNILRTQESACNCIGGDCYLMITDGPASGISECRFLKIREGRIIKEIFPTGRVKHTEECTCGFASNKTIECACRDNSYTAKRPFVKLNVETDTAEIRLMCTETYLDTPRPNDGSITGPCESNGDKGSGGIKGGFVHQRMASKIGRWYSRTMSKTKRMGMGLYVKYDGDPWTDSEALALSGVMVSMEEPGWYSFGFEIKDKKCDVPCIGIEMVHDGGKTTWHSAATAIYCLMGSGQLLWDTVTGVNMTL |
| B/Mississippi/UR06-0446/2007 | 2007 | LPSTIQTLTLFLTSGGVLLSLYVSASLSYLLYSDILLKFSPTEITAPTMPLDCANASNVQAVNRSATKGVTLLLPEPEWTYPRLSCPGSTFQKALLISPHRFGETKGNSAPLIIREPFIACGPNECKHFALTHYAAQPGGYYNGTRGDRNKLRHLISVKLGKIPTVENSIFHMAAWSGSACHDGKEWTYIGVDGPDNNALLKIKYGEAYTDTYHSYASKILRTQESACNCIGGNCYLMITDGSASGVSECRFLKIREGRIIKEIFPTGRVKHTEECTCGFASNKTIECACRDNSYTAKRPFVKLNVETDTAEIRLMCTDTYLDTPRPDDGSITGPCESNGDKGSGGIKGGFVHQRMASKIGRWYSRTMSKTERMGMGLYVKYDGDPWADSDALAFSGVMVSMKEPGWYSFGFEIKDKKCDVPCIGIEMVHDGGKETWHSAATAIYCLMGSGQLLWDTVTGVDMAL |
| B/Mississippi/UR06-0477/2007 | 2007 | LPSTIQTLTLFLTSGGVLLSLYVSASLSYLLYSDILLKFSPTEITAPTMPLDCANASNVHAVNRSATKGVTLLLPEPEWTYPRLSCPGSTFQKALLISPHRFGETKGNSAPLIIREPFIACGPNECKHFALTHYAAQPGGYYNGTRGDRNKLRHLISVKLGKIPTVENSIFHMAAWSGSACHDGKEWTYIGVDGPDNNALLKIKYGEAYTDTYHSYANNILRTQESACNCIGGNCYLMITDGSASGVSECRFLKIREGRIIKEIFPTGRVKHTEECTCGFASNKTIECACRDNSYTAKRPFVKLNVETDTAEIRLMCTDTYLDTPRPDDGSITGPCEFNGDKGSGGIKGGFVHQRMASKIGRWYSRTMSKTERMGMGLYVKYDGDPWADSDALAFSGVMVSMKEPGWYSFGFEIKDKKCDVPCIGIEMVHDGGKETWHSAATAIYCLMGSGQLLWDTVTGVDMAL |
| B/Mississippi/UR06-0492/2007 | 2007 | LPSTIQTLTLFLTSGGVLLSLYVSASLSYLLYSDILLKFSPTEITAPTMPLDCANASNVQAVNRSATKGVTLLLPEPEWTYPRLSCPGSTFQKALLISPHRFGETKGNSAPLIIREPFIACGPNECKHFALTHYAAQPGGYYNGTRGDRNKLRHLISVKLGKIPTVENSIFHMAAWSGSACHDGKEWTYIGVDGPDNNALLKIKYGEAYTDTYHSYANKILRTQESACNCIGGNCYLMITDGSASGVSECRFLKIREGRIIKEIFPTGRVKHTEECTCGFASNKTIECACRDNSYTAKRPFVKLNVETDTAEIRLMCTDTYLDTPRPDDGSITGPCESNGDKGSGGIKGGFVHQRMASKIGRWYSRTMSKTERMGMGLYVKYDGDPWADSDALAFSGVMVSMKEPGWYSFGFEIKDKKCDVPCIGIEMVHDGGKETWHSAATAIYCLMGSGQLLWDTVTGVDMAL |
| B/New York/1101/2007 | 2007 | LPSTIQTLTLFLTSGGVLLSLYVSASLSYLLYSDILLKFSPTEITAPTMPLDCANASNVQAVNHSATKGVTLLLPEPEWTYPRLSCPGSTFQKALLISPHRFGETKGNSAPLIIREPFIACGPNECKHFALTHYAAQPGGYYNGTRGDRNKLRHLISVKLGKIPTVENSIFHMAAWSGSACHDGKEWTYIGVDGPDNNALLKIKYGEAYTDTYHSYANKILRTQESACNCIGGNCYLMITDGSASGVSECRFLKIREGRIIKEIFPTGRVKHTEECTCGFASNKTIECACRDNSYTAKRPFVKLNVETDTAEIRLMCTDTYLDTPRPDDGSITGPCESNGDKGSGGIKGGFVHQRMASKIGRWYSRTMSKTERMGMGLYVKYDGDPWADSDALAFSGVMVSMKEPGWYSFGFEIKDKKCDVPCIGIEMVHDGGKETWHSAATAIYCLMGSGQLLWDTVTGVDMAL |
| B/New York/1103/2007 | 2007 | LPSTIQTLTLFLTSGGVLLSLYVSASLSYLLYSDILLKFSPTEITAPTMPLDCANASNVHAVNRSATKGVTLLLPEPEWTYPRLSCPGSTFQKALLISPHRFGETKGNSAPLIIREPFIACGPNECKHFALTHYAAQPGGYYNGTRGDRNKLRHLISVKLGKIPTVENSIFHMAAWSGSACHDGKEWTYIGVDGPDNNALLKIKYGEAYTDTYHSYANNILRTQESACNCIGGNCYLMITDGSASGVSECRFLKIREGRIIKEIFPTGRVKHTEECTCGFASNKTIECACRDNSYTAKRPFVKLNVETDTAEIRLMCTDTYLDTPRPDDGSITGPCESNGDKGGGGIKGGFVHQRMASKIGRWYSRTMSKTERMGMGLYVKYDGDPWADSDALAFSGVMVSLKEPGWYSFGFEIKDKKCDVPCIGIEMVHDGGKETWHSAATAIYCLMGSGQLLWDTVTGVDMAL |
| B/New York/1109/2007 | 2007 | LPSTIQTLTLFLTSGGVLLSLYVSASLSYLLYSDILLKFSPTEITAPTMPLDCANASNVQAVNHSATKGVTLLLPEPEWTYPRLSCPGSTFQKALLVSPHRFGETKGNSAPLIIREPFIACGPNECKHFALTHYAAQPGGYYNGTRGDRNKLRHLISVKLGKIPTVENSIFHMAAWSGSACHDGKEWTYIGVDGPDNNALLKIKYGEAYTDTYHSYANKILRTQESACNCIGGNCYLMITDGSASGVSECRFLKIREGRIIKEIFPTGRVKHTEECTCGFASNKTIECACRDNSYTAKRPFVKLNVETDTAEIRLMCTDTYLDTPRPDDGSITGPCESNGDKGSGGIKGGFVHQRMASKIGRWYSRTMSKTERMGMGLYVKYDGDPWADSDALAFSGVMVSMKEPGWYSFGFEIKDKKCDVPCIGIEMVHDGGKETWHSAATAIYCLMGSGQLLWDTVTGVDMAL |
| B/New York/1116/2007 | 2007 | LPSTIQTLTLFLTSGGVLLSLYVSASLSYLLYSDILLKFSQTEITAPIMPLDCANASNVQAVNRSAAKGVTLLLPEPEWTYPRLSCPGSTFQKALLISPHRFGETKGNSAPLIIREPFIACGPTECKHFALTHYAAQPGGYYNGTREDRNKLRHLISVKLGKIPTVENSIFHMAAWSGSACHDGKEWTYIGVDGPDSNALLKIKYGEAYTDTYHSYAKNILRTQESACNCIGGDCYLMITDGPASGISECRFLKIREGRIIKEIFPTGRVKHTEECTCGFASNKTIECACRDNSYTAKRPFVKLNVETDTAEIRLMCTETYLDTPRPNDGSITGPCESDGDKGSGGIKGGFVHQRMASKIGRWYSRTMSKTKRMGMGLYVKYDGDPWTDSEALALSGVMVSMEEPGWYSFGFEIKDKKCDVPCIGIEMVHDGGKTTWHSAATAIYCLMGSGQLLWDTVTGVDMAL |
| B/New York/1118/2007 | 2007 | LPSTIQTLTLFLTSGGVLLSLYVSASLSYLLYSDILLKFSPTEITASTMPLDCANASNVQAVNRSATKGVTLLLPEPEWTYPRLSCPGSTFQKALLISPHRFGETKGNSAPLIIREPFIACGPNECKHFALTHYAAQPGGYYNGTRGDRNKLRHLISVKLGKIPTVENSIFHMAAWSGSACHDGKEWTYIGVDGPDNDALLKIKYGEAYTDTYHSYANKILRTQESACNCIGGNCYLMITDGSASGVSECRFLKIREGRIIKEIFPTGRVKHTEECTCGFASNKTIECACRDNSYTAKRPFVKLNVETDTAEIRLMCTDTYLDTPRPDDGSITGPCESNGDKGSGGIKGGFVHQRMTSKIGRWYSRTMSKTERMGMGLYVKYDGDPWADSDALAFSGVMVSMKEPGWYSFGFEIKDKKCDVPCIGIEMVHDGGKETWHSAATAIYCLMGSGQLLWDTVTGVDMAL |
| B/New York/1133/2007 | 2007 | LPSTIQTLTLFLTSGGVLLSLYVSASLSYLLYSDILLKFSQTEITAPIMPLDCANASNVQAVNRSAAKGVTLLLPEPEWTYPRLSCPGSTFQKALLISPHRFGETKGNSAPLIIREPFIACGPKECKHFALTHYAAQPGGYYNGTREDRNKLRHLISVKLGKIPTVENSIFHMAAWSGSACHDGKEWTYVGVDGPDSNALLKIKYGEAYTDTYHSYAKNILRTQESACNCIGGDCYLMITDGPASGISECRFLKIREGRIIKEIFPTGRVKHTEECTCGFASNKTIECACRDNSYTAKRPFVKLNVETDTAEIRLMCTETYLDTPRPNDGSITGPCESDGDKGSGGIKGGFVHQRMASKIGRWYSRTMSKTKRMGMGLYVKYDGDPWTDSEALALSGVMVSMEEPGWYSFGFEIKDKKCDVPCIGIEMVHDGGKTTWHSAATAIYCLMGSGQLLWDTVTGVDMAL |
| B/Ohio/UR06-0428/2007 | 2007 | LPSTIQTLTLFLTSGGVLLSLYVSASLSYLLYSDILLKFSPTEITAPTMPLDCANASNVQAVNRSATKGVTLLLPEPEWTYPRLSCPGSTFQKALLISPHRFGETKGNSAPLIIREPFIACGPNECKHFALTHYAAQPGGYYNGTRGDRNKLRHLISIKLGKIPTVENSIFHMAAWSGSACHDGKEWTYIGVDGPDNNALLKIKYGEAYTDTYHSYANKILRTQESACNCIGGNCYLMITDGSASGVSECRFLKIREGRIIKEIFPTGRVKHTEECTCGFASNKTIECACRDNSYTAKRPFVKLNVETDTAEIRLMCTDTYLDTPRPDDGSITGPCESNGDKGSGGIKGGFVHQRMASKIGRWYSRTMSKTERMGMGLYVKYDGDPWADSDALAFSGVMISMKEPGWYSFGFEIKDKKCDVPCIGIEMVHDGGKETWHSAATAIYCLMGSGQLLWDTVTGVDMAL |
| B/Oregon/01/2007 | 2007 | LPSTIQTLTLFLTSGGVLLSLYVSASLSYLLYSDILLKFSPTEITAPTMPLDCANASNVQAVNHSATKGVTLLLPEPEWTYPRLSCPGSTFQKALLISPHRFGETKGNSAPLIIREPFIACGPNECKHFALTHYAAQPGGYYNGTRGDRNKLRHLISVKLGKIPTVENSIFHMAAWSGSACHDGKEWTYIGVDGPDNNALLKIKYGEAYTDTYHSYANKILRTQESACNCIGGNCYLMITDGSASGVSECRFLKIREGRIIKEIFPTGRVKHTEECTCGFASNKTIECACRDNSYTAKRPFVKLNVETDTAEIRLMCTDTYLDTPRPDDGSITGPCESNGDKGSGGIKGGFVHQRMASKIGRWYSRTMSKTERMGMGLYVKYDGDPWADSDALAFSGVMVSMKEPGWYSFGFEIKDKKCDVPCIGIEMVHDGGKETWHSAATAIYCLMGSGQLLWDTVTGVDMAL |
| B/Sydney/201/2007 | 2007 | LPSTIQTLTLFLTSGGVLLSLYVSASLSYLLYSDILLKFSQTEITAPIMPLDCANASNVQAVNRSAAKGVTLLLPEPEWTYPRLSCPGSTFQKALLISPHRFGETKGNSAPLIIREPFIACGPTECKHFALTHYAAQPGGYYNGTREDRNKLRHLISVKLGKIPTVENSIFHMAAWSGSACHDGKEWTYIGVDGPDSNALLKIKYGEAYTDTYHSYAKNILRTQESACNCIGGDCYLMITDGPASGVSECRFLKIREGRIIKEIFPTGRVKHTEECTCGFASNKTIECACRDNSYTAKRPFVKLNVETDTAEIRLMCTETYLDTPRPNDGSITGPCESDGDKGSGGIKGGFVHQRMASKIGRWYSRTMSKTKRMGMGLYVKYDGDPWTDSEALALSGVMVSMEEPGWYSFGFEIKDKKCDVPCIGIEMVHDGGKTTWHSAATAIYCLMGSGQLLWDTVTGVDMAL |
| B/Sydney/503/2007 | 2007 | LPSTIQTLTLFLTSGGVLLSLYVSASLSYLLYSDILLKFSQTEITAPIMPLDCANASNVQAVNRSAAKGVTLLLPEPEWTYPRLSCPGSTFQKALLISPHRFGETKGNSAPLIIREPFIACGPTECKHFALTHYAAQPGGYYNGTREDRNKLRHLISVKLGKIPTVENSIFHMAAWSGSACHDGKEWTYIGVDGPDSNALLKIKYGEAYTDTYHSYAKNILRTQESACNCIGGDCYLMITDGPASGVSECRFLKIREGRIIKEIFPTGRVKHTEECTCGFASNKTIECACRDNSYTAKRPFVKLNVETDTAEIRLMCTETYLDTPRPNDGSITGPCESDGDKGSGGIKGGFVHQRMASKIGRWYSRTMSKTKRMGMGLYVKYDGDPWTDSEALALSGVMVSMEEPGWYSFGFEIKDKKCDVPCIGIEMVHDGGKTTWHSAATAIYCLMGSGQLLWDTVTGVDMAL |
| B/Taiwan/45/2007 | 2007 | LPSTIQTLTLFLTSGGVLLSLYVSASLSYLLYSDILLKFSPTEITAPTMPLDCANASNVQAVNRSATKGVTLLLPEPEWTYPRLSCPGSTFQKALLISPHRFGETKGNSAPLIIREPFIACGPNECKHFALTHYAAQPGGYYNGTRGDRNKLRHLISVKLGKIPTVENSIFHMAAWSGSACHDGKEWTYIGVDGPDNNALLKIKYGEAYTDTYHSYANKILRTQESACNCIGGNCYLMITDGSASGVSECRFLKIREGRIIKEIFPTGRVKHTEECTCGFASNKTIECACRDNSYTAKRPFVKLNVETDTAEIRLMCTDTYLDTPRPDDGSITGPCESNGDRGSGGIKGGFVHQRMASKIGRWYSRTMSKTERMGMGLYVKYDGDPWADSDALAFSGVMVSMKEPGWYSFGFEIKDKKCDVPCIGIEMVHDGGKETWHSAATAIYCLMGSGQLLWDTVTGVDMAL |
| B/Taiwan/70546/2007 | 2007 | LSSTIQTLTLFLTSGGVLLSLYVSASLSYLLYSDILLKFSPTEITAPTMPLDCANASNVQAVNRSATKGVTLLLPEPEWTYPRLSCPGSTFQKALLISPHRFGETKGNSAPLIIREPFIACGPNECKHFALTHYAAQPGGYYNGTRGDRNKLRHLISVKLGKIPTVENSIFHMAAWSGSACHDGKEWTYIGVDGPDNNALLKIKYGEAYTDTYHSYANKILRTQESACNCIGGNCYLMITDGSASGVSECRFLKIREGRIIKEIFPTGRVKHTEECTCGFASNKTIECACRDNSYTAKRPFVKLNVETDTAEIRLMCTDTYLDTPRPDDGSITGPCESNGDRGSGGIKGGFVHQRMASKIGRWYSRTMSKTERMGMGLYVKYDGDPWADSDALAFSGVMVSMKEPGWYSFGFEIKDKKCDVPCIGIEMVHDGGKETWHSAATAIYCLMGSGQLLWDTVTGVDMAL |
| B/Taiwan/99/2007 | 2007 | LPSTIQTLTLFLTSGGVLLSLYVSASLSYLLYSDILLKFSPTEITAPTMPLDCANASNVQAVNRSATKGVTLLLPEPEWTYPRLSCPGSTFQKALLISPHRFGETKGNSAPLIIREPFIACGPNECKHFALTHYAAQPGGYYNGTRGDRNKLRHLISVKLGKIPTVENSIFHMAAWSGSACHDGKEWTYIGVDGPDNNALLKIKYGEAYTDTYHSYANKILRTQESACNCIGGNCYLMITDGSASGVSECRFLKIREGRIIKEIFPTGRVKHTEECTCGFASNKTIECACRDNSYTAKRPFVKLNVETDTAEIRLMCTDTYLDTPRPDDGSITGPCESNGDRGSGGIKGGFVHQRMASKIGRWYSRTMSKTERMGMGLYVKYDGDPWADSDALAFSGVMVSMKEPGWYSFGFEIKDKKCDVPCIGIEMVHDGGKETWHSAATAIYCLMGSGQLLWDTVTGVDMAL |
| B/Tennessee/UR06-0105/2007 | 2007 | LPSTIQTLTLFLTSGGVLLSLYVSASLSYLLYSDILLKFSPTEITASTMPLDCANASNVQAVNRSATKGVTLLLPEPEWTYPRLSCPGSTFQKALLISPHRFGETKGNSAPLIIREPFIACGPNECKHFALTHYAAQPGGYYNGTRGDRNKLRHLISVKLGKIPTVENSIFHMAAWSGSACHDGKEWTYIGVDGPDNNALLKIKYGEAYTDTYHSYANKILRTQESACNCIGGNCYLMITDGSASGVSECRFLKIREGRIIKEIFPTGRVKHTEECTCGFASNKTIECACRDNSYTAKRPFVKLNVETDTAEIRLMCTDTYLDTPRPDDGSITGPCESNGDKGSGGIKGGFVHQRMASKIGRWYSRTMSKTERMGMGLYVKYDGDPWADSDALAFSGVMVSMKEPGWYSFGFEIKDKKCDVPCIGIEMVHDGGKETWHSAATAIYCLMGSGQLLWDTVTGVDMAL |
| B/Tennessee/UR06-0431/2007 | 2007 | LPSTIQTLTLFLTSGGVLLSLYVSASLSYLLYSDILLKFSPTEITAPTMPLDCANASNVQAVNRSATKGVTLLLPEPEWTYPRLSCPGSTFQKALLISPHRFGETKGNSAPLIIREPFIACGPNECKHFALTHYAAQPGGYYNGTRGDRNKLRHLISVKLGKIPTVENSIFHMAAWSGSACHDGKEWTYIGVDGPDNNALLKIKYGEAYTDTYHSYANKILRTQESACNCIGGNCYLMITDGSASGVSECRFLKIREGRIIKEIFPTGRVKHTEECTCGFASNKTIECACRDNSYTAKRPFVKLNVETDTAEIRLMCTDTYLDTPRPDDGSITGPCESNGDKGSGGIKGGFVHQRMASKIGRWYSRTMSKTERMGMGLYVKYDGDPWADSDALAFSGVMVSMKEPGWYSFGFEIKDKKCDVPCIGIEMVHDGGKETWHSAATAIYCLMGSGQLLWDTVTGVDMAL |
| B/Ulaanbaatar/1803/2007 | 2007 | LPSTIQTLTLFLTSGGVLLSLYVSASLSYLLYSDILLKFSPTEITAPTMPLDCANTSNVQAVNRSATKGVTLLLPEPEWTYPRLSCPGSTFQKALLISPHRFGETKGNSAPLIIREPFIACGPNECKHFALTHYAAQPGGYYNGTRGDRNKLRHLISVKLGKIPTVENSIFHMAAWSGSACHDGKEWTYIGVDGPDNNALLKIKYGEAYTDTYHSYANKILRTQESACNCIGGDCYLMITDGPASGVSECRFLKIREGRIIKEIFPTGRVKHTEECTCGFASNKTIECACRDNSYTAKRPFVKLNVETDTAEIRLMCTDTYLDTPRPDDGSITGPCESNGDKGSGGIKGGFVHQRMESKIGRWYSRTMSKTERMGMGLYVKYDGDPWADSDALAFSGVMVSMKEPGWYSFGFEIKDKKCDVPCIGIEMVHDGGKETWHSAATAIYCLMGSGQLLWDTVTGVDMAL |
| B/Ulaanbaatar/7215/2007 | 2007 | LPSTIQTLTLFLTSGGVLLSLYVSASLSYLLYSDILLKFSRTEITAPIMPLDCANASNVQAVNRSATKGVTLLLPEPEWTYPRLSCPGSTFQKALLISPHRFGETKGNSAPLIIREPFIACGPKECKHFALTHYAAQPGGYYNGTREDRNKLRHLISVKLGKIPTVENSIFHMAAWSGSACHDGREWTYIGVDGPDSNALLKIKYGEAYTDTYHSYAKNILRTQESACNCIGGDCYLMITDGPASGISECRFLKIREGRIIKEIFPTGRVKHTEECTCGFASNKTIECACRDNSYTAKRPFVKLNVETDTAEIRLMCTETYLDTPRPNDGSITGPCESNGDKGSGGIKGGFVHQRMASKIGRWYSRTMSKTKRMGMGLYVKYDGDPWTDSEALALSGVMVSMEEPGWYSFGFEIKDKKCDVPCIGIEMVHDGGKTTWHSAATAIYCLMGSGQLLWDTVTGVNMTL |
| B/Victoria/215/2007 | 2007 | LPSTIQTLTLFLTSGGVLLSLYVSASLSYLLYSDILLKFSQTEITAPIMPLDCANASNAQAVNRSAAKGVTLLLPEPEWTYPRLSCPGSTFQKALLISPHRFGETKGNSAPLIIREPFIACGPTECKHFALTHYAAQPGGYYNGTREDRNKLRHLISVKLGKIPTVENSIFHMAAWSGSACHDGKEWTYIGVDGPDSNALLKIKYGEAYTDTYHSYAKNILRTQESACNCIGGDCYLMITDGPASGVSECRFLKIREGRIIKEIFPTGRVKHTEECTCGFASNKTIECACRDNSYTAKRPFVKLNVETDTAEIRLMCTETYLDTPRPNDGSITGPCESDGDKGSGGIKGGFVHQRMASKIGRWYSRTMSKTKRMGMGLYVKYDGDPWTDSEALALSGVMVSMEEPGWYSFGFEIKDKKCDVPCIGIEMVHDGGKTTWHSAATAIYCLMGSGQLLWDTVTGVDMAL |
| B/Victoria/217/2007 | 2007 | LPSTIQTLTLFLTSGGVLLSLYVSASLSYLLYSDILLKFSPTEIIAPTMPLDCANASNVHAVNRSATKGVTLLLPEPEWTYPRLSCPGSTFQKALLISPHRFGETKGNSAPLIIREPFIACGPNECKHFALTHYAAQPGGYYNGTRGDRNKLRHLISVKLGKIPTVENSIFHMAAWSGSACHDGKEWTYIGVDGPDNNALLKIKYGEAYTDTYHSYANNILRTQESACNCIGGNCYLMITDGSASGVSECRFLKIREGRIIKEIFPTGRVKHTEECTCGFASNKTIECACRDNSYTAKRPFVKLNVETDTAEIRLMCTDTYLDTPRPDDGSITGPCESNGDKGSGGIKGGFVHQRMASKIGRWYSRTMSKTERMGMGLYVKYDGDPWADSDALAFSGVMVSMKEPGWYSFGFEIKDKKCDVPCIGIEMVHDGGKETWHSAATAIYCLMGSGQLLWDTVTGVDMAL |
| B/Victoria/505/2007 | 2007 | LPSTIQTLTLLLTSGGVLLSLYVSASLSYLLYSDILLKFSQTEITAPIMPLDCANASNVQAVNRSAAKGVTLLLPEPEWTYPRLSCPGSTFQKALLISPHRFGETKGNSAPLIIREPFIACGPTECKHFALTHYAAQPGGYYNGTREDRNKLRHLISVKLGKIPTVENSIFHMAAWSGSACHDGKEWTYIGVDGPDSNALLKIKYGEAYTDTYHSYAKNILRTQESACNCIGGDCYLMITDGPASGVSECRFLKIREGRIIKEIFPTGRVKHTEECTCGFASNKTIECACRDNSYTAKRPFVKLNVETDTAEIRLMCTETYLDTPRPNDGSITGPCESDGDKGSGGIKGGFVHQRMASKIGRWYSRTMSKTKRMGMGLYVKYDGDPWTDSEALALSGVMVSMEEPGWYSFGFEIKDKKCDVPCIGIEMVHDGGKTTWHSAATAIYCLMGSGQLLWDTVTGVDMAL |
| B/Victoria/508/2007 | 2007 | LPSTIQTLTLFLTSGGVLLSLYVSASLSYLLYSDILLKFSQTEITAQIMPLDCANASNVQAVNRSAAKGVTLLLPEPEWTYPRLSCPGSTFQKALLISPHRFGETKGNSAPLIIREPFIACGPTECKHFALTHYAAQPGGYYNGTREDRNKLRHLISVKLGKIPTVENSIFHMAAWSGSACHDGKEWTYIGVDGPDSNALLKIKYGEAYTDTYHSYAKNILRTQESACNCIGGDCYLMITDGPASGISECRFLKIREGRIIKEIFPTGRVKHTEECTCGFASNKTIECACRDNRYTAKRPFVKLNVETDKAEIRLMCTETYLDTPRPNDGSITGPCESDGDKGSGGIKGGFVHQRMASKIGRWYSRTMSKTKRMGMGLYVKYDGDPWTDSGALALSGVMVSIEEPGWYSFGFEIKDKKCDVPCIGIEMVHDGGKTTWHSAATAIYCLMGSGQLLWDTVTGVDMAL |
| B/Victoria/805/2007 | 2007 | LPSTIQTLTLFLTSGGVLLSLYVSASLSYLLYSDILLKFSQTEITAPIMPLDCANASNVQAVNRSAAKGVTLLLPEPEWTYPRLSCPGSTFQKALLISPHRFGETKGNSAPLIIREPFIACGPTECKHFALTHYAAQPGGYYNGTREDRNKLRHLISVKLGKIPTVENSIFHMAAWSGSACHDGKEWTYIGVDGPDSNALLKIKYGEAYTDTYHSYAKNILRTQESACNCIGGDCYLMITDGPASGVSECRFLKIREGRIIKEIFPTGRVKHTEECTCGFASNKTIECACRDNSYTAKRPFVKLNVETDTAEIRLMCTETYLDTPRPNDGSITGPCESDGDKGSGGIKGGFVHQRMASKIGRWYSRTMSKTKRMGMGLYVKYDGDPWTDSEALALSGVMVSMEEPGWYSFGFEIKDKKCDVPCIGIEMVHDGGKTTWHSAATAIYCLMGSGQLLWDTVTGVDMAL |
| B/Vienna/23/2007 | 2007 | LPSTIQTLTLFLTSGGVLLSLYVSASLSYLLYSDILLKFSRTEITAPIMPLDCANASNVQAVNRSATKGVTLLLPEPEWTYPRLSCPGSTFQKALLISPHRFGETKGNSAPLIIREPFIACGPKECKHFALTHYAAQPGGYYNGTREDRNKLRHLISVKLGKIPTVENSIFHMAAWSGSACHDGREWTYIGVDGPDSNALLKIKYGEAYTDTYHSYAKNILRTQESACNCIGGDCYLMITDGPASGISECRFLKIREGRIIKEIFPTGRVKHTEECTCGFASNKTIECACRDNSYTAKRPFVKLNVETDTAEIRLMCTETYLDTPRPNDGSITGPCESNGDKGSGGIKGGFVHQRMASKIGRWYSRTMSKTERMGMGLYVKYDGDPWADSDALAFSGVMVSMKEPGWYSFGFEIKDKKCDVPCIGIEMVHDGGKETWHSAATAIYCLMGSGQLLWDTVTGVNMAL |
| B/AKITA/2/2008 | 2008 | LPSTIQTLTLFLTSGGVLLSLYVSASLSYLLYSDILLKFSRTEITAPIMPLDCANASNVQAVNRSATKGVTLLLPEPEWTYPRLSCPGSTFQKALLISPHRFGETKGNSAPLIIREPFIACGPKECKHFALTHYAAQPGGYYNGTREDRNKLRHLISVKLGKIPTVENSIFHMAAWSGSACHDGREWTYIGVDGPDSNALLKIKYGEAYTDTYHSYAKNILRTQESACNCIGGDCYLMITDGPASGISECRFLKIREGRIIKEIFPTGRVKHTEECTCGFASNKTIECACRDNSYTAKRPFVKLNVETDTAEIRLMCTETYLDTPRPNDGSITGPCESNGDKGSGGIKGGFVHQRMASKIGRWYSRTMSKTKRMGMGLYVKYDGDPWTDSEALALSGVMVSMEEPGWYSFGFEIKDKKCDVPCIGIEMVHDGGKTTWHSAATAIYCLMGSGQLLWDTVTGVNMTL |
| B/Auckland/23/2008 | 2008 | LPSTIQTLTLFLTSGGVLLSLYVSASLSYLLYSDILLKFSPTEITAPTMPLDCANASNVQAVNRSATKGVTLLLPEPEWTYPRLSCPGSTFQKALLISPHRFGETKGNSAPLIIREPFIACGPNECKHFALTHYAAQPGGYYNGTRGDRNKLRHLISVKLGKIPTVENSIFHMAAWSGSACHDGKEWTYIGVDGPDNNALLKVKYGEAYTDTYHSYANKILRTQESACNCIGGNCYLMITDGSASGVSECRFLKIREGRIIKEIFPTGRVKHTEECTCGFASNKTIECACRDNSYTAKRPFVKLNVETDTAEIRLMCTDTYLDTPRPNDGSITGPCESNGDKGSGGIKGGFVHQRMESKIGRWYSRTMSKTERMGMGLYVKYDGDPWADSDALAFSGVMVSMKEPGWYSFGFEIKDKKCDVPCIGIEMVHDGGKETWHSAATAIYCLMGSGQLLWDTVTGVDMAL |
| B/Auckland/63/2008 | 2008 | LPSTIQTLTLFLTSGGVLLSLYVSASLSYLLYSDILLKFSQTEITAPIMPLDCANASNVQAVNRSAAKGVTLLLPEPEWTYPRLSCPGSTFQKALLISPHRFGETKGNSAPLIIREPFIACGTTECKHFALTHYAAQPGGYYNGTREDRNKLRHLISVKLGKIPTVENSIFHMAAWSGSACHDGKEWTYIGVDGPDSNALLKIKYGEAYTDTYHSYAKNILRTQESACNCIGGDCYLMITDGPASGISECRFLKIREGRIIKEIFPTGRVKHTEECTCGFASNKTIECACRDNRYTAKRPFVKLNVETDTAEIRLMCTETYLDTPRPNDGSITGPCESDGDKGSGGIKGGFVHQRMASKIGRWYSRTMSKTKRMGMGLYVKYDGDPWTDSGALALSGVMVSIEEPGWYSFGFEIKDKKCDVPCIGIEMVHDGGKTTWHSAATTIYCLMGSGQLLWDTVTGVDMAL |
| B/beijingxicheng/178/2008 | 2008 | LPSTIQTLTLFLTSGGVLLSLYVSASLSYLLYSDILLKFPSTEITAPTMPLDCANASNVQAVNRSATKGVTLLLPEPEWTYPRLSCPGSTFQKALLISPHRFGETKGNSAPLIIREPFIACGPKECKHFALTHYAAQPGGYYNGTRGDRNKLRHLISVKLGKIPTVENSIFHMAAWSGSACHDGKEWTYIGVDGPDNNALLKIKYGEAYTDTYHSYANNILRTQESACNCIGGNCYLMITDGSASGVSECRFLKIREGRIIKEIFPTGRIKHTEECTCGFASNKTIECACRDNSYTAKRPFVKLNVETDTAEIRLMCTETYLDTPRPDDGSITGPCESNGGKGSGGIKGGFVHQRMASKIGRWYSRTMSKTERKGMGLYVKYDGDPWADSDALAFSGVMVSMEEPGWYSFGFEIKDKKCDVPCIGIEMVHDGGKETWHSAATAIYCLMGSGQLLWDTVTGVNMAL |
| B/Brisbane/115/2008 | 2008 | LPSTIQTLTLFLTSGGVLLSLYVSASLSYLLYSDILLKFSQTEITAPIMPLDCANASNVQAVNRSAAKGVTLLLPEPEWTYPRLSCPGSTFQKALLISPHRFGETKGNSAPLIIREPFIACGPTECKHFALTHYAAQPGGYYNGTREDRNKLRHLISVKLGKIPTVENSIFHMAAWSGSACHDGKEWTYIGVDGPDSNALLKIKYGEAYTDTYHSYAKNILRTQESACNCIGGDCYLMITDGPASGISECRFLKIREGRIIKEIFPTGRVKHTEECTCGFASNKTIECACRDNSYTAKRPFVKLNVETDTAEIRLMCTKTYLDTPRPNDGSITGPCESDGDKGSGGIKGGFVHQRMASKIGRWYSRTMSKTRRMGMGLYVKYDGDPWTDSEALALSGVMVSMEEPGWYSFGFEIKDKKCDVPCIGIEMVHDGGKTTWHSAATAIYCLMGSGQLLWDTVTGVDMAL |
| B/Brisbane/14/2008 | 2008 | LPSTIQTLTLFLTSGGVLLSLYVSASLSYLLYSDILLKFSRTEITAPTMPLDCANASNVQAVNRSATKGVTLLLPEPEWTYPRLSCPGSTFQKALLISPHRFGETKGNSAPLIIREPFIACGPKECKHFALTHYAAQPGGYYNGTREDRNKLRHLISVKLGKIPTVENSIFHMAAWSGSACHDGREWTYIGVDGPDSNALLKIKYGEAYTDTYHSYAKNILRTQESACNCIGGDCYLMITDGPASGISECRFLKIREGRIIKEIFPTGRVKHTEECTCGFASNKTIECACRDNSYTAKRPFVKLNVETDTAEIRLMCTETYLDTPRPNDGSITGPCESNGDKGSGGIKGGFVHQRMASKIGRWYSRTMSKTKRMGMGLYVKYDGDPWTDSEALALSGVMVSMEEPGWYSFGFEIKDKKCDVPCIGIEMVHDGGKTTWHSAATAIYCLMGSGQLLWDTVTGVNMTL |
| B/Brisbane/168/2008 | 2008 | LPSTIQTLTLFLTSGGVLLSLYVSASLSYLLYSDILLKFSQTEITAPIMPLDCANASNVQAVNRSAAKGVTLLLPEPEWTYPRLSCPGSTFQKALLISPHRFGETRGNSAPLIIREPFIACGPTECKHFALTHYAAQPGGYYNGTREDRNKLRHLISVKLGKIPTVENSIFHMAAWSGSACHDGKEWTYIGVDGPDSNALLKIKYGEAYTDTYHSYAKNILRTQESACNCIGGDCYLMITDGPASGISECRFLKIREGRIIKEIFPTGRVKHTEECTCGFASNKTIECACRDNSYTAKRPFVKLNVETDTAEIRLMCTETYLDTPRPNDGSITGPCESDGDKGSGGIKGGFVHQRMASKIGRWYSRTMSKTKRMGMGLYVKYDGDPWTDSEALALSGVMVSMEEPGWYSFGFEIKDKKCDVPCIGIEMVHDGGKTTWHSAATAIYCLMGSGQLLWDTVTGVDMAL |
| B/Brisbane/34/2008 | 2008 | LPSTIQTLTLFLTSGGVLLSLYVSASLSYLLYSDILLKFSQTEITAPIMPLDYANASNVQAVNHSAAKGVTLLLPEPEWTYPRLSCPGSTFQKALLISPHRFGETKGNSAPLIIREPFIACGPTECKHFALTHYAAQPGGYYNGTREDRNKLRHLISVKLGKIPTVENSIFHMAAWSGSACHDGKEWTYIGVDGPDSNALLKIKYGEAYTDTYHSYAKNILRTQESACNCIGGDCYLMITDGPASGISECRFLKIREGRIIKEIFPTGRVKHTEECTCGFASNKTIECACRDNSYTAKRPFVKLNVETDTAEIRLMCTETYLDTPRPNDGSITGPCESDGDKGSGGIKGGFVHQRMASKIGRWYSRTMSKTKRMGMGLYVKYDGDPWTDSEALALSGVMISMEEPGWYSFGFEIKDKKCDVPCIGIEMVHDGGKTTWHSAATAIYCLMGSGQLLWDTVTGVDMAL |
| B/CHIBA/13/2008 | 2008 | LPSTIQTLTLFLTSGGVLLSLYVSASLSYLLYSDILLKFSPTEITAPTMPLDCANASNVQAVNRSATKGVTLLLPEPEWTYPRLSCPGSTFQKALLISPHRFGETKGNSAPLIIREPFIACGPNECKHFALTHYAAQPGGYYNGTRGDRNKLRHLISVKLGKIPTVENSIFHMAAWSGSACHDGKEWTYIGVDGPDNNALLKVKYGEAYTDTYHSYANKILRTQESACNCIGGNCYLMITDGSASGVSECRFLKIREGRIIKEIFPTGRVKHTEECTCGFASNKTIECACRDNSYTAKRPFVKLNVETDTAEIRLMCTDTYLDTPRPNDGSITGPCESNGDKGSGGIKGGFVHQRMESKIGRWYSRTMSKTERMGMGLYVKYDGDPWADSDALAFSGVMVSMKEPGWYSFGFEIKDKKCDVPCIGIEMVHDGGKETWHSAATAIYCLMGSGQLLWDTVTGVDMAL |
| B/Christchurch/13/2008 | 2008 | LPTTIQTLTLFLTSGGVLLSLYVSASLSYLLYSDILLKFSRTEITAPIMPLDCANASNVQAVNRSATKGVTLLLPEPEWTYPRLSCPGSTFQKALLISPHRFGETKGNSAPLIIREPFIACGPKECKHFALTHYAAQPGGYYNGTREDRNKLRHLISVKLGKIPTVENSIFHMAAWSGSACHDGREWTYIGVDGPDSNALLKIKYGEAYTDTYHSYAKNILRTQESACNCIGGDCYLMITDGPASGISECRFLKIREGRIIKEIFPTGRVKHTEECTCGFASNKTIECACRDNSYTAKRPFVKLNVETDTAEIRLMCTETYLDTPRPNDGSITGPCESNGDKGSGGIKGGFVHQRMASKIGRWYSRTMSKTKRMGMGLYVKYDGDPWTDSEALALSGVMVSMEEPGWYSFGFEIKDKKCDVPCIGIEMVHDGGKTTWHSAATAIYCLMGSGQLLWDTVTGVNMTL |
| B/Christchurch/2/2008 | 2008 | LPSTIQTLTLFLTSGGVLLSLYVSASLSYLLYSDILLKFSQTEITAPIMPLDYANASNVQAVNHSAAKGVTLLLPEPEWTYPRLSCPGSTFQKALLISPHRFGETKGNSAPLIIREPFIACGPTECKHFALTHYAAQPGGYYNGTREDRNKLRHLISVKLGKIPTVENSIFHMAAWSGSACHDGKEWTYIGVDGPDSNALLKIKYGEAYTDTYHSYAKNILRTQESACNCIGGDCYLMITDGPASGISECRFLKIREGRIIKEIFPTGRVKHTEECTCGFASNKTIECACRDNSYTAKRPFVKLNVETDTAEIRLMCTETYLDTPRPNDGSITGPCESDGDKGSGGIKGGFVHQRMASKIGRWYSRTMSKTKRMGMGLYVKYDGDPWTDSEALALSGVMVSMEEPGWYSFGFEIKDKKCDVPCIGIEMVHDGGKTTWHSAATAIYCLMGSGQLLWDTVTGVDMAL |
| B/England/393/2007 | 2008 | LPSTIQTLTLFLTSGGVLLSLYVSASLSYLLYSDILLKFSPTEITAPTMPLDCANASNVQAVNRSATKGVTLLLPEPEWTYPRLSCPGSTFQKALLISPHRFGETKGNSAPLIIREPFIACGPNECKHFALTHYAAQPGGYYNGTRGDRNKLRHLISVKLGKIPTVENSIFHMAAWSGSACHDGKEWTYIGVDGPDNNALLKVKYGEAYTDTYHSYANKILRTQESACNCIGGNCYLMITDGSASGVSECRFLKIREGRIIKEIFPTGRVKHTEECTCGFASNKTIECACRDNSYTAKRPFVKLNVETDTAEIRLMCTDTYLDTPRPNDGSITGPCESNGDKGSGGIKGGFVHQRMESKIGRWYSRTMSKTERMGMGLYVKYDGDPWADSDALAFSGVMVSMKEPGWYSFGFEIKDKKCDVPCIGIEMVHDGGKETWHSAATAIYCLMGSGQLLWDTVTGVDMAL |
| B/Hubei-songzi/322/2008 | 2008 | LPSTIQTLTLFLTSGGVLLSLYVSASLSYLLYSDILLKFPSTEITAPTMPLDCANASNVQAVNRSATKGVTLLLPEPEWTYPRLSCPGSTFQKALLISPHRFGETKGNSAPLIIREPFIACGPKECKHFALTHYAAQPGGYYNGTRGDRNKLRHLISVKLGKIPTVENSIFHMAAWSGSACHDGKEWTYIGVDGPDNNALLKIKYGEAYTDTYHSYANNILRTQESACNCIGGNCYLMITDGSASGVSECRFLKIREGRIIKEIFPTGRIQHTEECTCGFASNKTIECACRDNSYTAKRPFVKLNVETDTAEIRLMCTKTYLDTPRPDDGSITGPCESNGGKGSGGIKGGFVHQRMASKIGRWYSRTMSKTERKGMGLYVKYDGDPWADSDALAFSGVMVSMEEPGWYSFGFEIKDKKCDVPCIGIEMVHDGGKETWHSAATAIYCLMGSGQLLWDTVTGVNMAL |
| B/IBARAKI/18/2008 | 2008 | LPSTIQTLTLFLTSGGVLLSLYVSASLSYLLYSDILLKFSPTEITAPTMPLDCANASNVQAVNRSATKGVTLLLPEPEWTYPRLSCPGSTFQKALLISPHRFGETKGNSAPLIIREPFIACGPNECKHFALTHYAAQPGGYYNGTRGDRNKLRHLISVKLGKIPTVENSIFHMAAWSGSACHDGKEWTYIGVDGPDNNALLKVKYGEAYTDTYHSYANKILRTQESACNCIGGNCYLMITDGSASGVSECRFLKIREGRIIKEIFPTGRVKHTEECTCGFASNKTIECACRDNSYTAKRPFVKLNVETDTAEIRLMCTDTYLDTPRPNDGSITGPCESNGDKGSGGIKGGFVHQRMESKIGRWYSRTMSKTERMGMGLYVKYDGDPWADSDALAFSGVMVSMKEPGWYSFGFEIKDKKCDVPCIGIEMVHDGGKETWHSAATAIYCLMGSGQLLWDTVTGVDMAL |
| B/KANAGAWA/2/2008 | 2008 | LPSTIQTLTLFLTSGGVLLSLYVSASLSYLLYSDILLKFSQTEITAPIMPLDCANASNVQAVNRSAAKGVTLLLPEPEWTYPRLSCPGSTFQKALLISPHRFGETKGNSAPLIIREPFIACGPTECKHFALTHYAAQPGGYYNGTREDRNKLRHLISVKLGKIPTVENSIFHMAAWSGSACHDGKEWTYIGVDGPDSNALLKIKYGEAYTDTYHSYAKNILRTQESACNCIGGDCYLMITDGPASGVSECRFLKIREGRIIKEIFPTGRIKHTEECTCGFASNKTIECACRDNSYTAKRPFVKLNVETDTAEIRLMCTETYLDTPRPNDGSITGPCESDGDKGSGGIKGGFVHQRMASKIGRWYSRTMSKTKRMGMGLYVKYDGDPWTDSEALALSGVMVSMEEPGWYSFGFEIKDKKCDVPCIGIEMVHDGGKTTWHSAATAIYCLMGSGQLLWDTVTGVDMAL |
| B/Managua/3342.01/2008 | 2008 | LPSTIQTLTLFLTSGGVLLSLYVSASLSYLLYSDILLKFSRTEITAPIMPLDCANASNVQAVNRSATKGVTLLLPEPEWTYPRLSCPGSTFQKALLISPHRFGETKGNSAPLIIREPFIACGPKECKHFALTHYAAQPGGYYNGTREDRNKLRHLISVKLGKIPTVENSIFHMAAWSGSACHDGREWTYIGVDGPDSNALLKIKYGEAYTDTYHSYAKNILRTQESACNCIGGDCYLMITDGPASGISECRFLKIREGRIIKEIFPTGRVKHTEECTCGFASNKTIECACRDNSYTAKRPFVKLNVETDTAEIRLMCTETYLDTPRPNDGSITGPCESNGDKGSGGIKGGFVHQRMASKIGRWYSRTMSKTKRMGMGLYVKYDGDPWTDSEALALSGVMVSMEEPGWYSFGFEIKDKKCDVPCIGIEMVHDGGKTTWHSAATAIYCLMGSGQLLWDTVTGVNMTL |
| B/Myanmar/97/2008 | 2008 | LPSTIQTLTLFLTSGGVLLSLYVSASLSYLLYSDILLKFSRTEITAPIMPLDCANASNVQAVNRSATKGVTLLLPEPEWTYPRLSCPGSTFQKALLISPHRFGETKGNSAPLIIREPFIACGPKECKHFALTHYAAQPGGYYNGTREDRNKLRHLISVKLGKIPTVENSIFHMAAWSGSACHDGREWTYIGVDGPDSNALLKIKYGEAYTDTYHSYAKNILRTQESACNCIGGDCYLMITDGPASGISECRFLKIREGRIIKEIFPTGRVKHTEECTCGFASNKTIECACRDNSYTAKRPFVKLNVETDTAEIRLMCTETYLDTPRPNDGSITGPCESNGDKGSGGIKGGFVHQRMASKIGRWYSRTMSKTKRMGMGLYVKYDGDPWTDSEALALSGVMVSMEEPGWYSFGFEIKDKKCDVPCIGIEMVHDGGKTTWHSAATAIYCLMGSGQLLWDTVTGVNMTL |
| B/New York/1134/2008 | 2008 | LPSTIQTLTLFLTSGGVLLSLYVSASLSYLLYSDILLKFSRTEITAPIMPLDCANASNVQAVNRSATKGVKLLLPEPEWTYPRLSCPGSTFQKALLISPHRFGETKGNSAPLIIREPFIACGPKECKHFALTHYAAQPGGYYNGTREDRNKLRHLISVKLGKIPTVENSIFHMAAWSGSACHDGREWTYIGVDGPDSNALLKIKYGEAYTDTYHSYAKNILRTQESACNCIGGDCYLMITDGPASGISECRFLKIREGRIIKEIFPTGRVKHTEECTCGFASNKTIECACRDNSYTAKRPFVKLNVETDTAEIRLMCTETYLDTPRPNDGSITGPCESNGDKGSGGIKGGFVHQRMASKIGRWYSRTMSKTKRMGMGLYVKYDGDPWTDSEALALSGVMVSMEEPGWYSFGFEIKDKKCDVPCIGIEMVHDGGKTTWHSAATAIYCLMGSGQLLWDTVTGVNMTL |
| B/New York/1136/2008 | 2008 | LPSTIQTLTLFLTSGGVLLSLYVSASLSYLLYSDILLKFSRTEITAPIMPLDCANASNVQAVNRSATKGVTLLLPEPEWTYPRLSCPGSTFQKALLISPHRFGETKGNSAPLIIREPFIACGPKECKHFALTHYAAQPGGYYNGTREDRNKLRHLISVKLGKIPTVENSIFHMAAWSGSACHDGREWTYIGVDGPDSNALLKIKYGEAYTDTYHSYAKNILRTQESACNCIGGDCYLMITDGPASGISECRFLKIREGRIIKEIFPTGRVKHTEECTCGFASNKTIECACRDNSYTAKRPFVKLNVETDTAEIRLMCTETYLDTPRPNDGSITGPCESNGDKGSGGIKGGFVHQRMASKIGRWYSRTMSKTKRMGMGLYVKYDGDPWTDSEALALSGVMVSMEEPGWYSFGFEIKDKKCDVPCIGIEMVHDGGKTTWHSAATAIYCLMGSGQLLWDTVTGVNMTL |
| B/New York/1138/2008 | 2008 | LPSTIQTLTLFLTSGGVLLSLYVSASLSYLLYSDILLKFSRTEITAPIMPLDCANASNVQAVNRSATKGVTLLLPEPEWTYPRLSCPGSTFQKALLISPHRFGETKGNSAPLIIREPFIACGPKECKHFALTHYAAQPGGYYNGTREDRNKLRHLISVKLGKIPTVENSIFHMAAWSGSACHDGREWTYIGVDGPDSNALLKIKYGEAYTDTYHSYAKNILRTQESACNCIGGDCYLMITDGPASGISECRFLKIREGRIIKEIFPTGRVKHTEECTCGFASNKTIECACRDNSYTAKRPFVKLNVETDTAEIRLMCTETYLDTPRPNDGSITGPCESNGDKGSGGIKGGFVHQRMASKIGRWYSRTMSKTKRMGMGLYVKYDGDPWTDSEALALSGVMVSMEEPGWYSFGFEIKDKKCDVPCIGIEMVHDGGKTTWHSAATAIYCLMGSGQLLWDTVTGVNMTL |
| B/New York/1151/2008 | 2008 | LPSTIQTLTLFLTSGGVLLSLYVSASLSYLLYSDILLKFSRTEITAPIMPLDCANASNVQAVNRSATKGVTLLLPEPEWTYPRLSCPGSTFQKALLISPHRFGETKGNSAPLIIREPFIACGPKECKHFALTHYAAQPGGYYNGTREDRNKLRHLISVKLGKIPTVENSIFHMAAWSGSACHDGREWTYIGVDGPDSNALLKIKYGEAYTDTYHSYAKNILRTQESACNCIGGDCYLMITDGPASGISECRFLKIREGRIIKEIFPTGRVKHTEECTCGFASNKTIECACRDNSYTAKRPFVKLNVETDTAEIRLMCTETYLDTPRPNDGSITGPCESNGDKGSGGIKGGFVHQRMASKIGRWYSRTMSKTKRMGMGLYVKYDGDPWTDSEALALSGVMVSMEEPGWYSFGFEIKDKKCDVPCIGIEMVHDGGKTTWHSAATAIYCLMGSGQLLWDTVTGVNMTL |
| B/New York/1154/2008 | 2008 | LPSTIQTLTLFLTSGGVLLSLYVSASLSYLLYSDILLKFSQTEITAPIMPLDCANASNVQAVNRSAAKGVTLLLPEPEWTYPRLSCPGSTFQKALLISPHRFGETKGNSAPLIIREPFIACGPTECKHFALTHYAAQPGGYYNGTREDRNKLRHLISVKLGKIPTVENSIFHMAAWSGSACHDGKEWTYIGVDGPDSNALLKIKYGEAYTDTYHSYAKNILRTQESACNCIGGDCYLMITDGPASGISECRFLKIREGRIIKEIFPTGRVKHTEECTCGFASNKTIECACRDNSYTAKRPFVKLNVETDTAEIRLMCTETYLDTPRPNDGSITGPCESDGDKGSGGIKGGFVHQRMASKIGRWYSRTMSKTKRMGMGLYVKYDGDPWTDSEALALSGVMVSMEEPGWYSFGFEIKDKKCDVPCIGIEMVHDGGKTTWHSAATAIYCLMGSGQLLWDTVTGVDMAL |
| B/New York/1158/2008 | 2008 | LPSTIQTLTLFLTSGGVLLSLYVSASLSYLLYSDILLKFSRTEITAPIMPLDCANASNVQAVNRSATKGVTFLLPEPEWTYPRLSCPGSTFQKALLISPHRFGETKGNSAPLIIREPFIACGPKECKHFALTHYAAQPGGYYNGTREDRNKLRHLISVKLGKIPIVENSIFHMAAWSGSACHDGREWTYIGVDGPDSNALLKIKYGEAYTDTYHSYAKNILRTQESACNCIGGDCYLMITDGPASGISECRFLKIREGRIIKEIFPTGRVKHTEECTCGFASNKTIECACRDNSYTAKRPFVKLNVETDTAEIRLMCTETYLDTPRPNDGSITGPCESNGDKGSGGIKGGFVHQRMASKIGRWYSRTMSKTKRMGMGLYVKYDGDPWTDSEALALSGVMVSMEEPGWYSFGFEIKDKKCDVPCIGIEMVHDGGKTTWHSAATAIYCLMGSGQLLWDTVTGVNMTL |
| B/New York/1160/2008 | 2008 | LPSTIQTLTLFLTSGGVLLSLYVSASLSYLLYSDILLKFSQTEITAPIMPLDCANASNVQAVNRSAAKGVTLLLPEPEWTYPRLSCPGSTFQKALLISPHRFGETKGNSAPLIIREPFIACGPTECKHFALTHYAAQPGGYYNGTREDRNKLRHLISVKLGKIPTVENSIFHMAAWSGSACHDGKEWTYIGVDGPDSNALLKIKYGEAYTDTYHSYAKNILRTQESACNCIGGDCYLMITDGPASGISECRFLKIREGRIIKEIFPTGRVKHTEECTCGFASNKTIECACRDNSYTAKRPFVKLNVETDTAEIRLMCTETYLDTPRPNDGSITGPCESDGDKGSGGIKGGFVHQRMASKIGRWYSRTMSKTKRMGMGLYVKYDGDPWTDSEALALSGVMVSMEEPGWYSFGFEIKDKKCDVPCIGIEMVHDGGKTTWHSAATAIYCLMGSGQLLWDTVTGVDMAL |
| B/New York/1162/2008 | 2008 | LPSTIQTLTLFLTSGGVLLSLYVSASLSYLLYSDILLKFSRTEITAPIMPLDCANASNVQAVNRSATKGVTLLLPEPEWTYPRLSCPGSTFQKALLISPHRFGETKGNSAPLIIREPFIACGPKECKHFALTHYAAQPGGYYNGTREDRNKLRHLISVKLGKIPTVENSIFHMAAWSGSACHDGREWTYIGVDGPDSNALLKIKYGEAYTDTYHSYAKNILRTQESACNCIGGDCYLMITDGPASGISECRFLKIREGRIIKEIFPTGRVKHTEECTCGFASNKTIECACRDNSYTAKRPFVKLNVETDTAEIRLMCTETYLDTPRPNDGSITGPCESNGDKGSGGIKGGFVHQRMASKIGRWYSRTMSKTKRMGMGLYVKYDGDPWTDSEALALSGVMVSMEEPGWYSFGFEIKDKKCDVPCIGIEMVHDGGKTTWHSAATAIYCLMGSGQLLWDTVTGVNMTL |
| B/New York/1174/2008 | 2008 | LPSTIQTLTLFLTSGGVLLSLYVSASLSYLLYSDILLKFSRTEITAPIMPLDCANASNVQAVNRSAIKGVTLLLPEPEWTYPRLSCPGSTFQKALLISPHRFGETKGNSAPLIIREPFIACGPKECKHFALTHYAAQPGGYYNGTREDRNKLRHLISVKLGKIPTVENSIFHMAAWSGSACHDGREWTYIGVDGPDSNALLKIKYGEAYTDTYHSYAKNILRTQESACNCIGGDCYLMITDGPASGISECRFLKIREGRIIKEIFPTGRVKHTEECTCGFASNKTIECACRDNSYTAKRPFVKLNVETDTAEIRLMCTETYLDTPRPNDGSITGPCESNGDKGSGGIKGGFVHQRMASKIGRWYSRTMSKTKRMGMGLYVKYDGDPWTDSEALALSGVMVSMEEPGWYSFGFEIKDKKCDVPCIGIEMVHDGGKTTWHSAATAIYCLMGSGQLLWDTVTGVNMTL |
| B/New York/1183/2008 | 2008 | LPSTIQTLTLFLTSGGVLLSLYVSASLSYLLYSDILLKFSRTEITAPIMPLDCANASNVQAVNRSATKGVTLLLPEPEWTYPRLSCPGSTFQKALLISPHRFGETKGNSAPLIIREPFIACGPKECKHFALTHYAAQPGGYYNGTREDRNKLRHLISVKLGKIPTVENSIFHMAAWSGSACHDGREWTYIGVDGPDSNALLKIKYGEAYTDTYHSYAKNILRTQESACNCIGGDCYLMITDGPASGISECRFLKIREGRIIKEIFPTGRVKHTEECTCGFASNKTIECACRDNSYTAKRPFVKLNVETDTAEIRLMCTETYLDTPRPNDGSITGPCESNGDKGSGGIKGGFVHQRMASKIGRWYSRTMSKTKRMGMGLYVKYDGDPWTDSEALALSGVMVSMEEPGWYSFGFEIKDKKCDVPCIGIEMVHDGGKTTWHSAATAIYCLMGSGQLLWDTVTGVNMTL |
| B/New York/1188/2008 | 2008 | LPSTIQTLTLFLTSGGVLLSLYVSASLSYLLYSDILLKFSRTEITAPIMPLDCANASNVQAVNRSATKGVTLLLPEPEWTYPRLSCPGSTFQKALLISPHRFGETKGNSAPLIIREPFIACGPKECKHFALTHYAAQPGGYYNGTREDRNKLRHLISVKLGKIPTVENSIFHMAAWSGSACHDGREWTYIGVDGPDSNALLKIKYGEAYTDTYHSYAKNILRTQESACNCIGGDCYLMITDGPASGISECRFLKIREGRIIKEIFPTGRVKHTEECTCGFASNKTIECACRDNSYTAKRPFVKLNVETDTAEIRLMCTETYLDTPRPNDGSITGPCESNGDKGSGGIKGGFVHQRMASKIGRWYSRTMSKTKRMGMGLYVKYDGDPWTDSEALALSGVMVSMEEPGWYSFGFEIKDKKCDVPCIGIEMVHDGGKTTWHSAATAIYCLMGSGQLLWDTVTGVNMTL |
| B/New York/1190/2008 | 2008 | LPSTIQTLTLFLTSGGVLLSLYVSASLSYLLYSDILLKFSRTEITAPIMPLDCANASNVQAVNRSATKGVTLLLPEPEWTYPRLSCPGSTFQKALLISPHRFGETKGNSAPLIIREPFIACGPKECKHFALTHYAAQPGGYYNGTREDRNKLRHLISVKLGKIPTVENSIFHMAAWSGSACHDGREWTYIGVDGPDSNALLKIKYGEAYTDTYHSYAKNILRTQESACNCIGGDCYLMITDGPASGISECRFLKIREGRIIKEIFPTGRVKHTEECTCGFASNKTIECACRDNSYTAKRPFVKLNVETDTAEIRLMCTETYLDTPRPNDGSITGPCESNGDKGSGGIKGGFVHQRMASKIGRWYSRTMSKTKRMGMGLYVKYDGDPWTDSEALALSGVMVSMEEPGWYSFGFEIKDKKCDVPCIGIEMVHDGGKTTWHSAATAIYCLMGSGQLLWDTVTGVNMTL |
| B/Perth/6/2008 | 2008 | LPSTIQTLTLFLTSGGVLLSLYVSASLSYLLYSDILLKFSQTEITAPIMPLDCANASNVQAVNRSAAKGVTLLLPEPEWTYPRLSCPGSTFQKALLISPHRFGETKGNSAPLIIREPFIACGPTECKHFALTHYAAQPGGYYNGTREDRNKLRHLISVKLGKIPTVENSIFHMAAWSGSACHDGKEWTYIGVDGPDSNALLKIKYGEAYTDTYHSYAKNILRTQESACNCIGGDCYLMITDGPASGISECRFLKIREGRIIKEIFPTGRVKHTEECTCGFASNKTIECACRDNSYTAKRPFVKLNVETDTAEIRLMCTETYLDTPRPNDGSITGPCESDGDKGSGGIKGGFVHQRMASKIGRWYSRTMSKTKRMGMGLYVKYDGDPWTDSEALALSGVMVSMEEPGWYSFGFEIKDKKCDVPCIGIEMVHDGGKTTWHSAATAIYCLMGSGQLLWDTVTGVDMAL |
| B/SAKAI/43/2008 | 2008 | LPSTIQTLTLFLTSGGVLLSLYVSASLSYLLYSDILLKFSPTEITAPTMPLDCANASNVQAVNRSATKGVTLLLPEPEWTYPRLSCPGSTFQKALLISPHRFGETKGNSAPLIIREPFIACGPNECKHFALTHYAAQPGGYYNGTRGDRNKLRHLISVKLGKIPTVENSIFHMAAWSGSACHDGKEWTYIGVDGPDNNALLKVKYGEAYTDTYHSYANKILRTQESACNCIGGNCYLMITDGSASGVSECRFLKIREGRIIKEIFPTGRVKHTEECTCGFASNKTIECACRDNSYTAKRPFVKLNVETDTAEIRLMCTDTYLDTPRPNDGSITGPCESNGDKGSGGIKGGFVHQRMESKIGRWYSRTMSKTERMGMGLYVKYDGDPWADSDALAFSGVMVSMKEPGWYSFGFEIKDKKCDVPCIGIEMVHDGGKETWHSAATAIYCLMGSGQLLWDTVTGVDMAL |
| B/SHIGA/6/2008 | 2008 | LPSTTQTLTLFLTSGGVLLSLYVSASLSYLLYSDILLKFPSTEITAPTMPLDCANASNVQAVNRSATKGVTLLLPEPEWTYPRLSCPGSTFQKALLISPHRFGETKGNSAPLIIREPFIACGPKECKHFALTHYAAQPGGYYNGTRGDRNKLRHLISVKLGKIPTVENSIFHMAAWSGSACHDGKEWTYIGVDGPDNNALLKIKYGEAYTDTYHSYANNILRTQESACNCIGGNCYLMITDGSASGVSECRFLKIREGRIIKEIFPTGRIQHTEECTCGFASNKTIECACRDNSYTAKRPFVKLNVETDTAEIRLMCTETYLDTPRPDDGSITGPCESNGGKGSGGIKGGFVHQRMASKIGRWYSRTMSKTERKGMGLYVKYDGDPWADSDALAFSGVMVSMEEPGWYSFGFEIKDKKCDVPCIGIEMVHDGGKETWHSAATAIYCLMGSGQLLWDTVTGVNMAL |
| B/Singapore/616/2008 | 2008 | LPSTIQMLTLFLTLGGVLLSLYVSASLSYLLYSDILLKFPPTEITAPTMPLDCANASNVQAVNRSATKGVTLLLPEPEWTYPRLSCPGSTFQKALLISPHRFGETKGNSAPLIIREPFIACGPKECKHFALTHYAAQPGGYYNGTRGDRNKLRHLISVKLGKIPTVENSIFHMAAWSGSACHDGKEWTYIGVDGPDNNALLKIKYGEAYTDTYHSYANNILRTQESACNCIGGNCYLMITDGSASGVSECRFLKIREGRIIKEIFPTGRVKHTEECTCGFASNKTIECACRDNSYTAKRPFVKLNVETDTAEIRLMCTETYLDTPRPDDGSITGPCESNGDKGSGGIKGGFVHQRMASKIGRWYSRTMSKTERMGMGLYVKYDGDPWADSDALAFSGVMVSMKEPGWYSFGFEIKDKKCDVPCIGIEMVHDGGKETWHSAATAIYCLMGSGQLLWDTVTGVNMAL |
| B/Singapore/EN071/2008 | 2008 | LPSTIQMLTLFLTLGGVLLSLYVSASLSYLLYSDILLKFPPTEITAPTMPLDCANASNVQAVNRSATKGVTLLLPEPEWTYPRLSCPGSTFQKALLISPHRFGETKGNSAPLIIREPFIACGPKECKHFALTHYAAQPGGYYNGTRGDRNKLRHLISVKLGKIPTVENSIFHMAAWSGSACHDGKEWTYIGVDGPDNNALLKIKYGEAYTDTYHSYANNILRTQESACNCIGGNCYLMITDGSASGVSECRFLKIREGRIIKEIFPTGRVKHTEECTCGFASNKTIECACRDNSYTAKRPFVKLNVETDTAEIRLMCTETYLDTPRPDDGSITGPCESNGDKGSGGIKGGFVHQRMASKIGRWYSRTMSKTERMGMGLYVKYDGDPWADSDALAFSGVMVSMKEPGWYSFGFEIKDKKCDVPCIGIEMVHDGGKETWHSAATAIYCLMGSGQLLWDTVTGVNMAL |
| B/Sydney/13/2008 | 2008 | LPSTIQTLTLFLTSGGVLLSLYVSASLSYLLYSDILLKFSQTEITAPIMPLDCANASNVQAVNRSAAKGVTLLLPEPEWTYPRLSCPGSTFQKALLISPHRFGETKGNSAPLIIREPFIACGPTECKHFALTHYAAQPGGYYNGTREDRNKLRHLISVKLGKIPTVENSIFHMAAWSGSACHDGKEWTYIGVDGPDSNALLKIKYGEAYTDTYHSYAKNILRTQESACNCIGGDCYLMITDGPASGISECRFLKIREGRIIKEIFPTGRVKHTEECTCGFASNKTIECACRDNRYTAKRPFVKLNVETDTAEIRLMCTETYLDTPRPNDGSITGPCESDGDKGSGGIKGGFVHQRMASKIGRWYSRTMSKTKRMGMGLYVKYDGDPWTDSGALALSGVMVSIEEPGWYSFGFEIKDKKCDVPCIGIEMVHDGGKTTWHSAATAIYCLMGSGQLLWDTVTGVDMAL |
| B/Sydney/2/2008 | 2008 | LPSTIQTLTLFLTSGGVLLSLYVSASLSYLLYSDILLKFSRTEITAPIMPLDCANASNVQAVNRSATKGVTLLLPEPEWTYPRLSCPGSTFQKALLISPHRFGETKGNSAPLIIREPFIACGPKECKHFALTHYAAQPGGYYNGTREDRNKLRHLISVKLGKIPTVENSIFHMAAWSGSACHDGREWTYIGVDGPDSNALLKIKYGEAYTDTYHSYAKNILRTQESACNCIGGDCYLMITDGPASGISECRFLKIREGRIIKEIFPTGRVKHTEECTCGFASNKTIECACRDNSYTAKRPFVKLNVETDTAEIRLMCTETYLDTPRPNDGSITGPCESNGDKGSGGIKGGFVHQRMASKIGRWYSRTMSKTKRMGMGLYVKYDGDPWTDSEALALSGVMVSMEEPGWYSFGFEIKDKKCDVPCIGIEMVHDGGKTTWHSAATAIYCLMGSGQLLWDTVTGVNMTL |
| B/Sydney/24/2008 | 2008 | LPSTIQTLTLFLTSGGVLLSLYVSASLSYLLYSDILLKFSQTEITAPIMPLDCANASNVQAVNRSAAKGVTLLLPEPEWTYPRLSCPGSTFQKALLISPHRFGETKGNSAPLIIREPFIACGPTECKHFALTHYAAQPGGYYNGTREDRNKLRHLISVKLGKIPTVENSIFHMAAWSGSACHDGKEWTYIGVDGPDSNALLKIKYGEAYTDTYHSYAKNILRTQESACNCIGGDCYLMITDGPASGISECRFLKIREGRIIKEIFPTGRVKHTEECTCGFASNKTIECACRDNSYTAKRPFVKLNVETDTAEIRLMCTETYLDTPRPNDGSITGPCESDGDKGSGGIKGGFVHQRMASKIGRWYSRTMSKTRRMGMGLYVKYDGDPWTDSEALALSGVMVSMEEPGWYSFGFEIKDKKCDVPCIGIEMVHDGGKTTWHSAATAIYCLMGSGQLLWDTVTGVDMAL |
| B/Sydney/521/2008 | 2008 | LPSTIQTLTLFLTSGGVLLSLYVSASLSYLLYSDILLKFSPTEITAPTMPLDCANASNVHAVNHSATKGVTLLLPEPEWTYPRLSCPGSTFQKALLISPHRFGETKGNSAPLIIREPFIACGPNECKHFALTHYAAQPGGYYNGTRGDRNKLRHLISVKLGKIPTVENSIFHMAAWSGSACHDGKEWTYIGVDGPDNNALLKIKYGEAYTDTYHSYANNILRTQESACNCIGGNCYLMVTDGSASGVSECRFLKIREGRIIKEIFPTGRVKHTEECTCGFASNKTIECACRDNSYTAKRPFVKLNVETDTAEIRLMCTDTYLDTPRPDDGSITGPCESNGDKGSGGIKGGFVHQRMASKIGRWYSRTMSKTERMGMGLYVKYDGDPWADSDALAFSGVMVSMKEPGWYSFGFEIKDKKCDVPCIGIEMVHDGGKETWHSAATAIYCLMGSGQLLWDTVTGVDMAL |
| B/Sydney/538/2008 | 2008 | LPSTIQTLTLFLTSGGVLLSLYVSASLSYLLYSDILLKFSPTEITAPTMPLDCANASNVQAVNRSATKGVTLLLPEPEWTYPRLSCPGSTFQKALLISPHRFGETKGNSAPLIIREPFIACGPNECKHFALTHYAAQPGGYYNGTRGDRNKLRHLISVKLGKIPTVENSIFHMAAWSGSACHDGKEWTYIGVDGPDNNALLKVKYGEAYTDTYHSYANKILRTQESACNCIGGNCYLMITDGSASGVSECRFLKIREGRIIKEIFPTGRVKHTEECTCGFASNKTIECACRDNSYTAKRPFVKLNVETDTAEIRLMCTDTYLDTPRPNDGSITGPCESNGDKGSGGIKGGFVHQRMESKIGRWYSRTMSKTERMGMGLYVKYDGDPWADSDALAFSGVMVSMKEPGWYSFGFEIKDKKCDVPCIGIEMVHDGGKETWHSAATAIYCLMGSGQLLWDTVTGVDMAL |
| B/Sydney/548/2008 | 2008 | LPSTIQTLTLFLTSGGVLLSLYVSASLSYLLYSDILLKFSPTEITAPTMPLDCANASNVHAVNHSATKGVTLLLPEPEWTYPRLSCPGSTFQKALLISPHRFGETKGNSAPLIIREPFIACGPNECKHFALTHYAAQPGGYYNGTRGDRNKLRHLISVKLGKIPTVENSIFHMAAWSGSACHDGKEWTYIGVDGPDNNALLKIKYGEAYTDTYHSYANNILRTQESACNCIGGNCYLMVTDGSASGVSECRFLKIREGRIIKEIFPTGRVKHTEECTCGFASNKTIECACRDNSYTAKRPFVKLNVETDTAEIRLMCTDTYLDTPRPDDGSITGPCESNGDKGSGGIKGGFVHQRMASKIGRWYSRTMSKTERMGMGLYVKYDGDPWADSDALAFSGVMVSMKEPGWYSFGFEIKDKKCDVPCIGIEMVHDGGKETWHSAATAIYCLMGSGQLLWDTVTGVDMAL |
| B/Sydney/61/2008 | 2008 | LPSTIQTLTLFLTSGGVLLSLYVSASLSYLLYSDILLKFSRTEITAPIMPLDCANASNVQAVNRSATKGVTLLLPEPEWTYPRLSCPGSTFQKALLISPHRFGETKGNSAPLIIREPFIACGPKECKHFALTHYAAQPGGYYNGTREDRNKLRHLISVKLGKIPTVENSIFHMAAWSGSACHDGREWTYIGVDGPDSNALLKIKYGEAYTDTYHSYAKNILRTQESACNCIGGDCYLMITDGPASGISECRFLKIREGRIIKEIFPTGRVKHTEECTCGFASNKTIECACRDNSYTAKRPFVKLNVETDTAEIRLMCTETYLDTPRPNDGSITGPCESNGDKGSGGIKGGFVHQRMASKIGRWYSRTMSKTKRMGMGLYVKYDGDPWTDSEALTLSGVMVSMEEPGWYSFGFEIKDKKCDVPCIGIEMVHDGGKTTWHSAATAIYCLMGSGQLLWDTVTGVNMTL |
| B/Sydney/76/2008 | 2008 | LPSTIQTLTLFLTSGGVLLSLYVSASLSYLLYSDILLKFSQTEITAPIMPLDCANASNVQAVNRSAAKGVTLLIPEPEWTYPRLSCPGSTFQKALLISPHRFGETKGNSAPLIIREPFIACGPTECKHFALTHYAAQPGGYYNGTREDRNKLRHLISVKLGKIPTVENSIFHMAAWSGSACHDGKEWTYIGVDGPDSNALLKIKYGEAYTDTYHSYAKNILRTQESACNCIGGDCYLMITDGPASGISECRFLKIREGRIIKEIFPTGRVKHTEECTCGFASNKTIECACRDNSYTAKRPFVKLNVETDTAEIRLMCTETYLDTPRPNDGSITGPCESDGDKGSGGIKGGFVHQRMASKIGRWYSRTMSKTKRMGMGLYVKYDGDPWTDSEALALSGVMVSMEEPGWYSFGFEIKDKKCDVPCIGIEMVHDGGKTTWHSAATAIYCLMGSGQLLWDTVTGVDMAL |
| B/Taiwan/418/2008 | 2008 | LPSTIQTLTLFLTSGGVLLSLYVSASLSYLLYSDILLKFSRTEITAPIMPLDCANASNVQAVNRSATKGVTLLLPEPEWTYPRLSCPGSTFQKALLISPHRFGETKGNSAPLIIREPFIACGPKECKHFALTHYAAQPGGYYNGTREDRNKLRHLISVKLGKIPTVENSIFHMAAWSGSACHDGREWTYIGVDGPDSNALLKIKYGEAYTDTYHSYAKNILRTQESACNCIGGDCYLMITDGPASGISECRFLKIREGRIIKEIFPTGRVKHTEECTCGFASNKTIECACRDNSYTAKRPFVKLNVETDTAEIRLMCTETYLDTPRPNDGSITGPCESNGDKGSGGIKGGFVHQRMASKIGRWYSRTMSKTKRMGMGLYVKYDGDPWTDSEALALSGVMVSMEEPGWYSFGFEIKDKKCDVPCIGIEMVHDGGKTTWHSAATAIYCLMGSGQLLWDTVTGVNMTL |
| B/Thailand/Siriraj ICRC SEA-3604/2008 | 2008 | LPSTIQTLTLFLTSGGVLLSLYVSASLSYLLYSDILLKFPSTEITAPTMPLDCANASNVQAVNRSATKGVTLLLPEPEWTYPRLSCPGSTFQKALLISPHRFGETKGNSAPLIIREPFIACGPKECKHFALTHYAAQPGGYYNGTRGDRNKLRHLISVKLGKIPTVENSIFHMAAWSGSACHDGKEWTYIGVDGPDNNALLKIKYGEAYTDTYHSYANNILRTQESACNCIGGNCYLMITDGSASGVSECRFLKIREGRIIKEIFPTGRIKHTEECTCGFASNKTIECACRDNSYTAKRPFVKLNVETDTAEIRLMCTETYLDTPRPDDGSITGPCESNGGKGSGGIKGGFVHQRMASKIGRWYSRTMSKTERKGMGLYVKYDGDPWADSDALVFSGVMVSMEEPGWYSFGFEIKDKKCDVPCIGIEMVHDGGKETWHSAATAIYCLMGSGQLLWDTVTGVNMAL |
| B/TOCHIGI/15/2008 | 2008 | LPSTIQTLTLFLTSGGVLLSLYVSASLSYLLYSDILLKFSPTEITAPTMPLDCANASNVHAVNHSATKGVTLLLPEPEWTYPRLSCPGSTFQKALLISPHRFGETKGNSAPLIIREPFIACGPNECKHFALTHYAAQPGGYYNGTRGDRNKLRHLISVKLGKIPTVENSIFHMAAWSGSACHDGKEWTYIGVDGPDNNALLKIKYGEAYTDTYHSYANNILRTQESACNCIGGNCYLMVTDGSASGVSECRFLKIREGRIIKEIFPTGRVKHTEECTCGFASNKTIECACRDNSYTAKRPFVKLNVETDTAEIRLMCTDTYLDTPRPDDGSITGPCESNGDKGSGGIKGGFVHQRMASKIGRWYSRTMSKTERMGMGLYVKYDGDPWADSDALAFSGVMVSMKEPGWYSFGFEIKDKKCDVPCIGIEMVHDGGKETWHSAATAIYCLMGSGQLLWDTVTGVDMAL |
| B/Ulaanbaatar/0176/2008 | 2008 | LPSTIQTLTLFLTSGGVLLSLYVSASLSYLLYSDILLKFSQTEITTPIMPLDCANASNVQAVNRSAAKGVTLLLPEPEWTYPRLSCPGSTFQKALLISPHRFGETKGNSAPLIIREPFIACGPTECKHFALTHYAAQPGGYYNGTREDRNKLRHLISVKLGKIPTVENSIFHMAAWSGSACHDGKEWTYIGVDGPDSNALLKIKYGEAYTDTYHSYAKNILRTQESACNCIGGDCYLMITDGPASGVSECRFLKIREGRIIKEIFPTGRVKHTEECTCGFASNKTIECACRDNSYTAKRPFVKLNVETDTAEIRLMCTETYLDTPRPNDGSITGPCESDGDKGSGGIKGGFVHQRMASKIGRWYSRTMSKTKRMGMGLYVKYDGDPWTDSEALALSGVMVSMEEPGWYSFGFEIKDKKCDVPCIGIEMVHDGGKTTWHSAATAIYCLMGSGQLLWDTVTGVDMAL |
| B/Victoria/514/2008 | 2008 | LPSTIQTLTLFLTSGGVLLSLYVSASLSYLLYSDILLKFSPTEITAPTMPLDCANASNVQAVNRSATKGVTLLLPEPEWTYPRLSCPGSTFQKALLISPHRFGETKGNSAPLIIREPFIACGPNECKHFALTHYAAQPGGYYNGTRGDRNKLRHLISVKLGKIPTVENSIFHMAAWSGSACHDGKEWTYIGVDGPDNNALLKVKYGEAYTDTYHSYANKILRTQESACNCIGGNCYLMITDGSASGVSECRFLKIREGRIIKEIFPTGRVKHTEECTCGFASNKTIECACRDNSYTAKRPFVKLNVETDTAEIRLMCTDTYLDTPRPNDGSITGPCESNGDKGSGGIKGGFVHQRMESKIGRWYSRTMSKTERMGMGLYVKYDGDPWADSDALAFSGVMVSMKEPGWYSFGFEIKDKKCDVPCIGIEMVHDGGKETWHSAATAIYCLMGSGQLLWDTVTGVDMAL |
| B/Victoria/521/2008 | 2008 | LPSTIQTLTLFLTSGGVLLSLYVSASLSYLLYSDILLKFSPTEITAPTMPLDCANASNVHAMNHSATKGVTLLLPEPEWTYPRLSCPGSTFQKALLISPHRFGETKGNSAPLIIREPFIACGPNECKHFALTHYAAQPGGYYNGTRGDRNKLRHLISVKLGKIPTVENSIFHMAAWSGSACHDGKEWTYIGVDGPDNNALLKIKYGEAYTDTYHSYANNILRTQESACNCIGGNCYLMVTDGSASGVSECRFLKIREGRIIKEIFPTGRVKHTEECTCGFASNKTIECACRDNSYTAKRPFVKLNVETDTAEIRLMCTDTYLDTPRPDDGSITGPCESNGDKGSGGIKGGFVHQRMASKIGRWYSRTMSKTERMGMGLYVKYDGDPWADSDALAFSGVMVSMKEPGWYSFGFEIKDKKCDVPCIGIEMVHDGGKETWHSAATAIYCLMGSGQLLWDTVTGVDMAL |
| B/Wellington/13/2008 | 2008 | LPSTIQTLTLFLTSGGVLLSLYVSASLSYLLYSDILLKFSQTEITAPIMPLDCANASNVQAVNRSAAKGVTLLLPEPEWTYPRLSCPGSTFQKALLISPHRFGETKGNSAPLIIREPFIACGPTECKHFALTHYAAQPGGYYNGTREDRNKLRHLISVKLGKIPTVENSIFHMAAWSGSACHDGKEWTYIGVDGPDSNALLKIKYGEAYTDTYHSYAKNILRTQESACNCIGGDCYLMITDGPASGISECRFLKIREGRIIKEIFPTGRVKHTEECTCGFASNKTIECACRDNRYTAKRPFVKLNVETDTAEIRLMCTETYLDTPRPNDGSITGPCESDGDKGSGGIKGGFVHQRMASKIGRWYSRTMSKTKRMGMGLYVKYDGDPWTDSGALALSGVMVSIEEPGWYSFGFEIKDKKCDVPCIGIEMVHDGGKTTWHSAATAIYCLMGSGQLLWDTVTGVDMAL |
| B/YAMAGATA/118/2008 | 2008 | LPSTIQTLTLFLTSGGVLLSLYASASLSYLLYSDILLKFSQTEITAPIMPLDCANASNVQAVNRSAAKGVTLLLPEPEWTYPRLSCPGSTFQKALLISPHRFGETKGNSAPLIIREPFIACGPTECKHFALTHYAAQPGGYYNGTREDRNKLRHLISVKLGKIPTVENSIFHMAAWSGSACHDGKEWTYIGVDGPDSNALLKIKYGEAYTDTYHSYAKNILRTQESACNCIGGDCYLMITDGPASGISECRFLKIREGRIIKEIFPTGRVKHTEECTCGFASNKTIECACRDNSYTAKRPFVKLNVETDTAEIRLMCTETYLDTPRPNDGSITGPCESDGDKGSGGIKGGFVHQRMASKIGRWYSRTMSKTRRMGMGLYVKYDGDPWTDSEALALSGVMVSMEEPGWYSFGFEIKDKKCDVPCIGIEMVHDGGKTTWHSAATAIYCLMGSGQLLWDTVTGVDMAL |
| B/AICHI/16/2009 | 2009 | LPSTIQTLTLFLTSGGVLLSLYVSASLSYLLYSDILLKFSPTEITAPTMPLDCANASNVQAVNRSATKGVTLLLPEPEWTYPRLSCPGSAFQKALLISPHRFGETKGNSAPLIIREPFIACGPNECKHFALTHYAAQPGGYYNGTRGDRNKLRHLISVKLGKIPTVENSIFHMAAWSGSACHDGKEWTYIGVDGPDNNALLKVKYGEAYTDTYHSYANKILRTQESACNCIGGNCYLMITDGSASGVSECRFLKIREGRIIKEIFPTGRVKHTEECTCGFASNKTIECACRDNSYTAKRPFVKLNVETDTAEIRLMCTDTYLDTPRPNDGSITGPCESNGDKGSGGIKGGFVHQRMESKIGRWYSRTMSKTERMGMGLYVKYDGDPWADSDALAFSGVMVSMKEPGWYSFGFEIKDKKCDVPCIGIEMVHDGGKETWHSAATAIYCLMGSGQLLWDTVTGVDMAL |
| B/AICHI/30/2009 | 2009 | LPSTIQTLTLFLTSGGVLLSLYVSASLSYLLYSDLLLKFSPTEITAPTMPLDCANASNVQAVNRSATKGVTLLLPEPEWTYPRLSCPGSTFQKALLISPHRFGETKGNSAPLIIREPFIACGPNECKHFALTHYAAQPGGYYNGTRGDRNKLRHLISVKLGKIPTVENSIFHMAAWSGSACHDGKEWTYIGVDGPDNNALLKVKYGEAYTDTYHSYANKILRTQESACNCIGGNCYLMITDGSASGVSECRFLKIREGRIIKEIFPTGRVKHTEECTCGFASNKTIECACRDNSYTAKRPFVKLNVETDTAEIRLMCTDTYLDTPRPNDGSITGPCESNGDKGSGGIKGGFVHQRMESKIGRWYSRTMSKTERMGMGLYVKYDGDPWADSDALVFSGVMVSMKEPGWYSFGFEIKDKKCDVPCIGIEMVHDGGKETWHSAATAIYCLMGSGQLLWDTVTGVDMAL |
| B/CHANTHABURI/329/2009 | 2009 | LPSTIQTLTLFLTSGGVLLSLYVSASLSYLLYSDILLKFSPTEITAPTMPLDCANASNVQAVNRSATKGVTLLLPEPEWTYPRLSCPGSTFQKALLISPHRFGETKGNSAPLIIREPFIACGPNECKHFALTHYAAQPGGYYNGTRGDRNKLRHLISVKLGKIPTVENSIFHMAAWSGSACHDGKEWTYIGVDGPDNNALLKVKYGEAYTDTYHSYANKILRTQESACNCIGGNCYLMITDGSASGVSECRFLKIREGRIIKEIFPTGRVKHTEECTCGFASNKTIECACRDNSYTAKRPFVKLNVETDTAEIRLMCTDTYLDTPRPNDGSITGPCESNGDKGSGGIKGGFVHQRMESKIGRWYSRTMSKTERMGMGLYVKYDGDPWADSDALAFSGVMVSMKEPGWYSFGFEIKDKKCDVPCIGIEMVHDGGKETWHSAATAIYCLMGSGQLLWDTVTGVNMAL |
| B/Chongqing-yuzhong/194/2009 | 2009 | LPSTIQTLTLFLTSGGVLLSLYVSASLSYLLYSDILLKFSPTEITAPTMSLDCANASNVQAVNRSATKGVTFLLPEPEWTYPRLSCPGSTFQKALLISPHRFGETKGNSAPLIIREPFIACGPNECKHFALTHYAAQPGGYYNGTRGDRNKLRHLISVKLGKIPTVENSIFHMAAWSGSACHDGKEWTYIGVDGPDNNALLKVKYGEAYTDTYHSYANKILRTQESACNCIGGNCYLMITDGSASGVSECRFLKIREGRIIKEIFPTGRVKHTEECTCGFASNKTIECACRDNSYTAKRPFVKLNVETDTAEIRLMCTDTYLDTPRPDDGSITGPCESNGDKGSGGIKGGFVHQRMESKIGRWYSRTMSKTERMGMGLYVKYDGDPWADSDALAFSGVMVSMKEPGWYSFGFEIKDKKCDVPCIGIEMVHDGGKETWHSAATAIYCLMGSGQLLWDTVTGVDMAL |
| B/FIJI/1/2009 | 2009 | LPSTIQTLTLFLTSGGVLLSLYVSASLSYLLYSDILLKFSPTEITAPTMPLDCANASNVQAVNRSATKGVTLLLPEPEWTYPRLSCPGSTFQKALLISPHRFGETKGNSAPLIIREPFIACGPNECKHFALTHYAAQPGGYYNGTRGDRNKLRHLISVKLGKIPTVENSIFHMAAWSGSACHDGKEWTYIGVDGPDNNALLKVKYGEAYTDTYHSYANKILRTQESACNCIGGNCYLMITDGSASGVSECRFLKIREGRIIKEIFPTGRVKHTEECTCGFASNKTIECACRDNSYTAKRPFVKLNVETDTAEIRLMCTDTYLDTPRPNDGSITGPCESNGDKGSGGIKGGFVHQRMESKIGRWYSRTMSKTERMGMGLYVKYDGDPWADSDALAFSGVMVSMKEPGWYSFGFEIKDKKCDVPCIGIEMVHDGGKETWHSAATAIYCLMGSGQLLWDTVTGVDMAL |
| B/Finland/543/2009 | 2009 | LPSTIQTLTLFLTSGGVLLSLYVSASLSYLLYSDILLKFSPTEITAPTMPLDCANASNVQAVNRSATKGVTLLLPEPEWTYPRLSCPGSTFQKALLISPHRFGETKGNSAPLIIREPFIACGPNECKHFALTHYAAQPGGYYNGTRGDRNKLRHLISVKLGKIPTVENSIFHMAAWSGSACHDGKEWTYIGVDGPDNNALLKVKYGEAYTDTYHSYANKILRTQESACNCIGGNCYLMITDGSASGVSECRFLKIREGRIIKEIFPTGRVKHTEECTCGFASNKTIECACRDNSYTAKRPFVKLNVETDTAEIRLMCTDTYLDTPRPNDGSITGPCESNGDKGSGGIKGGFVHQRMESKIGRWYSRTMSKTERMGMGLYVKYDGDPWADSDALAFSGVMVSMKEPGWYSFGFEIKDKKCDVPCIGIEMVHDGGKETWHSAATAIYCLMGSGQLLWDTVTGVDMAL |
| B/FUKUI/48/2009 | 2009 | LPSTIQTLTLLLTSGGVLLSLYVSASLSYLLYSDILLKFSPTEITAPTMPLDCANASNVQAVNRSATKGVTLLLPEPEWTYPRLSCPGSTFQKALLISPHRFGETKGNSAPLIIREPFIACGPNECKHFALTHYAAQPGGYYNGTRGDRNKLRHLISVKLGKIPTVENSIFHMAAWSGSACHDGKEWTYIGVDGPDNNALLKVKYGEAYTDTYHSYANKILRTQESACNCIGGNCYLMITDGSASGVSECRFLKIREGRIIKEIFPTGRVKHTEECTCGFASNKTIECACRDNSYTAKRPFVKLNVETDTAEIRLMCTDTYLDTPRPNDGSITGPCESNGDKGSGGIKGGFVHQRMESKIGRWYSRTMSKTERMGMGLYVKYDGDPWADSDALAFSGVMVSMKEPGWYSFGFEIKDKKCDVPCIGIEMVHDGGKETWHSAATAIYCLMGSGQLLWDTVTGVDMAL |
| B/FUKUOKA/1/2009 | 2009 | LPSTIQTLTLFLTSGGVLLSLYVSASLSYLLYSDILLKFSPTEITAPTMPLDCANASNVQAVNRSATKGVTLLLPEPEWTYPRLSCPGSTFQKALLISPHRFGETKGNSAPLIIREPFIACGPNECKHFALTHYAAQPGGYYNGTRGDRNKLRHLISVKLGKIPTVENSIFHMAAWSGSACHDGKEWTYIGVDGPDNNALLKVKYGEAYTDTYHSYANKILRTQESACNCIGGNCYLMITDGSASGVSECRFLKIREGRIIKEIFPTGRVKHTEECTCGFASNKTIECACRDNSYTAKRPFVKLNVETDTAEIRLMCTDTYLDTPRPNDGSITGPCESNGDKGSGGIKGGFVHQRMESKIGRWYSRTMSKTERMGMGLYVKYDGDPWADSDALAFSGVMVSMKEPGWYSFGFEIKDKKCDVPCIGIEMVHDGGKETWHSAATAIYCLMGSGQLLWDTVTGVDMAL |
| B/Guangdong-xinxing/145/2009 | 2009 | LPSTIQTLTLFLTSGGVLLSLYVSASLSYLLYSDILLKFPSTEITAPTMPLDCANASNVQAVNLSATKGVTLLLPEPEWTYPRLSCPGSTFQKALLISPHRFGETKGNSAPLIIREPFIACGPKECKHFALTHYAAQPGGYYNGTRGDRNKLRHLISVKLGKIPTVENSIFHMAAWSGSACHDGKEWTYIGVDGPDNNALLKIKYGEAYTDTYHSYANNILRTQESACNCIGGNCYLMITDGSASGVSECRFLKIREGRIIKEIFPTGRIQHTEECTCGFASNKTIECACRDNSYTAKRPFVKLNVETDTAEIRLMCTKTYLDTPRPDDGSITGPCESNGGKGSGGIKGGFVHQRMASKIGRWYSRTMSKTERKGMGLYVKYDGDPWADSDALAFSGVMVSMEEPGWYSFGFEIKDKKCDVPCIGIEMVHDGGKETWHSAATAIYCLMGSGQLLWDTVTGVNMAL |
| B/HAMAMATU-C/9109/2009 | 2009 | LPSTIQTLTLFLTSGGVLLSLYVSASLSYLLYSDILLKFSPTEITAPTMPLDCANASNVQAVNRSATKGVTFLLPEPEWTYPRLSCPGSTFQKALLISPHRFGETKGNSAPLIIREPFIACGPNECKHFALTHYAAQPGGYYNGTRGDRNKLRHLISVKLGKIPTVENSIFHMAAWSGSACHDGKEWTYIGVDGPDNNALLKVKYGEAYTDTYHSYANKILRTQESACNCIGGNCYLMITDGSASGVSECRFLKIREGRIIKEIFPTGRVKHTEECTCGFASNKTIECACRDNSYTAKRPFVKLNVETDTAEIRLMCTDTYLDTPRPDDGSITGPCESNGDKGSGGIKGGFVHQRMESKIGRWYSRTMSKTERMGMGLYVKYDGDPWADSDALAFSGVMVSMKEPGWYSFGFEIKDKKCDVPCIGIEMVHDGGKETWHSAATAIYCLMGSGQLLWDTVTGVDMAL |
| B/HIROSHIMA-C/2/2009 | 2009 | LPSTIQTLTLFLTSGGVLLSLYVSASLSYLLYSDILLKFSRTEITAPIMPLDCANASNVQAVNRSATKGVTLLLPEPEWTYPRLSCPGSTFQKALLISPHRFGETKGNSAPLIIREPFIACGPKECKHFALTHYAAQPGGYYNGTREDRNKLRHLISVKLGKIPTVENSIFHMAAWSGSACHDGREWTYIGVDGPDNNALLKIKYGEAYTDTYHSYAKNILRTQESACNCIGGDCYLMITDGSASGISECRFLKIREGRIIKEIFPTGRVKHTEECTCGFASNKTIECACRDNSYTAKRPFVKLNVETDTAEIRLMCTETYLDTPRPNDGSITGPCESNGDKGSGGIKGGFVHQRMASKIGRWYSRTMSKTKRMGMGLYVKYDGDPWTDSEALALSGVMVSMEEPGWYSFGFEIKDKKCDVPCIGIEMVHDGGKTTWHSAATAIYCLMGSGQLLWDTVTGVNMTL |
| B/Hubei-jiang an/154/2009 | 2009 | LPSTIQTLTLFLTSGGVLLSLYVSASLSYLLYSDILLKFPSTEITAPTMPLDCANASNVQAVNRSATKGVTLLLPEPEWTYPRLSCPGSTFQKALLISPHRFGETKGNSAPLIIREPFIACGPKECKHFALTHYAAQPGGYYNGTRGDRNKLRHLISVKLGKIPTVENSIFHMAAWSGSACHDGKEWTYIGVDGPDNNALLKIKYGEAYTDTYHSYANNILRTQESACNCIGGNCYLMITDGSASGVSECRFLKIREGRIIKEIFPTGRIKHTEECTCGFASNKTIECACRDNSYTAKRPFVKLNVETDTAEIRLMCTETYLDTPRPDDGSITGPCESNGGKGSGGIKGGFVHQRMASKIGRWYSRTMSKTERKGMGLYVKYDGDPWADSDALAFSGVMVSMEEPGWYSFGFEIKDKKCDVPCIGIEMVHDGGKETWHSAATAIYCLMGSGQLLWDTVTGVNMAL |
| B/Hunan-wuling/33/2009 | 2009 | LPSTIQTLTLFLTSGGVLLSLYVSASLSYLLYSDILLKFPSTEITAPTMPLDCANASNVQAVNRSATKGVTLLLPEPEWTYPRLSCPGSTFQKALLISPHRFGETKGNSAPLIIREPFIACGPKECKHFALTHYAAQPGGYYNGTRGDRNKLRHLISVKLGKIPTVENSIFHMAAWSGSACHDGKEWTYIGVDGPDNNALLKIKYGEAYTDTYHSYANNILRTQESACNCIGGNCYLMITDGSASGVSECRFLKIREGRIIKEIFPTGRIQHTEECTCGFASNKTIECACRDNSYTAKRPFVKLNVETDTAEIRLMCTKTYLDTPRPDDGSITGPCESNGGKGSGGIKGGFVHQRMASKIGRWYSRTMSKTERKGMGLYVKYDGDPWADSDALAFSGVMVSMEEPGWYSFGFEIKDKKCDVPCIGIEMVHDGGKETWHSAATAIYCLMGSGQLLWDTVTGVNMAL |
| B/ISHIKAWA/1/2009 | 2009 | LPSTIQTLTLFLTSGGVLLSLYVSASLSYLLYSDILLKFSPTEITAPTMPLDCANASNIQAVNRSATKGVTLLLPEPEWTYPRLSCPGSTFQKALLISPHRFGETKGNSAPLIIREPFIACGPNECKHFALTHYAAQPGGYYNGTRGDRNKLRHLISVKLGKIPTVENSIFHMAAWSGSACHDGKEWTYIGVDGPDNNALLKVKYGEAYTDTYHSYANKILRTQESACNCIGGNCYLMITDGSASGVSECRFLKIREGRIIKEIFPTGRVKHTEECTCGFASNKTIECACRDNSYTAKRPFVKLNVETDTAEIRLMCTDTYLDTPRPNDGSITGPCESNGDKGSGGIKGGFVHQRMESKIGRWYSRTMSKTERMGMGLYVKYDGDPWADSDALAFSGVMVSMKEPGWYSFGFEIKDKKCDVPCIGIEMVHDGGKETWHSAATAIYCLMGSGQLLWDTVTGVDMAL |
| B/IWATE/15/2009 | 2009 | LPSTIQTLTLFLTSGGVLLSLYVSASLSYLLYSDILLKFSPTEITAPTMPLDCVNASNVQAVNRSATKGVTLLLPEPEWTYPRLSCPGSTFQKALLISPHRFGETKGNSAPLIIREPFIACGPNECKHFALTHYAAQPGGYYNGTRGDRNKLRHLISVKLGKIPTVENSIFHMAAWSGSACHDGKEWTYIGVDGPDNNALLKVKYGEAYTDTYHSYANKILRTQESACNCIGGNCYLMITDGSASGVSECRFLKIREGRIIKEIFPTGRVKHTEECTCGFASNKTIECACRDNSYTAKRPFVKLNVETDTAEIRLMCTDTYLDTPRPNDGSITGPCESNGDKGSGGIKGGFVHQRMESKIGRWYSRTMSKTERMGMGLYVKYDGDPWADSDALAFSGVMVSMKEPGWYSFGFEIKDKKCDVPCIGIEMVHDGGKETWHSAATAIYCLMGSGQLLWDTVTGVDMAL |
| B/KAGOSHIMA/15/2009 | 2009 | LPSTIQTLTLFLTSGGVLLSLYVSASLSYLLYSDILLKFSPTEITAPTMPLDCANASNVQAVNRSATKGVTFLLPEPEWTYPRLSCPGSTFQKALLISPHRFGETKGNSAPLIIREPFIACGPNECKHFALTHYAAQPGGYYNGTRGDRNKLRHLISVKLGKIPTVENSIFHMAAWSGSACHDGKEWTYIGVDGPDNNALLKVKYGEAYTDTYHSYANKILRTQESACNCIGGNCYLMITDGSASGVSECRFLKIREGRIIKEIFPTGRVKHTEECTCGFASNKTIECACRDNSYTAKRPFVKLNVETDTAEIRLMCTDTYLDTPRPDDGSITGPCESNGDKGSGGIKGGFVHQRMESKIGRWYSRTMSKTERMGMGLYVKYGGDPWADSDALAFSGVMVSMKEPGWYSFGFEIKDKKCDVPCIGIEMVHDGGKETWHSAATAIYCLMGSGQLLWDTVTGVDMAL |
| B/KOCHI/4/2009 | 2009 | LPSTIQTLTLFLTSGGVLLSLYVSASLSYLLYSDILLKFSPTEITAPTMPLDCANASNIQAVNRSATKGVTLLLPEPEWTYPRLSCPGSTFQKALLISPHRFGETKGNSAPLIIREPFIACGPNECKHFALTHYAAQPGGYYNGTRGDRNKLRHLISVKLGKIPTVENSIFHMAAWSGSACHDGKEWTYIGVDGPDNNALLKVKYGEAYTDTYHSYANKILRTQESACNCIGGNCYLMITDGSASGVSECRFLKIREGRIIKEIFPTGRVKHTEECTCGFASNKTIECACRDNSYTAKRPFVKLNVETDTAEIRLMCTDTYLDTPRPNDGSITGPCESNGDKGSGGIKGGFVHQRMESKIGRWYSRTMSKTERMGMGLYVKYDGDPWADSDALAFSGVMVSMKEPGWYSFGFEIKDKKCDVPCIGIEMVHDGGKETWHSAATAIYCLMGSGQLLWDTVTGVDMAL |
| B/Malaysia/5693/2009 | 2009 | LPSTIQTLTLFLTSGGVLLSLYVSASLSYLLYSDILLKFSPTEITAPTMPLDCANASNVQAVNRSATKGVTLLLPEPEWTYPRLSCPGSTFQKALLISPHRFGETKGNSAPLIIREPFIACGPNECKHFALTHYAAQPGGYYNGTRGDRNKLRHLISVKLGKIPTVENSIFHMAAWSGSACHDGKEWTYIGVDGPDNNALLKVKYGEAYTDTYHSYANKILRTQESACNCIGGNCYLMITDGSASGVSECRFLKIREGRIIKEIFPTGRVKHTEECTCGFASNKTIECACRDNSYTAKRPFVKLNVETDTAEIRLMCTDTYLDTPRPDDGSITGPCESNGDKGSGGIKGGFVHQRMESKIGRWYSRTMSKTERMGMGLYVKYDGDPWADSDALAFSGVMVSMKEPGWYSFGFEIKDKKCDVPCIGIEMVHDGGKETWHSAATAIYCLMGSGQLLWDTVTGVDMAL |
| B/MALAYSIA/947/2009 | 2009 | LPSTIQMLTLFLTLGGVLLSLYVSASLSYLLYSDILLKFPPTEITAPTMPLDCANASNVQAVNRSATKGVTLFLPEPEWTYPRLSCPGSTFQKALLISPHRFGETKGNSAPLIIREPFIACGPKECKHFALTHYAAQPGGYYNGTRGDRNKLRHLISVKLGKIPTVENSIFHMAAWSGSACHDGKEWTYIGVDGPDNNALLKIKYGEAYTDTYHSYANNILRTQESACNCIGGNCYLMITDGSASGVSECRFLKIREGRIIKEIFPTGRVKHTEECTCGFANNKTIECACRDNSYTAKRPFVKLNVETDTAEIRLMCTETYLDTPRPDDGSITGPCESNGDKGSGGIKGGFVHQRMASKIGRWYSRTMSKTERMGMGLYVKYDGDPWADSDALAFSGVMVSMKEPGWYSFGFEIKDKKCDVPCIGIEMVHDGGKETWHSAATAIYCLMGSGQLLWDTVTGVNMAL |
| B/Mauritius/515/2009 | 2009 | LPSTIQTLTLFLTSGGVLLSLYVSASLSYLLYSDILLKFSPTEIIAPTMPLDCANASNVQAVNRSATKGVTLLLPEPEWTYPRLSCPGSTFQKALLISPHRFGETKGNSAPLIIREPFIACGPNECKHFALTHYAAQPGGYYNGTRGDRNKLRHLISVKLGKIPTVENSIFHMAAWSGSACHDGKEWTYIGVDGPDNNALLKVKYGEAYTDTYHSYANKILRTQESACNCIGGNCYLMITDGSASGVSECRFLKIREGRIIKEIFPTGRVKHTEECTCGFASNKTIECACRDNSYTAKRPFVKLNVETDTAEIRLMCTDTYLDTPRPNDGSITGPCESNGDKGSGGIKGGFVHQRMESKIGRWYSRTMSKTERMGMGLYVKYDGDPWADSDALAFSGVMVSMKEPGWYSFGFEIKDKKCDVPCIGIEMVHDGGKETWHSAATAIYCLMGSGQLLWDTVTGVDMAL |
| B/MIYAGI/6/2009 | 2009 | LPSTIQTLTLFLTSGGVLLSLYVSASLSYLLYSDILLKFSPTEITAPTMPLDCANASNVHAVNHSATKGVTLLLPEPEWTYPRLSCPGSTFQKALLISPHRFGETKGNSAPLIIREPFIACGPNECKHFALTHYAAQPGGYYNGTRGDRNKLRHLISVKLGKIPTVENSIFHMAAWSGSACHDGKEWTYIGVDGPDNNALLKIKYGEAYTDTYHSYANNILRTQESACNCIGGNCYLMVTDGSASGVSECRFLKIREGRIIKEIFPTGRVKHTEECTCGFASNKTIECACRDNSYTAKRPFVKLNVETDTAEIRLMCTDTYLDTPRPDDGSITGPCESNGDKGSGGIKGGFVHQRMASKIGRWYSRTMSKTERMGMGLYVKYDGDPWADSDALAFSGVMVSMKEPGWYSFGFEIKDKKCDVPCIGIEMVHDGGKETWHSAATAIYCLMGSGQLLWDTVTGVDMAL |
| B/NAGANO/1226/2009 | 2009 | LPSTIQTLTLFLTSGGVLLSLYVSASLSYLLYSDILLKFSPTEITAPTMPLDCANASNVQAVNRSATKGVTLLLPEPEWTYPRLSCPGSTFQKALLISPHRFGETKGNSAPLIIREPFIACGPNECKHFALTHYAAQPGGYYNGTRGDRNKLRHLISVKLGKIPTVENSIFHMAAWSGSACHDGKEWTYIGVDGPDNNALLKVKYGEAYTDTYHSYANKILRTQESACNCIGGNCYLMITDGSASGVSECRFLKIREGRIIKEIFPTGRVKHTEECTCGFASNKTIECACRDNSYTAKRPFVKLNVETDTAEIRLMCTDTYLDTPRPNDGSITGPCESNGDKGSGGIKGGFVHQRMESKIGRWYSRTMSKTERMGMGLYVKYDGDPWADSDALAFSGVMVSMKEPGWYSFGFEIKDKKCDVPCIGIEMVHDGGKETWHSAATAIYCLMGSGQLLWDTVTGVDMAL |
| B/New York/1204/2009 | 2009 | LPSTIQTLTLFLTSGGVLLSLYVSASLSYLLYSDILLKFSPTEITAPTMPLDCANASNVQAVNRSATKGVTFLLPEPEWTYPRLSCPGSTFQKALLISPHRFGETKGNSAPLIIREPFIACGPNECKHFALTHYAAQPGGYYNGTRGDRNKLRHLISVKLGKIPTVENSIFHMAAWSGSACHDGKEWTYIGVDGPDNNALLKVKYGEAYTDTYHSYANKILRTQESACNCIGGNCYLMITDGSASGVSECRFLKIREGRIIKEIFPTGRVKHTEECTCGFASNKTIECACRDNSYTAKRPFVKLNVETDTAEIRLMCTDTYLDTPRPDDGSITGPCESNGDKGSGGIKGGFVHQRMESKIGRWYSRTMSKTERMGMGLYVKYDGDPWADSDALAFSGVMVSMKEPGWYSFGFEIKDKKCDVPCIGIEMVHDGGKETWHSAATAIYCLMGSGQLLWDTVTGVDMAL |
| B/New York/1208/2009 | 2009 | LPSTIQTLTLFLTSGGVLLSLYVSASLSYLLYSDILLKFSPTEITAPTMPLDCANASNVQAVNRSATKGVTLLLPEPEWTYPRLSCPGSTFQKALLISPHRFGETKGNSAPLIIREPFIACGPNECKHFALTHYAAQPGGYYNGTRGDRNKLRHLISVKLGKIPTVENSIFHMAAWSGSACHDGKEWTYIGVDGPDNNALLKVKYGEAYTDTYHSYANKILRTQESACNCIGGNCYLMITDGSASGVSECRFLKIREGRIIKEIFPTGRVKHTEECTCGFASNKTIECACRDNSYTAKRPFVKLNVETDTAEIRLMCTDTYLDTPRPNDGSITGPCESNGDKGSGGIKGGFVHQRMESKIGRWYSRTMSKTERMGMGLYVKYDGDPWADSDALAFSGVMVSMKEPGWYSFGFEIKDKKCDVPCIGIEMVHDGGKETWHSAATAIYCLMGSGQLLWDTVTGVDMAL |
| B/New York/1211/2009 | 2009 | LPSTIQTLTLFLTSGGVLLSLYVSASLSYLLYSDILLKFSPTEITAPTMPLDCANASNVQAVNRSATKGVTLLLPEPEWTYPRLSCPGSTFQKALLISPHRFGETKGNSAPLIIREPFIACGPNECKHFALTHYAAQPGGYYNGTRGDRNKLRHLISVKLGKIPTVENSIFHMAAWSGSACHDGKEWTYIGVDGPDNNALLKVKYGEAYTDTYHSYANKILRTQESACNCIGGNCYLMITDGSASGVSECRFLKIREGRIIKEIFPTGRVKHTEECTCGFASNKTIECACRDNSYTAKRPFVKLNVETDTAEIRLMCTDTYLDTPRPNDGSITGPCESNGDKGSGGIKGGFVHQRMESKIGRWYSRTMSKTERMGMGLYVKYDGDPWADSDALAFSGVMVSMKEPGWYSFGFEIKDKKCDVPCIGIEMVHDGGKETWHSAATAIYCLMGSGQLLWDTVTGVDMAL |
| B/New York/1213/2009 | 2009 | LPSTIQTLTLFLTSGGVLLSLYVSASLSYLLYSDILLKFSPTEITAPTMPLDCANASNVQAVNRSATKGVTLLLPEPEWTYPRLSCPGSTFQKALLISPHRFGETKGNSAPLIIREPFIACGPNECKHFALTHYAAQPGGYYNGTRGDRNKLRHLISVKLGKIPTVENSIFHMAAWSGSACHDGKEWTYIGVDGPDNNALLKVKYGEAYTDTYHSYANKILRTQESACNCIGGNCYLMITDGSASGVSECRFLKIREGRIIKEIFPTGRVKHTEECTCGFASNKTIECACRDNSYTAKRPFVKLNVETDAAEIRLMCTDTYLDTPRPNDGSITGPCESNGDKGSGGIKGGFVHQRMESKIGRWYSRTMSKTERMGMGLYVKYDGDPWADSDALAFSGVMVSMKEPGWYSFGFEIKDKKCDVPCIGIEMVHDGGKETWHSAATAIYCLMGSGQLLWDTVTGVDMAL |
| B/New York/1217/2009 | 2009 | LPSTIQTLTLFLTSGGVLLSLYVSASLSYLLYSDILLKFSPTEITAPTMPLDCANASNVQAVNRSATKGVTLLLPEPEWTYPRLSCPGSTFQKALLISPHRFGETKGNSAPLIIREPFIACGPNECKHFALTHYAAQPGGYYNGTRGDRNKLRHLISVKLGKIPTVENSIFHMAAWSGSACHDGKEWTYIGVDGPDNNALLKVKYGEAYTDTYHSYANKILRTQESACNCIGGNCYLMITDGSASGVSECRFLKIREGRIIKEIFPTGRVKHTEECTCGFASNKTIECACRDNSYTAKRPFVKLNVETDTAEIRLMCTDTYLDTPRPNDGSITGPCESNGDKGSGGIKGGFVHQRMESKIGRWYSRTMSKTERMGMELYVKYDGDPWADSDALAFSGVMVSMKEPGWYSFGFEIKDKKCDVPCIGIEMVHDGGKETWHSAATAIYCLMGSGQLLWDTVTGVNMAL |
| B/New York/1218/2009 | 2009 | LPSTIQTLTLFLTSGGVLLSLYVSASLSYLLYSDILLKFSPTEITAPTMPLDCANASNVQAVNRSATKGVTLLLPEPEWTYPRLSCPGSTFQKALLISPHRFGETKGNSAPLIIREPFIACGPNECKHFALTHYAAQPGGYYNGTRGDRNKLRHLISVKLGKIPTVENSIFHMAAWSGSACHDGKEWTYIGVDGPDNNALLKVKYGEAYTDTYHSYANKILRTQESACNCIGGNCYLMITDGSASGVSECRFLKIREGRIIKEIFPTGRVKHTEECTCGFASNKTIECACRDNSYTAKRPFVKLNVETDTAEIRLMCTDTYLDTPRPNDGSITGPCESNGDKGSGGIKGGFVHQRMESKIGRWYSRTMSKTERMGMGLYVKYDGDPWADSDALAFSGVMVSMKEPGWYSFGFEIKDKKCDVPCIGIEMVHDGGKETWHSAATAIYCLMGSGQLLWDTVTGVDMAL |
| B/New York/1221/2009 | 2009 | LPSTIQTLTLFLTSGGVLLSLYVSASLSYLLYSDILLKFSPTEITAPTMPLDCANASNVQAVNRSATKGVTLLLPEPEWTYPRLSCPGSTFQKALLISPHRFGETKGNSAPLIIREPFIACGPNECKHFALTHYAAQPGGYYNGTRGDRNKLRHLISVKLGKIPTVENSIFHMAAWSGSACHDGKEWTYIGVDGPDNNALLKVKYGEAYTDTYHSYANKILRTQESACNCIGGNCYLMITDGSASGVSECRFLKIREGRIIKEIFPTGRVKHTEECTCGFASNKTIECACRDNSYTAKRPFVKLNVETDTAEIRLMCTDTYLDTPRPNDGSITGPCESNGDKGSGGIKGGFVHQRMESKIGRWYSRTMSKTERMGMGLYVKYDGDPWADSDALAFSGVMVSMKEPGWYSFGFEIKDKKCDVPCIGIEMVHDGGKETWHSAATAIYCLMGSGQLLWDTVTGVDMAL |
| B/New York/1222/2009 | 2009 | LPSTIQTLTLFLTSGGVLLSLYVSASLSYLLYSDILLKFSPTEITAPTMPLDCANASNVQAVNRSATKGVTLLLPEPEWTYPRLSCPGSTFQKALLISPHRFGETKGNSAPLIIREPFIACGPNECKHFALTHYAAQPGGYYNGTRGDRNKLRHLISVKLGKIPTVENSIFHMAAWSGSACHDGKEWTYIGVDGPDNNALLKVKYGEAYTDTYHSYANKILRTQESACNCIGGNCYLMITDGSASGVSECRFLKIREGRIIKEIFPTGRVKHTEECTCGFASNKTIECACRDNSYTAKRPFVKLNVETDTAEIRLMCTDTYLDTPRPNDGSITGPCESNGDKGSGGIKGGFVHQRMESKIGRWYSRTMSKTERMGMGLYVKYDGDPWADSDALAFSGVMVSMKEPGWYSFGFEIKDKKCDVPCIGIEMVHDGGKETWHSAATAIYCLMGSGQLLWDTVTGVDMAL |
| B/New York/1227/2009 | 2009 | LPSTIQTLTLFLTSGGVLLSLYVSASLSYLLYSDILLKFSPTEITAPTMPLDCANASNVQAVNRSATKGVTLLLPEPEWTYPRLSCPGSTFQKALLISPHRFGETKGNSAPLIIREPFIACGPNECKHFALTHYAAQPGGYYNGTRGDRNKLRHLISVKLGKIPTVENSIFHMAAWSGSACHDGKEWTYIGVDGPDNNALLKVKYGEAYTDTYHSYANKILRTQESACNCIGGNCYLMITDGSASGVSECRFLKIREGRIIKEIFPTGRVKHTEECTCGFASNKTIECACRDNSYTAKRPFVKLNVETDTAEIRLMCTDTYLDTPRPNDGSITGPCESNGDKGSGGIKGGFVHQRMESKIGRWYSRTMSKTERMGMELYVKYDGDPWADSDALAFSGVMVSMKEPGWYSFGFEIKDKKCDVPCIGIEMVHDGGKETWHSAATAIYCLMGSGQLLWDTVTGVNMAL |
| B/New York/1229/2009 | 2009 | LPSTIQTLTLFLTSGGVLLSLYVSASLSYLLYSDILLKFSPTEITAPTMPLDCANASNVQAVNRSATKGVTLLLPEPEWTYPRLSCPGSTFQKALLISPHRFGETKGNSAPLIIREPFIACGPNECKHFALTHYAAQPGGYYNGTRGDRNKLRHLISVKLGKIPTVENSIFHMAAWSGSACHDGKEWTYIGVDGPDNNALLKVKYGEAYTDTYHSYANKILRTQESACNCIGGNCYLMITDGSASGVSECRFLKIREGRIIKEIFPTGRVKHTEECTCGFASNKTIECACRDNSYTAKRPFVKLNVETDTAEIRLMCTDTYLDTPRPNDGSITGPCESNGDKGSGGIKGGFVHQRMESKIGRWYSRTMSKTERMGMGLYVKYDGDPWADSDALAFSGVMVSMKEPGWYSFGFEIKDKKCDVPCIGIEMVHDGGKETWHSAATAIYCLMGSGQLLWDTVTGVDMAL |
| B/NIIGATA/755/2009 | 2009 | LPSTIQTLTLFLTSGGVLLSLYVSASLSYLLYSDILLKFSPTEITAPTMPLDCANASNIQAVNRSATKGVTLLLPEPEWTYPRLSCPGSTFQKALLISPHRFGETKGNSAPLIIREPFIACGPNECKHFALTHYAAQPGGYYNGTRGDRNKLRHLISVKLGKIPTVENSIFHMAAWSGSACHDGKEWTYIGVDGPDNNALLKVKYGEAYTDTYHSYANKILRTQESACNCIGGNCYLMITDGSASGVSECRFLKIREGRIIKEIFPTGRVKHTEECTCGFASNKTIECACRDNSYTAKRPFVKLNVETDTAEIRLMCTDTYLDTPRPNDGSITGPCESNGDKGSGGIKGGFVHQRMESKIGRWYSRTMSKTERMGMGLYVKYDGDPWADSDALAFSGVMVSMKEPGWYSFGFEIKDKKCDVPCIGIEMVHDGGKETWHSAATAIYCLMGSGQLLWDTVTGVDMAL |
| B/Novosibirsk/715/2009 | 2009 | LPSTIQTLTLFLTSGGVLLSLYVSASLSYLLYSDILLKFSPTEITAPTMPLDCANASNVQAVNRSATKGVTLLLPEPEWTYPRLSCPGSTFQKALLISPHRFGETKGNSAPLIIREPFIACGPNECKHFALTHYAAQPGGYYNGTRGDRNKLRHLISVKLGKIPTVENSIFHMAAWSGSACHDGKEWTYIGVDGPDNNALLKVKYGEAYTDTYHSYANKILRTQESACNCIGGNCYLMITDGSASGVSECRFLKIREGRIIKEIFPTGRVKHTEECTCGFASNKTIECACRDNSYTAKRPFVKLNVETDTAEIRLMCTDTYLDTPRPNDGSITGPCESNGDKGSGGIKGGFVHQRMESKIGRWYSRTMSKTERMGMELYVKYDGDPWADSDALAFSGVMVSMKEPGWYSFGFEIKDKKCDVPCIGIEMVHDGGKETWHSAATAIYCLMGSGQLLWDTVTGVNMAL |
| B/OITA/3/2009 | 2009 | LPSTIQTLTLFLTSGGVLLSLYVSASLSYLLYSDILLKFSPTEITAPTMPLDCANASNVQAVNRSATKGVTLLLPEPEWTYPRLSCPGSTFQKALLISPHRFGETKGNSAPLIIREPFIACGPNECKHFALTHYAAQPGGYYNGTRGDRNKLRHLISVKLGKIPTVENSIFHMAAWSGSACHDGKEWTYIGVDGPDNNALLKVKYGEAYTDTYHSYANKILRTQESACNCIGGNCYLMITDGSASGVSECRFLKIREGRIIKEIFPTGRVKHTEECTCGFASNKTIECACRDNSYTAKRPFVKLNVETDTAEIRLMCTDTYLDTPRPNDGSITGPCESNGDKGSGGIKGGFVHQRMESKIGRWYSRTMSKTERMGMGLYVKYDGDPWADSDALAFSGVMVSMKEPGWYSFGFEIKDKKCDVPCIGIEMVHDGGKETWHSAATAIYCLMGSGQLLWDTVTGVDMAL |
| B/OKAYAMA/1/2009 | 2009 | LPSTIQTLTLFLTSGGVLLSLYVSASLSYLLYSDILLKFSPTEITAPTMPLDCANASNVQAVNRSATKGVTFLLPEPEWTYPRLSCPGSTFQKALLISPHRFGETKGNSAPLIIREPFIACGPNECKHFALTHYAAQPGGYYNGTRGDRNKLRHLISVKLGKIPTVENSIFHMAAWSGSACHDGKEWTYIGVDGPDNNALLKVKYGEAYTDTYHSYANKILRTQESACNCIGGNCYLMITDGSASGVSECRFLKIREGRIIKEIFPTGRVKHTEECTCGFASNKTIECACRDNSYTAKRPFVKLNVETDTAEIRLMCTDTYLDTPRPDDGSITGPCESNGDKGSGGIKGGFVHQRMESKIGRWYSRTMSKTERMGMGLYVKYDGDPWADSDALAFSGVMVSMKEPGWYSFGFEIKDKKCDVPCIGIEMVHDGGKETWHSAATAIYCLMGSGQLLWDTVTGVDMAL |
| B/Oklahoma/WRAIR1587P/2009 | 2009 | LPSTIQTLTLFLTSGGVLLSLYVSASLSYLLYSDILLKFSPTEITAPTMPLDCANASNVQAMNRSATKGVTLLLPEPEWTYPRLSCPGSTFQKALLISPHRFGETKGNSAPLIIREPFIACGPNECKHFALTHYAAQPGGYYNGTREDRNKLRHLISVKLGKIPTVENSIFHMAAWSGSACHDGREWTYIGVDGPDSNALLKIKYGEAYTDTYHSYAKNILRTQESACNCIGGDCYLMITDGPASGISECRFLKIREGRIIKEIFPTGRVKHTEECTCGFASNKTIECACRDNSYTAKRPFVKLNVETDTAEIRLMCTDTYLDTPRPDDGSITGPCESNGDKGSGGIKGGFVHQRMESKIGRWYSRTMSKTERMGMGLYVKYDGDPWADSDALAFSGVMVSMKEPGWYSFGFEIKDKKCDVPCIGIEMVHDGGKETWHSAATAIYCLMGSGQLLWDTVTGVDMAL |
| B/OSAKA/12/2009 | 2009 | LPSTIQTLTLFLTSGGVLLSLYVSASLSYLLYSDILLKFSPTEITAPTMPLDCANASNVQAVNRSATKGVTLLLPEPEWTYPRLSCPGSTFQKALLISPHRFGETKGNSAPLIIREPFIACGPNECKHFALTHYAAQPGGYYNGTRGDRNKLRHLISVKLGKIPTVENSIFHMAAWSGSACHDGKEWTYIGVDGPDNNALLKVKYGEAYTDTYHSYANKILRTQESACNCIGGNCYLMITDGSASGVSECRFLKIREGRIIKEIFPTGRVKHTEECTCGFASNKTIECACRDNSYTAKRPFVKLNVETDTAEIRLMCTDTYLDTPRPNDGSITGPCESNGDKGSGGIKGGFVHQRMESKIGRWYSRTMSKTERMGMGLYVKYDGDPWADSDALAFSGVMVSMKEPGWYSFGFEIKDKKCDVPCIGIEMVHDGGKETWHSAATAIYCLMGSGQLLWDTVTGVDMAL |
| B/Philippines/6075/2009 | 2009 | LPSTIQMLTLFLTSGGVLLSLYVSASLSYLLYSDILLKFPPTEITAPTMPLDCANASNVQAVNRSATKGVTLLLPEPEWTYPRLSCPGSTFQKALLISPHRFGETKGNSAPLIIREPFIACGPKECKHFALTHYAAQPGGYYNGTRGDRNKLRHLISVKLGKIPTVENSIFHMAAWSGSACHDGKEWTYIGVDGPDNNALLKIKYGEAYTDTYHSYANNILRTQESACNCIGGNCYLMITDGSASGVSECRFLKIREGRIIKEIFPTGRVKHTEECTCGFASNKTIECACRDNSYTAKRPFVKLNVETDTAEIRLMCTETYLDTPRPDDGSITGPCESNGDKGSGGIKGGFVHQRMASKIGRWYSRTMSKTERMGMGLYIKYDGDPWADSDALAFSGVMVSMEEPGWYSFGFEIKDKKCDVPCIGIEMVHDGGKETWHSAATAIYCLMGSGQLLWDTVTGVNMAL |
| B/Philippines/6087/2009 | 2009 | LPSTIQMLTLFLTSGGVLLSLYVSASLSYLLYSDILLKFPPTEITAPTMPLDCANASNVQAVNRSATKGVTLLLPEPEWTYPRLSCPGSTFQKALLISPHRFGETKGNSAPLIIREPFIACGPKECKHFALTHYAAQPGGYYNGTRGDRNKLRHLISVKLGKIPTVENSIFHMAAWSGSACHDGKEWTYIGVDGPDNNALLKIKYGEAYTDTYHSYANNILRTQESACNCIGGNCYLMITDGSASGVSECRFLKIREGRIIKEIFPTGRVKHTEECTCGFASNKTIECACRDNSYTAKRPFVKLNVETDTAEIRLMCTETYLDTPRPDDGSITGPCESNGDKGSGGIKGGFVHQRMASKIGRWYSRTMSKTERMGMGLYIKYDGDPWADSDALAFSGVMISMEEPGWYSFGFEIKDKKCDVPCIGIEMVHDGGKETWHSAATAIYCLMGSGQLLWDTVTGVNMAL |
| B/SAITAMA-C/12/2009 | 2009 | LPSTIQTLTLFLTSGGVLLSLYVSASLSYLLYSDILLKFSPTEITAPTMPLDCANASNVQAVNRSATKGVTLLLPEPEWTYPRLSCPGSTFQKALLISPHRFGETKGNSAPLIIREPFIACGPNECKHFALTHYAAQPGGYYNGTRGDRNKLRHLISVKLGKIPTVENSIFHMAAWSGSACHDGKEWTYIGVDGPDNNALLKVKYGEAYTDTYHSYANKILRTQESACNCIGGNCYLMITDGSASGVSECRFLKIREGRIIKEIFPTGRVKHTEECTCGFASNKTIECACRDNSYTAKRPFVKLNVETDTAEIRLMCTDTYLDTPRPNDGSITGPCESNGDKGSGGIKGGFVHQRMESKIGRWYSRTMSKTERMGMGLYVKYDGDPWADSDALAFSGVMVSMKEPGWYSFGFEIKDKKCDVPCIGIEMVHDGGKETWHSAATAIYCLMGSGQLLWDTVTGVDMAL |
| B/Shanghai-nanhui/1101/2009 | 2009 | LPSTIQTLTLFLTSGGVLLSLYVSASLSYLLYSDILLKFPSTEITAPTMPLDCANASNVQAVNRSATKEVTLLLPEPEWTYPRLSCPGSTFQKALLISPHRFGETKGNSAPLIIREPFIACGPKECKHFALTHYAAQPGGYYNGTRGDRNKLRHLISVRLGKIPTVENSIFHMAAWSGSACHDGKEWTYIGVDGPDNNALLKIKYGEAYTDTYHSYANNILRTQESACNCIGGNCYLMITDGSASGVSECRFLKIREGRIIKEIFPTGRIKHTEECTCGFASNKTIECACRDNSYTAKRPFVKLNVETDTAEIRLMCTETYLDTPRPDDGSITGPCESNGGKGSGGIKGGFVHQRMASKIGRWYSRTMSKTERKGMGLYVKYDGDPWADSDALAFSGVMVSMEEPGWYSFGFEIKDKKCDVPCIGIEMVHDGGKETWHSAATAIYCLMGSGQLLWDTVTGVNMAL |
| B/SHIZUOKA/133/2009 | 2009 | LPSTIQTLTLFLTSGGVLLSLYVSASLSYLLYSDILLKFSPTEITAPTMPLDCANASNVQAVNRSATKGVTLLLPEPEWTYPRLSCPGSTFQKALLISPHRFGETKGNSAPLIIREPFIACGPNECKHFALTHYAAQPGGYYNGTRGDRNKLRHLISVKLGKIPTVENSIFHMAAWSGSACHDGKEWTYIGVDGPDNNALLKVKYGEAYTDTYHSYANKILRTQESACNCIGGNCYLMITDGSASGVSECRFLKIREGRIIKEIFPTGRVKHTEECTCGFASNKTIECACRDNSYTAKRPFVKLNVETDTAEIRLMCTDTYLDTPRPNDGSITGPCESNGDKGSGGIKGGFVHQRMESKIGRWYSRTMSKTERMGMGLYVKYDGDPWADSDALAFSGVMVSMKEPGWYSFGFEIKDKKCDVPCIGIEMVHDGGKETWHSAATAIYCLMGSGQLLWDTVTGVDMAL |
| B/Sydney/1/2009 | 2009 | LPSTIQTLTLFLTSGGVLLSLYVSASLSYLLYSDILLKFSPTEITAPTMPLDCANASNVQAVNRSATKGVTLLLPEPEWTYPRLSCPGSTFQKALLISPHRFGETKGNSAPLIIREPFIACGPNECKHFALTHYAAQPGGYYNGTRGDRNKLRHLISVKLGKIPTVENSIFHMAAWSGSACHDGKEWTYIGVDGPDNNALLKVKYGEAYTDTYHSYANKILRTQESACNCIGGNCYLMITDGSASGVSECRFLKIREGRIIKEIFPTGRVKHTEECTCGFASNKTIECACRDNSYTAKRPFVKLNVETDTAEIRLMCTDTYLDTPRPNDGSITGPCESNGDKGSGGIKGGFVHQRMESKIGRWYSRTMSKTERMGMGLYVKYDGDPWADSDALAFSGVMVSMKEPGWYSFGFEIKDKKCDVPCIGIEMVHDGGKETWHSAATAIYCLMGSGQLLWDTVTGVDMAL |
| B/Sydney/4/2009 | 2009 | LPSTIQTLTLFLTSGGVLLSLYVSASLSYLLYSDILLKFSPTEITAPTMPLDCANASNVQAVNRSATKGVTLLLPEPEWTYPRLSCPGSTFQKALLISPHRFGETKGNSAPLIIREPFIACGPNECKHFALTHYAAQPGGYYNGTRGDRNKLRHLISVKLGKIPTVENSIFHMAAWSGSACHDGKEWTYIGVDGPDNNALLKVKYGEAYTDTYHSYANKILRTQESACNCIGGNCYLMITDGSASGVSECRFLKIREGRIIKEIFPTGRVKHTEECTCGFASNKTIECACRDNSYTAKRPFVKLNVETDTAEIRLMCTDTYLDTPRPNDGSITGPCESNGDKGSGGIKGGFVHQRMESKIGRWYSRTMSKTERMGMGLYVKYDGDPWADSDALAFSGVMVSMKEPGWYSFGFEIKDKKCDVPCIGIEMVHDGGKETWHSAATAIYCLMGSGQLLWDTVTGVDMAL |
| B/Taiwan/70094/2009 | 2009 | LPSTIQTLTLFLTSGGVLLSLYVSASLSYLLYSDILLKFSRTEITAPIMPLDCANASNVQAVNRSATKGVTLLLPEPEWTYPRLSCPGSTFQKALLISPHRFGETKGNSAPLIIREPFIACGPKECKHFALTHYAAQPGGYYNATREDRNKLRHLISVKLGKIPTVENSIFHMAAWSGSACHDGREWTYIGVDGPDSNALLKIKYGEAYTDTYHSYAKNILRTQESACNCIGGDCYLMITDGPASGISECRFLKIREGRIIKEIFPTGRVKHTEECTCGFASNKTIECACRDNSYTAKRPFVKLNVETDTAEIRLMCTETYLDTPRPNDGSITGPCESNGDKGSGGIKGGFVHQRMASKIGRWYSRTMSKTKRMGMGLYVKYDGDPWTDSEALTLSGVMVSMEEPGWYSFGFEIKDKKCDVPCIGIEMVHDGGKTTWHSAATAIYCLMGSGQLLWDTVTGVNMTL |
| B/Taiwan/70231/2009 | 2009 | LPSTIQTLTLFLTSGGVLLSLYVSASLSYLLYSDILLKFSRTEITAPIMPLDCANASNVQAVNRSATKGVTLLLPEPEWTYPRLSCPGSTFQKALLISPHRFGETKGNSAPLIIREPFIACGPKECKHFALTHYAAQPGGYYNGTREDRNKLRHLISVKLGKIPTVENSIFHMAAWSGSACHDGREWTYIGVDGPDSNALLKIKYGEAYTDTYHSYAKNILRTQESACNCIGGDCYLMITDGPASGISECRFLKIREGRIIKEIFPTGRVKHTEECTCGFASNKTIECACRDNSYTAKRPFVKLNVETDTAEIRLMCTETYLDTPRPNDGSITGPCESNGDKGSGGIKGGFVHQRMASKIGRWYSRTMSKTKRMGMGLYVKYDGDPWTDSEALALSGVMVSMEEPGWYSFGFEIKDKKCDVPCIGIEMVHDGGKTTWHSAATAIYCLMGSGQLLWDTVTGVNMTL |
| B/TOTTORI/65/2009 | 2009 | LPSTIQTLTLFLTSGGVLLSLYVSASLSYLLYSDILLKFSPTEITAPTMPLDCANASNVQAVNRSATKGVTLLLPEPEWTYPRLSCPGSAFQKALLISPHRFGETKGNSAPLIIREPFIACGPNECKHFALTHYAAQPGGYYNGTRGDRNKLRHLISVKLGKIPTVENSIFHMAAWSGSACHDGKEWTYIGVDGPDNNALLKVKYGEAYTDTYHSYANKILRTQESACNCIGGNCYLMITDGSASGVSECRFLKIREGRIIKEIFPTGRVKHTEECTCGFASNKTIECACRDNSYTAKRPFVKLNVETDTAEIRLMCTDTYLDTPRPNDGSITGPCESNGDKGSGGIKGGFVHQRMESKIGRWYSRTMSKTERMGMGLYVKYDGDPWADSDALAFSGVMVSMKEPGWYSFGFEIKDKKCDVPCIGIEMVHDGGKETWHSAATAIYCLMGSGQLLWDTVTGVDMAL |
| B/TOYAMA/92/2009 | 2009 | LPSTIQMLTLFLTSGGVLLSLYVSASLSYLLYSDILLKFPPTEITAPTMPLDCANASNVQAVNRSATKGVTLLLPEPEWTYPRLSCPGSTFQKALLISPHRFGETKGNSAPLIIREPFIACGPKECKHFALTHYAAQPGGYYNGTRGDRNKLRHLISVKLGKIPTVENSIFHMAAWSGSACHDGKEWTYIGVDGPDNNALLKIKYGEAYTDTYHSYANNILRTQESACNCIGGNCYLMITDGSASGVSECRFLKIREGRIIKEIFPTGRVKHTEECTCGFASNKTIECACRDNSYTAKRPFVKLNVETDTAEIRLMCTETYLDTPRPDDGSITGPCESNGDKGSGGIKGGFVHQRMASKIGRWYSRTMSKTERMGMGLYVKYDGDPWADSDALAFSGVMVSMEEPGWYSFGFEIKDKKCDVPCIGIEMVHDGGKETWHSAATAIYCLMGSGQLLWDTVTGVNMAL |
| B/YAMAGUCHI/4/2009 | 2009 | LPSTIQTLTLFLTSGGVLLSLYVSASLSYLLYSDILLKFSPTEITAPTMPLDCANASNVQAVNRSATKGVTLLLPEPEWTYPRLSCPGSTFQKALLISPHRFGETKGNSAPLIIREPFIACGPNECKHFALTHYAAQPGGYYNGTRGDRNKLRHLISVKLGKIPTVENSIFHMAAWSGSACHDGKEWTYIGVDGPDNNALLKVKYGEAYTDTYHSYANKILRTQESACNCIGGNCYLMITDGSASGVSECRFLKIREGRIIKEIFPTGRVKHTEECTCGFASNKTIECACRDNSYTAKRPFVKLNVETDTAEIRLMCTDTYLDTPRPNDGSITGPCESNGDKGSGGIKGGFVHQRMESKIGRWYSRTMSKTERMGMGLYVKYDGDPWADSDALAFSGVMVSMKEPGWYSFGFEIKDKKCDVPCIGIEMVHDGGKETWHSAATAIYCLMGSGQLLWDTVTGVDMAL |
| B/Beijing-Xicheng/1961/2010 | 2010 | LPSTIQTLTLFLTSGGVLLSLYVSASLSYLLYSDILLKFSRTEINAPIMPLDCANASNVQAVNRSATKGVTLLLPEPEWTYPRLSCPGSTFQKALLISPHRFGETKGNSAPLIIREPFIACGPKECKHFALTHYAAQPGGYYNGTREDRNKLRHLISVKLGKIPTVENSIFHMAAWSGSACHDGREWTYIGVDGPDSNALLKIKYGEAYTDTYHSYAKNILRTQESACNCIGGDCYLMITDGPASGISECRFLKIREGRIIKEIFPTGRVKHTEECTCGFASNKTIECACRDNSYTAKRPFVKLNVETDTAEIRLMCTETYLDTPRPNDGSITGPCESNGDKGSGGIKGGFVHQRMASKIGRWYSRTMSKTKRMGMGLYVKYDGDPWTDSEALALSGVMVSMEEPGWYSFGFEIKDKKCDVPCIGIEMVHDGGKTTWHSAATAIYCLMGSGQLLWDTVTGVNMTL |
| B/Brisbane/60-5/2010 | 2010 | LPSTIQTLTLFLTSGGVLLSLYVSASLSYLLYSDILLKFSPTEITAPTMPLDCANASNVQAVNRSATKGVTLLLPEPEWTYPRLSCPGSTFQKALLISPHRFGETKGNSAPLIIREPFIACGPNECKHFALTHYAAQPGGYYNGTRGDRNKLRHLISVKLGKIPTVENSIFHMAAWSGSACHDGKEWTYIGVDGPDNNALLKVKYGEAYTDTYHSYANKILRTQESACNCIGGNCYLMITDGSASGVSECRFLKIREGRIIKEIFPTGRVKHTEECTCGFASNKTIECACRDNSYTAKRPFVKLNVETDTAEIRLMCTDTYLDTPRPNDGSITGPCESDGDKGSGGIKGGFVHQRMESKIGRWYSRTMSKTERMGMGLYVKYDGDPWADSDALAFSGVMVSMKEPGWYSFGFEIKDKKCDVPCIGIEMVHDGGKETWHSAATAIYCLMGSGQLLWDTVTGVDMAL |
| B/CHIBA-C/52/2010 | 2010 | LPSTIQTLTLFLTSGGVLLSLYVSASLSYLLYSDILLKFSPTEITAPTMPLDCANASNVQAVNRSATKGVTLLLPEPEWTYPRLSCPGSTFQKALLISPHRFGETKGNSAPLIIREPFIACGPNECKHFALTHYAAQPGGYYNGTRGDRNKLRHLISVKLGKIPTVENSIFHMAAWSGSACHDGKEWTYIGVDGPDNNALLKVKYGEAYTDTYHSYANKILRTQESACNCIGGNCYLMITDGSASGXSECRFLKIREGRIIKEIFPTGRVKHTEECTCGFASNKTIECACRDNSYTAKRPFVKLNVETDTAEIRLMCTDTYLDTPRPNDGSITGPCESDGDKGSGGIKGGFVHQRMGSKIGRWYSRTMSKTERMGMGLYVKYDGDPWADSDALAFSGVMVSMKEPGWYSFGFEIKDKKCDVPCIGIEMVHDGGKETWHSAATAIYCLMGSGQLLWDTVTGVDMAL |
| B/Chongqing-yuzhong/1254/2010 | 2010 | LPSTIQTLTLFLTSGGVLLSLYVSASLSYLLYSDILLKFSRTEITAPIMPLDCANASNVQAVNRSATKGVTLLLPEPEWTYPRLSCPGSTFQKALLISPHRFGETKGNSAPLIIREPFIACGPKECKHFALTHYAAQPGGYYNGTREDRNKLRHLISVKLGKIPTVENSIFHMAAWSGSACHDGREWTYIGVDGPDSNALLKIKYGEAYTDTYHSYAKNILRTQESACNCIGGDCYLMITDGPASGISECRFLKIREGRIIKEIFPTGRVKHTEECTCGFASNKTIECACRDNSYTAKRPFVKLNVETDTAEIRLMCTETYLDTPRPNDGSITGPCESNGDKGSGGIKGGFVHQRMASKIGRWYSRTMSKTKRMGMGLYVKYDGDPWTDSEALALSGVMVSMEEPGWYSFGFEIKDKKCDVPCIGIEMVHDGGKTTWHSAATAIYCLMGSGQLLWDTVTGVNMTL |
| B/Chongqing-yuzhong/1260/2010 | 2010 | LPSTIQTLTLFLTSGGVLLSLYVSASLSYLLYSDILLKFSRTEITAPIMPLDCANASNVQAVNRSATKGVTLLLPEPEWTYPRLSCPGSTFQKALLISPHRFGETKGNSAPLIIREPFIACGPKECKHFALTHYAAQPGGYYNGTREDRNKLRHLISVKLGKIPTVENSIFHMAAWSGSACHDGREWTYIGVDGPDSNALLKIKYGEAYTDTYHSYAKNILRTQESACNCIGGDCYLMITDGPASGISECRFLKIREGRIIKEIFPTGRVKHTEECTCGFASNKTIECACRDNSYTAKRPFVKLNVETDTAEIRLMCTETYLDTPRPNDGSITGPCESNGDKGSGGIKGGFVHQRMASKIGRWYSRTMSKTKRMGMGLYVKYDGDPWTDSEALALSGVMVSMEEPGWYSFGFEIKDKKCDVPCIGIEMVHDGGKTTWHSAATAIYCLMGSGQLLWDTVTGVNMTL |
| B/Egypt/46/2010 | 2010 | LPSTIQTLTLFLTSGGVLLSLYVSASLSYLLYSDILLKFSPTEITAPTMPLDCANASNVQAVNRSATKGVTLLLPEPEWTYPRLSCPGSTFQKALLISPHRFGETKGNSAPLIIREPFIACGPNECKHFALTHYAAQPGGYYNGTRGDRNKLRHLISVKLGKIPTVENSIFHMAAWSGSACHDGKEWTYIGVDGPDNNALLKVKYGEAYTDTYHSYANKILRTQESACNCIGGNCYLMITDGSASGVSECRFLKIREGRIIKEIFPTGRVKHTEECTCGFASNKTIECACRDNSYTAKRPFVKLNVETDTAEIRLMCTDTYLDTPRPNDGSITGPCESNGDKGSGGIKGGFVHQRMESKIGRWYSRTMSKTERMGMGLYVKYDGDPWTDSDALAFSGVMVSMKEPGWYSFGFEIKDKKCDVPCIGIEMVHDGGKETWHSAATAIYCLMGSGQLLWDTVTGVDMAL |
| B/England/684/2010 | 2010 | LPSTIQTLTLFLTSGGVLLSLYVSALLSYLLYSDILLKFSPTEITAPTMSLDCANASNVQAVNRSATKGVTFLLPEPEWTYPRLSCPGSTFQKALLISPHRFGETKGNSAPLIIREPFIACGPNECKHFALTHYAAQPGGYYNGTRGDRNKLRHLISVKLGKIPTVENSIFHMAAWSGSACHDGKEWTYIGVDGPDNDALLKVKYGEAYTDTYHSYANKILRTQESACNCIGGNCYLMITDGSASGISECRFLKIREGRIIKEIFPTGRVKHTEECTCGFASNKTIECACRDNSYTAKRPFVKLNVETDTAEIRLMCTDTYLDTPRPDDGSITGPCESNGDKGSGGIKGGFVHQRMESKIGRWYSRTMSKTERMGMGLYVKYDGDPWADSDALAFSGVMVSMKEPGWYSFGFEIKDKKCDVPCIGIEMVHDGGKETWHSAATAIYCLMGSGQLLWDTVTGVDMAL |
| B/Fujian-Meilie/1440/2010 | 2010 | LPSTIQTLTLFLTSGGVLLSLYVSASLSYLLYSDILLKFSRTEITAPIMPLDCANASNVQAVNRSATKGVTLLLPEPEWTYPRLSCPGSTFQKALLISPHRFGETKGNSAPLIIREPFIACGPKECKHFALTHYAAQPGGYYNGTREDRNKLRHLISVKLGKIPTVENSIFHMAAWSGSACHDGREWTYIGVDGPDSNALLKIKYGEAYTDTYHSYAKNILRTQESACNCIGGDCYLMITDGPASGISECRFLKIREGRIIKEIFPTGRVKHTEECTCGFASNKTIECACRDNSYTAKRPFVKLNVETDTAEIRLMCTETYLDTPRPNDGSITGPCESNGDKGSGGIKGGFVHQRMASKIGRWYSRTMSKTKRMGMGLYVKYDGDPWTDSEALALSGVMVSMEEPGWYSFGFEIKDKKCDVPCIGIEMVHDGGKTTWHSAATAIYCLMGSGQLLWDTVTGVNMTL |
| B/FUKUSHIMA/1/2010 | 2010 | LPSTIQTLTLFLTSGGVLLSLYVSASLSYLLYSDILLKFSPTETTAPTMSLDCANASNVQAVNRSATKGVTFLLPEPEWTYPRLSCPGSTFQKALLISPHRFGETKGNSAPLIIREPFIACGPNECKHFALTHYAAQPGGYYNGTRGDRNKLRHLISVKLGKIPTVENSIFHMAAWSGSACHDGKEWTYIGVDGPDNDALLKVKYGEAYTDTYHSYANKILRTQESACNCIGGNCYLMITDGSASGVSECRFLKIREGRIIKEIFPTGRVKHTEECTCGFASNKTIECACRDNSYTAKRPFVKLNVETDTAEIRLMCTDTYLDTPRPDDGSITGPCESNGDKGSGGIKGGFVHQRMESKIGRWYSRTMSKTERMGMGLYVKYDGDPWADSDALAFSGVMVSMKEPGWYSFGFEIKDKKCDVPCIGIEMVHDGGKETWHSAATAIYCLMGSGQLLWDTVTGVDMAL |
| B/Guangdong-Dongguanbendi/1721/2010 | 2010 | LPSTIQTLTLFLTSGGVLLSLYVSASLSYLLYSDILLKFSRTEITAPIMPLDCANASNVQAVNRSATKGVTLLLPEPEWMYPRLSCPGSTFQKALLISPHRFGETKGNSAPLIIREPFIACGPKECKHFALTHYAAQPGGYYNGTREDRNKLRHLISVKLGKIPTVENSIFHMAAWSGSACHDGREWTYIGVDGPDSNALLKIKYGEAYTDTYHSYAKNILRTQESACNCIGGDCYLMITDGPASGISECRFLKIREGRIIKEIFPTGRVKHTEECTCGFASNKTIECACRDNSYTAKRPFVKLNVETDTAEIRLMCTETYLDTPRPNDGSITGPCESNGDKGSGGIKGGFVHQRMASKIGRWYSRTMSKTKRMGMGLYVKYDGDPWTDSEALALSGVMVSMEEPGWYSFGFEIKDKKCDVPCIGIEMVHDGGKTTWHSAATAIYCLMGSGQLLWDTVTGVNMTL |
| B/Guangxi-Xixiangtang/1455/2010 | 2010 | LPSTIQTLTLFLTSGGVLLSLYVSALLSYLLYLDILLKFSPTEITAPTMSLDCANASNVQAVNRSATKGVTFLLPEPEWTYPRLSCPGSTFQKALLISPHRFGETKGNSAPLIIREPFIACGPNECKHFALTHYAAQPGGYYNGTRGDRNKLRHLISVKLGKIPTVENSIFHMAAWSGSACHDGKEWTYIGVDGPDNDALLKVKYGEAYTDTYHSYANKILRTQESACNCIGGNCYLMITDGSASGVSECRFLKIREGRIIKEIFPTGRVKHTEECTCGFASNKTIECACRDNSYTAKRPFVKLNVETDTAEIRLMCTDTYLDTPRPDDGSITGPCESNGDKGSGGIKGGFVHQRMESKIGRWYSRTMSKTERMGMGLYVKYDGDPWADSDALAFSGVMVSMKEPGWYSFGFEIKDKKCDVPCIGIEMVHDGGKGTWHSAATAIYCLMGSGQLLWDTVTGVDMAL |
| B/HIROSHIMA-C/11/2010 | 2010 | LPSTIQTLTLFLTSGGVLLSLYLSALLSYLLYSDILLKFSPTEITAPTMSLDCANASNVQAVNRSATKGVTFLLPEPEWTYPRLSCPGSTFQKALLISPHRFGETKGNSAPLIIREPFIACGPNECKHFALTHYAAQPGGYYNGTRGDRNKLRHLISVKLGKIPTVENSIFHMAAWSGSACHDGKEWTYIGVDGPDNDALLKVKYGEAYTDTYHSYANKILRTQESACNCIGGNCYLMITDGSASGVSECRFLKIREGRIIKEIFPTGRVKHTEECTCGFASNKTIECACRDNSYTAKRPFVKLNVETDTAEIRLMCTDTYLDTPRPDDGSITGPCESNGDKGSGGIKGGFVHQRMESKIGRWYSRTMSKTERMGMGLYVKYDGDPWADSDALAFSGVMVSMKEPGWYSFGFEIKDKKCDVPCIGIEMVHDGGKETWHSAATAIYCLMGSGQLLWDTVTGVDMAL |
| B/HIROSHIMA-C/21/2010 | 2010 | LPSTIQTLTLFLTSGGVLLSLYVSALLSYLLYSDILLKFSPTEITAPTMSLDCANASNVQAVNRSATKGVTFLLPEPEWTYPRLSCPGSTFQKALLISPHRFGETKGNSAPLIIREPFIACGPNECKHFALTHYAAQPGGYYNGTRGDRNKLRHLISVKLGKIPTVENSIFHMAAWSGSACHDGKEWTYIGVDGPDNDALLKVKYGEAYTDTYHSYANKILRTQESACNCIGGNCYLMITDGSASGVSECRFLKIREGRIIKEIFPTGRVKHTEECTCGFASNKTIECACRDNSYTAKRPFVKLNVETDTAEIRLMCTDTYLDTPRPDDGSITGPCESNGDKGSGGIKGGFVHQRMESKIGRWYSRTMSKTERMGMGLYVKYDGDPWADSXALAFSGVMVSMKEPGWYSFGFEIKDKKCDVPCIGIEMVHDGGKETWHSAATAIYCLMGSGQLLWDTVTGVDMAL |
| B/Hubei-Wujiagang/1131/2010 | 2010 | LPSTIQTLTLLLTSGGVLLSLYVSASLSYLLYSDILLKFSRTEITAPIMPLDCANASNVQAVNRSATKGVTLLLPEPEWTYPRLSCPGSTFQKALLISPHRFGETKGNSAPLIIREPFIACGPKECKHFALTHYAAQPGGYYNGTREDRNKLRHLISVKLGKIPTVENSIFHMAAWSGSACHDGREWTYIGVDGPDSNALLKIKYGEAYTDTYHSYAKNILRTQESACNCIGGDCYLMITDGPASGISECRFLKIREGRIIKEIFPTGRVKHTEECTCGFASNKTIECACRDNSYTAKRPFVKLNVETDTAEIRLMCTETYLDTPRPNDGSITGPCESNGDKGSGGIKGGFVHQRMASKIGRWYSRTMSKTKRMGMGLYVKYDGDPWTDSEALALSGVMVSMEEPGWYSFGFEIKDKKCDVPCIGIEMVHDGGKTTWHSAATAIYCLMGSGQLLWDTVTGVNMTL |
| B/Hunan-Yuhua/1686/2010 | 2010 | LPSTIQTLTLFLTSGGVLLSLYVSALLSYLLYSDILLKFSPTEITAPTMSLDCANASNVQAVNRSATKGVTFLLPEPEWTYPRLSCPGSTFQKALLISPHRFGETKGNSAPLIIREPFIACGPNECKHFALTHYAAQPGGYYNGTRGDRNKLRHLISVKLGKIPTVENSIFHMAAWSGSACHDGKEWTYIGVDGPDNDALLKVKYGEAYTDTYHSYANKILRTQESACNCIGGNCYLMITDGSASGVSECRFLKIREGRIIKEIFPTGRVKHTEECTCGFASNKTIECACRDNSYTAKRPFVKLNVETDTAEIRLMCTDTYLDTPRPDDGSITGPCESNGDKGSGGIKGGFVHQRMESKIGRWYSRTMSKTERMGMGLYVKYDGDPWADSDALAFSGVMVSMKEPGWYSFGFEIKDKKCDVPCIGIEMVHDGGKETWHSAATAIYCLMGSGQLLWDTVTGVDMAL |
| B/La Rioja/RR6969/2010 | 2010 | LPSTIQTLTLFLTSGGVLLSLYVSASLSYLLYSDILLKFSPKEITAPTMPLDCANASNVQAVNHSATKGVTLLLPEPEWTYPRLSCPGSTFQKALLISPHRFGETKGNSAPLIIREPFIACGPNECKHFALTHYAAQPGGYYNGTRGDRNKLRHLISVKLGKIPTVENSIFHMAAWSGSACHDGKEWTYIGVDGPDNNALLKVKYGEAYTDTYHSYANKILRTQESACNCIGGNCYLMITDGSASGVSECRFLKIREGRIIKEIFPTGRVKHTEECTCGFASNKTIECACRDNSYTAKRPFVKLNVETDTAEIRLMCTDTYLDTPRPDDGSITGPCESNGDKGSGGIKGGFVHQRMESKIGRWYSRTMSKTERMGMGLYVKYDGDPWADSDALAFSGVMVSMKEPGWYSFGFEIKDKKCDVPCIGIEMVHDGGKETWHSAATAIYCLMGSGQLLWDTVTGVDMAL |
| B/Laos/I155/2010 | 2010 | LPSTIQTLTLFLTSGGVLLSLYVSASLSYLLYSDILLKFSPTEITAPTMPLDCANASNVQAVNRSATKGVALLLPEPEWTYPRLSCPGSTFQKALLISPHRFGETKGNSAPLIIREPFIACGPNECKHFALTHYAAQPGGYYNGTRGDRNKLRHLISVKLGKIPTVENSIFHMAAWSGSACHDGKEWTYIGVDGPDNNALLKVKYGEAYTDTYHSYANKILRTQESACNCIGGNCYLMITDGSASGVSECRFLKIREGRIIKEIFPTGRVKHTEECTCGFASNKTIECACRDNSYTAKRPFVKLNVETDTAEIRLMCTDTYLDTPRPNDGSITGPCESNGDKGSGGIKGGFVHQRMESKIGRWYSRTMSKTERMGMGLYVKYDGDPWADSDALAFSGVMVSMKEPGWYSFGFEIKDKKCDVPCIGIEMVHDGGKETWHSAATAIYCLMGSGQLLWDTVTGVDMAL |
| B/Laos/I452/2010 | 2010 | LPSTIQTLTLFLTSGGVLLSLYVSASLSYLLYSDILLKFSPTETTAPTMSLDCANASNVQAVNRSATKGVTFLLPEPEWTYPRLSCPGSTFQKALLISPHRFGETKGNSAPLIIREPFIACGPNECKHFALTHYAAQPGGYYNGTRGDRNKLRHLISVKLGKIPTVENSIFHMAAWSGSACHDGKEWTYIGVDGPDNDALLKVKYGEAYTDTYHSYANKILRTQESACNCIGGNCYLMITDGSASGVSECRFLKIREGRIIKEIFPTGRVKYTEECTCGFASNKTIECACRDNSYTAKRPFVKLNVETDTAEIRLMCTDTYLDTPRPDDGSITGPCESNGDKGSGGIKGGFVHQRMESKIGRWYSRTMSKTERMGMGLYVKYDGDPWADSDALAFSGVMVSMKEPGWYSFGFEIKDKKCDVPCIGIEMVHDGGKETWHSAATAIYCLMGSGQLLWDTVTGVDMAL |
| B/Laos/I727/2010 | 2010 | LPSTIQTLTLFLTSGGVLLSLYVSASLSYLLYSDILLKFSPTETTAPTMSLDCANASNVQAVNRSATKGVTFLLPEPEWTYPRLSCPGSTFQKALLISPHRFGETKGNSAPLIIREPFIACGPNECKHFALTHYAAQPGGYYNGTRGDRNKLRHLISVKLGKIPTVENSIFHMAAWSGSACHDGKEWTYIGVDGPDNDALLKVKYGEAYTDTYHSYANKILRTQESACNCIGGNCYLMITDGSASGVSECRFLKIREGRIIKEIFPTGRVKHTEECTCGFASNKTIECACRDNSYTAKRPFVKLNVETDTAEIRLMCTDTYLDTPRPDDGSITGPCESNGDKGSGGIKGGFVHQRMESKIGRWYSRTMSKTERMGMGLYVKYDGDPWADSDALAFSGVMVSMKEPGWYSFGFEIKDKKCDVPCIGIEMVHDGGKETWHSAATAIYCLMGSGQLLWDTVTGVDMAL |
| B/Laos/I753/2010 | 2010 | LPSTIQTLTLFLTSGGVLLSLYVSASLSYLLYSDILLKFSPTEITAPTMPLDCANASNVQAVNRSATKGVTLLLPEPEWTYPRLSCPGSTFQKALLISPHRFGETKGNSAPLIIREPFIACGPNECKHFALTHYAAQPGGYYNGTRGDRNKLRHLISVKLGKIPTVENSIFHMAAWSGSACHDGKEWTYIGVDGPDNNALLKVKYGEAYTDTYHSYANKILRTQESACNCIGGNCYLMITDGSASGVSECRFLKIREGRIIKEIFPTGRVKHTEECTCGFASNKTIECACRDNSYTAKRPFVKLNVETDTAEIRLMCTDTYLDTPRPDDGSITGPCESNGDKGSGGIKGGFVHQRMESKIGRWYSRTMSKTERMGMGLYVKYDGDPWADSDALTFSGVMVSMKEPGWYSFGFEIKDKKCDVPCIGIEMVHDGGKETWHSAATAIYCLMGSGQLLWDTVTGVDMAL |
| B/Laos/I763/2010 | 2010 | LPSTIQTLTLFLTSGGVLLSLYVSASLSYLLYSDILLKFSPTETTAPTMSLDCANASNVQAVNRSATKGVTFLLPEPEWTYPRLSCPGSTFQKALLISPHRFGETKGNSAPLIIREPFIACGPNECKHFALTHYAAQPGGYYNGTRGDRNKLRHLISVKLGKIPTVENSIFHMAAWSGSACHDGKEWTYIGVDGPDNDALLKVKYGEAYTDTYHSYANKILRTQESACNCIGGNCYLMITDGSASGVSECRFLKIREGRIIKEIFPTGRVKHTEECTCGFASNKTIECACRDNSYTAKRPFVKLNVETDTAEIRLMCTDTYLDTPRPDDGSITGPCESNGDKGSGGIKGGFVHQRMESKIGRWYSRTMSKTERMGMGLYVKYDGDPWADSDALAFSGVMVSMKEPGWYSFGFEIKDKKCDVPCIGIEMVHDGGKETWHSAATAIYCLMGSGQLLWDTVTGVDMAL |
| B/Latvia/12-41046/2010 | 2010 | LPSTIQTLTLFLTSGGVLLSLYVSASLSYLLYSDILLKFSPTEITAPTMPLDCANASNVQAVNRSATKGVTLLLPEPEWTYPRLSCPGSTFQKALLISPHRFGETKGNSAPLIIREPFIACGPNECKHFALTHYAAQPGGYYNGTRGDRNKLRHLISVKLGKIPTVENSIFHMAAWSGSACHDGKEWTYIGVDGPDNNALLKVKYGEAYTDTYHSYANKILRTQESACNCIGGNCYLMVTDGSASGVSECRFLKIREGRIIKEIFPTGRVKHTEECTCGFASNKTIECACRDNSYTAKRPFVKLNVETDTAEIRLMCTDTYLDTPRPDDGSITGPCESNGDKGSGGIKGGFVHQRMESKIGRWYSRTMSKTERMGMGLYVKYDGDPWADSDALAFSGVMVSMKEPGWYSFGFEIKDKKCDVPCIGIEMVHDGGKETWHSAATAIYCLMGSGQLLWDTVTGVDMAL |
| B/Latvia/12-43110p/2010 | 2010 | LPSTIQTLTLFLTSGGVLLSLYVSASLSYLLYSDILLKFSPTEITAPAMPLDCANASNVQAVNRSATKGVTLLLPEPEWTYPRLSCPGSTFQKALLISPHRFGETKGNSAPLIIREPFIACGPNECKHFALTHYAAQPGGYYNGTRGDRNKLRHLISVKLGKIPTVENSIFHMAAWSGSACHDGKEWTYIGVDGPDNNALLKVKYGEAYTDTYHSYANKILRTQESACNCIGGNCYLMITDGSASGVSECRFLKIREGRIIKEIFPTGRVKHTEECTCGFASNKTIECACRDNSYTAKRPFVKLNVETDTAEIRLMCTDTYLDTPRPNDGSITGPCESDGDKGSGGIKGGFVHQRMESKIGRWYSRTMSKTERMGMGLYVKYDGDPWADSDALAFSGVMVSMKEPGWYSFGFEIKDKKCDVPCIGIEMVHDGGKETWHSAATAIYCLMGSGQLLWDTVTGVDMAL |
| B/Lisboa/13/2010 | 2010 | LPSTIQTLTLFLTSGGVLLSLYVSASLSYLLYSDILLKFSPTEITAPTMPLDCANASNVQAVNRSATKGVTFLLPEPEWTYPRLSCPGSTFQKALLISPHRFGETKGNSAPLIIREPFIACGPNECKHFALTHYAAQPGGYYNGTRGDRNKLRHLISVKLGKIPTVENSIFHMAAWSGSACHDGKEWTYIGVDGPDNNALLKVKYGEAYTDTYHSYANKILRTQESACNCIGGNCYLMVTDGSASGVSECRFLKIREGRIIKEIFPTGRVKHTEECTCGFASNKTIECACRDNSYTAKRPFVKLNVETDTAEIRLMCTDTYLDTPRPDDGSITGPCESNGDKGSGGIKGGFVHQRMESKIGRWYSRTMSKTERMGMGLYVKYDGDPWADSDALAFSGVMVSMKEPGWYSFGFEIKDKKCDVPCIGIEMVHDGGKETWHSAATAIYCLMGSGQLLWDTVTGVDMAL |
| B/Madagascar/7766/2010 | 2010 | LPSTIQTLTLFLTSGGVLLSLYVSASLSYLLYSDILLKFSPTEITAPTMPLDCANASNVQAVNRSATKGVTLLLPEPEWTYPRLSCPGSTFQKALLISPHRFGETKGNSAPLIIREPFIACGPNECKHFALTHYAAQPGGYYNGTRGDRNKLRHLISVKLGKIPTVENSIFHMAAWSGSACHDGKEWTYIGVDGPDNNALLKVKYGEAYTDTYHSYANKILRTQESACNCIGGNCYLMITDGSASGVSECRFLKIREGRIIKEIFPTGRVKHTEECTCGFASNKTIECACRDNSYTAKRPFVKLNVETDTAEIRLMCTDTYLDTPRPNDGSITGPCESNGDKGSGGIKGGFVHQRMESKIGRWYSRTMSKTERMGMGLYVKYDGDPWADSDALAFSGVMVSMKEPGWYSFGFEIKDKKCDVPCIGIEMVHDGGKETWHSAATAIYCLMGSGQLLWDTVTGVDMAL |
| B/Malaysia/3023819/2010 | 2010 | LPSTIQTLTLFLTSGGVLLSLYVSASLSYLLYSDILLKFSPTEITAPTMPLDCANASNVQAVNRSATKGVTLLLPEPEWTYPRLSCPGSTFQKALLISPHRFGETKGNSAPLIIREPFIACGPNECKHFALTHYAAQPGGYYNGTRGDRNKLRHLISVKLGKIPTVENSIFHMAAWSGSACHDGKEWTYIGVDGPDNNALLKVKYGEAYTDTYHSYANKILRTQESACNCIGGNCYLMITDGSASGVSECIFLKIREGRIIKEIFPTGRVKHTEECTCGFASNKTIECACRDNSYTAKRPFVKLNVETDTAEIRLMCTDTYLDTPRPNDGSITGPCESDGDKGSGGVKGGFVHQRMESKIGRWYSRTMSKTERMGMGLYVKYDGDPWADSDALAFSGVMVSMKEPGWYSFGFEIKDKKCDVPCIGIEMVHDGGKETWHSAATAIYCLMGSGQLLWDTVTGVDMAL |
| B/Managua/2630.01/2010 | 2010 | LPSTIQTLTLFLTSGGVLLSLYVSASLSYLLYSDILLKFSPKEITAPTMPLDCANASNVQAVNHSATKGVTLLLPEPEWTYPRLSCPGSTFQKALLISPHRFGETKGNSAPLIIREPFIACGPNECKHFALTHYAAQPGGYYNGTRGDRNKLRHLISVKLGKIPTVENSIFHMAAWSGSACHDGKEWTYIGVDGPDNNALLKVKYGEAYTDTYHSYANKILRTQESACNCIGGNCYLMITDGSASGVSECRFLKIREGRIIKEIFPTGRVKHTEECTCGFASNKTIECACRDNSYTAKRPFVKLNVETDTAEIRLMCTDTYLDTPRPDDGSITGPCESNGDKGSGGIKGGFVHQRMESKIGRWYSRTMSKTERMGMGLYVKYDGDPWADSDALAFSGVMVSMKEPGWYSFGFEIKDKKCDVPCIGIEMVHDGGKETWHSAATAIYCLMGSGQLLWDTVTGVDMAL |
| B/Managua/4015.05/2010 | 2010 | LPSTIQTLTLFLTSGGVLLSLYVSASLSYLLYSDILLKFSPKEITAPTMPLDCANASNVQAVNHSATKGVTLLLPEPEWTYPRLSCPGSTFQKALLISPHRFGETKGNSAPLIIREPFIACGPNECKHFALTHYAAQPGGYYNGTRGDRNKLRHLISVKLGKIPTVENSIFHMAAWSGSACHDGKEWTYIGVDGPDNNALLKVKYGEAYTDTYHSYANKILRTQESACNCIGGNCYLMITDGSASGVSECRFLKIREGRIIKEIFPTGRVKHTEECTCGFASNKTIECACRDNSYTAKRPFVKLNVETDTAEIRLMCTDTYLDTPRPDDGSITGPCESNGDKGSGGIKGGFVHQRMESKIGRWYSRTMSKTERMGMGLYVKYDGDPWADSDALAFSGVMVSMKEPGWYSFGFEIKDKKCDVPCIGIEMVHDGGKETWHSAATAIYCLMGSGQLLWDTVTGVDMAL |
| B/Managua/4468.03/2010 | 2010 | LPSTIQTLTLFLTSGGVLLSLYVSASLSYLLYSDILLKFSPKEITAPTMPLDCANASNVQAVNHSATKGVTLLLPEPEWTYPRLSCPGSTFQKALLISPHRFGETKGNSAPLIIREPFIACGPNECKHFALTHYAAQPGGYYNGTRGDRNKLRHLISVKLGKIPTVENSIFHMAAWSGSACHDGKEWTYIGVDGPDNNALLKVKYGEAYTDTYHSYANKILRTQESACNCIGGNCYLMITDGSASGVSECRFLKIREGRIIKEIFPTGRVKHTEECTCGFASNKTIECACRDNSYTAKRPFVKLNVETDTAEIRLMCTDTYLDTPRPDDGSITGPCESNGDKGSGGIKGGFVHQRMESKIGRWYSRTMSKTERMGMGLYVKYDGDPWADSDALAFSGVMVSMKEPGWYSFGFEIKDKKCDVPCIGIEMVHDGGKETWHSAATAIYCLMGSGQLLWDTVTGVDMAL |
| B/Managua/5549.04/2010 | 2010 | LPSTIQTLTLFLTSGGVLLSLYVSASLSYLLYSDILLKFSPKEITAPTMPLDCANASNVQAVNHSATKGVTLLLPEPEWTYPRLSCPGSTFQKALLISPHRFGETKGNSAPLIIREPFIACGPNECKHFALTHYAAQPGGYYNGTRGDRNKLRHLISVKLGKIPTVENSIFHMAAWSGSACHDGKEWTYIGVDGPDNNALLKVKYGEAYTDTYHSYANKILRTQESACNCIGGNCYLMITDGSASGVSECRFLKIREGRIIKEIFPTGRVKHTEECTCGFASNKTIECACRDNSYTAKRPFVKLNVETDTAEIRLMCTDTYLDTPRPDDGSITGPCESNGDKGSGGIKGGFVHQRMESKIGRWYSRTMSKTERMGMGLYVKYDGDPWADSDALAFSGVMVSMKEPGWYSFGFEIKDKKCDVPCIGIEMVHDGGKETWHSAATAIYCLMGSGQLLWDTVTGVDMAL |
| B/New York/1235/2009 | 2010 | LPSTIQTLTLFLTSGGVLLSLYVSASLSYLLYSDILLKFSPTETTAPTMSLDCANASNVQAVNRSATKGVTFLLPEPEWTYPRLSCPGSTFQKALLISPHRFGETKGNSAPLIIREPFIACGPNECKHFALTHYAAQPGGYYNGTRGDRNKLRHLISVKLGKIPTVENSIFHMAAWSGSACHDGKEWTYIGVDGPDNDALLKVKYGEAYTDTYHSYANKILRTQESACNCIGGNCYLMITDGSASGVSECRFLKIREGRIIKEIFPTGRVKHTEECTCGFASNKTIECACRDNSYTAKRPFVKLNVETDTAEIRLMCTDTYLDTPRPDDGSITGPCESNGDKGSGGIKGGFVHQRMESKIGRWYSRTMSKTERMGMGLYVKYDGDPWADSDALAFSGVMVSMKEPGWYSFGFEIKDKKCDVPCIGIEMVHDGGKETWHSAATAIYCLMGSGQLLWDTVTGVDMAL |
| B/New York/1239/2010 | 2010 | LPSTIQTLTLFLTSGGVLLSLYVSASLSYLLYSDILLKFSRTEITAPIMPLDCANASNVQAVNRSATKGVTLLLPEPEWTYPRLSCPGSTFQKALLISPHRFGETKGNSAPLIIREPFIACGPKECKHFALTHYAAQPGGYYNGTREDRNKLRHLISVKLGKIPTVENSIFHMAAWSGSACHDGREWTYIGVDGPDSNALLKIKYGEAYTDTYHSYAKNILRTQESACNCIGGDCYLMITDGPASGISECRFLKIREGRIIKEIFPTGRVKHTEECTCGFASNKTIECACRDNSYTAKRPFVKLNVETDTAEIRLMCTETYLDTPRPNDGSITGPCESNGDKGSGGIKGGFVHQRMASKIGRWYSRTMSKTKRMGMGLYVKYDGDPWTDSEALALSGVMVSMEEPGWYSFGFEIKDKKCDVPCIGIEMVHDGGKTTWHSAATAIYCLMGSGQLLWDTVTGVNMTL |
| B/Niedersachsen/2/2010 | 2010 | LPSTIQTLTLFLTSGGVLLSLYVSASLSYLLYSDILLKFSRTEITAPIMPLDCANASNVQAVNRSATKGVTLLLPEPEWTYPRLSCPGSTFQKALLISPHRFGETKGNSAPLIIREPFIACGPKECKHFALTHYAAQPGGYYNGTREDRNKLRHLISVKLGKIPTVENSIFHMAAWSGSACHDGREWTYIGVDGPDSNALLKIKYGEAYTDTYHSYAKNILRTQESACNCIGGDCYLMITDGPASGISECRFLKIREGRIIKEIFPTGRVKHTEECTCGFASNKTIECACRDNSYTAKRPFVKLNVETDTAEIRLMCTETYLDTPRPNDGSITGPCESNGDKGSGGIKGGFVHQRMASKIGRWYSRTMSKTKRMGMGLYVKYDGDPWTDSEALALSGVMVSMEEPGWYSFGFEIKDKKCDVPCIGIEMVHDGGKTTWHSAATAIYCLMGSGQLLWDTVTGVNMTL |
| B/Odessa/3886/2010 | 2010 | LPSTIQTLTLFLTSGGVLLSLYVSALLSYLLYSDILLKFSPTEITAPTMSLDCANASNVQAVNRSATKGVTFLLPEPEWTYPRLSCPGSTFQKALLISPHRFGETKGNSAPLIIREPFIACGPNECKHFALTHYAAQPGGYYNGTRGDRNKLRHLISVKLGKIPTVENSIFHMAAWSGSACHDGKEWTYIGVDGPDNDALLKVKYGEAYTDTYHSYANKILRTQESACNCIGGNCYLMITDGSASGVSECRFLKIREGRIIKEIFPTGRVKHTEECTCGFASNKTIECACRDNSYTAKRPFVKLNVETDTAEIRLMCTDTYLDTPRPDDGSITGPCESNGDKGSGGIKGGFVHQRMESKIGRWYSRTMSKTERMGMGLYVKYDGDPWADSDALAFSGVMVSMKEPGWYSFGFEIKDKKCDVPCIGIEMVHDGGKETWHSAATAIYCLMGSGQLLWDTVTGVDMAL |
| B/OKAYAMA/1/2010 | 2010 | LPSTIQTLTLFLTSGGVLLSLYVSALLSYLLYSDILLKFSPTEITAPTMSLDCANASNVQAVNRSATKGVTFLLPEPEWTYPRLSCPGSTFQKALLISPHRFGETKGNSAPLIIREPFIACGPNECKHFALTHYAAQPGGYYNGTRGDRNKLRHLISVKLGKIPTVENSIFHMAAWSGSACHDGKEWTYIGVDGPDNDALLKVKYGEAYTDTYHSYANKILRTQESACNCIGGNCYLMITDGSASGVSECRFLKIREGRIIKEIFPTGRVKHTEECTCGFASNKTIECACRDNSYTAKRPFVKLNVETDTAEIRLMCTDTYLDTPRPDDGSITGPCESNGDKGSGGIKGGFVHQRMESKIGRWYSRTMSKTERMGMGLYVKYDGDPWADSDALAFSGVMVSMKEPGWYSFGFEIKNKKCDVPCIGIEMVHDGGKETWHSAATAIYCLMGSGQLLWDTVTGVDMAL |
| B/PERTH/362/2010 | 2010 | LPSTIQTLTLFLTSGGVLLSLYVSALLSYLLYSDILLKFSPTEITAPTMSLDCANASNVQAVNRSATKGVTFLLPEPEWTYPRLSCPGSTFQKALLISPHRFGETKGNSAPLIIREPFIACGPNECKHFALTHYAAQPGGYYNGTRGDRNKLRHLISVKLGKIPTVENSIFHMAAWSGSACHDGKEWTYIGVDGPDNDALLKVKYGEAYTDTYHSYANKILRTQESACNCIGGNCYLMITDGSASGVSECRFLKIREGRIIKEIFPTGRVKHTEECTCGFASNKTIECACRDNSYTAKRPFVKLNVETDTAEIRLMCTDTYLDTPRPDDGSITGPCESNGDKGSGGIKGGFVHQRMESKIGRWYSRTMSKTERMGMGLYVKYDGDPWADSDALAFSGVMVSMKEPGWYSFGFEIKDKKCDVPCIGIEMVHDGGKETWHSAATAIYCLMGSGQLLWDTVTGVDMAL |
| B/PERTH/81/2010 | 2010 | LPSTIQTLTLFLTSGGVLLSLYVSASLSYLLYSDILLKFSRTEITAPIMPLDCANASNVQAVNRSATKGVTPLLPEPEWTYPRLSCPGSTFQKALLISPHRFGETKGNSAPLIIREPFIACGPKECKHFALTHYAAQPGGYYNGTREDRNKLRHLISVKLGKIPTVENSIFHMAAWSGSACHDGREWTYIGVDGPDSNALLKIKYGEAYTDTYHSYAKNILRTQESACNCIGGDCYLMITDGPASGISECRFLKIREGRIIKEIFPTGRVKHTEECTCGFASNKTIECACRDNSYTAKRPFVKLNVETDTAEIRLMCTETYLDTPRPNDGSITGPCESNGDEGSGGIKGGFVHQRMASKIGRWYSRTMSKTKRMGMGLYVKYDGDPWTDSEALALSGVMVSMEEPGWYSFGFEIKDKKCDVPCIGIEMVHDGGKTTWHSAATAIYCLMGSGQLLWDTVTGVNMTL |
| B/PHILIPPINES/2576/2010 | 2010 | LPSTIQMLTLFLTSGGVLLSLYVSASLSYLLYSDILLKFPPTEITAPTMPLDCANASNVQAVNRSATKGVTLLLPEPEWTYPRLSCPGSTFQKALLISPHRFGETKGNSAPLIIREPFIACGPKECKHFALTHYAAQPGGYYNGTRGDRNKLRHLISVKLGKIPTVENSIFHMAAWSGSACHDGKEWTYIGVDGPDNNALLKIKYGEAYTDTYHSYANNILRTQESACNCIGGNCYLMITDGSASGVSECRFLKIREGRIIKEIFPTGRVKHTEECTCGFASNKTIECACRDNSYTAKRPFVKLNVETDTAEIRLMCTETYLDTPRPDDGSITGPCESNGDKGSGGIKGGFVHQRMASKIGRWYSRTMSKTERMGMGLYIKYDGDPWADSDALAFSGVMVSMEEPGWYSFGFEIKDKKCDVPCIGIEMVHDGGKETWHSAATAIYCLMGSGQLLWDTVTGVNMAL |
| B/SAPPORO/5/2010 | 2010 | LPSTIQTLTLFLTSGGVLLSLYVSASLSYLLYSDILLKFSPTETTAPTMSLDCANASNVQAVNRSATKGVTFLLPEPEWTYPRLSCPGSTFQKALLISPHRFGETKGNSAPLIIREPFIACGPNECKHFALTHYAAQPGGYYNGTRGDRNKLRHLISVKLGKIPTVENSIFHMAAWSGSACHDGKEWTYIGVDGPDNDALLKVKYGEAYTDTYHSYANKILRTQESACNCIGGNCYLMITDGSASGVSECRFLKIREGRIIKEIFPTGRVKHTEECTCGFASNKTIECACRDNSYTAKRPFVKLNVETDTAEIRLMCTDTYLDTPRPDDGSITGPCESNGDKGSGGIKGGFVHQRMESKIGRWYSRTMSKTERMGMGLYVKYDGDPWADSDALAFSGVMVSMKEPGWYSFGFEIKDKKCDVPCIGIEMVHDGGKEXWHSAATAIYCLMGSGQLLWDTVTGVDMAL |
| B/Shanghai-Pudongxin/1441/2010 | 2010 | LPSTIQTLTLFLTSGGVLLSLYVSALLSYLLYLDILLKFSPTEITAPTMSLDCANASNVQAVNRSATKGVTFLLPEPEWTYPRLSCPGSTFQKALLISPHRFGETKGNSAPLIIREPFIACGPNECKHFALTHYAAQPGGYYNGTRGDRNKLRHLISVKLGKIPTVENSIFHMAAWSGSACHDGKEWTYIGVDGPDNDALLKVKYGEAYTDTYHSYANKILRTQESACNCIGGNCYLMITDGSASGVSECRFLKIREGRIIKEIFPTGRVKHTEECTCGFASNKTIECACRDNSYTAKRPFVKLNVETDTAEIRLMCTDTYLDTPRPDDGSITGPCESNGDKGSGGIKGGFVHQRMESKIGRWYSRTMSKTERMGMGLYVKYDGDPWADSDALAFSGVMVSMKEPGWYSFGFEIKDKKCDVPCIGIEMVHDGGKETWHSAATAIYCLMGSGQLLWDTVTGVDMAL |
| B/Stockholm/9/2010 | 2010 | LPSTIQTLTLFLTSGGVLLSLYVSASLSYLLYSDILLKFSTTEITAPTMPLDCANASNVQAVNRSATKGVTLLLPEPEWTYPRLSCPGSTFQKALLISPHRFGENKGNSAPLIIREPFIACGPNECKHFALTHYAAQPGGYYNGTRGDRNKLRHLISVKLGKIPTVENSIFHMAAWSGSACHDGKEWTYIGVDGPDNNALLKVKYGEAYTDTYHSYANKILRTQESACNCIGGNCYLMITDGSASGVSECRFLKIREGRIIKEIFPTGRVKHTEECTCGFASNKTIECACRDNSYTAKRPFVKLNVETDTAEIRLMCTDTYLDTPRPNDGSITGPCESDGDKGSGGIKGGFVHQRMESKIGRWYSRTMSKTERMGMGLYVKYDGDPWADSDALAFSGVMVSMKEPGWYSFGFEIKDKKCDVPCIGIEMVHDGGKETWHSAATAIYCLMGSGQLLWDTVTGVDMAL |
| B/Sydney/2/2010 | 2010 | LPSTIQTLTLFLTSGGVLLSLYVSALLSYLLYSDILLKFSPTEITAPTMSLDCANASNVQAVNRSATKGVTFLLPEPEWTYPRLSCPGSTFQKALLISPHRFGETKGNSAPLIIREPFIACGPNECKHFALTHYAAQPGGYYNGTRGDRNKLRHLISVKLGKIPTVENSIFHMAAWSGSACHDGKEWTYIGVDGPDNDALLKVKYGEAYTDTYHSYANKILRTQESACNCIGGNCYLMITDGSASGVSECRFLKIREGRIIKEIFPTGRVKHTEECTCGFASNKTIECACRDNSYTAKRPFVKLNVETDTAEIRLMCTDTYLDTPRPDDGSITGPCESNGDKGSGGIKGGFVHQRMESKIGRWYSRTMSKTERMGMGLYVKYDGDPWADSDALAFSGVMVSMKEPGWYSFGFEIKDKKCDVPCIGIEMVHDGGKETWHSAATAIYCLMGSGQLLWDTVTGVDMAL |
| B/Sydney/206/2010 | 2010 | LPSTIQTLTLFLTSGGVLLSLYVSALLSYLLYSDILLKFSPTEITAPTMSLDCANASNVQAVNRSATKGVTFLLPEPEWTYPRLSCPGSTFQKALLISPHRFGETKGNSAPLIIREPFIACGPNECKHFALTHYAAQPGGYYNGTRGDRNKLRHLISVKLGKIPTVENSIFHMAAWSGSACHDGKEWTYIGVDGPDNDALLKVKYGEAYTDTYHSYANKILRTQESACNCIGGNCYLMITDGSASGVSECRFLKIREGRIIKEIFPTGRVKHTEECTCGFASNKTIECACRDNSYTAKRPFVKLNVETDTAEIRLMCTDTYLDTPRPDDGSITGPCESNGDKGSGGIKGGFVHQRMESKIGRWYSRTMSKTERMGMGLYVKYDGDPWADSDALAFSGVMVSMKEPGWYSFGFEIKDKKCDVPCIGIEMVHDGGKETWHSAATAIYCLMGSGQLLWDTVTGVDMAL |
| B/SYDNEY/210/2010 | 2010 | LPSTIQTLTLFLTSGGVLLSLYVSALLSYLLYSDILLKFSPTEITAPTMSLDCANASNVQAVNRSATKGVTFLLPEPEWTYPRLSCPGSTFQKALLISPHRFGETKGNSAPLIIREPFIACGPNECKHFALTHYAAQPGGYYNGTRGDRNKLRHLISVKLGKIPTVENSIFHMAAWSGSACHDGKEWTYIGVDGPDNDALLKVKYGEAYTDTYHSYANKILRTQESACNCIGGNCYLMITDGSASGVSECRFLKIREGRIIKEIFPTGRVKHTEECTCGFASNKTIECACRDNSYTAKRPFVKLNVETDTAEIRLMCTDTYLDTPRPDDGSITGPCESNGDKGSGGIKGGFVHQRMESKIGRWYSRTMSKTERMGMGLYVKYDGDPWADSDALAFSGVMVSMKEPGWYSFGFEIKDKKCDVPCIGIEMVHDGGKETWHSAATAIYCLMGSGQLLWDTVTGVDMAL |
| B/Sydney/503/2010 | 2010 | LPSTIQTLTLFLTSGGVLLSLYVSASLSYLLYSDILLKFSPTEITAPTMPLDCANASNVQAVNRSATKGVTLLLPEPEWTYPRLSCPGSTFQKALLISPHRFGETKGNSAPLIIREPFIACGPNECKHFALTHYAAQPGGYYNGTRGDRNKLRHLISVKLGKIPTVENSIFHMAAWSGSACHDGKEWTYIGVDGPDNNALLKVKYGEAYTDTYHSYANKILRTQESACNCIGGNCYLMITDGSASGVSECRFLKIREGRIIKEIFPTGRVKHTEECTCGFASNKTIECACRDNSYTAKRPFVKLNVETDTAEIRLMCTDTYLDTPRPNDGSITGPCESNGDKGSGGIKGGFVHQRMESRIGRWYSRTMSKTERMGMGLYVKYDGDPWTDSDALAFSGVMVSMKEPGWYSFGFEIKDKKCDVPCIGIEMVHDGGKETWHSAATAIYCLMGSGQLLWDTVTGVDMAL |
| B/Taiwan/101/2010 | 2010 | LPSTIQTLTLFLTSGGVLLSLYVSASLSYLLYSDILLKFSRTEITAPIMPLDCANASNVQAVNRSATKGVTLLLPEPEWTYPRLSCPGSTFQKALLISPHRFGETKGNSAPLIIREPFIACGPKECKHFALTHYAAQPGGYYNGTREDRNKLRHLISVKLGKIPTVENSIFHMAAWSGSACHDGREWTYIGVDGPDSNALLKIKYGEAYTDTYHSYAKNILRTQESACNCIGGDCYLMITDGPASGISECRFLKIREGRIIKEIFPTGRVKHTEECTCGFASNKTIECACRDNSYTAKRPFVKLNVETDTAEIRLMCTETYLDTPRPNDGSITGPCESNGDKGSGGIKGGFVHQRMASKIGRWYSRTMSKTKRMGMGLYVKYDGDPWTDSEALALSGVMVSMEEPGWYSFGFEIKDKKCDVPCIGIEMVHDGGKTTWHSAATAIYCLMGSGQLLWDTVTGVNMTL |
| B/Taiwan/51/2010 | 2010 | LPSTIQTLTLFLTSGGVLLSLYVSASLSYLLYSDILLKFSRTEITAPIMPLDCANASNVQAVNRSATKGVTLLLPEPEWTYPRLSCPGSTFQKALLISPHRFGETKGNSAPLIIREPFIACGPKECKHFALTHYAAQPGGYYNGTREDRNKLRHLISVKLGKIPTVENSIFHMAAWSGSACHDGREWTYIGVDGPDSNALLKIKYGEAYTDTYHSYAKNILRTQESACNCIGGDCYLMITDGPASGISECRFLKIREGRIIKEIFPTGRVKHTEECTCGFASNKTIECACRDNSYTAKRPFVKLNVETDTAEIRLMCTETYLDTPRPNDGSITGPCESNGDKGSGGIKGGFVHQRMASKIGRWYSRTMSKTKRMGMGLYVKYDGDPWTDSEALALSGVMVSMEEPGWYSFGFEIKDKKCDVPCIGIEMVHDGGKTTWHSAATAIYCLMGSGQLLWDTVTGVNMTL |
| B/Taiwan/74/2010 | 2010 | LPSTIQTLTLFLTSGGVLLSLYVSASLSYLLYSDILLKFSPTETTAPTMSLDCANASNVQAVNRSATKGVTFLLPEPEWTYPRLSCPGSTFQKALLISPHRFGETKGNSAPLIIREPFIACGPNECKHFALTHYAAQPGGYYNGTRGXRNKLRHLISVKLGKIPTVENSIFHMAAWSGSACHDGKEWTYIGVDGPDNDALLKVKYGEAYTDTYHSYANKILRTQESACNCIGGNCYLMITDGSASGVSECRFLKIREGRIIKEIFPTGRVKHTEECTCGFASNKTIECACRDNNYTAKRPFVKLNVETDTAEIRLMCTDTYLDTPRPDDGSITGPCESNGDKGSGGIKGGFVHQRMESKIGRWYSRTMSKTERMGMGLYVKYDGDPWADSDALAFSGVMVSMKEPGWYSFGFEIKDKKCDVPCIGIEMVHDGGKETWHSAATAIYCLMGSGQLLWDTVTGVNMAL |
| B/VICTORIA/507/2010 | 2010 | LPSTIQTLTLFLTSGGVLLSLYVSASLSYLLYSGILLKFSPTEITAPTMPLDCANASNVQAVNRSATKGVTFLLPEPEWTYPRLSCPGSTFQKALLISPHRFGETKGNSAPLIIREPFIACGPNECKHFALTHYAAQPGGYYNGTRGDRNKLRHLISVKLGKIPTVENSIFHMAAWSGSACHDGKEWTYIGVDGPDNNALLKVKYGEAYTDTYHSYANKILRTQESACNCIGGNCYLMITDGSASGVSECRFLKIREGRIIKEIFPTGRVKHTEECTCGFASNETIECACRDNSYTAKRPFVKLNVETDTAEIRLMCTDTYLDTPRPNDGSITGPCESNGDKGGGGIKGGFVHQRMESKIGRWYSRTMSKTERMGMGLYVKYDGDPWADSDALAFSGVMVSMKEPGWYSFGFEIKDKKCDVPCIGIEMVHDGGKETWHSAATAIYCLMGSGQLLWDTVTGVDMAL |
| B/YAMAGUCHI/2/2010 | 2010 | LPSTIQTLTLFLTSGGVLLSLYVSALLSYLLYSDILLKFSPTEITAPTMSLDCANASNVQVVNRSATKGVTFLLPEPEWTYPRLSCPGSTFQKALLISPHRFGETKGNSAPLIIREPFIACGPNECKHFALTHYAAQPGGYYNGTRGDRNKLRHLISVKLGKIPTVENSIFHMAAWSGSACHDGKEWTYIGVDGPDNDALLKVKYGEAYTDTYHSYANKILRTQESACNCIGGNCYLMITDGSASGVSECRFLKIREGRIIKEIFPTGRVKHTEECTCGFASNKTIECACRDNSYTAKRPFVKLNVETDTAEIRLMCTDTYLDTPRPDDGSITGPCESNGDKGSGGIKGGFVHQRMESKIGRWYSRTMSKTERMGMGLYVKYDGDPWADSDALAFSGVMVSMKEPGWYSFGFEIKDKKCDVPCIGIEMVHDGGKETWHSAATAIYCLMGSGQLLWDTVTGVDMAL |
| B/Agadir/156/2011 | 2011 | LPSTIQTLTLLLTSGGVLLSLYVSASLSYLLYSDILLKFSPTEITAPTMPLDCANASNVQAVNLSATKGVTLLLPEPEWTYPRLSCPGSTFQKALLISPHRFGETKGNSAPLIIREPFIACGPNECKHFALTHYAAQPGGYYNGTRGDRNKLRHLISVKLGKIPTVENSIFHMAAWSGSACHDGKEWTYIGVDGPDNNALLKVKYGEAYTDTYHSYANKILRTQESACNCIGGNCYLMITDGSASGVSECRFLKIREGRIIKEIFPTGRVKHTEECTCGFASNKTIECACRDNSYTAKRPFVKLNVETDTAEIRLMCTDTYLDTPRPNDGSITGPCESNGDKGSGGIKGGFVHQRMESKIGRWYSRTMSKTERMGMGLYVKYDGDPWADSDALAFSGVMVSMKEPGWYSFGFEIKDKKCDVPCIGIEMVHDGGKETWHSAATAIYCLMGSGQLLWDTVTGVDMAL |
| B/Anhui-Huashan/1216/2011 | 2011 | LPSTIQTLTLFLTSGGVLLSLYVSASLSYLLYSDILLKFSRTEITAPIMPLDCANASNVQAVNRSATKGVTLLLPEPEWTYPRLSCPGSTFQKALLISPHRFGETKGNSAPLIIREPFIACGPKECKHFALTHYAAQPGGYYNGTREDRNKLRHLISVKLGKIPTVENSIFHMAAWSGSACHDGREWTYIGVDGPDSNALLKIKYGEAYTDTYHSYAKNILRTQESACNCIGGDCYLMITDGPASGISECRFLKIREGRIIKEIFPTGRVKHTEECTCGFASNKTIECACRDNSYTAKRPFVKLNVETDTAEIRLMCTETYLDTPRPNDGSITGPCESNGDKGSGGIKGGFVHQRMASKIGRWYSRTMSKTKRMGMGLYVKYDGDPWTDSEALALSGVMVSMEEPGWYSFGFEIKDKKCDVPCIGIEMVHDGGKTTWHSAATAIYCLMGSGQLLWDTVTGVNMTL |
| B/Ankara/TR-21/2011 | 2011 | LPSTIQTLTLFLTSGGVLLSLYVSASLSYLLYSDILLKFSRTEITAPIMPLDCANASNVQAVNRSATKGVTLLLPEPEWTYPRLSCPGSTFQKALLISPHRFGETKGNSAPLIIREPFIACGPKECKHFALTHYAAQPGGYYNGTREDRNKLRHLISVKLGKIPTVENSIFHMAAWSGSACHDGREWTYIGVDGPDSNALLKIKYGEAYTDTYHSYAKNILRTQESACNCIGGDCYLMITDGPASGISECRFLKIREGRIIKEIFPTGRVKHTEECTCGFASNKTIECACRDNSYTAKRPFVKLNVETDTAEIRLMCTETYLDTPRPNDGSITGPCESNGDKGSGGIKGGFVHQRMASKIGRWYSRTMSKTKRMGMGLYVKYDGDPWTDSEALALSGVMVSMEEPGWYSFGFEIKDKKCDVPCIGIEMVHDGGKTTWHSAATAVYCLMGSGQLLWDTVTGVNMTL |
| B/BRISBANE/23/2011 | 2011 | LPSTIQTLTLFLTSGGVLLSLYVSALLSYLLYSDILLKFSPTEITAPTMSLDCANTSNVQAVNRSATKGVTFLLPEPEWTYPRLSCPGSTFQKALLISPHRFGETKGNSAPLIIREPFIACGPNECKHFALTHYAAQPGGYYNGTRGDRNKLRHLISVKLGKIPTVENSIFHMAAWSGSACHDGKEWTYIGVDGPDNDALLKVKYGEAYTDTYHSYANKILRTQESACNCIGGNCYLMITDGSASGVSECRFLKIREGRIIKEIFPTGRVKHTEECTCGFASNKTIECACRDNSYTAKRPFVKLNVETDTAEIRLMCTDTYLDTPRPDDGSITGPCESNGDKGSGGIKGGFVHQRMESKIGRWYSRTMSKTERMGMGLYVKYDGDPWTDSDALAFSGVMVSMKEPGWYSFGFEIKDKKCDVPCIGIEMVHDGGKETWHSAATAIYCLMGSGQLLWDTVTGVNMAL |
| B/Brisbane/6/2011 | 2011 | LPSTIQTLTLFLTSGGVLLSLYVSASLSYLLYSDILLKFSPTEITAPTMPLDCANASNVQAVNRSATKGVTLLLPEPEWTYPRLSCPGSTFQKALLISPHRFGETKGNSAPLIIREPFIACGPNECKHFALTHYAAQPGGYYNGTRGDRNKLRHLISVKLGKIPTVENSIFHMAAWSGSACHDGKEWTYIGVDGPDNNALLKVKYGEAYTDTYHSYANKILRTQESACNCIGGNCYLMITDGSASGVSECRFLKIREGRIIKEIFPTGRVKHTEECTCGFASNKTIECACRDNSYTAKRPFVKLNVETDTAEIRLMCTDTYLDTPRPNDGSITGPCESDGDKGSGGIKGGFVHQRIESRIGRWYSRTMSKTERMGMGLYVKYDGDPWADSDALAFSGVMVSMKEPGWYSFGFEIKDKKCDVPCIGIEMVHDGGKETWHSAATAIYCLMGSGQLLWDTVTGVDMAL |
| B/CAMBODIA/121/2011 | 2011 | LPSTIQTLTLFLTSGGVLLSLYVSASLSYLLYSDILLKFSPTEITAPTMPLDCANASNVQAVNRSATKGVTLLLPEPEWTYPRLSCPGSTFQKALLISPHRFGETKGNSAPLIIREPFIACGPNECKHFALTHYAAQPGGYYNGTRGDRNKLRHLISVKLGKIPTVENSIFHMAAWSGSACHDGKEWTYIGVDGPDNNALLKVKYGEAYTDTYHSYANKILRTQESACNCIGGNCYLMITDGSASGVSECRFLKIREGRIIKEIFPTGRVKHTEECTCGFASNKTIECACRDNSYTAKRPFVKLNVETDTAEIRLMCTDTYLDTPRPNDGSITGPCESNGEKGSGGIKGGFVHQRMESKIGRWYSRTMSKTERMGMGLYVKYDGDPWADSDALTFSGVMVSMKEPGWYSFGFEIKDKKCDVPCIGIEMVHDGGKETWHSAATAIYCLMGSGQLLWDTVTGVDMAL |
| B/CAMBODIA/2/2011 | 2011 | LPSTIQMLTLFLTLGGVLLSLYVSASLSYLLYLDILLKFPPTEITAPTMPLDCANASNVQAVNRSATKGVTLLLPEPEWTYPRLSCPGSTFQKALLISPHRFGETKGNSAPLIIREPFIACGPKECKHFALTHYAAQPGGYYNGTRGDRNKLRHLISVKLGKIPTVENSIFHMAAWSGSACHDGKEWTYIGVDGPDNNALLKIKYGEAFTDTYHSYANNILRTQESACNCIGGNCYLMITDGSASGVSECRFLKIREGRIIKEIFPTGRVKHTEECTCGFASNKTIECACRDNSYTAKRPFVKLNVETDTAEIRLMCTETYLDTPRPDDGSITGPCESNGDKGSGGIKGGFVHQRMASKIGRWYSRTMSKTERMGMGLYVKYDGDPWADSDALAFSGVMVSMKEPGWYSFGFEIKDKKCDVPCIGIEMVHDGGKETWHSAATAIYCLMGSGQLLWDTVTGVNMAL |
| B/CHIBA/22/2011 | 2011 | LPSTIQTLTLFLTSGGVLLSLYVSASLSYLLYSDILLKFSPTEITAPTMPLDCANASNVQAVNRSATKGVTLLLPEPEWTYPRLSCPGSTFQKALLISPHRFGETKGNSAPLIIREPFIACGPNECKHFALTHYAAQPGGYYNGTRGDRNKLRHLISVKLGKIPTVENSIFHMAAWSGSACHDGKEWTYIGVDGPDNNALLKVKYGEAYTDTYHSYANKILRTQESACNCIGGNCYLMITDGSASGVSECRFLKIREGRIIKEIFPTGRVKHTEECTCGFASNKTIECACRDNRYTAKRPFVKLNVETDTAEIRLMCTDTYLDTPRPNDGSITGPCESDGDKGSGGIKGGFVHQRMKSKIGRWYSRTMSKTERMGMGLYVKYDGDPWADSDALAFSGVMVSMKEPGWYSFGFEIKDKKCDVPCIGIEMVHDGGKETWHSAATAIYCLMGSGQLLWDTVTGVDMAL |
| B/Christchurch/25/2011 | 2011 | LPSTIQTLTLFLTSGGVLLSLYVSALLSYLLYSDILLKFSPTEITAPTMSLDCANASNVQAVNRSATKGVTFLLPEPEWTYPRLSCPGSTFQKALLISPHRFGETKGNSAPLIIREPFIACGPNECKHFALTHYAAQPGGYYNGTRGDRNKLRHLISVKLGKIPTVENSIFHMAAWSGSACHDGKEWTYIGVDGPDNDALLKVKYGEAYTDTYHSYANKILRTQESACNCIGGNCYLMITDGSASGVSECRFLKIREGRIIKEIFPTGRVKHTEECTCGFASNKTIECACRDNSYTAKRPFVKLNMETDTAEIRLMCTDTYLDTPRPDDGSITGPCESNGDKGSGGIKGGFVHQRMESKIGRWYSRTMSKTERMGMGLYVKYDGDPWADSDALAFSGVMVSMKEPGWYSFGFEIKDKKCDVPCIGIEMVHDGGKETWHSAATAIYCLMGSGQLLWDTVTGVDMAL |
| B/Donetsk/5206/2011 | 2011 | LPSTIQTLTLFLTSGGVLLSLYVSALLSYLLYSDILLKFSPTEITAPTMSLDCANASNVQAVNRSATKGVTFLLPEPEWTYPRLSCPGSTFQKALLISPHRFGETKGNSAPLIIREPFIACGPNECKHFALTHYAAQPGGYYNGTRGDRNKLRHLISVKLGKIPTVENSIFHMAAWSGSACHDGKEWTYIGVDGPDNDALLKVKYGEAYTDTYHSYANKILRTQESACNCIGGNCYLMITDGSASGVSECRFLKIREGRIIKEIFPTGRVKHTEECTCGFASNKTIECACRDNSYTAKRPFVKLNVETDTAEIRLMCTDTYLDTPRPDDGSITGPCESNGDKGSGGIKGGFVHQRMESKIGRWYSRTMSKTERMGMGLYVKYDGDPWADSDALAFSGVMVSMKEPGWYSFGFEIKDKKCDVPCIGIEMVHDGGKETWHSAATAIYCLMGSGQLLWDTVTGVDMAL |
| B/FIJI/8/2011 | 2011 | LPSTIQTLTLFLTSGGVLLSLYVSALLSYLLYSDILLKFSPTEITAPTMSLDCANASNVQAVNRSATKGVTFLLPEPEWTYPRLSCPGSTFQKALLISPHRFGETKGNSAPLIIREPFIACGPNECKHFALTHYAAQPGGYYNGTRGDRNKLRHLISVKLGKIPTVENSIFHMAAWSGSACHDGKEWTYIGVDGPDNDALLKVKYGEAYTDTYHSYANKILRTQESACNCIGGNCYLMITDGSASGVSECRFLKIREGRIIKEIFPTGRVKHTEECTCGFASNKTIECACRDNSYTAKRPFVKLNVETDTAEIRLMCTDTYLDTPRPDDGSITGPCESNGDKGSGGIKGGFVHQRMESKIGRWYSRTMSKTERMGMGLYVKYDGDPWADSDALAFSGVMVSMKEPGWYSFGFEIKDKKCDVPCIGIEMVHDGGKETWHSAATAIYCLMGSGQLLWDTVTGVDMAL |
| B/Fujian-Gulou/1727/2011 | 2011 | LPSTIQTLTLFLTSGGVLLSLYVSASLSYLLYSDILLKFSRTEITAPIMPLDCANASNVQAVNRSATKGVTLLLPEPEWMYPRLSCPGSTFQKALLISPHRFGETKGNSAPLIIREPFIACGPKECKHFALTHYAAQPGGYYNGTREDRNKLRHLISVKLGKIPTVENSIFHMAAWSGSACHDGREWTYIGVDGPDSNALLKIKYGEAYTDTYHSYAKNILRTQESACNCIGGDCYLMITDGPASGISECRFLKIREGRIIKEIFPTGRVKHTEECTCGFASNKTIECACRDNSYTAKRPFVKLNVETDTAEIRLMCTETYLDTPRPNDGSITGPCESNGDKGSGGIKGGFVHQRMASKIGRWYSRTMSKTKRMGMGLYVKYDGDPWTDSEALALSGVMVSMEEPGWYSFGFEIKDKKCDVPCIGIEMVHDGGKTTWHSAATAIYCLMGSGQLLWDTVTGVNMTL |
| B/Fujian-Yanping/1361/2011 | 2011 | LPSTIQTLTLFLTSGGVLLSLYVSASLSYLLYSDILLKFPSTEITAPTMPLDCANASNVQAVNRSATKGVTLLLPEPEWTYPRLSCPGSTFQKALLISPHRFGETKGNSAPLIIREPFIACGPKECKHFALTHYAAQPGGYYNGTRGDRNKLRHLISVKLGKIPTVENSIFHMAAWSGSACHDGKEWTYIGVDGPDNNALLKIKYGEAYTDTYHSYANNILRTQESACNCIGGNCYLMITDGSASGVSECRFLKIREGRIIKEIFPTGRIQHTEECTCGFASNKTIECACRDNSYTAKRPFVKLNVETDTAEIRLMCTKTYLDTPRPEDGSITGPCESNGGKGSGGIKGGFVHQRMASKIGRWYSRTMSKTERKGMGLYVKYNGDPWADSDALVFSGVMVSMEEPGWYSFGFEIKDKKCDVPCIGIEMVHDGGKETWHSAATAIYCLMGSGQLLWDTVTGVNMTL |
| B/FUKUSHIMA/45/2011 | 2011 | LPSTIQTLTLFLTSGGVLLSLYVSASLSYLLYSDILLKFSPTEITAPTMPLDCANASNVQAVNRSATKGVTLLLPEQEWTYPRLSCPGSTFQKALLISPHRFGETKGNSAPLIIREPFIACGPNECKHFALTHYAAQPGGYYNGTRGDRNKLRHLISVKLGKIPTVENSIFHMAAWSGSACHDGKEWTYIGVDGPDNNALLKVKYGEAYTDTYHSYANKILRTQESACNCIGGNCYLMITDGSASGVSECRFLKIREGRIIKEIFPTGRVKHTEECTCGFASNKTIECACRDNSYTAKRPFVKLNVETDTAEIRLMCTDTYLDTPRPNDGSITGPCESDGDKGSGGIKGGFVHQRMESKIGRWYSRTMSKTERMGMGLYVKYDGDPWTDSDALAFSGVMVSMKEPGWYSFGFEIKDKKCDVPCIGIEMVHDGGKETWHSAATAIYCLMGSGQLLWDTVTGVDMAL |
| B/Guangdong-Dongguanbendi/5398/2011 | 2011 | LPSTIQTLTLFLTSGGVLLSLYVSASLSYLLYSDILLKFSRTEITAPIMPLDCANASNVQAVNRSATKGVTLLLPEPEWTYPRLSCPGSTFQKALLISPHRFGETKGNSAPLIIREPFIACGPKECKHFALTHYAAQPGGYYNGTREDRNKLRHLISVKLGKIPTVENSIFHMAAWSGSACHDGREWTYIGVDGPDSNALLKIKYGEAYTDTYHSYAKNILRTQESACNCIGGDCYLMITDGPASGISECRFLKIREGRIIKEIFPTGRVKHTEECTCGFASNKTIECACRDNSYTAKRPFVKLNVETDTAEIRLMCTETYLDTPRPNDGSITGPCESNGDKGSGGIKGGFVHQRMASKIGRWYSRTMSKTKRMGMGLYVKYDGDPWTDSEALALSGVMVSMEEPGWYSFGFEIKDKKCDVPCIGIEMVHDGGKTTWHSAATAIYCLMGSGQLLWDTVTGVNMTL |
| B/Hubei-Shashi/37/2011 | 2011 | LPSTIQTLTLFLTSGGVLLSLYVSASLSYLLYLDILLKFPSTEITAPTMPLDCANASNVQAVNRSATKGVTLLLPEPEWTYPRLSCPGSTFQKALLISPHRFGETKGNSAPLIIREPFIACGPKECKHFALTHYAAQPGGYYNGTRGDRNKLRHLISVKLGKIPTVENSIFHMAAWSGSACHDGKEWTYIGVDGPDNNALLKIKYGEAYTDTYHSYANNILRTQESACNCIGGNCYLMITDGSASGVSECRFLKIREGRIMKEIFPTGRTQHTEECTCGFASNKTIECACRDNSYTAKRPFVKLNVETDTAEIRLMCTKTYLDTPRPDDGSITGPCESNGGKGSGGIKGGFVHQRMASKIGRWYSRTMSKTERKGMGLYVKYNGDPWADSDALAFSGVMVSMEEPGWYSFGFEIKDKKCDVPCIGIEMVHDGGKETWHSAATAIYCLMGSGQLLWDTVTGVNMTL |
| B/Hunan-Hengnan/35/2011 | 2011 | LPSTIQTLTLFLTSGGVLLSLYVSASLSYLLYLDILLKFPSTEITAPTMPLDCANASNVQAVNRSATKGVTLLLPEPEWTYPRLSCPGSTFQKALLISPHRFGETKGNSAPLIIREPFIACGPKECKHFALTHYAAQPGGYYNGTRGDRNKLRHLISVKLGKIPTVENSIFHMAAWSGSACHDGKEWTYIGVDGPDNNALLKIKYGEAYTDTYHSYANNILRTQESACNCIGGNCYLMITDGSASGVSECRFLKIREGRIMKEIFPTGRTQHTEECTCGFASNKTIECACRDNSYTAKRPFVKLNVETDTAEIRLMCTKTYLDTPRPDDGSITGPCESNGGKGSGGIKGGFVHQRMASKIGRWYSRTMSKTERKGMGLYVKYNGDPWADSDALAFSGVMVSMEEPGWYSFGFEIKDKKCDVPCIGIEMVHDGGKETWHSAATAIYCLMGSGQLLWDTVTGVNMTL |
| B/ISHIKAWA/108/2011 | 2011 | LPSTIQTLTLFLTSGGVLLSLYVSALLSYLLYSDILLKFSPTEITAPTMSLDCANASNVQAVNRSATKGVTFLLPEPEWTYPRLSCPGSTFQKALLISPHRFGETKGNSAPLIIREPFIACGPNECKHFALTHYAAQPGGYYNGTRGDRNKLRHLISVKLGKIPTVENSIFHMAAWSGSACHDGKEWTYIGVDGPDNDALLKVKYGEAYTDTYHSYANKILRTQESACNCIGGNCYLMITDGSASGVSECRFLKIREGRIIKEIFPTGRVKHTEECTCGFASNKTIECACRDNSYTAKRPFVKLNVETDTAEIRLMCTDTYLDTPRPDDGSITGPCESNGDKGSGGIKGGFVHQRMESKIGRWYSRTMSKTERMGMGLYVKYDGDPWADSDALAFSGVMVSMKEPGWYSFGFEIKDKKCDVPCIGIEMVHDGGKETWHSAATAIYCLMGSGQLLWDTVTGVDMAL |
| B/Israel/17/2011 | 2011 | LPSTIQTLTLFLTSGGVLLSLYVSASLSYLLYSGILLKFSPTEITAPTMPLDCANASDVQAVNRSATKGVTLLLPEPEWTYPRLSCPGSTFQKALLISPHRFGETKGNSAPLIIREPFIACGPNECKHFALTHYAAQPGGYYNGTRGDRNKLRHLISVKLGKIPTVENSIFHMAAWSGSACHDGKEWTYIGVDGPDNNALLKVKYGEAYTDTYHSYANKILRTQESACNCIGGNCYLMITDGSASGVSECRFLKIREGRIIKEIFPTGRVKHTEECTCGFASNKTIECACRDNSYTAKRPFVKLNVETDTAEIRLMCTDTYLDTPRPNDGSITGPCESNGDKGSGGIKGGFVHQRMESKIGRWYSRTMSKTERMGMGLYVKYDGDPWADSDALTFIGVMVSMKEPGWYSFGFEIKDKKCDVPCIGIEMVHDGGKETWHSAATAIYCLMGSGQLLWDTVTGVDMAL |
| B/Jiangsu-Canglang/1538/2011 | 2011 | LPSTIQTLTLFLTSGGVLLSLYVSASLSYLLYSDILLKFPSTEITAPTMPLDCANASNVQAVNRSATKGVTLLLPEPEWTYPRLSCPGSTFQKALLISPHRFGETKGNSAPLIIREPFIACGPKECKHFALTHYAAQPGGYYNGTRGDRNKLRHLISVKLGKIPTVENSIFHMAAWSGSACHDGKEWTYIGVDGPDNNALLKIKYGEAYTDTYHSYANNILRTQESACNCIGGNCYLMITDGSASGVSECRFLKIREGRIIKEIFPTGRIQHTEECTCGFASNKTIECACRDNSYTAKRPFVKLNVETDTAEIRLMCTKTYLDTPRPEDGSITGPCESNGGKGSGGIKGGFVHQRMASKIGRWYSRTMSKTERKGMGLYVKYNGDPWADSDALVFSGVMVSMEEPGWYSFGFEIKDKKCDVPCIGIEMVHDGGKETWHSAATAIYCLMGSGQLLWDTVTGVNMTL |
| B/Kenya/M03/2011 | 2011 | LPSTIQTLTLFLTSGGVLLSLYVSASLSYLLYSDILLKFSPTEITAPAMPLDCANASNVQAVNRSATKGVTLLLPEPEWTYPRLSCPGSTFQKALLISPHRFGETKGNSAPLIIREPFIACGPNECKHFALTHYAAQPGGYYNGTRGDRNKLRHLISVKLGKIPTVENSIFHMAAWSGSACHDGKEWTYIGVDGPDNNALLKVKYGEAYTDTYHSYANKILRTQESACNCIGGNCYLMITDGSASGVSECRFLKIREGRIIKEIFPTGRVKHTEECTCGFASNKTIECACRDNSYTAKRPFVKLNVETDTAEIRLMCTDTYLDTPRPNDGSITGPCESDGDKGSGGIKGGFVHQRMESKIGRWYSRTMSKTERMGMGLYVKYDGDPWADSDALAFSGVMVSMKEPGWYSFGFEIKDKKCDVPCIGIEMVHDGGKETWHSAATAIYCLMGSGQLLWDTVTGVDMAL |
| B/Laos/I973/2011 | 2011 | LPSTIQTLTLFLTSGGVLLSLYVSASLSYLLYSDILLKFSPTEITAPTMPLDCANASNVQAVNRSATKGVTLLLPEPEWTYPRLSCPGSTFQKALLISPHRFGETKGNSAPLIIREPFIACGPNECKHFALTHYAAQPGGYYNGTRGDRNKLRHLISVKLGKIPTVENSIFHMAAWSGSACHDGKEWTYIGVDGPDNNALLKVKYGEAYTDTYHSYANKILRTQESACNCIGGNCYLMITDGSASGVSECRFLKIREGRIIKEIFPTGRVKHTEECTCGFASNKTIECACRDNSYTAKRPFVKLNVETDTAEIRLMCTDTYLDTPRPNDGSITGPCESNGDKGSGGIKGGFVHQRMESKIGRWYSRTMSKTERMGMELYVKYDGDPWADSDALDFSGVMVSMKEPGWYSFGFEIKDKKCDVPCIGIEMVHDGGKETWHSAATAIYCLMGSGQLLWDTVTGVDMAL |
| B/Madagascar/1191/2011 | 2011 | LPSTIQTLTLFLTSGGVLLSLYVSASLSYLLYSDILLKFSPTEITAPTMPLDCANASNVQAVNRSATKGVTLLLPEPEWTYPRLSCPGSTFQKALLISPHRFGETKGNSAPLIIREPFIACGPNECKHFALTHYAAQPGGYYNGTRGDRNKLRHLISVKLGKIPTVENSIFHMAAWSGSACHDGKEWTYIGVDGPDNNALLKVKYGEAYTDTYHSYANKILRTQESACNCIGGNCYLMITDGSASGVSECRFLKIREGRIIKEIFPTGRVKHTEECTCGFASNKTIECACRDNSYTAKRPFVKLNVETDTAEIRLMCTDTYLDTPRPNDGSITGPCESNGDKGSGGIKGGFVHQRMESKIGRWYSRTMSKTERMGMGLYVKYDGDPWADSDALAFSGVMVSMKEPGWYSFGFEIKDKKCDVPCIGIEMVHDGGKETWHSAATAIYCLMGSGQLLWDTVTGVDMAL |
| B/New York/1248/2011 | 2011 | LPSTIQTLTLFLTSGGVLLSLYVSASLSYLLYSDILLKFSPTEITAPTMPLDCANASNVQAVNRSATKGVTLLLPEPEWTYPRLSCPGSTFQKALLISPHRFGETKGNSAPLIIREPFIACGPNECKHFALTHYAAQPGGYYNGTRGDRNKLRHLISVKLGKIPTVENSIFHMAAWSGSACHDGKEWTYIGVDGPDNNALLKVKYGEAYTDTYHSYANKILRTQESACNCIGGNCYLMVTDGSASGVSECRFLKIREGRIIKEIFPTGRVKHTEECTCGFASNKTIECACRDNSYTAKRPFVKLNVETDTAEIRLMCTDTYLDTPRPDDGSITGPCESNGDKGSGGIKGGFVHQRMESKIGRWYSRTMSKTERMGMGLYVKYDGDPWADSDALVFSGVMVSMKEPGWYSFGFEIKDKKCDVPCIGIEMVHDGGKETWHSAATAIYCLMGSGQLLWDTVTGVDMAL |
| B/New York/1295/2011 | 2011 | LPSTIQMLTLFLTSGGVLLSLYVSASLSYLLYSDILLKFSPTEITAPTMPLDCANASNVHAVNRSATKGVTFLLPEPEWTYPRLSCPGSTFQKALLISPHRFGETKGNSAPLIIREPFIACGPNECKHFALTHYAAQPGGYYNGTRGDRNKLRHLISVKLGKIPTVENSIFHMAAWSGSACHDGKEWTYIGVDGPDNNALLKIKYGEAYTDTYHSYANNILRTQESACNCIGGNCYLMITDGSASGVSECRFLKIREGRIIKEIFPTGRVKHTEECTCGFASNKTIECACRDNSYTAKRPFVKLNVETDTAEIRLMCTDTYLDTPRPDDGSITGPCESNGDKGSGGIKGGFVHQRMASKIGRWYSRTMSKTERKGMGLYVKYDGDPWTDSDALAFRGVMVSMKEPGWYSFGFEIKDKKCDVPCIGIEMVHDGGKETWHSAATAIYCLMGSGQLLWDTVTGVDMAL |
| B/Norway/1276/2011 | 2011 | LPSTIQTLTLFLTSGGVLLSLYVSALLSYLLYSDILLKFSPTEITAPTMSLDCANASNVQAVNRSATKGVTFLLPEPEWTYPRLSCPGSTFQKALLISPHRFGETKGNSAPLIIREPFIACGPNECKHFALTHYAAQPGGYYNGTRGDRNKLRHLISVKLGKIPTVENSIFHMAAWSGSACHDGKEWTYIGVDGPDNDALLKVKYGEAYTDTYHSYANKILRTQESACNCIGGNCYLMITDGSASGVSECRFLKIREGRIIKEIFPTGRVKHTEECTCGFASNKTIECACRDNSYTAKRPFVKLNVETDTAEIRLMCTDTYLDTPRPDDGSITGPCESNGDKGSGGIKGGFVHQRMESKIGRWYSRTMSKTERMGMGLYVKYDGDPWADSDALAFSGVMVSMKEPGWYSFGFEIKDKKCDVPCIGIEMVHDGGKETWHSAATTIYCLMGSGQLLWDTVTGVDMAL |
| B/Novosibirsk/05/2011 | 2011 | LPSTIQTLTLFLTSGGVLLSLYVSALLSYLLYSDILPKFSPTEITAPTMSLDCANASNVQAVNRSATKGVTFLLSEPEWTYPRLSCPGSTFQKALLISPHRFGETKGNSAPLIIREPFIACGPNECKHFALTHYAAQPGGYYNGTRGDRNKLRHLISVKLGKIPTVENSIFHMAAWSGSACHDGKEWTYIGVDGPDNDALLKVKYGEAYTDTYHSYANKILRTQESACNCIGGNCYLMITDGSASGVSECRFLKIREGRIIKEIFPTGRVKHTEECTCGFASNKTIECACRDNSYTAKRPFVKLNVETDTAEIRLMCTDTYLDTPRPDDGSITGPCESNGDKGSGGIKGGFVHQRMESKIGRWYSRTMSKTERMGMGLYVKYDGDPWADSDALAFSGVMVSMKEPGWYSFGFEIKDKKCDVPCIGIEMVHDGGKETWHSAATAIYCLMGSGQLLWDTVTGVDMAL |
| B/PHILIPPINES/3701/2011 | 2011 | LPSTIQTLTLFLTSGGVLLSLYVSALLSYLLYSDVLLKFSPTEITAPTMSLDCANASNVQAVNRSATKGVTFLLPEPEWTYPRLSCPGSTFQKALLISPHRFGETKGNSAPLIIREPFIACGPNECKHFALTHYAAQPGGYYNRTRGDRNKLRHLISVKLGKIPTVENSIFHMAAWSGSACHDGKEWTYIGVDGPDNDALLKVKYGEAYTDTYHSYANKILRTQESACNCIGGNCYLMITDGSASGVSECRFLKIREGRIIKEIFPIGRVKHTEECTCGFASNKTIECACRDNSYTAKRPFVKLNVETDTAEIRLMCTDTYLDTPRPDDGSITGPCESNGDKGSGGIKGGFVHQRMESKIGRWYSRTMSKTERMGMGLYVKYDGDPWADSDALAFSGVMVSMKEPGWYSFGFEIKDKKCDVPCIGIEMVHDGGKETWHSAATAIYCLMGSGQLLWDTVTGVDMAL |
| B/PHILIPPINES/3971/2011 | 2011 | LPSTIQTLTLFLTSGGVLLSLYVSASLSYLLYSDILLKFSPTEITAPTMPLDCANASNVQAVNRSATKGVTLLLPEPEWTYPRLSCPGSTFQKALLISPHRFGETKGNSAPLIIREPFIACGPNECKHFALTHYAAQPGGYYNGTRGDRNKLRHLISVKLGKIPTVENSIFHMAAWSGSACHDGKEWTYIGVDGPDNNALLKVKYGEAYTDTYHSYANKILRTQESACNCIGGNCYLMITDGSASGVSECRFLKIREGRIIKEIFPTGRVKHTEECTCGFASNKTIECACRDNSYTAKRPFVKLNVETDTAEIRLMCTDTYLDTPRPNDGSITGPCESNGEKGSGGIKGGFVHQRMESKIGRWYSRTMSKTERMGMGLYVKYDGDPWADSDALTFSGVMVSMKEPGWYSFGFEIKDKKCDVPCIGIEMVHDGGKETWHSAATAIYCLMGSGQLLWDTVTGVDMAL |
| B/Shanghai-Luwan/1135/2011 | 2011 | LPSTIQTLTLFLTSGGVLLSLYVSASLSYLLYSDILLKFPSTEITAPTMPLDCANASNVQAVNRSATKGVTLLLPEPEWTYPRLSCPGSTFQKALLISPHRFGETKGNSAPLIIREPFIACGPKECKHFALTHYAAQPGGYYNGTRGDRNKLRHLISVKLGKIPTVENSIFHMAAWSGSACHDGKEWTYIGVDGPDNNALLKIKYGEAYTDTYHSYANNILRTQESACNCIGGNCYLMITDGSASGVSECRFLKIREGRIIKEIFPTGRIQHTEECTCGFASNKTIECACRDNSYTAKRPFVKLNVETDTAEIRLMCTKTYLDTPRPEDGSITGPCESNGGKGSGGIKGGFVHQRMASKIGRWYSRTMSKTERKGMGLYVKYNGDPWADSDALVFSGVMVSMEEPGWYSFGFEIKDKKCDVPCIGIEMVHDGGKETWHSAATAIYCLMGSGQLLWDTVTGVNMTL |
| B/Shanghai-Songjiang/176/2011 | 2011 | LPSTIQTLTLFLTSGGVLLSLYVSASLSYLLYSDILLKFPSTEITAPTMPLDCANASNVQAVNRSATKGVTLLLPEPEWTYPRLSCPGSTFQKALLISPHRFGETKGNSAPLIIREPFIACGPKECKHFALTHYAAQPGGYYNGTRGDRNKLRHLISVKLGKIPTVENSIFHMAAWSGSACHDGKEWTYIGVDGPDNNALLKIKYGEAYTDTYHSYANNILRTQESACNCIGGNCYLMITDGSASGVSECRFLKIREGRIIKEIFPTGRIQHTEECTCGFASNKTIECACRDNSYTAKRPFVKLNVETDTAEIRLMCTKTYLDTPRPDDGSITGPCESNGGKGSGGIKGGFVHQRMASKIGRWYSRTMSKTERKGMGLYVKYNGDPWADSDALVFSGVMVSMEEPGWYSFGFEIKDKKCDVPCIGIEMVHDGGKETWHSAATAIYCLMGSGQLLWDTVTGVNMTL |
| B/Singapore/DMS1493/2011 | 2011 | LPSTIQTLTLFLTSGGVLLSLYVSASLSYLLYSDILLKFSRTEITAPIMPLDCANASNVQAVNRSATKGVTPLLPEPEWTYPRLSCPGSTFQKALLISPHRFGETKGNSAPLIIREPFIACGPKECKHFALTHYAAQPGGYYNGTREDRNKLRHLISVKLGKIPTVENSIFHMAAWSGSACHDGREWTYIGVDGPDSNALLKIKYGEAYTDTYHSYAKNILRTQESACNCIGGDCYLMITDGPASGISECRFLKIREGRIIKEIFPTGRVKHTEECTCGFASNKTIECACRDNSYTAKRPFVKLNVETDTAEIRLMCTETYLDTPRPNDGSITGPCESNGDEGSGGIKGGFVHQRMASKIGRWYSRTMSKTKRMGMGLYVKYDGDPWTDSEALALSGVMVSMEEPGWYSFGFEIKDKKCDVPCIGIEMVHDGGKTTWHSAATAIYCLMGSGQLLWDTVTGVNMTM |
| B/Singapore/DMS1613/2011 | 2011 | LPSTIQTLTLFLTSGGVLLSLYVSASLSYLLYSDILLKFSPTEITAPTIPLDCANASNVQAVNRSATKGVTLLLPEPEWTYPRLSCPGSTFQKALLISPHRFGETKGNSAPLIIREPFIACGPNECKHFALTHYAAQPGGYYNGTRGDRNKLRHLISVKLGKIPTVENSIFHMAAWSGSACHDGKEWTYIGVDGPDNNALLKVKYGEAYTDTYHSYANKILRTQESACNCIGGNCYLMITDGSASGVSECRFLKIREGRIIKEIFPTGRVKHTEECTCGFASNKTIECACRDNSYTAKRPFVKLNVETDTAEIRLMCTDTYLDTPRPNDGSITGPCESNGDKGSGGIKGGFVHQRMESKIGRWYSRTMSKTERMGMGLYVKYDGDPWTDSDALAFSGVMVSMKEPGWYSFGFEIKDKKCDVPCIGIEMVHDGGKETWHSAATAIYCLMGSGQLLWDTVTGVDMAL |
| B/Singapore/DMS1625/2011 | 2011 | LPSTIQTLTLFLTSGGVLLSLYVSASLSYLLYSDILLKFSPTEITAPTMPLDCANASNVQAVNRSATKGVTLLLPEPEWTYPRLSCPGSTFQKALLISPHRFGETKGNSAPLIIREPFIACGPNECKHFALTHYAAQPGGYYNGTRGDRNKLRHLISVKLGKIPTVENSIFHMAAWSGSACHDGKEWTYIGVDGPDNNALLKVKYGEAYTDTYHSYANKILRTQESACNCIGGNCYLMITDGSASGVSECRFLKIREGRIIKEIFPTGRVKHTEECTCGFASNKTIECACRDNSYTAKRPFVKLNVETDTAEIKLMCTDTYLDTPRPNDGSITGPCESDGDKGSGGIKGGFVHQRMESKIGRWYSRTMSKTERMGMGLYVKYDGDPWTDSDALAFSGVMVSMKEPGWYSFGFEIKDKKCDVPCIGIEMVHDGGKETWHSAATAIYCLMGSGQLLWDTVTGVDMAL |
| B/Slovakia/302/2011 | 2011 | LPSTIQTLTLFLTSGGVLLSLYVSALLSYLLYSDILLKFSPTEITAPTMSLDCANASNVQAVNRSATKGVTFLLPEPEWTYPRLSCPGSTFQKALLISPHRFGETKGNSAPLIIREPFIACGPNECKHFALTHYAAQPGGYYNGTRGDRNKLRHLISVKLGKIPTVENSIFHMAAWSGSACHDGKEWTYIGVDGPDNDALLKVKYGEAYTDTYHSYANKILRTQESACNCIGGNCYLMITDGSASGVSECRFLKIREGRIIKEILPTGRVKHTEECTCGFASNKTIECACRDNSYTAKRPFVKLNVETDTAEIRLMCTDTYLDTPRPDDGSITGPCESNGDKGSGGIKGGFVHQRMESKIGRWYSRTMSKTERMGMGLYVKYDGDPWADSDALAFSGVMVSMKEPGWYSFGFEIKDKKCDVPCIGIEMVHDGGKETWHSAATAIYCLMGSGQLLWDTVTGVDMAL |
| B/South Auckland/12/2011 | 2011 | LPSTIQTLTLFLTSGGVLLSLYVSASLSYLLYSDILLKFSPTEITAPTMPLDCANASNVQAVNRSATKGVTLLLPEPEWTYPRLSCPGSTFQKALLISPHRFGETKGNSAPLIIREPFIACGPNECKHFALTHYAAQPGGYYNGTRGDRNKLRHLISVKLGKIPTVENSIFHMAAWSGSACHDGKEWTYIGVDGPDNNALLKVKYGEAYTDTYHSYANKILRTQESACNCIGGNCYLMITDGSASGVSECRFLKIREGRIIKEIFPTGRVKHTEECTCGFASNKTIECACRDNSYTAKRPFVKLNVETDTAEVRLMCTDTYLDTPRPNDGSITGPCESDGDKGSGGIKGGFVHQRMESKIGRWYSRTMSKTERMGMGLYVKYDGDPWADSDALTFSGVMVSMKEPGWYSFGFEIKDKKCDVPCIGIEMVHDGGKETWHSAATAIYCLMGSGQLLWDTVTGVDMAL |
| B/SOUTH AUSTRALIA/2/2011 | 2011 | LPSTIQMLTLFLTSGGVLLSLYVSASLSYLLYSDILLKFSPTEITAPTMPLDCANASNVHAVNRSATKGVTFLLPEPEWTYPRLSCPGSTFQKALLISPHRFGETKGNSAPLIIREPFIACGPNECKHFALTHYAAQPGGYYNGTRGDRNKLRHLISVKLGKIPTVENSIFHMAAWSGSACHDGKEWTYIGVDGPDNNALLKIKYGEAYTDTYHSYANNILRTQESACNCIGGNCYLMITDGSASGVSECRFLKIREGRIIKEIFPTGRVKHTEECTCGFASNKTIECACRDNSYTAKRPFVKLNVETDTAEIRLMCTDTYLDTPRPDDGSITGPCESNGDKGSGGIKGGFVHQRMASKIGRWYSRTMSKTERKGMGLYVKYDGDPWTDSDALAFRGVMVSMKEPGWYSFGFEIKDKKCDVPCIGIEMVHDGGKETWHSAATAIYCLMGSGQLLWDTVTGVDMAL |
| B/St Petersburg/5/2011 | 2011 | LPSTIQTLTLFLTSGGVLLSLYVSALLSYLLYSDILLKFSPTEITAPTMSLDCANASNVQAVNRSATKGVTFLLPEPEWTYPRLSCPGSTFQKALLISPHRFGETKGNSAPLIIREPFIACGPNECKHFALTHYAAQPGGYYNGTRGDRNKLRHLISVKLGKIPTVENSIFHMAAWSGSACHDGKEWTYIGVDGPDNDALLKVKYGEAYTDTYHSYANKILRTQESACNCIGGNCYLMITDGSASGVSECRFLKIREGRIIKEIFPTGRVKHTEECTCGFASNKTIECACRDNSYTAKRPFVKLNVETDTAEIRLMCTDTYLDTPRPDDGSITGPCESNGDKGSGGIKGGFVHQRMESKIGRWYSRTMSKTERMGMGLYVKYDGDPWADSDALAFSGVMVSMKEPGWYSFGFEIKDKKCDVPCIGIEMVHDGGKETWHSAATAIYCLMGSGQLLWDTVTGVDMAL |
| B/Stavropol/RII7/2011 | 2011 | LPSTIQTLTLFLTSGGVLLSLYVSASLSYLLYSDILLKFSPTETTAPTMSLDCANASNVQAVNRSATKGVTFLLPEPEWTYPRLSCPGSTFQKALLISPHRFGETKGNSAPLIIREPFIACGPNECKHFALTHYAAQPGGYYNGTRGDRNKLRHLISVKLGKIPTVENSIFHMAAWSGSACHDGKEWTYIGVDGPDNDALLKVKYGEAYTDTYHSYANKILRTQESACNCIGGNCYLMITDGSASGVSECRFLKIREGRIIKEIFPTGRVKHTEECTCGFASNKTIECACRDNSYTAKRPFVKLNVETDTAEIRLMCTDTYLDTPRPDDGSITGPCESNGDKGSGGIKGGFVHQRMESKIGRWYSRTMSKTERMGMGLYVKYDGDPWADSDALAFSGVMVSMKEPGWYSFGFEIKDKKCDVPCIGIEMVHDGGKETWHSAATAIYCLMGSGQLLWDTVTGVDMAL |
| B/Taiwan/1128/2011 | 2011 | LPSTIQTLTLFLTSGGVLLSLYVSASLSYLLYSDILLKFSQTEITAPIMPLDCANASNVQAVNHSAAKGVTLLLPEPEWTYPRLSCPGSTFQKALLISPHRFGEIKGNSAPLIIREPFIACGPTECKHFALTHYAAQPGGYYNGTREDRNKLRHLISVKLGKIPTVENSIFHMAAWSGSACHDGKEWTYIGVDGPDSNALLKIKYGEAYTDTYHSYAKNILRTQESACNCIGGDCYLMITDGPASGVSECRFLKIREGRIIKEIFPTGRVKHTEECTCGFASNKTIECACRDNRYTAKRPFVKLNVETDTAEIRLMCTETYLDTPRPNDGSITGPCESDGDKGSGGIKGGFVHQRMASKIGRWYSRTMSKTKRMGMGLYVKYDGDPWTDSEALALSGVMVSMEEPGWYSFGFEIKDKKCDVPCIGIEMVHDGGKTTWHSAATAIYCLMGSGQLLWDTVTGVDIAL |
| B/Thailand/CU-C1768/2011 | 2011 | LPSTIQTLTLFLTSGGVLLSLYVSASLSYLLYSDILLKFSPTEITAPTMPLDCANASNVQAVNRSATKGVTLLLPEPEWTYPRLSCPGSTFQKALLISPHRFGETKGNSAPLIIREPFIACGPNECKHFALTHYAAQPGGYYNGTRGDRNKLRHLISVKLGKIPTVENSIFHMAAWSGSACHDGKEWTYIGVDGPDNNALLKVKYGEAYTDTYHSYANKILRTQESACNCIGGNCYLMITDGSASGVSECRFLKIREGRIIKEIFPTGRVKHTEECTCGFASNKTIECACRDNSYTAKRPFVKLNVETDTAEIRLMCTDTYLDTPRPNDGSITGPCESNGDKGSGGIKGGFVHQRMESKIGRWYSRTMSKTERMGMGLYVKYDGDPWADSDALAFSGVMVSMKEPGWYSFGFEIKDKKCDVPCIGIEMVHDGGKETWHSAATAIYCLMGSGQLLWDTVTGVDMAL |
| B/Tianjin-Hexi/156/2011 | 2011 | LPSTIQTLTLFLTSGGVLLSLYVSASLSYLLYSDILLKFSRTEITAPIMPLDCANASNVQAVNRSATKGVTLLLPEQEWMYPRLSCPGSTFQKALLISPHRFGETKGNSAPLIIREPFIACGPKECKHFALTHYAAQPGGYYNGTREDRNKLRHLISVKLGKIPTVENSIFHMAAWSGSACHDGREWTYIGVDGPDSNALLKIKYGEAYTDTYHSYAKNILRTQESACNCIGGDCYLMITDGPASGISECRFLKIREGRIIKEIFPTGRVKHTEECTCGFASNKTIECACRDNSYTAKRPFVKLNVETDTAEIRLMCTETYLDTPRPNDGSITGPCESNGDKGSGGIKGGFVHQRMASKIGRWYSRTMSKTKRMGMGLYVKYDGDPWTDSEALALSGVMVSMEEPGWYSFGFEIKDKKCDVPCIGIEMVHDGGKTTWHSAATAIYCLMGSGQLLWDTVTGVNMTL |
| B/TOKYO/98724/2011 | 2011 | LPSTIQTLTLFLTSGGVLLSLYVSALLSYLLYSDILLKFSRTEITAPIMPLDCANASNVQAVNRSATKGVTLLLPEPEWTYPRLSCPGSTFQKALLISPHRFGENKGNSAPLIIREPFIACGPKECKHFALTXYAAQPGGYYNXTREXRNKLRHLISVKLGKIPTVENSIFHMAAWSGSACHDGREWTYIGVDGPDSNALLKIKYGEAYTDTYHSYAKNILRTQESACNCIGGDCYLMITDGPASGISECRFLKIREGRIIKEIFPTGRVKHTEECTCGFASNKTIECACRDNNYTAKRPFVKLNVETDTAEIRLMCTETYLDTPRPNDGSITGPCESNGDKGSGGIKGGFVHQRMASKIGRWYSRTMSKTKRMGMGLYVKYDGDPWTDSEALALSGVMVSMEEPGWYSFGFEIKDKKCDVPCIGIEMVHDGGKTTWHSAATAIYCLMGSGQLLWDTVTGVNMTL |
| B/Townsville/26/2011 | 2011 | LPSTIQMLTLFLTSGGVLLSLYVSASLSYLLYSDILLKFSPTEITAPTMPLDCANASNVHAVNRSATKGVTFLLPEPEWTYPRLSCPGSTFQKALLISPHRFGETKGNSAPLIIREPFIACGPNECKHFALTHYAAQPGGYYNGTRGDRNKLRHLISVKLGKIPTVENSIFHMAAWSGSACHDGKEWTYIGVDGPDNNALLKIKYGEAYTDTYHSYANNILRTQESACNCIGGNCYLMITDGSASGVSECRFLKIREGRIIKEIFPTGRVKHTEECTCGFASNKTIECACRDNSYTAKRPFVKLNVETDTAEIRLMCTDTYLDTPRPDDGSITGPCESNGDKGSGGIKGGFVHQRMASKIGRWYSRTMSKTERKGMGLYVKYDGDPWTDSDALAFRGVMVSMKEPGWYSFGFEIKDKKCDVPCIGIEMVHDGGKETWHSAATAIYCLMGSGQLLWDTVTGVDMAL |
| B/TOWNSVILLE/4/2011 | 2011 | LPSTIQTLTLFLTSGGVLLSLYVSALLSYLLYSDILLKFSPTEITAPTMSLDCANTSNVQAVNRSATKGVTFLLPEPEWTYPRLSCPGSTFQKALLISPHRFGETKGNSAPLIIREPFIACGPNECKHFALTHYAAQPGGYYNGTRGDRNKLRHLISVKLGKIPTVENSIFHMAAWSGSACHDGKEWTYIGVDGPDNDALLKVKYGEAYTDTYHSYANKILRTQESACNCIGGNCYLMITDGSASGVSECRFLKIREGRIIKEIFPTGRVKHTEECTCGFASNKTIECACRDNSYTAKRPFVKLNVETDTAEIRLMCTDTYLDTPRPDDGSITGPCESNGDKGSGGIKGGFVHQRMESKIGRWYSRTMSKTERMGMGLYVKYDGDPWADSDALAFSGVMVSMKEPGWYSFGFEIKDKKCDVPCIGIEMVHDGGKETWHSAATAIYCLMGSGQLLWDTVTGVNMAL |
| B/Ukraine/7/2011 | 2011 | LPSTIQTLTLFLTSGGVLLSLYVSASLSYLLYSDILLKFSPTETTAPTMSLDCANASNVQAVNRSATKGVTFLFPEPEWTYPRLSCPGSTFQKALLISPHRFGETKGNSAPLIIREPFIACGPNECKHFALTHYAAQPGGYYNGTRGDRNKLRHLISVKLGKIPTVENSIFHMAAWSGSACHDGKEWTYIGVDGPDNDALLKVKYGEAYTDTYHSYANKILRTQESACNCIGGNCYLMITDGSASGVSECRFLKIREGRIIKEIFPTGRVKHTEECTCGFASNKTIECACRDNSYTAKRPFVKLNVETDTAEIRLMCTDTYLDTPRPDDGSITGPCESNGDKGSGGIKGGFVHQRMESKIGRWYSRTMSKTERMGMGLYVKYDGDPWADSDALAFSGVMVSMKEPGWYSFGFEIKDKKCDVPCIGIEMVHDGGKETWHSAATAIYCLMGSGQLIWDTVTGVDMAL |
| B/Victoria/506/2011 | 2011 | LPSTIQTLTLFLTSGGVLLSLYVSALLSYLLYSDILLKFSPTEITAPTMSLDCANASNVQAVNRSATKGVTFLLPEPEWTYPRLSCPGSTFQKALLISPHRFGETKGNSAPLIIREPFIACGPNECKHFALTHYAAQPGGYYNGTRGDRNKLRHLISVKLGKIPTVENSIFHMAAWSGSACHDGKEWTYIGVDGPDNDALLKVKYGEAYTDTYHSYANKILRTQESACNCIGGNCYLMITDGSASGVSECRFLKIQEGRIIKEIFPTGRVKHTEECTCGFASNKTIECACRDNSYTAKRPFVKLNVETDTAEIRLMCTDTYLDTPRPDDGSITGPCESNGDKGSGGIKGGFVHQRMESKIGRWYSRTMSKTERMGMGLYVKYDGDPWADSDALAFSGVMVSMKEPGWYSFGFEIKDKKCDVPCIGIEMVHDGGKETWHSAATAIYCLMGSGQLLWDTVTGVDMAL |
| B/WELLINGTON/1/2011 | 2011 | LPSTIQTLTLFLTSGGVLLSLYVSALLSYLLYSDILPKFSPTEITAPTMSLDCANASNVQAVNRSATKGVTFLLPEPEWTYPRLSCPGSTFQKALLISPHRFGETKGNSAPLIIREPFIACGPNECKHFALTHYAAQPGGYYNGTRGDRNKLRHLISVKLGKIPTVENSIFHMAAWSGSACHDGKEWTYIGVDGPDNDALLKVKYGEAYTDTYHSYANKILRTQESACNCIGGNCYLMITDGSASGVSECRFLKIREGRIIKEIFPTGRVKHTEECTCGFASNKTIECACRDNSYTAKRPFVKLNVETDTAEIRLMCTDTYLDTPRPDDGSITGPCESNGDKGSGGIKGGFVHQRMESKIGRWYSRTMSKTERMGMGLYVKYDGDPWADSDALVFSGVMVSMKEPGWYSFGFEIKDKKCDVPCIGIEMVHDGGKETWHSAATAIYCLMGSGQLLWDTVTGVDMAL |
| B/Wellington/26/2011 | 2011 | LPSTIQTLTLFLTSGGVLLSLYVSALLSYLLYSDILLKFSPTEITAPTMSLDCANASNVQAVNRSATKGVTFLLPEPEWTYPRLSCPGSTFQKALLISPHRFGETKGNSAPLIIREPFIACGPNECKHFALTHYAAQPGGYYNGTRGDRNKLRHLISVKLGKIPTVENSIFHMAAWSGSACHDGKEWTYIGVDGPDNDALLKVKYGEAYTDTYHSYANKILRTQESACNCIGGNCYLMITDGSASGVSECRFLKIREGRITKEIFPTGRVKHTEECTCGFASNKTIECACRDNSYTAKRPFVKLNVETDTAEIRLMCTDTYLDTPRPDDGSITGPCESNGDKGSGGIKGGFVHQRMESKIGRWYSRTMSKTERMGMGLYVKYDGDPWADSDALAFSGVMVSMKEPGWYSFGFEIKDKKCDVPCIGIEMVHDGGKETWHSAATAIYCLMGSGQLLWDTVTGVDMAL |
| B/YOKOHAMA/64/2011 | 2011 | LPSTIQTLTLFLTSGGVLLSLYVSALLSYLLYSDILLKFSPTEITAPTMSLDCANASNVQAVNRSATKGVTFLLPEPEWTYPRLSCPGSTFQKALLISPHRFGETKGNSAPLIIREPFIACGPNECKHFALTHYAAQPGGYYNGTRGDRNKLRHLISVKLGKIPTVENSIFHMAAWSGSACHDGKEWTYIGVDGPDNDALLKVKYGEAYTDTYHSYANKILRTQESACNCIGGNCYLMITDGSASGVSECRFLKIREGRIIKEIFPTGRVKHTEECTCGFASNKTIECACRDNSYTAKRPFVKLNVETDTAEIRLMCTDTYLDTPRPDDGSITGPCESNGDKGSGGIKGGFVHQRMESKIGRWYSRTMSKTERMGMGLYVKYDGDPWADSDALAFSGVMVSMKEPGWYSFGFEIKDKKCDVPCIGIEMVHDGGKETWHSAATAIYCLMGSGQLLWDTVTGVDMAL |
| B/CANBERRA/1/2012 | 2012 | LPSTIQTLTLFLTSGGVLLSLYVSASLSYLLYSDILLKFSPTEITAPTMPLDCANASNVQAVNRSATKGVTFLLPEPEWTYPRLSCPGSTFQKALLISPHRFGETKGNSAPLIIREPFIACGPNECKHFALTHYAAQPGGYYNGTRGDRNKLRHLISVKLGKIPTVENSIFHMAAWSGSACHDGKEWTYIGVDGPDNNALLKVKYGEAYTDTYHSYANKILRTQESACNCIGGNCYLMITDGSASGVSECRFLKIREGRIIKEIFPTGRVKHTEECTCGFASNKTIECACRDNRYTAKRPFVKLNVETDTAEIRLMCTDTYLDTPRPNDGSITGPCESDGDKGSGGIKGGFVHQRMKSKIGRWYSRTMSKTERMGMGLYVKYDGDPWADSDALAFSGVMVSMKEPGWYSFGFEIKDKKCDVPCIGIEMVHDGGKETWHSAATAIYCLMGSGQLLWDTVTGVDMAL |
| B/Catalonia/S5057/2012 | 2012 | LPSTIQTLTLFLTSGGVLLSLYVSASLSYLLYSDILLKFSRTEITAPIMPLDCANASNVQAVNRSATKGVTPLLPEPEWTYPRLSCPGSTFQKALLISPHRFGETKGNSAPLIIREPFIACGPKECKHFALTHYAAQPGGYYNGTREDRNKLRHLISVKLGKIPTVENSIFHMAAWSGSACHDGREWTYIGVDGPDSNALLKIKYGEAYTDTYHSYAKNILRTQESACNCIGGDCYLMITDGPASGISECRFLKIREGRIIKEIFPTGRVKHTEECTCGFASNKTIECACRDNSYTAKRPFVKLNVETDTAEIRLMCTETYLDTPRPNDGSITGPCESNGDEGSGGIKGGFVHQRMASKIGRWYSRTMSKTKRMGMGLYVKYGGDPWTDSEALALSGVMVSMEEPGWYSFGFEIKDKKCDVPCIGIEMVHDGGKTTWHSAATAIYCLMGSGQLLWDTVTGVNMTL |
| B/Chattisgarh/V25/2012 | 2012 | LPSTIQTLTLFLTSGGVLLSLYVSASLSYLLYSDILLKFSPTEITAPTMPLDCANASNVQAVNRSATKGVTLLLPEPEWTYPRLSCPGSTFQKALLISPHRFGETKGNSAPLIIREPFIACGPNECKHFALTHYAAQPGGYYNGTRGDRNKLRHLISVKLGKIPTVENSIFHMAAWSGSACHDGKEWTYIGVDGPDNNALLKVKYGEAYTDTYHSYANKILRTQESACNCIGGNCYLMITDGSASGVSECRFLKIREGRIIKEIFPTGRVKHTEECTCGFASNKTIECACRDNSYTAKRPFVKLNVETDTAEIRLMCTDTYLDTPRPNDGSITGPCESNGDKGSGGIKGGFVHQRMESKIGRWYSRTMSKTERMGMGLYVKYDGDPWADSDALAFSGVMVSMKEPGWYSFGFEIKDKKCDVPCIGIEMVHDGGKETWHSAATAIYCLMGSGQLLWDTVTGVDMAL |
| B/Delhi/21/2012 | 2012 | LPSTIQTLTLFLTSGGVLLSLYVSASLSYLLYSDILLKFSPTEITAPTMPLDCANASNVQAVNRSATKGVTLLLPEPEWTYPRLSCPGSTFQKALLISPHRFGETKGNSAPLIIREPFIACGPNECKHFALTHYAAQPGGYYNGTRGDRNKLRHLISVKLGKIPTVENSIFHMAAWSGSACHDGKEWTYIGVDGPDNNALLKVKYGEAYTDTYHSYANKILRTQESACNCIGGNCYLMITDGSASGVSECRFLKIREGRIIKEIFPTGRVKHTEECTCGFASNKTIECACRDNSYTAKRPFVKLNVETDTAEIRLMCTDTYLDTPRPNDGSITGPCESNGDKGSGGIKGGFVHQRMESKIGRWYSRTMSKTERMGMGLYVKYDGDPWADSDALAFSGVMVSMKEPGWYSFGFEIKDKKCDVPCIGIEMVHDGGKETWHSAATAIYCLMGSGQLLWDTVTGVDMAL |
| B/Delhi/V18/2012 | 2012 | LPSTIQTLTLFLTSGGVLLSLYVSASLSYLLYSDILLKFSPTEITAPTMPLDCANASNVQAVNRSATKGVTLLLPEPEWTYPRLSCPGSTFQKALLISPHRFGETKGNSAPLIIREPFIACGPNECKHFALTHYAAQPGGYYNGTRGDRNKLRHLISVKLGKIPTVENSIFHMAAWSGSACHDGKEWTYIGVDGPDNNALLKVKYGEAYTDTYHSYANKILRTQESACNCIGGNCYLMITDGSASGVSECRFLKIREGRIIKEIFPTGRVKHTEECTCGFASNKTIECACRDNSYTAKRPFVKLNVETDTAEIRLMCTDTYLDTPRPNDGSITGPCESNGDKGSGGIKGGFVHQRMESKIGRWYSRTMSKTERMGMGLYVKYDGDPWADSDALAFSGVMVSMKEPGWYSFGFEIKDKKCDVPCIGIEMVHDGGKETWHSAATAIYCLMGSGQLLWDTVTGVDMAL |
| B/Finland/235/2012 | 2012 | LPSTIQTLTLFLTSGGVLLSLYVSASLSYLLYSDILLKFSPTEITAPTMPLDCANASNVQAVNRSATKGVTLLLPEPEWTYPRLSCPGSTFQKALLISPHRFGETKGNSAPLIIREPFIACGPNECKHFALTHYAAQPGGYYNGTRGDRNKLRHLISVKLGKIPTVENSIFHMAAWSGSACHDGKEWTYIGVDGPDNNALLKVKYGEAYTDTYHSYANKILRTQESACNCIGGNCYLMITDGSASGVSECRFLKIREGRIIKEIFPTGRVKHTEECTCGFASNKTIECACRDNSYTAKRPFVKLNVETDTAEIRLMCTDTYLDTPRPDDGSITGPCESNGDKGSGGIKGGFVHQRMESKIGRWYSRTMSKTERMGMGLYVKYDGDPWADSDALTFSGVMVSMKEPGWYSFGFEIKDKKCDVPCIGIEMVHDGGKETWHSAATAIYCLMGSGQLLWDTVTGVDMAL |
| B/Fujian-Gulou/1168/2012 | 2012 | LPSTIQTLTLFLTSGGVLLSLYVSASLSYLLYLDILLKFPSTEITAPTMPLDCANASNVQAVNRSATKGVTLLLPEPEWTYPRLSCPGSTFQKALLISPHRFGETKGNSAPLIIREPFIACGPKECKHFALTHYAAQPGGYYNGTRGDRNKLRHLISVKLGKIPTVENSIFHMAAWSGSACHDGKEWTYIGVDGPDNNALLKIKYGEAYTDTYHSYANNILRTQESACNCIGGNCYLMITDGSASGVSECRFLKIREGRIMKEIFPTGRTQHTEECTCGFASNKTIECACRDNSYTAKRPFVKLNVETDTAEIRLMCTKTYLDTPRPDDGSITGPCESNGGKGSGGIKGGFVHQRMASKIGRWYSRTMSKTERKGMGLYVKYNGDPWADSDALAFSGVMVSMEEPGWYSFGFEIKDKKCDVPCIGIEMVHDGGKETWHSAATAIYCLMGSGQLLWDTVTGVNMTL |
| B/Gambia/G0068640/2012 | 2012 | LPSTIQMLTLFLTSGGVLLSLYVSASLSYLLYSNILLKFSPTEITAPTMPLDCANASNAHAVNRSATKGVTFLLPEPEWTYPRLSCPGSTFQKALLISPHRFGETKGNSAPLIIREPFIACGPNECKHFALTHYAAQPGGYYNGTRGDRNKLRHLISVKLGKIPTVENSIFHMAAWSGSACHDGKEWTYVGVDGPDNNALLKIKYGEAYTDTYHSYANNILRTQESACNCIGGNCYLMITDGSASGVSECRFLKIREGRIIKEIFPTGRVKHTEECTCGFASNETIECACRDNSYTAKRPFVKLNVETDTAEIRLMCTDTYLDTPRPDDGSITGPCESNGDKGSGGIKGGFVHQRMASKIGRWYSRTMSKTERKGMGLYVKYDGDPWTNSDALAFRGVMVSMKEPGWYSFGFEIKDKKCDVPCIGIEMVHDGGKETWHSAATAIYCLMGSGQLLWDTVTGVDMAL |
| B/Heilongjiang-Saertu/1171/2012 | 2012 | LPSTIQTLTLFLTSGGVLLSLYVSASLSYLLYSDILLKFSRTEITAPIMPLDCANASNVQAVNRSATKGVTLLLPEPEWTYPRLSCPGSTFQKALLISPHRFGETKGNSAPLIIREPFIACGPKECKHFALTHYAAQPGGYYNGTREDRNKLRHLISVKLGKIPTVENSIFHMAAWSGSACHDGREWTYIGVDGPDSNALLKIKYGEAYTDTYHSYAKNILRTQESACNCIGGDCYLMITDGPASGISECRFLKIREGRIIKEIFPTGRVKHTEECTCGFASNKTIECACRDNSYTAKRPFVKLNVETDTAEIRLMCTETYLDTPRPNDGSITGPCESNGDKGSGGIKGGFVHQRMASKIGRWYSRTMSKTKRMGMGLYVKYDGDPWTDSEALALSGVMVSMEEPGWYSFGFEIKDKKCDVPCIGIEMVHDGGKTTWHSAATAIYCLMGSGQLLWDTVTGVNMTL |
| B/HIROSHIMA/28/2012 | 2012 | LPSTIQTLTLFLTSGGVLLSLYVSASLSYLLYSDILLKFSQTEITAPIMPLDCANASNFQAVNRSAAKGVTLLLPEPEWTYPRLSCPGSTFQKALLISPHRFGEIKGNSAPLIIREPFIACGPTECKHFALTHYAAQPGGYYNGTREDRNKLRHLISVKLGKIPTVENSIFHMAAWSGSACHDGKEWTYIGVDGPDSNALLKIKYGEAYTDTYHSYAKNILRTQESACNCIGGDCYLMITDGPASGVSECRFLKIREGRIIKEIFPTGRVKHTEECTCGFASNKTIECACRDNRYTAKRPFVKLNVETDTAEIRLMCTETYLDTPRPNDGSITGPCESDGDKGSGGIKGGFVHQRMASKIGRWYSRTMSKTKRMGMGLYVKYDGDPWTDSEALALSGVMVSMEEPGWYSFGFEIKDKKCDVPCIGIEMVHDGGKTTWHSAATAIYCLMGSGQLLWDTVTGVDMAL |
| B/Hong Kong/3501/2012 | 2012 | LPSTIQTLTLFLTSGGVLLSLYVSASLSYLLYSDILLKFSPTEITAPTMPLDCANASNVQAVNRSATKGVTLLLPEPEWTYPRLSCPGSTFQKALLISPHRFGETKGNSAPLIIREPFIACGPNECKHFALTHYAAQPGGYYNGTRGDRNKLRHLISVKLGKIPTVENSIFHMAAWSGSACHDGKEWTYIGVDGPDNNALLKVKYGEAYTDTYHSYANKILRTQESACNCIGGNCYLMITDGSASGVSECRFLKIREGRIIKEIFPTGRVKHTEECTCGFASNKTIECACRDNRYTAKRPFVKLNVETDTAEIRLMCTDTYLDTPRPNDGSITGPCESDGDKGSGGIKGGFVHQRMKSKIGRWYSRTMSKTERMGMGLYVKYDGDPWADSDALAFSGVMVSMKEPGWYSFGFEIKDKKCDVPCIGIEMVHDGGKETWHSAATAIYCLMGSGQLLWDTVTGVDMAL |
| B/Hong Kong/3579/2012 | 2012 | LPSTIQTLTLFLTSGGVLLSLYVSASLSYLLYSDILLKFSQTEITAPIMPLDCANASNVQAVNRSAAKGVTLLLPEPEWTYPRLSCPGSTFQKALLISPHRFGEIKGNSAPLIIREPFIACGPTECKHFALTHYAAQPGGYYNGTREDRNKLRHLISVKLGKIPTVENSIFHMAAWSGSACHDGKEWTYIGVDGPDSNALLKIKYGEAYTDTYHSYAKNILRTQESACNCIGGDCYLMITDGPASGVSECRFLKIREGRIIKEIFPTGRVKHTEECTCGFASNKTIECACRDNRYTAKRPFVKLNVETDTAEIRLMCTETYLDTPRPNDGSITGPCESDGDKGSGGIKGGFVHQRMASKIGRWYSRTMSKTKRMGMGLYVKYDGDPWTDSEALALSGVMVSMEEPGWYSFGFEIKDKKCDVPCIGIEMVHDGGKTTWHSAATAIYCLMGSGQLLWDTVTGVDMAL |
| B/Hubei-Wuchang/1118/2012 | 2012 | LPSTIQTLTLFLTSGGVLLSLYVSASLSYLLYSDILLKFSRTEITAPTMPLDCANASKVQAVNRSATKGVTLLLPEPEWTYPRLSCPGSTFQKALLISPHRFGETKGNSAPLIIREPFIACGPKECKHFALTHYAAQPGGYYNGTREDRNKLRHLISVKLGKIPTVENSIFHMAAWSGSACHDGREWTYIGVDGPDSNALLKIKYGEAYTDTYHSYAKNILRTQESACNCIGGDCYLMITDGPASGISECRFLKIREGRIIKEIFPTGRVKHTEECTCGFASNKTIECACRDNSYTAKRPFVKLNVETDTAEIRLMCTETYLDTPRPNDGSITGPCESNGDKGSGGIKGGFVHQRMASKIGRWYSRTMSKTKRMGMGLYVKYDGDPWTDSEALALSGVMVSMEEPGWYSFGFEIKDKKCDVPCIGIEMVHDGGKTTWHSAATAIYCLMGSGQLLWDTVTGVNMTL |
| B/Laos/I057/2012 | 2012 | LPSTIQTLTLFLTSGGVLLSLYVSASLSYLLYSDILLKFSPTEITAPTMPLDCANASNVQAANRSATKGVTLLLPEPEWTYPRLSCPGSTFQKALLISPHRFGETKGNSAPLIIREPFIACGPNECKHFALTHYAAQPGGYYNGTRGDRNKLRHLISVKLGKIPTVENSIFHMAAWSGSACHDGKEWTYIGVDGPDNNALLKVKYGEAYTDTYHSYANKILRTQESACNCIGGNCYLMITDGSASGVSECRFLKIREGRIIKEIFPTGRVKHTEECTCGFASNKTIECACRDNSYTAKRPFVKLNVETDTAEIRLMCTDTYLDTPRPNDGSITGPCESNGDKGSGGIKGGFVHQRMESKIGRWYSRTMSKTERMGMGLYVKYDGDPWXDSDALAFSGVMVSMKEPGWYSFGFEIKDKKCDVPCIGIEMVHDGGKETWHSAATAIYCLMGSGQLLWDTVTGVDMAL |
| B/Laos/I118/2012 | 2012 | LPSTIQTLTLFLTSGGVLLSLYVSASLSYLLYSDILLKFSPTEITAPTMPLDCANASNVQAANRSATKGVTLLLPEPEWTYPRLSCPGSTFQKALLISPHRFGETKGNSAPLIIREPFIACGPNECKHFALTHYAAQPGGYYNGTRGDRNKLRHLISVKLGKIPTVENSIFHMAAWSGSACHDGKEWTYIGVDGPDNNALLKVKYGEAYTDTYHSYANKILRTQESACNCIGGNCYLMITDGSASGVSECRFLKIREGRIIKEIFPTGRVKHTEECTCGFASNKTIECACRDNSYTAKRPFVKLNXETDTAEIRLMCTDTYLDTPRPNDGSITGPCESNGDKGSGGIKGGFVHQRMESKIGRWYSRTMSKTERMGMGLYVKYDGDPWADSDALAFSGVMVSMKEPGWYSFGFEIKDKKCDVPCIGIEMVHDGGKETWHSAATAIYCLMGSGQLLWDTVTGVDMAL |
| B/MACAU/200052/2012 | 2012 | LPSTIQTLTLFLTSGGVLLSLYVSASLSYLLYSDILLKFPSTEITAPTMPLDCANASNVQAVNRSATKGVTLLLPEPEWTYPRLSCPGSTFQKALLISPHRFGETKGNSAPLIIREPFIACGPKECKHFALTHYAAQPGGYYNGTRGDRNKLRHLISVKLGKIPTVENSIFHMAAWSGSACHDGKEWTYIGVDGPDNNALLKIKYGEAYTDTYHSYANNILRTQESACNCIGGNCYLMITDGSASGVSECRFLKIREGRIIKEIFPTGRIQHTEECTCGFASNKTIECACRDNSYTAKRPFVKLNVETDTAEIRLMCTKTYLDTPRPEDGSITGPCESNGGKGSGGIKGGFVHQRMASKIGRWYSRTMSKTERKGMGLYVKYNGDPWADSDALVFSGVMVSMEEPGWYSFGFEIKDKKCDVPCIGIEMVHDGGKETWQSAATAIYCLMGSGQLLWDTVTGVNMTL |
| B/MACAU/200143/2012 | 2012 | LPSTIQTLTLFLTSGGVLLSLYVSASLSYLLYSDILLKFPSTEITAPTMPLDCANASNVQAVNRSATKGVTLLLPEPEWTYPRLSCPGSTFQKALLISPHRFGETKGNSAPLIIREPFIACGPKECKHFALTHYAAQPGGYYNGTRGDRNKLRHLISVKLGKIPTVENSIFHMAAWSGSACHDGKEWTYIGVDGPDNNALLKIKYGEAYTDTYHSYANNILRTQESACNCIGGNCYLMITDGSASGVSECRFLKIREGRIIKEIFPTGRIQHTEECTCGFASNKTIECACRDNSYTAKRPFVKLNVETDTAEIRLMCTKTYLDTPRPEDGSITGPCESNGGKGSGGIKGGFVHQRMASKIGRWYSRTMSKTERKGMGLYVKYNGDPWADSDALVFSGVMVSMEEPGWYSFGFEIKDKKCDVPCIGIEMVHDGGKETWHSAATAIYCLMGSGQLLWDTVTGVNMTL |
| B/Malaysia/U1267/2012 | 2012 | LPSTIQTLTLFLTSGGVLLSLYVSASLSYLLYSDILLKFSPTEITAPTMPLDCANASNVQAVNRSATKGVTLLLPEPEWTYPRLSCPGSTFQKALLISPHRFGETNGNSAPLIIREPFIACGPNECKHFALTHYAAQPGGYYNGTRGDRNKLRHLISVKLGKIPTVENSIFHMAAWSGSACHDGKEWTYIGVDGPDNNALLKVKYGEAYTDTYHSYANKILRTQESACNCIGGNCYLMITDGSASGVSECRFLKIREGRIIKEIFPTGRVKHTEECTCGFASNKTIECACRDNRYTAKRPFVKLNVETDTAEIRLMCTDTYLDTPRPNDGSITGPCESDGDEGSGGIKGGFVHQRMKSKIGRWYSRTMSKTERMGMGLYVKYDGDPWADSDALAFSGVMVSMKEPGWYSFGFEIKDKKCDVPCIGIEMVHDGGKETWHSAATAIYCLMGSGQLLWDTVTGVDMAL |
| B/Malaysia/U169/2012 | 2012 | LPSTIQTLTLFLTSGGVLLSLYVSASLSYLLYSDILLKFSRTEITAPIMPLDCANASNVQAVNRSATKGVTPLLPEPEWTYPRLSCPGSTFQKALLISPHRFGETKGNSAPLIIREPFIACGPKECKHFALTHYAAQPGGYYNGTREDRNKLRHLISVKLGKIPTVENSIFHMAAWSGSACHDGREWTYIGVDGPDSNALLKIKYGEAYTDTYHSYAKNILRTQESACNCIGGDCYLMITDGPASGISECRFLKIREGRIIKEIFPTGRVKHTEECTCGFASNKTIECACRDNSYTAKRPFVKLNVETDTAEIRLMCTETYLDTPRPNDGSITGPCESNGDEGSGGIKGGFVHQRMASKIGRWYSRTMSKTKRMGMGLYVKYDGDPWTDSEALALSGVMVSMEEPGWYSFGFEIKDKKCDVPCIGIEMVHDGGKTTWHSAATAIYCLMGSGQLLWDTVTGVNMTL |
| B/MASSACHUSETTS/02/2012 | 2012 | LPSTIQTLTLFLTSGGVLLSLYVSASLSYLLYSDILLKFSQTEITAPIMPLDCANASNVQAVNHSAAKGVTLLLPEPEWTYPRLSCPGSTFQKALLISPHRFGEIKGNSAPLIIREPFIACGPTECKHFALTHYAAQPGGYYNGTREDRNKLRHLISVKLGKIPTVENSIFHMAAWSGSACHDGKEWTYIGVDGPDSNALLKIKYGEAYTDTYHSYAKNILRTQESACNCIGGDCYLMITDGPASGVSECRFLKIREGRIIKEIFPTGRVKHTEECTCGFASNKTIECACRDNRYTAKRPFVKLNVETDTAEIRLMCTETYLDTPRPNDGSITGPCESDGDKGSGGIKGGFVHQRMASKIGRWYSRTMSKTKRMGMGLYVKYDGDPWTDSEALALSGVMVSMEEPGWYSFGFEIKDKKCDVPCIGIEMVHDGGKTTWHSAATAIYCLMGSGQLLWDTVTGVDMAL |
| B/New York/1331/2012 | 2012 | LPSTIQTLTLFLTSGGVLLSLYVSASLSYLLYSDILLKFSRTEITAPIMPLDCANASNVQAVNRSATKGVTPLLPEPEWTYPRLSCPGSTFQKALLISPHRFGETKGNSAPLIIREPFIACGPKECKHFALTHYAAQPGGYYNGTREDRNKLRHLISVKLGKIPTVENSIFHMAAWSGSACHDGREWTYIGVDGPDSNALLKIKYGEAYTDTYHSYAKNILRTQESACNCIGGDCYLMITDGSASGISECRFLKIREGRIIKEIFPTGRVKHTEECTCGFASNKTIECACRDNSYTAKRPFVKLNVETDTAEIRLMCTETYLDTPRPNDGSIAGPCESNGDEGSGGIKGGFVHQRMASKIGRWYSRTMSKTKRMGMGLYVKYDGDPWTDSEALALSGVMVSMEEPGWYSFGFEIKDKKCDVPCIGIEMVHDGGKTTWHSAATAIYCLMGSGQLLWDTVTGVNMTL |
| B/New York/1333/2012 | 2012 | LPSTIQTLTLFLTSGGVLLSLYVSASLSYLLYSDILLKFSRTEITAPIMPLDCANASNVQAVNRSATKGVTPLLPEPEWTYPRLSCPGSTFQKALLISPHRFGETKGNSAPLIIREPFIACGPKECKHFALTHYAAQPGGYYNGTREDRNKLRHLISVKLGKIPTVENSIFHMAAWSGSACHDGREWTYIGVDGPDSNALLKIKYGEAYTDTYHSYAKNILRTQESACNCIGGDCYLMITDGSASGISECRFLKIREGRIIKEIFPTGRVKHTEECTCGFASNKTIECACRDNSYTAKRPFVKLNVETDTAEIRLMCTETYLDTPRPNDGSIAGPCESNGDEGSGGIKGGFVHQRMASKIGRWYSRTMSKTKRMGMGLYVKYDGDPWTDSEALALSGVMVSMEEPGWYSFGFEIKDKKCDVPCIGIEMVHDGGKTTWHSAATAIYCLMGSGQLLWDTVTGVNMTL |
| B/New York/1341/2012 | 2012 | LPSTIQTLTLFLTSGGVLLSLYVSASLSYLLYSDILLKFSPTEITAPTMPLDCANASNVQAVNRSATKGVTLLLPEPEWTYPRLSCPGSTFQKALLISPHRFGETKGNSAPLIIREPFIACGPNECKHFALTHYAAQPGGYYNGTRGDRNKLRHLISVKLGKIPTVENSIFHMAAWSGSACHDGKEWTYIGVDGPDNNALLKVKYGEAYTDTYHSYANKILRTQESACNCIGGNCYLMITDGSASGVSECRFLKIREGRIIKEIFPTGRVKHTEECTCGFASNKTIECACRDNRYTAKRPFVKLNVETDTAEIRLMCTDTYLDTPRPNDGSITGPCESDGDKGSGGIKGGFVHQRMKSKIGRWYSRTMSKTERMGMGLYVKYDGDPWADSDALAFSGVMVSMKEPGWYSFGFEIKDKKCDVPCIGIEMVHDGGKETWHSAATAIYCLMGSGQLLWDTVTGVDMAL |
| B/Nicaragua/6546 05/2012 | 2012 | LPSTIQTLTLFLTSGGVLLSLYVSASLSYLLYSDILLKFSPTEITAQTMPLDCANASNVQAVNRSATKGVTLLPPEPEWTYPRLSCPGSTFQKALLISPHRFGETKGNSAPLIIREPFIACGPNECKHFALTHYAAQPGGYYNGTRGDRNKLRHLISVKLGKIPTVENSIFHMAAWSGSACHDGKEWTYIGVDGPDNNALLKVKYGEAYTDTYHSYANKILRTQESACNCIGGNCYLMITDGSASGVSECRFLKIREGRIIKEIFPTGRVKHTEECTCGFASNKTIECACRDNRYTAKRPFVKLNVETDTAEIRLMCTDTYLDTPRPNDGSITGPCESDGDKGSGGIKGGFVHQRMKSKIGRWYSRTMSKTERMGMGLYVKYDGDPWADSDALAFSGVMVSMKEPGWYSFGFEMKDKKCDVPCIGIEMVHDGGKETWHSAATAIYCLMGSGQLLWDTVTGVDMAL |
| B/Nicaragua/AGB2-14/2012 | 2012 | LPSTIQTLTLFLTSGGVLLSLYVSASLSYLLYSDILLKFSQTEITAPIMPLDCTNASNVQAVNRSAAKGVTLLLPEPEWTYPRLSCPGSTFQKALLISPHRFGEIKGNSAPLIIREPFIACGPTECKHFALTHYAAQPGGYYNGTREDRNKLRHLISVKLGKIPTVENSIFHMAAWSGSACHDGKEWTYIGVDGPDSNALLKIKYGEAYTDTYHSYAKNILRTQESACNCIGGDCYLMITDGPASGVSECRFLKIREGRIIREIFPTGRVKHTEECTCGFASNKTIECACRDNRYTAKRPFVKLNVETDTAEIRLMCTETYLDTPRPNDGSITGPCESDGDKGSGGIKGGFVHQRMASKIGRWYSRTMSKTKRMGMGLYVKYDGDPWTDSEALALSGVMVSMEEPGWYSFGFEIKDKKCDVPCIGIEMVHDGGKTTWHSAATAIYCLMGSGQLLWDTVTGVDMAL |
| B/Nicaragua/AGB2-29/2012 | 2012 | LPSTIQMLTLFLTSGGVLLSLYVSASLSYLLYSDILLKFSPTEITAPTMPLDCANASNVHAVNRSATKGVTFLLPEPEWTYPRLSCPGSTFQKALLISPHRFGETKGNSAPLIIREPFIACGPNECKHFALTHYAAQPGGYYNGTRGDRNKLRHLISVKLGKIPTVENSIFHMAAWSGSACHDGKEWTYIGVDGPDNNALLKIKYGEAYTDTYHSYANNILRTQESACNCIGGNCYLMITDGSASGVSECRFLKIREGRIIKEIFPTGRVKHTEECTCGFASNKTIECACRDNSYTAKRPFVKLNVETDTAEIRLMCTDTYLDTPRPDDGSITGPCESNGDKGSGGIKGGFVHQRMASKIGRWYSRTMSKTERMGMGLYVKYDGDPWTDSDALAFRGVMVSMKEPGWYSFGFEIKDKKCDVPCIGIEMVHDGGKETWHSAATAIYCLMGSGQLLWDTVTGVDMAL |
| B/Nicaragua/AGB2-48/2012 | 2012 | LPSTIQTLTLFLTSGGVLLSLYVSASLSYLLYSDILLKFSPTEITAPTRPLDCANASNVQAVNRSATKGVTLLLPEPEWTYPRLSCPGSTFQKALLISPHRFGETKGNSAPLIIREPFIACGPNECKHFALTHYAAQPGGYYNGTRGDRNKLRHLISVKLGKIPTVENSIFHMAAWSGSACHDGKEWTYIGVDGPDNNALLKVKYGEAYTDTYHSYANKILRTQESACNCIGGNCYLMITDGSASGVSECRFLKIREGRIIKEIFPTGRVKHTEECTCGFASNKTIECACRDNRYTAKRPFVKLNVETDTAEIRLMCTDTYLDTPRPNDGSITGPCESDGDKGSGGIKGGFVHQRMKSKIGRWYSRTMSKTERMGMGLYVKYDGDPWADSDALAFSGVMVSMKEPGWYSFGFEIKDKKCDVPCIGIEMVHDGGKETWHSAATAIYCLMGSGQLLWDTVTGVDMAL |
| B/Norway/1133/2012 | 2012 | LPSTIQTLTLFLTSGGVLLSLYVSASLSYLLYSDILLKFSPTEITAPTMPLDCANASTVQAVNRSATKGVTLLLPEPEWTYPRLSCPGSTFQKALLISPHRFGETKGNSAPLIIREPFIACGPNECKHFALTHYAAQPGGYYNGTRGDRNKLRHLISVKLGKIPTVENSIFHMAAWSGSACHDGKEWTYIGVDGPDNNALLKVKYGEAYTDTYHSYANKILRTQESACNCIGGNCYLMITDGSASGVSECRFLKIREGRIIKEIFPTGRVKHTEECTCGFASNKTIECACRDNRYTAKRPFVKLNVETDTAEIRLMCTDTYLDTPRPNDGSITGPCESDGDKGSGGIKGGFVHQRMKSKIGRWYSRTMSKTERMGMGLYVKYDGDPWADSDALAFSGVMVSMKEPGWYSFGFEIKDKKCDVPCIGIEMVHDGGKEIWHSAATAIYCLMGSGQLLWDTVTGVDMAL |
| B/Novosibirsk/RII01/2012 | 2012 | LPSTIQTLTLFLTSGGVLLSLYVSASLSYLLYSDILLKFSRTEITAPIMPLDCANASNVQAVNRSATKGVTLLLPEPEWTYPRLSCPGSTFQKALLISPHRFGETKGNSAPLIIREPFIACGPKECKHFALTHYAAQPGGYYNGTREDRNKLRHLISVKLGKIPTVENSIFHMAAWSGSACHDGREWTYIGVDGPDSNALLKIKYGEAYTDTYHSYAKNILRTQESACNCIGGDCYLMITDGPASGISECRFLKIREGRIIKEIFPTGRVKHTEECTCGFASNKTIECACRDNSYTAKRPFVKLNVETDTAEIRLMCTETYLDTPRPNDGSITGPCESNGDKGSGGIKGGFVHQRMASKIGRWYSRTMSKTKRMGMGLYVKYDGDPWTDSEALALSGVMVSMEEPGWYSFGFEIKDKKCDVPCIGIEMVHDGGKTTWHSAATAIYCLMGSGQLLWDTVTGVNMTL |
| B/Novosibirsk/RII03/2012 | 2012 | LPSTIQTLTLFLTSGGVLLSLYVSASLSYLLYSDILLKFSRTEITAPIMPLDCANASNVQAVNRSATKGVTLLLPEPEWTYPRLSCPGSTFQKALLISPHRFGETKGNSAPLIIREPFIACGPKECKHFALTHYAAQPGGYYNGTREDRNKLRHLISVKLGKIPTVENSIFHMAAWSGSACHDGREWTYIGVDGPDSNALLKIKYGEAYTDTYHSYAKNILRTQESACNCIGGDCYLMITDGPASGISECRFLKIREGRIIKEIFPTGRVKHTEECTCGFASNKTIECACRDNSYTAKRPFVKLNVETDTAEIRLMCTETYLDTPRPNDGSITGPCESNGDKGSGGIKGGFVHQRMASKIGRWYSRTMSKTKRMGMGLYVKYDGDPWTDSEALALSGVMVSMEEPGWYSFGFEIKDKKCDVPCIGIEMVHDGGKTTWHSAATAIYCLMGSGQLLWDTVTGVNMTL |
| B/Ontario/0010241/2012 | 2012 | LPSTIQMLTLFLTSGGVLLSLYVSASLSYLLYSDILLKFSPTEITAPTMPLDCANASNVHAVNRSATKGVTFLLPEPEWTYPRLSCPGSTFQKALLISPHRFGETKGNSAPLIIREPFIACGPNECKHFALTHYAAQPGGYYNGTRGDRNKLRHLISVKLGKIPTVENSIFHMAAWSGSACHDGKEWTYIGVDGPDNNALLKIKYGEAYTDTYHSYANNILRTQESACNCIGGNCYLMITDGSASGVSECRFLKIREGRIIKEILPTGRVKHTEECTCGFASNKTIECACRDNSYTAKRPFVKLNVETDTAEIRLMCTDTYLDTPRPDDGSITGPCESNGGKGSGGIKGGFVHQRMASKIGRWYSRTMSKTERKGMGLYVKYDGDPWTDSDALAFRGVMVSMKEPGWYSFGFEIKDKKCDVPCIGIEMVHDGGKETWHSAATAIYCLMGSGQLLWDTVTGVDMAL |
| B/Ontario/0020731/2012 | 2012 | LPSTIQTLTLFLTSGGVLLSLYVSASLSYLLYSDILLKFSPTEITAPTMPLDCANASNVQAVNRSATKGVTLLLPEPEWTYPRLSCPGSTFQKALLISPHRFGETKGNSAPLIIREPFIACGPNECKHFALTHYAAQPGGYYNGTRGDRNKLRHLISVKLGKIPTVENSIFHMAAWSGSACHDGKEWTYIGVDGPDNNALLKVKYGEAYTDTYHSYANKILRTQESACNCIGGNCYLMITDGSASGVSECRFLKIREGRIIKEIFPTGRVKHTEECTCGFASNKTIECACRDNRYTAKRPFVKLNVETDTAEIRLMCTDTYLDTPRPNDGSITGPCESDGDKGSGGIKGGFVHQRMKSKIGRWYSRTMSKTERMGMGLYVKYDGDPWADSDALAFSGVMVSMKEPGWYSFGFEIKDKKCDVPCIGIEMVHDGGKETWHSAATAIYCLMGSGQLLWDTVTGVDMAL |
| B/PERTH/16/2012 | 2012 | LPSTIQMLTLFLTSGGVLLSLYVSASLSYLLYSDILLKFSPTEITAPTMPLDCANASNVHAVNRSATKGVIFLLPEPEWTYPRLSCPGSTFQKALLISPHRFGETKGNSAPLIIREPFIACGPNECKHFALTHYAAQPGGYYNGTRGDRNKLRHLISVKLGKIPTVENSIFHMAAWSGSACHDGKEWTYIGVDGPDNNALLKIKYGEAYTDTYHSYANNILRTQESACNCIGGNCYLMITDGSASGVSECRFLKIREGRIIKEIFPTGRVKHTEECTCGFASNKTIECACRDNSYTAKRPFVKLNVETDTAEIRLMCTDTYLDTPRPDDGSITGPCESSGDKGSGGIKGGFVHQRMASKIGRWYSRTMSKTERKGMGLYVKYDGDPWTDSDALAFRGVMVSMKEPGWYSFGFEIKDKKCDVPCIGIEMVHDGGKETWHSAATAIYCLMGSGQLLWDTVTGVDMAL |
| B/Perth/212/2012 | 2012 | LPSTIQTLTLFLTSGGVLLSLYVSASLSYLLYSDILLKFSQTEITAPIMPLDCTNASNVQAVNRSAAKGVTLLLPEPEWTYPRLSCPGSTFQKALLISPHRFGEIKGNSAPLIIREPFIACGPTECKHFALTHYAAQPGGYYNGTREDRNKLRHLISVKLGKIPTVENSIFHMAAWSGSACHDGKEWTYIGVDGPDSNALLKIKYGEAYTDTYHSYAKNILRTQESACNCIGGDCYLMITDGPASGVSECRFLKIREGRIIKEIFPTGRVKHTEECTCGFASNKTIECACRDNRYTAKRPFVKLNVETDTAEIRLMCTETYLDTPRPNDGSITGPCESDGDKGSGGIKGGFVHQRMASKIGRWYSRTMSKTKRMGMGLYVKYDGDPWTDSEALALSGVMVSMEEPGWYSFGFEIKDKKCDVPCIGIEMVHDGGKTTWHSAATAIYCLMGSGQLLWDTVTGVDMAL |
| B/Perth/68/2012 | 2012 | LPSTIQTLTLFLTSGGVLLSLYVSASLSYLLYSDILLKFSRTEITAPIMPLDCANASNVQAVNRSATKGVTPLLPEPEWTYPRLSCPGSTFQKALLISPHRFGETKGNSAPLIIREPFIACGPKECKHFALTHYAAQPGGYYNGTREDRNKLRHLISVKLGKIPTVENSIFHMAAWSGSACHDGREWTYIGVDGPDSNALLKIKYGEAYTDTYHSYAKNILRTQESACNCIGGDCYLMITDGPASGISECRFLKIREGRIIKEIFPTGRVKHTEECTCGFASNKTIECACRDNSYTAKRPFVKLNVETDTAEIRLMCTETYLDTPRPNDGSITGPCESNGDEGSGGIKGGFVHQRMASKIGRWYSRTMSKTKRMGMGLYVKYDGDPWTDSEALALSGVMVSMEEPGWYSFGFEIKDKKCDVPCIGIEMVHDGGKTTWHSAATAIYCLMGSGQLLWDTVTGVNMTL |
| B/Roma/01/2012 | 2012 | LPSTIQTLTLFLTSGGVLLSLYVSASLSYLLYSDILLKFSQTEITAPIMPLDCANASSVQAVNHSAAKGVTLLLPEPEWTYPRLSCPGSTFQKALLISPHRFGEIKGNSAPLIIREPFIACGPTECKHFALTHYAAQPGGYYNGTREDRNKLRHLISVKLGKIPTVENSIFHMAAWSGSACHDGKEWTYIGVDGPDSNALLKIKYGEAYTDTYHSYAKNILRTQESACNCIGGDCYLMITDGPASGVSECRFLKIREGRIIKEIFPTGRVKHTEECTCGFASNKTIECACRDNRYTAKRPFVKLNVETDTAEIKLMCTETYLDTPRPNDGSITGPCESDGDKGSGGIKGGFVHQRMASKIGRWYSRTMSKTKRMGMGLYVKYDGDPWTDSEALALSGVMVSMEEPGWYSFGFEIKDKKCDVPCIGIEMVHDGGKTTWHSAATAIYCLMGSGQLLWDTVTGVDMAL |
[truncated: 232,335 more chars]
